# Astrobiological implications of the stability and reactivity of peptide nucleic acid (PNA) in concentrated sulfuric acid

**DOI:** 10.1126/sciadv.adr0006

**Published:** 2025-03-26

**Authors:** Janusz J. Petkowski, Sara Seager, Maxwell D. Seager, William Bains, Nittert Marinus, Mael Poizat, Chad Plumet, Jim van Wiltenburg, Ton Visser, Martin Poelert

**Affiliations:** ^1^ Faculty of Environmental Engineering, Wroclaw University of Science and Technology, 50-370 Wroclaw, Poland.; ^2^ JJ Scientific, Mazowieckie, Warsaw 02-792, Poland.; ^3^ Department of Earth, Atmospheric and Planetary Sciences, Massachusetts Institute of Technology, 77 Massachusetts Avenue, Cambridge, MA 02139, USA.; ^4^ Department of Physics, Massachusetts Institute of Technology, 77 Massachusetts Avenue, Cambridge, MA 02139, USA.; ^5^ Department of Aeronautics and Astronautics, Massachusetts Institute of Technology, 77 Massachusetts Avenue, Cambridge, MA 02139, USA.; ^6^ Nanoplanet Consulting, Concord, MA 01742, USA.; ^7^ Department of Chemistry and Biochemistry, Worcester Polytechnic Institute, Worcester, MA 01609, USA.; ^8^ School of Physics & Astronomy, Cardiff University, 4 The Parade, Cardiff CF24 3AA, UK.; ^9^ Symeres Netherlands BV, Kerkenbos 1013, 6546 BB Nijmegen, Netherlands.

## Abstract

Recent renewed interest regarding the possibility of life in the Venusian clouds has led to new studies on organic chemistry in concentrated sulfuric acid. However, life requires complex genetic polymers for biological function. Therefore, finding suitable candidates for genetic polymers stable in concentrated sulfuric acid is a necessary first step to establish that biologically functional macromolecules can exist in this environment. We explore peptide nucleic acid (PNA) as a candidate for a genetic-like polymer in a hypothetical sulfuric acid biochemistry. PNA hexamers undergo between 0.4 and 28.6% degradation in 98% (w/w) sulfuric acid at ~25°C, over the span of 14 days, depending on the sequence, but undergo complete solvolysis above 80°C. Our work is the first key step toward the identification of a genetic-like polymer that is stable in this unique solvent and further establishes that concentrated sulfuric acid can sustain a diverse range of organic chemistry that might be the basis of a form of life different from Earth’s.

## Teaser

PNA, a genetic-like polymer, persists in concentrated sulfuric acid at room temperature, relevant for life’s potential on Venus.

## INTRODUCTION

Organic chemistry in concentrated sulfuric acid is an understudied yet unexpectedly rich field in which there has been a recent renewed interest to support the notion that complex organic molecules can survive in such a harsh environment (*1*–*3*). Work by Spacek *et al.* (*1*) and Spacek and Benner (*4*, *5*) demonstrated that a rich organic chemistry can spontaneously arise in concentrated sulfuric acid from simple precursors such as formaldehyde or carbon monoxide. Our group has measured the stability of nucleic acid bases (*2*, *6*) and amino acids (*3*) and observed the formation of lipid vesicles (*7*) in concentrated sulfuric acid at room temperature (RT). Older work dates back many decades, before people knew that the Venus clouds are composed of concentrated sulfuric acid [e.g., (*8*–*12*)]. The renewed interest in the organic chemistry of sulfuric acid is motivated by the speculation of the potential habitability of Venus, not at the 700-K surface, but in the cloud layers located at 48- to 60-km altitudes, where temperatures match those found on Earth’s surface [e.g., (*13*–*21*)]. While complex organic chemistry is not life, its potential existence in a planetary environment is a required foundation for habitability (*22*).

The stability of simple organic molecules in recent studies is promising, but life requires more structurally complex molecules for biological function, especially complex polymers. The reliance on complex polymers, particularly as a molecular basis for genetics with functional properties analogous to RNA and DNA, is expected to be a universal feature of all life, no matter its chemical makeup (*22*, *23*). If life requires genetic polymers to exist, then finding suitable candidates for genetic polymers that are stable in concentrated sulfuric acid is a necessary step to establish that the possibility of life in concentrated sulfuric acid cannot be ruled out.

**Fig. 1.**
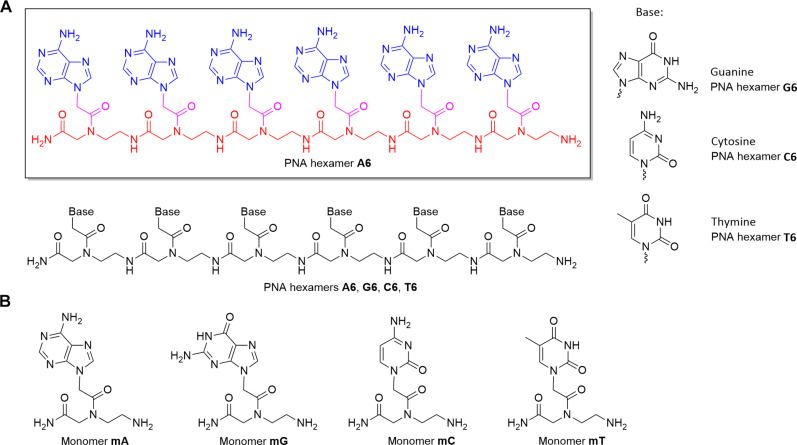
PNA hexamers and monomers. (**A**) PNA hexamers composed of six identical, consecutive units of nucleic acid bases: adenine (**A6**), guanine (**G6**), cytosine (**C6**), and thymine (**T6**). PNA backbone (AEG) residues are colored in red, the acetyl linker residues are in pink, and the nucleic acid bases are in blue. (**B**) Structures of PNA monomers, **mA**, **mG**, **mC**, and **mT**.

We have previously shown that glycylglycine (Gly-Gly), a dipeptide composed of two glycine amino acid residues, is stable in 98% (w/w) concentrated sulfuric acid for many months (*24*, *25*). The remarkable stability of the Gly-Gly dipeptide opens the possibility that a molecular motif similar to the Gly-Gly dipeptide could serve as a backbone for genetic polymer stable in 98% (w/w) sulfuric acid. One such potential candidate is peptide nucleic acid (PNA) (*26*). PNA has a *N*-(2-aminoethyl)glycine (AEG) backbone that is closely structurally related to the Gly-Gly dipeptide. The AEG backbone connects to a base via a tertiary amide bond in an acetyl group linker (Fig. 1). While PNA does not occur naturally in known life today, it has been hypothesized as a first genetic polymer for life on Earth [e.g., (*27*–*30*)]. PNA will tightly and specifically interact with DNA and RNA, and because of this feature, it is widely used as a DNA analog in biomedical research, including diagnostics, antisense therapy, and other molecular biological applications (*31*). Therefore, PNA is of high relevance for astrobiology and planetary science.

To explore PNA as a potential candidate for a genetic-like polymer of a hypothetical sulfuric acid biochemistry, we test the stability and reactivity of four 6–nt (nucleotide)–long single strands of PNA (PNA hexamers) in 98% (w/w) sulfuric acid. We use liquid chromatography–mass spectrometry (LC-MS) and ^1^H nuclear magnetic resonance (NMR) spectroscopy to assess the stability of the PNA molecules in liquid 98% (w/w) sulfuric acid at various temperatures over timescales of hours, days, and weeks.

## RESULTS

Our main result is that PNA homohexamers, as well as PNA monomers, show less than 28.6% degradation in 98% (w/w) sulfuric acid at RT (18° to 25°C) for at least 14 days. Hexamers do, however, undergo rapid solvolysis at temperatures above 80°C. We find that PNA solvolysis proceeds by cleavage of a single tertiary amide bond in an acetyl group linker. The solvolysis yields two distinct products that appear to be stable to further degradation.

### PNA hexamers are persistent in 98% (w/w) sulfuric acid at RT

**Fig. 2.**
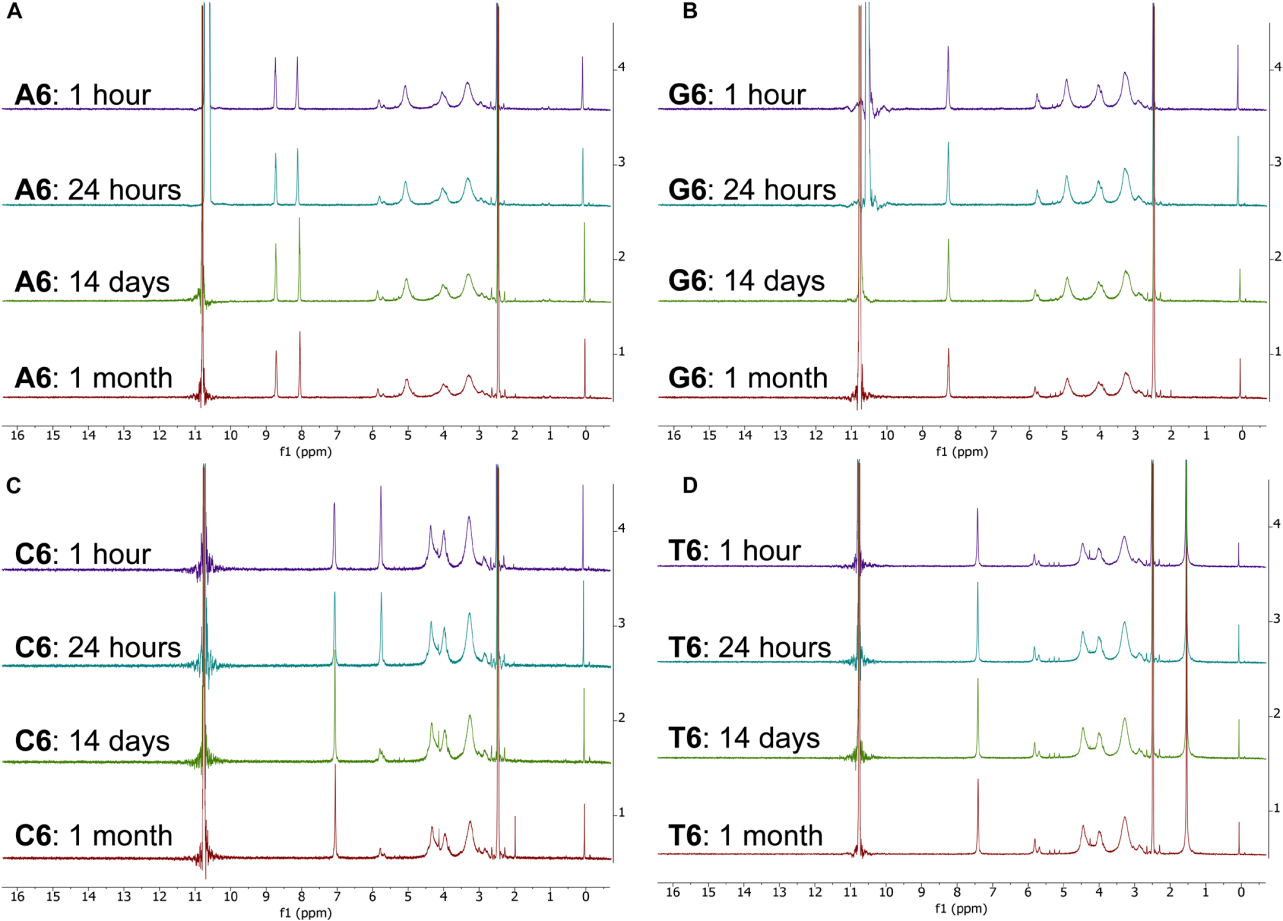
Comparison of ^1^H NMR spectra of PNA hexamers A6, G6, C6, and T6 in 98% D_2_SO_4_/2% D_2_O (by weight) for four different time periods at RT. The intensity (*y* axis) is shown as a function of spectral shift in parts per million (ppm). We show each PNA hexamer NMR spectrum in an individual subfigure (**A** to **D**). Within each subfigure, we compare the NMR spectra collected after 1-hour (purple)–, 24-hour (teal)–, 14-day (green)–, and 1-month (red)–long incubation. The large peak around 11 ppm corresponds to the residual protons in the D_2_SO_4_ solvent. All spectra, with a possible exception of C6 (see main text), are consistent with the hexamers being stable and the structure not being substantially affected by the concentrated sulfuric acid solvent at RT for a month. The spectra at four different time periods overlap closely, demonstrating an overall stability of the PNA hexamers in 98% (w/w) sulfuric acid solvent at RT.

We used LC-MS and ^1^H NMR to study the stability of four hexamer PNAs composed of six identical, consecutive units containing nucleic acid bases: adenine (**A6**), guanine (**G6**), cytosine (**C6**), and thymine (**T6**), as well as the PNA monomers **mA**, **mG**, **mC**, and **mT** (Fig. 1). We show that all four PNA hexamers undergo only limited degradation in 98% (w/w) sulfuric acid at RT for at least 14 days (Fig. 2) but undergo rapid solvolysis at high temperature (>80°C).

We assess the stability of the PNA hexamers via LC-MS by measuring the fraction of the original hexamers’ degradation after 1 hour, 24 hours, and 14 days of incubation in 98% (w/w) sulfuric acid, as compared to the sample measured in methanol (MeOH). The LC-MS analysis shows all four hexamers at the expected retention times with correct molecular masses (see Table 1 and the Supplementary Materials). In most of LC-MS measurements, we see only limited degradation at RT after 14-day-long incubation of PNA hexamers in 98% (w/w) sulfuric acid (Table 2). In some cases, e.g., in one replicate of (**A6**), we do, however, see a considerable degradation (<28.6%) (Table 2 and see discussion below).

**Table 1.** Example LC-MS results. The retention times and mass/charge ratio (*m*/z) of PNA hexamers **A6**, **G6**, **C6**, and **T6** in 98% (w/w) sulfuric acid after 1 hour, 24 hours, and 14 days at RT are determined by LC-MS [buffered with 3 M ammonium acetate (NH_4_OAc)].

PNAs	Retention time (min)/*m*/*z* after 1 hour	Retention time (min)/*m*/*z* after 24 hours	Retention time (min)/*m*/*z* after 14 days
Adenine hexamer **A6**	4.86/835.6	4.83/835.8	4.96/835.1
Guanine hexamer **G6**	4.83/883.7	4.78/883.2	4.85/883.0
Cytosine hexamer **C6**	4.45/762.7	4.42/763.0	4.55/763.2
Thymine hexamer **T6**	9.82/807.8	9.83/807.9	9.83/807.9

**Table 2.** Assessment of the stability of PNA hexamers A6, G6, C6, and T6 in 98% (w/w) sulfuric acid at RT. The samples are measured after 1 hour, 24 hours, and 14 days at RT [in triplicate (1/2/3)] as determined by LC-MS (buffered with 3 M NH_4_OAc). Starting purity has been measured in MeOH.

PNAs	Starting purity	Peak area after 1 hour (%)	Peak area after 24 hours (%)	Peak area after 14 days (%)
Adenine hexamer **A6**	99.8%	97.2%/99.2%/99.3%	*98.4%/98.7%/92.9%	97.2%/97.5%/71.2%
Guanine hexamer **G6**	98.5%	96.3%/96.9%/97.8%	*97.4%/*97.2%/97.6%	85.2%/82.4%/97.0%
Cytosine hexamer **C6**	98.2%	96.8%/97.8%/97.8%	*99.1%/96.9%/*98.0%	86.7%/93.3%/97.8%
Thymine hexamer **T6**	75.8%	69.1%/71.2%/74.4%	70.6%/69.8%/74.0%	65.0%/64.6%/63.5%

*Note that in few instances, the peak area recorded after 24 hours can have a slightly larger value than the value recorded after 1 hour. This discrepancy can be attributed to an inefficient solubility of the sample after 1-hour incubation at RT, followed by complete dissolution of PNA hexamers after 24-hour incubation in 98% (w/w) sulfuric acid.

We confirm the results of the LC-MS PNA hexamer stability assay with qualitative ^1^H NMR spectroscopic measurements in 98% (w/w) sulfuric acid [98% D_2_SO_4_/2% D_2_O by weight with 10% (v/v) dimethyl sulfoxide (DMSO)–*d*_6_]. The ^1^H NMR spectra of all four hexamers change only to a small degree after 1-month incubation in 98% (w/w) sulfuric acid at RT (Fig. 2). The spectra collected after 1-hour incubation overlap closely with the spectra collected after 24 hours, 14 days, and 1 month, suggesting that, in all four cases, there is very little degradation of PNA hexamers after 1-month incubation in 98% (w/w) sulfuric acid at RT (Fig. 2). We note, however, that the ^1^H NMR results are qualitative. In the LC-MS experiments, we see an unexplained sequence-independent variation (0.4 to 28.6%) in the degree of PNA degradation (Table 2). We note that organic impurities in individual samples could be responsible for this variability in PNA stability. Reactive organic impurities could promote reactivity of PNA in 98% (w/w) sulfuric acid. Such runaway, often autocatalytic, reactions producing complex organics in concentrated sulfuric acid have been known in industrial processes [e.g., (*9*)].

We identify the ^1^H NMR signal of all of the aromatic protons of purine and pyrimidine rings in all four tested hexamers after prolonged incubation in 98% (w/w) D_2_SO_4_. As expected, the ^1^H signals show around 6 to 9 parts per million (ppm), in the aromatic region of the NMR spectrum. The chemical shifts corresponding to the aromatic protons are consistent with our previous study (*2*) and confirm the stability of the nucleic acid base rings in 98% (w/w) sulfuric acid. We note that, over time, the ^1^H NMR signal corresponding to the H5 hydrogen (~5.8 ppm) in cytosine splits and broadens (Fig. 2). The splitting and broadening of the H5 peak indicate an exchange of the H5 proton of the pyrimidine ring with the D_2_SO_4_ deuterium (i.e., H/D exchange) and are not a sign of instability of the pyrimidine ring or the cytosine hexamer **C6** as a whole. Such a H/D exchange is known to happen in acidic solutions (*32*). We observe similar behavior of pyrimidine nucleic acid bases incubated over a long period of time in concentrated sulfuric acid before (*6*). We note that the LC-MS analysis of the NMR sample of the **C6** hexamer shows the **C6** hexamer at the expected retention time and mass analysis confirms the incorporation of six deuterium atoms [[M + 2H]^2+^ ion: mass/charge ratio (*m*/z), 766.0] into the **C6** structure (see the Supplementary Materials).

The aliphatic region of the ^1^H NMR spectrum (~0 to 6 ppm) also remains largely unchanged over time, confirming the stability and structural integrity of the linker and backbone regions of all four PNA hexamers at RT (Fig. 2). We note that the peaks in the aliphatic region of the ^1^H NMR spectrum are broad or give complicated multiplets. These characteristics of the aliphatic region of the spectrum indicate that all tested PNA hexamers (and all PNA monomers) have multiple stable rotamers (conformational isomers) that exist simultaneously in the 98% (w/w) sulfuric acid solution. Such a conformational diversity of PNA hexamers is not a sign of their chemical instability.

### Solvolysis of the PNA hexamers and monomers in 98% (w/w) sulfuric acid at high temperature

In contrast to their overall stability at RT, the hexamers undergo rapid solvolysis at elevated temperatures. The LC-MS stability assay at 50°C shows that the pyrimidine hexamers [cytosine (**C6**) and thymine (**T6**)] are more susceptible to solvolysis in 98% (w/w) sulfuric acid than their purine counterparts [adenine (**A6**) and guanine (**G6**)] (Table 3). For example, the degradation of the **C6** hexamer after 24-hour incubation in 98% (w/w) sulfuric acid at 50°C is as high as ~60%, while for **G6** hexamer, the most stable of the four hexamers, the degradation is only ~7% (Table 3). We observe full degradation (solvolysis) of all four hexamers after 24-hour incubation in 98% (w/w) sulfuric acid at 80°C (Table 4).

**Table 3.** Assessment of the stability of PNA hexamers A6, G6, C6, and T6 in 98% (w/w) sulfuric acid at 50°C. The samples are measured after 1 and 24 hours at 50°C in duplicate (1/2) as determined by LC-MS (buffered with 3 M NH_4_OAc). Starting purity has been measured in MeOH.

PNAs	Starting purity	Peak area after 1 hour (%)	Peak area after 24 hours (%)
Adenine hexamer **A6**	99.8%	97.4%/97.3%	75.4%/64.8%
Guanine hexamer **G6**	98.5%	96.8%/96.5%	89.9%/82.7%
Cytosine hexamer **C6**	98.2%	97.7%/94.6%	36.7%/36.5%
Thymine hexamer **T6**	75.8%	65.0%/63.7%	26.5%/28.9%

**Table 4.** Assessment of the stability of PNA hexamers A6, G6, C6, and T6 in 98% (w/w) sulfuric acid at 80°C. The samples are measured after 1 and 24 hours at 80°C in duplicate (1/2) as determined by LC-MS (buffered with 3 M NH_4_OAc). Starting purity has been measured in MeOH.

PNAs	Starting purity	Peak area after 1 hour (%)	Peak area after 24 hours (%)
Adenine hexamer **A6**	99.8%	39.8%/28.9%	Full degradation
Guanine hexamer **G6**	98.5%	68.5%/63.9%	Full degradation
Cytosine hexamer **C6**	98.2%	Full degradation	Full degradation
Thymine hexamer **T6**	75.8%	Full degradation	Full degradation

**Fig. 3.**
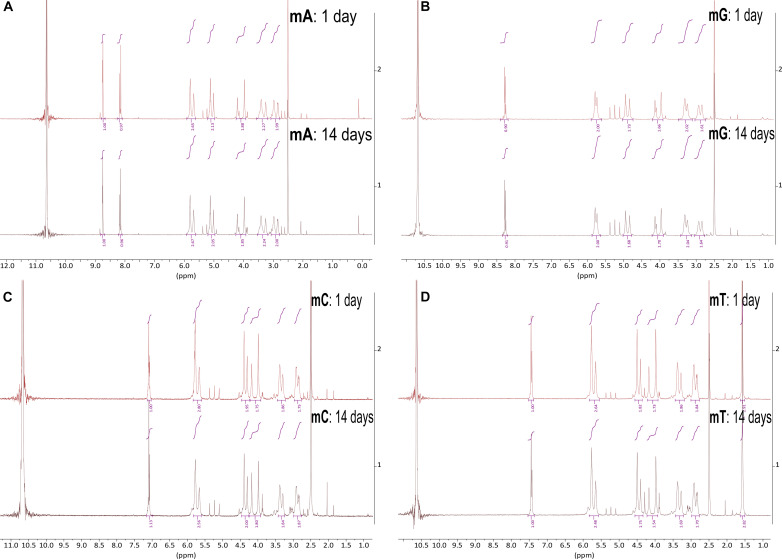
Comparison of ^1^H NMR spectra of PNA monomers mA, mG, mC, and mT in 98% (w/w) sulfuric acid for two different time periods at RT. The intensity (*y* axis) is shown as a function of spectral shift in parts per million. Each PNA monomer NMR spectrum is shown in an individual subfigure. Within each subfigure (**A** to **D**), we compare the NMR spectra collected after 24-hour (top)– and 14-day (bottom)–long incubation. We dissolved all monomers in 98% D_2_SO_4_/2% D_2_O (by weight) with DMSO-*d*_6_ as a reference and at RT. The large peak around 11 ppm corresponds to the residual protons in the D_2_SO_4_ solvent. All peaks are consistent with the molecules being stable and the overall structure not being substantially affected by the concentrated sulfuric acid solvent at RT. The spectra at two different time periods overlap closely, demonstrating an overall stability of the PNA monomers in 98% (w/w) sulfuric acid solvent at RT.

**Fig. 4.**
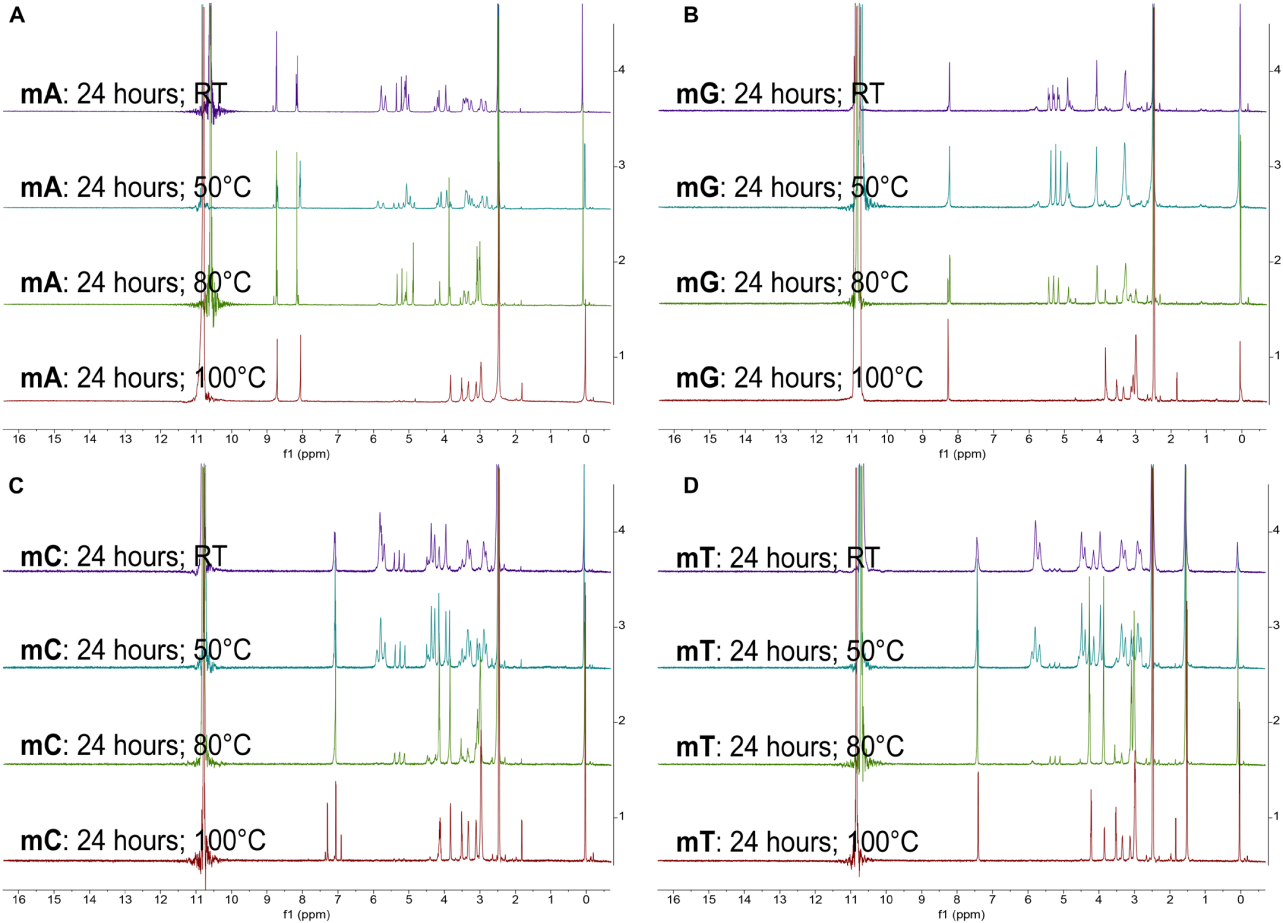
Comparison of ^1^H NMR spectra of PNA monomers mA, mG, mC, and mT in 98% D_2_SO_4_/2% D_2_O (by weight) after 24-hour incubation at four different temperatures. The intensity (*y* axis) is shown as a function of spectral shift in parts per million. We show each PNA monomer NMR spectrum in an individual subfigure (**A** to **D**). Within each subfigure, we compare the NMR spectra collected after 24-hour-long incubation at four different temperatures: RT (purple), 50°C (teal), 80°C (green), and 100°C (red). Because of shimming issues, the measurement of the NMR spectrum of **mG** (24 hours at 100°C) required additional DMSO-*d*_6_ in the solution. The large peak around 11 ppm corresponds to the residual protons in the D_2_SO_4_ solvent. All PNA monomers undergo rapid solvolysis at high temperature (>80°C) in 98% (w/w) sulfuric acid.

To understand the mechanism and the resulting products of the solvolysis of PNA in 98% (w/w) sulfuric acid at high temperature (>80°C), we perform a series of ^1^H NMR experiments on PNA monomers (**mA**, **mG**, **mC**, and **mT**) at RT, 50°, 80°, and 100°C. As expected, all four monomers are stable at RT for at least 2 weeks (Fig. 3), but they do undergo solvolysis in 98% (w/w) sulfuric acid at higher temperatures (Fig. 4).

**Fig. 5.**
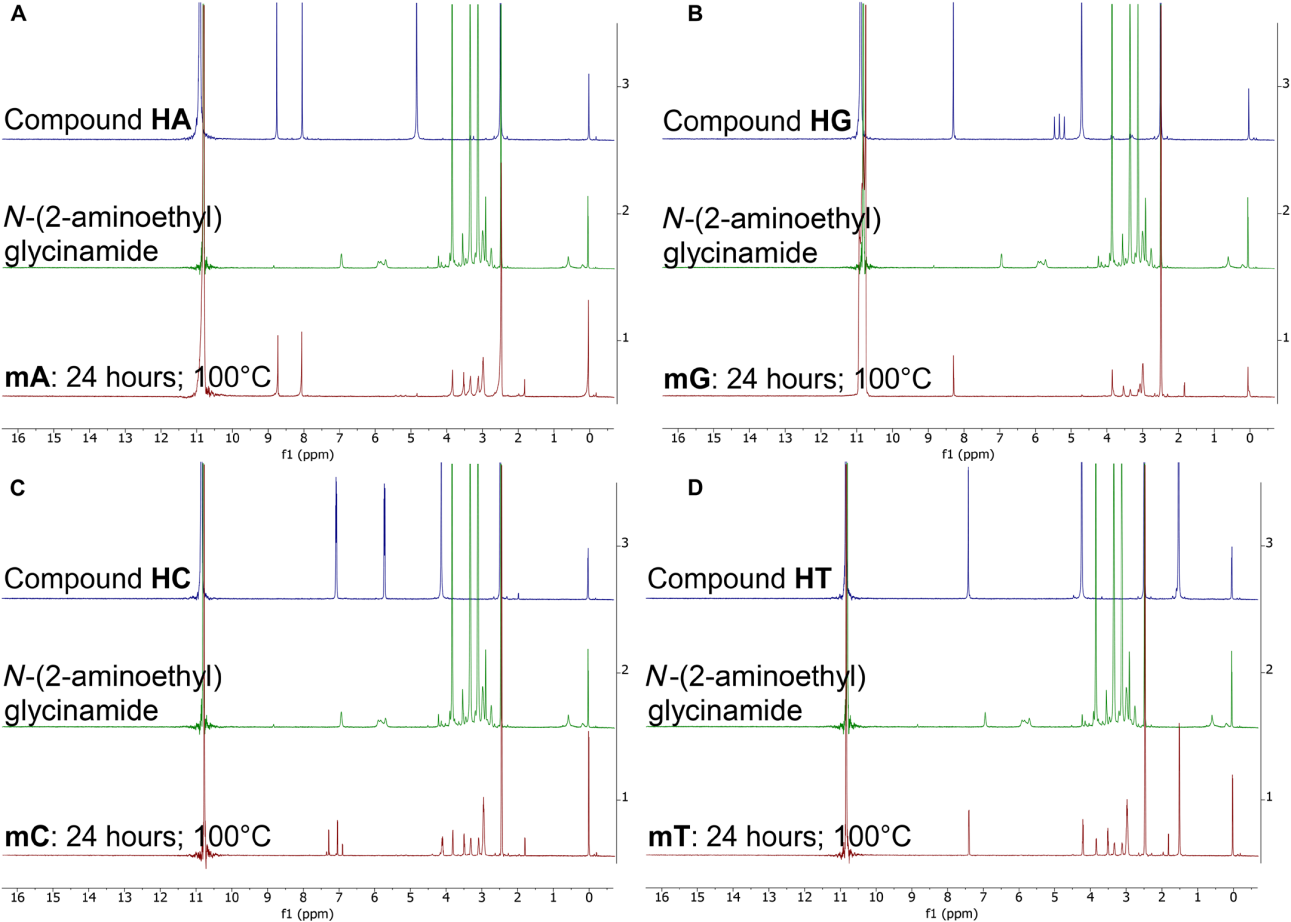
Solvolysis of PNA monomers mA, mG, mC, and mT in 98% D_2_SO_4_/2% D_2_O (by weight) after 24-hour incubation at 100°C. The intensity (*y* axis) is shown as a function of spectral shift in parts per million. We show each NMR spectrum in an individual subfigure (**A** to **D**). Within each subfigure, we compare the NMR spectra of native solvolysis products, **HA**, **HG**, **HC**, and **HT** (purple) and *N*-(2-aminoethyl)glycinamide (green), to NMR spectra of individual PNA monomers (**mA**, **mG**, **mC**, and **mT**) collected after 24-hour-long incubation at 100°C (red). The large peak around 11 ppm corresponds to the residual protons in the D_2_SO_4_ solvent. All PNA monomers undergo rapid solvolysis at 100°C in 98% (w/w) sulfuric acid with a release of the **HA**, **HG**, **HC**, **HT**, and *N*-(2-aminoethyl)glycinamide products that, with a possible exception of **mC**, appear to be stable to further reactivity.

**Fig. 6.**
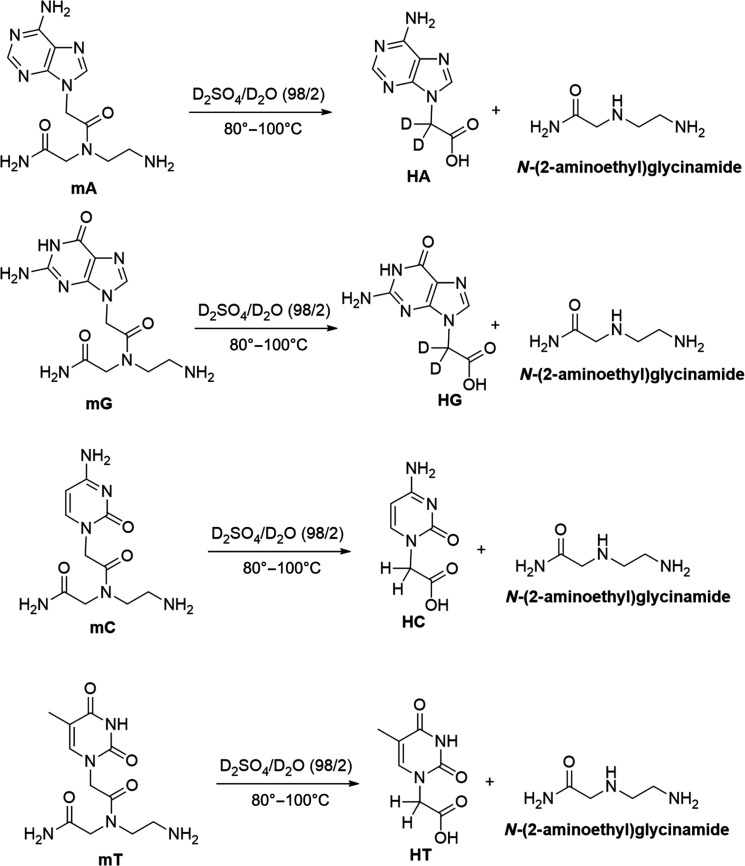
Schematic of the hypothesized solvolysis of PNA monomers in 98% (w/w) sulfuric acid. PNA monomers undergo decomposition in 98% sulfuric acid at temperatures above 80°C, which results in the solvolysis of the tertiary amide bond of the monomer and the release of an acetic acid derivative of nucleobases (**HA**, **HG**, **HC**, and **HT**, respectively) and *N*-(2-aminoethyl)glycinamide.

Heating PNA monomers in 98% sulfuric acid above 80°C results in the solvolysis of the tertiary amide bond and the release of two products, an acetic acid derivative of nucleobases (**HA**, **HG**, **HC**, and **HT**, respectively) and *N*-(2-aminoethyl)glycinamide (Figs. 5 and 6). The identity of the two solvolysis products is shown by a comparison of the ^1^H NMR spectra of the monomers incubated for 24 hours at 100°C in 98% (w/w) sulfuric acid to the ^1^H NMR spectra of pure solvolysis products, **HA**, **HG**, **HC**, **HT**, and *N*-(2-aminoethyl)glycinamide. The matching ^1^H NMR spectra confirm that the instability of the PNA in 98% (w/w) sulfuric acid results from the solvolysis of the single tertiary amide bond connecting the *N*-(2-aminoethyl)glycinamide residue to the acetyl nucleobase of PNA (Fig. 5). In more detail, the ^1^H NMR signals in the region between 3 and 4 ppm of the product monomers incubated at 100°C for 24 hours do match the spectra of the pure *N*-(2-aminoethyl)glycinamide, despite some impurities present in the sample (the starting purity of the glycinamide was only 95%). The aromatic and the acetate group signals also match the hydrolysis products **HA**, **HG**, **HC**, and **HT**, except for cytosine that displays few additional aromatic signals around 7 ppm. These additional signals suggest that cytosine PNA monomer undergoes further reactivity at 100°C that goes beyond the solvolysis of the tertiary amide bond. This result agrees with our LC-MS analysis, which shows that the cytosine PNA hexamer **C6** is the least stable of the four (Tables 2 to 4). Our previous work on the stability of nucleic acid bases in concentrated sulfuric acid did not explore these high temperatures (*2*, *6*).

We note that the NMR spectra of purine compounds **mA** and **mG** show no signals for the aliphatic protons in alpha position of the nucleic base, suggesting efficient deuteration of the carbon atom at this position. Pyrimidine compounds **mC** and **mT** do not seem to be affected by this deuteration, and the ^1^H NMR signals fully match the solvolysis products **HC** and **HT** at this position (Figs. 5 and 6). We leave the detailed investigation of the reasons behind the apparent sequence-dependent variability in the degradation rate of different PNA hexamers and monomers as part of future work focusing on identifying stable variants of PNA that survive temperatures above 50°C.

## DISCUSSION

We show an unexpectedly high stability of single strands of PNA hexamers in concentrated sulfuric acid at RT. By demonstrating the stability of a polymer in 98% (w/w) sulfuric acid that is structurally related to DNA and is known to interact specifically with nucleic acids, we have taken a substantial step forward in exploring the potential of concentrated sulfuric acid as a solvent that could support the complex chemistry needed for life and, hence, the potential habitability of the Venus cloud environment. Our recent work describes how concentrated sulfuric acid fulfils all the chemical requirements to be a solvent for life (*33*). Concentrated sulfuric acid as a planetary solvent could be one of the most common liquids in the Galaxy (*34*).

The carbonyl backbone of the PNA is structurally and functionally very well suited to provide the basis for a genetic polymer that can function in concentrated sulfuric acid solvent. A permanent repeating charge, no matter if negative (as phosphates in DNA in water) or positive (as protonated carbonyls in concentrated sulfuric acid), is likely a universal requirement for any genetic polymer of life regardless of its biochemical makeup (*35*–*37*) [but see also (*38*)]. The PNA backbone, while not charged in water, is expected to be permanently positively charged in concentrated sulfuric acid due to stable protonation of the carbonyl groups (*39*–*41*). The protonation of carbonyl groups gives the PNA polymer a permanent repeating positive charge in concentrated sulfuric acid. Thus, in contrast to water at pH 7, the PNA AEG backbone conforms to the structural requirements of the polyelectrolyte theory of the gene in concentrated sulfuric acid (*35*–*37*).

We aim to synthesize a genetic polymer that is stable in the aggressive solvent concentrated sulfuric acid, and this work is a substantial, informative step forward. Our findings of the instability of PNA at temperatures higher than 50°C mean that PNA on its own cannot be the genetic polymer for planets with liquid concentrated sulfuric acid where the environmental temperature sometimes exceeds 50°C, as it does in the clouds of Venus. Moreover, we have based this prototype polymer on the nucleic acid bases used by terrestrial life: adenine, thymine, guanine, and cytosine. In concentrated sulfuric acid, these bases will be protonated differently from those in water, which is likely to interfere with hydrogen bonding and, hence, a double helix structure. A true genetic polymer for sulfuric acid would, therefore, probably require different bases.

We emphasize that our work presented here focuses on the chemical stability of single strands of PNA in concentrated sulfuric acid only and not on their potential genetic function. In particular, it is very likely that the protonation and tautomeric forms of the bases will be different in concentrated sulfuric acid than in water, and consequently classical “Watson:Crick” base pairing might not be possible between complementary PNA strands in this solvent.

Last, any potential genetic polymer in concentrated sulfuric acid needs to resist any runaway unspecific reactivity with other dissolved compounds and be stable across different concentrations of acid present in the planetary environment, a subject for future investigation.

This work is a part of a larger effort to explore the stability and reactivity of organic chemicals in concentrated sulfuric acid. We ultimately aim to find suitable structural and functional candidate analogs of terrestrial genetic polymers, proteins, and membranes that are stable in this chemically aggressive solvent. Finding these analogs strengthens the viability of concentrated sulfuric acid as a potential solvent for a biochemistry that is not dependent on liquid water. Our results on the stability and reactivity of PNA hexamers establish that concentrated sulfuric acid can support complex organic chemistry that is fundamentally structurally and functionally different from the highly cross-linked, aromatic oxidized molecules that are the end-product of organic contamination chemistry in sulfuric acid, such as the compounds identified under the umbrella term “red oil” (*8*, *9*, *42*).

We advocate the notion that liquid concentrated sulfuric acid, either in the liquid droplets of the clouds of Venus or on exoplanets, can sustain a diverse range of organic chemistry that might be able to support a form of life different from Earth’s. We continue to challenge the conventional planetary science view that only simple organic chemistry with limited functionality could be stable in this solvent. The characteristics of concentrated sulfuric acid vary notably from those of its aqueous diluted forms, challenging common beliefs in organic chemistry.

We are at the beginning of new developments in organic chemistry for Astrobiology. As a community, we should focus on researching organic chemistry in solvents other than water, which is essential for understanding the extent of the habitability of the Galaxy.

## MATERIALS AND METHODS

### General synthesis of PNA hexamers A6, G6, C6, and T6

The general synthesis of **A6**, **G6**, **C6**, and **T6** is based on a known literature procedure (*43*). We summarize the synthetic procedure below with the following steps. (i) Weigh out of Rink amide ChemMatrix resin (loading, 0.63 mmol/g) in a reaction vessel. (ii) Swell the resin in *N*,*N*′-dimethylformamide (DMF) (2× for 30 min) and then drain. (iii) The resin is treated with 20% piperidine in DMF (3× for 5 min) at RT to remove the 9-fluorenyl methoxycarbonyl (Fmoc)–protecting group on the Rink amide linker. Following deprotection, the resin is washed with DMF (3× for 1 min). (iv) To load the first PNA monomer, dissolve the preweighted PyOXime [1 equivalent (Eq.)] and preweighted monomer (1 Eq.) in DMF. Next, add DIPEA (1 Eq.), after which the mixture is left for activation for 10 min before being added to the Fmoc-deprotected resin. Loading is continued for 2 hours, followed by draining. (v) Upon loading of the C-terminal residue, the resin is washed with DMF (3× for 1 min) and NMP (1× for 1 min), and then unreacted linker sites are capped with a mixture of NMP/2,6-lutidine/Ac_2_O (89/6/5; 3× for 5 min). The resin is then washed with DMF (3× for 1 min). (vi) Fmoc deprotection is performed by treatment with 20% piperidine in DMF (3× for 5 min), followed by washing with DMF (3× for 1 min). (vii) To couple the PNA monomers, dissolve the preweighted PyOxime (2 Eq.) and preweighted monomer (2 Eq.) in DMF. Add DIPEA (2 Eq.), and the mixture is left for activation for 10 min before being added to the Fmoc-deprotected resin. Coupling is continued for 2 hours, followed by washing with DMF (3× for 1 min). (viii) Repeat steps vi and vii until completion of the desired sequence. (ix) At the end of the synthesis, Fmoc deprotection is performed as described in step vi, followed by washing with DMF (4× for 2 min) and with dichloromethane (4× for 2 min). (x) The washed resin is treated with trifluoroacetic acid (TFA)/triethyl silane/H_2_O (95/2.5/2.5; 2× for 30 min + 1× for 1 min), and the combined cleavage mixtures are concentrated in vacuo. The residue is dissolved in a minimal amount of MeOH and added to a large volume of Et_2_O, and the resulting white suspension is filtered, washed with Et_2_O, and dried in vacuo. The residue is then further purified by preparative reversed-phase chromatography and lyophilized.

### PNA adenine hexamer synthesis (A6)

The PNA hexamer **A6** was synthesized according to the general procedure of the synthesis of PNA hexamers (fig. S1). The first adenine was loaded on the resin (0.63 mmol/g, 545 mg) with a mixture of Fmoc PNA-A(Bhoc)-OH (120 mg, 1 Eq., 165 μmol), PyOXime (85 mg, 1 Eq., 166 μmol), and DIPEA (29 μl, 1 Eq., 165 μmol) in DMF (4 ml). Coupling of the following bases was done with a mixture of Fmoc PNA-A(Bhoc)-OH (240 mg, 2 Eq., 331 μmol), PyOXime (169 mg, 2 Eq., 331 μmol), and DIPEA (58 μl, 2 Eq., 331 μmol) in DMF (4 ml). The product obtained after filtration (227 mg) was purified further by preparative reversed-phase chromatography to obtain adenine hexamer **A6** (150 mg, 54% yield based on the free base, and purity of 97.4%) as the TFA salt. Compound **A6** was dissolved in 98 wt % D_2_SO_4_ and DMSO-*d*_6_ (9:1, v/v), and NMR spectra were measured: ^1^H NMR (400 MHz, D_2_SO_4_): δ 8.88 to 8.62 (m, 6*H*), 8.26 to 8.05 (m, 6*H*), 5.34 to 4.78 (m, 12*H*), 4.44 to 3.73 (m, 12*H*), and 3.65 to 2.81 (m, 24*H*).

### PNA guanine hexamer synthesis (G6)

The PNA hexamer **G6** was synthesized according to the general procedure of the synthesis of PNA hexamers (fig. S1). The first guanine was loaded on the resin (0.63 mmol/g, 395 mg) with a mixture of Fmoc PNA-G(Bhoc)-OH (120 mg, 1 Eq., 162 μmol), PyOXime (83 mg, 1 Eq., 162 μmol), and DIPEA (28 μl, 1 Eq., 162 μmol) in DMF (4 ml). Coupling of the following bases was done with a mixture of Fmoc PNA-G(Bhoc)-OH (240 mg, 2 Eq., 324 μmol), PyOXime (166 mg, 2 Eq., 324 μmol), and DIPEA (56 μl, 2 Eq., 324 μmol) in DMF (4 ml). The product obtained after filtration (124 mg) was purified further by preparative reversed-phase chromatography to obtain guanine hexamer **G6** (72 mg, 25% yield based on the free base, and purity of 95.9%) as the TFA salt. Compound **G6** was dissolved in 98 wt % D_2_SO_4_ and DMSO-*d*_6_ (9:1, v/v), and NMR spectra were measured: ^1^H NMR (400 MHz, D_2_SO_4_): δ 8.27 (s, 6*H*), 5.32 to 4.47 (m, 12*H*), 4.40 to 3.70 (m, 12*H*), and 3.64 to 2.78 (m, 24*H*).

### PNA cytosine hexamer synthesis (C6)

The PNA hexamer **C6** was synthesized according to the general procedure of the synthesis of PNA hexamers (fig. S1). The first cytosine was loaded on the resin (0.63 mmol/g, 350 mg) with a mixture of Fmoc PNA-C(Bhoc)-OH (100 mg, 1 Eq., 143 μmol), PyOXime (73 mg, 1 Eq., 143 μmol), and DIPEA (25 μl, 1 Eq., 143 μmol) in DMF (4 ml). Coupling of the following bases was done with a mixture of Fmoc PNA-C(Bhoc)-OH (200 mg, 2 Eq., 285 μmol), PyOXime (146 mg, 2 Eq., 285 μmol), and DIPEA (50 μl, 2 Eq., 285 μmol) in DMF (4 ml). The product obtained after filtration (215 mg) was purified further by preparative reversed-phase chromatography to obtain cytosine hexamer **C6** (156 mg, 72% yield based on the free base, and purity of 98.4%) as the TFA salt. Compound **C6** was dissolved in 98 wt % D_2_SO_4_ and DMSO-*d*_6_ (9:1, v/v), and NMR spectra were measured: ^1^H NMR (400 MHz, D_2_SO_4_): δ 7.21 to 6.98 (m, 6*H*), 5.88 to 5.64 (m, 6*H*), 4.65 to 3.75 (m, 24*H*), and 3.63 to 2.74 (m, 24*H*).

### PNA thymine hexamer synthesis (T6)

The PNA hexamer **T6** was synthesized according to the general procedure of the synthesis of PNA hexamers (fig. S1). The first cytosine was loaded on the resin (0.63 mmol/g, 350 mg) with a mixture of Fmoc PNA-T-OH (100 mg, 1 Eq., 197 μmol), PyOXime (101 mg, 1 Eq., 197 μmol), and DIPEA (34 μl, 1 Eq., 197 μmol) in DMF (4 ml). Coupling of the following bases was done with a mixture of Fmoc PNA-T-OH (200 mg, 2 Eq., 395 μmol), PyOXime (202 mg, 2 Eq., 395 μmol), and DIPEA (69 μl, 2 Eq., 395 μmol) in DMF (4 ml). The product obtained after filtration (257 mg) was purified further by preparative reversed-phase chromatography to obtain thymine hexamer **T6** (103 mg, 32% yield based on the free base, and purity of 82.4%) as the TFA salt. Compound **T6** was dissolved in 98 wt % D_2_SO_4_ and DMSO-*d*_6_ (9:1, v/v), and NMR spectra were measured: ^1^H NMR (400 MHz, D_2_SO_4_): δ 7.44 (s, 6*H*), 4.74 to 4.27 (m, 12*H*), 4.27 to 3.81 (m, 12*H*), 3.64 to 2.74 (m, 24*H*), and 1.58 (s, 18*H*).

### General synthesis of PNA monomers mA, mG, mC, and mT

The general synthesis of **mA**, **mG**, **mC**, and **mT** is based on a known literature procedure (*43*). We summarize the synthetic procedure below with the following steps. (i) Weigh out of Rink amide ChemMatrix resin (loading, 0.63 mmol/g) in a reaction vessel. (ii) Swell the resin in DMF (2× for 30 min) and then drain. (iii) The resin is treated with 20% piperidine in DMF (3× for 5 min) at RT to remove the Fmoc-protecting group on the Rink amide linker. Following deprotection, the resin is washed with DMF (3× for 1 min). (iv) To load the PNA monomer, dissolve the preweighted PyOXime (1 Eq.) and preweighted monomer (1 Eq.) in DMF. Next, add DIPEA (1 Eq.), after which the mixture is left for activation for 10 min before being added to the Fmoc-deprotected resin. Loading is continued for 2 hours, followed by draining. (v) Upon loading, the resin is washed with DMF (3× for 1 min) and NMP (1× for 1 min), and then unreacted linker sites are capped with a mixture of NMP/2,6-lutidine/Ac_2_O (89/6/5; 3× for 5 min). The resin is then washed with DMF (3× for 1 min). (vi) Fmoc deprotection is performed by treatment with 20% piperidine in DMF (3× for 5 min), followed by washing with DMF (4× for 2 min) and with dichloromethane (4× for 2 min). (vii) The washed resin is treated with TFA/triethyl silane/H_2_O (95/2.5/2.5; 2× for 30 min + 1× for 1 min), and the combined cleavage mixtures are concentrated in vacuo. The residue is dissolved in a minimal amount of MeOH and added to a large volume of Et_2_O, and the resulting white suspension is filtered, washed with Et_2_O, and dried in vacuo. The residue is then lyophilized to obtain the monomers as the TFA salt.

### PNA adenine monomer synthesis (mA)

PNA monomer **mA** was synthesized according to the general synthesis of PNA monomers (fig. S2). The protected adenine monomer was loaded on the resin (0.63 mmol/g, 650 mg) with a mixture of Fmoc PNA-A(Bhoc)-OH (250 mg, 1 Eq., 344 μmol), PyOXime (176 mg, 1 Eq., 344 μmol), and DIPEA (60 μl, 1 Eq., 344 μmol) in DMF (4 ml). Adenine monomer **mA** (67 mg, 48% yield based on the mono-TFA salt, and purity of 72%) was obtained as the TFA salt as a white solid after lyophilization. Compound **mA** was dissolved in 98 wt % D_2_SO_4_ and DMSO-*d*_6_ (9:1, v/v), and NMR spectra were measured: ^1^H NMR (400 MHz, D_2_SO_4_): δ 8.74 (d, *J* = 3.3 Hz, 1*H*), 8.16 (d, *J* = 14.4 Hz, 1*H*), 5.90 to 5.61 (m, 2*H*), 5.21 to 4.96 (m, 2*H*), 4.24 to 3.91 (m, 2*H*), 3.55 to 3.17 (m, 2*H*), and 3.06 to 2.76 (m, 2*H*).

### PNA guanine monomer synthesis (mG)

PNA monomer **mG** was synthesized according to the general synthesis of PNA monomers (fig. S2). The protected guanine monomer was loaded on the resin (0.63 mmol/g, 650 mg) with a mixture of Fmoc PNA-G(Bhoc)-OH (250 mg, 1 Eq., 337 μmol), PyOXime (172 mg, 1 Eq., 337 μmol), and DIPEA (59 μl, 1 Eq., 337 μmol) in DMF (4 ml). Guanine monomer **mG** (52 mg, 37% yield based on the mono-TFA salt, and purity of 78%) was obtained as the TFA salt as a white solid after lyophilization. Compound **mG** was dissolved in 98 wt % D_2_SO_4_ and DMSO-*d*_6_ (9:1, v/v), and NMR spectra were measured: ^1^H NMR (400 MHz, D_2_SO_4_): δ 8.41 to 8.19 (m, 1*H*), 5.90 to 5.61 (m, 2*H*), 5.04 to 4.75 (m, 2*H*), 4.22 to 3.92 (m, 2*H*), 3.50 to 3.11 (m, 2*H*), and 3.04 to 2.74 (m, 2*H*).

### PNA cytosine monomer synthesis (mC)

PNA monomer **mC** was synthesized according to the general synthesis of PNA monomers (fig. S2). The protected cytosine monomer was loaded on the resin (0.63 mmol/g, 650 mg) with a mixture of Fmoc PNA-C(Bhoc)-OH (250 mg, 1 Eq., 356 μmol), PyOXime (182 mg, 1 Eq., 356 μmol), and DIPEA (62 μl, 1 Eq., 356 μmol) in DMF (4 ml). Cytosine monomer **mC** (96 mg, 70% yield based on the mono-TFA salt, and purity of 78%) was obtained as the TFA salt as a white solid after lyophilization. Compound **mC** was dissolved in 98 wt % D_2_SO_4_ and DMSO-*d*_6_ (9:1, v/v), and NMR spectra were measured: ^1^H NMR (400 MHz, D_2_SO_4_): δ 7.15 to 7.04 (m, 1*H*), 5.85 to 5.60 (m, 3*H*), 4.48 to 4.26 (m, 2*H*), 4.23 to 3.93 (m, 2*H*), 3.46 to 3.24 (m, 2*H*), and 2.98 to 2.78 (m, 2*H*).

### PNA thymine monomer synthesis (mT)

PNA monomer **mT** was synthesized according to the general synthesis of PNA monomers (fig. S2). The protected thymine monomer was loaded on the resin (0.63 mmol/g, 650 mg) with a mixture of Fmoc PNA-T-OH (200 mg, 1 Eq., 395 μmol), PyOXime (202 mg, 1 Eq., 395 μmol), and DIPEA (69 μl, 1 Eq., 395 μmol) in DMF (4 ml). Thymine monomer **mT** (76 mg, 48% yield based on the mono-TFA salt, and purity of 79%) was obtained as the TFA salt as a white solid after lyophilization. Compound **mT** was dissolved in 98 wt % D_2_SO_4_ and DMSO-*d*_6_ (9:1, v/v), and NMR spectra were measured: ^1^H NMR (400 MHz, D_2_SO_4_): δ 7.52 to 7.38 (m, 1*H*), 5.83 to 5.56 (m, 2*H*), 4.57 to 4.36 (m, 2*H*), 4.21 to 3.92 (m, 2*H*), 3.45 to 3.19 (m, 2*H*), 3.01 to 2.77 (m, 2*H*), and 1.58 (s, 3*H*). The solvolysis products (**HA**, **HG**, **HC**, and **HT**) were ordered from Enamine (catalog nos. EN300-71413 for **HA**, EN300-317437 for **HG**, and BBV-38304768 for **HC**) and Ambeed (A152627 for **HT**) and used without further purification.

### Summary of the synthesis of *N*-(2-aminoethyl)glycinamide

Synthesis of *N*-(2-aminoethyl)glycinamide was successfully performed by reacting bromoacetamide in ethylene diamine neat (fig. S3). Around 100 mg of *N*-(2-aminoethyl)glycinamide is available. The compound was obtained in 13.2% yield and a purity of 95% (based on ^1^H NMR measurements).

### Stability testing of PNA hexamers in H_2_SO_4_ by LC-MS and ^1^H NMR analysis

PNA hexamers (**A6**, **G6**, **C6**, and **T6**) (approximately 10 mg) were dissolved in H_2_SO_4_ (98 wt %; concentration, 10 mg/ml) and kept at RT. After three time points (*t*1 = 1 hour, *t*2 = 24 hours, and *t*3 = 14 days), an aliquot (0.10 ml) was diluted with aqueous ammonium acetate (NH_4_OAc) (3 M, 0.90 ml), resulting in solutions with a pH of 3. These samples were then analyzed by LC-MS. To acquire LC-MS data, we used an Agilent 1260 series with ultraviolet detector, ELSD 1260 detector, and Agilent 6120 mass detector at appropriate temperatures (25°, 50°, and 80°C).

We prepared our NMR samples by dissolving 10 mg of the **A6**, **G6**, **C6**, and **T6** hexamers and 10 mg of **mA**, **mG**, **mC**, and **mT** monomers into 1 ml of solvent D_2_SO_4_ in D_2_O in glass vials. We added DMSO-*d*_6_, used as a chemical shift reference compound, to a final concentration of 10% by volume.

To acquire NMR data, we used a Bruker AvanceNeo 400 MHz spectrometer at the appropriate temperature (25°, 50°, 80°, or 100°C). In all cases, we locked on DMSO-*d*_6_ for consistency.

We used MNova software (Mestrelab Research) to process and analyze the NMR data (*44*). The original data for all NMR and LC-MS experiments are available for download as supplementary datasets from Zenodo at https://zenodo.org/records/14632709.
