# Supplementary material for: Astrobiological implications of the stability and reactivity of peptide nucleic acid (PNA) in concentrated sulfuric acid
Source: Sci Adv. 2025 Mar 26;11(13):eadr0006. doi: 10.1126/sciadv.adr0006 (PMC11939054; doi:10.1126/sciadv.adr0006)
Supplement: Supplementary file 1 — Figs. S1 to S3 Legends for data S1 and S2 [file sciadv.adr0006_sm.pdf]

Science Advances
AAAS

Supplementary Materials for

Astrobiological implications of the stability and reactivity of peptide nucleic acid (PNA) in concentrated sulfuric acid

Janusz J. Petkowski *et al.*

Corresponding author: Janusz J. Petkowski, janusz.petkowski@pwr.edu.pl

Sci. Adv. **11**, eadr0006 (2025)
DOI: 10.1126/sciadv.adr0006

The PDF file includes:

Figs. S1 to S3
Legends for data S1 and S2

Other Supplementary Material for this manuscript includes the following:

Data S1 and S2

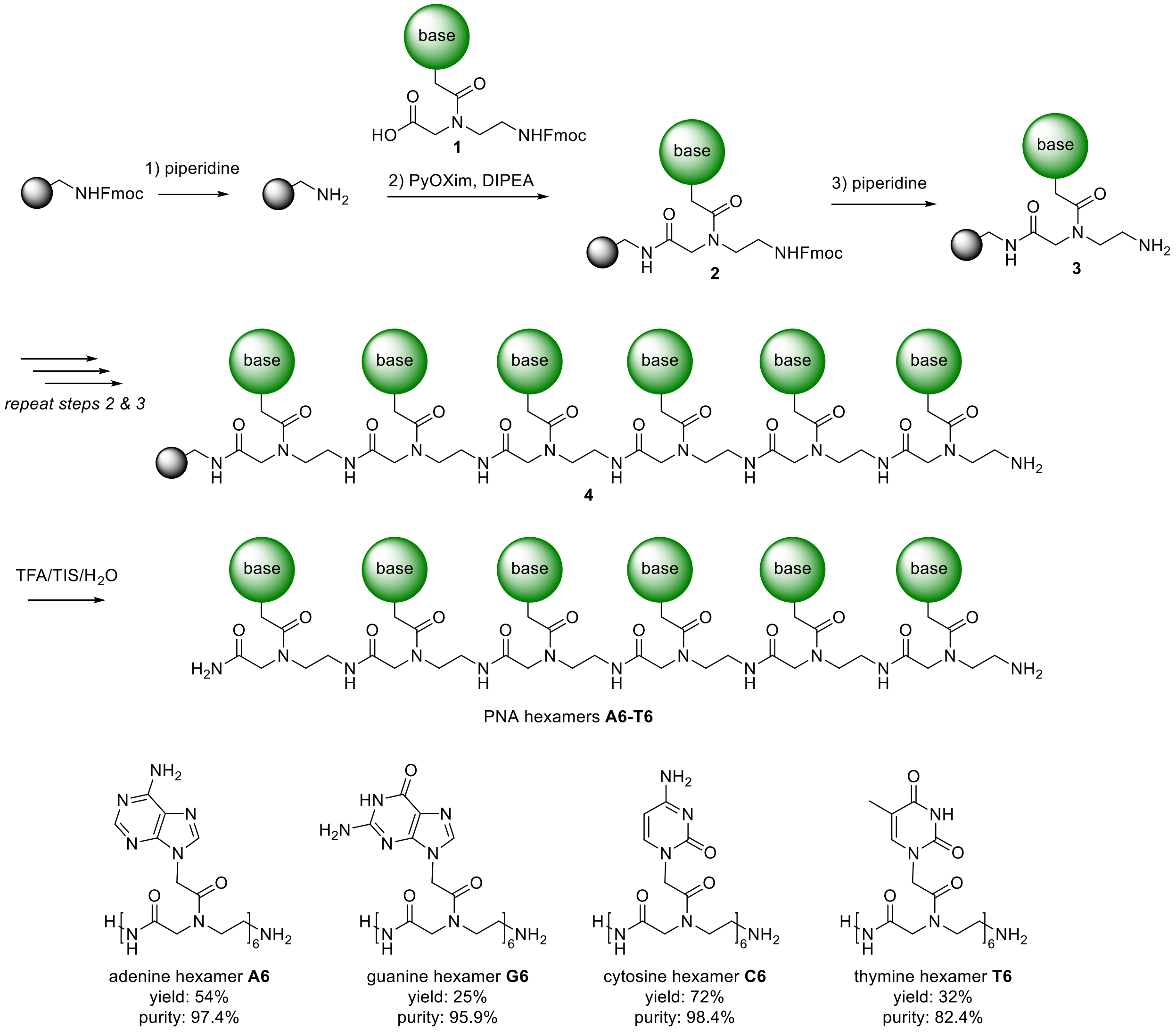

Fig. S1. Synthesis of PNA hexamers A6-T6. 50-150 mg of the PNA hexamers **A6-T6** have been prepared using solid-phase chemistry, with a 25-72% yield and a purity of >82%. In brief, the first chemically protected PNA monomer in the hexamer has been attached to a solid support (Step 2) before removal of the Fmoc protecting group, yielding an intermediate compound (Step 3). Steps 2 and 3 are repeated to eventually yield the desired hexamer attached to a solid support resin. In a final step of the synthesis PNA hexamers are liberated by treating the resin with trifluoracetic acid (TFA).

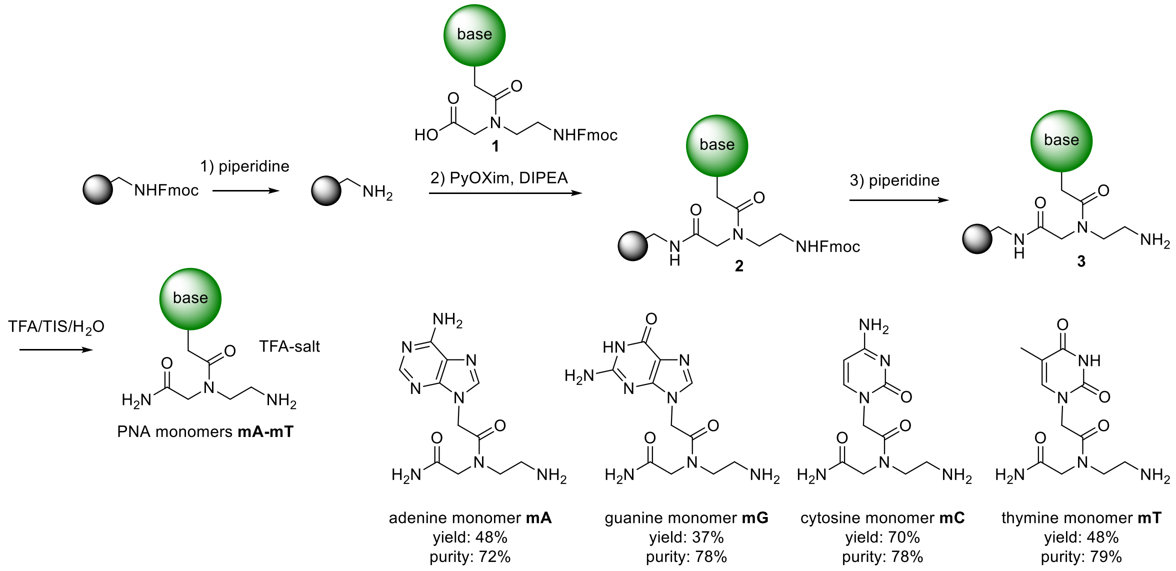

Fig. S2. Synthesis of PNA monomers mA-mT. The respective PNA monomers **mA-mT** were synthesized with the same strategy as the hexamers **A6-T6**, except that the intermediate compound (Step 3) was directly removed from the resin with TFA. All monomers were obtained in 37-70% yield and a purity of 72-79%.

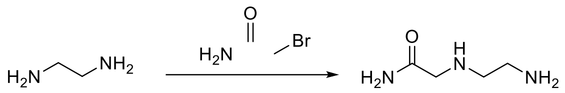

Fig. S3. Preparation of N-(2-aminoethyl) glycinamide. *N*-(2-aminoethyl) glycinamide has been prepared by direct alkylation of ethylene diamine. The compound was obtained in 13.2% yield and a purity of 95%.

Data S1. LCMS data for PNA hexamers.

The folder Supplementary Dataset 1-LCMS DATA contains pdfs of all LCMS spectra. The original LCMS data include the LCMS analysis of PNA hexamers A-T at various time points, at room temperature (RT) (1h, 24h, 14 days; t = RT) as well as at high temperature (50°C and 80°C). The original data for all LCMS experiments are available for download as Supplementary Datasets from Zenodo at https://zenodo.org/records/14632709.

Data S2. ^{1}H NMR spectra for PNA hexamers and monomers.

The folder Supplementary Dataset 2-NMR DATA contains the original NMR data. The original data for all NMR experiments are available for download as Supplementary Datasets from Zenodo at https://zenodo.org/records/14632709.
