# Supplementary material for: Astrobiological implications of the stability and reactivity of peptide nucleic acid (PNA) in concentrated sulfuric acid
Source: Sci Adv. 2025 Mar 26;11(13):eadr0006. doi: 10.1126/sciadv.adr0006 (PMC11939054; doi:10.1126/sciadv.adr0006)
Supplement: Supplementary file 2 — Data S1 and S2 [file sciadv.adr0006_data_s1_and_s2.zip › Supplementary Dataset 1-LCMS DATA/LCMS PNA Hexamers A-T/LCMS A6 50C_80C/50C/1h/CPT22010446-13-A1-50deg-1h.pdf]

Sample-> CPT22010446-13-A1-50deg-1h

Injection Date : Tue, 17. Oct. 2023

Seq Line : 19
Location : 28
Inj. Vol. : 2 µl

Acq. Method : C:\Users\Public\Documents\ChemStation\1\Data\SE17OCT 2023-10-17 07-53-29\22010446 LCMS-6.M
Analysis Method : C:\Users\Public\Documents\ChemStation\1\Data\SE17OCT 2023-10-17 07-53-29\22010446 LCMS-6.M (Sequence Method)
Waters XBridge Phenyl (4.6 * 150 mm; 3.5 µm); 0.05% TFA (aq) / AcN: 100/0 (0.0 min) --> (6.0 min) --> 70/30 (0.0 min) --> (2.0 min) --> 10/90 (2.0 min); Flow: 1.0 ml/min;
MSD1 = positive; MSD2 = negative

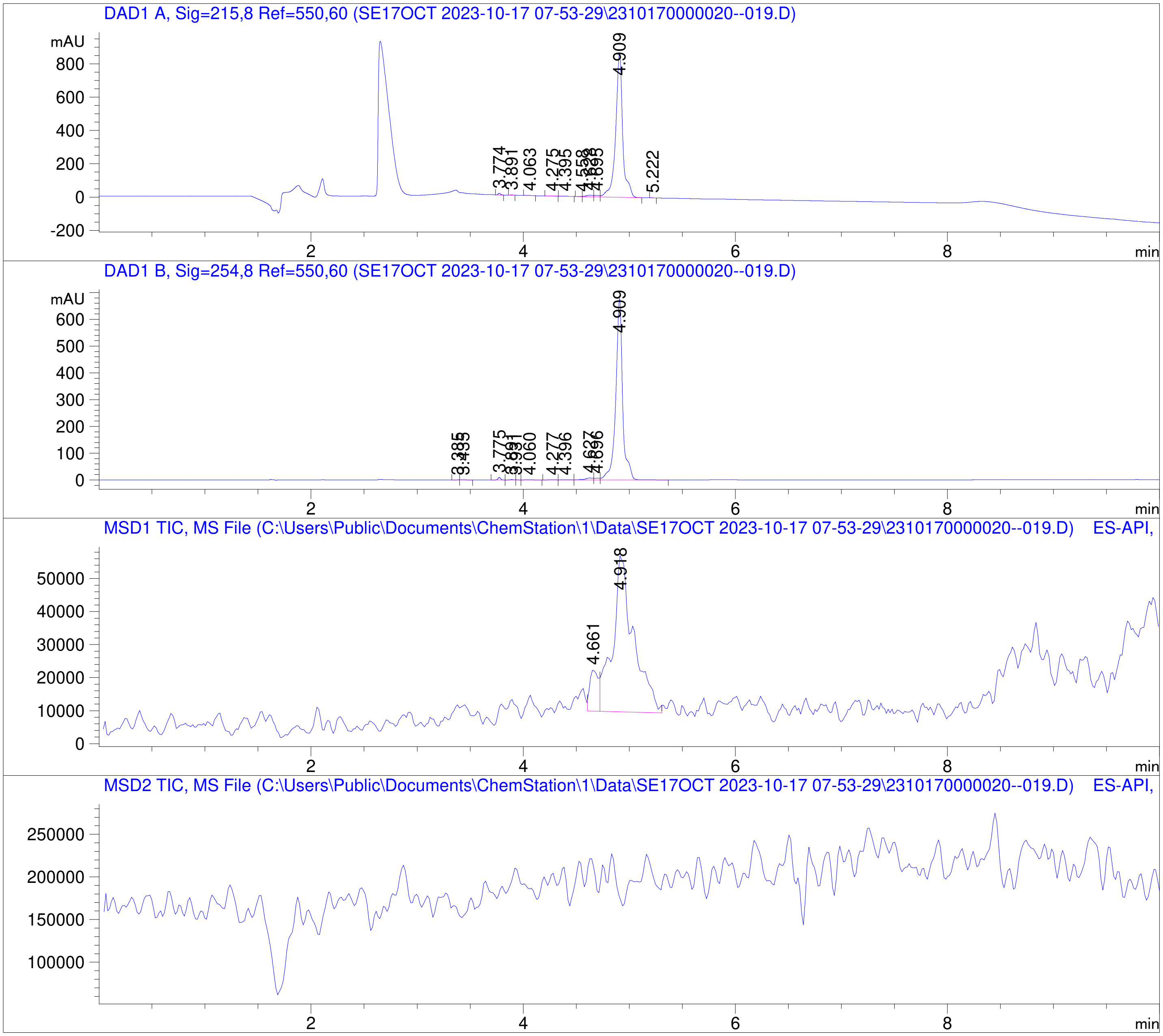

DAD1 A, Sig=215,8 Ref=550,60

Peak #	Ret. Time [min]	Area [mV *s]	Area %
1	3.774	15.645	0.379
2	3.891	2.347	0.057
3	4.063	2.730	0.066
4	4.275	2.643	0.064
5	4.395	4.181	0.101
6	4.558	4.702	0.114
7	4.628	42.389	1.027
8	4.695	31.135	0.754
9	4.909	4020.296	97.420
10	5.222	0.691	0.017

DAD1 B, Sig=254,8 Ref=550,60

Peak #	Ret. Time [min]	Area [mV *s]	Area %
1	3.385	0.729	0.023
2	3.435	2.479	0.078
3	3.775	15.976	0.505
4	3.891	3.441	0.109
5	3.931	0.599	0.019
6	4.060	2.922	0.092
7	4.277	2.821	0.089
8	4.396	4.261	0.135
9	4.627	36.782	1.162
10	4.696	23.933	0.756
11	4.909	3070.850	97.032

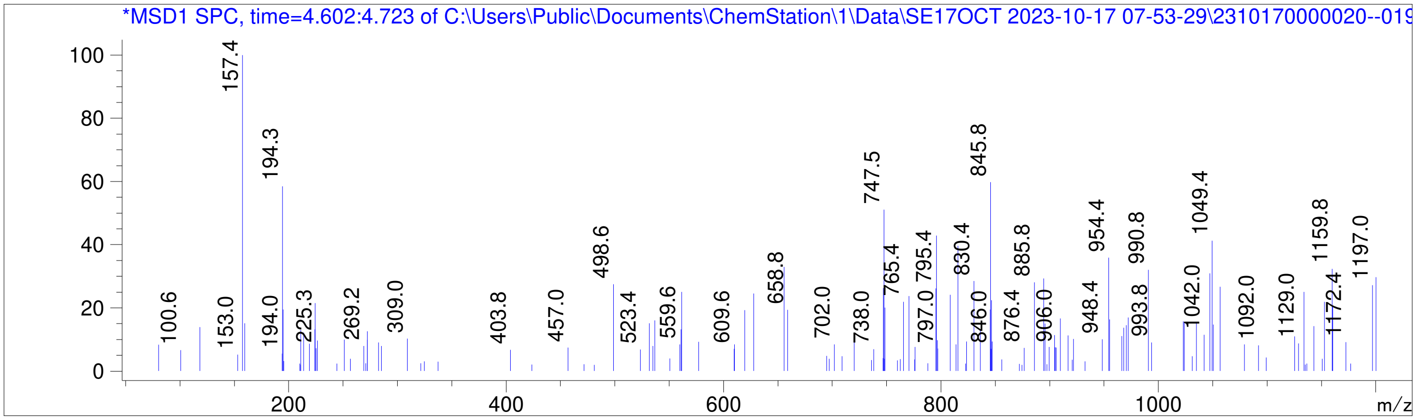
*MSD1 SPC, time=4.602:4.723 of C:\Users\Public\Documents\ChemStation\1\Data\SE17OCT 2023-10-17 07-53-29\2310170000020--019
157.4
194.3
747.5
845.8
498.6
1049.4
954.4
990.8
1159.8
1197.0
m/z

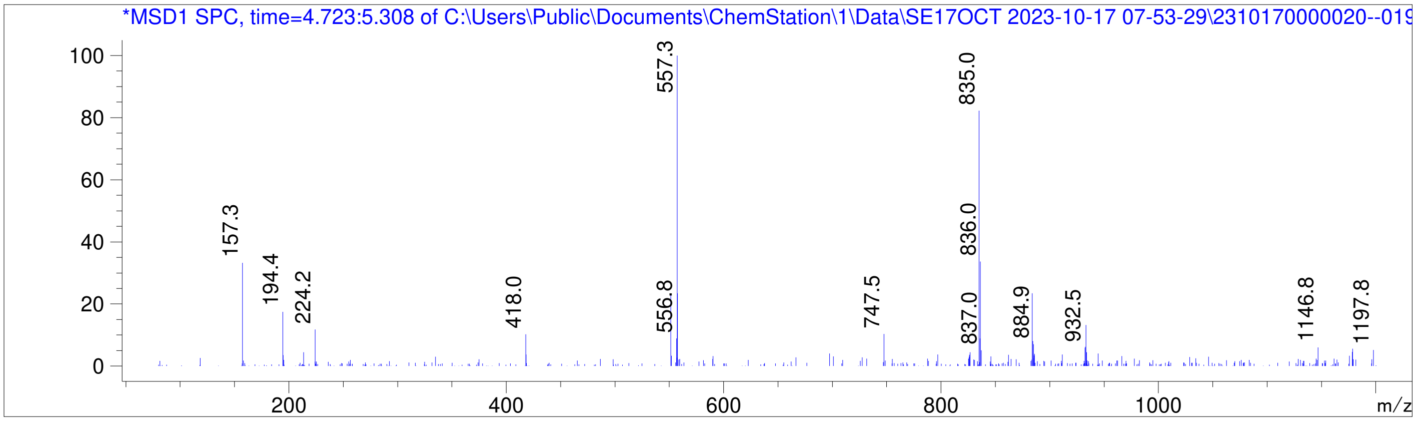
*MSD1 SPC, time=4.723:5.308 of C:\Users\Public\Documents\ChemStation\1\Data\SE17OCT 2023-10-17 07-53-29\2310170000020--019
557.3
835.0
157.3
836.0
194.4
224.2
418.0
556.8
747.5
837.0
884.9
932.5
1146.8
1197.8
m/z
