# Supplementary material for: Astrobiological implications of the stability and reactivity of peptide nucleic acid (PNA) in concentrated sulfuric acid
Source: Sci Adv. 2025 Mar 26;11(13):eadr0006. doi: 10.1126/sciadv.adr0006 (PMC11939054; doi:10.1126/sciadv.adr0006)

Data -> C:\Users\Public\Documents\ChemStation\1\Data\SE01NOV 2023-11-01 15-33-53\ ->
Sample-> CPT22010446-13-A2-50deg-1h

===

Injection Date : Thu, 2. Nov. 2023

Seq Line : 46
Location : 16
Inj. Vol. : 2 µl

Acq. Method : C:\Users\Public\Documents\ChemStation\1\Data\SE01NOV 2023-11-01 15-33-53\22010446 LCMS-6.M
Analysis Method : C:\Users\Public\Documents\ChemStation\1\Data\SE01NOV 2023-11-01 15-33-53\22010446 LCMS-6.M (Sequence Method)
Waters XBridge Phenyl (4.6 * 150 mm; 3.5 µm); 0.05% TFA (aq) / AcN: 100/0 (0.0 min) --> (6.0 min) --> 70/30 (0.0 min) --> (2.0 min) --> 10/90 (2.0 min); Flow: 1.0 ml/min;
MSD1 = positive; MSD2 = negative

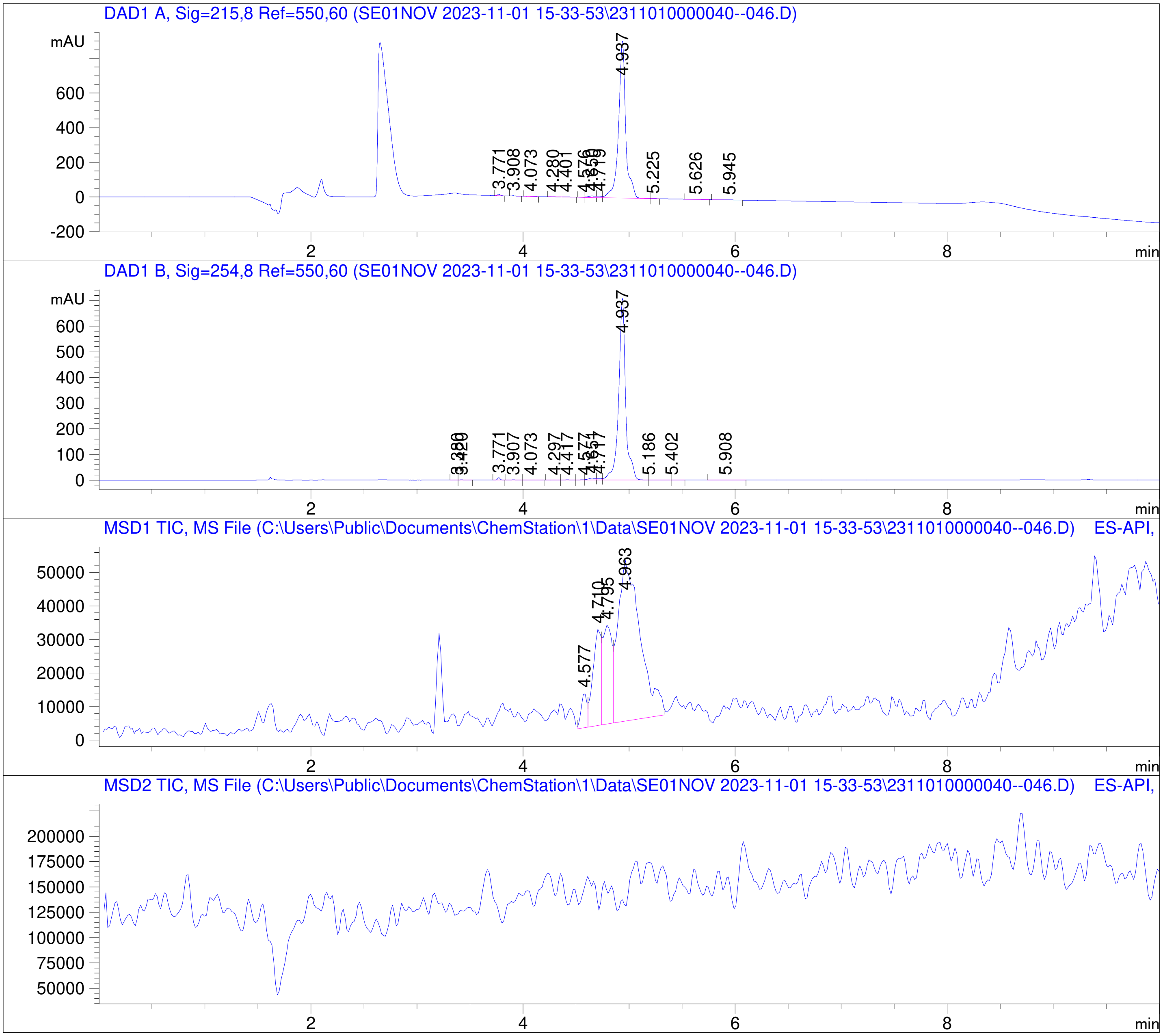

DAD1 A, Sig=215,8 Ref=550,60

Peak #	Ret. Time [min]	Area [mV *s]	Area %
1	3.771	16.163	0.367
2	3.908	3.909	0.089
3	4.073	3.281	0.075
4	4.280	2.800	0.064
5	4.401	4.599	0.104
6	4.576	3.748	0.085
7	4.650	44.380	1.008
8	4.719	29.067	0.660
9	4.937	4282.617	97.266
10	5.225	1.683	0.038
11	5.626	4.185	0.095
12	5.945	6.570	0.149

DAD1 B, Sig=254,8 Ref=550,60

Peak #	Ret. Time [min]	Area [mV *s]	Area %
1	3.380	0.646	0.019
2	3.420	2.813	0.083
3	3.771	15.782	0.468
4	3.907	3.746	0.111
5	4.073	2.941	0.087
6	4.297	3.192	0.095
7	4.417	4.649	0.138
8	4.577	3.974	0.118
9	4.651	34.296	1.016
10	4.717	22.551	0.668
11	4.937	3270.388	96.895
12	5.186	2.376	0.070
13	5.402	0.439	0.013
14	5.908	7.380	0.219

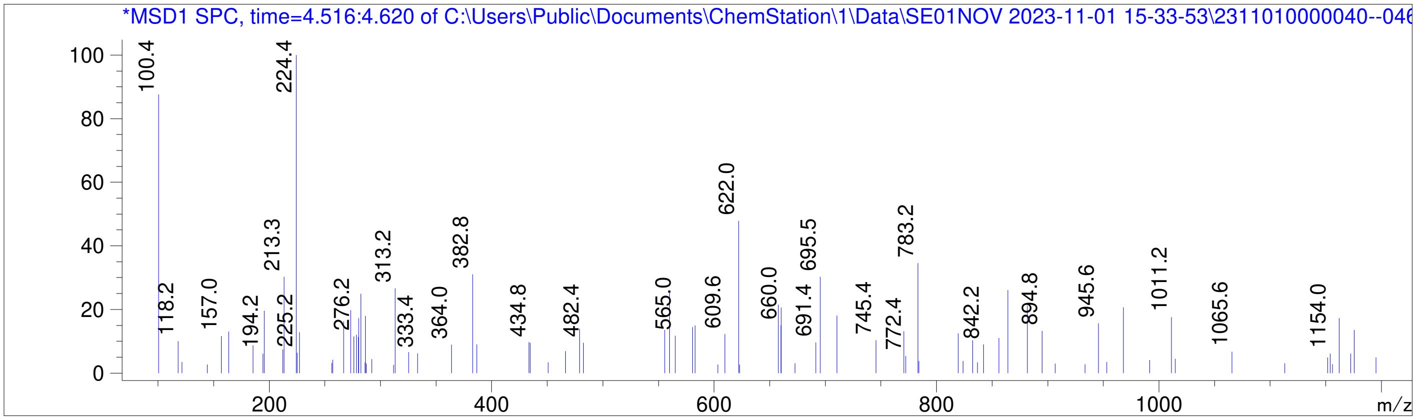
*MSD1 SPC, time=4.516:4.620 of C:\Users\Public\Documents\ChemStation\1\Data\SE01NOV 2023-11-01 15-33-53\2311010000040--046
100.4
224.4
118.2
157.0
194.2
213.3
225.2
276.2
313.2
333.4
364.0
382.8
434.8
482.4
565.0
609.6
622.0
660.0
691.4
695.5
745.4
772.4
783.2
842.2
894.8
945.6
1011.2
1065.6
1154.0
m/z

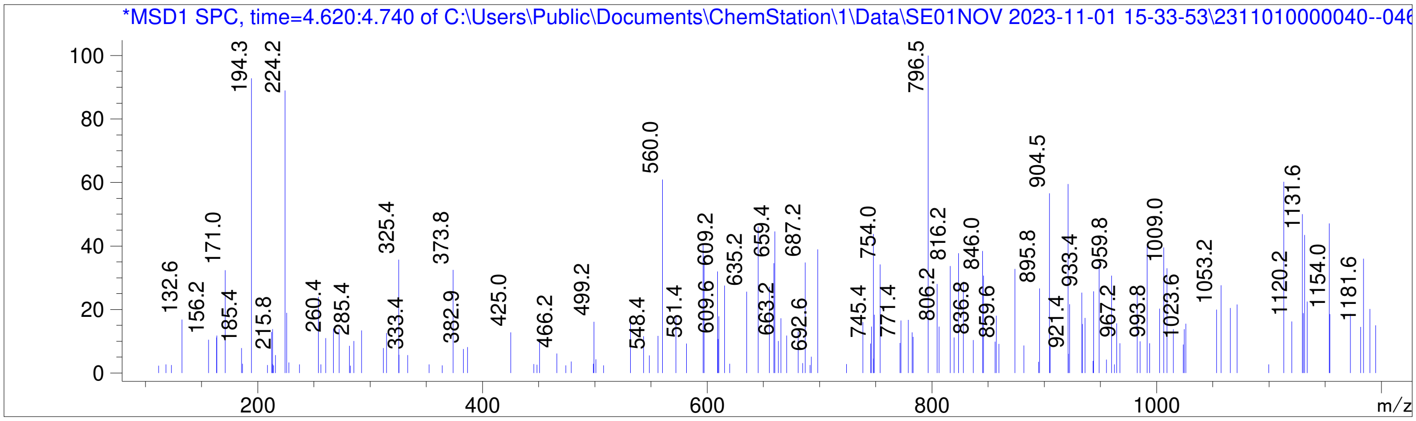
*MSD1 SPC, time=4.620:4.740 of C:\Users\Public\Documents\ChemStation\1\Data\SE01NOV 2023-11-01 15-33-53\2311010000040--046
194.3
224.2
796.5
560.0
904.5
1131.6
132.6
156.2
171.0
185.4
215.8
260.4
285.4
325.4
333.4
373.8
382.9
425.0
466.2
499.2
548.4
581.4
609.6
609.2
635.2
659.4
663.2
687.2
692.6
745.4
754.0
771.4
806.2
816.2
836.8
846.0
859.6
895.8
921.4
933.4
959.8
967.2
993.8
1009.0
1023.6
1053.2
1120.2
1154.0
1181.6
m/z

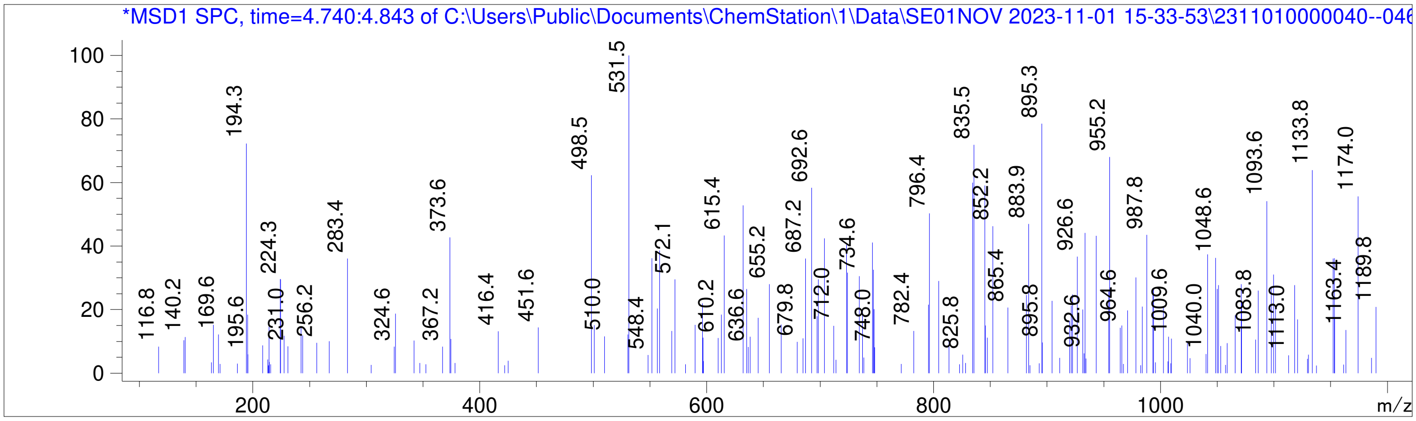
*MSD1 SPC, time=4.740:4.843 of C:\Users\Public\Documents\ChemStation\1\Data\SE01NOV 2023-11-01 15-33-53\2311010000040--046
531.5
194.3
498.5
895.3
835.5
955.2
1133.8
1174.0
1093.6
116.8
140.2
169.6
195.6
224.3
231.0
256.2
283.4
324.6
367.2
373.6
416.4
451.6
510.0
548.4
572.1
610.2
615.4
636.6
655.2
679.8
687.2
692.6
712.0
734.6
748.0
782.4
796.4
825.8
852.2
865.4
883.9
895.8
926.6
932.6
964.6
987.8
1009.6
1040.0
1048.6
1083.8
1113.0
1163.4
1189.8
m/z

Sample-> CPT22010446-13-A2-50deg-1h

==

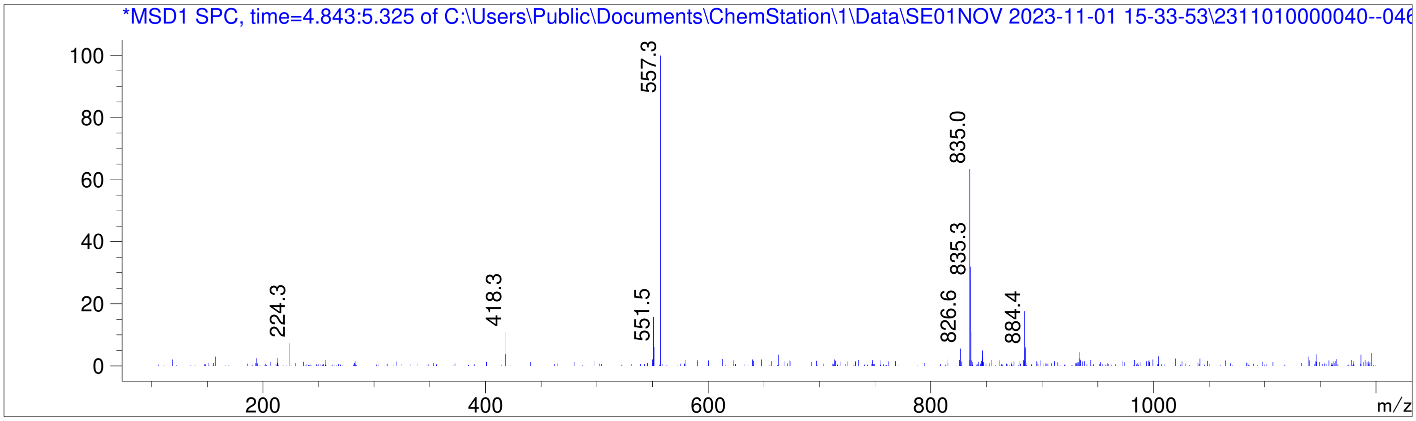

Supplement: Supplementary file 2 — Data S1 and S2 [file sciadv.adr0006_data_s1_and_s2.zip › Supplementary Dataset 1-LCMS DATA/LCMS PNA Hexamers A-T/LCMS A6 50C_80C/50C/1h/CPT22010446-13-A2-50deg-1h.pdf]
