# Supplementary material for: Astrobiological implications of the stability and reactivity of peptide nucleic acid (PNA) in concentrated sulfuric acid
Source: Sci Adv. 2025 Mar 26;11(13):eadr0006. doi: 10.1126/sciadv.adr0006 (PMC11939054; doi:10.1126/sciadv.adr0006)

===

Injection Date : Tue, 17. Oct. 2023

Seq Line : 4
Location : 48
Inj. Vol. : 2 µl

Acq. Method : C:\Users\Public\Documents\ChemStation\1\Data\SE17OCT 2023-10-17 16-51-41\22010446 LCMS-6.M

Analysis Method : C:\Users\Public\Documents\ChemStation\1\Data\SE17OCT 2023-10-17 16-51-41\22010446 LCMS-6.M (Sequence Method)

Waters XBridge Phenyl (4.6 * 150 mm; 3.5 µm); 0.05% TFA (aq) / AcN: 100/0 (0.0 min) --> (6.0 min) --> 70/30 (0.0 min) --> (2.0 min) --> 10/90 (2.0 min); Flow: 1.0 ml/min;
MSD1 = positive; MSD2 = negative

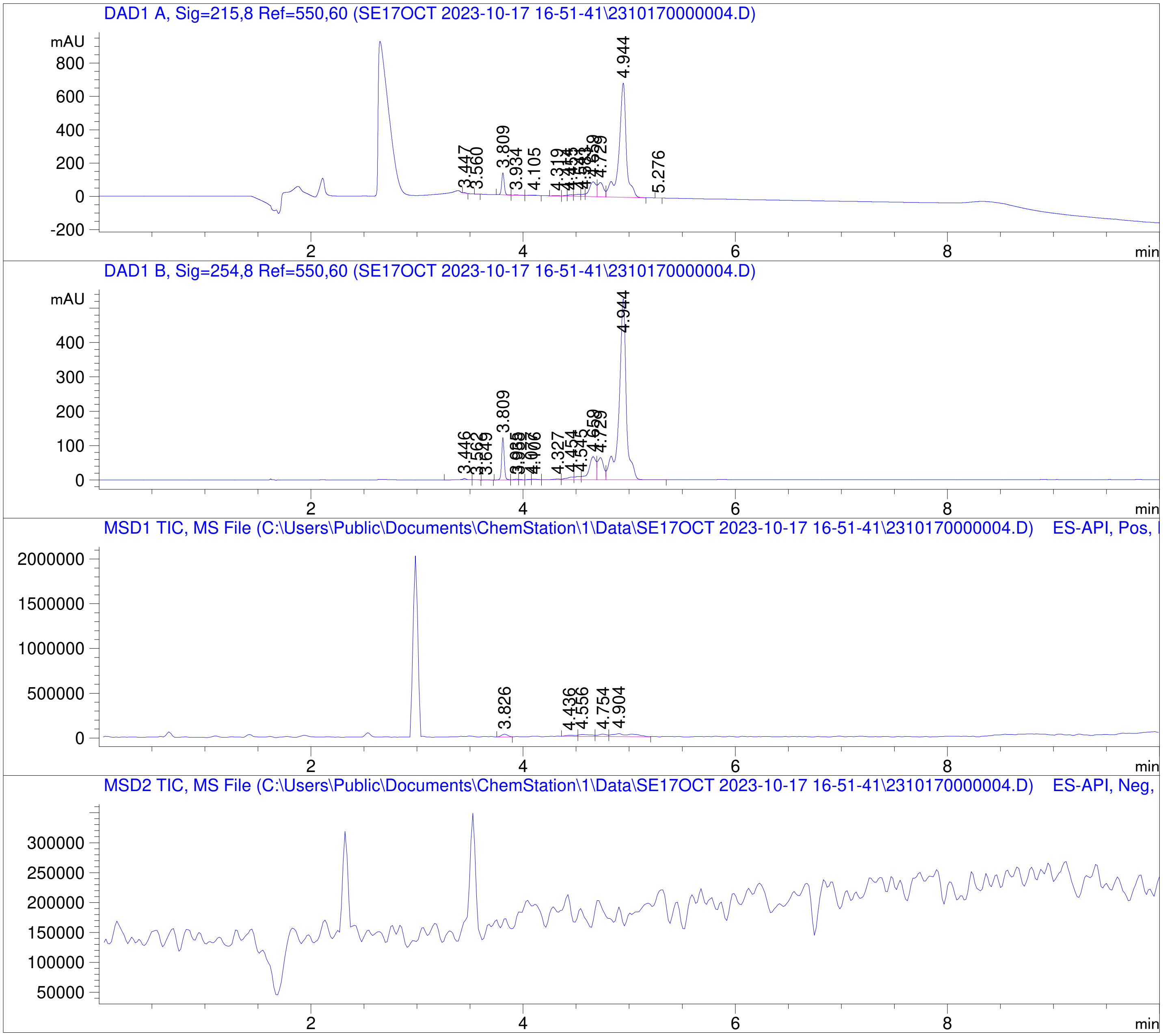

DAD1 A, Sig=215,8 Ref=550,60

Peak #	Ret. Time [min]	Area [mV *s]	Area %
1	3.447	3.523	0.080
2	3.560	0.353	0.008
3	3.809	219.853	4.983
4	3.934	5.421	0.123
5	4.105	9.679	0.219
6	4.319	7.500	0.170
7	4.414	9.194	0.208
8	4.455	27.381	0.621
9	4.541	43.772	0.992
10	4.583	32.011	0.725
11	4.659	391.378	8.870
12	4.729	333.520	7.559
13	4.944	3328.029	75.425
14	5.276	0.753	0.017

DAD1 B, Sig=254,8 Ref=550,60

Peak #	Ret. Time [min]	Area [mV *s]	Area %
1	3.446	10.586	0.310
2	3.562	0.677	0.020
3	3.649	0.510	0.015
4	3.809	205.529	6.010
5	3.935	5.499	0.161
6	3.958	2.864	0.084
7	4.077	3.966	0.116
8	4.106	7.561	0.221
9	4.327	14.050	0.411
10	4.454	37.875	1.108
11	4.545	37.008	1.082
12	4.659	318.689	9.320
13	4.729	253.327	7.408
14	4.944	2521.394	73.735

==

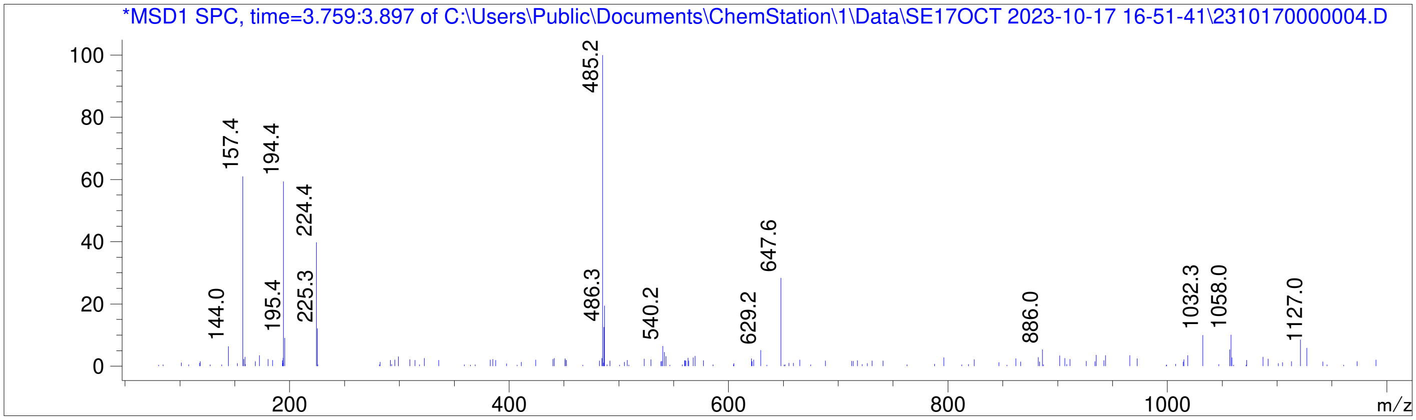
*MSD1 SPC, time=3.759:3.897 of C:\Users\Public\Documents\ChemStation\1\Data\SE17OCT 2023-10-17 16-51-41\2310170000004.D
100
80
60
40
20
0
485.2
157.4
194.4
224.4
647.6
144.0
195.4
225.3
486.3
540.2
629.2
886.0
1032.3
1058.0
1127.0
200
400
600
800
1000
m/z

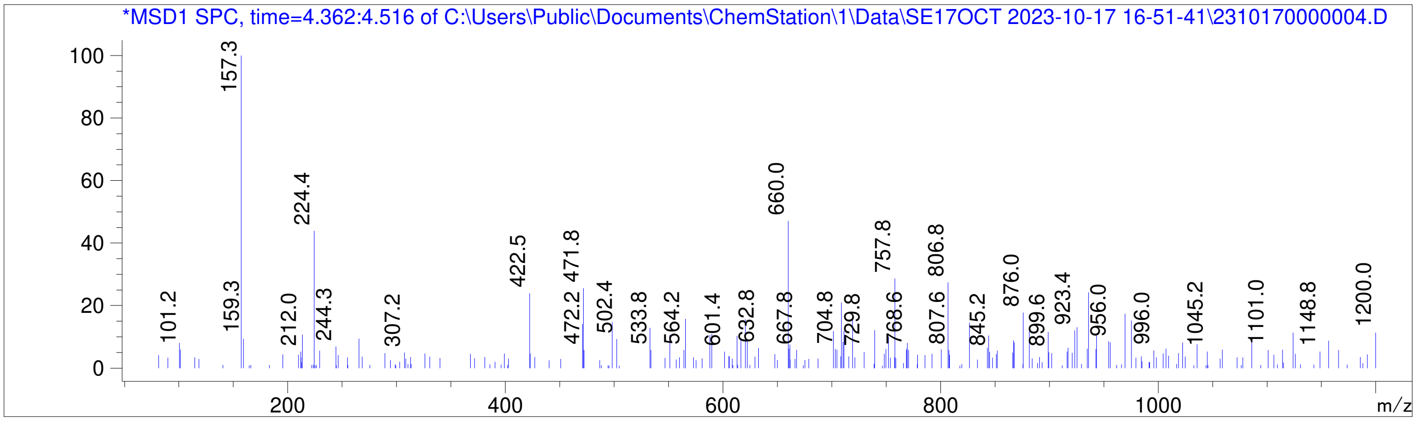
*MSD1 SPC, time=4.362:4.516 of C:\Users\Public\Documents\ChemStation\1\Data\SE17OCT 2023-10-17 16-51-41\2310170000004.D
100
80
60
40
20
0
157.3
224.4
660.0
422.5
471.8
757.8
806.8
101.2
159.3
212.0
244.3
307.2
472.2
502.4
533.8
564.2
601.4
632.8
667.8
704.8
729.8
768.6
807.6
845.2
876.0
899.6
923.4
956.0
996.0
1045.2
1101.0
1148.8
1200.0
200
400
600
800
1000
m/z

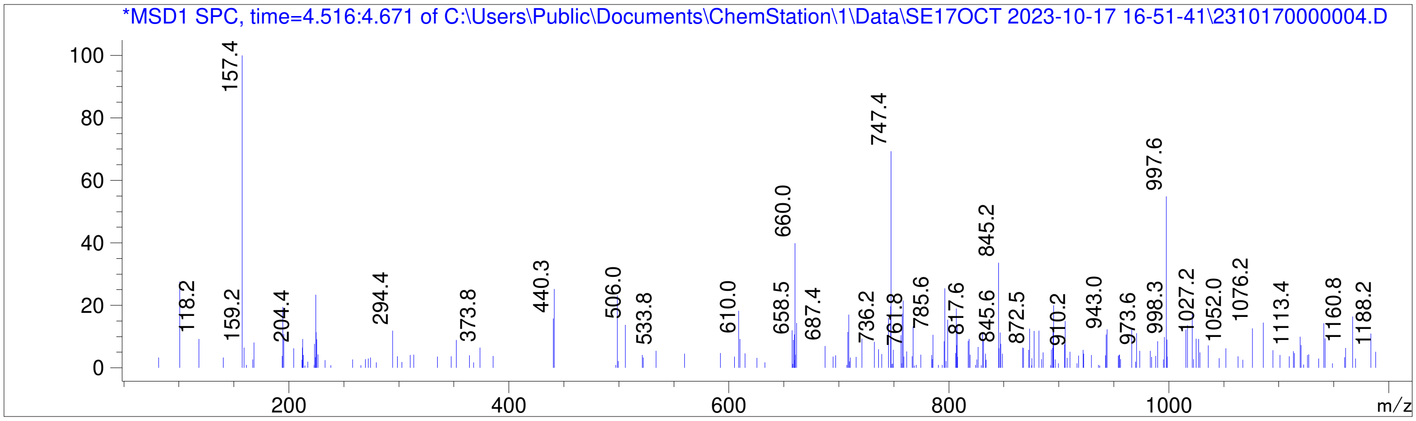
*MSD1 SPC, time=4.516:4.671 of C:\Users\Public\Documents\ChemStation\1\Data\SE17OCT 2023-10-17 16-51-41\2310170000004.D
100
80
60
40
20
0
157.4
747.4
997.6
660.0
845.2
118.2
159.2
204.4
294.4
373.8
440.3
506.0
533.8
610.0
658.5
687.4
736.2
761.8
785.6
817.6
845.6
872.5
910.2
943.0
973.6
998.3
1027.2
1052.0
1076.2
1113.4
1160.8
1188.2
200
400
600
800
1000
m/z

Sample-> CPT22010446-13-A1-50deg-1h

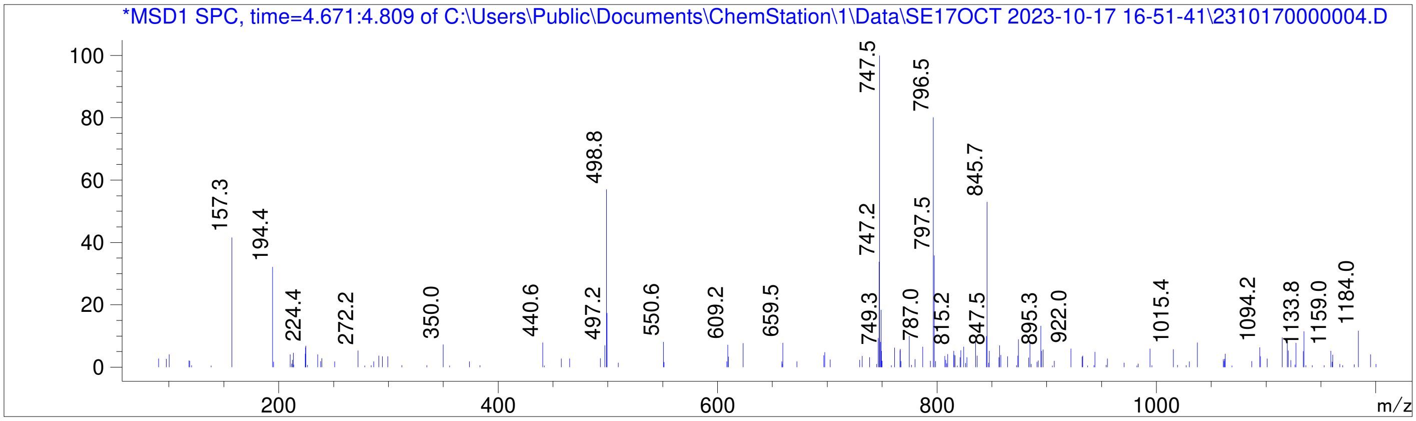

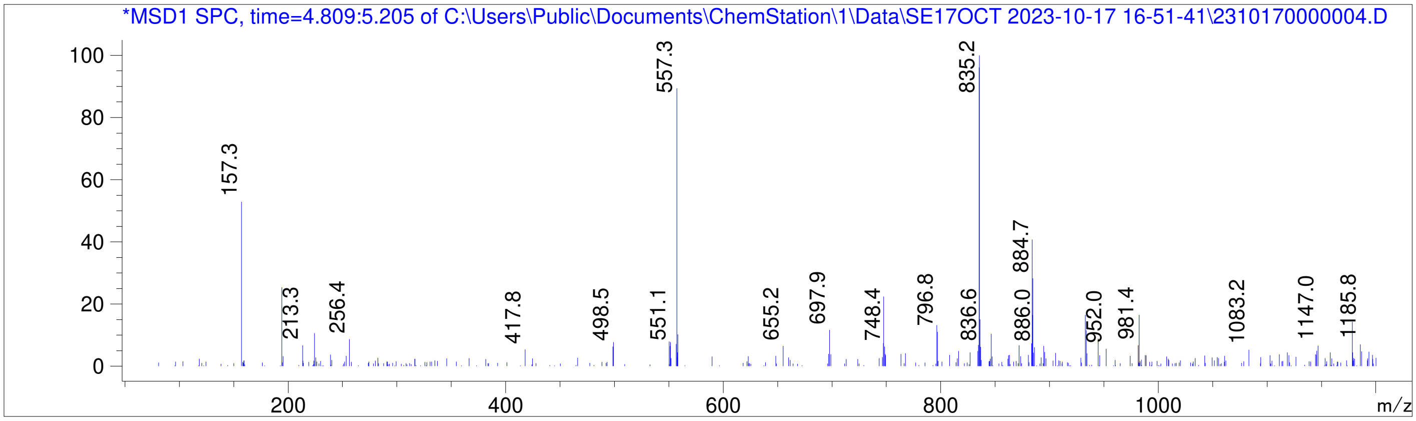

Supplement: Supplementary file 2 — Data S1 and S2 [file sciadv.adr0006_data_s1_and_s2.zip › Supplementary Dataset 1-LCMS DATA/LCMS PNA Hexamers A-T/LCMS A6 50C_80C/50C/24h/CPT22010446-13-A1-50deg-24h.pdf]
