# Supplementary material for: Astrobiological implications of the stability and reactivity of peptide nucleic acid (PNA) in concentrated sulfuric acid
Source: Sci Adv. 2025 Mar 26;11(13):eadr0006. doi: 10.1126/sciadv.adr0006 (PMC11939054; doi:10.1126/sciadv.adr0006)
Supplement: Supplementary file 2 — Data S1 and S2 [file sciadv.adr0006_data_s1_and_s2.zip › Supplementary Dataset 1-LCMS DATA/LCMS PNA Hexamers A-T/LCMS A6 50C_80C/50C/24h/CPT22010446-13-A2-50deg-24h.pdf]

Data -> C:\USERS\PUBLIC\DOCUMENTS\CHEMSTATION\1\DATA\SE02NOV 2023-11-02 14-31-42\ ->
Sample-> CPT22010446-13-A2-50deg-24h

Injection Date :	Thu, 2. Nov. 2023	Seq Line :	11
		Location :	12
		Inj. Vol. :	2 µl

Acq. Method : C:\Users\Public\Documents\ChemStation\1\Data\SE02NOV 2023-11-02 14-31-42\22010446 LCMS-6.M
Analysis Method : C:\Users\Public\Documents\ChemStation\1\Data\SE02NOV 2023-11-02 14-31-42\22010446 LCMS-6.M (Sequence Method)
Waters XBridge Phenyl (4.6 * 150 mm; 3.5 µm); 0.05% TFA (aq) / AcN: 100/0 (0.0 min) --> (6.0 min) --> 70/30 (0.0 min) --> (2.0 min) --> 10/90 (2.0 min); Flow: 1.0 ml/min;
MSD1 = positive; MSD2 = negative

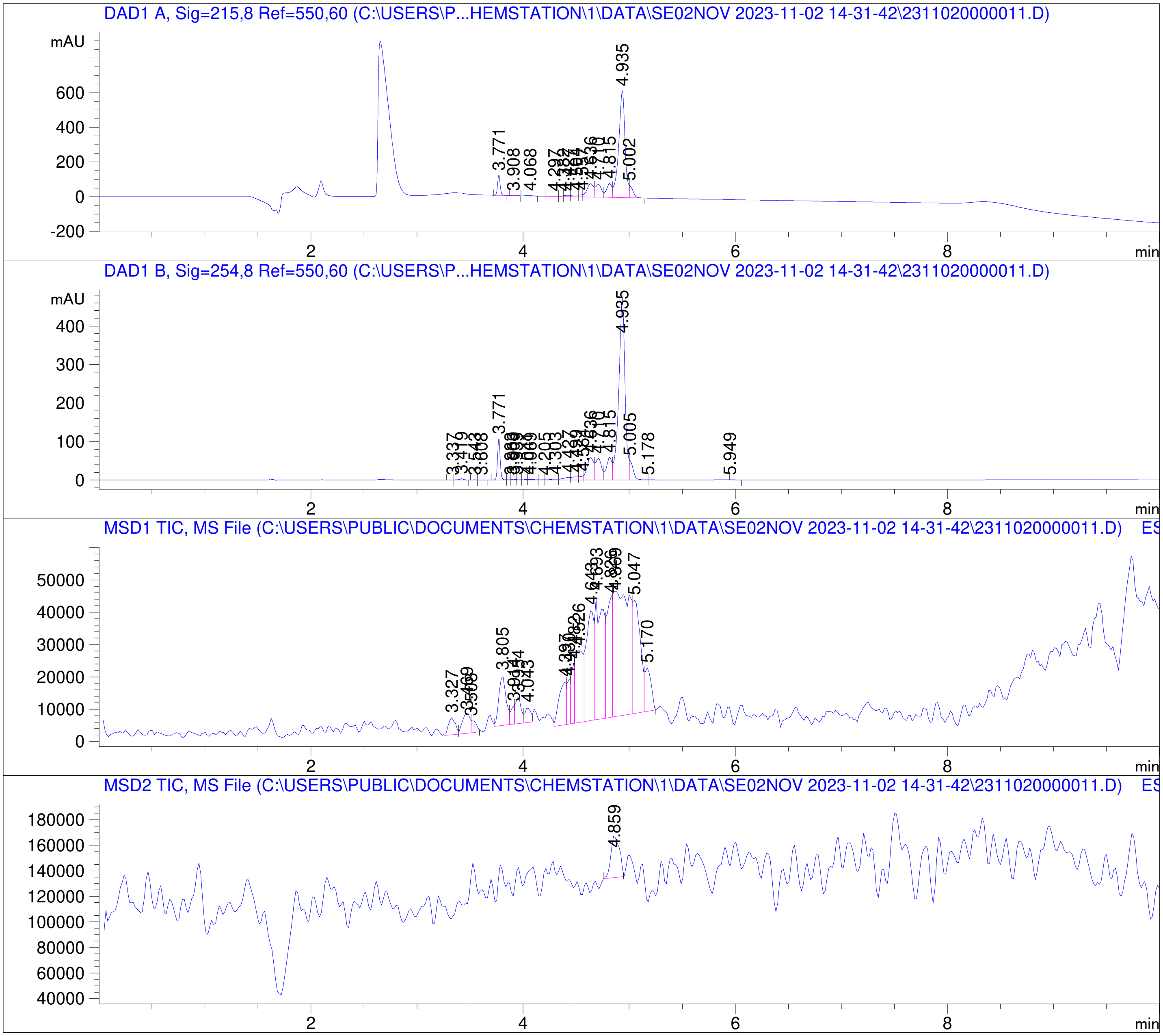

DAD1 A, Sig=215,8 Ref=550,60

Peak #	Ret. Time [min]	Area [mV *s]	Area %
1	3.771	197.992	4.974
2	3.908	3.896	0.098
3	4.068	8.249	0.207
4	4.297	7.341	0.184
5	4.382	7.580	0.190
6	4.427	27.707	0.696
7	4.504	41.417	1.041
8	4.557	21.469	0.539
9	4.636	348.794	8.763
10	4.710	293.710	7.379
11	4.815	284.868	7.157
12	4.935	2578.843	64.789
13	5.002	158.496	3.982

DAD1 B, Sig=254,8 Ref=550,60

Peak #	Ret. Time [min]	Area [mV *s]	Area %
1	3.337	0.441	0.014
2	3.419	8.390	0.273
3	3.543	0.580	0.019
4	3.608	0.494	0.016
5	3.771	182.761	5.939
6	3.883	1.349	0.044
7	3.909	4.202	0.137
8	3.939	1.770	0.058
9	4.041	3.228	0.105
10	4.069	6.612	0.215
11	4.205	1.499	0.049
12	4.303	10.461	0.340
13	4.427	31.016	1.008
14	4.499	33.050	1.074
15	4.564	22.987	0.747
16	4.636	260.418	8.462
17	4.710	222.027	7.215
18	4.815	210.512	6.841
19	4.935	1962.768	63.780
20	5.005	110.659	3.596
21	5.178	1.030	0.033
22	5.949	1.134	0.037

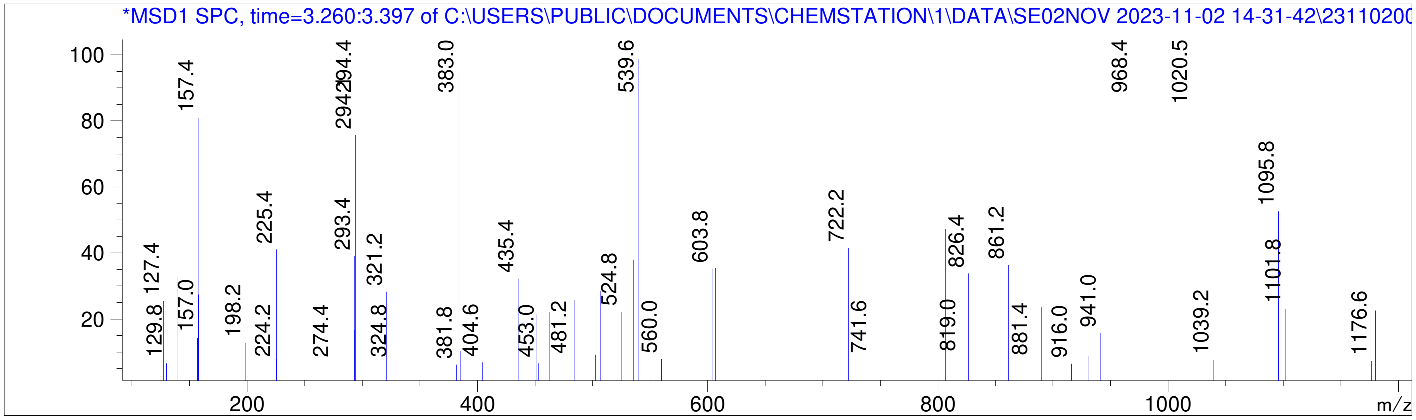
*MSD1 SPC, time=3.260:3.397 of C:\USERS\PUBLIC\DOCUMENTS\CHEMSTATION\1\DATA\SE02NOV 2023-11-02 14-31-42\2311020
127.4
129.8
157.4
157.0
198.2
225.4
224.2
274.4
294.4
293.4
321.2
324.8
383.0
381.8
404.6
435.4
453.0
481.2
524.8
539.6
560.0
603.8
722.2
741.6
826.4
819.0
861.2
881.4
916.0
941.0
968.4
1020.5
1039.2
1095.8
1101.8
1176.6
m/z

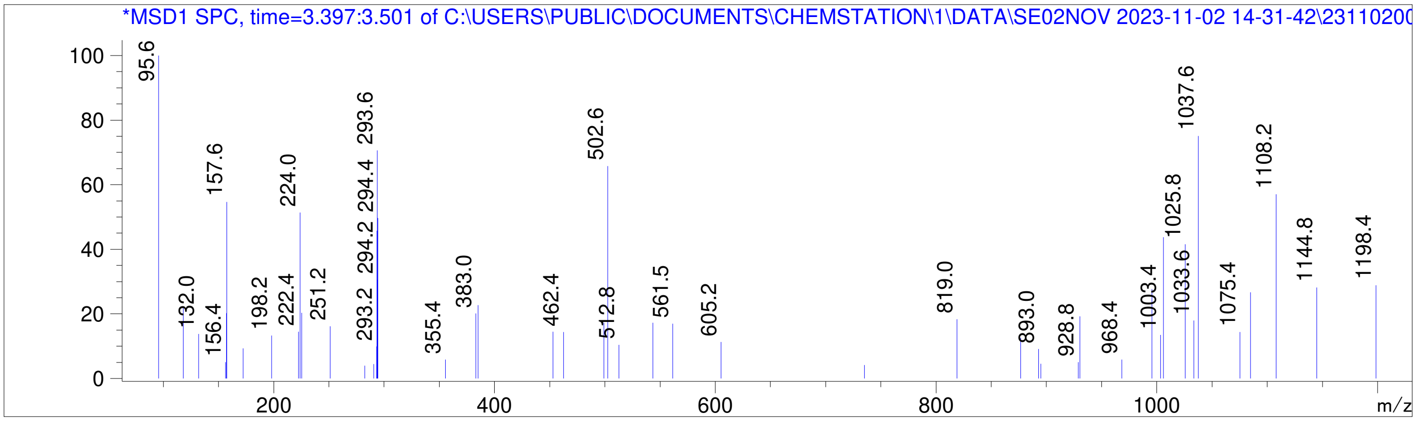
*MSD1 SPC, time=3.397:3.501 of C:\USERS\PUBLIC\DOCUMENTS\CHEMSTATION\1\DATA\SE02NOV 2023-11-02 14-31-42\2311020
95.6
132.0
157.6
156.4
198.2
224.0
222.4
251.2
293.6
294.4
294.2
293.2
355.4
383.0
462.4
502.6
512.8
561.5
605.2
819.0
893.0
928.8
968.4
1003.4
1025.8
1033.6
1037.6
1075.4
1108.2
1144.8
1198.4
m/z

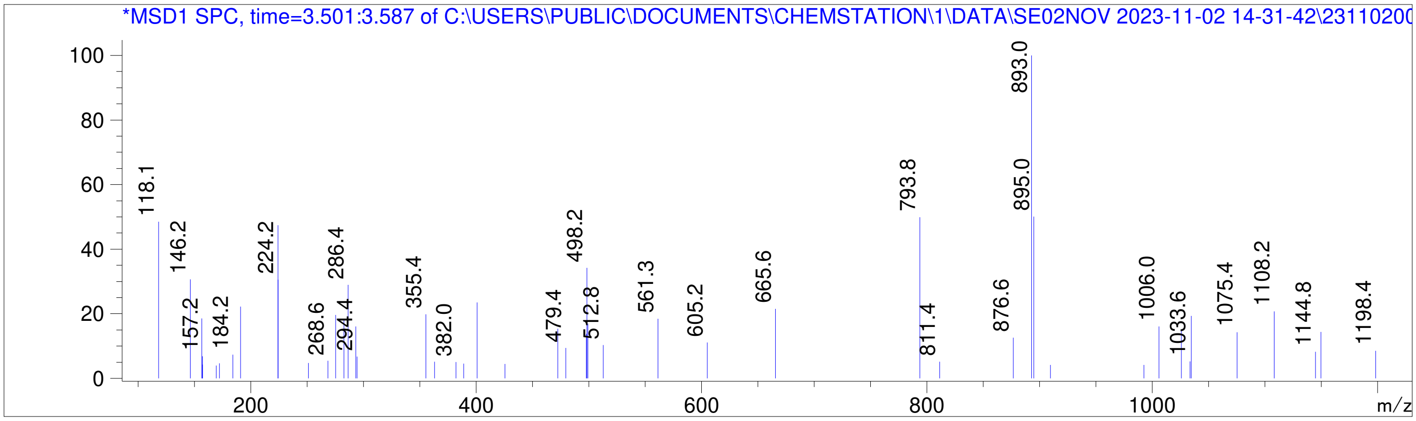
*MSD1 SPC, time=3.501:3.587 of C:\USERS\PUBLIC\DOCUMENTS\CHEMSTATION\1\DATA\SE02NOV 2023-11-02 14-31-42\2311020
118.1
146.2
157.2
184.2
224.2
268.6
286.4
294.4
355.4
382.0
479.4
498.2
512.8
561.3
605.2
665.6
793.8
811.4
876.6
893.0
895.0
1006.0
1033.6
1075.4
1108.2
1144.8
1198.4
m/z

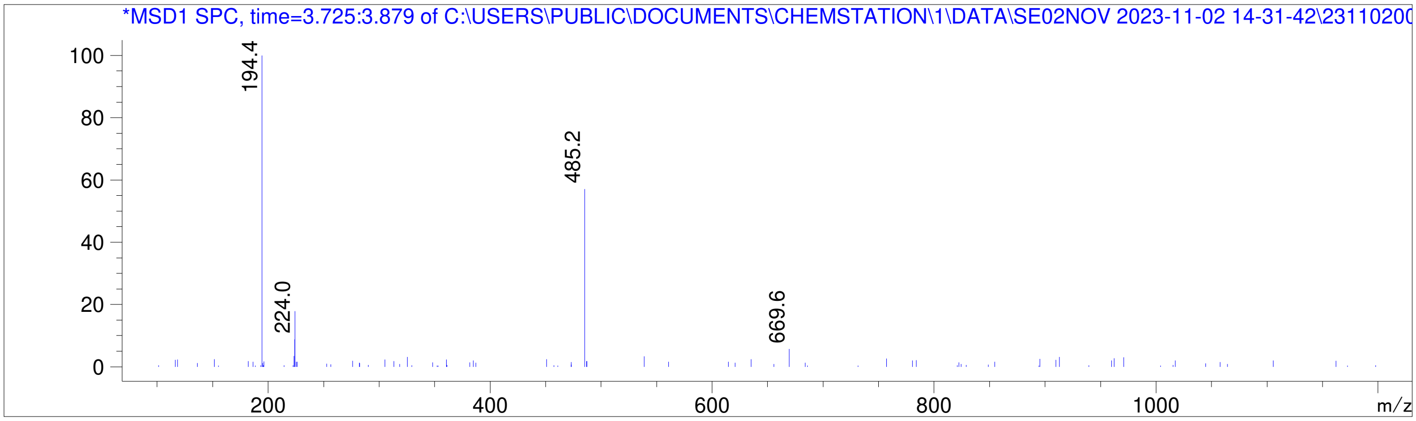
*MSD1 SPC, time=3.725:3.879 of C:\USERS\PUBLIC\DOCUMENTS\CHEMSTATION\1\DATA\SE02NOV 2023-11-02 14-31-42\2311020
194.4
485.2
224.0
669.6
m/z

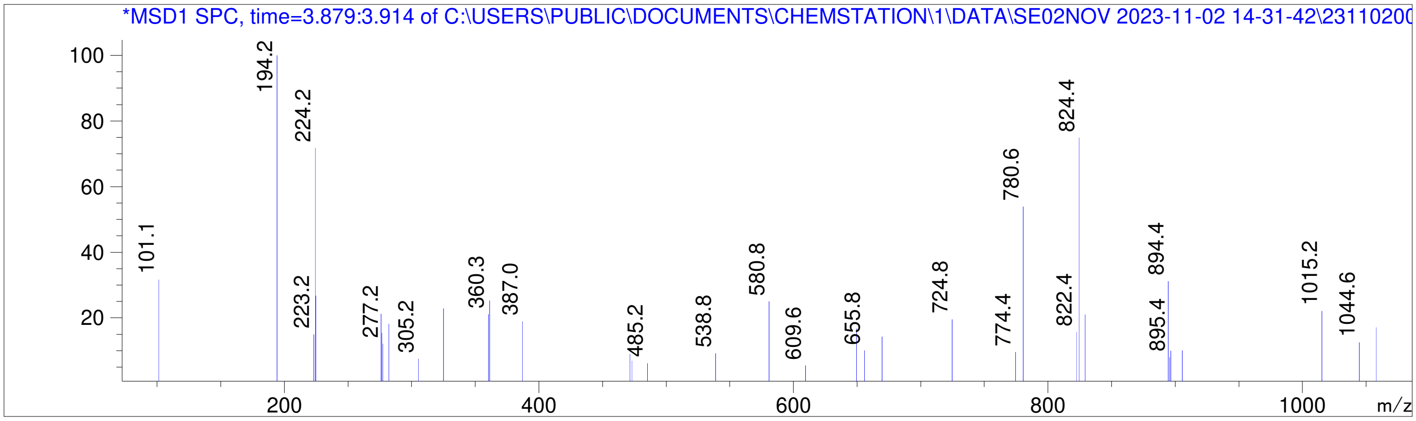
*MSD1 SPC, time=3.879:3.914 of C:\USERS\PUBLIC\DOCUMENTS\CHEMSTATION\1\DATA\SE02NOV 2023-11-02 14-31-42\2311020
194.2
224.2
824.4
780.6
101.1
580.8
894.4
1015.2
360.3
387.0
724.8
1044.6
223.2
277.2
305.2
485.2
538.8
609.6
655.8
774.4
822.4
895.4
m/z

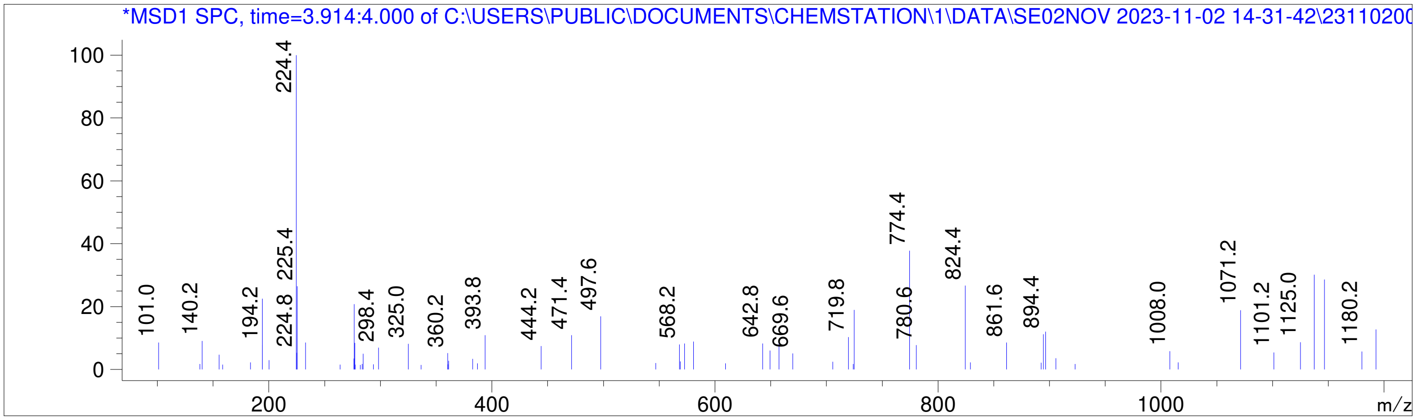
*MSD1 SPC, time=3.914:4.000 of C:\USERS\PUBLIC\DOCUMENTS\CHEMSTATION\1\DATA\SE02NOV 2023-11-02 14-31-42\2311020
224.4
774.4
225.4
824.4
1071.2
101.0
140.2
194.2
224.8
298.4
325.0
360.2
393.8
444.2
471.4
497.6
568.2
642.8
669.6
719.8
780.6
861.6
894.4
1008.0
1101.2
1125.0
1180.2
m/z

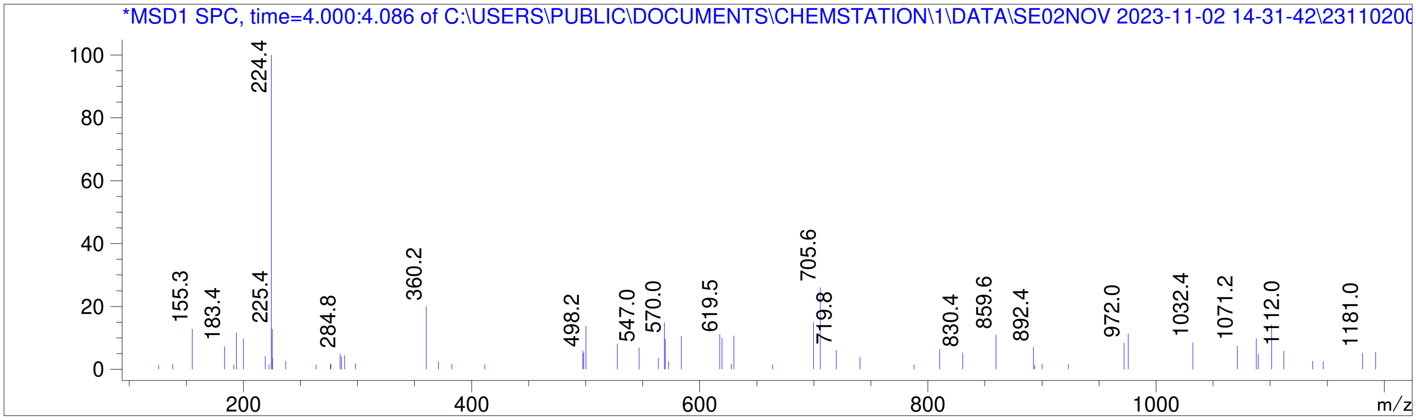
*MSD1 SPC, time=4.000:4.086 of C:\USERS\PUBLIC\DOCUMENTS\CHEMSTATION\1\DATA\SE02NOV 2023-11-02 14-31-42\2311020
224.4
705.6
155.3
183.4
225.4
284.8
360.2
498.2
547.0
570.0
619.5
719.8
830.4
859.6
892.4
972.0
1032.4
1071.2
1112.0
1181.0
m/z

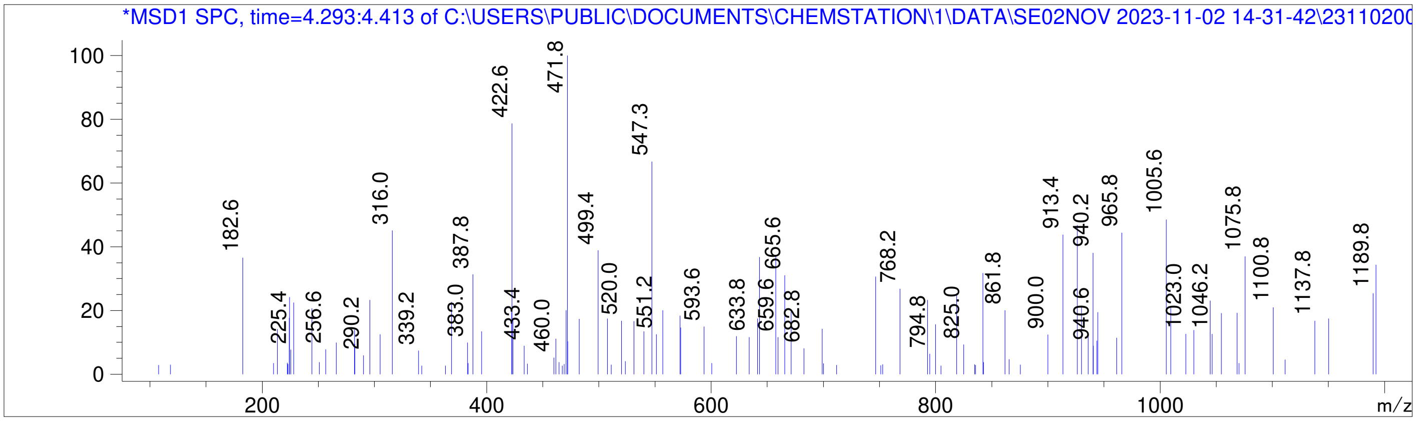
*MSD1 SPC, time=4.293:4.413 of C:\USERS\PUBLIC\DOCUMENTS\CHEMSTATION\1\DATA\SE02NOV 2023-11-02 14-31-42\2311020
182.6
225.4
256.6
290.2
316.0
339.2
383.0
387.8
422.6
433.4
460.0
471.8
499.4
520.0
547.3
551.2
593.6
633.8
659.6
665.6
682.8
768.2
794.8
825.0
861.8
900.0
913.4
940.2
940.6
965.8
1005.6
1023.0
1046.2
1075.8
1100.8
1137.8
1189.8
m/z

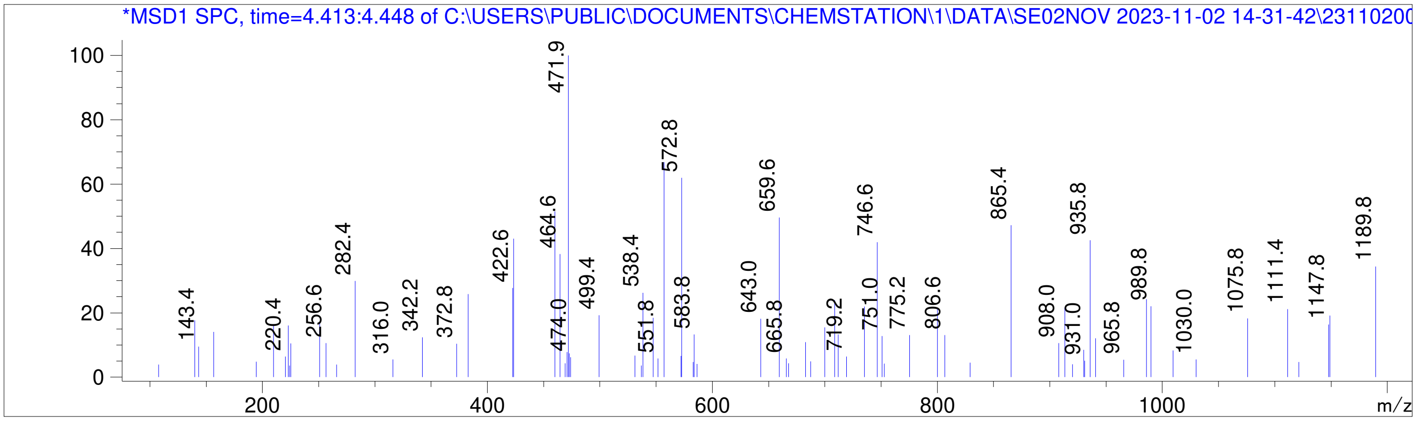
*MSD1 SPC, time=4.413:4.448 of C:\USERS\PUBLIC\DOCUMENTS\CHEMSTATION\1\DATA\SE02NOV 2023-11-02 14-31-42\2311020
143.4
220.4
256.6
282.4
316.0
342.2
372.8
422.6
464.6
474.0
471.9
499.4
538.4
551.8
572.8
583.8
643.0
659.6
665.8
719.2
746.6
751.0
775.2
806.6
865.4
908.0
931.0
935.8
965.8
989.8
1030.0
1075.8
1111.4
1147.8
1189.8
m/z

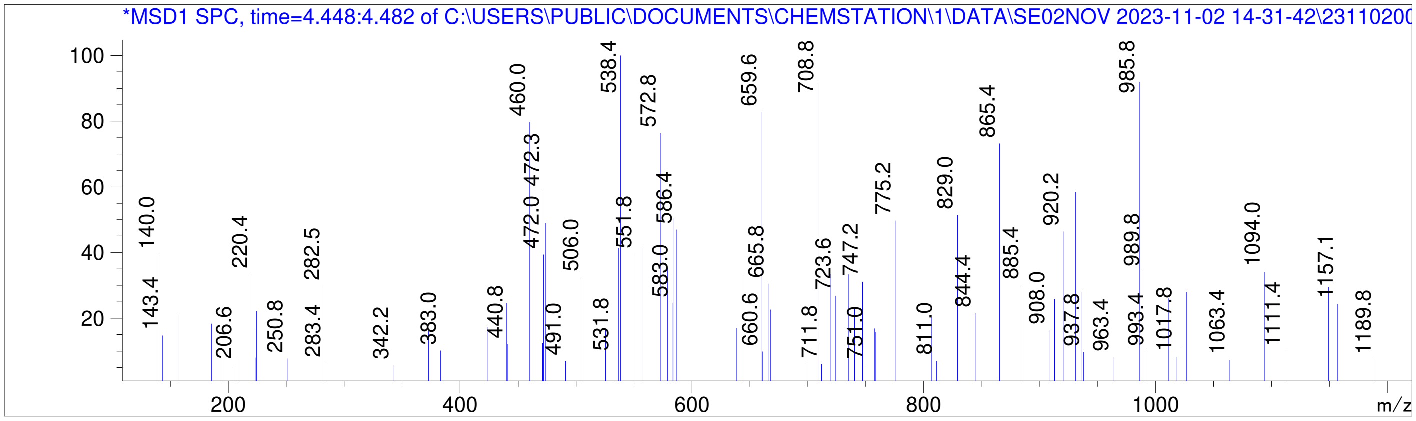
*MSD1 SPC, time=4.448:4.482 of C:\USERS\PUBLIC\DOCUMENTS\CHEMSTATION\1\DATA\SE02NOV 2023-11-02 14-31-42\2311020
140.0
143.4
206.6
220.4
250.8
282.5
283.4
342.2
383.0
440.8
460.0
472.0
472.3
491.0
506.0
531.8
538.4
551.8
572.8
583.0
586.4
659.6
660.6
665.8
708.8
711.8
723.6
747.2
751.0
775.2
811.0
829.0
844.4
865.4
885.4
908.0
920.2
937.8
963.4
985.8
989.8
993.4
1017.8
1063.4
1094.0
1111.4
1157.1
1189.8
m/z

Sample-> CPT22010446-13-A2-50deg-24h

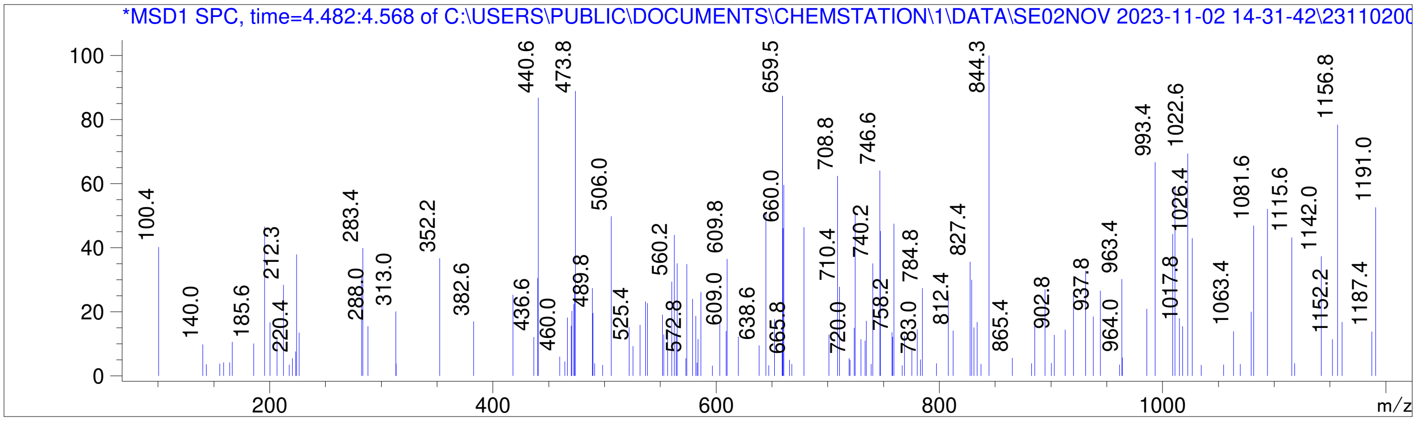

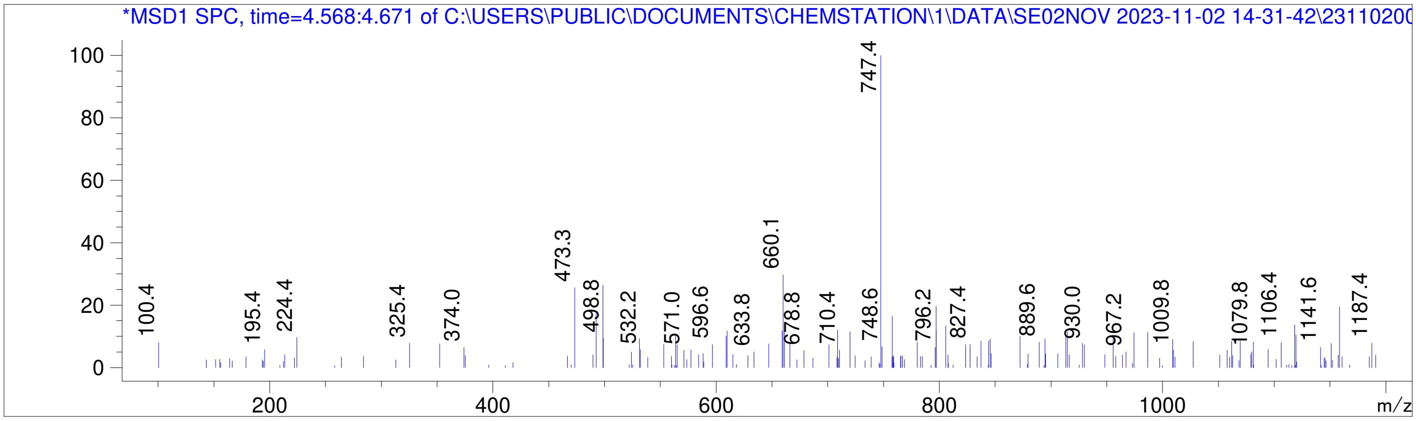

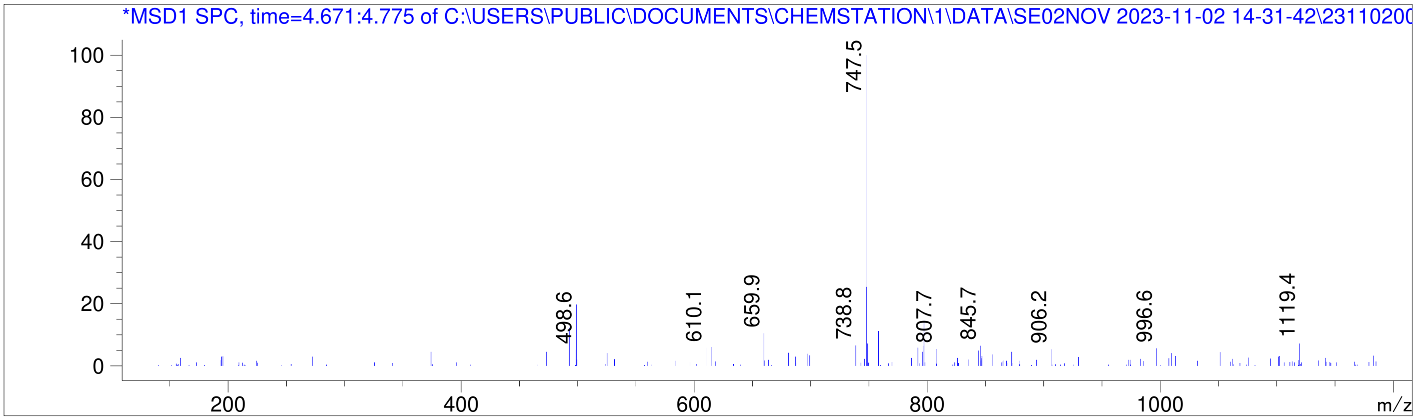

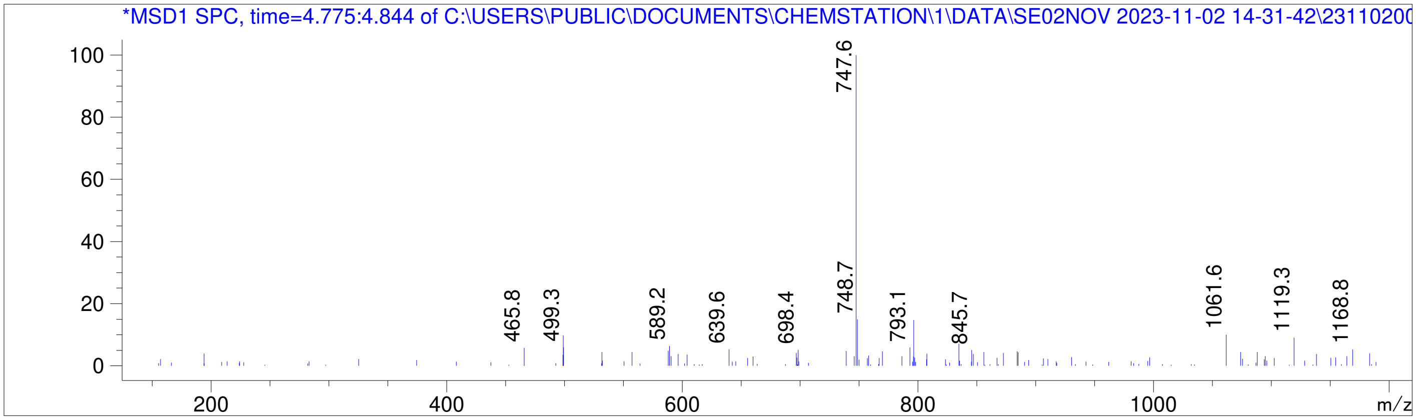

Sample-> CPT22010446-13-A2-50deg-24h

==

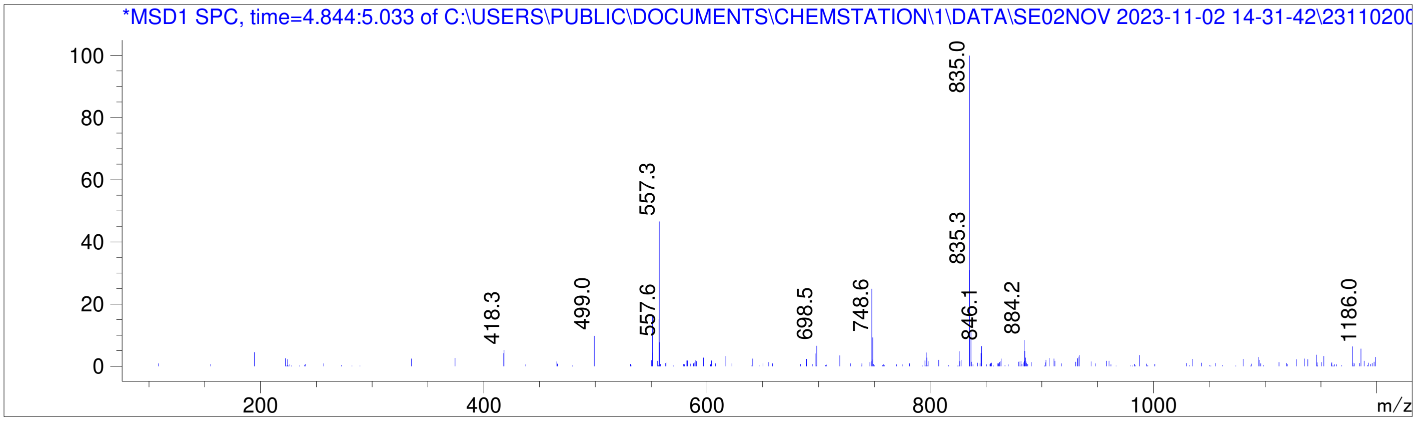

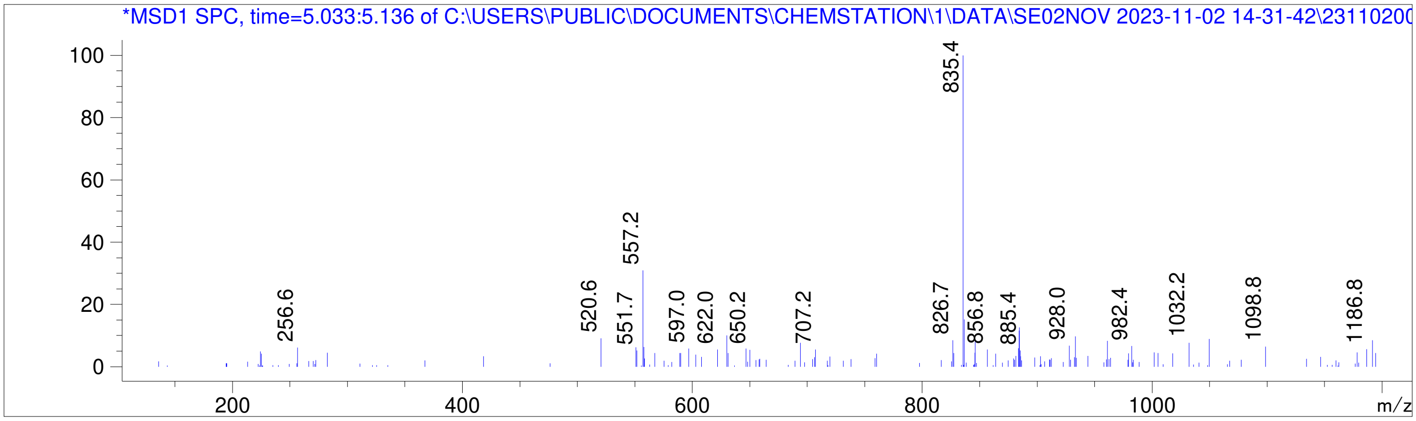

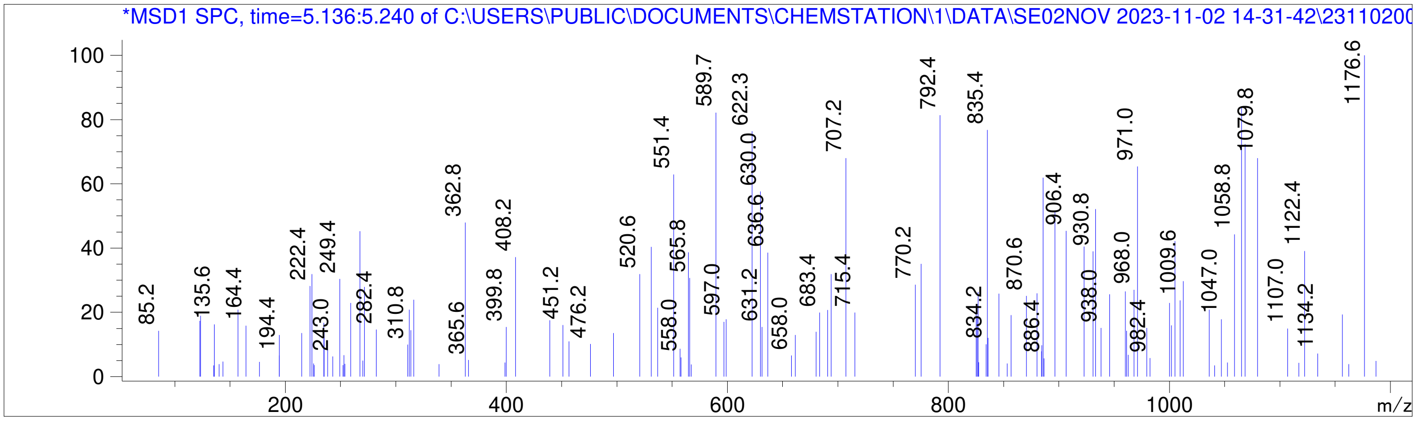

==

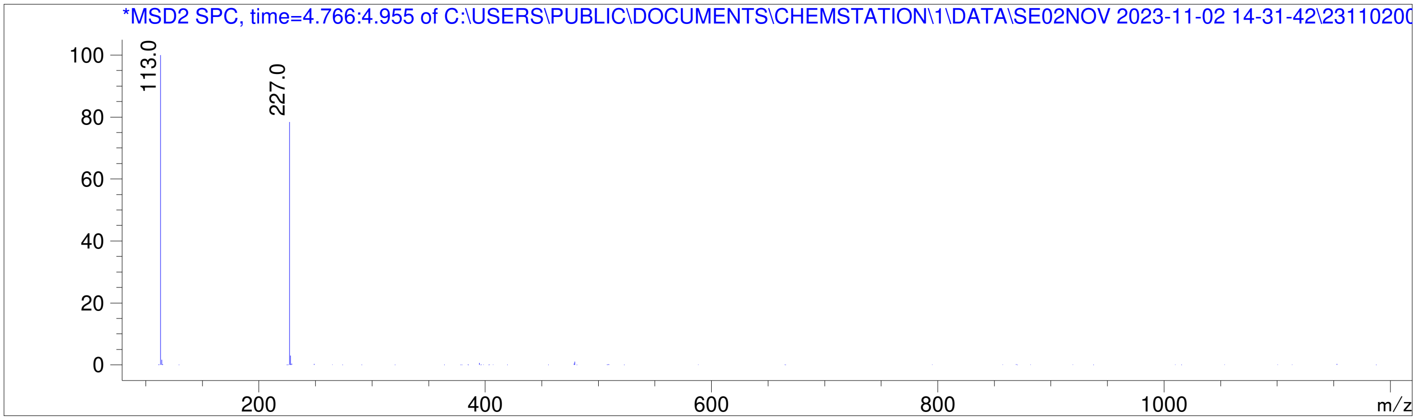
*MSD2 SPC, time=4.766:4.955 of C:\USERS\PUBLIC\DOCUMENTS\CHEMSTATION\1\DATA\SE02NOV 2023-11-02 14-31-42\2311020
113.0
227.0
100
80
60
40
20
0
200
400
600
800
1000
m/z
