# Supplementary material for: Astrobiological implications of the stability and reactivity of peptide nucleic acid (PNA) in concentrated sulfuric acid
Source: Sci Adv. 2025 Mar 26;11(13):eadr0006. doi: 10.1126/sciadv.adr0006 (PMC11939054; doi:10.1126/sciadv.adr0006)
Supplement: Supplementary file 2 — Data S1 and S2 [file sciadv.adr0006_data_s1_and_s2.zip › Supplementary Dataset 1-LCMS DATA/LCMS PNA Hexamers A-T/LCMS A6 50C_80C/80C/1h/CPT22010446-13-A1-80dg-1h.pdf]

Data -> C:\USERS\PUBLIC\DOCUMENTS\CHEMSTATION\1\DATA\SE19OCT 2023-10-19 16-56-54\ ->
Sample-> CPT22010446-13-A1-80dg-1h

===

Injection Date : Fri, 20. Oct. 2023   Seq Line : 35
Location : 10
Inj. Vol. : 2 µl

Acq. Method   : C:\Users\Public\Documents\ChemStation\1\Data\SE19OCT 2023-10-19 16-56-54\22010446 LCMS-6.M
Analysis Method : C:\Users\Public\Documents\ChemStation\1\Data\SE19OCT 2023-10-19 16-56-54\22010446 LCMS-6.M (Sequence Method)
Waters XBridge Phenyl (4.6 * 150 mm; 3.5 µm); 0.05% TFA (aq) / AcN: 100/0 (0.0 min) --> (6.0 min) --> 70/30 (0.0 min) --> (2.0 min) --> 10/90 (2.0 min); Flow: 1.0 ml/min;
MSD1 = positive; MSD2 = negative

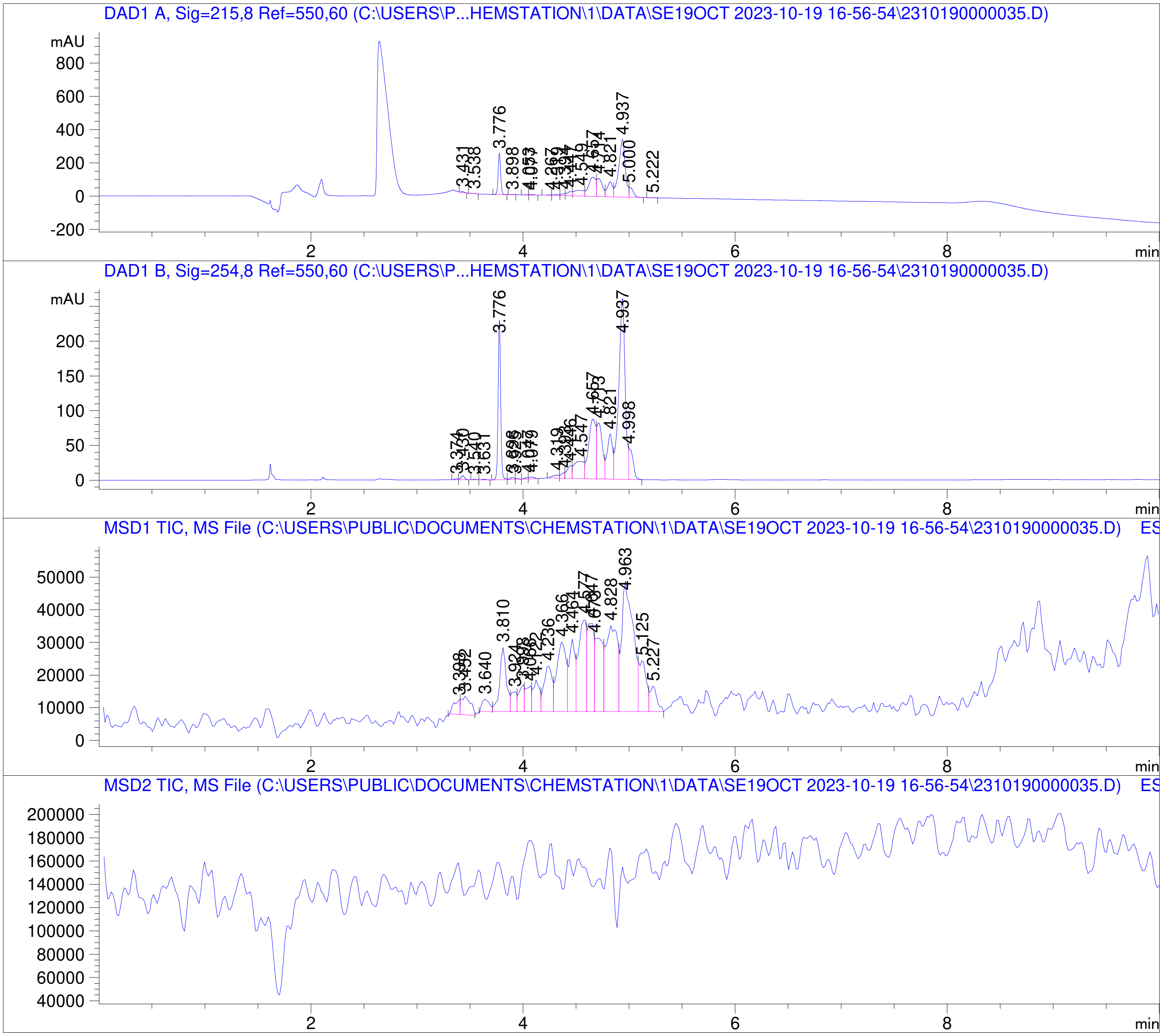

DAD1 A, Sig=215,8 Ref=550,60

Peak #	Ret. Time [min]	Area [mV *s]	Area %
1	3.431	9.521	0.256
2	3.538	1.885	0.051
3	3.776	415.683	11.162
4	3.898	4.114	0.110
5	4.053	7.392	0.198
6	4.077	11.587	0.311
7	4.267	9.063	0.243
8	4.319	26.627	0.715
9	4.394	27.412	0.736
10	4.447	84.068	2.257
11	4.549	221.936	5.960
12	4.657	547.298	14.697
13	4.714	396.815	10.656
14	4.821	320.728	8.613
15	4.937	1482.382	39.807
16	5.000	155.715	4.181
17	5.222	1.726	0.046

DAD1 B, Sig=254,8 Ref=550,60

Peak #	Ret. Time [min]	Area [mV *s]	Area %
1	3.374	2.822	0.099
2	3.430	13.421	0.472
3	3.540	1.121	0.039
4	3.631	1.965	0.069
5	3.776	385.377	13.560
6	3.898	7.194	0.253
7	3.925	2.600	0.091
8	4.047	4.291	0.151
9	4.079	8.816	0.310
10	4.319	18.840	0.663
11	4.393	19.398	0.683
12	4.446	60.300	2.122
13	4.547	160.147	5.635
14	4.657	416.857	14.667
15	4.713	283.673	9.981
16	4.821	234.100	8.237
17	4.937	1101.377	38.752
18	4.998	119.782	4.215

==

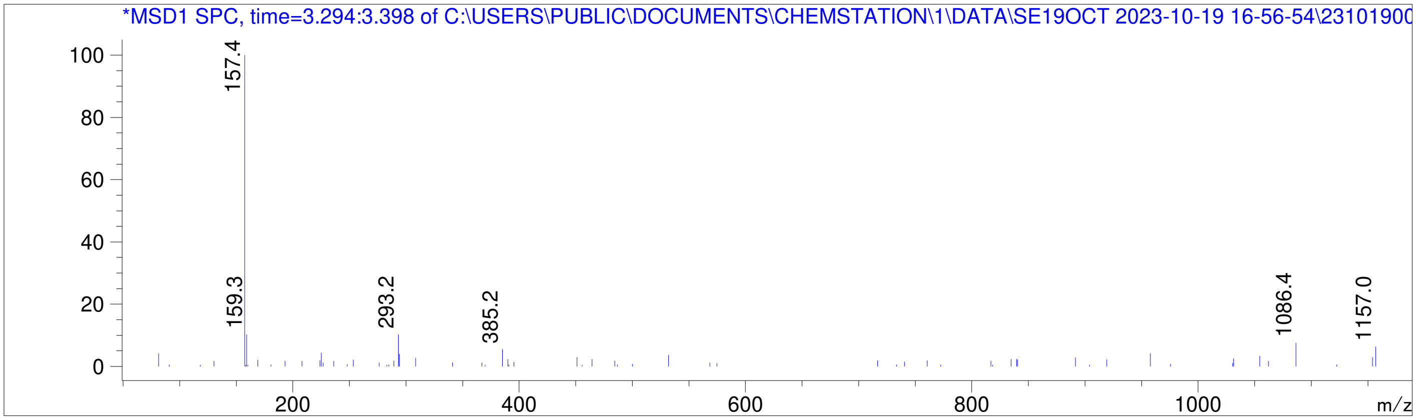
*MSD1 SPC, time=3.294:3.398 of C:\USERS\PUBLIC\DOCUMENTS\CHEMSTATION\1\DATA\SE19OCT 2023-10-19 16-56-54\23101900
157.4
159.3
293.2
385.2
1086.4
1157.0
m/z

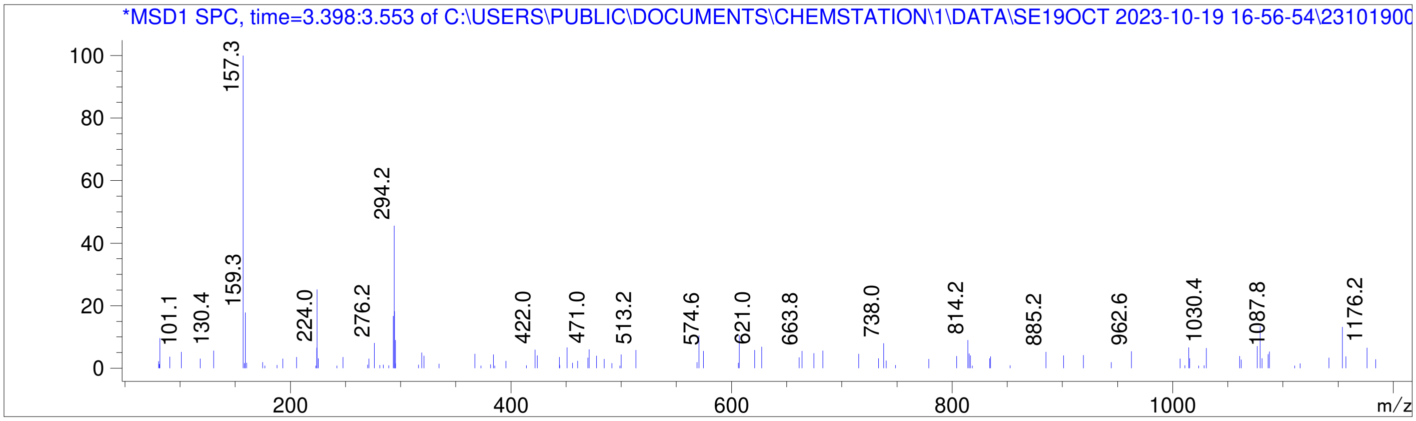
*MSD1 SPC, time=3.398:3.553 of C:\USERS\PUBLIC\DOCUMENTS\CHEMSTATION\1\DATA\SE19OCT 2023-10-19 16-56-54\23101900
157.3
294.2
101.1
130.4
159.3
224.0
276.2
422.0
471.0
513.2
574.6
621.0
663.8
738.0
814.2
885.2
962.6
1030.4
1087.8
1176.2
m/z

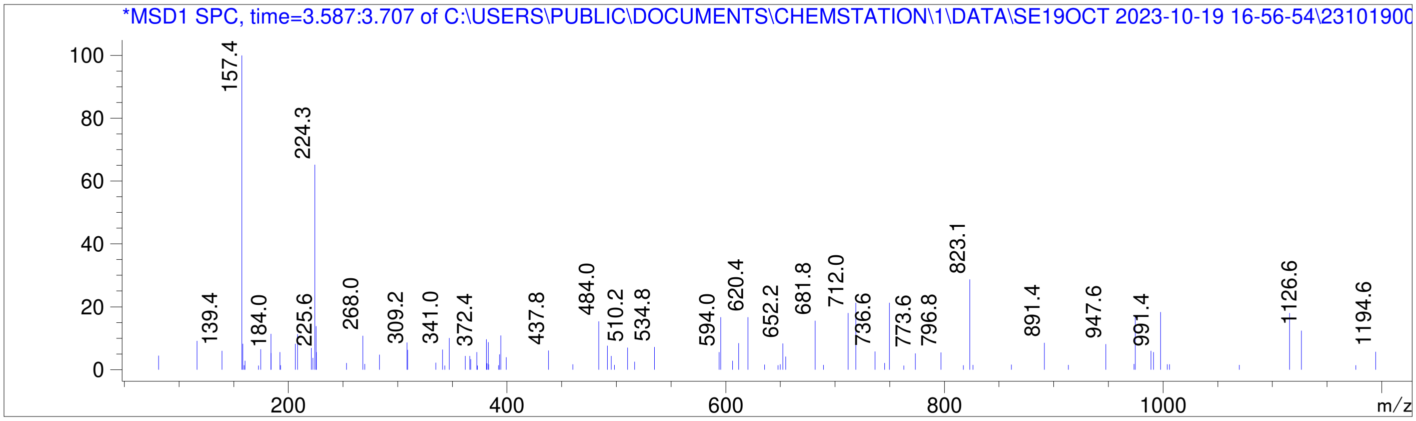
*MSD1 SPC, time=3.587:3.707 of C:\USERS\PUBLIC\DOCUMENTS\CHEMSTATION\1\DATA\SE19OCT 2023-10-19 16-56-54\23101900
157.4
224.3
139.4
184.0
225.6
268.0
309.2
341.0
372.4
437.8
484.0
510.2
534.8
594.0
620.4
652.2
681.8
712.0
736.6
773.6
796.8
823.1
891.4
947.6
991.4
1126.6
1194.6
m/z

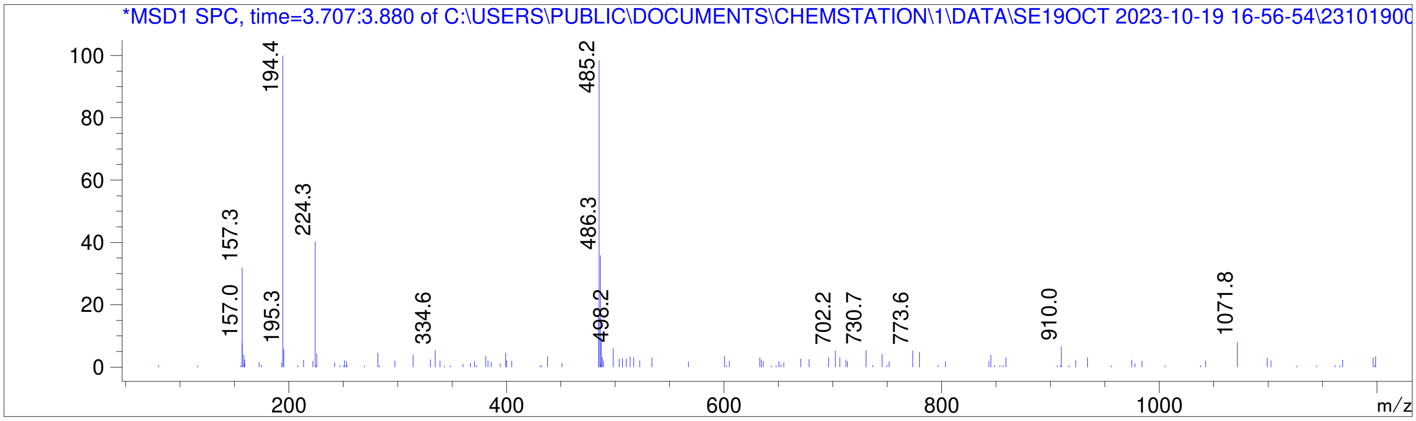
*MSD1 SPC, time=3.707:3.880 of C:\USERS\PUBLIC\DOCUMENTS\CHEMSTATION\1\DATA\SE19OCT 2023-10-19 16-56-54\23101900
194.4
485.2
157.3
224.3
486.3
157.0
195.3
334.6
498.2
702.2
730.7
773.6
910.0
1071.8
100
80
60
40
20
0
200
400
600
800
1000
m/z

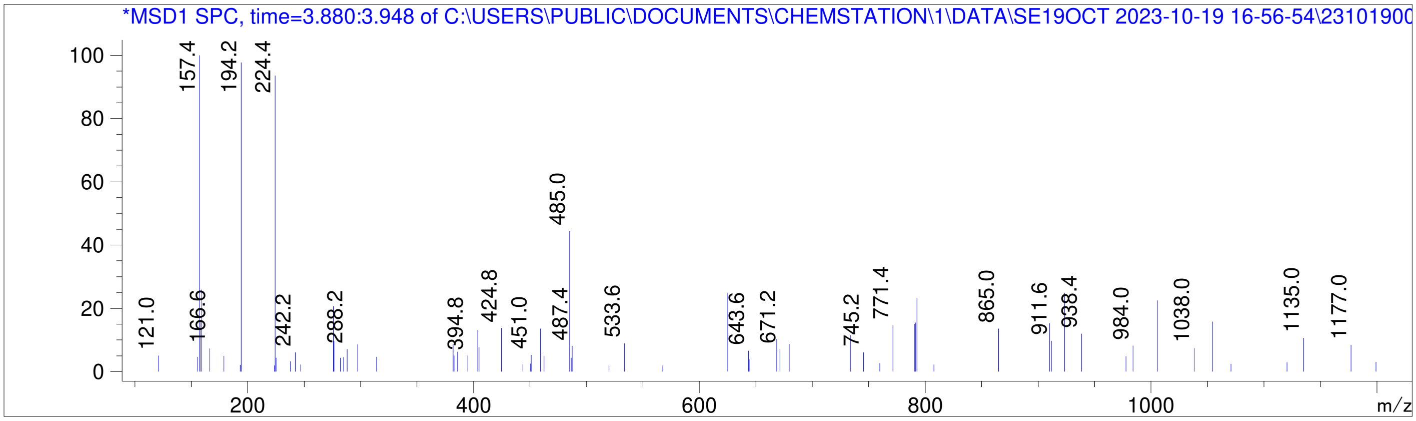
*MSD1 SPC, time=3.880:3.948 of C:\USERS\PUBLIC\DOCUMENTS\CHEMSTATION\1\DATA\SE19OCT 2023-10-19 16-56-54\23101900
157.4
194.2
224.4
485.0
121.0
166.6
242.2
288.2
394.8
424.8
451.0
487.4
533.6
643.6
671.2
745.2
771.4
865.0
911.6
938.4
984.0
1038.0
1135.0
1177.0
100
80
60
40
20
0
200
400
600
800
1000
m/z

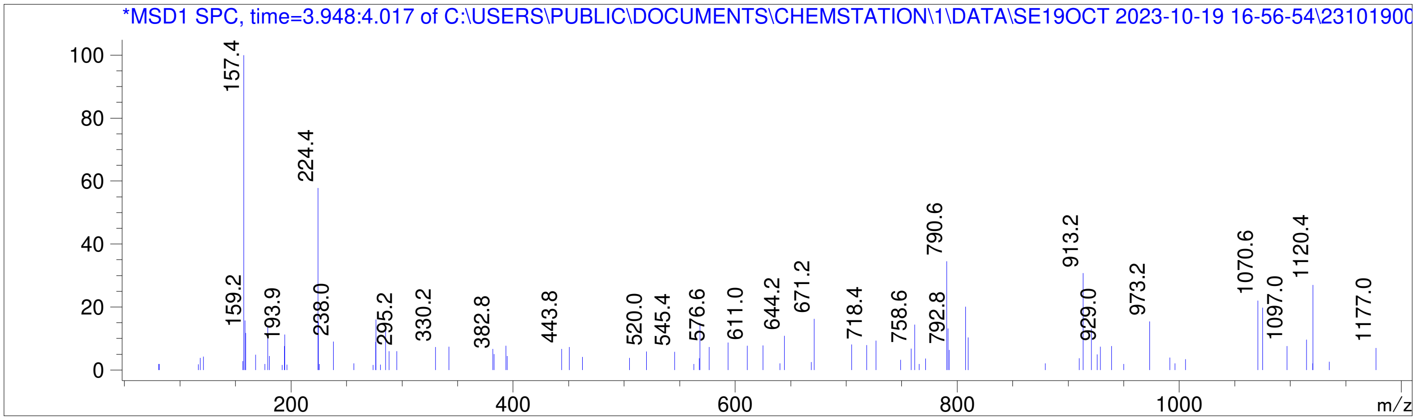
*MSD1 SPC, time=3.948:4.017 of C:\USERS\PUBLIC\DOCUMENTS\CHEMSTATION\1\DATA\SE19OCT 2023-10-19 16-56-54\23101900
157.4
224.4
790.6
913.2
1070.6
1120.4
159.2
193.9
238.0
295.2
330.2
382.8
443.8
520.0
545.4
576.6
611.0
644.2
671.2
718.4
758.6
792.8
929.0
973.2
1097.0
1177.0
100
80
60
40
20
0
200
400
600
800
1000
m/z

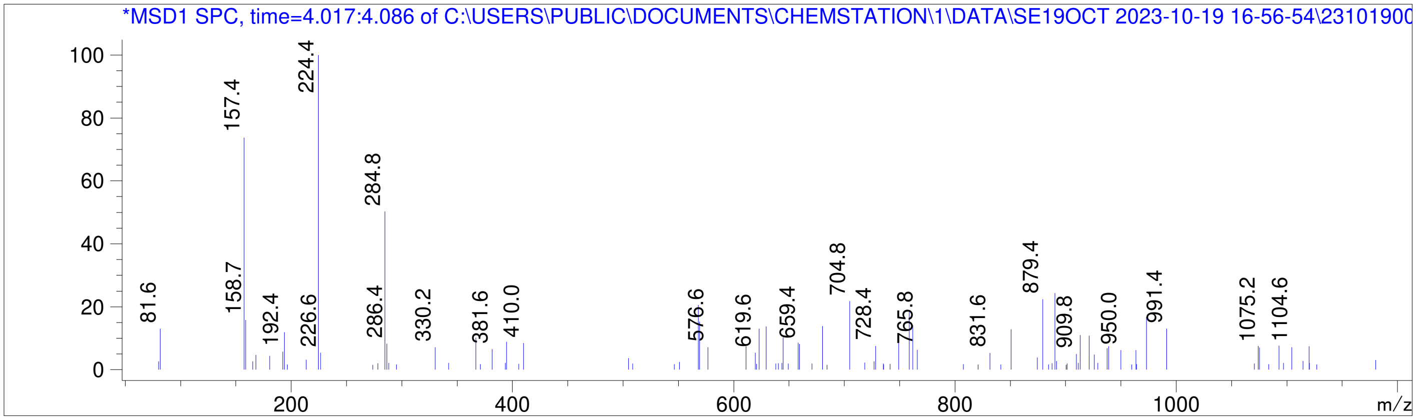
*MSD1 SPC, time=4.017:4.086 of C:\USERS\PUBLIC\DOCUMENTS\CHEMSTATION\1\DATA\SE19OCT 2023-10-19 16-56-54\23101900
224.4
157.4
284.8
81.6
158.7
192.4
226.6
286.4
330.2
381.6
410.0
576.6
619.6
659.4
704.8
728.4
765.8
831.6
879.4
909.8
950.0
991.4
1075.2
1104.6
100
80
60
40
20
0
200
400
600
800
1000
m/z

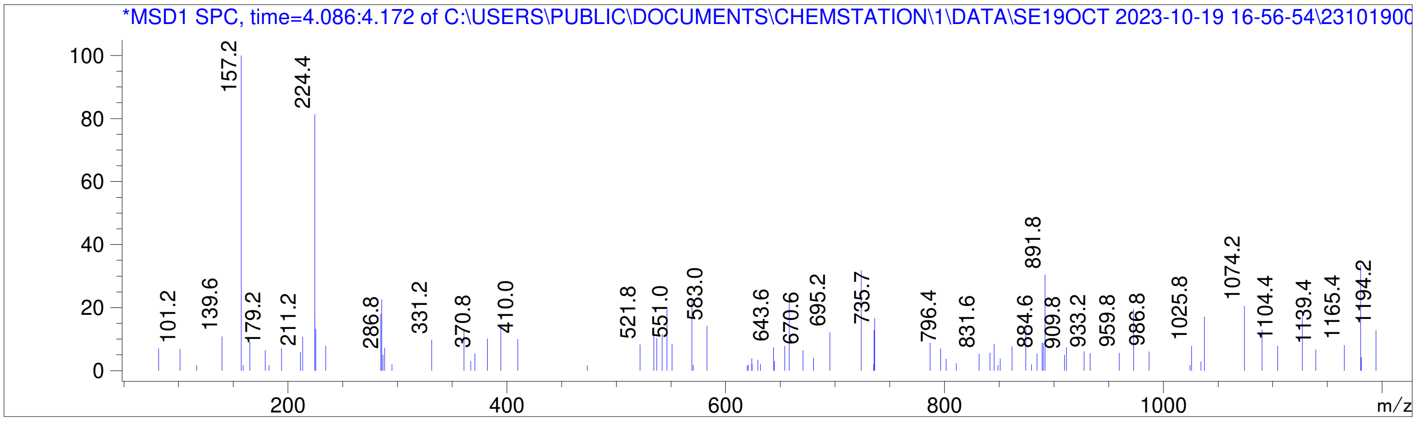
*MSD1 SPC, time=4.086:4.172 of C:\USERS\PUBLIC\DOCUMENTS\CHEMSTATION\1\DATA\SE19OCT 2023-10-19 16-56-54\23101900
m/z

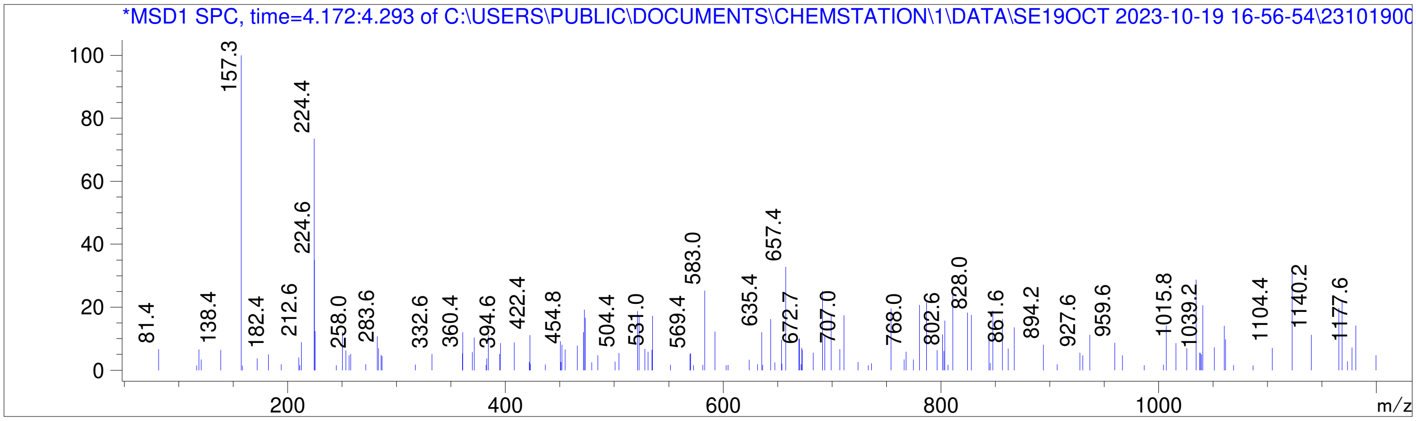
*MSD1 SPC, time=4.172:4.293 of C:\USERS\PUBLIC\DOCUMENTS\CHEMSTATION\1\DATA\SE19OCT 2023-10-19 16-56-54\23101900
m/z

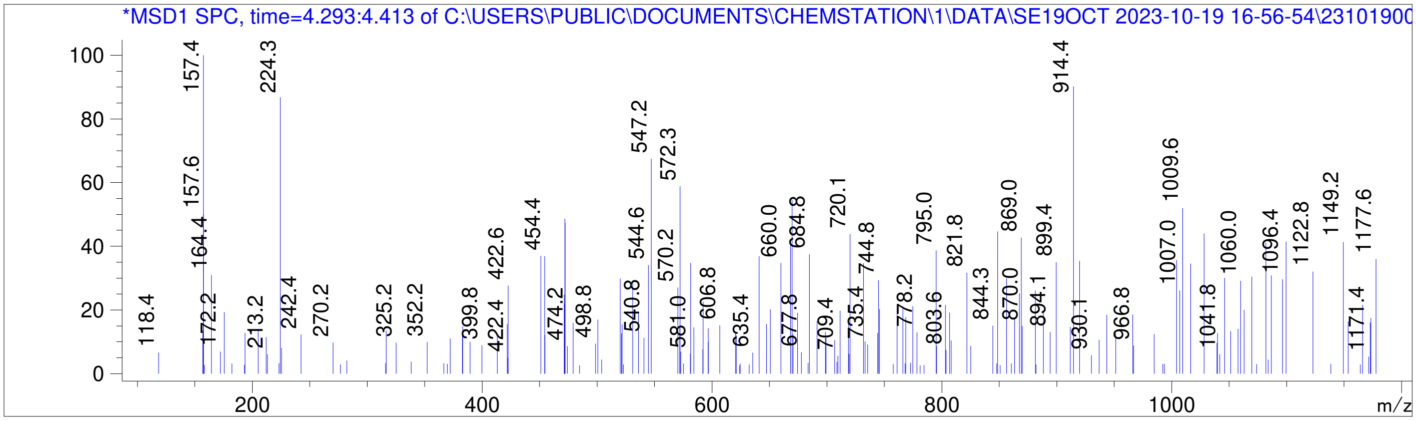
*MSD1 SPC, time=4.293:4.413 of C:\USERS\PUBLIC\DOCUMENTS\CHEMSTATION\1\DATA\SE19OCT 2023-10-19 16-56-54\23101900
m/z

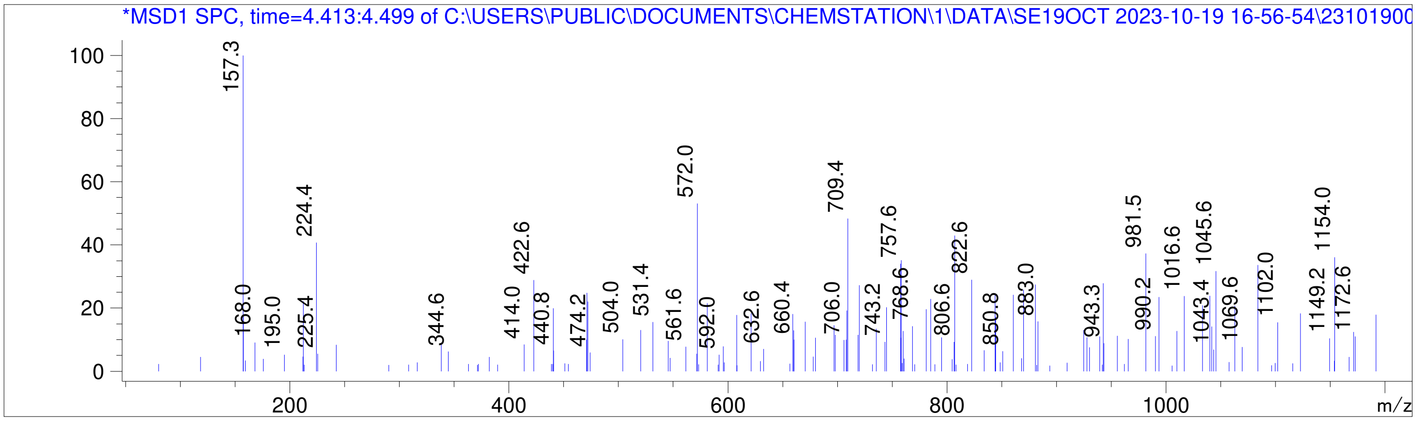
*MSD1 SPC, time=4.413:4.499 of C:\USERS\PUBLIC\DOCUMENTS\CHEMSTATION\1\DATA\SE19OCT 2023-10-19 16-56-54\23101900
157.3
168.0
195.0
224.4
225.4
344.6
414.0
422.6
440.8
474.2
504.0
531.4
561.6
572.0
592.0
632.6
660.4
706.0
709.4
743.2
757.6
768.6
806.6
822.6
850.8
883.0
943.3
981.5
990.2
1016.6
1043.4
1045.6
1069.6
1102.0
1149.2
1154.0
1172.6
m/z

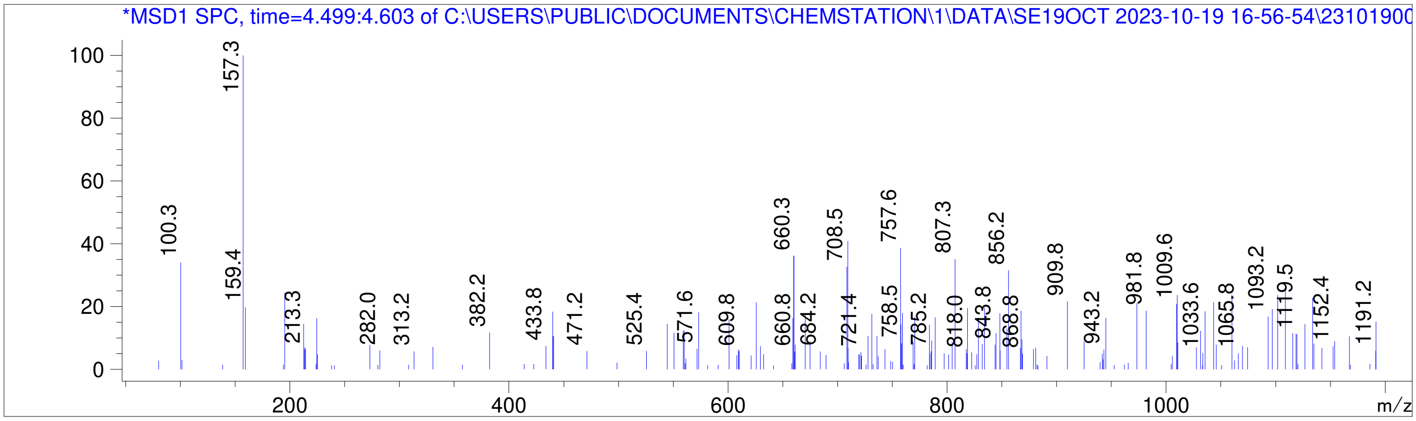
*MSD1 SPC, time=4.499:4.603 of C:\USERS\PUBLIC\DOCUMENTS\CHEMSTATION\1\DATA\SE19OCT 2023-10-19 16-56-54\23101900
100.3
157.3
159.4
213.3
282.0
313.2
382.2
433.8
471.2
525.4
571.6
609.8
660.3
660.8
684.2
708.5
721.4
757.6
758.5
785.2
807.3
818.0
843.8
856.2
868.8
909.8
943.2
981.8
1009.6
1033.6
1065.8
1093.2
1119.5
1152.4
1191.2
m/z

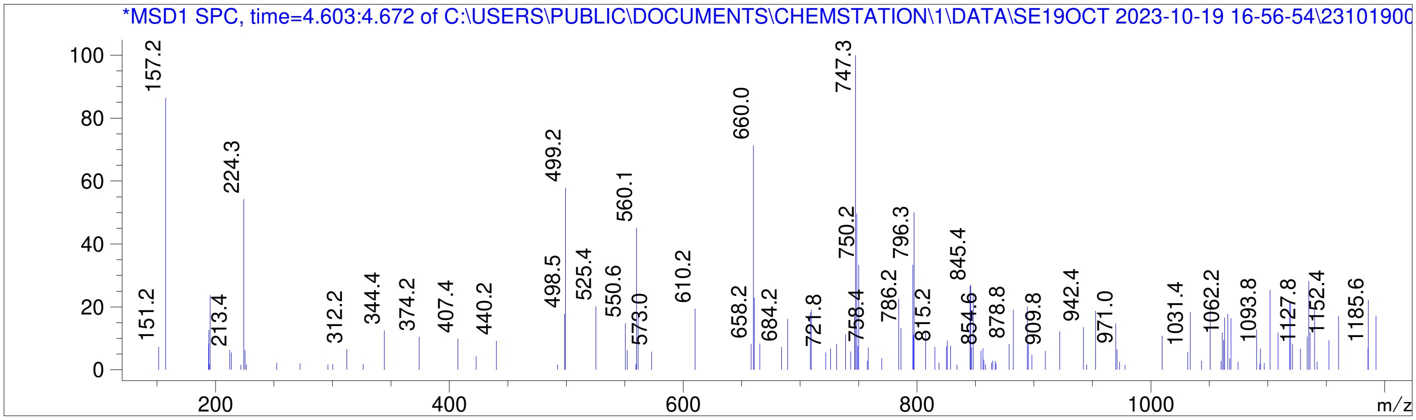
*MSD1 SPC, time=4.603:4.672 of C:\USERS\PUBLIC\DOCUMENTS\CHEMSTATION\1\DATA\SE19OCT 2023-10-19 16-56-54\23101900
151.2
157.2
213.4
224.3
312.2
344.4
374.2
407.4
440.2
498.5
499.2
525.4
550.6
560.1
573.0
610.2
658.2
660.0
684.2
721.8
747.3
750.2
758.4
786.2
796.3
815.2
845.4
854.6
878.8
909.8
942.4
971.0
1031.4
1062.2
1093.8
1127.8
1152.4
1185.6
m/z

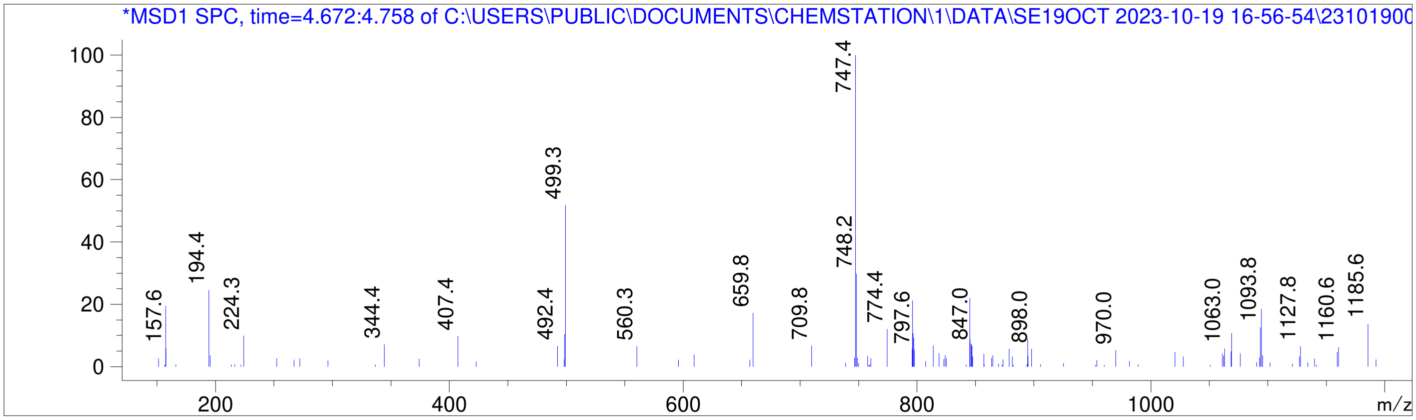
*MSD1 SPC, time=4.672:4.758 of C:\USERS\PUBLIC\DOCUMENTS\CHEMSTATION\1\DATA\SE19OCT 2023-10-19 16-56-54\23101900
157.6
194.4
224.3
344.4
407.4
492.4
499.3
560.3
659.8
709.8
747.4
748.2
774.4
797.6
847.0
898.0
970.0
1063.0
1093.8
1127.8
1160.6
1185.6
m/z

Data -> C:\USERS\PUBLIC\DOCUMENTS\CHEMSTATION\1\DATA\SE19OCT 2023-10-19 16-56-54\   ->
Sample-> CPT22010446-13-A1-80dg-1h
==

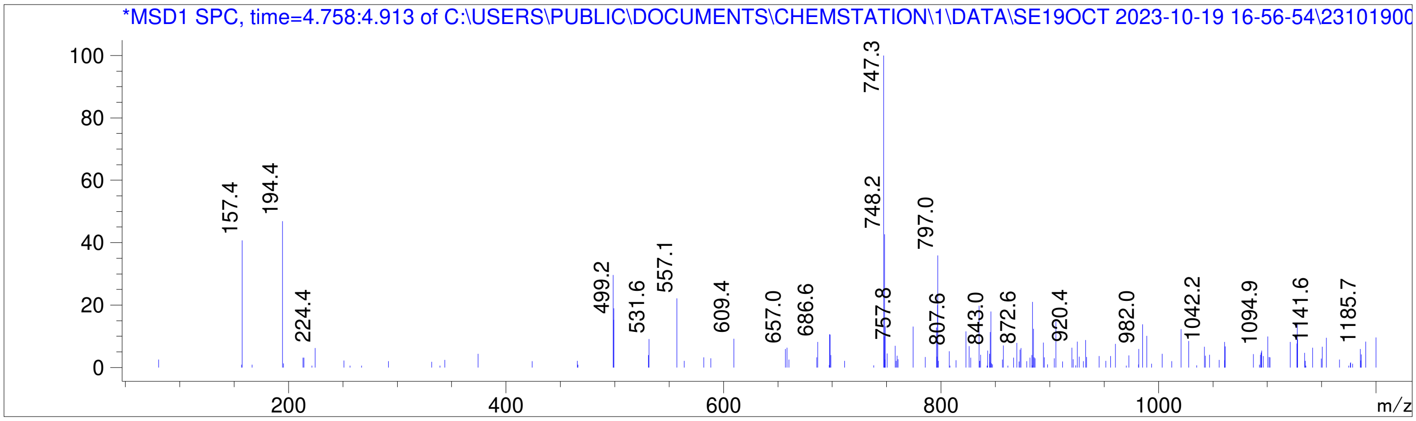

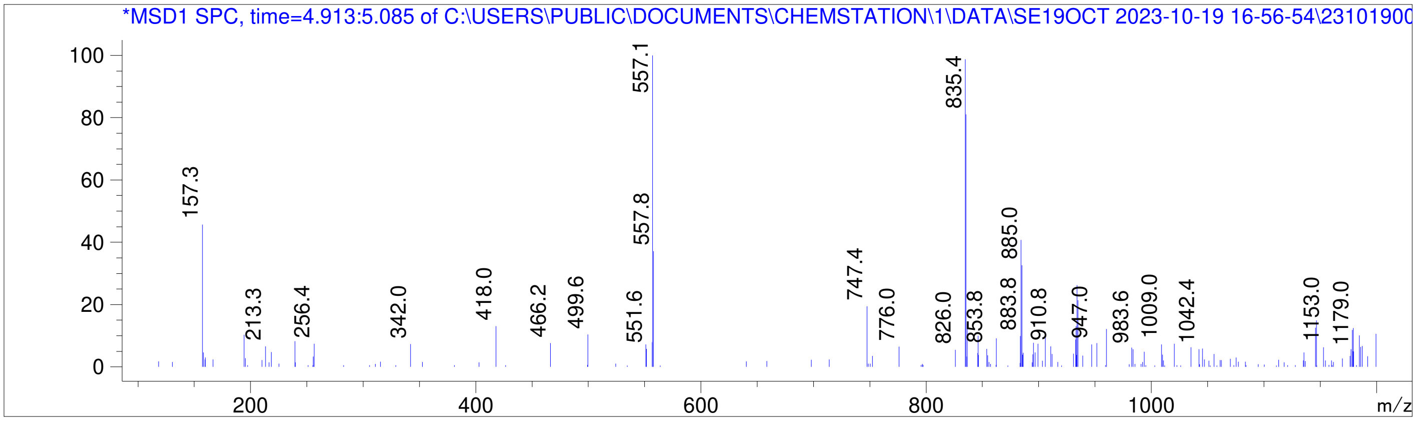

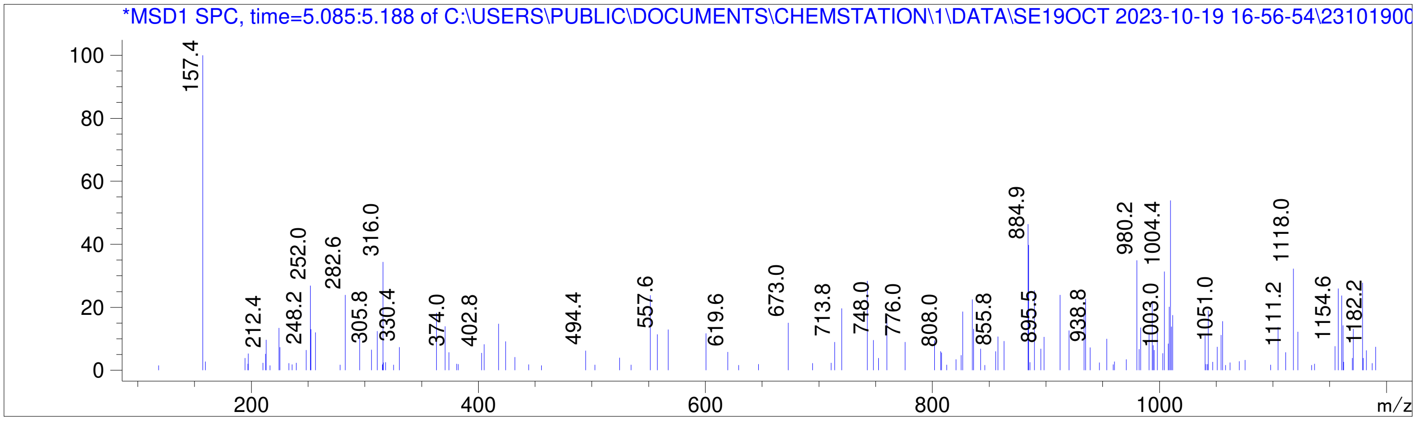

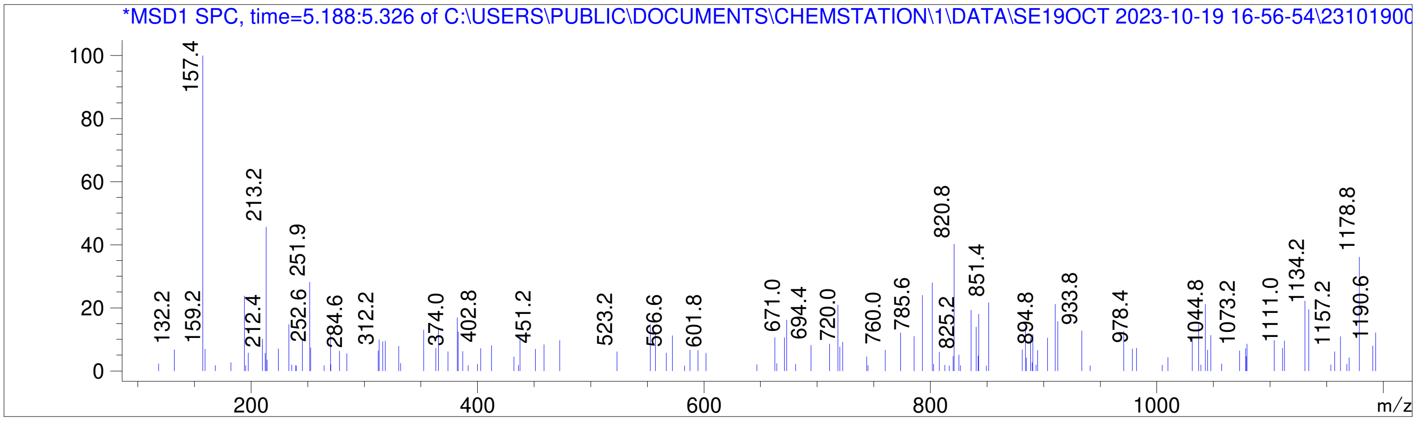
*MSD1 SPC, time=5.188:5.326 of C:\USERS\PUBLIC\DOCUMENTS\CHEMSTATION\1\DATA\SE19OCT 2023-10-19 16-56-54\23101900
100
80
60
40
20
0
157.4
213.2
251.9
132.2
159.2
212.4
252.6
284.6
312.2
374.0
402.8
451.2
523.2
566.6
601.8
671.0
694.4
720.0
760.0
785.6
820.8
825.2
851.4
894.8
933.8
978.4
1044.8
1073.2
1111.0
1134.2
1157.2
1178.8
1190.6
200
400
600
800
1000
m/z
