# Supplementary material for: Astrobiological implications of the stability and reactivity of peptide nucleic acid (PNA) in concentrated sulfuric acid
Source: Sci Adv. 2025 Mar 26;11(13):eadr0006. doi: 10.1126/sciadv.adr0006 (PMC11939054; doi:10.1126/sciadv.adr0006)

Sample-> CPT22010446-13-A2-80deg-1h

===

Injection Date : Tue, 7. Nov. 2023

Seq Line : 5
Location : 45
Inj. Vol. : 2 µl

Acq. Method : C:\Users\Public\Documents\ChemStation\1\Data\SE07NOV 2023-11-07 08-21-00\22010446 LCMS-6.M
Analysis Method : C:\Users\Public\Documents\ChemStation\1\Data\SE07NOV 2023-11-07 08-21-00\22010446 LCMS-6.M (Sequence Method)
Waters XBridge Phenyl (4.6 * 150 mm; 3.5 µm); 0.05% TFA (aq) / AcN: 100/0 (0.0 min) --> (6.0 min) --> 70/30 (0.0 min) --> (2.0 min) --> 10/90 (2.0 min); Flow: 1.0 ml/min; MSD1 = positive; MSD2 = negative

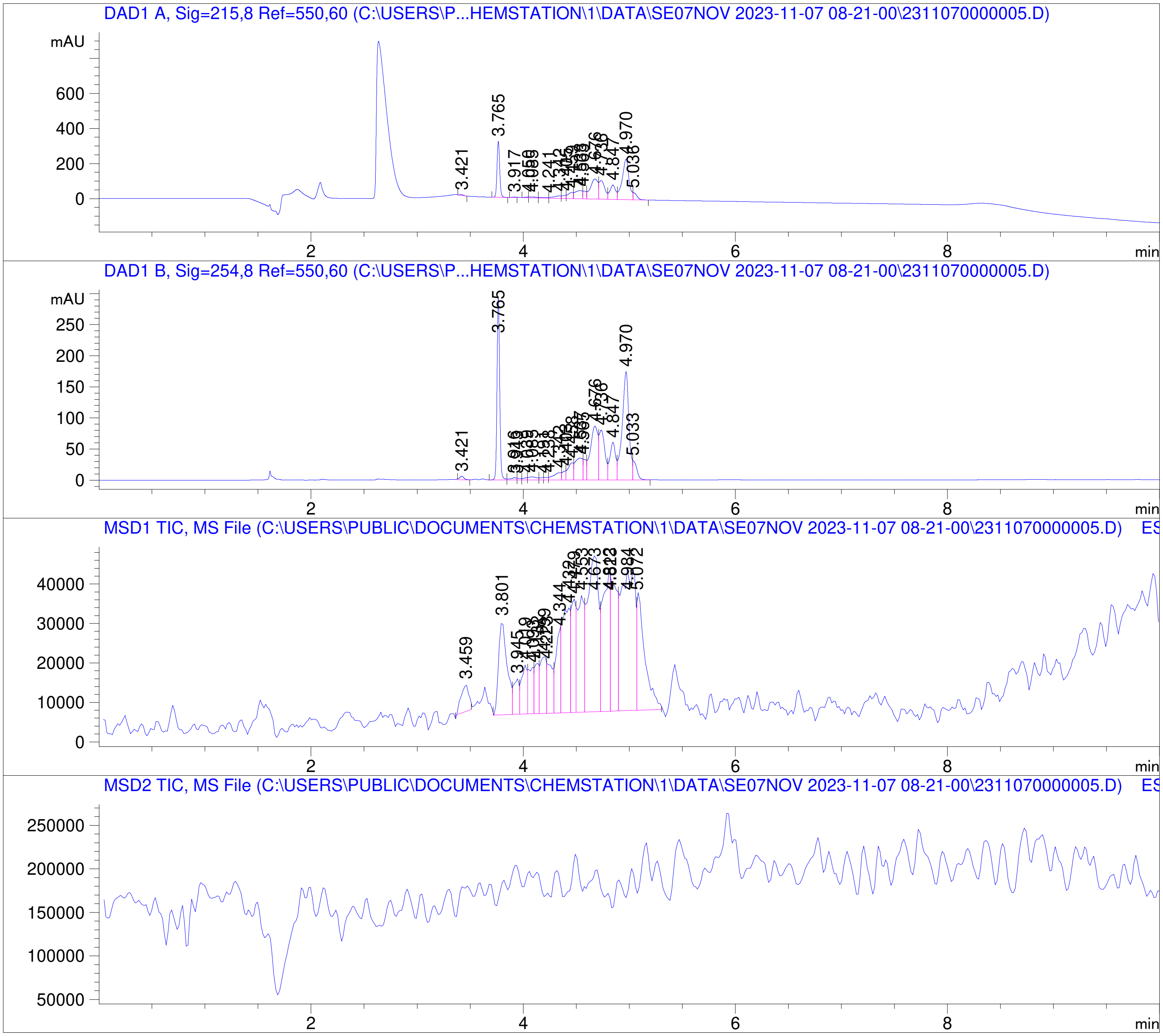

DAD1 A, Sig=215,8 Ref=550,60

Peak #	Ret. Time [min]	Area [mV *s]	Area %
1	3.421	10.923	0.302
2	3.765	555.849	15.382
3	3.917	6.628	0.183
4	4.050	10.350	0.286
5	4.085	25.285	0.700
6	4.241	21.332	0.590
7	4.342	66.913	1.852
8	4.405	45.942	1.271
9	4.459	121.329	3.357
10	4.538	224.231	6.205
11	4.563	91.845	2.542
12	4.676	570.252	15.780
13	4.736	399.846	11.065
14	4.847	316.735	8.765
15	4.970	1043.728	28.883
16	5.036	102.478	2.836

DAD1 B, Sig=254,8 Ref=550,60

Peak #	Ret. Time [min]	Area [mV *s]	Area %
1	3.421	17.602	0.616
2	3.765	510.819	17.887
3	3.916	13.167	0.461
4	3.943	6.184	0.217
5	4.039	9.068	0.318
6	4.085	26.240	0.919
7	4.191	8.787	0.308
8	4.238	10.797	0.378
9	4.342	61.204	2.143
10	4.403	32.836	1.150
11	4.458	97.784	3.424
12	4.537	173.121	6.062
13	4.565	65.178	2.282
14	4.676	426.032	14.918
15	4.736	306.232	10.723
16	4.847	232.067	8.126
17	4.970	777.895	27.239
18	5.033	80.816	2.830

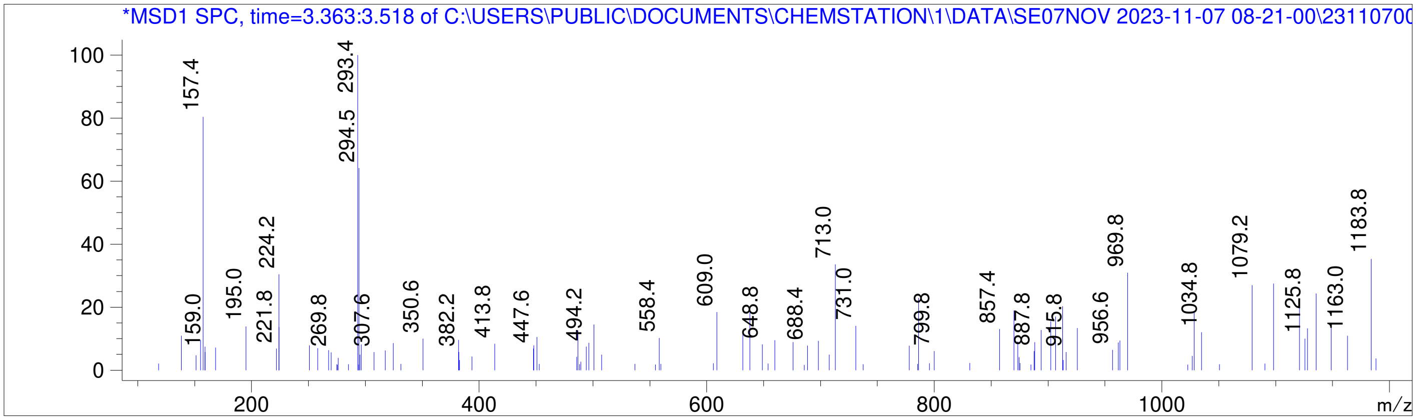
*MSD1 SPC, time=3.363:3.518 of C:\USERS\PUBLIC\DOCUMENTS\CHEMSTATION\1\DATA\SE07NOV 2023-11-07 08-21-00\2311070
157.4
293.4
294.5
224.2
195.0
159.0
221.8
269.8
307.6
350.6
382.2
413.8
447.6
494.2
558.4
609.0
648.8
688.4
713.0
731.0
799.8
857.4
887.8
915.8
956.6
969.8
1034.8
1079.2
1125.8
1163.0
1183.8
m/z

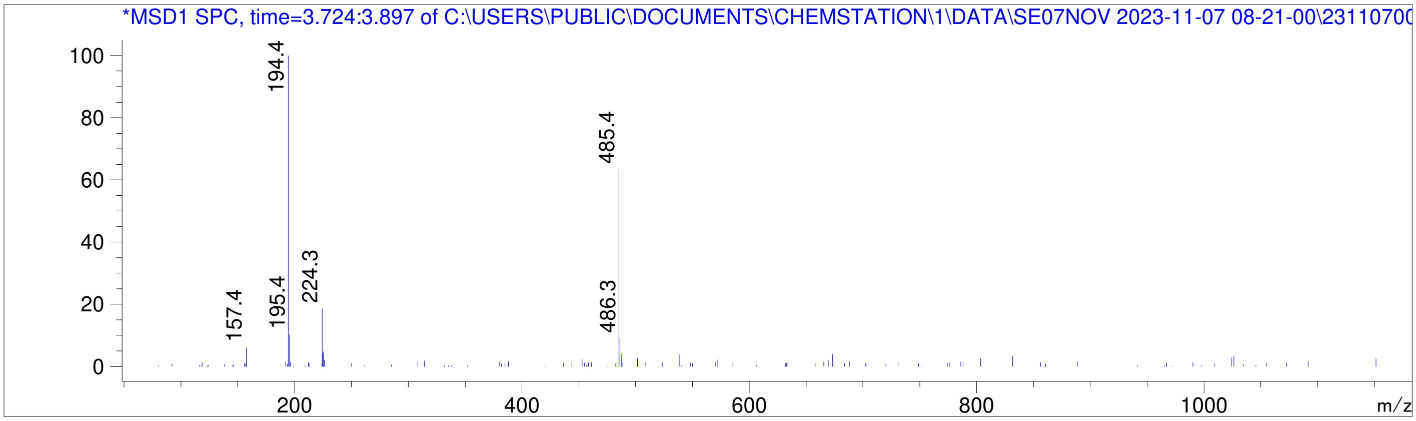
*MSD1 SPC, time=3.724:3.897 of C:\USERS\PUBLIC\DOCUMENTS\CHEMSTATION\1\DATA\SE07NOV 2023-11-07 08-21-00\2311070
194.4
485.4
157.4
195.4
224.3
486.3
m/z

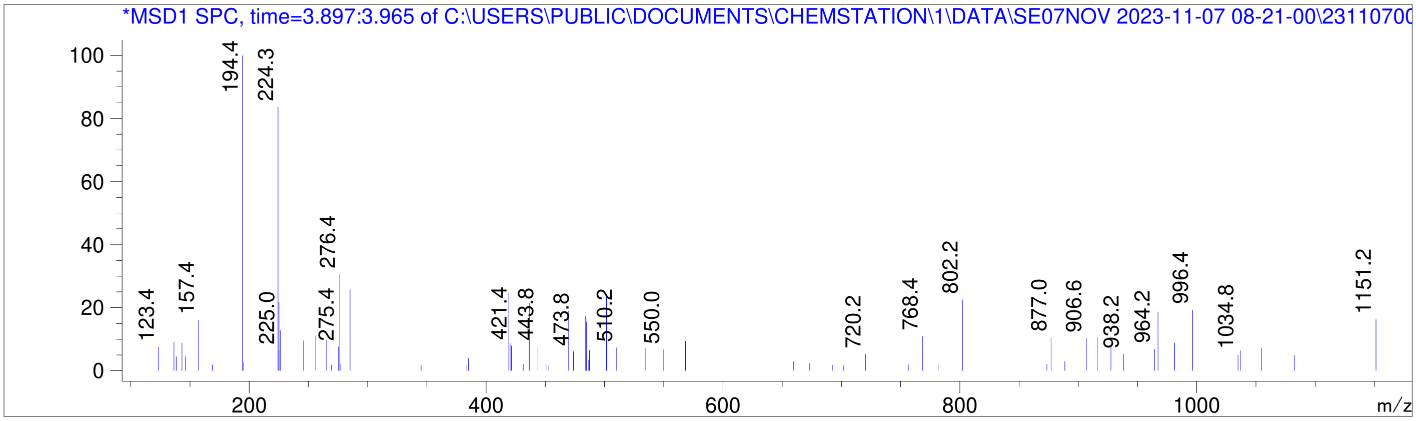
*MSD1 SPC, time=3.897:3.965 of C:\USERS\PUBLIC\DOCUMENTS\CHEMSTATION\1\DATA\SE07NOV 2023-11-07 08-21-00\2311070
194.4
224.3
276.4
123.4
157.4
225.0
275.4
421.4
443.8
473.8
510.2
550.0
720.2
768.4
802.2
877.0
906.6
938.2
964.2
996.4
1034.8
1151.2
m/z

*MSD1 SPC, time=3.965:4.052 of C:\USERS\PUBLIC\DOCUMENTS\CHEMSTATION\1\DATA\SE07NOV 2023-11-07 08-21-00\2311070

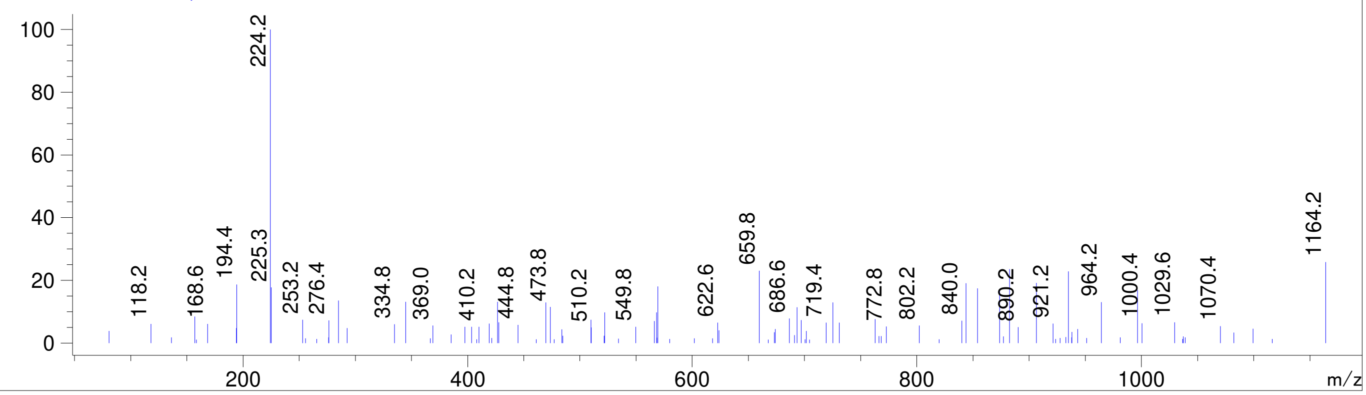

*MSD1 SPC, time=4.052:4.103 of C:\USERS\PUBLIC\DOCUMENTS\CHEMSTATION\1\DATA\SE07NOV 2023-11-07 08-21-00\2311070

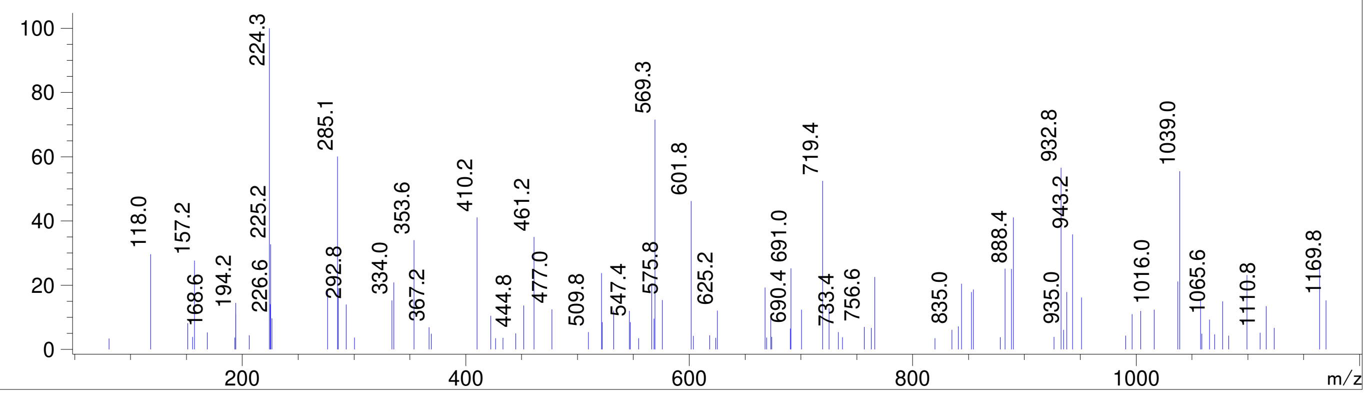

*MSD1 SPC, time=4.103:4.155 of C:\USERS\PUBLIC\DOCUMENTS\CHEMSTATION\1\DATA\SE07NOV 2023-11-07 08-21-00\2311070

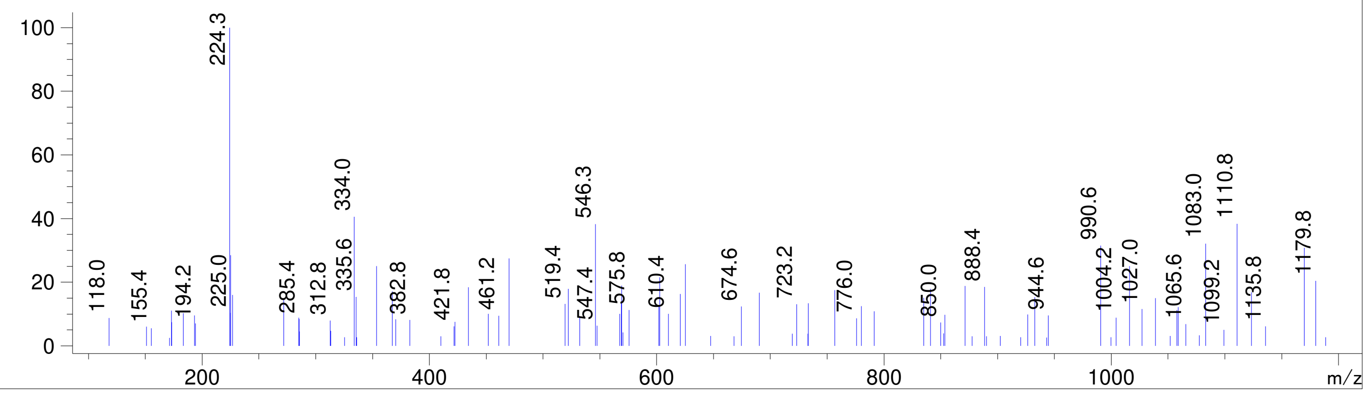

*MSD1 SPC, time=4.155:4.224 of C:\USERS\PUBLIC\DOCUMENTS\CHEMSTATION\1\DATA\SE07NOV 2023-11-07 08-21-00\2311070

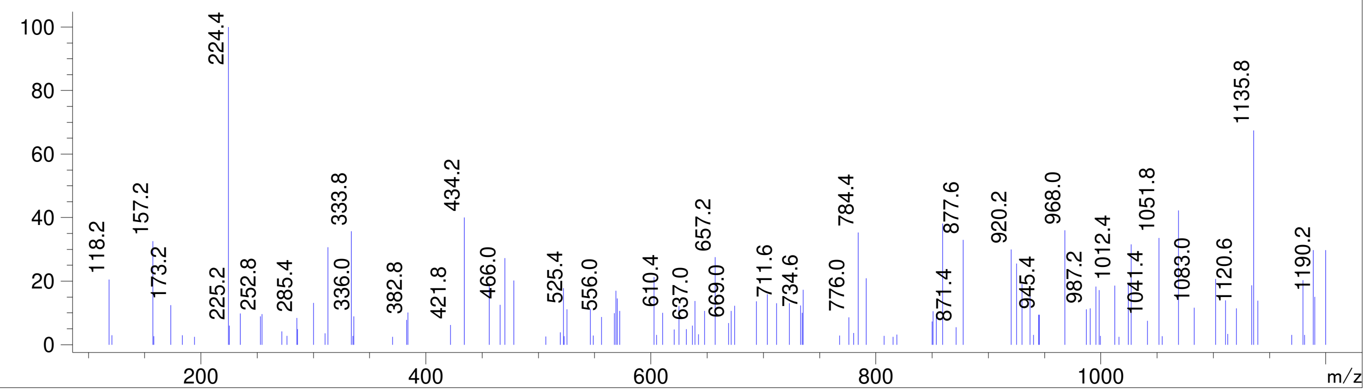

Data -> C:\USERS\PUBLIC\DOCUMENTS\CHEMSTATION\1\DATA\SE07NOV 2023-11-07 08-21-00\   ->
Sample-> CPT22010446-13-A2-80deg-1h

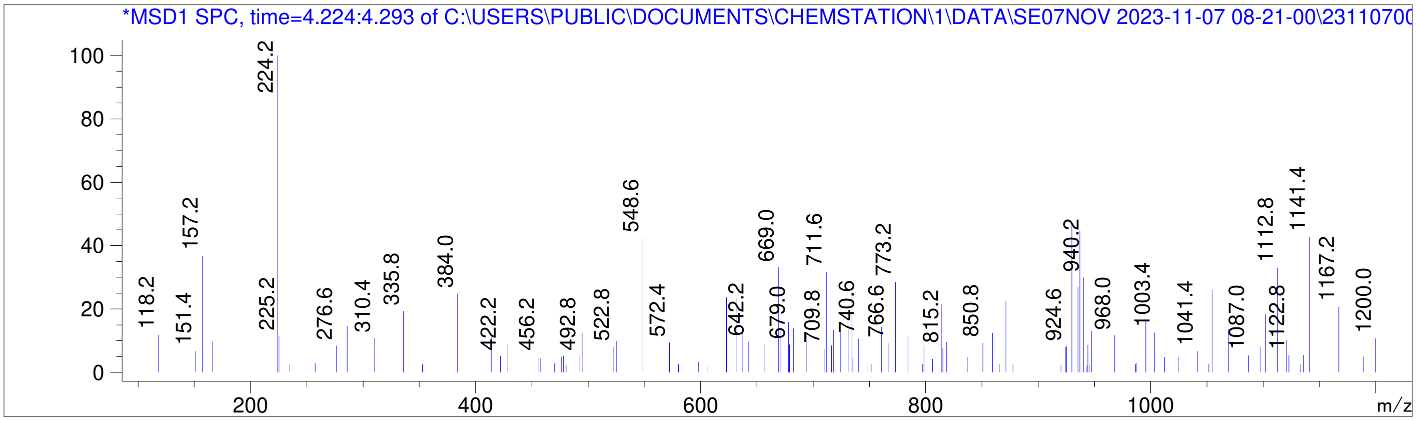

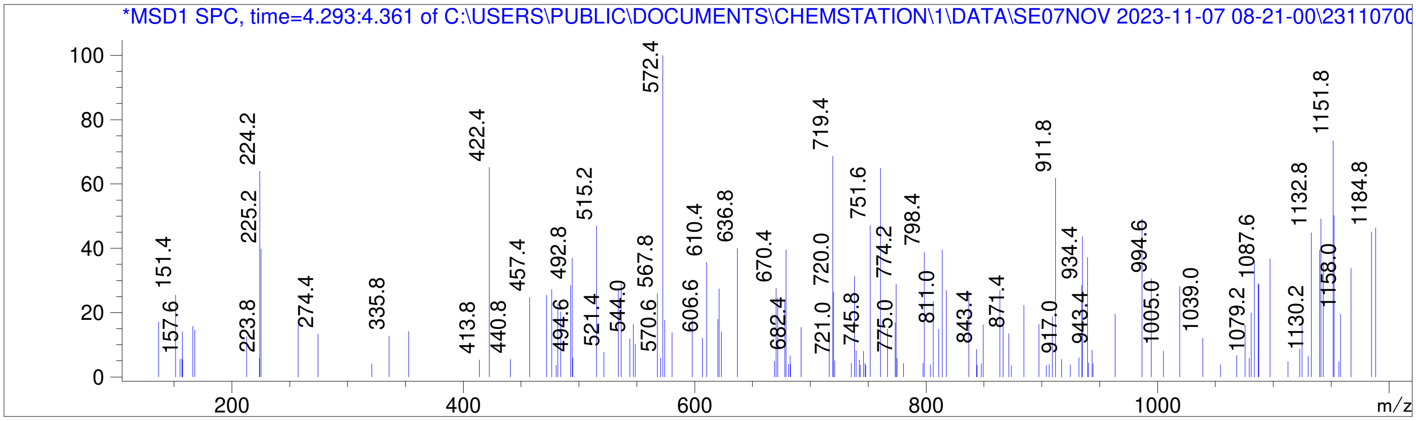

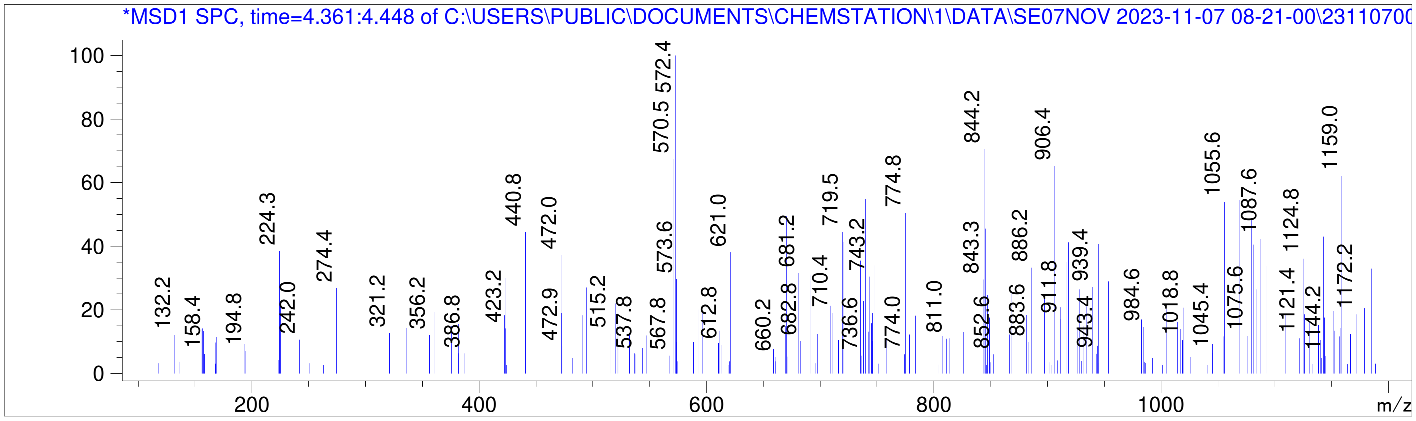

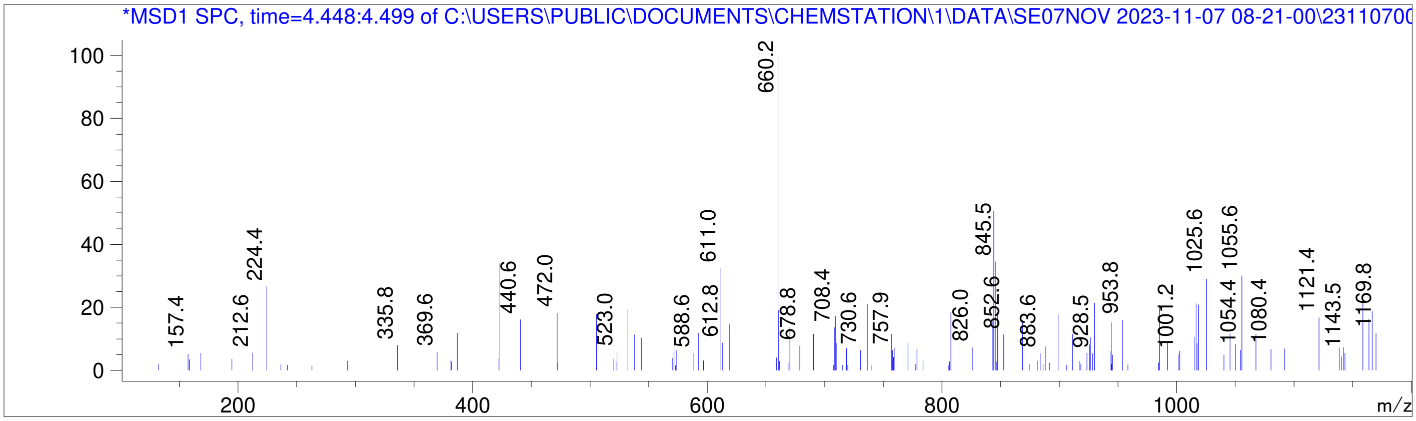
*MSD1 SPC, time=4.448:4.499 of C:\USERS\PUBLIC\DOCUMENTS\CHEMSTATION\1\DATA\SE07NOV 2023-11-07 08-21-00\2311070
660.2
845.5
224.4
611.0
1025.6
1055.6
m/z

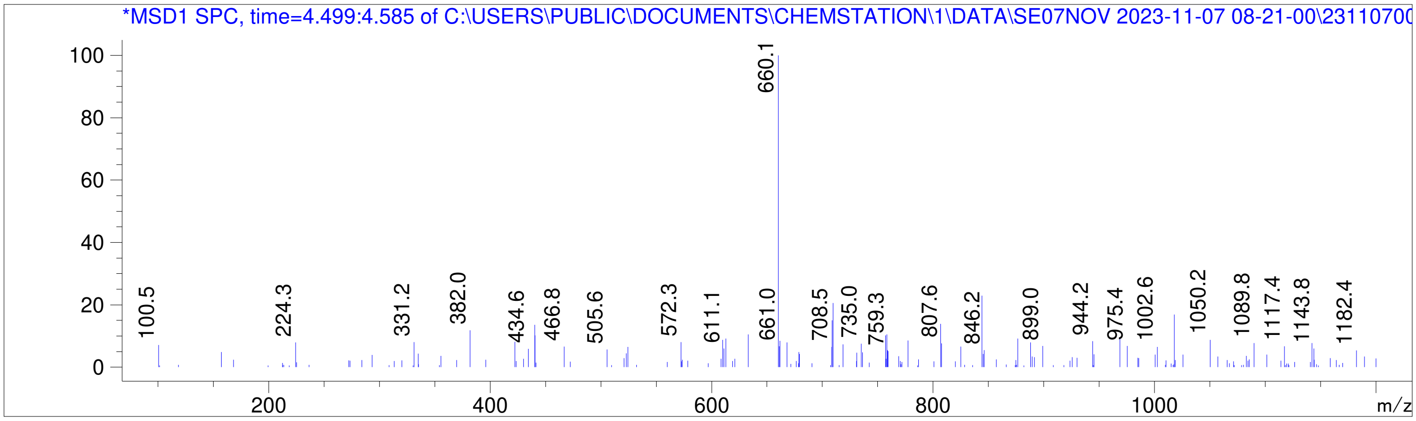
*MSD1 SPC, time=4.499:4.585 of C:\USERS\PUBLIC\DOCUMENTS\CHEMSTATION\1\DATA\SE07NOV 2023-11-07 08-21-00\2311070
660.1
m/z

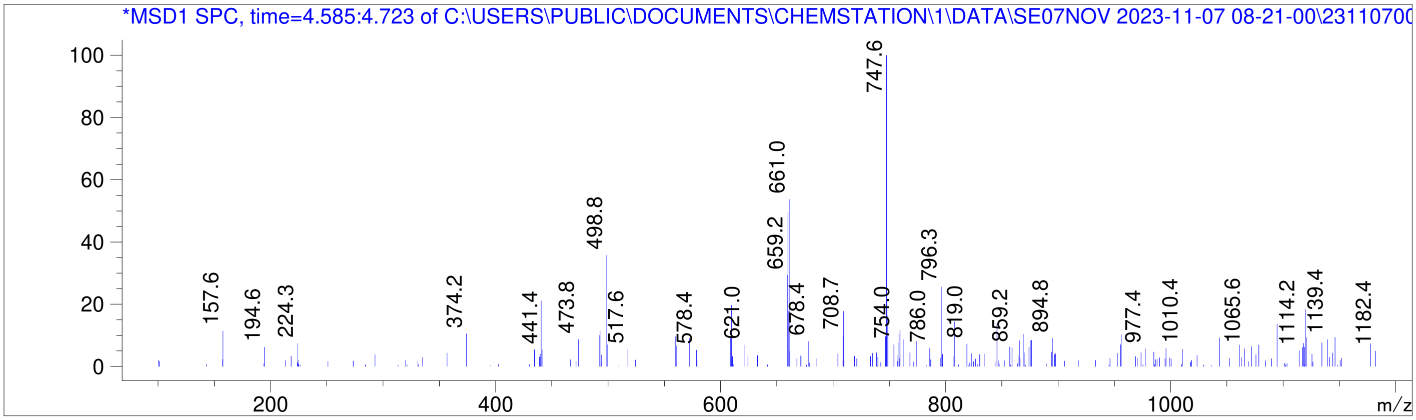
*MSD1 SPC, time=4.585:4.723 of C:\USERS\PUBLIC\DOCUMENTS\CHEMSTATION\1\DATA\SE07NOV 2023-11-07 08-21-00\2311070
747.6
661.0
659.2
498.8
796.3
m/z

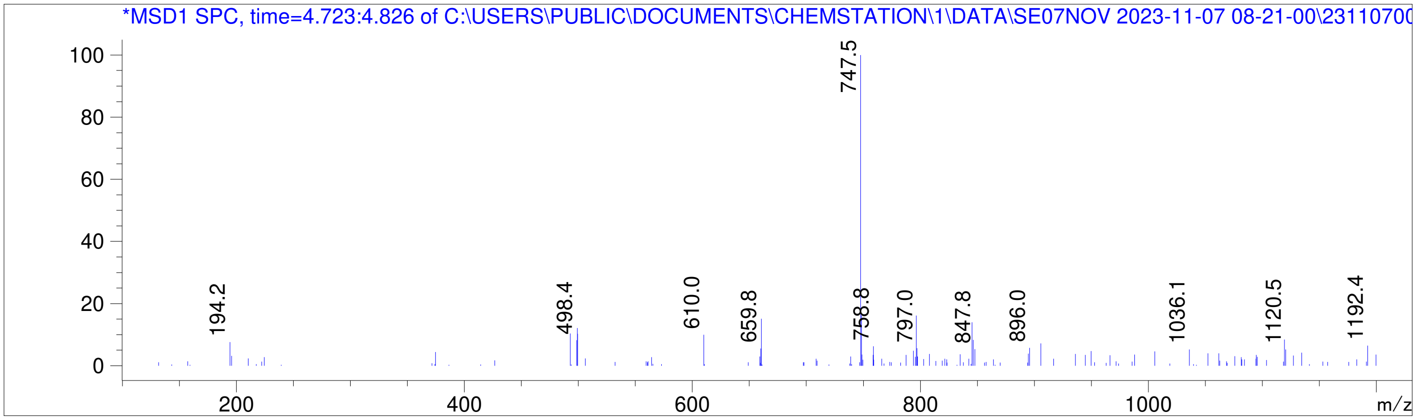
*MSD1 SPC, time=4.723:4.826 of C:\USERS\PUBLIC\DOCUMENTS\CHEMSTATION\1\DATA\SE07NOV 2023-11-07 08-21-00\2311070
747.5
m/z

Sample-> CPT22010446-13-A2-80deg-1h

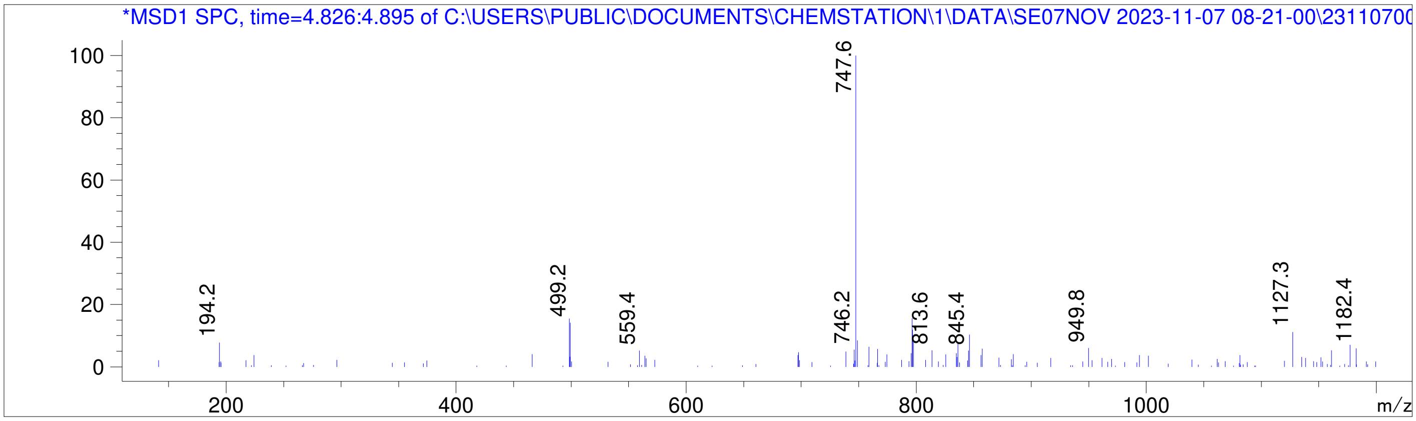

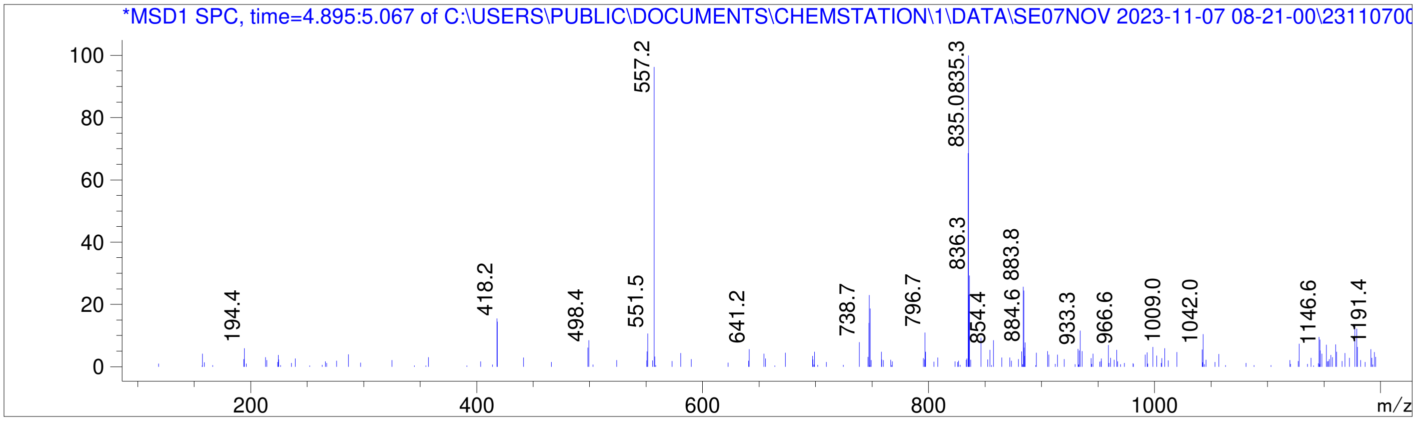

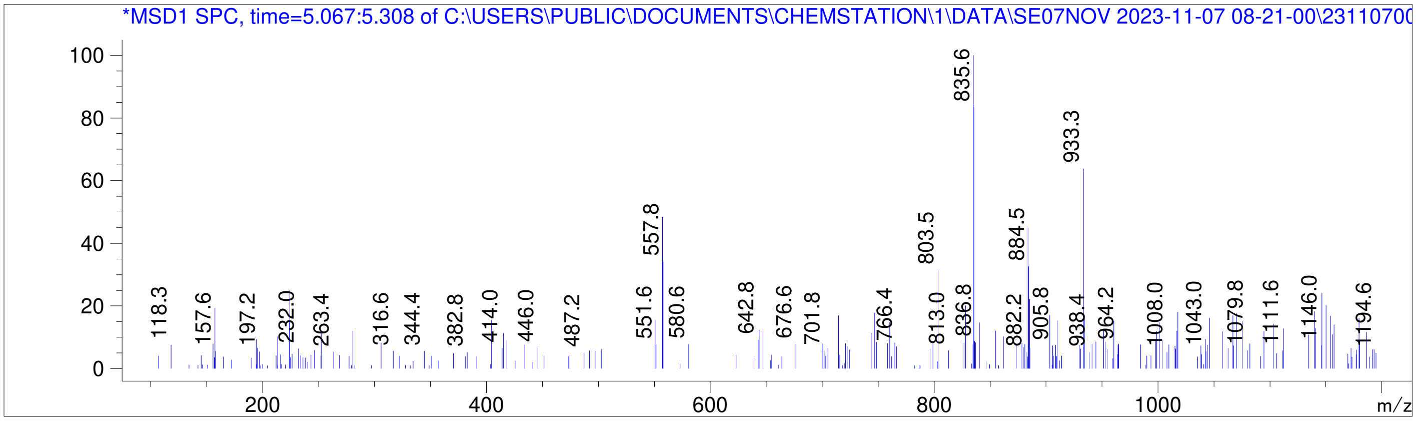

Supplement: Supplementary file 2 — Data S1 and S2 [file sciadv.adr0006_data_s1_and_s2.zip › Supplementary Dataset 1-LCMS DATA/LCMS PNA Hexamers A-T/LCMS A6 50C_80C/80C/1h/CPT22010446-13-A2-80deg-1h.pdf]
