# Supplementary material for: Astrobiological implications of the stability and reactivity of peptide nucleic acid (PNA) in concentrated sulfuric acid
Source: Sci Adv. 2025 Mar 26;11(13):eadr0006. doi: 10.1126/sciadv.adr0006 (PMC11939054; doi:10.1126/sciadv.adr0006)
Supplement: Supplementary file 2 — Data S1 and S2 [file sciadv.adr0006_data_s1_and_s2.zip › Supplementary Dataset 1-LCMS DATA/LCMS PNA Hexamers A-T/LCMS A6 50C_80C/80C/24h/CPT22010446-13-A1-80dg-24h.pdf]

Data -> C:\USERS\PUBLIC\DOCUMENTS\CHEMSTATION\1\DATA\SE19OCT 2023-10-19 16-56-54\ ->
Sample-> CPT22010446-13-A1-80dg-24h

===

Injection Date : Fri, 20. Oct. 2023		Seq Line :	41
		Location :	21
		Inj. Vol. :	2 µl

Acq. Method : C:\Users\Public\Documents\ChemStation\1\Data\SE19OCT 2023-10-19 16-56-54\22010446 LCMS-6.M
Analysis Method : C:\Users\Public\Documents\ChemStation\1\Data\SE19OCT 2023-10-19 16-56-54\22010446 LCMS-6.M (Sequence Method)
Waters XBridge Phenyl (4.6 * 150 mm; 3.5 µm); 0.05% TFA (aq) / AcN: 100/0 (0.0 min) --> (6.0 min) --> 70/30 (0.0 min) --> (2.0 min) --> 10/90 (2.0 min); Flow: 1.0 ml/min;
MSD1 = positive; MSD2 = negative

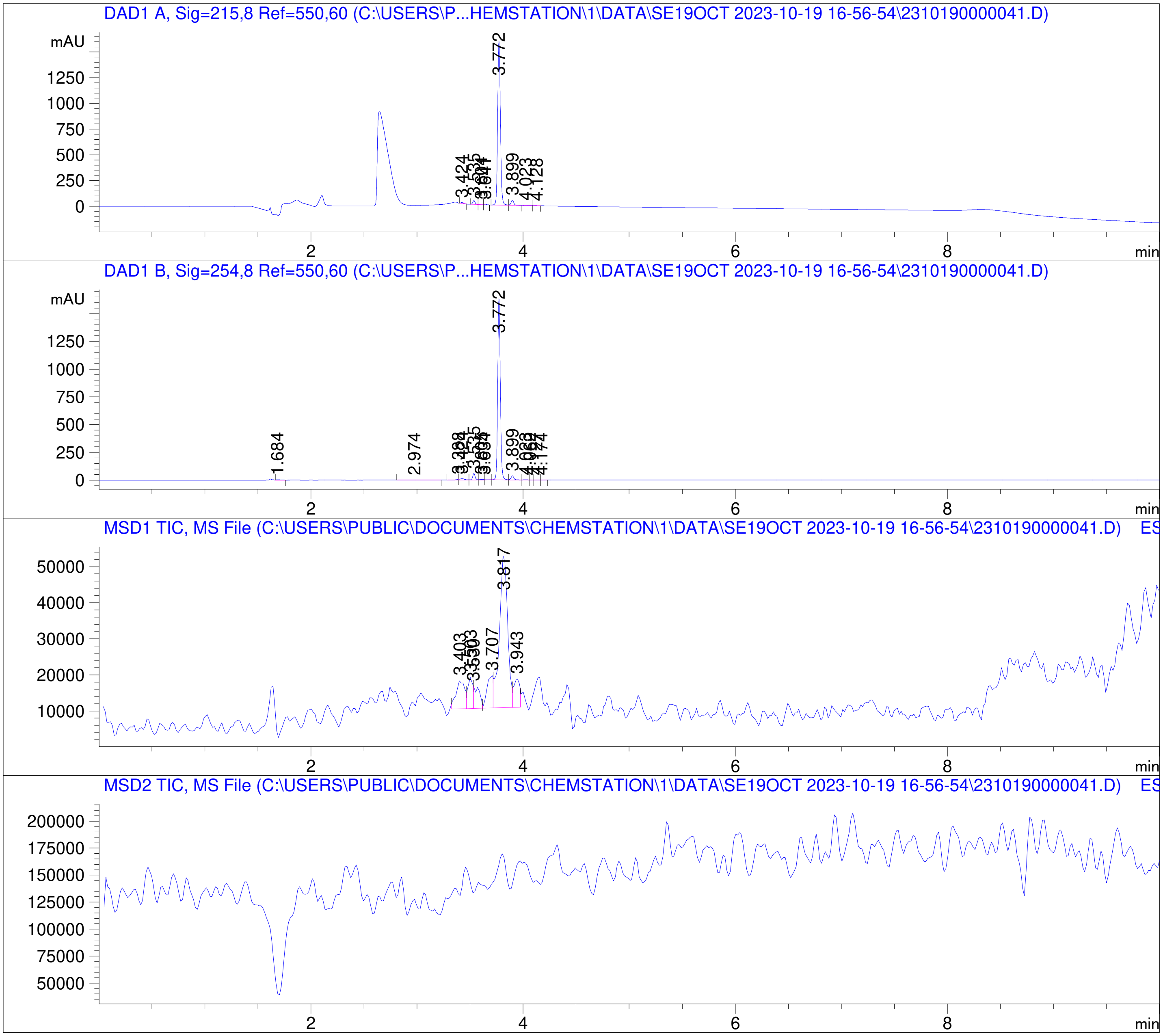

DAD1 A, Sig=215,8 Ref=550,60

Peak #	Ret. Time [min]	Area [mV *s]	Area %
1	3.424	17.599	0.575
2	3.535	55.980	1.829
3	3.604	9.119	0.298
4	3.641	7.126	0.233
5	3.772	2872.030	93.847
6	3.899	92.085	3.009
7	4.023	4.792	0.157
8	4.128	1.602	0.052

DAD1 B, Sig=254,8 Ref=550,60

Peak #	Ret. Time [min]	Area [mV *s]	Area %
1	1.684	9.483	0.308
2	2.974	16.145	0.525
3	3.388	8.950	0.291
4	3.424	40.518	1.317
5	3.535	95.417	3.102
6	3.605	11.512	0.374
7	3.634	4.097	0.133
8	3.772	2811.608	91.403
9	3.899	68.359	2.222
10	4.023	5.951	0.193
11	4.062	0.654	0.021
12	4.127	2.831	0.092
13	4.174	0.536	0.017

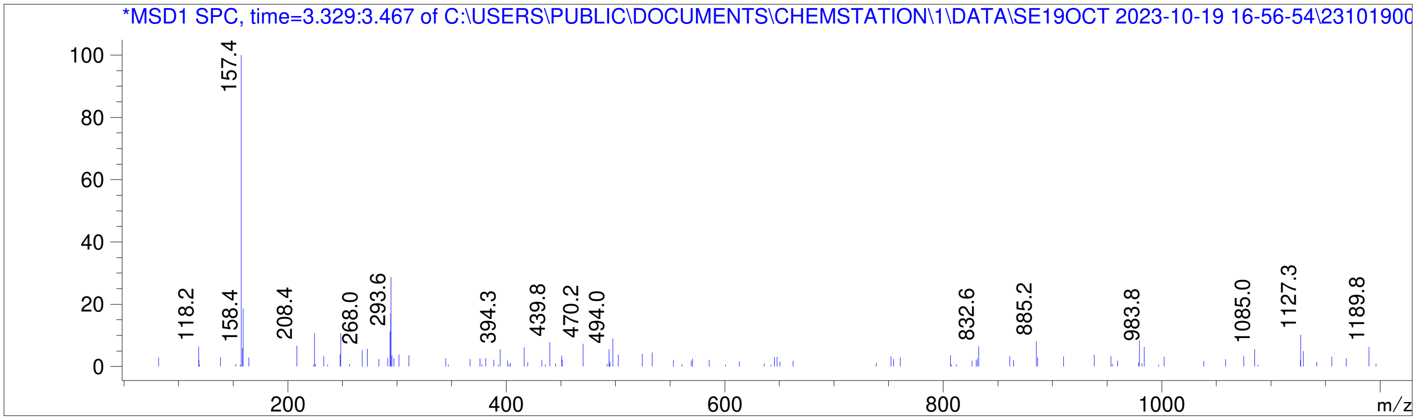
*MSD1 SPC, time=3.329:3.467 of C:\USERS\PUBLIC\DOCUMENTS\CHEMSTATION\1\DATA\SE19OCT 2023-10-19 16-56-54\23101900
157.4
118.2
158.4
208.4
268.0
293.6
394.3
439.8
470.2
494.0
832.6
885.2
983.8
1085.0
1127.3
1189.8
m/z

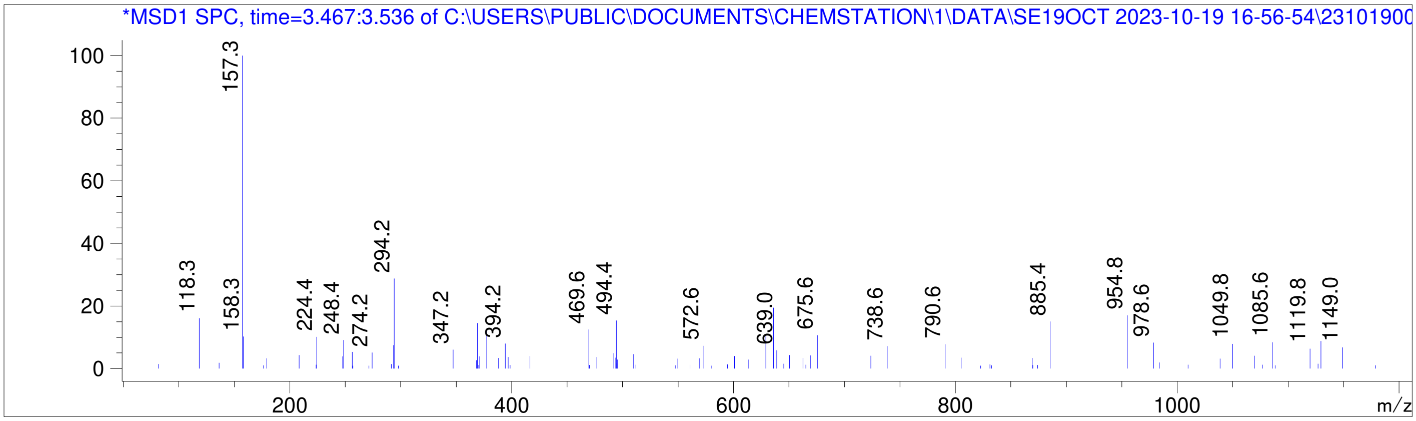
*MSD1 SPC, time=3.467:3.536 of C:\USERS\PUBLIC\DOCUMENTS\CHEMSTATION\1\DATA\SE19OCT 2023-10-19 16-56-54\23101900
157.3
118.3
158.3
224.4
248.4
274.2
294.2
347.2
394.2
469.6
494.4
572.6
639.0
675.6
738.6
790.6
885.4
954.8
978.6
1049.8
1085.6
1119.8
1149.0
m/z

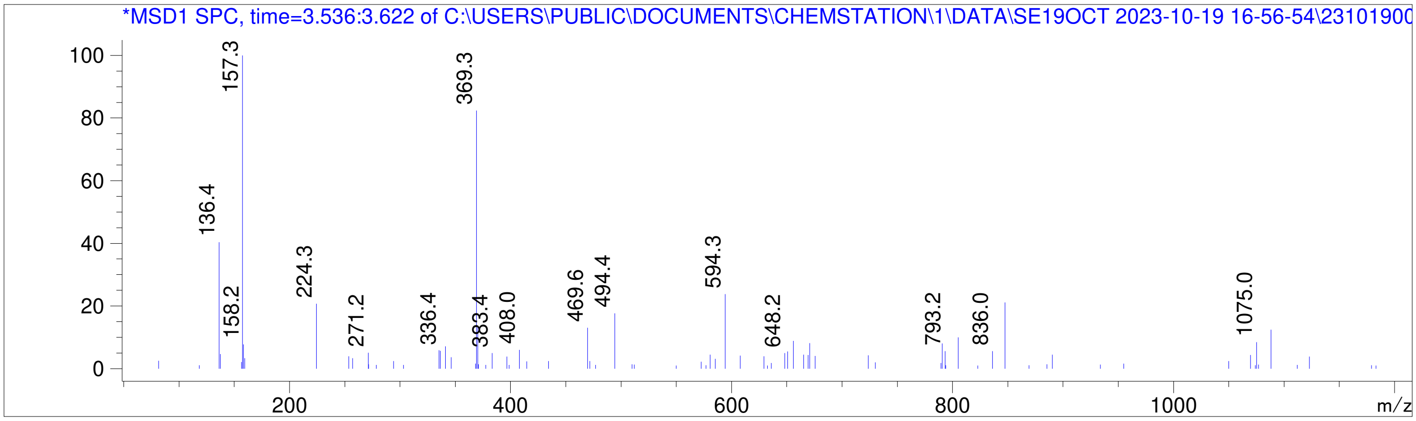
*MSD1 SPC, time=3.536:3.622 of C:\USERS\PUBLIC\DOCUMENTS\CHEMSTATION\1\DATA\SE19OCT 2023-10-19 16-56-54\23101900
157.3
136.4
158.2
224.3
271.2
336.4
369.3
383.4
408.0
469.6
494.4
594.3
648.2
793.2
836.0
1075.0
m/z

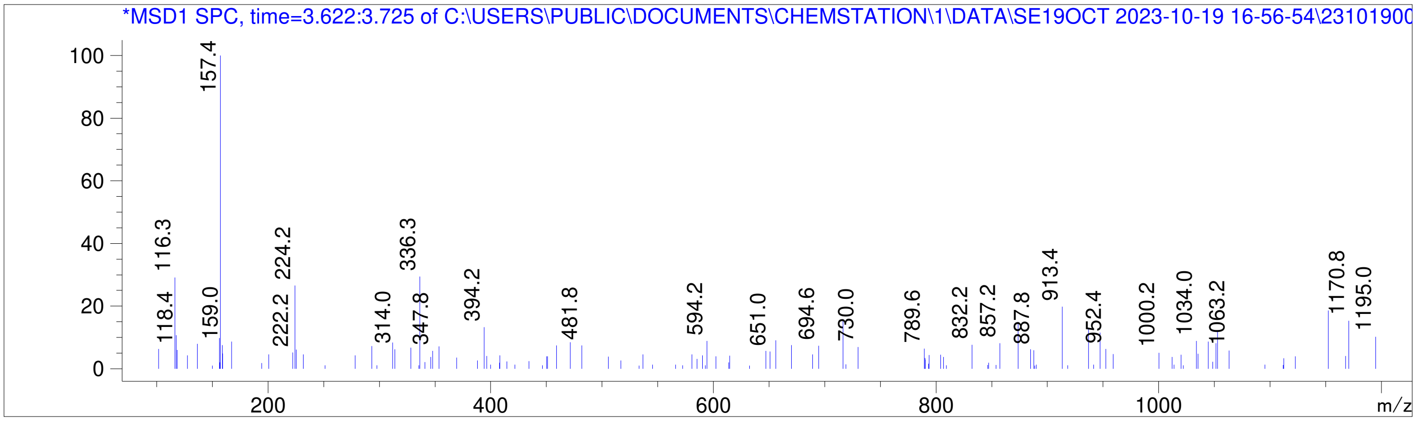
*MSD1 SPC, time=3.622:3.725 of C:\USERS\PUBLIC\DOCUMENTS\CHEMSTATION\1\DATA\SE19OCT 2023-10-19 16-56-54\23101900
157.4
116.3
118.4
159.0
224.2
222.2
314.0
336.3
347.8
394.2
481.8
594.2
651.0
694.6
730.0
789.6
832.2
857.2
887.8
913.4
952.4
1000.2
1034.0
1063.2
1170.8
1195.0
m/z

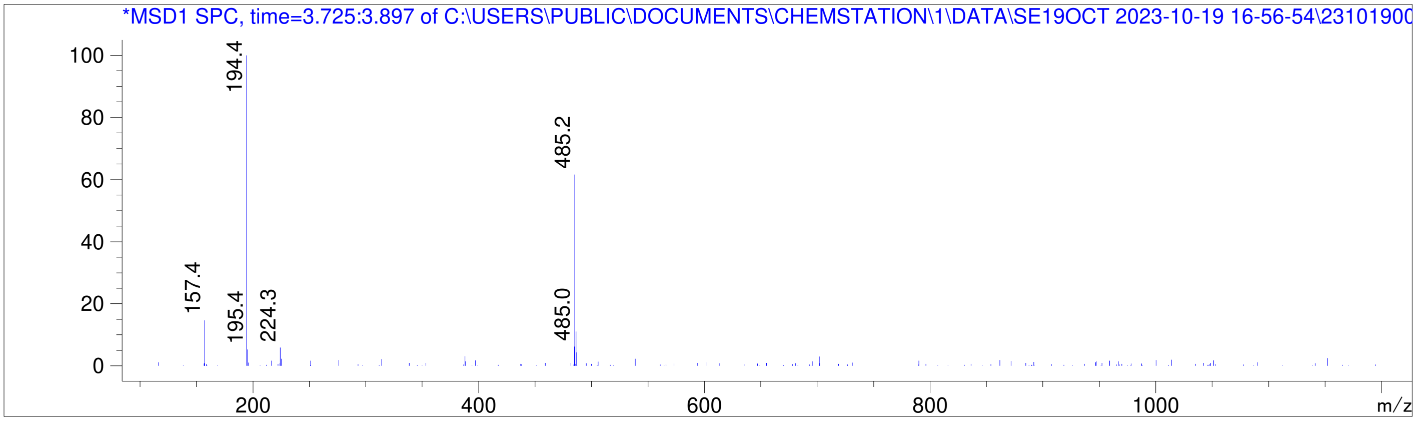
*MSD1 SPC, time=3.725:3.897 of C:\USERS\PUBLIC\DOCUMENTS\CHEMSTATION\1\DATA\SE19OCT 2023-10-19 16-56-54\23101900
194.4
485.2
157.4
195.4
224.3
485.0
m/z

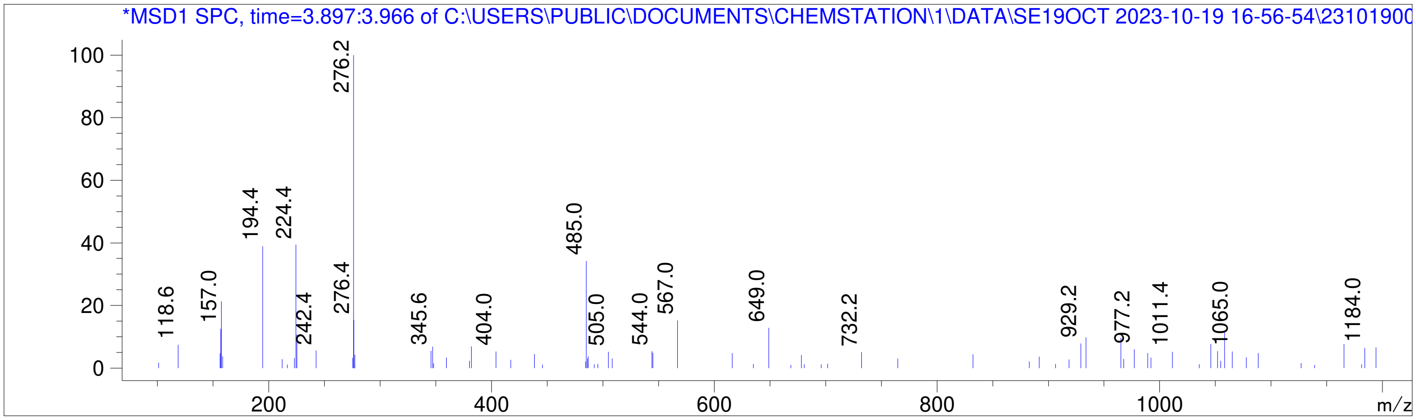
*MSD1 SPC, time=3.897:3.966 of C:\USERS\PUBLIC\DOCUMENTS\CHEMSTATION\1\DATA\SE19OCT 2023-10-19 16-56-54\23101900
276.2
194.4
224.4
485.0
118.6
157.0
242.4
276.4
345.6
404.0
505.0
544.0
567.0
649.0
732.2
929.2
977.2
1011.4
1065.0
1184.0
m/z
