# Supplementary material for: Astrobiological implications of the stability and reactivity of peptide nucleic acid (PNA) in concentrated sulfuric acid
Source: Sci Adv. 2025 Mar 26;11(13):eadr0006. doi: 10.1126/sciadv.adr0006 (PMC11939054; doi:10.1126/sciadv.adr0006)
Supplement: Supplementary file 2 — Data S1 and S2 [file sciadv.adr0006_data_s1_and_s2.zip › Supplementary Dataset 1-LCMS DATA/LCMS PNA Hexamers A-T/LCMS A6 50C_80C/80C/24h/CPT22010446-13-A2-80deg-24h.pdf]

Data -> C:\Users\Public\Documents\ChemStation\1\Data\SE07NOV 2023-11-07 14-56-21\   ->
Sample-> CPT22010446-13-A2-80deg-24h

===

Injection Date   : Wed, 8. Nov. 2023   Seq Line   :   36
                                                           Location   :   73
                                                           Inj. Vol. :   2 µl

Acq. Method   : C:\Users\Public\Documents\ChemStation\1\Data\SE07NOV 2023-11-07 14-56-21\22010446 LCMS-6.M
Analysis Method : C:\Users\Public\Documents\ChemStation\1\Data\SE07NOV 2023-11-07 14-56-21\22010446 LCMS-6.M (Sequence Method)
Waters XBridge Phenyl (4.6 * 150 mm; 3.5 µm); 0.05% TFA (aq) / AcN: 100/0 (0.0 min) --> (6.0 min) --> 70/30 (0.0 min) --> (2.0 min) --> 10/90 (2.0 min); Flow: 1.0 ml/min;
MSD1 = positive; MSD2 = negative

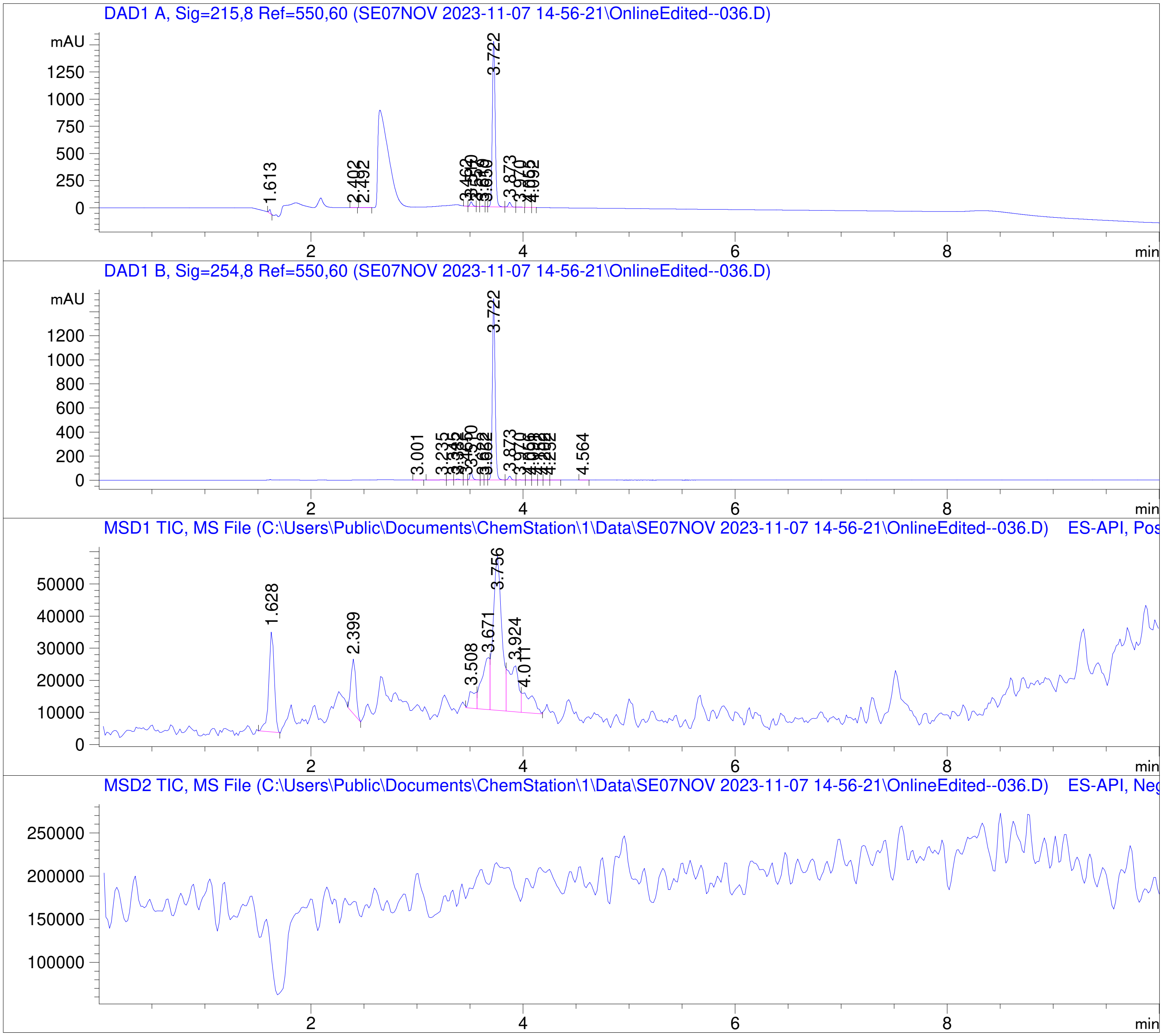

DAD1 A, Sig=215,8 Ref=550,60

Peak #	Ret. Time [min]	Area [mV *s]	Area %
1	1.613	38.548	1.261
2	2.402	0.384	0.013
3	2.492	1.381	0.045
4	3.462	0.989	0.032
5	3.510	66.694	2.181
6	3.557	1.071	0.035
7	3.619	8.045	0.263
8	3.650	2.118	0.069
9	3.722	2856.494	93.407
10	3.873	75.659	2.474
11	3.970	4.542	0.149
12	4.055	1.434	0.047
13	4.092	0.742	0.024

DAD1 B, Sig=254,8 Ref=550,60

Peak #	Ret. Time [min]	Area [mV *s]	Area %
1	3.001	0.339	0.011
2	3.235	10.642	0.356
3	3.345	8.928	0.299
4	3.382	23.723	0.794
5	3.465	6.797	0.227
6	3.510	115.629	3.868
7	3.622	4.729	0.158
8	3.652	6.231	0.208
9	3.722	2738.326	91.611
10	3.873	61.301	2.051
11	3.970	6.789	0.227
12	4.056	2.140	0.072
13	4.091	1.335	0.045
14	4.152	0.829	0.028
15	4.202	0.696	0.023
16	4.252	0.428	0.014
17	4.564	0.219	0.007

Sample-> CPT22010446-13-A2-80deg-24h

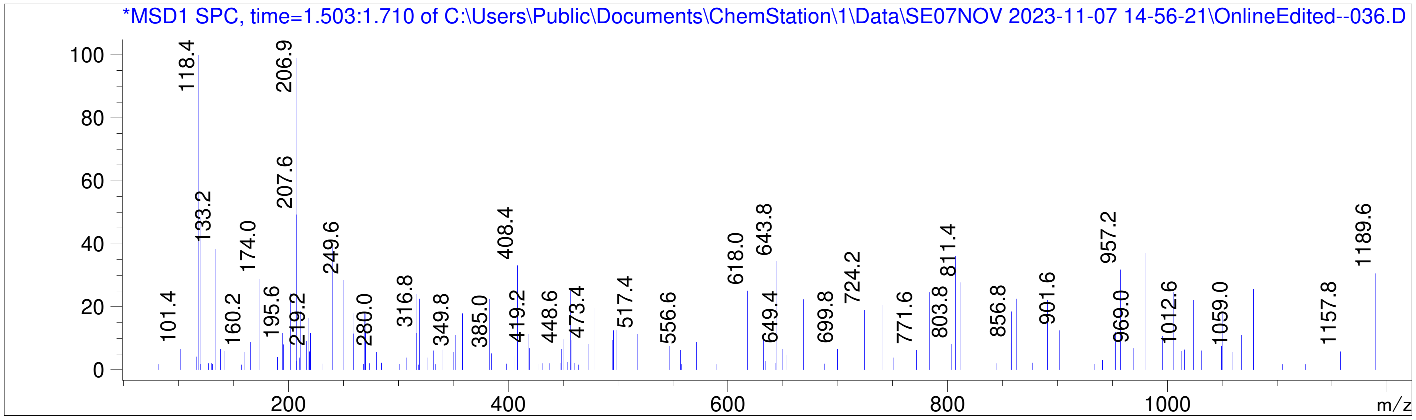

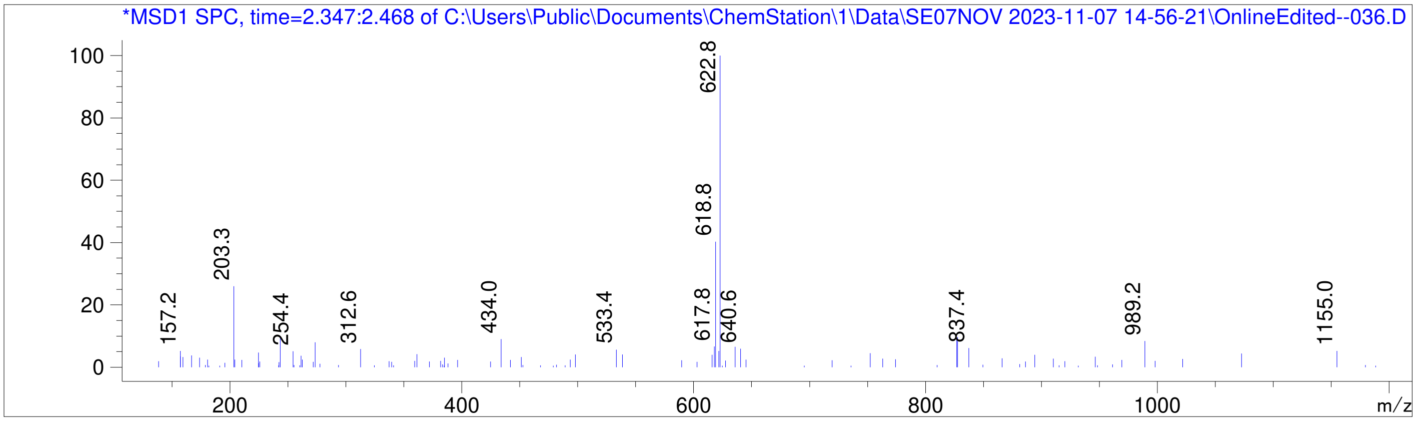

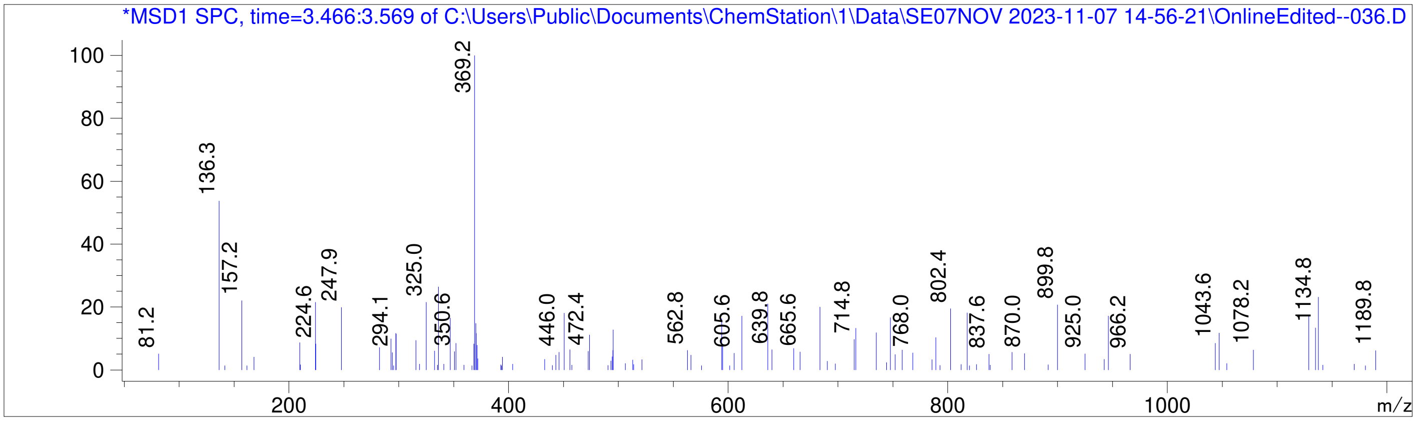

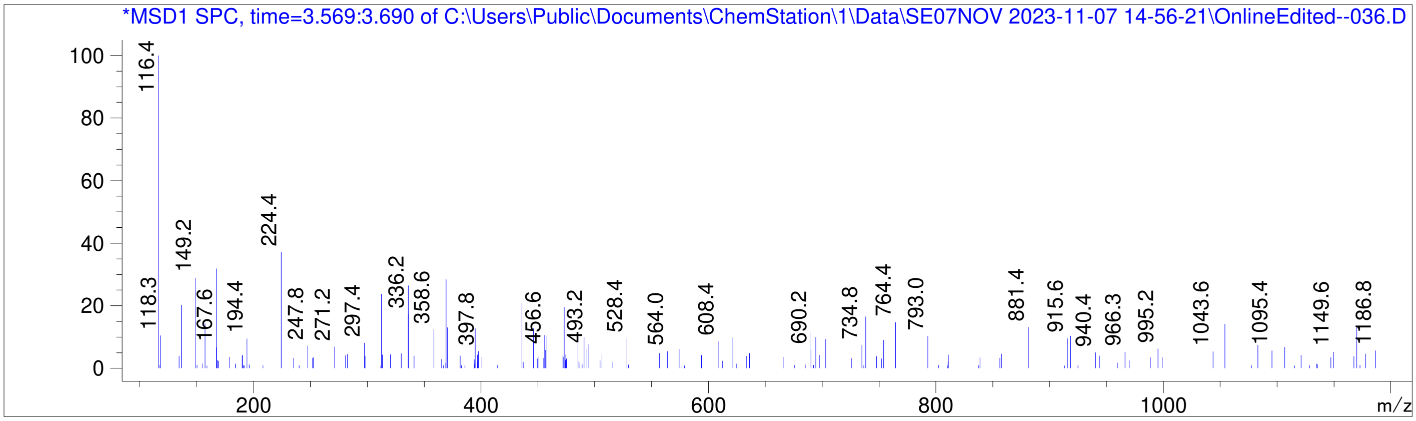
*MSD1 SPC, time=3.569:3.690 of C:\Users\Public\Documents\ChemStation\1\Data\SE07NOV 2023-11-07 14-56-21\OnlineEdited--036.D
116.4
118.3
149.2
167.6
194.4
224.4
247.8
271.2
297.4
336.2
358.6
397.8
456.6
493.2
528.4
564.0
608.4
690.2
734.8
764.4
793.0
881.4
915.6
940.4
966.3
995.2
1043.6
1095.4
1149.6
1186.8
0
20
40
60
80
100
200
400
600
800
1000
m/z

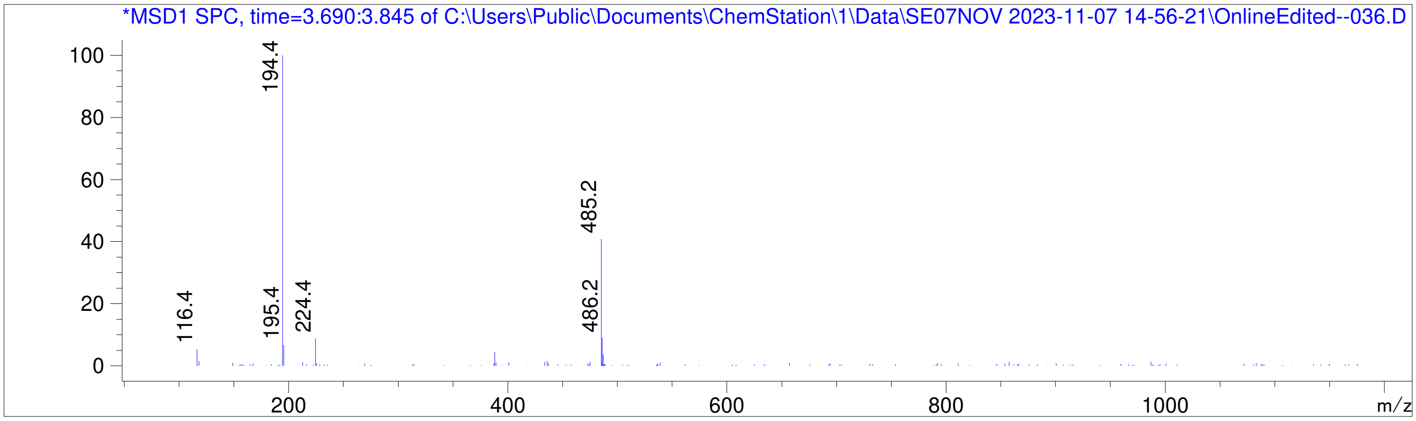
*MSD1 SPC, time=3.690:3.845 of C:\Users\Public\Documents\ChemStation\1\Data\SE07NOV 2023-11-07 14-56-21\OnlineEdited--036.D
194.4
485.2
116.4
195.4
224.4
486.2
0
20
40
60
80
100
200
400
600
800
1000
m/z

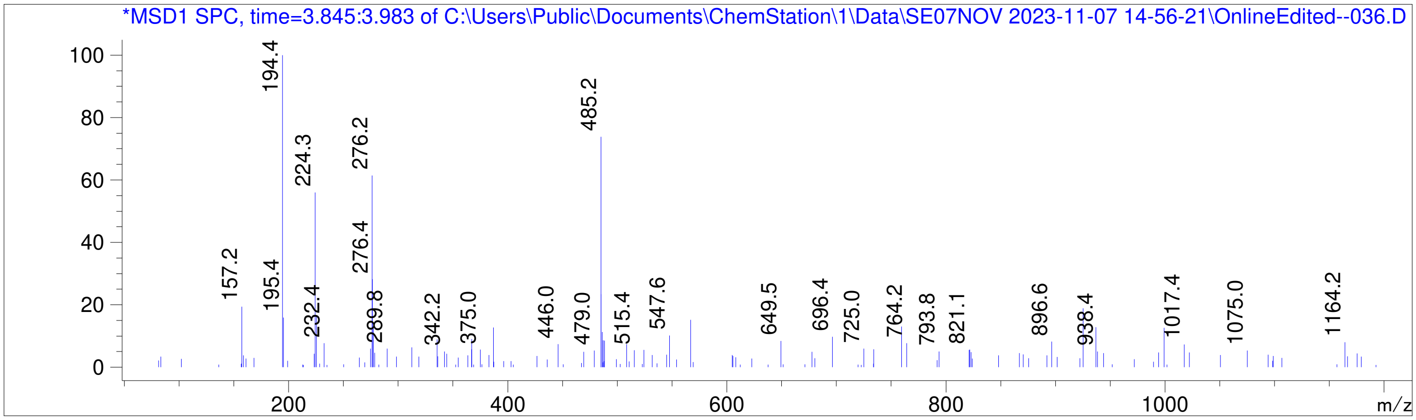
*MSD1 SPC, time=3.845:3.983 of C:\Users\Public\Documents\ChemStation\1\Data\SE07NOV 2023-11-07 14-56-21\OnlineEdited--036.D
194.4
485.2
224.3
276.2
276.4
157.2
195.4
232.4
289.8
342.2
375.0
446.0
479.0
515.4
547.6
649.5
696.4
725.0
764.2
793.8
821.1
896.6
938.4
1017.4
1075.0
1164.2
0
20
40
60
80
100
200
400
600
800
1000
m/z

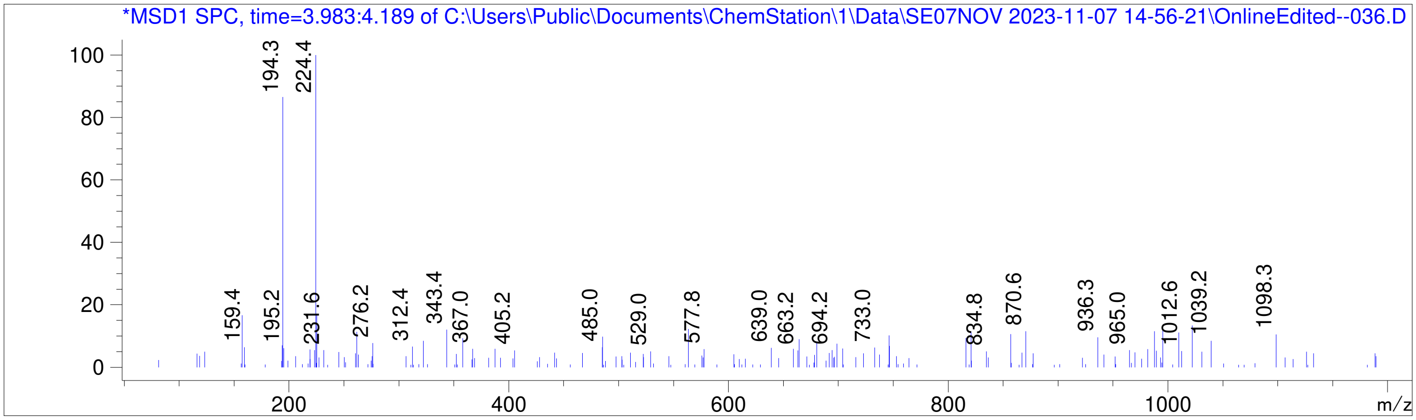
*MSD1 SPC, time=3.983:4.189 of C:\Users\Public\Documents\ChemStation\1\Data\SE07NOV 2023-11-07 14-56-21\OnlineEdited--036.D
194.3
224.4
159.4
195.2
231.6
276.2
312.4
343.4
367.0
405.2
485.0
529.0
577.8
639.0
663.2
694.2
733.0
834.8
870.6
936.3
965.0
1012.6
1039.2
1098.3
0
20
40
60
80
100
200
400
600
800
1000
m/z
