# Supplementary material for: Astrobiological implications of the stability and reactivity of peptide nucleic acid (PNA) in concentrated sulfuric acid
Source: Sci Adv. 2025 Mar 26;11(13):eadr0006. doi: 10.1126/sciadv.adr0006 (PMC11939054; doi:10.1126/sciadv.adr0006)

Data -> C:\Users\Public\Documents\ChemStation\1\Data\SE09OCT 2023-10-09 14-54-12\ ->
Sample-> CPT22010446-13-A-1-14d

===

Injection Date : Mon, 9. Oct. 2023

Seq Line : 13
Location : 82
Inj. Vol. : 2 µl

Acq. Method : C:\Users\Public\Documents\ChemStation\1\Data\SE09OCT 2023-10-09 14-54-12\22010446 LCMS-6.M
Analysis Method : C:\Users\Public\Documents\ChemStation\1\Data\SE09OCT 2023-10-09 14-54-12\22010446 LCMS-6.M (Sequence Method)
Waters XBridge Phenyl (4.6 * 150 mm; 3.5 µm); 0.05% TFA (aq) / AcN: 100/0 (0.0 min) --> (6.0 min) --> 70/30 (0.0 min) --> (2.0 min) --> 10/90 (2.0 min); Flow: 1.0 ml/min;
MSD1 = positive; MSD2 = negative

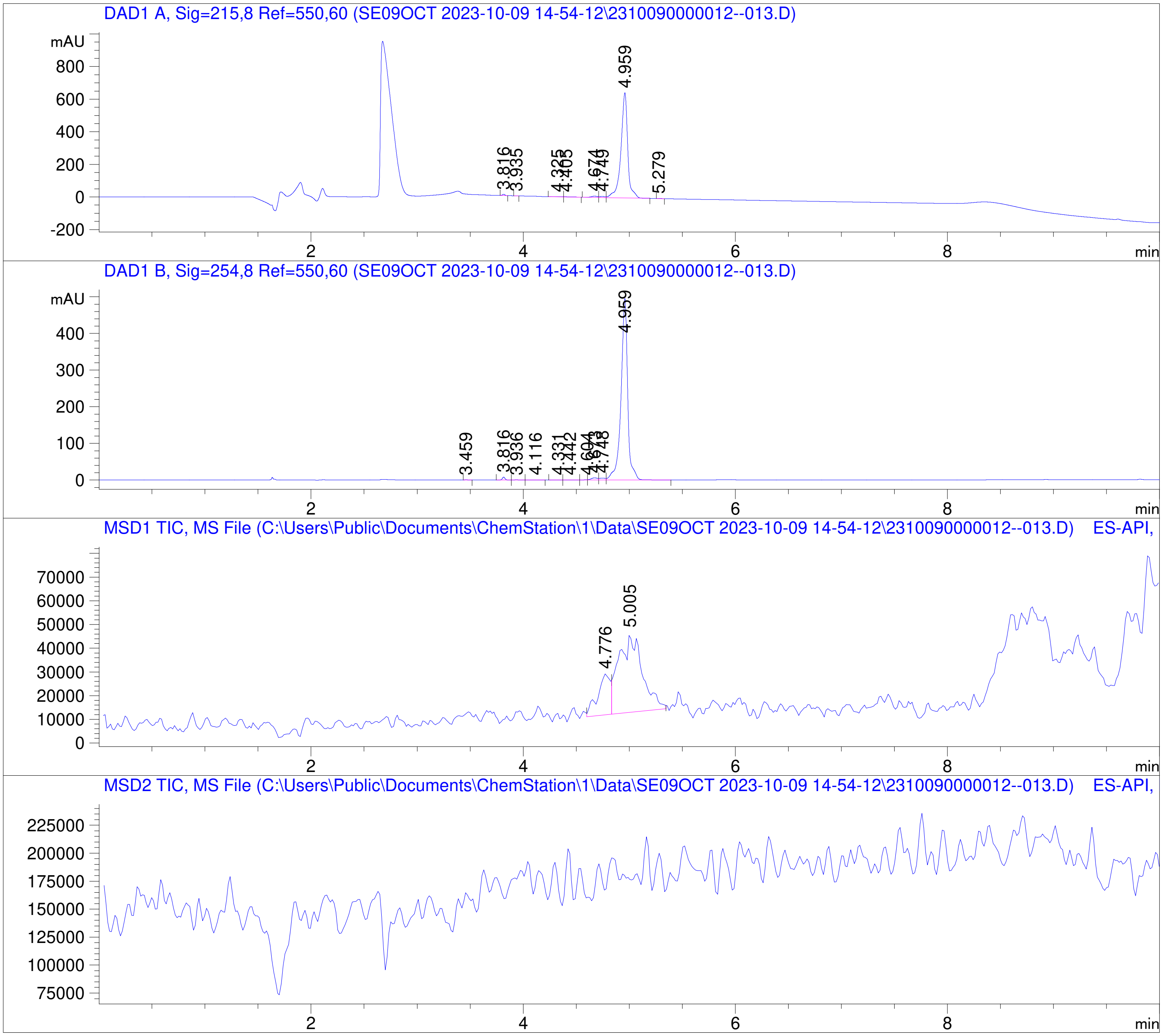

DAD1 A, Sig=215,8 Ref=550,60

Peak #	Ret. Time [min]	Area [mV *s]	Area %
1	3.816	12.812	0.433
2	3.935	0.695	0.023
3	4.325	6.329	0.214
4	4.405	6.084	0.206
5	4.674	32.025	1.083
6	4.749	25.806	0.872
7	4.959	2873.438	97.150
8	5.279	0.542	0.018

DAD1 B, Sig=254,8 Ref=550,60

Peak #	Ret. Time [min]	Area [mV *s]	Area %
1	3.459	1.448	0.065
2	3.816	13.396	0.598
3	3.936	1.608	0.072
4	4.116	1.301	0.058
5	4.331	0.869	0.039
6	4.442	1.924	0.086
7	4.604	1.397	0.062
8	4.673	24.645	1.099
9	4.748	19.669	0.877
10	4.959	2175.559	97.044

Data -> C:\Users\Public\Documents\ChemStation\1\Data\SE09OCT 2023-10-09 14-54-12\   ->
Sample-> CPT22010446-13-A-1-14d
==

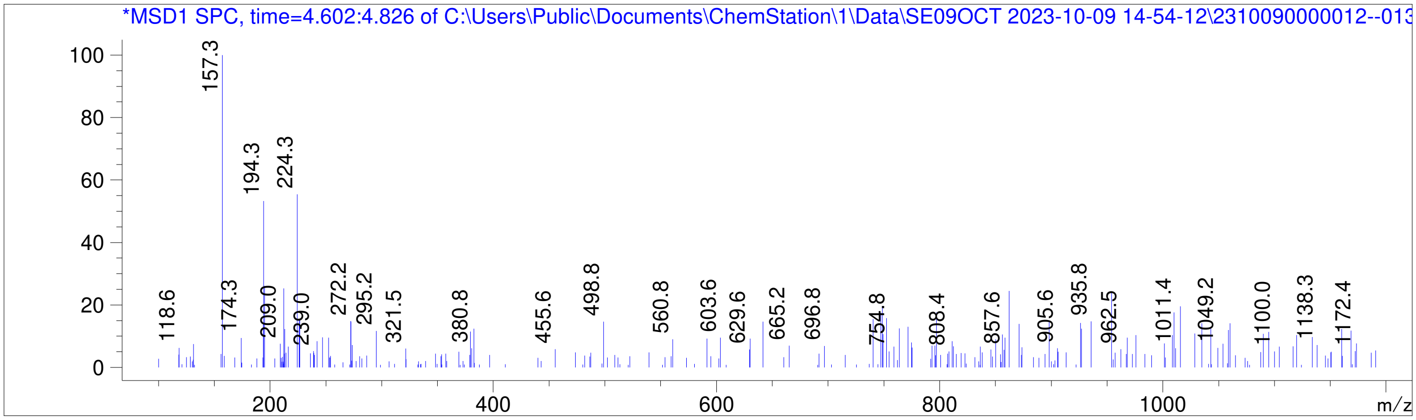

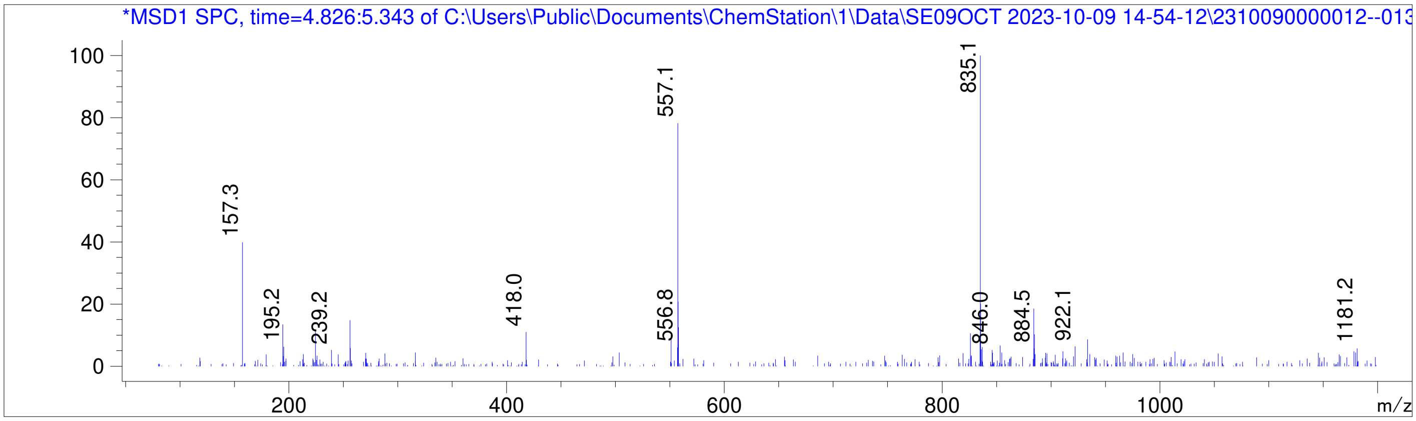

Supplement: Supplementary file 2 — Data S1 and S2 [file sciadv.adr0006_data_s1_and_s2.zip › Supplementary Dataset 1-LCMS DATA/LCMS PNA Hexamers A-T/LCMS A6 RT/14d/CPT22010446-13-A-1-14d.pdf]
