# Supplementary material for: Astrobiological implications of the stability and reactivity of peptide nucleic acid (PNA) in concentrated sulfuric acid
Source: Sci Adv. 2025 Mar 26;11(13):eadr0006. doi: 10.1126/sciadv.adr0006 (PMC11939054; doi:10.1126/sciadv.adr0006)
Supplement: Supplementary file 2 — Data S1 and S2 [file sciadv.adr0006_data_s1_and_s2.zip › Supplementary Dataset 1-LCMS DATA/LCMS PNA Hexamers A-T/LCMS A6 RT/14d/CPT22010446-13-A2-14d.pdf]

Sample-> CPT22010446-13-A2-14d

==

Injection Date :	Wed, 11. Oct. 2023	Seq Line	:	21
		Location	:	19
		Inj. Vol.	:	2 µl

Acq. Method   : C:\Users\Public\Documents\ChemStation\1\Data\SE11OCT 2023-10-11 13-11-06\22010446 LCMS-6.M

Analysis Method : C:\Users\Public\Documents\ChemStation\1\Data\SE11OCT 2023-10-11 13-11-06\22010446 LCMS-6.M (Sequence Method)

Waters XBridge Phenyl (4.6 * 150 mm; 3.5 µm); 0.05% TFA (aq) / AcN: 100/0 (0.0 min) --> (6.0 min) --> 70/30 (0.0 min) --> (2.0 min) --> 10/90 (2.0 min); Flow: 1.0 ml/min; MSD1 = positive; MSD2 = negative

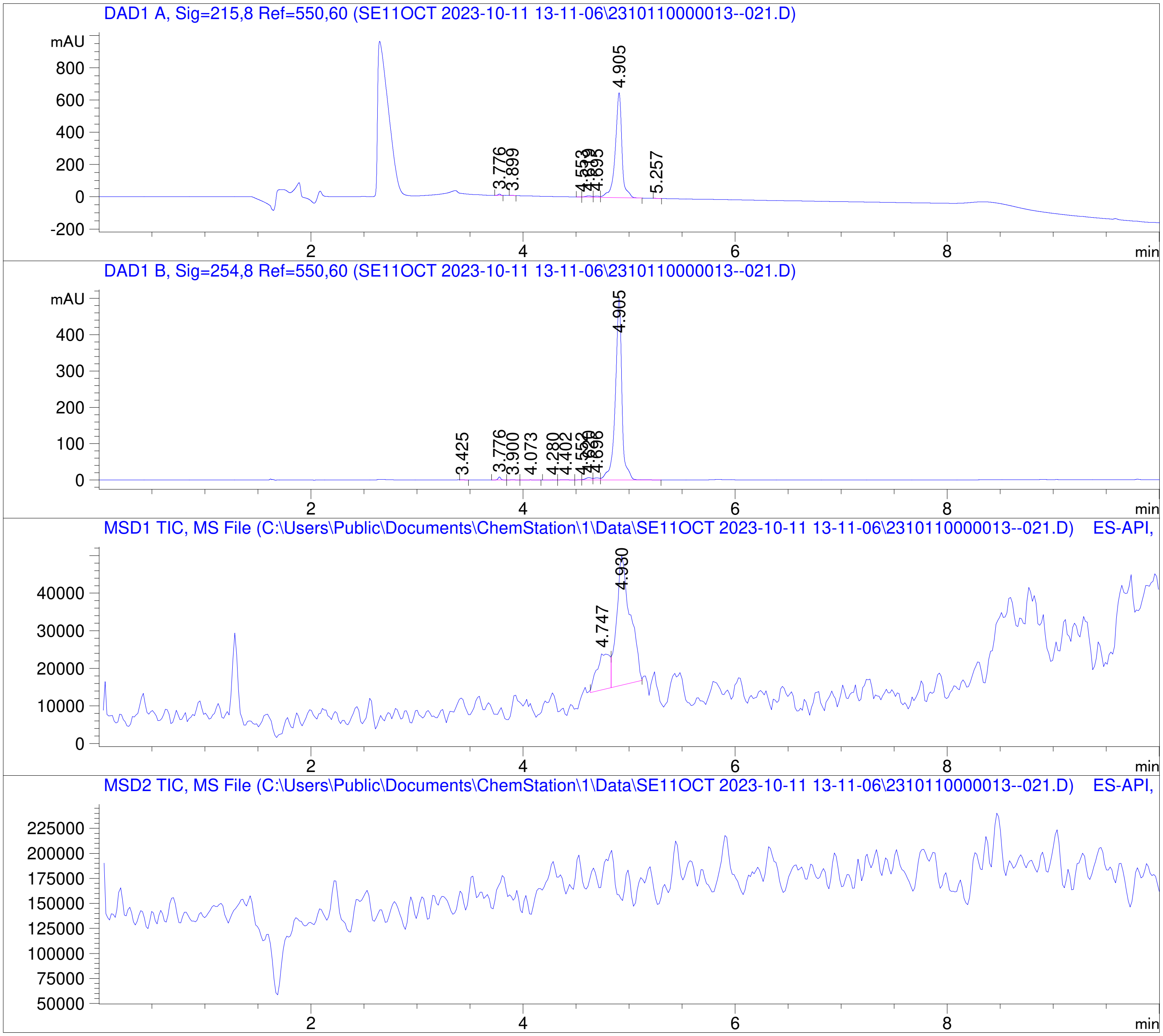

DAD1 A, Sig=215,8 Ref=550,60

Peak #	Ret. Time [min]	Area [mV *s]	Area %
1	3.776	13.216	0.453
2	3.899	1.775	0.061
3	4.553	1.100	0.038
4	4.619	31.597	1.083
5	4.695	25.815	0.885
6	4.905	2843.573	97.462
7	5.257	0.543	0.019

DAD1 B, Sig=254,8 Ref=550,60

Peak #	Ret. Time [min]	Area [mV *s]	Area %
1	3.425	1.498	0.067
2	3.776	13.986	0.628
3	3.900	2.198	0.099
4	4.073	1.421	0.064
5	4.280	0.994	0.045
6	4.402	2.090	0.094
7	4.552	1.346	0.060
8	4.620	25.638	1.152
9	4.696	20.647	0.927
10	4.905	2156.581	96.864

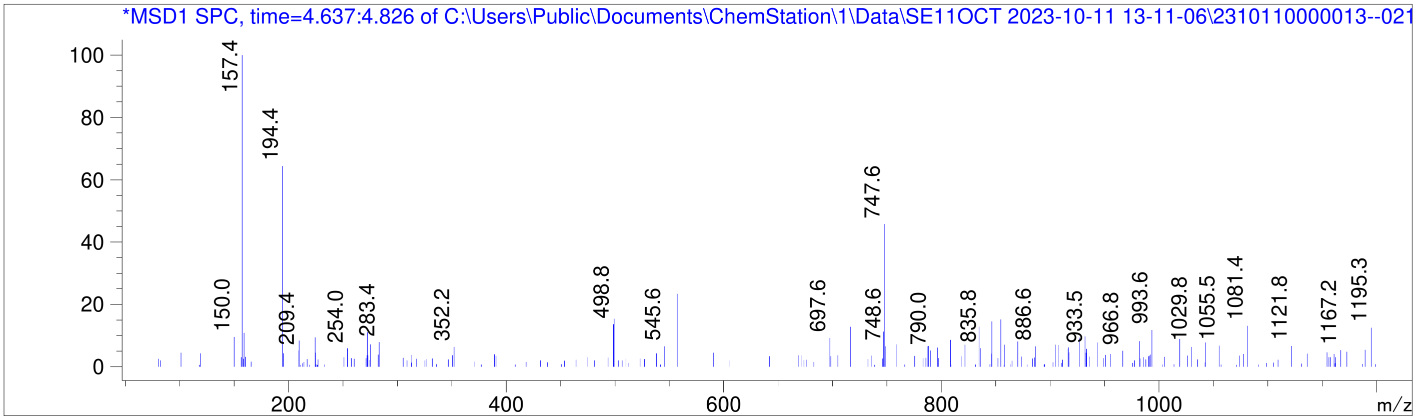
*MSD1 SPC, time=4.637:4.826 of C:\Users\Public\Documents\ChemStation\1\Data\SE11OCT 2023-10-11 13-11-06\2310110000013--021
157.4
194.4
747.6
150.0
209.4
254.0
283.4
352.2
498.8
545.6
697.6
748.6
790.0
835.8
886.6
933.5
966.8
993.6
1029.8
1055.5
1081.4
1121.8
1167.2
1195.3
0
20
40
60
80
100
200
400
600
800
1000
m/z

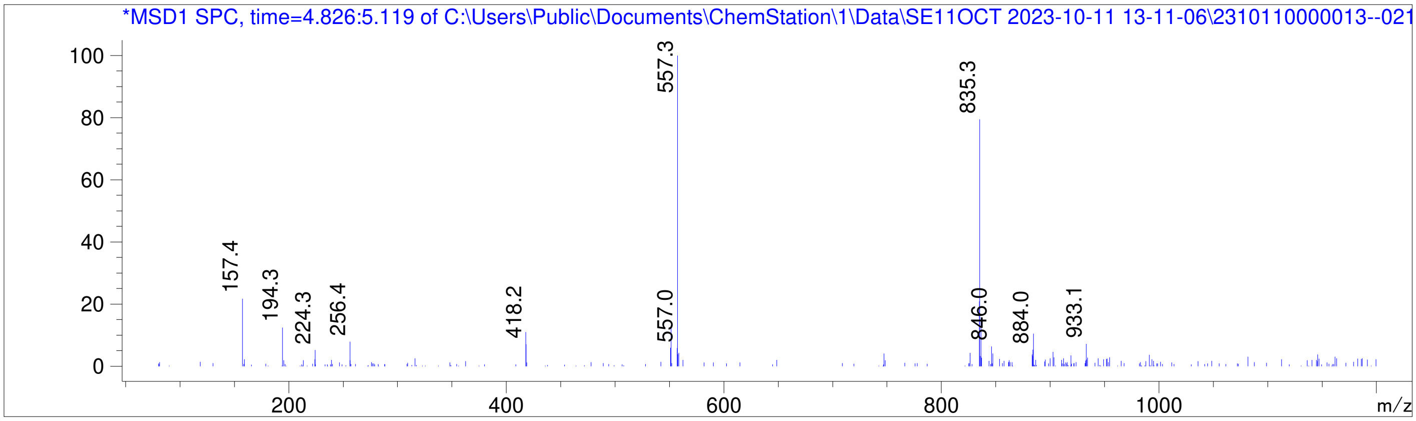
*MSD1 SPC, time=4.826:5.119 of C:\Users\Public\Documents\ChemStation\1\Data\SE11OCT 2023-10-11 13-11-06\2310110000013--021
557.3
835.3
157.4
194.3
224.3
256.4
418.2
557.0
846.0
884.0
933.1
0
20
40
60
80
100
200
400
600
800
1000
m/z
