# Supplementary material for: Astrobiological implications of the stability and reactivity of peptide nucleic acid (PNA) in concentrated sulfuric acid
Source: Sci Adv. 2025 Mar 26;11(13):eadr0006. doi: 10.1126/sciadv.adr0006 (PMC11939054; doi:10.1126/sciadv.adr0006)
Supplement: Supplementary file 2 — Data S1 and S2 [file sciadv.adr0006_data_s1_and_s2.zip › Supplementary Dataset 1-LCMS DATA/LCMS PNA Hexamers A-T/LCMS A6 RT/14d/CPT22010446-13-A3-14d.pdf]

==

Injection Date	:	Tue, 17. Oct. 2023	Seq Line	:	13
			Location	:	19
			Inj. Vol.	:	2 µl

Acq. Method : C:\Users\Public\Documents\ChemStation\1\Data\SE17OCT 2023-10-17 07-53-29\22010446 LCMS-6.M

Analysis Method : C:\Users\Public\Documents\ChemStation\1\Data\SE17OCT 2023-10-17 07-53-29\22010446 LCMS-6.M (Sequence Method)

Waters XBridge Phenyl (4.6 * 150 mm; 3.5 µm); 0.05% TFA (aq) / AcN: 100/0 (0.0 min) --> (6.0 min) --> 70/30 (0.0 min) --> (2.0 min) --> 10/90 (2.0 min); Flow: 1.0 ml/min; MSD1 = positive; MSD2 = negative

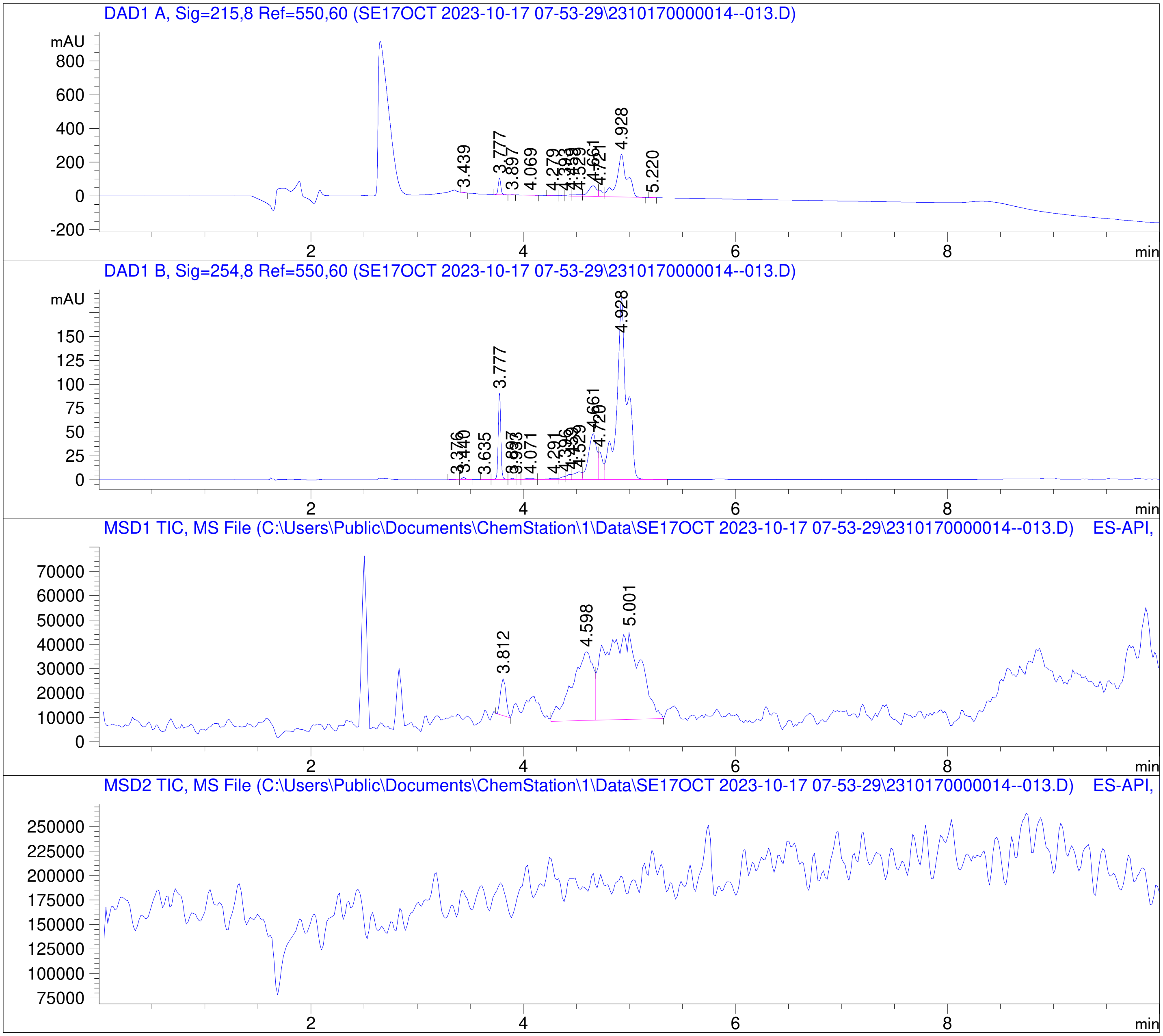

===

DAD1 A, Sig=215,8 Ref=550,60

Peak #	Ret. Time [min]	Area [mV *s]	Area %
1	3.439	2.937	0.120
2	3.777	163.396	6.696
3	3.897	1.812	0.074
4	4.069	6.768	0.277
5	4.279	3.066	0.126
6	4.393	7.258	0.297
7	4.459	20.493	0.840
8	4.529	51.960	2.129
9	4.661	341.189	13.981
10	4.721	104.421	4.279
11	4.928	1736.528	71.158
12	5.220	0.557	0.023

DAD1 B, Sig=254,8 Ref=550,60

Peak #	Ret. Time [min]	Area [mV *s]	Area %
1	3.376	1.153	0.061
2	3.440	5.054	0.269
3	3.635	0.418	0.022
4	3.777	150.772	8.020
5	3.897	3.080	0.164
6	3.933	1.024	0.054
7	4.071	6.970	0.371
8	4.291	8.368	0.445
9	4.396	8.877	0.472
10	4.459	17.602	0.936
11	4.529	40.993	2.181
12	4.661	253.735	13.497
13	4.720	82.620	4.395
14	4.928	1299.316	69.113

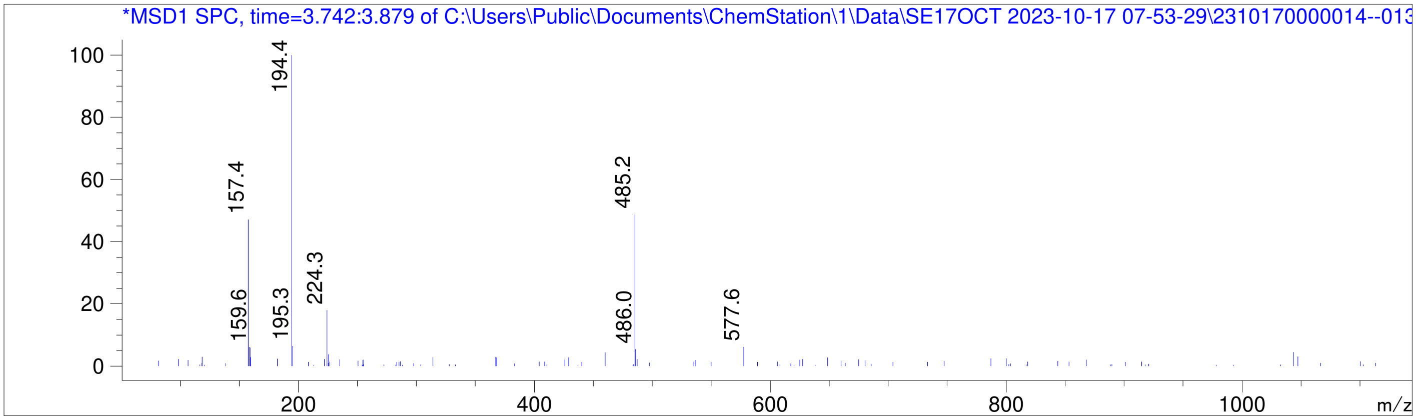
*MSD1 SPC, time=3.742:3.879 of C:\Users\Public\Documents\ChemStation\1\Data\SE17OCT 2023-10-17 07-53-29\2310170000014--013
194.4
157.4
485.2
224.3
159.6
195.3
486.0
577.6
m/z

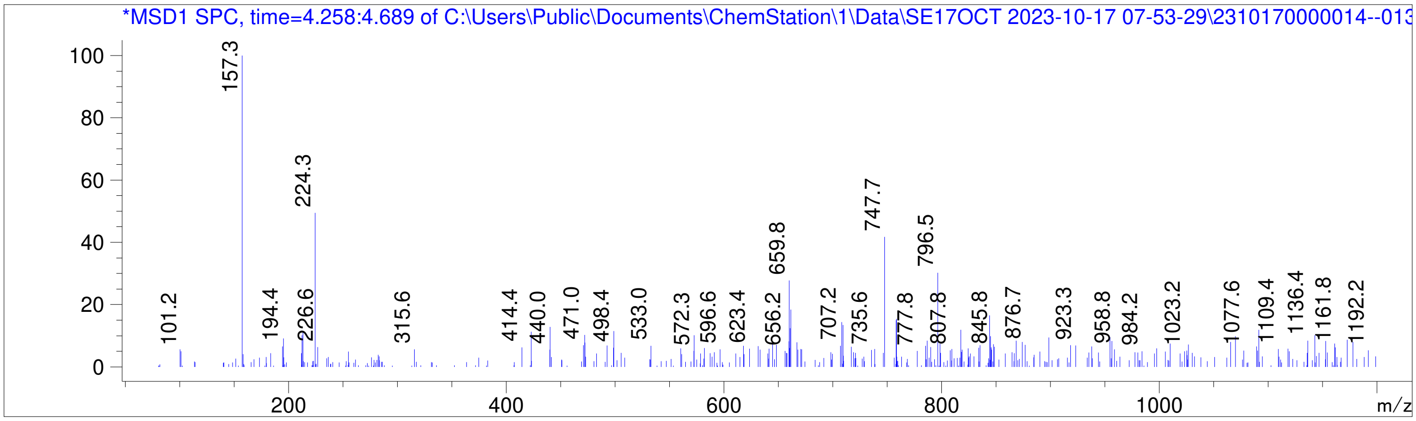
*MSD1 SPC, time=4.258:4.689 of C:\Users\Public\Documents\ChemStation\1\Data\SE17OCT 2023-10-17 07-53-29\2310170000014--013
157.3
224.3
747.7
796.5
659.8
101.2
194.4
226.6
315.6
414.4
440.0
471.0
498.4
533.0
572.3
596.6
623.4
656.2
707.2
735.6
777.8
807.8
845.8
876.7
923.3
958.8
984.2
1023.2
1077.6
1109.4
1136.4
1161.8
1192.2
m/z

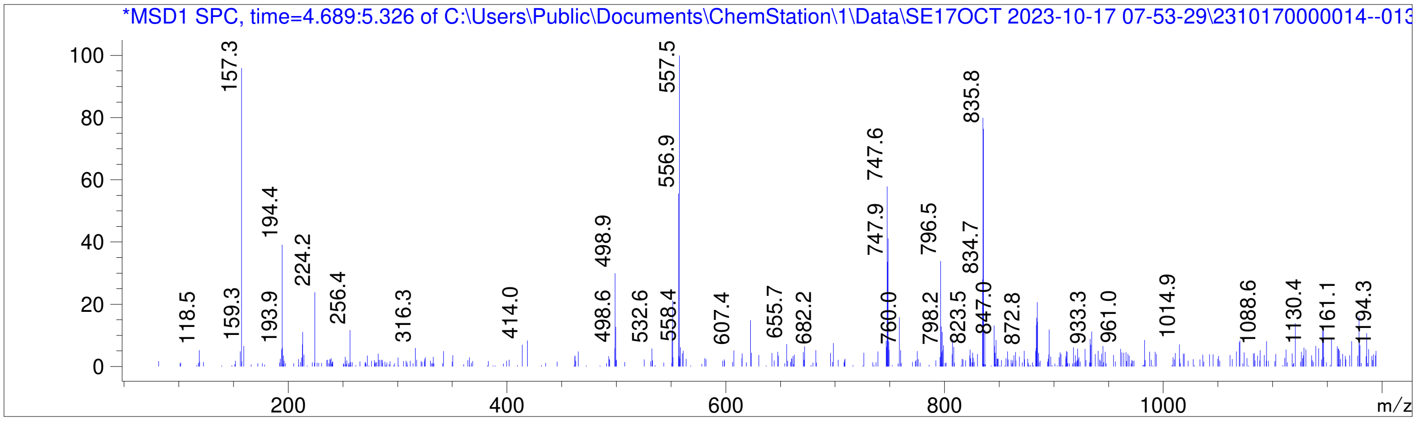
*MSD1 SPC, time=4.689:5.326 of C:\Users\Public\Documents\ChemStation\1\Data\SE17OCT 2023-10-17 07-53-29\2310170000014--013
157.3
557.5
835.8
556.9
747.6
194.4
498.9
747.9
796.5
224.2
834.7
256.4
118.5
159.3
193.9
316.3
414.0
498.6
532.6
558.4
607.4
655.7
682.2
760.0
798.2
823.5
847.0
872.8
933.3
961.0
1014.9
1088.6
1130.4
1161.1
1194.3
m/z
