# Supplementary material for: Astrobiological implications of the stability and reactivity of peptide nucleic acid (PNA) in concentrated sulfuric acid
Source: Sci Adv. 2025 Mar 26;11(13):eadr0006. doi: 10.1126/sciadv.adr0006 (PMC11939054; doi:10.1126/sciadv.adr0006)

Data -> C:\Users\Public\Documents\ChemStation\1\Data\09. September\SE26SEP\SE26SEP 202->
Sample-> CPT22010446-13-A

===

Injection Date :	Tue, 26. Sep. 2023	Seq Line :	4
		Location :	56
		Inj. Vol. :	2 µl

Acq. Method : C:\Users\Public\Documents\ChemStation\1\Data\SE26SEP 2023-09-26 10-10-32\22010446 LCMS-6.M
Analysis Method : C:\Users\Public\Documents\ChemStation\1\Data\09. September\SE26SEP\SE26SEP 2023-09-26 10-10-32\22010446 LCMS-6.M (Sequence->
Waters XBridge Phenyl (4.6 * 150 mm; 3.5 µm); 0.05% TFA (aq) / AcN: 100/0 (0.0 min) --> (6.0 min) --> 70/30 (0.0 min) --> (2.0 min) --> 10/90 (2.0 min); Flow: 1.0 ml/min;
MSD1 = positive; MSD2 = negative

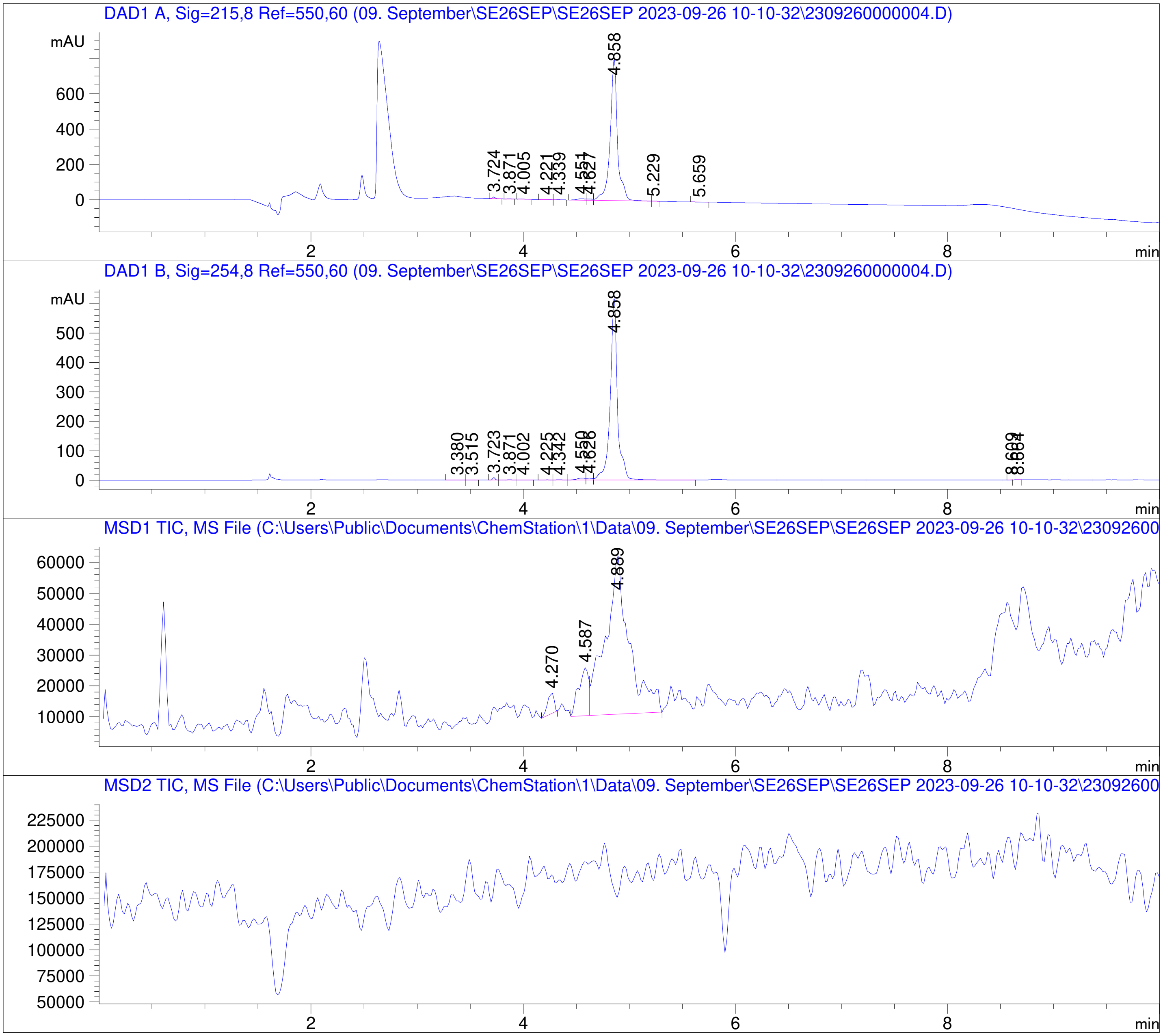

DAD1 A, Sig=215,8 Ref=550,60

Peak #	Ret. Time [min]	Area [mV *s]	Area %
1	3.724	16.874	0.421
2	3.871	4.619	0.115
3	4.005	3.411	0.085
4	4.221	4.620	0.115
5	4.339	5.744	0.143
6	4.551	44.456	1.110
7	4.627	28.191	0.704
8	4.858	3894.903	97.234
9	5.229	1.551	0.039
10	5.659	1.336	0.033

DAD1 B, Sig=254,8 Ref=550,60

Peak #	Ret. Time [min]	Area [mV *s]	Area %
1	3.380	2.895	0.095
2	3.515	0.338	0.011
3	3.723	14.565	0.476
4	3.871	4.755	0.155
5	4.002	3.129	0.102
6	4.225	3.271	0.107
7	4.342	4.406	0.144
8	4.550	35.643	1.164
9	4.626	23.604	0.771
10	4.858	2970.055	96.971
11	8.609	0.057	0.002
12	8.664	0.113	0.004

Data -> C:\Users\Public\Documents\ChemStation\1\Data\09. September\SE26SEP\SE26SEP 202->
Sample-> CPT22010446-13-A
==

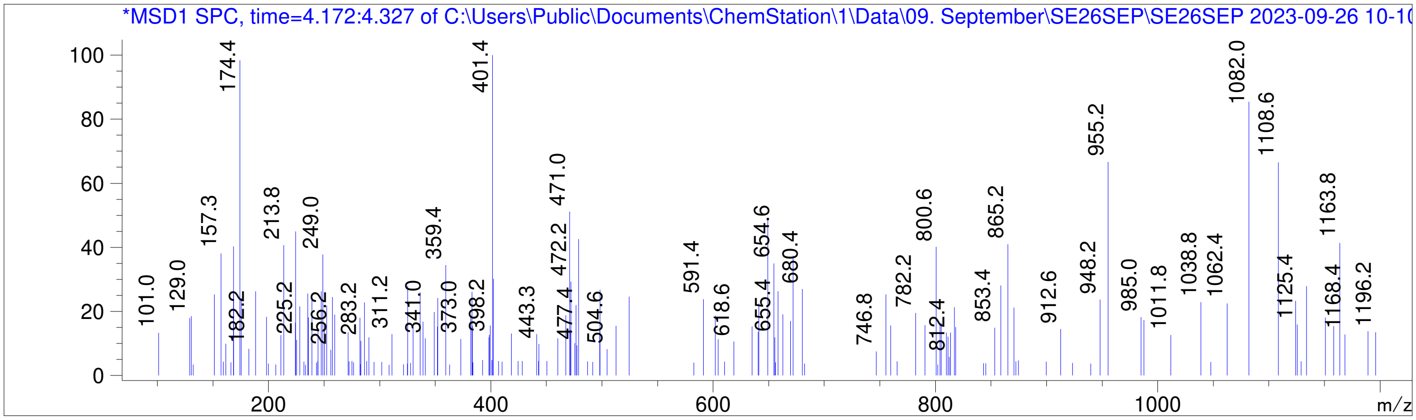

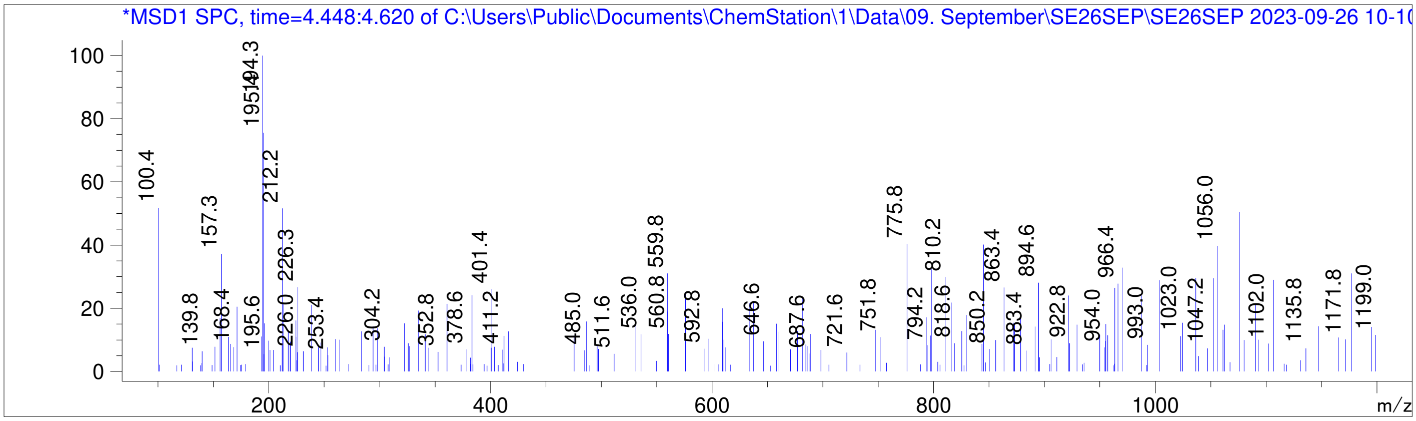

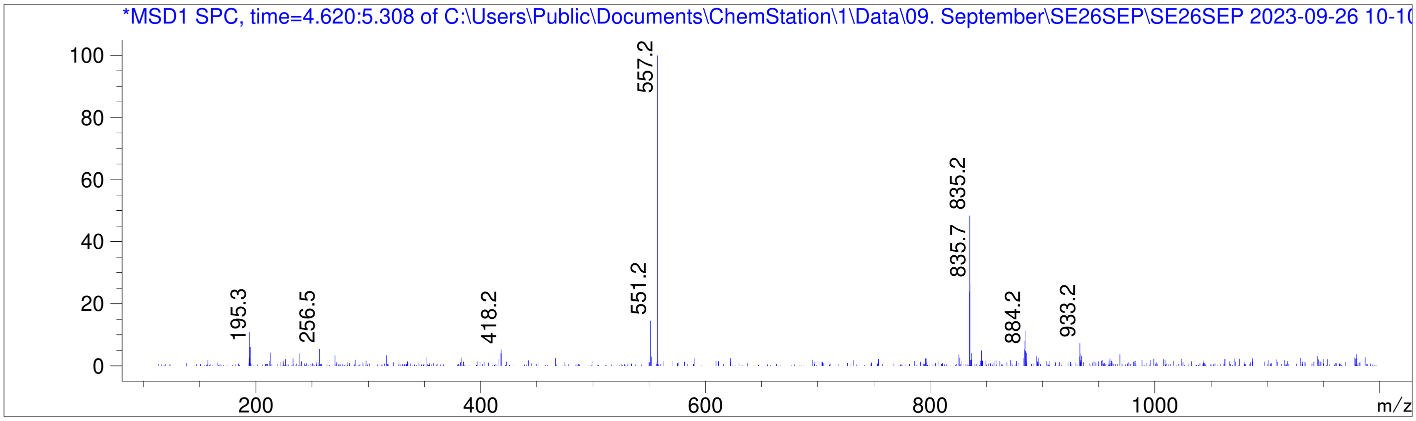

Supplement: Supplementary file 2 — Data S1 and S2 [file sciadv.adr0006_data_s1_and_s2.zip › Supplementary Dataset 1-LCMS DATA/LCMS PNA Hexamers A-T/LCMS A6 RT/1h/LCMS-6_CPT22010446-13-A.pdf]
