# Supplementary material for: Astrobiological implications of the stability and reactivity of peptide nucleic acid (PNA) in concentrated sulfuric acid
Source: Sci Adv. 2025 Mar 26;11(13):eadr0006. doi: 10.1126/sciadv.adr0006 (PMC11939054; doi:10.1126/sciadv.adr0006)
Supplement: Supplementary file 2 — Data S1 and S2 [file sciadv.adr0006_data_s1_and_s2.zip › Supplementary Dataset 1-LCMS DATA/LCMS PNA Hexamers A-T/LCMS A6 RT/1h/LCMS-6_CPT22010446-13-A-2.pdf]

==

Injection Date : Tue, 26. Sep. 2023		Seq Line :	16
		Location :	66
		Inj. Vol. :	2 µl

Acq. Method : C:\Users\Public\Documents\ChemStation\1\Data\SE26SEP 2023-09-26 10-10-32\22010446 LCMS-6.M

Analysis Method : C:\Users\Public\Documents\ChemStation\1\Data\09. September\SE26SEP\SE26SEP 2023-09-26 10-10-32\22010446 LCMS-6.M (Sequence->

Waters XBridge Phenyl (4.6 * 150 mm; 3.5 µm); 0.05% TFA (aq) / AcN: 100/0 (0.0 min) --> (6.0 min) --> 70/30 (0.0 min) --> (2.0 min) --> 10/90 (2.0 min); Flow: 1.0 ml/min; MSD1 = positive; MSD2 = negative

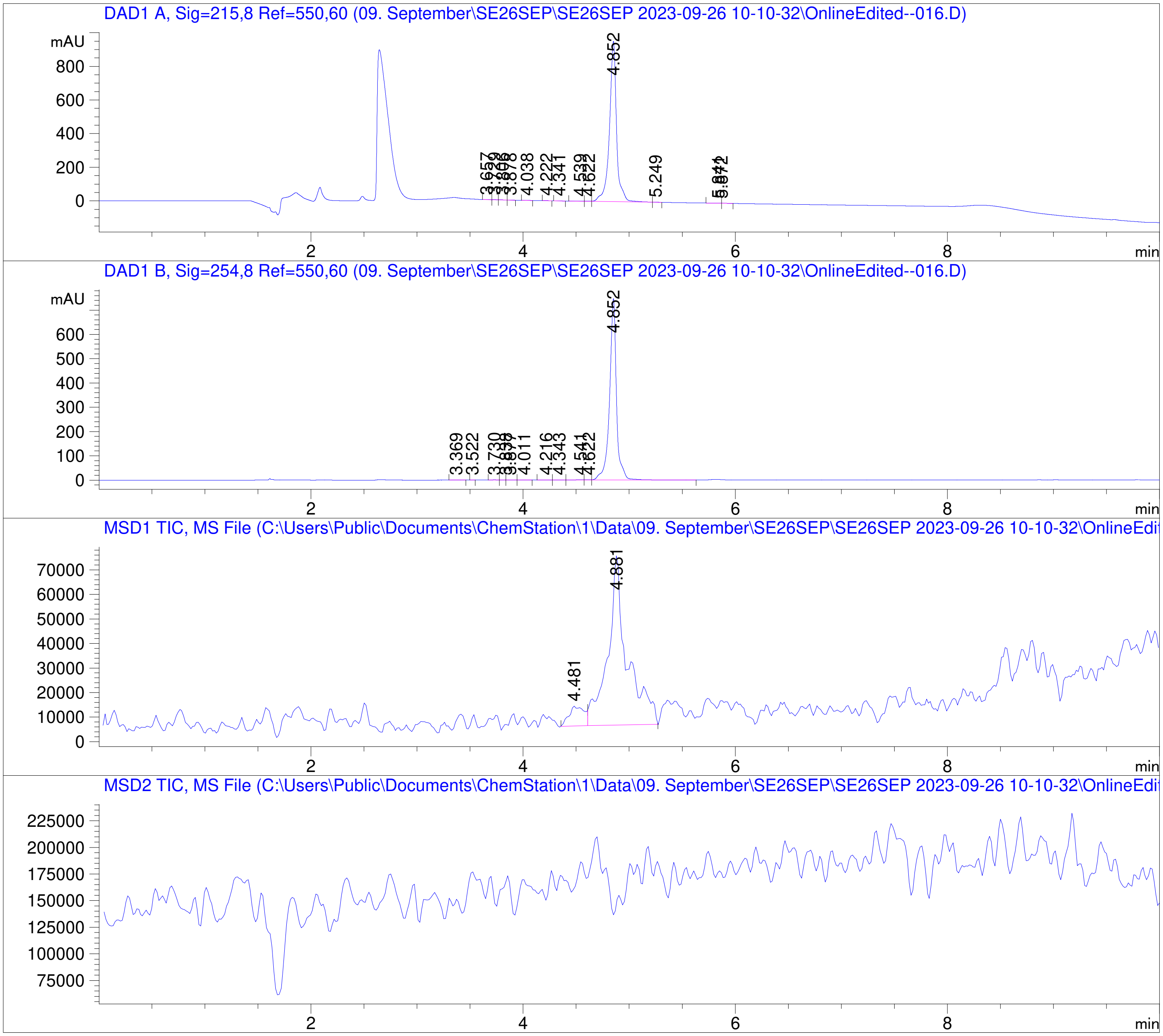

===

DAD1 A, Sig=215,8 Ref=550,60

Peak #	Ret. Time [min]	Area [mV *s]	Area %
1	3.657	2.179	0.049
2	3.729	3.079	0.069
3	3.806	0.887	0.020
4	3.878	1.708	0.038
5	4.038	1.106	0.025
6	4.222	0.907	0.020
7	4.341	1.059	0.024
8	4.539	8.781	0.196
9	4.622	7.474	0.166
10	4.852	4452.605	99.182
11	5.249	1.724	0.038
12	5.841	5.113	0.114
13	5.872	2.694	0.060

DAD1 B, Sig=254,8 Ref=550,60

Peak #	Ret. Time [min]	Area [mV *s]	Area %
1	3.369	1.103	0.032
2	3.522	0.062	0.002
3	3.730	2.977	0.087
4	3.838	0.305	0.009
5	3.877	1.847	0.054
6	4.011	1.147	0.033
7	4.216	1.224	0.036
8	4.343	1.104	0.032
9	4.541	7.246	0.211
10	4.622	5.928	0.173
11	4.852	3410.323	99.332

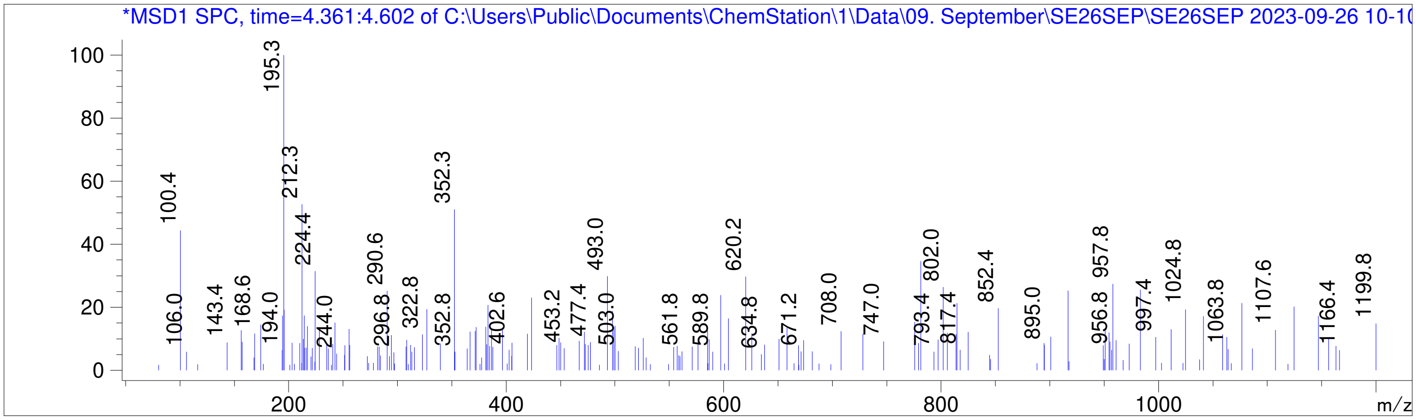
*MSD1 SPC, time=4.361:4.602 of C:\Users\Public\Documents\ChemStation\1\Data\09. September\SE26SEP\SE26SEP 2023-09-26 10-1
100
80
60
40
20
0
200
400
600
800
1000
m/z
195.3
212.3
352.3
100.4
224.4
290.6
493.0
620.2
802.0
957.8
1024.8
852.4
1107.6
1199.8
106.0
143.4
168.6
194.0
244.0
296.8
322.8
352.8
402.6
453.2
477.4
503.0
561.8
589.8
634.8
671.2
708.0
747.0
793.4
817.4
895.0
956.8
997.4
1063.8
1166.4

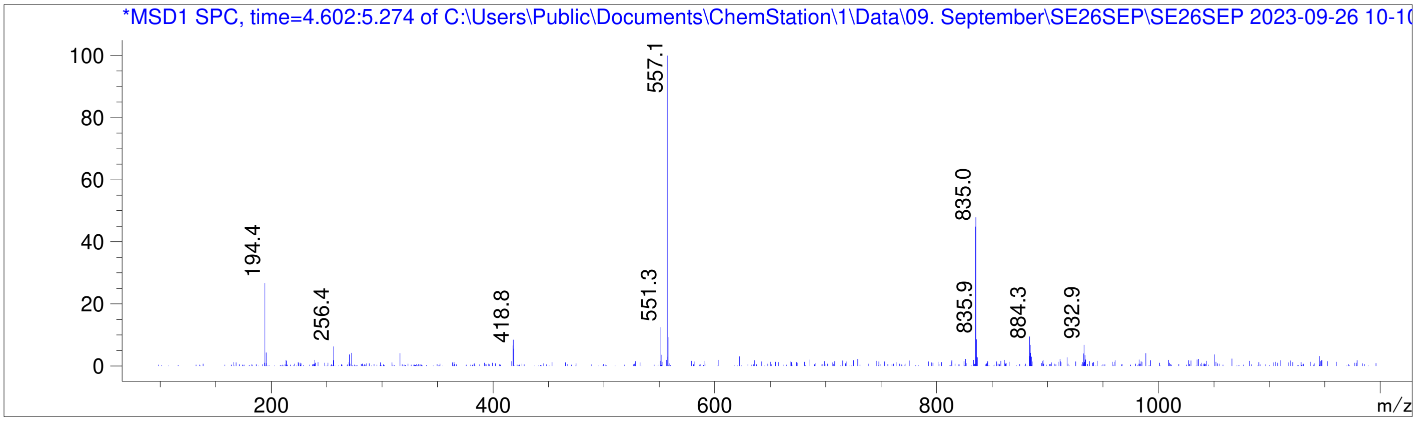
*MSD1 SPC, time=4.602:5.274 of C:\Users\Public\Documents\ChemStation\1\Data\09. September\SE26SEP\SE26SEP 2023-09-26 10-1
100
80
60
40
20
0
200
400
600
800
1000
m/z
557.1
835.0
194.4
256.4
418.8
551.3
835.9
884.3
932.9
