# Supplementary material for: Astrobiological implications of the stability and reactivity of peptide nucleic acid (PNA) in concentrated sulfuric acid
Source: Sci Adv. 2025 Mar 26;11(13):eadr0006. doi: 10.1126/sciadv.adr0006 (PMC11939054; doi:10.1126/sciadv.adr0006)
Supplement: Supplementary file 2 — Data S1 and S2 [file sciadv.adr0006_data_s1_and_s2.zip › Supplementary Dataset 1-LCMS DATA/LCMS PNA Hexamers A-T/LCMS A6 RT/1h/LCMS-6_CPT22010446-13-A3.pdf]

Data -> C:\Users\Public\Documents\ChemStation\1\Data\Se02Oct\SE02OCT 2023-10-02 11-39-->
Sample-> CPT22010446-13-A3

===

Injection Date : Mon, 2. Oct. 2023 Seq Line : 7
Location : 37
Inj. Vol. : 2 µl

Acq. Method : C:\Users\Public\Documents\ChemStation\1\Data\SE02OCT 2023-10-02 11-39-16\22010446 LCMS-6.M
Analysis Method : C:\Users\Public\Documents\ChemStation\1\Data\Se02Oct\SE02OCT 2023-10-02 11-39-16\22010446 LCMS-6.M (Sequence Method)
Waters XBridge Phenyl (4.6 * 150 mm; 3.5 µm); 0.05% TFA (aq) / AcN: 100/0 (0.0 min) --> (6.0 min) --> 70/30 (0.0 min) --> (2.0 min) --> 10/90 (2.0 min); Flow: 1.0 ml/min;
MSD1 = positive; MSD2 = negative

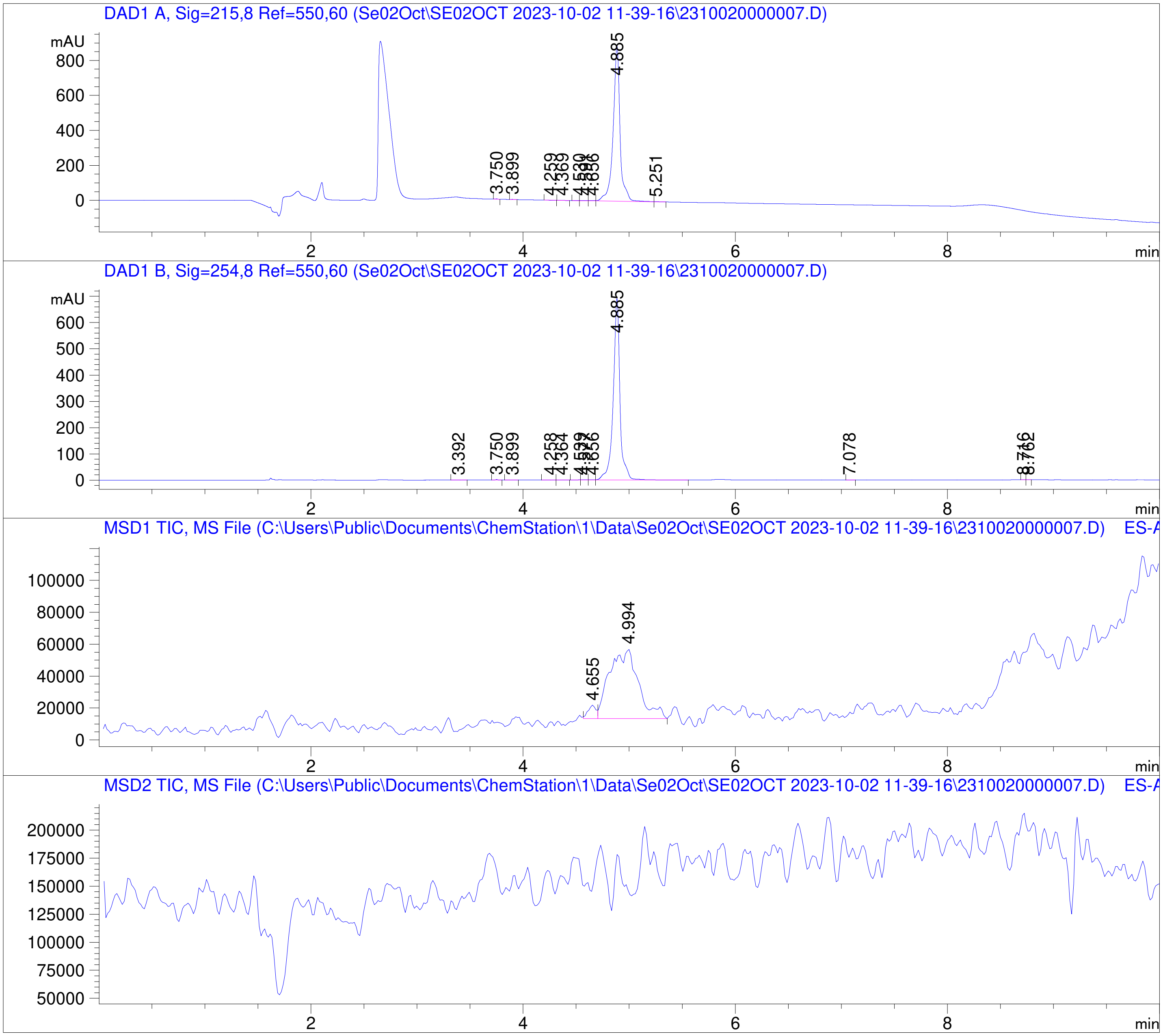

DAD1 A, Sig=215,8 Ref=550,60

Peak #	Ret. Time [min]	Area [mV *s]	Area %
1	3.750	2.938	0.072
2	3.899	1.389	0.034
3	4.259	1.193	0.029
4	4.369	1.834	0.045
5	4.530	1.891	0.046
6	4.591	8.458	0.208
7	4.656	9.193	0.226
8	4.885	4039.713	99.258
9	5.251	3.300	0.081

DAD1 B, Sig=254,8 Ref=550,60

Peak #	Ret. Time [min]	Area [mV *s]	Area %
1	3.392	1.284	0.041
2	3.750	3.141	0.101
3	3.899	1.597	0.051
4	4.258	1.297	0.042
5	4.364	1.279	0.041
6	4.539	2.194	0.071
7	4.577	5.923	0.191
8	4.656	6.047	0.195
9	4.885	3082.098	99.246
10	7.078	0.156	0.005
11	8.716	0.360	0.012
12	8.762	0.137	0.004

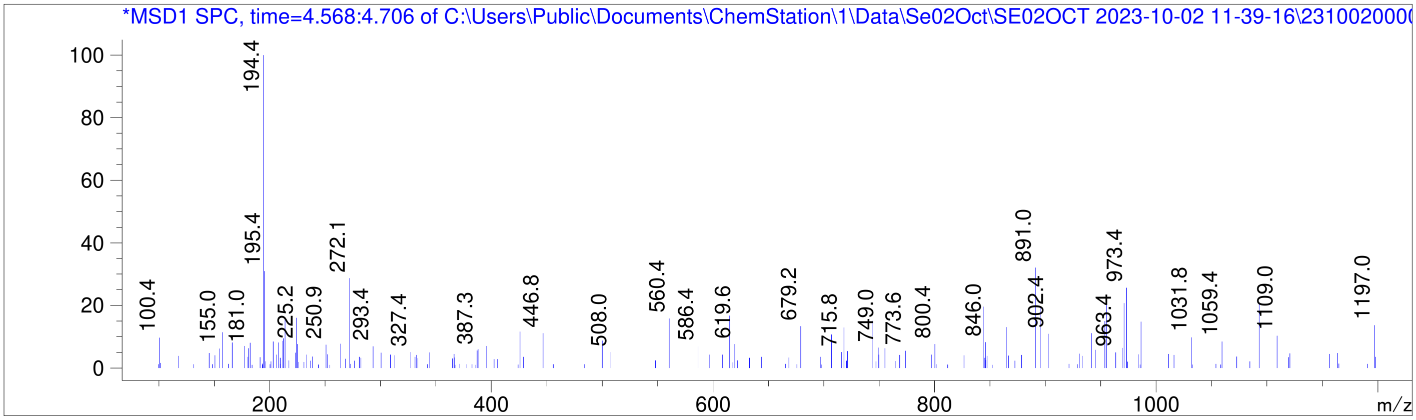
*MSD1 SPC, time=4.568:4.706 of C:\Users\Public\Documents\ChemStation\1\Data\Se02Oct\SE02OCT 2023-10-02 11-39-16\2310020000
100
80
60
40
20
0
194.4
195.4
272.1
100.4
155.0
181.0
225.2
250.9
293.4
327.4
387.3
446.8
508.0
560.4
586.4
619.6
679.2
715.8
749.0
773.6
800.4
846.0
891.0
902.4
963.4
973.4
1031.8
1059.4
1109.0
1197.0
200
400
600
800
1000
m/z

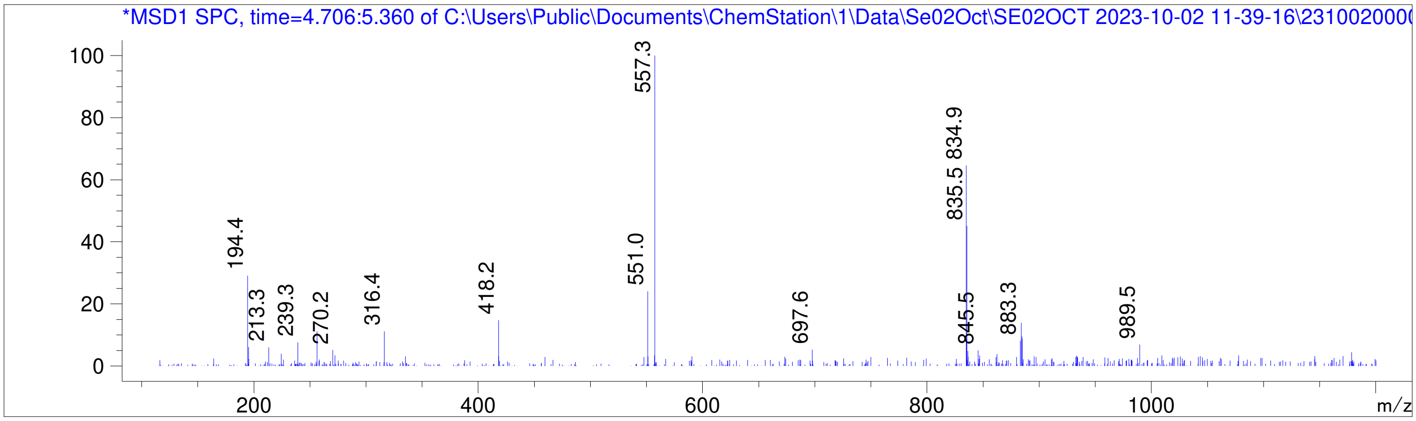
*MSD1 SPC, time=4.706:5.360 of C:\Users\Public\Documents\ChemStation\1\Data\Se02Oct\SE02OCT 2023-10-02 11-39-16\2310020000
100
80
60
40
20
0
557.3
835.5 834.9
194.4
213.3
239.3
270.2
316.4
418.2
551.0
697.6
845.5
883.3
989.5
200
400
600
800
1000
m/z
