# Supplementary material for: Astrobiological implications of the stability and reactivity of peptide nucleic acid (PNA) in concentrated sulfuric acid
Source: Sci Adv. 2025 Mar 26;11(13):eadr0006. doi: 10.1126/sciadv.adr0006 (PMC11939054; doi:10.1126/sciadv.adr0006)
Supplement: Supplementary file 2 — Data S1 and S2 [file sciadv.adr0006_data_s1_and_s2.zip › Supplementary Dataset 1-LCMS DATA/LCMS PNA Hexamers A-T/LCMS A6 RT/24h/LCMS-6_CPT22010446-13-A2-24h.pdf]

Data -> C:\Users\Public\Documents\ChemStation\1\Data\09. September\Se27Sep\SE27SEP 202->
Sample-> CPT22010446-13-A2-24h

==

Injection Date	:	Wed, 27. Sep. 2023	Seq Line	:	4
			Location	:	3
			Inj. Vol.	:	2 µl

Acq. Method : C:\Users\Public\Documents\ChemStation\1\Data\SE27SEP 2023-09-27 12-47-15\22010446 LCMS-6.M
Analysis Method : C:\Users\Public\Documents\ChemStation\1\Data\09. September\Se27Sep\SE27SEP 2023-09-27 12-47-15\22010446 LCMS-6.M (Sequence->
Waters XBridge Phenyl (4.6 * 150 mm; 3.5 µm); 0.05% TFA (aq) / AcN: 100/0 (0.0 min) --> (6.0 min) --> 70/30 (0.0 min) --> (2.0 min) --> 10/90 (2.0 min); Flow: 1.0 ml/min;
MSD1 = positive; MSD2 = negative

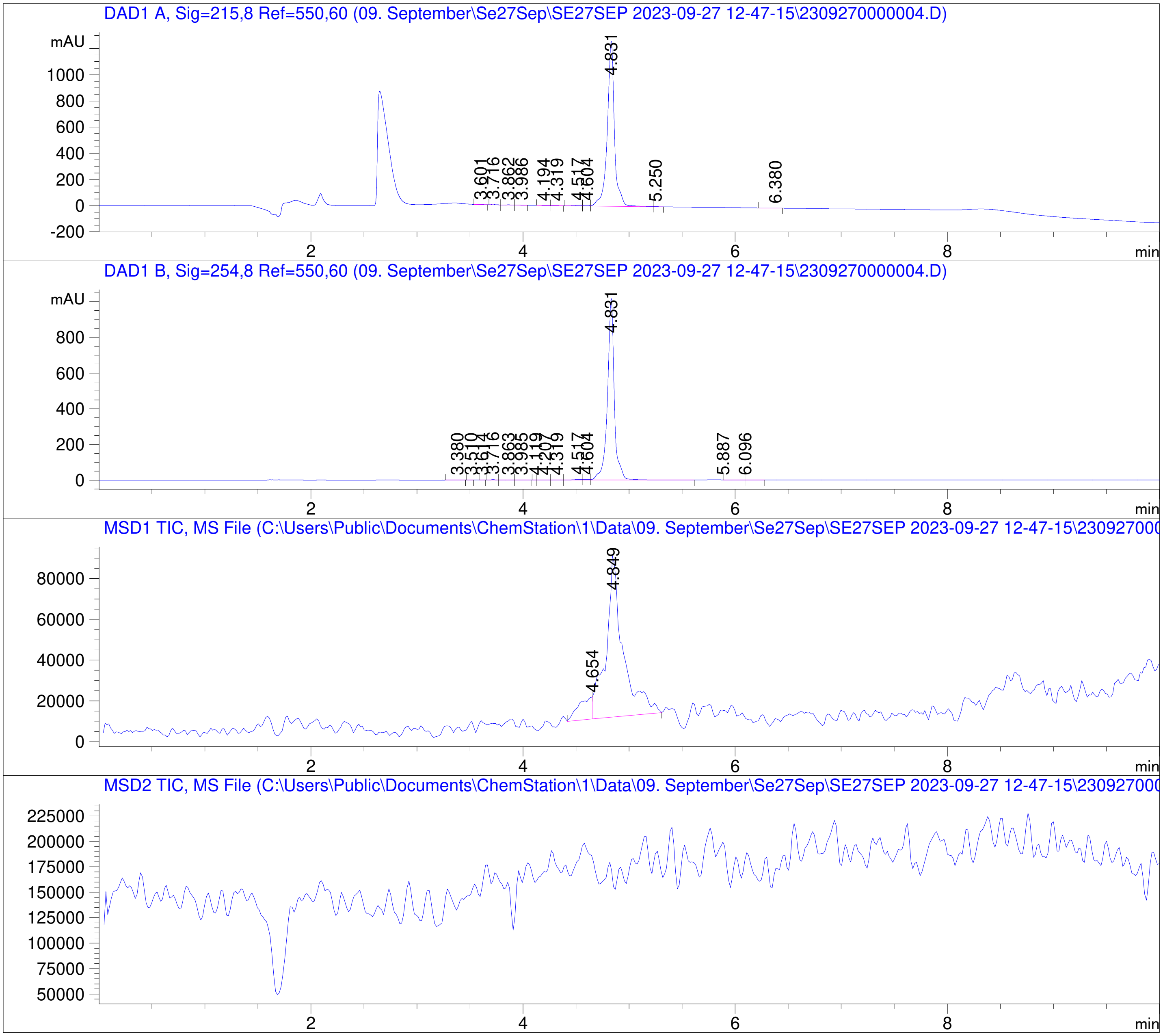

DAD1 A, Sig=215,8 Ref=550,60

Peak #	Ret. Time [min]	Area [mV *s]	Area %
1	3.601	2.928	0.048
2	3.716	11.089	0.183
3	3.862	4.631	0.077
4	3.986	2.457	0.041
5	4.194	2.237	0.037
6	4.319	2.421	0.040
7	4.517	27.127	0.449
8	4.604	22.680	0.375
9	4.831	5963.497	98.674
10	5.250	2.760	0.046
11	6.380	1.839	0.030

DAD1 B, Sig=254,8 Ref=550,60

Peak #	Ret. Time [min]	Area [mV *s]	Area %
1	3.380	1.536	0.033
2	3.510	0.108	0.002
3	3.614	0.055	0.001
4	3.716	8.377	0.178
5	3.863	3.400	0.072
6	3.985	2.156	0.046
7	4.119	0.061	0.001
8	4.207	2.433	0.052
9	4.319	2.722	0.058
10	4.517	20.865	0.444
11	4.604	14.852	0.316
12	4.831	4632.500	98.679
13	5.887	4.569	0.097
14	6.096	0.888	0.019

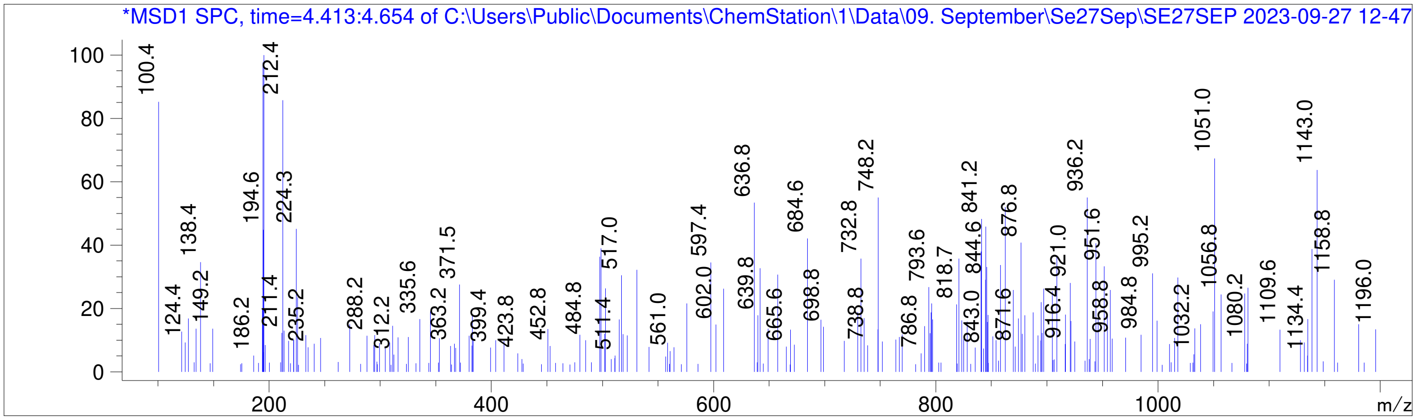
*MSD1 SPC, time=4.413:4.654 of C:\Users\Public\Documents\ChemStation\1\Data\09. September\Se27Sep\SE27SEP 2023-09-27 12-47
100.4
124.4
138.4
149.2
186.2
194.6
211.4
212.4
224.3
235.2
288.2
312.2
335.6
363.2
371.5
399.4
423.8
452.8
484.8
511.4
517.0
561.0
597.4
602.0
636.8
639.8
665.6
684.6
698.8
732.8
738.8
748.2
786.8
793.6
818.7
841.2
843.0
844.6
871.6
876.8
916.4
921.0
936.2
951.6
958.8
984.8
995.2
1032.2
1051.0
1056.8
1080.2
1109.6
1134.4
1143.0
1158.8
1196.0
m/z

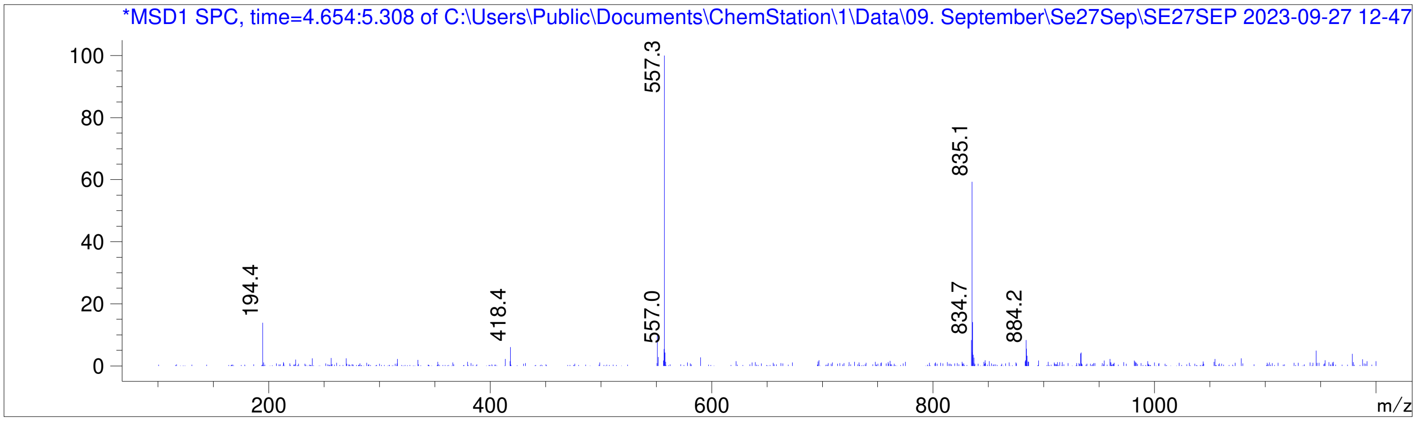
*MSD1 SPC, time=4.654:5.308 of C:\Users\Public\Documents\ChemStation\1\Data\09. September\Se27Sep\SE27SEP 2023-09-27 12-47
194.4
418.4
557.0
557.3
834.7
835.1
884.2
m/z
