# Supplementary material for: Astrobiological implications of the stability and reactivity of peptide nucleic acid (PNA) in concentrated sulfuric acid
Source: Sci Adv. 2025 Mar 26;11(13):eadr0006. doi: 10.1126/sciadv.adr0006 (PMC11939054; doi:10.1126/sciadv.adr0006)
Supplement: Supplementary file 2 — Data S1 and S2 [file sciadv.adr0006_data_s1_and_s2.zip › Supplementary Dataset 1-LCMS DATA/LCMS PNA Hexamers A-T/LCMS A6 RT/24h/LCMS-6_CPT22010446-13-A-24h.pdf]

===

Injection Date : Tue, 26. Sep. 2023   Seq Line   :   25
                                                         Location   :   80
                                                         Inj. Vol. :   2 µl

Acq. Method   : C:\Users\Public\Documents\ChemStation\1\Data\SE26SEP 2023-09-26 16-24-18\22010446 LCMS-6.M
Analysis Method : C:\Users\Public\Documents\ChemStation\1\Data\09. September\SE26SEP\SE26SEP 2023-09-26 16-24-18\22010446 LCMS-6.M (Sequence->
Waters XBridge Phenyl (4.6 * 150 mm; 3.5 µm); 0.05% TFA (aq) / AcN: 100/0 (0.0 min) --> (6.0 min) --> 70/30 (0.0 min) --> (2.0 min) --> 10/90 (2.0 min); Flow: 1.0 ml/min;
MSD1 = positive; MSD2 = negative

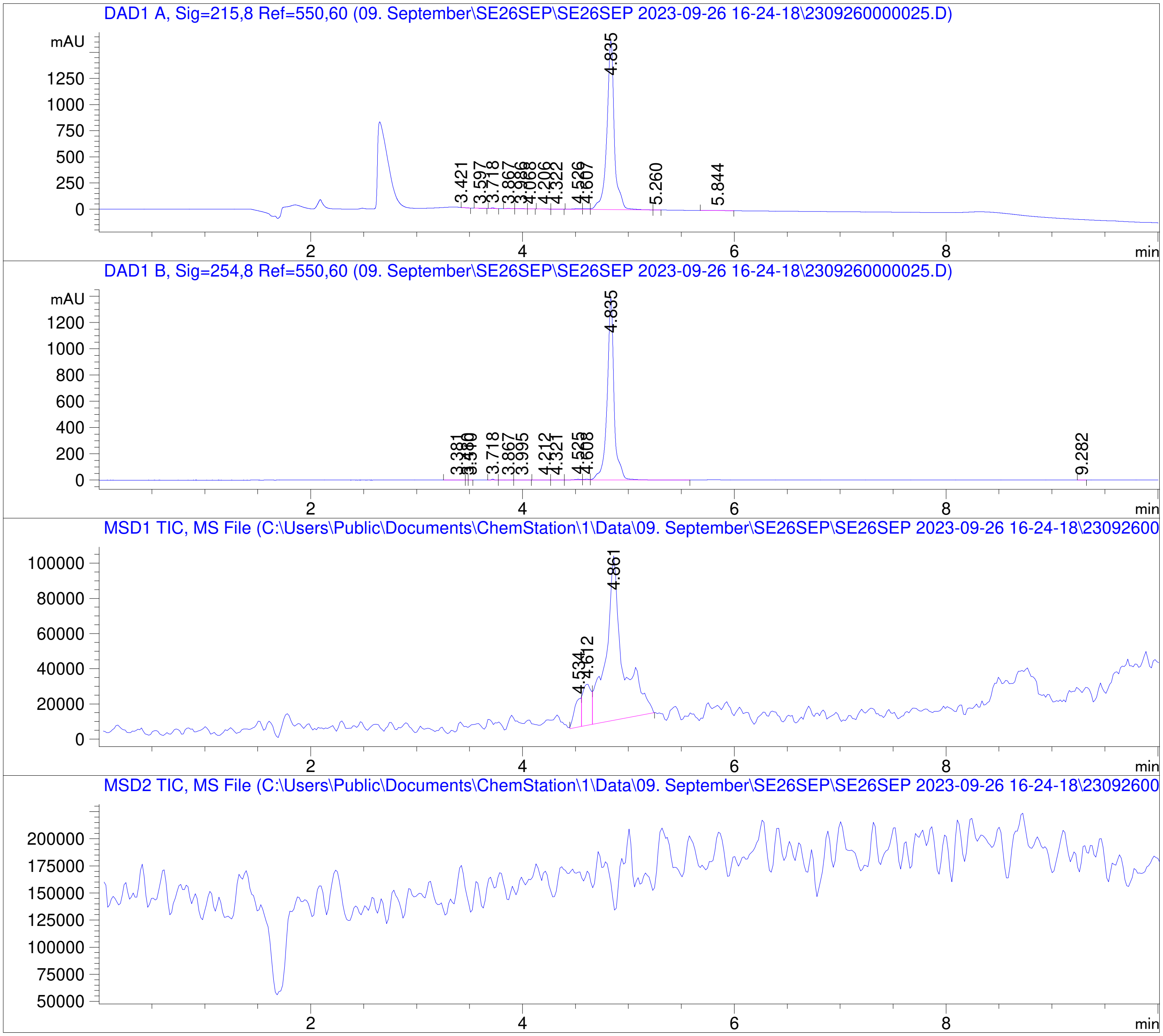

DAD1 A, Sig=215,8 Ref=550,60

Peak #	Ret. Time [min]	Area [mV *s]	Area %
1	3.421	2.158	0.027
2	3.597	3.052	0.038
3	3.718	13.573	0.168
4	3.867	6.857	0.085
5	3.986	4.730	0.058
6	4.068	0.815	0.010
7	4.206	4.795	0.059
8	4.322	4.834	0.060
9	4.526	38.756	0.478
10	4.607	30.729	0.379
11	4.835	7973.869	98.420
12	5.260	1.881	0.023
13	5.844	15.806	0.195

DAD1 B, Sig=254,8 Ref=550,60

Peak #	Ret. Time [min]	Area [mV *s]	Area %
1	3.381	2.239	0.035
2	3.480	0.062	0.001
3	3.510	0.136	0.002
4	3.718	11.482	0.178
5	3.867	4.837	0.075
6	3.995	3.303	0.051
7	4.212	3.663	0.057
8	4.321	4.073	0.063
9	4.525	30.096	0.466
10	4.608	23.136	0.358
11	4.835	6372.491	98.707
12	9.282	0.452	0.007

===

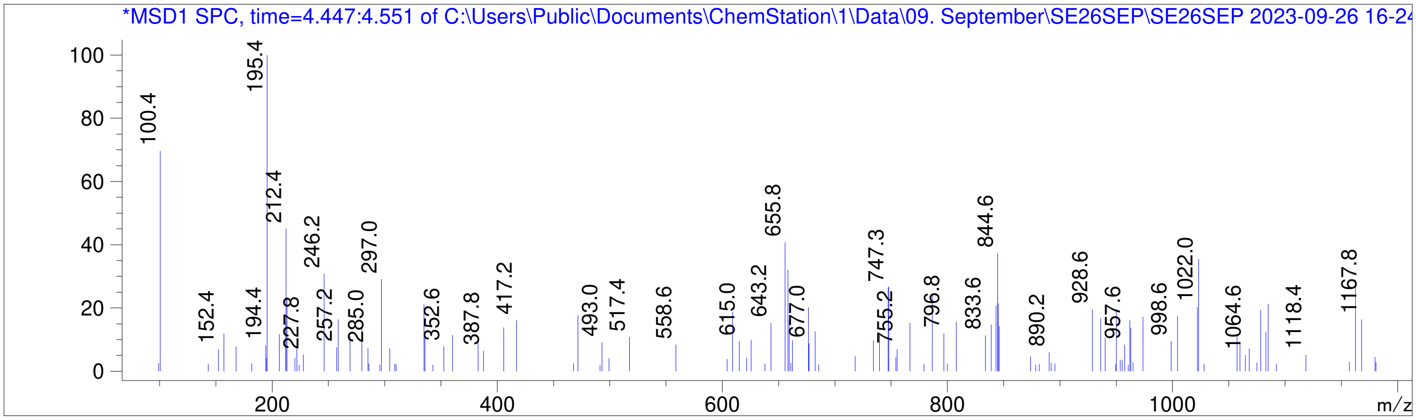
*MSD1 SPC, time=4.447:4.551 of C:\Users\Public\Documents\ChemStation\1\Data\09. September\SE26SEP\SE26SEP 2023-09-26 16-24
100.4
152.4
194.4
195.4
212.4
227.8
246.2
257.2
285.0
297.0
352.6
387.8
417.2
493.0
517.4
558.6
615.0
643.2
655.8
677.0
747.3
755.2
796.8
833.6
844.6
890.2
928.6
957.6
998.6
1022.0
1064.6
1118.4
1167.8
m/z

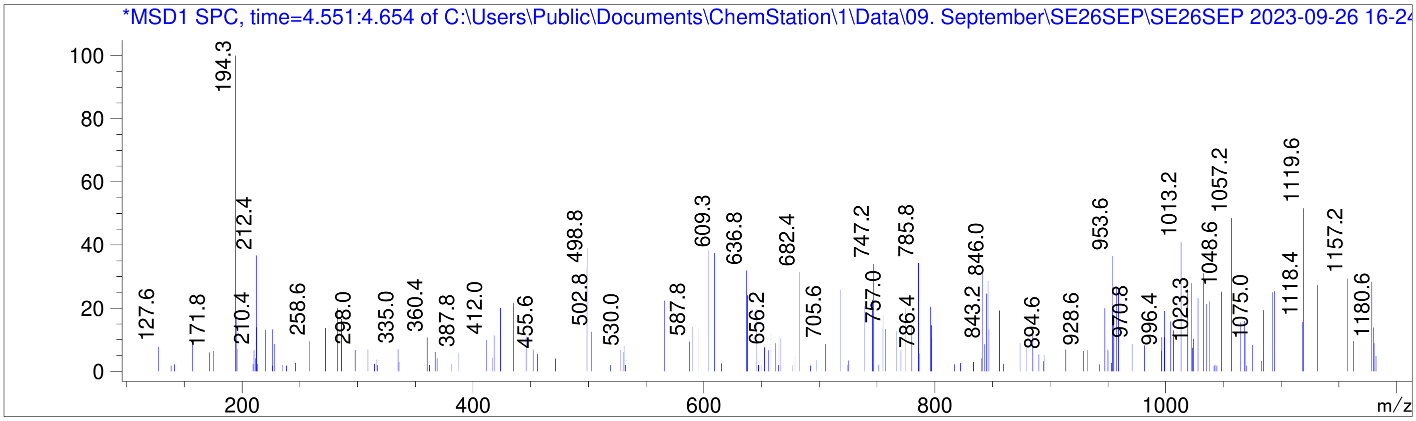
*MSD1 SPC, time=4.551:4.654 of C:\Users\Public\Documents\ChemStation\1\Data\09. September\SE26SEP\SE26SEP 2023-09-26 16-24
127.6
171.8
194.3
210.4
212.4
258.6
298.0
335.0
360.4
387.8
412.0
455.6
498.8
502.8
530.0
587.8
609.3
636.8
656.2
682.4
705.6
747.2
757.0
785.8
786.4
843.2
846.0
894.6
928.6
953.6
970.8
996.4
1013.2
1023.3
1048.6
1057.2
1075.0
1118.4
1119.6
1157.2
1180.6
m/z

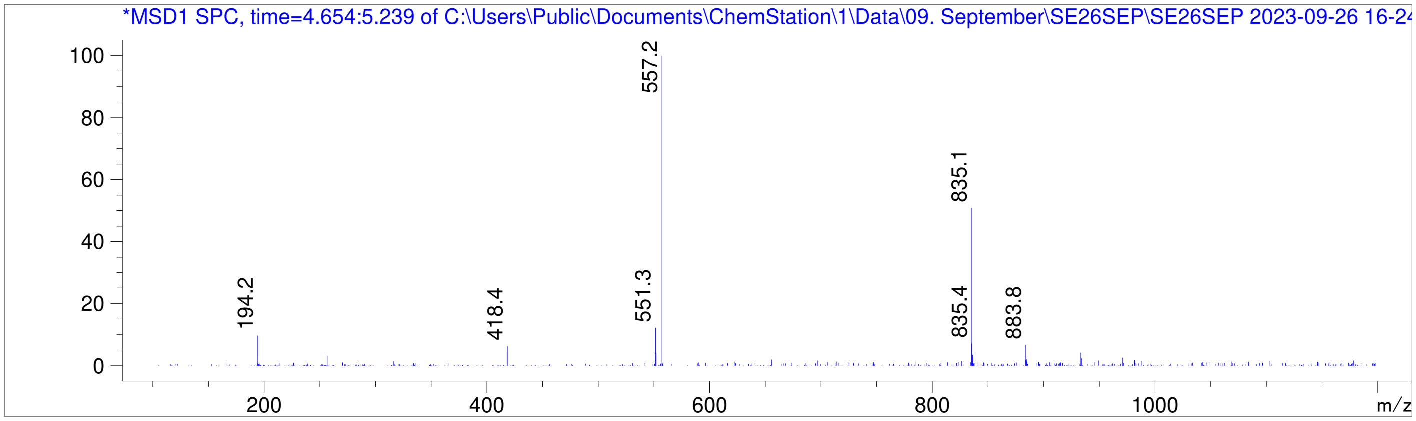
*MSD1 SPC, time=4.654:5.239 of C:\Users\Public\Documents\ChemStation\1\Data\09. September\SE26SEP\SE26SEP 2023-09-26 16-24
194.2
418.4
551.3
557.2
835.1
835.4
883.8
m/z
