# Supplementary material for: Astrobiological implications of the stability and reactivity of peptide nucleic acid (PNA) in concentrated sulfuric acid
Source: Sci Adv. 2025 Mar 26;11(13):eadr0006. doi: 10.1126/sciadv.adr0006 (PMC11939054; doi:10.1126/sciadv.adr0006)

Data -> C:\Users\Public\Documents\ChemStation\1\Data\Se03Oct\SE03OCT 2023-10-03 13-55-->
Sample-> CPT22010446-13-A3-24h

=====================================================================

| | | | | | |
|---|---|---|---|---|---|
| Injection Date | : | Tue, 3. Oct. 2023 | Seq Line | : | 4 |
| | | | Location | : | 60 |
| | | | Inj. Vol. | : | 2 µl |

Acq. Method : C:\Users\Public\Documents\ChemStation\1\Data\SE03OCT 2023-10-03 13-55-25\22010446 LCMS-6.M
Analysis Method : C:\Users\Public\Documents\ChemStation\1\Data\Se03Oct\SE03OCT 2023-10-03 13-55-25\22010446 LCMS-6.M (Sequence Method)
Waters XBridge Phenyl (4.6 * 150 mm; 3.5 µm); 0.05% TFA (aq) / AcN: 100/0 (0.0 min) --> (6.0 min) --> 70/30 (0.0 min) --> (2.0 min) --> 10/90 (2.0 min); Flow: 1.0 ml/min;
MSD1 = positive; MSD2 = negative

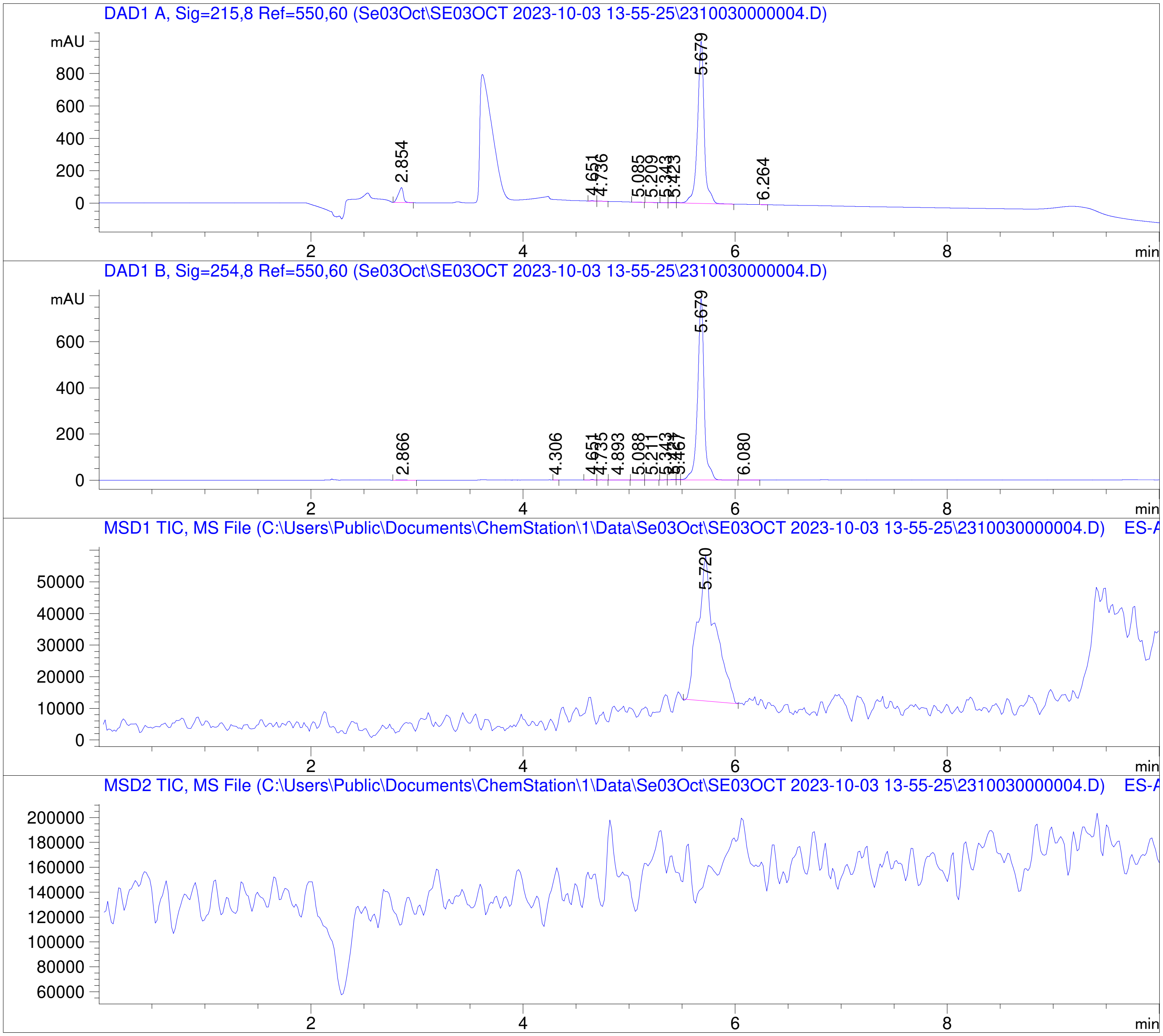

DAD1 A, Sig=215,8 Ref=550,60

| Peak # | Ret. Time [min] | Area [mV *s] | Area % |
|---|---|---|---|
| 1 | 2.854 | 307.529 | 6.458 |
| 2 | 4.651 | 5.518 | 0.116 |
| 3 | 4.736 | 2.669 | 0.056 |
| 4 | 5.085 | 1.722 | 0.036 |
| 5 | 5.209 | 1.700 | 0.036 |
| 6 | 5.343 | 4.176 | 0.088 |
| 7 | 5.423 | 13.315 | 0.280 |
| 8 | 5.679 | 4424.362 | 92.915 |
| 9 | 6.264 | 0.751 | 0.016 |

DAD1 B, Sig=254,8 Ref=550,60

| Peak # | Ret. Time [min] | Area [mV *s] | Area % |
|---|---|---|---|
| 1 | 2.866 | 2.728 | 0.080 |
| 2 | 4.306 | 0.570 | 0.017 |
| 3 | 4.651 | 5.406 | 0.158 |
| 4 | 4.735 | 2.136 | 0.063 |
| 5 | 4.893 | 1.419 | 0.042 |
| 6 | 5.088 | 1.878 | 0.055 |
| 7 | 5.211 | 1.794 | 0.053 |
| 8 | 5.343 | 2.332 | 0.068 |
| 9 | 5.421 | 9.392 | 0.275 |
| 10 | 5.467 | 6.247 | 0.183 |
| 11 | 5.679 | 3382.068 | 98.984 |
| 12 | 6.080 | 0.803 | 0.024 |

Data  -> C:\Users\Public\Documents\ChemStation\1\Data\Se03Oct\SE03OCT 2023-10-03 13-55-->
Sample-> CPT22010446-13-A3-24h

================================================================================

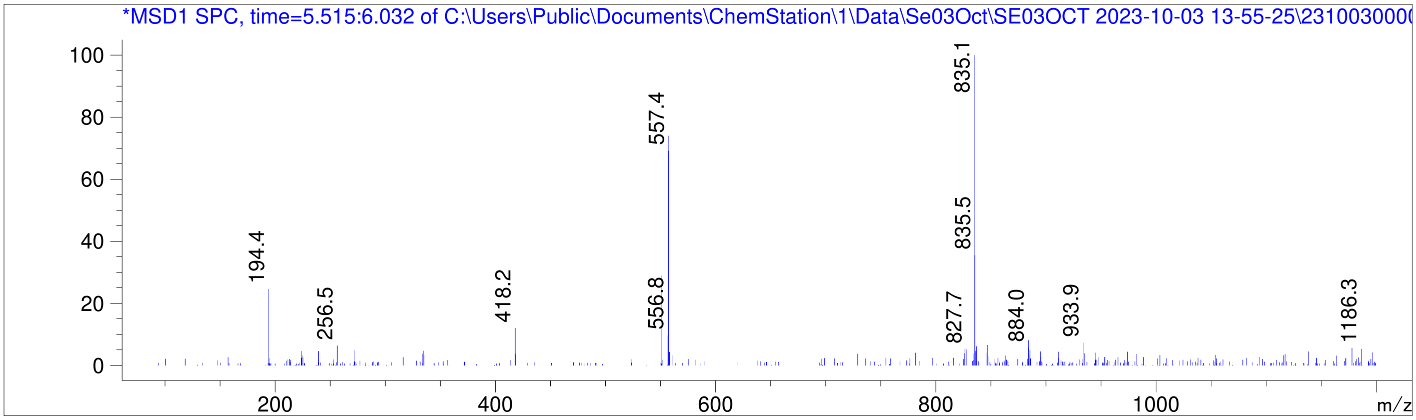

Supplement: Supplementary file 2 — Data S1 and S2 [file sciadv.adr0006_data_s1_and_s2.zip › Supplementary Dataset 1-LCMS DATA/LCMS PNA Hexamers A-T/LCMS A6 RT/24h/LCMS-6_CPT22010446-13-A3-24h.pdf]
