# Supplementary material for: Astrobiological implications of the stability and reactivity of peptide nucleic acid (PNA) in concentrated sulfuric acid
Source: Sci Adv. 2025 Mar 26;11(13):eadr0006. doi: 10.1126/sciadv.adr0006 (PMC11939054; doi:10.1126/sciadv.adr0006)
Supplement: Supplementary file 2 — Data S1 and S2 [file sciadv.adr0006_data_s1_and_s2.zip › Supplementary Dataset 1-LCMS DATA/LCMS PNA Hexamers A-T/LCMS C6 50C_80C/50C/1h/CPT22010446-21-C1-50deg-1h.pdf]

===

Injection Date : Tue, 17. Oct. 2023

Seq Line : 23
Location : 32
Inj. Vol. : 2 µl

Acq. Method : C:\Users\Public\Documents\ChemStation\1\Data\SE17OCT 2023-10-17 07-53-29\22010446 LCMS-6.M
Analysis Method : C:\Users\Public\Documents\ChemStation\1\Data\SE17OCT 2023-10-17 07-53-29\22010446 LCMS-6.M (Sequence Method)
Waters XBridge Phenyl (4.6 * 150 mm; 3.5 µm); 0.05% TFA (aq) / AcN: 100/0 (0.0 min) --> (6.0 min) --> 70/30 (0.0 min) --> (2.0 min) --> 10/90 (2.0 min); Flow: 1.0 ml/min;
MSD1 = positive; MSD2 = negative

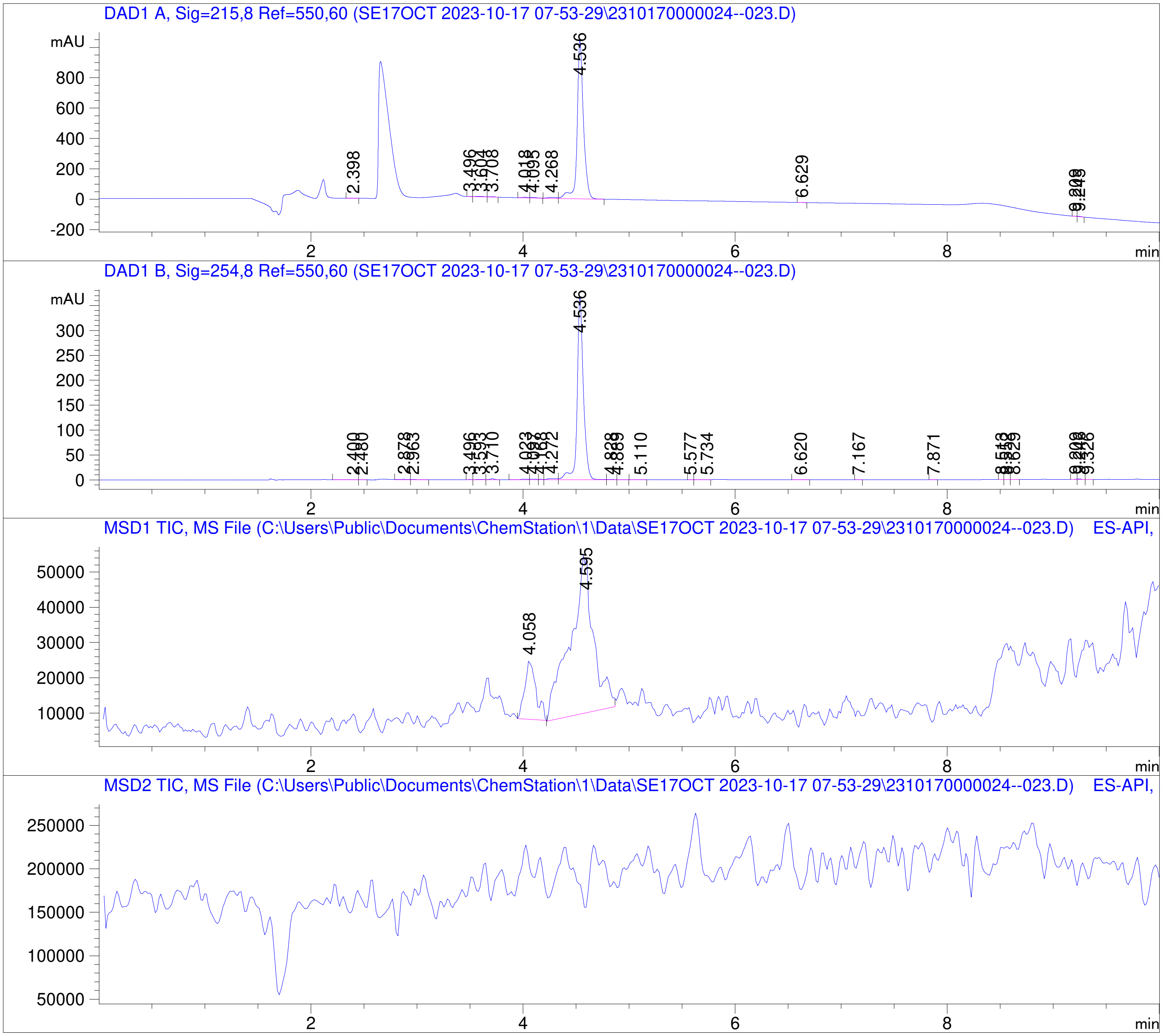

DAD1 A, Sig=215,8 Ref=550,60

Peak #	Ret. Time [min]	Area [mV *s]	Area %
1	2.398	1.556	0.035
2	3.496	2.151	0.049
3	3.604	14.482	0.328
4	3.708	10.824	0.245
5	4.018	15.832	0.359
6	4.095	14.537	0.329
7	4.268	34.234	0.776
8	4.536	4310.510	97.667
9	6.629	1.293	0.029
10	9.209	2.702	0.061
11	9.245	5.336	0.121

DAD1 B, Sig=254,8 Ref=550,60

Peak #	Ret. Time [min]	Area [mV *s]	Area %
1	2.400	1.970	0.126
2	2.480	0.619	0.040
3	2.878	7.343	0.469
4	2.963	2.995	0.191
5	3.496	0.722	0.046
6	3.593	3.157	0.202
7	3.710	5.757	0.368
8	4.023	5.851	0.374
9	4.097	5.134	0.328
10	4.168	2.259	0.144
11	4.272	14.630	0.935
12	4.536	1504.442	96.106
13	4.828	1.703	0.109
14	4.889	0.600	0.038
15	5.110	0.490	0.031
16	5.577	0.112	0.007
17	5.734	1.410	0.090
18	6.620	0.351	0.022
19	7.167	0.163	0.010
20	7.871	0.245	0.016
21	8.513	0.260	0.017
22	8.552	0.293	0.019
23	8.629	0.200	0.013
24	9.209	1.249	0.080
25	9.246	2.893	0.185
26	9.326	0.544	0.035

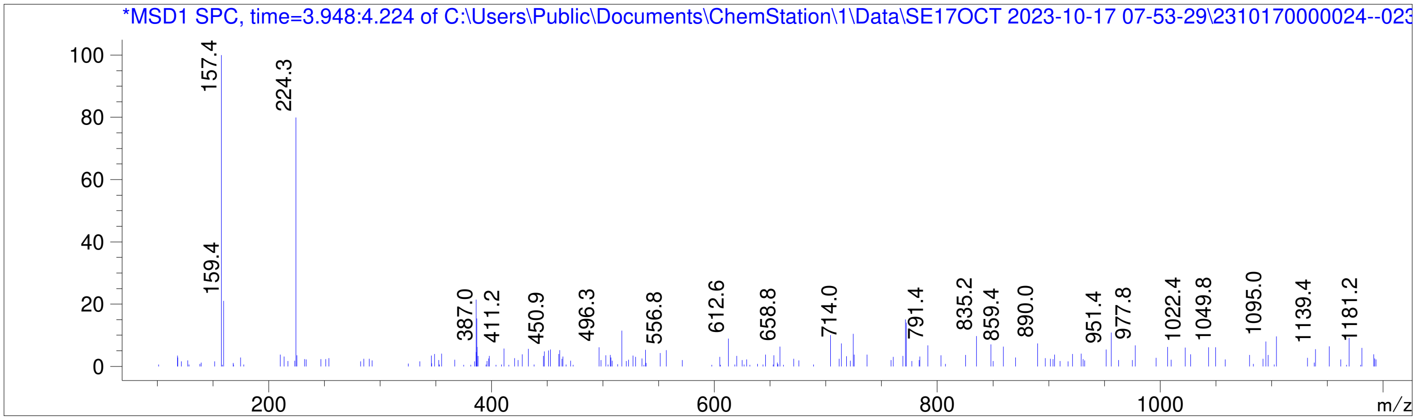
*MSD1 SPC, time=3.948:4.224 of C:\Users\Public\Documents\ChemStation\1\Data\SE17OCT 2023-10-17 07-53-29\2310170000024--023
157.4
224.3
159.4
387.0
411.2
450.9
496.3
556.8
612.6
658.8
714.0
791.4
835.2
859.4
890.0
951.4
977.8
1022.4
1049.8
1095.0
1139.4
1181.2
m/z

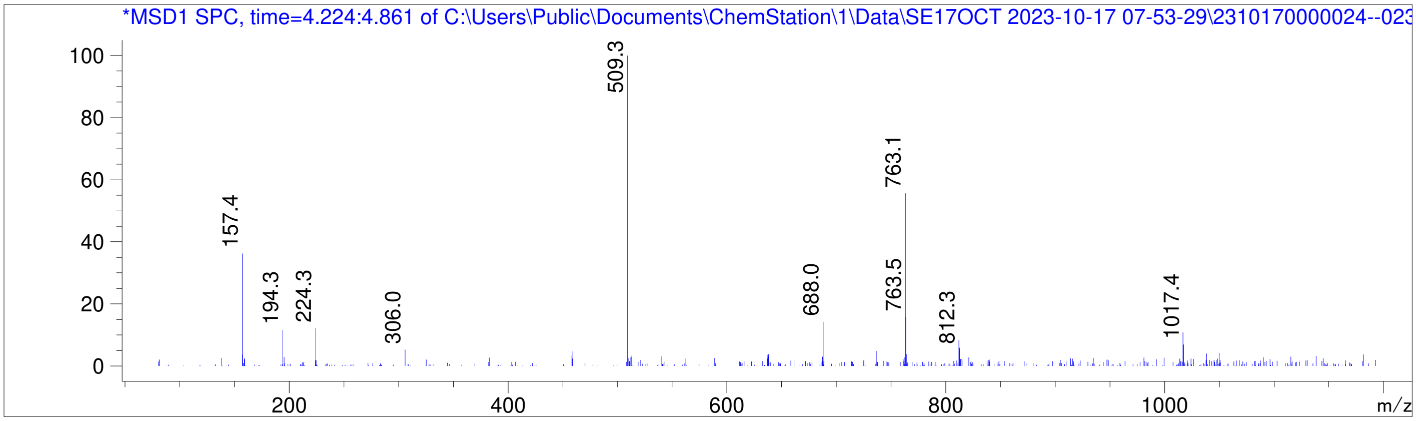
*MSD1 SPC, time=4.224:4.861 of C:\Users\Public\Documents\ChemStation\1\Data\SE17OCT 2023-10-17 07-53-29\2310170000024--023
509.3
763.1
157.4
194.3
224.3
306.0
688.0
763.5
812.3
1017.4
m/z
