# Supplementary material for: Astrobiological implications of the stability and reactivity of peptide nucleic acid (PNA) in concentrated sulfuric acid
Source: Sci Adv. 2025 Mar 26;11(13):eadr0006. doi: 10.1126/sciadv.adr0006 (PMC11939054; doi:10.1126/sciadv.adr0006)

Data -> C:\Users\Public\Documents\ChemStation\1\Data\SE01NOV 2023-11-01 15-33-53\ ->
Sample-> CPT22010446-21-C2-50deg-1h

===

Injection Date : Thu, 2. Nov. 2023 Seq Line : 50
Location : 20
Inj. Vol. : 2 µl

Acq. Method : C:\Users\Public\Documents\ChemStation\1\Data\SE01NOV 2023-11-01 15-33-53\22010446 LCMS-6.M
Analysis Method : C:\Users\Public\Documents\ChemStation\1\Data\SE01NOV 2023-11-01 15-33-53\22010446 LCMS-6.M (Sequence Method)
Waters XBridge Phenyl (4.6 * 150 mm; 3.5 µm); 0.05% TFA (aq) / AcN: 100/0 (0.0 min) --> (6.0 min) --> 70/30 (0.0 min) --> (2.0 min) --> 10/90 (2.0 min); Flow: 1.0 ml/min;
MSD1 = positive; MSD2 = negative

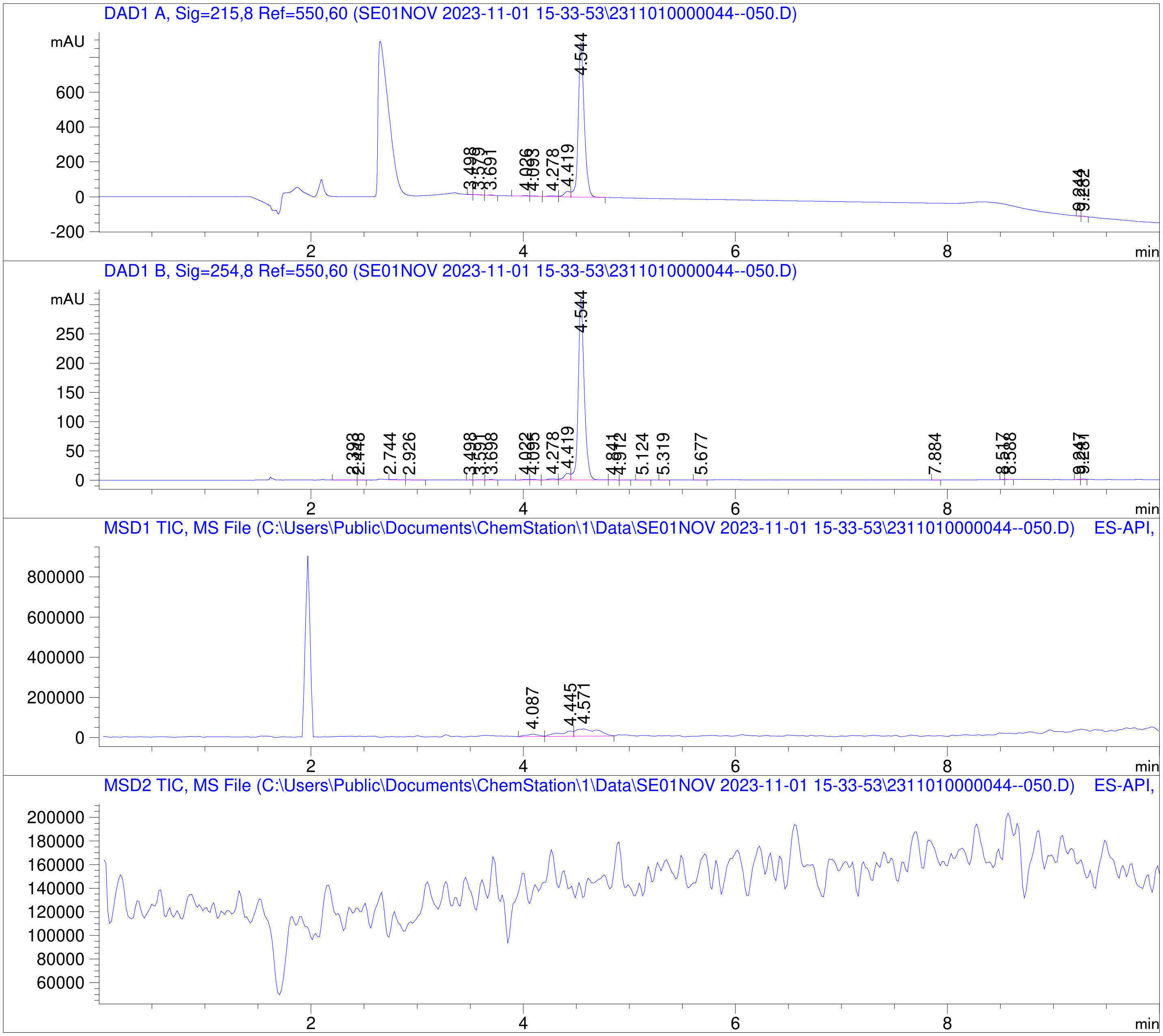

DAD1 A, Sig=215,8 Ref=550,60

Peak #	Ret. Time [min]	Area [mV *s]	Area %
1	3.498	1.493	0.040
2	3.579	5.163	0.140
3	3.691	9.223	0.250
4	4.026	13.225	0.358
5	4.093	9.958	0.270
6	4.278	25.658	0.695
7	4.419	128.916	3.490
8	4.544	3494.878	94.600
9	9.244	1.758	0.048
10	9.282	4.086	0.111

DAD1 B, Sig=254,8 Ref=550,60

Peak #	Ret. Time [min]	Area [mV *s]	Area %
1	2.393	1.496	0.114
2	2.448	0.394	0.030
3	2.744	7.664	0.585
4	2.926	2.214	0.169
5	3.498	0.569	0.043
6	3.591	2.504	0.191
7	3.698	3.102	0.237
8	4.022	4.275	0.326
9	4.095	3.482	0.266
10	4.278	10.142	0.774
11	4.419	46.515	3.551
12	4.544	1220.076	93.153
13	4.841	1.576	0.120
14	4.912	0.512	0.039
15	5.124	0.484	0.037
16	5.319	0.145	0.011
17	5.677	0.197	0.015
18	7.884	0.146	0.011
19	8.517	0.139	0.011
20	8.588	0.217	0.017
21	9.247	0.931	0.071
22	9.281	2.975	0.227

*MSD1 SPC, time=3.948:4.207 of C:\Users\Public\Documents\ChemStation\1\Data\SE01NOV 2023-11-01 15-33-53\2311010000044--05(

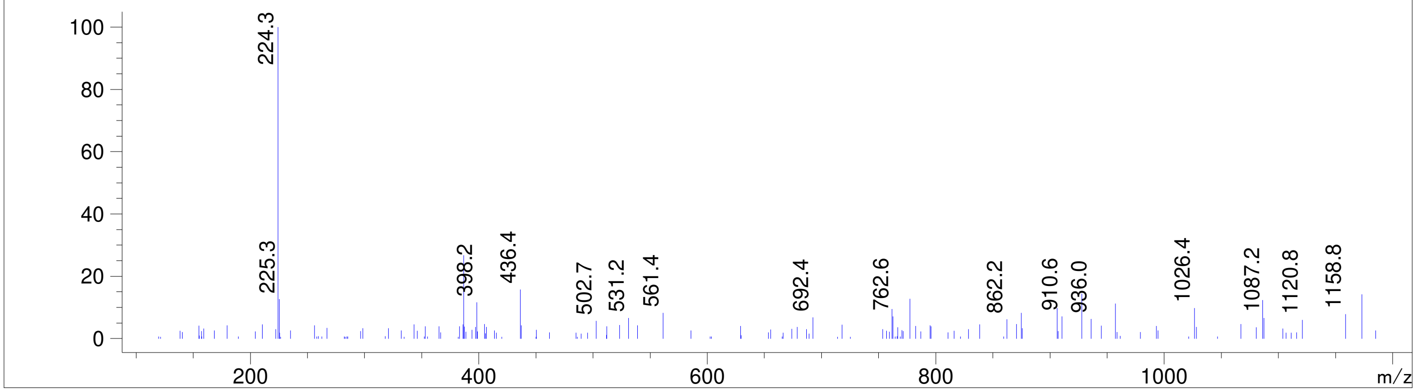

*MSD1 SPC, time=4.207:4.482 of C:\Users\Public\Documents\ChemStation\1\Data\SE01NOV 2023-11-01 15-33-53\2311010000044--05(

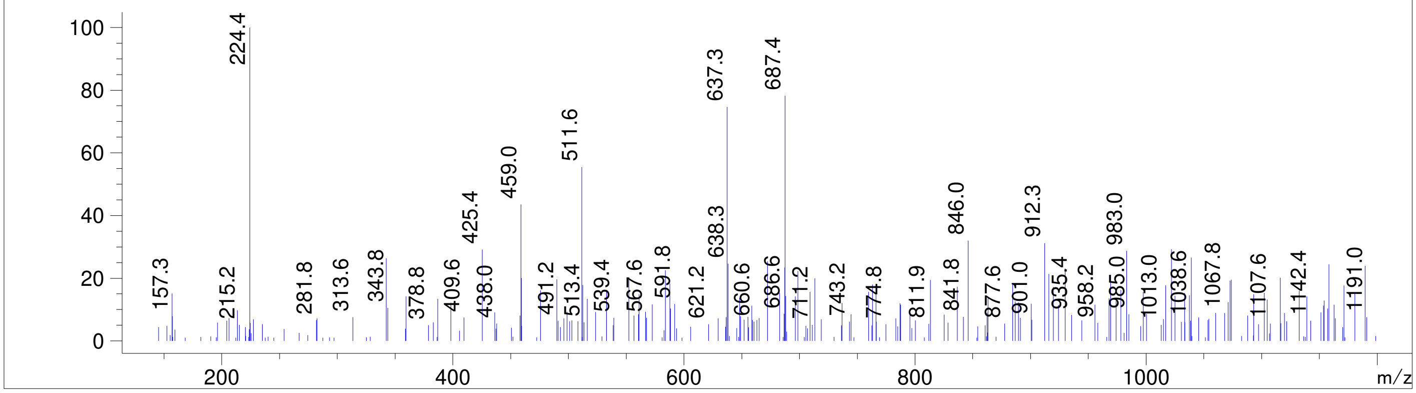

*MSD1 SPC, time=4.482:4.861 of C:\Users\Public\Documents\ChemStation\1\Data\SE01NOV 2023-11-01 15-33-53\2311010000044--05(

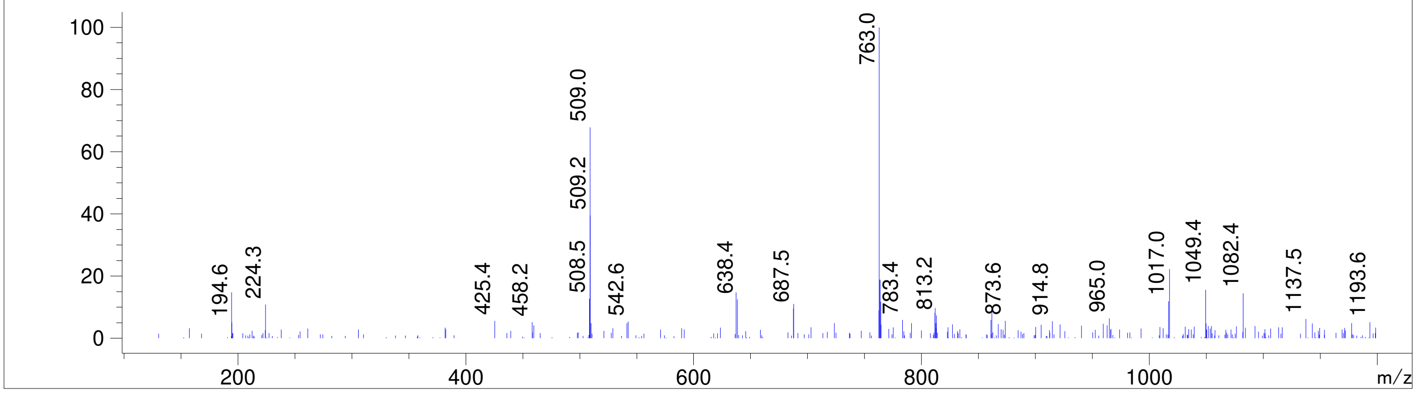

Supplement: Supplementary file 2 — Data S1 and S2 [file sciadv.adr0006_data_s1_and_s2.zip › Supplementary Dataset 1-LCMS DATA/LCMS PNA Hexamers A-T/LCMS C6 50C_80C/50C/1h/CPT22010446-21-C2-50deg-1h.pdf]
