# Supplementary material for: Astrobiological implications of the stability and reactivity of peptide nucleic acid (PNA) in concentrated sulfuric acid
Source: Sci Adv. 2025 Mar 26;11(13):eadr0006. doi: 10.1126/sciadv.adr0006 (PMC11939054; doi:10.1126/sciadv.adr0006)
Supplement: Supplementary file 2 — Data S1 and S2 [file sciadv.adr0006_data_s1_and_s2.zip › Supplementary Dataset 1-LCMS DATA/LCMS PNA Hexamers A-T/LCMS C6 50C_80C/50C/24h/CPT22010446-21-C1-50deg-24h.pdf]

Data -> C:\Users\Public\Documents\ChemStation\1\Data\SE17OCT 2023-10-17 16-51-41\ ->
Sample-> CPT22010446-21-C1-50deg-1h

Injection Date :	Tue, 17. Oct. 2023	Seq Line :	8
		Location :	52
		Inj. Vol. :	2 µl

Acq. Method : C:\Users\Public\Documents\ChemStation\1\Data\SE17OCT 2023-10-17 16-51-41\22010446 LCMS-6.M
Analysis Method : C:\Users\Public\Documents\ChemStation\1\Data\SE17OCT 2023-10-17 16-51-41\22010446 LCMS-6.M (Sequence Method)
Waters XBridge Phenyl (4.6 * 150 mm; 3.5 µm); 0.05% TFA (aq) / AcN: 100/0 (0.0 min) --> (6.0 min) --> 70/30 (0.0 min) --> (2.0 min) --> 10/90 (2.0 min); Flow: 1.0 ml/min;
MSD1 = positive; MSD2 = negative

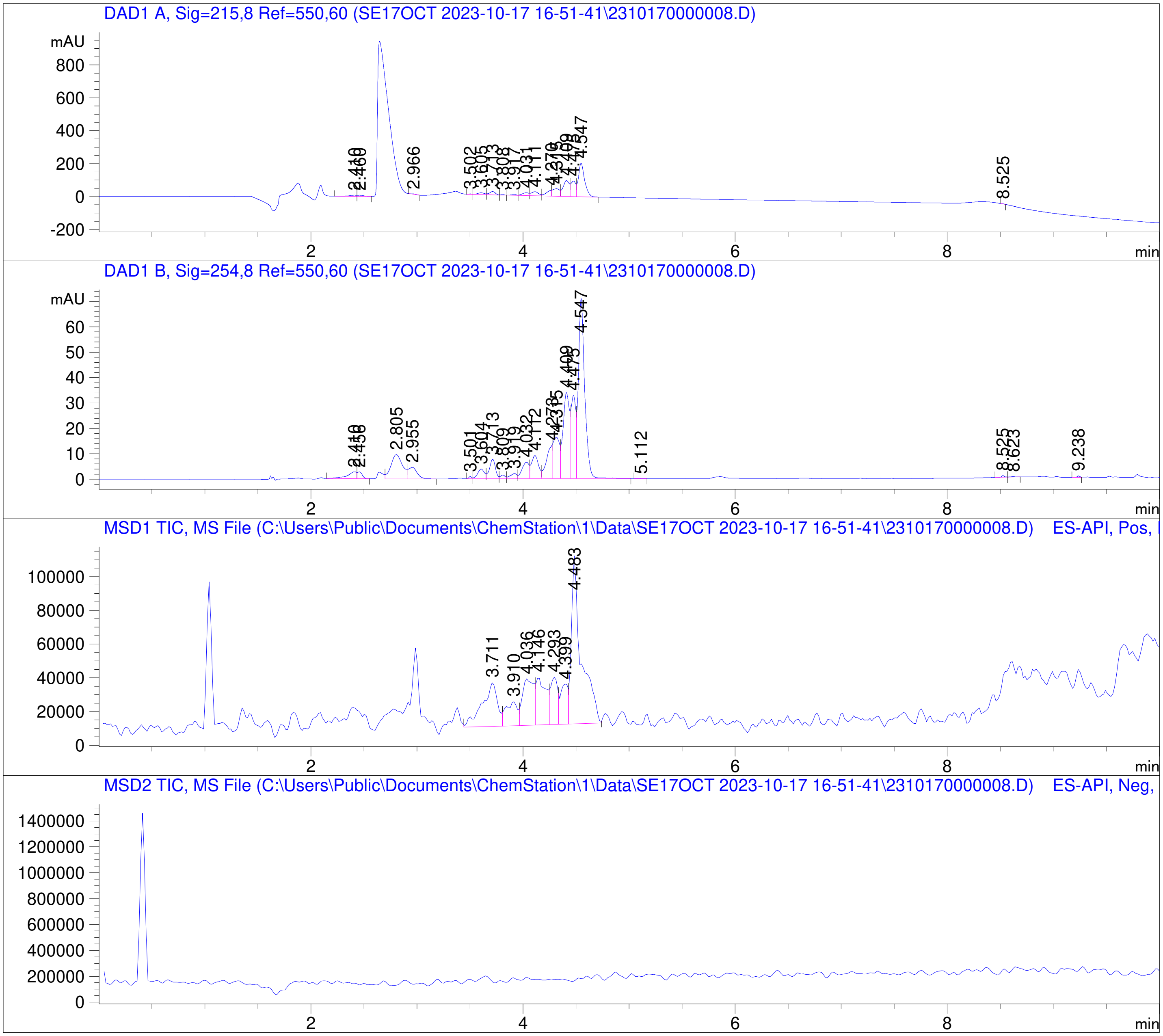

DAD1 A, Sig=215,8 Ref=550,60

Peak #	Ret. Time [min]	Area [mV *s]	Area %
1	2.410	34.176	1.498
2	2.460	26.610	1.166
3	2.966	8.756	0.384
4	3.502	4.537	0.199
5	3.605	49.545	2.171
6	3.713	77.346	3.389
7	3.808	6.351	0.278
8	3.917	15.849	0.694
9	4.031	76.715	3.361
10	4.111	116.234	5.093
11	4.270	119.376	5.231
12	4.315	207.576	9.095
13	4.409	390.683	17.119
14	4.475	308.568	13.521
15	4.547	838.023	36.720
16	8.525	1.865	0.082

DAD1 B, Sig=254,8 Ref=550,60

Peak #	Ret. Time [min]	Area [mV *s]	Area %
1	2.410	16.722	1.825
2	2.456	8.425	0.920
3	2.805	71.956	7.854
4	2.955	26.965	2.943
5	3.501	1.301	0.142
6	3.604	16.097	1.757
7	3.713	29.537	3.224
8	3.809	4.211	0.460
9	3.919	8.405	0.917
10	4.032	29.904	3.264
11	4.112	42.509	4.640
12	4.273	47.085	5.139
13	4.315	70.413	7.685
14	4.409	139.024	15.174
15	4.475	106.674	11.643
16	4.547	294.232	32.114
17	5.112	0.141	0.015
18	8.525	1.060	0.116
19	8.623	0.368	0.040
20	9.238	1.167	0.127

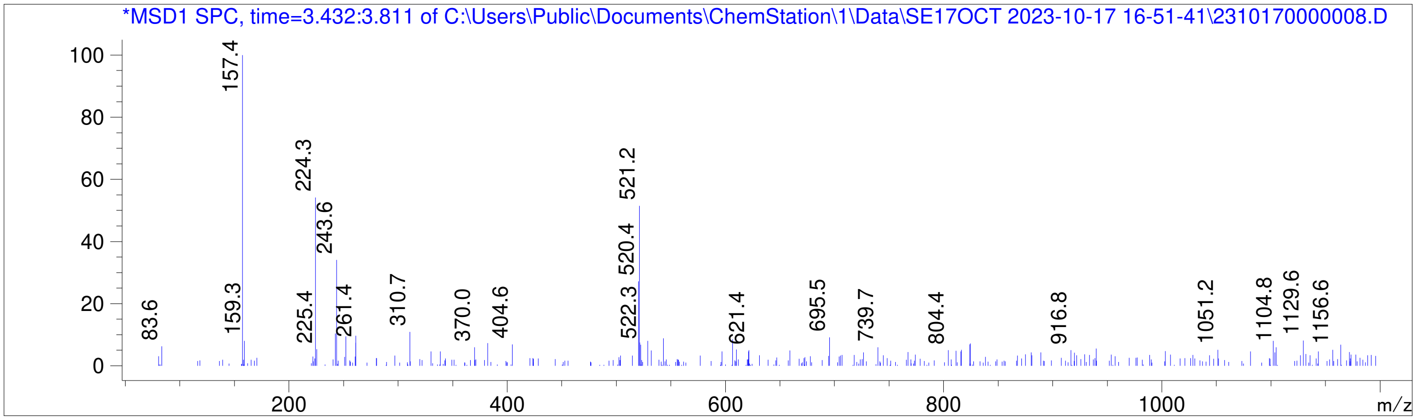
*MSD1 SPC, time=3.432:3.811 of C:\Users\Public\Documents\ChemStation\1\Data\SE17OCT 2023-10-17 16-51-41\2310170000008.D
157.4
224.3
521.2
243.6
520.4
83.6
159.3
225.4
261.4
310.7
370.0
404.6
522.3
621.4
695.5
739.7
804.4
916.8
1051.2
1104.8
1129.6
1156.6
m/z

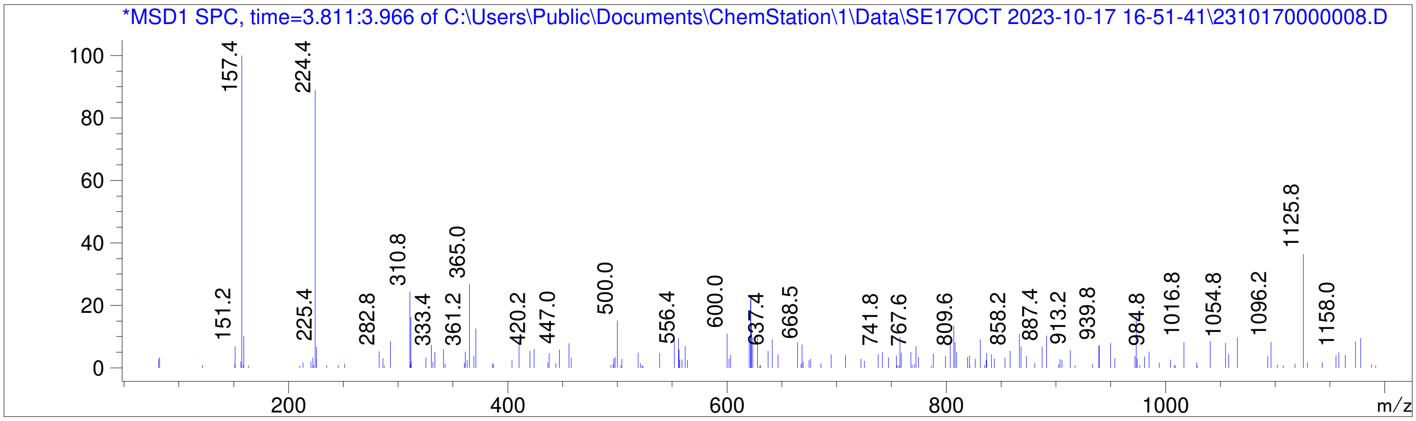
*MSD1 SPC, time=3.811:3.966 of C:\Users\Public\Documents\ChemStation\1\Data\SE17OCT 2023-10-17 16-51-41\2310170000008.D
157.4
224.4
1125.8
310.8
365.0
151.2
225.4
282.8
333.4
361.2
420.2
447.0
500.0
556.4
600.0
637.4
668.5
741.8
767.6
809.6
858.2
887.4
913.2
939.8
984.8
1016.8
1054.8
1096.2
1158.0
m/z

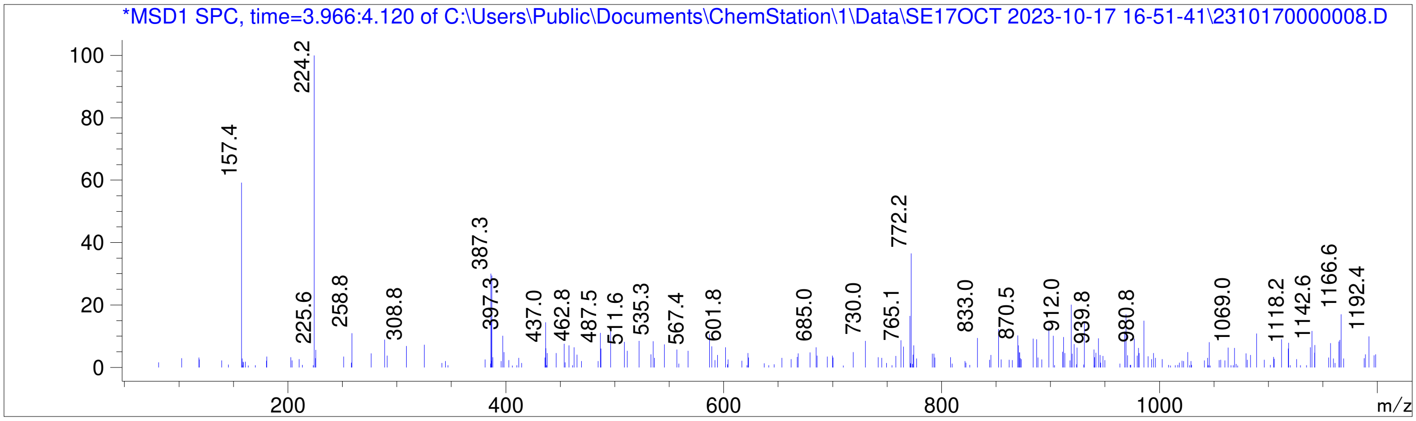
*MSD1 SPC, time=3.966:4.120 of C:\Users\Public\Documents\ChemStation\1\Data\SE17OCT 2023-10-17 16-51-41\2310170000008.D
224.2
157.4
772.2
387.3
225.6
258.8
308.8
397.3
437.0
462.8
487.5
511.6
535.3
567.4
601.8
685.0
730.0
765.1
833.0
870.5
912.0
939.8
980.8
1069.0
1118.2
1142.6
1166.6
1192.4
m/z

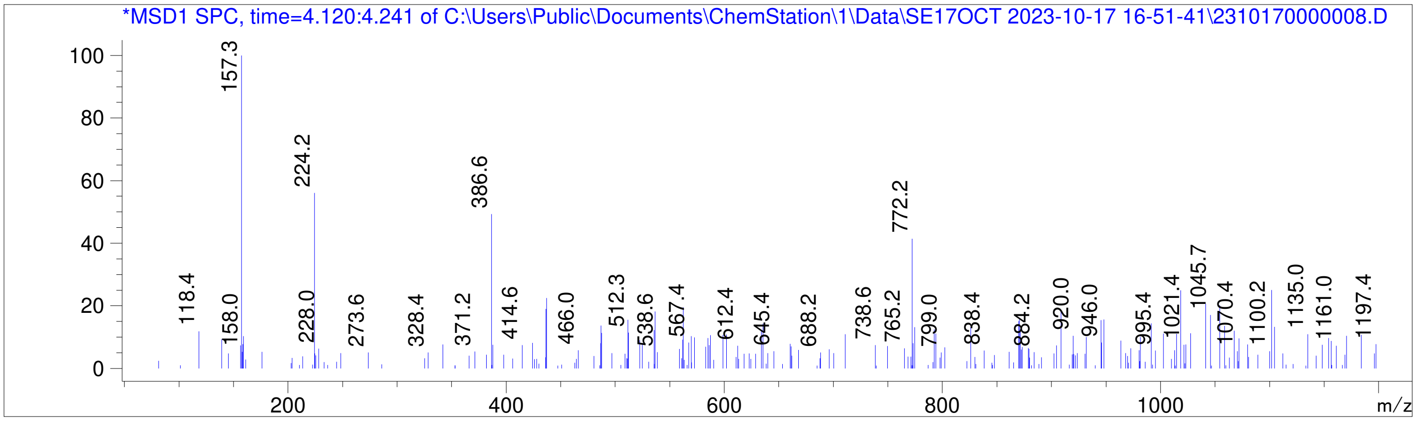
*MSD1 SPC, time=4.120:4.241 of C:\Users\Public\Documents\ChemStation\1\Data\SE17OCT 2023-10-17 16-51-41\2310170000008.D
157.3
224.2
386.6
772.2
118.4
158.0
228.0
273.6
328.4
371.2
414.6
466.0
512.3
538.6
567.4
612.4
645.4
688.2
738.6
765.2
799.0
838.4
884.2
920.0
946.0
995.4
1021.4
1045.7
1070.4
1100.2
1135.0
1161.0
1197.4
m/z

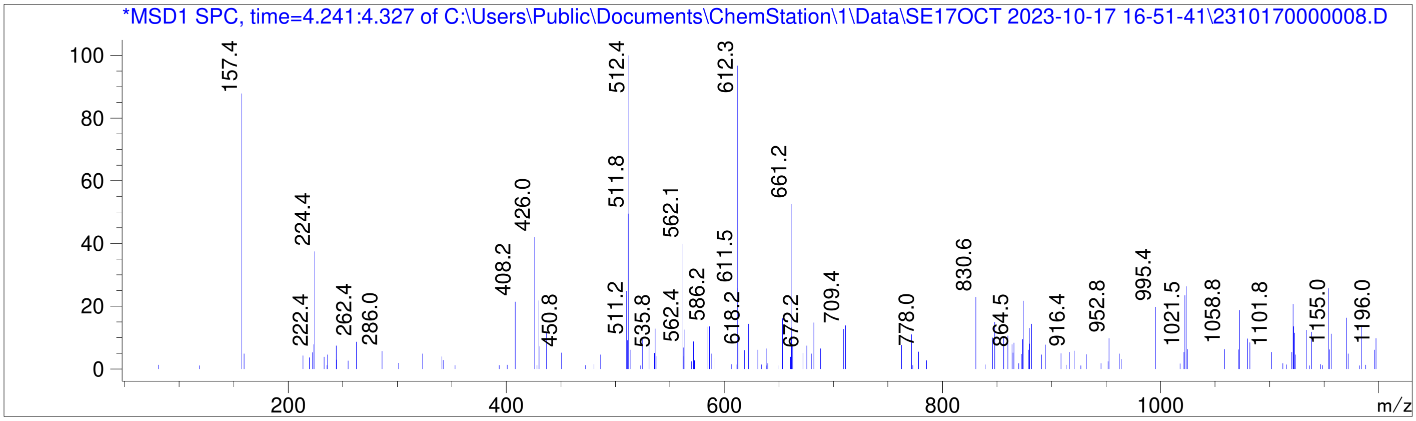
*MSD1 SPC, time=4.241:4.327 of C:\Users\Public\Documents\ChemStation\1\Data\SE17OCT 2023-10-17 16-51-41\2310170000008.D
157.4
512.4
612.3
511.8
661.2
562.1
224.4
426.0
408.2
611.5
830.6
995.4
586.2
709.4
222.4
262.4
286.0
450.8
511.2
535.8
562.4
618.2
672.2
778.0
864.5
916.4
952.8
1021.5
1058.8
1101.8
1155.0
1196.0
m/z

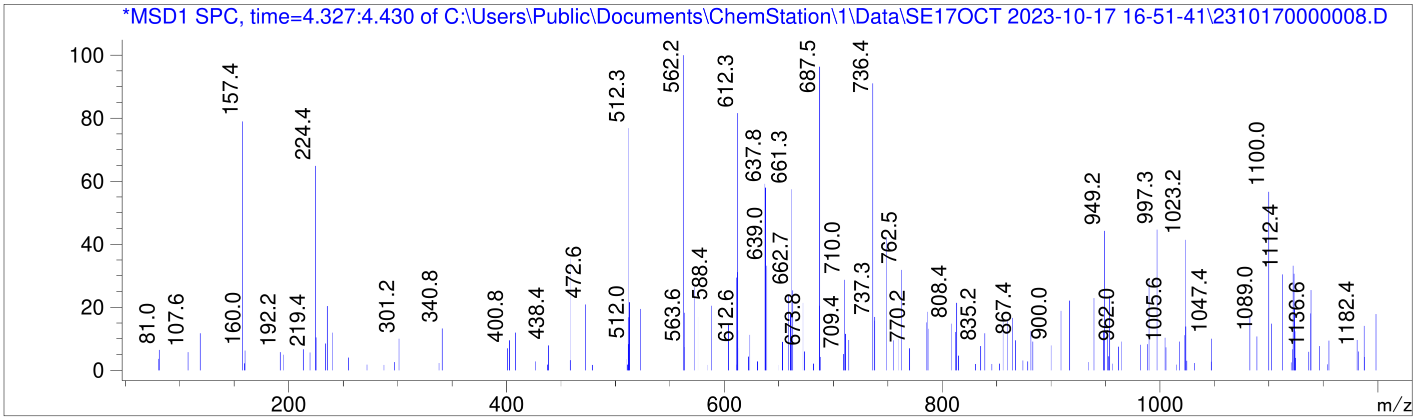
*MSD1 SPC, time=4.327:4.430 of C:\Users\Public\Documents\ChemStation\1\Data\SE17OCT 2023-10-17 16-51-41\2310170000008.D
562.2
687.5
736.4
157.4
512.3
612.3
224.4
637.8
661.3
1100.0
949.2
997.3
1023.2
639.0
762.5
710.0
1112.4
662.7
472.6
588.4
737.3
340.8
808.4
1047.4
81.0
107.6
160.0
192.2
219.4
301.2
400.8
438.4
512.0
563.6
612.6
673.8
709.4
770.2
835.2
867.4
900.0
962.0
1005.6
1089.0
1136.6
1182.4
m/z

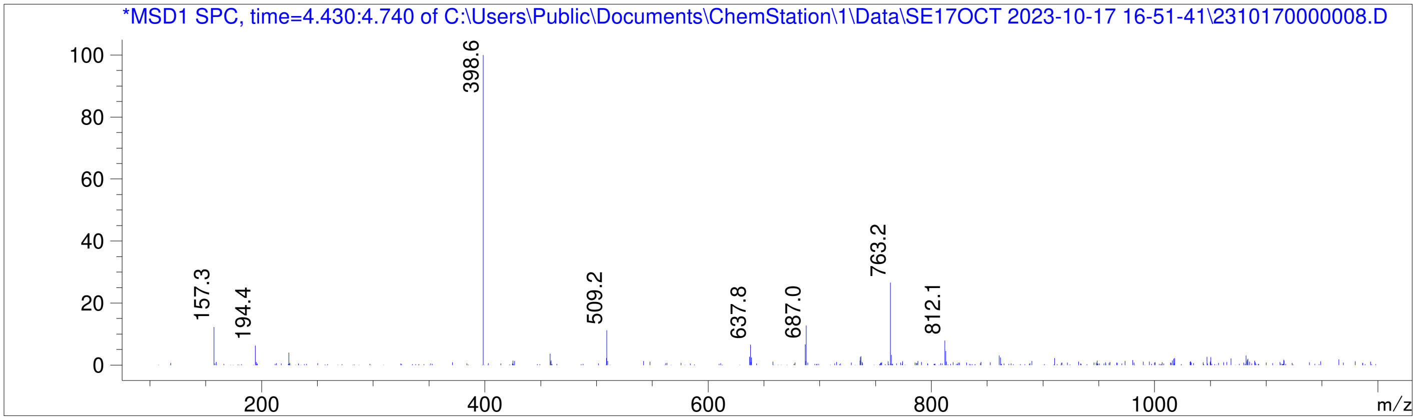
*MSD1 SPC, time=4.430:4.740 of C:\Users\Public\Documents\ChemStation\1\Data\SE17OCT 2023-10-17 16-51-41\2310170000008.D
398.6
763.2
157.3
194.4
509.2
637.8
687.0
812.1
m/z
