# Supplementary material for: Astrobiological implications of the stability and reactivity of peptide nucleic acid (PNA) in concentrated sulfuric acid
Source: Sci Adv. 2025 Mar 26;11(13):eadr0006. doi: 10.1126/sciadv.adr0006 (PMC11939054; doi:10.1126/sciadv.adr0006)
Supplement: Supplementary file 2 — Data S1 and S2 [file sciadv.adr0006_data_s1_and_s2.zip › Supplementary Dataset 1-LCMS DATA/LCMS PNA Hexamers A-T/LCMS C6 50C_80C/50C/24h/CPT22010446-21-C2-50deg-24h.pdf]

Sample-> CPT22010446-21-C2-50deg-24h

===

Injection Date : Thu, 2. Nov. 2023

Seq Line : 15
Location : 16
Inj. Vol. : 2 µl

Acq. Method : C:\Users\Public\Documents\ChemStation\1\Data\SE02NOV 2023-11-02 14-31-42\22010446 LCMS-6.M
Analysis Method : C:\Users\Public\Documents\ChemStation\1\Data\SE02NOV 2023-11-02 14-31-42\22010446 LCMS-6.M (Sequence Method)
Waters XBridge Phenyl (4.6 * 150 mm; 3.5 µm); 0.05% TFA (aq) / AcN: 100/0 (0.0 min) --> (6.0 min) --> 70/30 (0.0 min) --> (2.0 min) --> 10/90 (2.0 min); Flow: 1.0 ml/min;
MSD1 = positive; MSD2 = negative

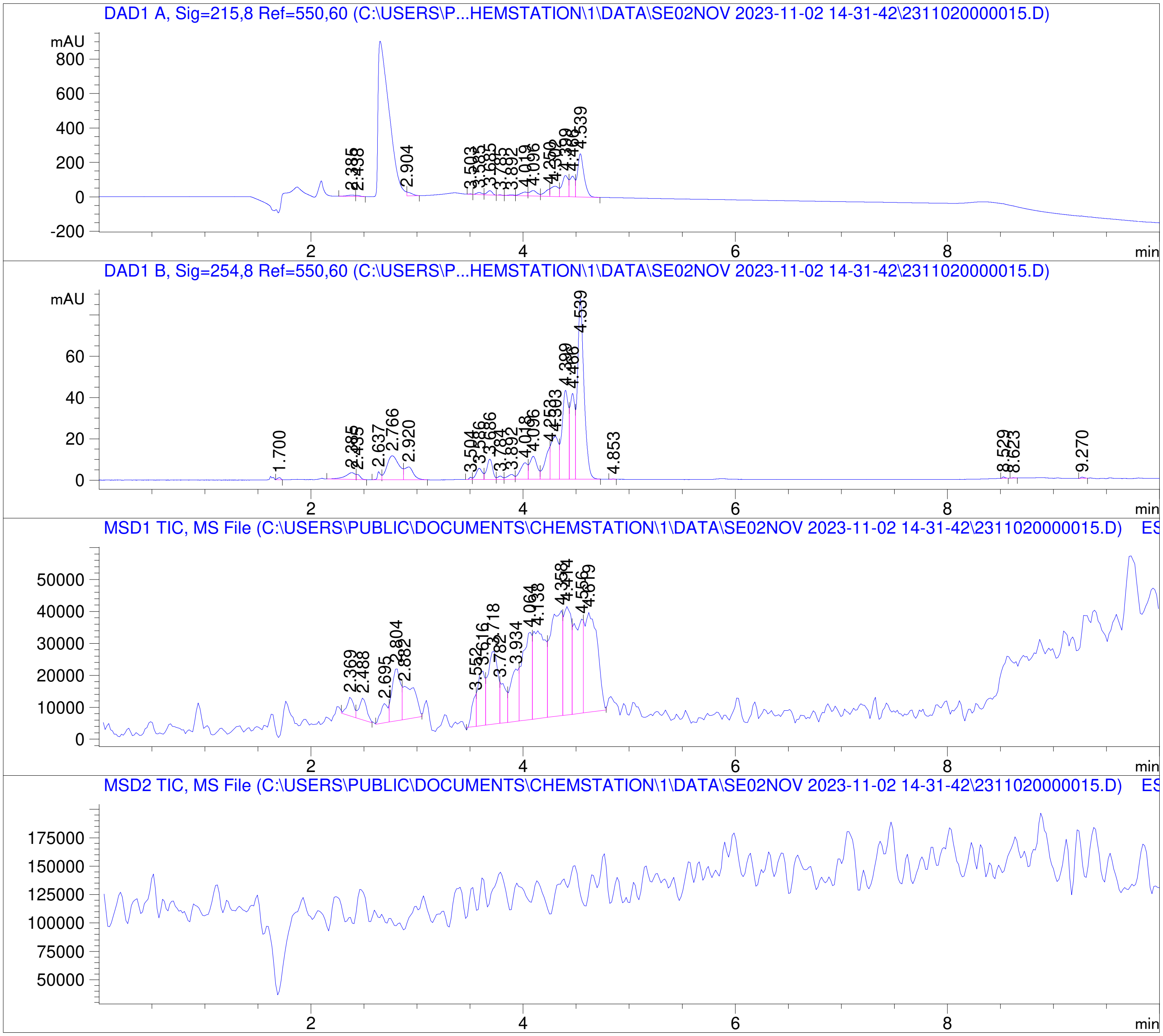

Sample-> CPT22010446-21-C2-50deg-24h

DAD1 A, Sig=215,8 Ref=550,60

Peak #	Ret. Time [min]	Area [mV *s]	Area %
1	2.385	39.350	1.335
2	2.438	17.008	0.577
3	2.904	66.345	2.250
4	3.503	4.011	0.136
5	3.585	43.594	1.479
6	3.685	85.938	2.915
7	3.785	5.701	0.193
8	3.892	17.829	0.605
9	4.019	99.924	3.389
10	4.096	156.961	5.324
11	4.250	140.224	4.756
12	4.302	291.750	9.896
13	4.399	506.406	17.176
14	4.466	396.463	13.447
15	4.539	1076.811	36.523

DAD1 B, Sig=254,8 Ref=550,60

Peak #	Ret. Time [min]	Area [mV *s]	Area %
1	1.700	2.184	0.185
2	2.385	21.514	1.823
3	2.435	6.399	0.542
4	2.637	8.320	0.705
5	2.766	93.191	7.897
6	2.920	35.654	3.021
7	3.504	1.832	0.155
8	3.586	21.611	1.831
9	3.686	37.137	3.147
10	3.784	5.071	0.430
11	3.892	10.394	0.881
12	4.018	38.711	3.281
13	4.096	54.530	4.621
14	4.253	51.107	4.331
15	4.303	99.961	8.471
16	4.399	183.592	15.558
17	4.466	131.802	11.169
18	4.539	374.003	31.695
19	4.853	0.243	0.021
20	8.529	1.146	0.097
21	8.623	0.285	0.024
22	9.270	1.335	0.113

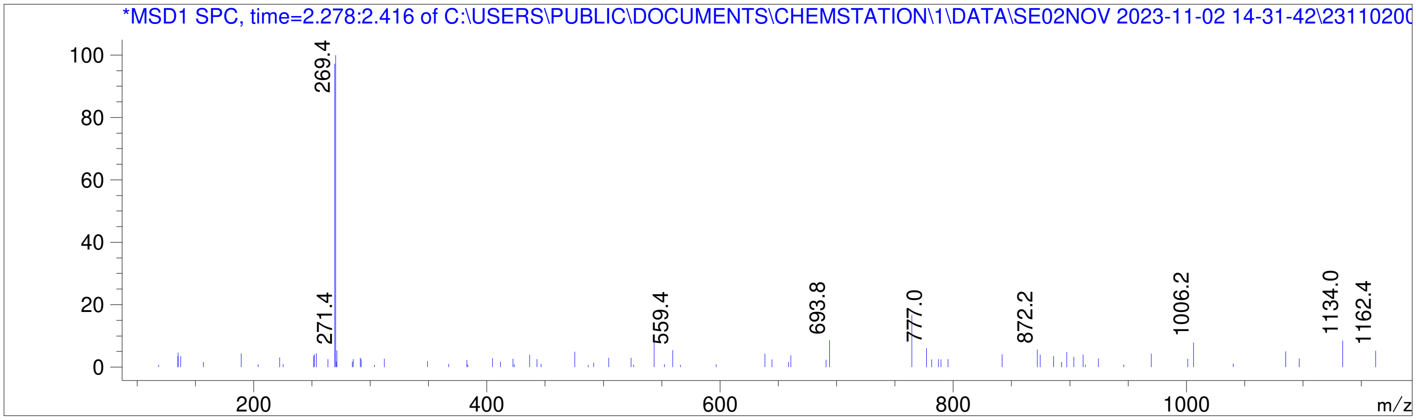
*MSD1 SPC, time=2.278:2.416 of C:\USERS\PUBLIC\DOCUMENTS\CHEMSTATION\1\DATA\SE02NOV 2023-11-02 14-31-42\2311020
269.4
271.4
559.4
693.8
777.0
872.2
1006.2
1134.0
1162.4
m/z

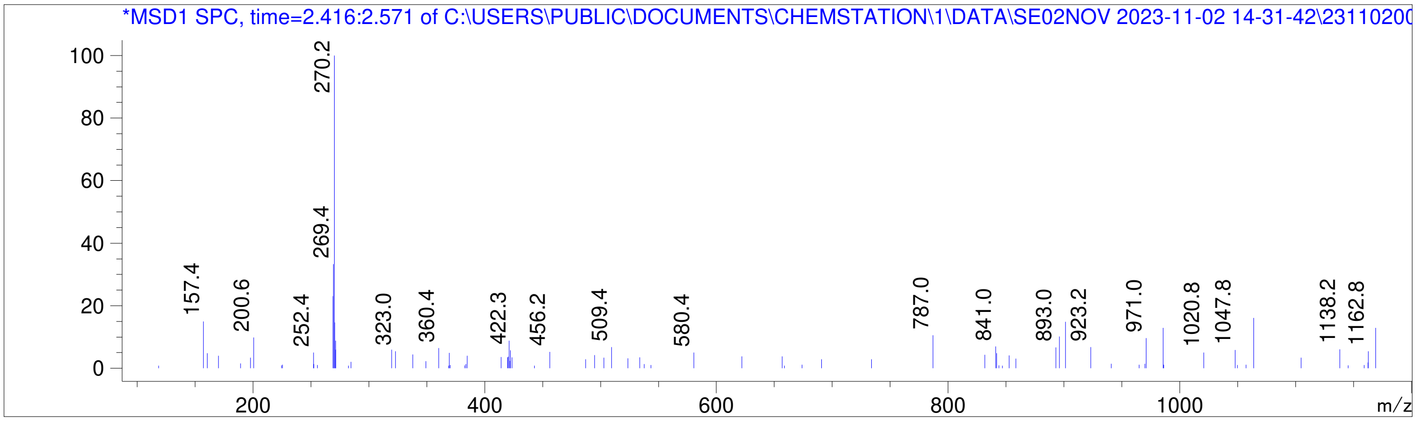
*MSD1 SPC, time=2.416:2.571 of C:\USERS\PUBLIC\DOCUMENTS\CHEMSTATION\1\DATA\SE02NOV 2023-11-02 14-31-42\2311020
270.2
269.4
157.4
200.6
252.4
323.0
360.4
422.3
456.2
509.4
580.4
787.0
841.0
893.0
923.2
971.0
1020.8
1047.8
1138.2
1162.8
m/z

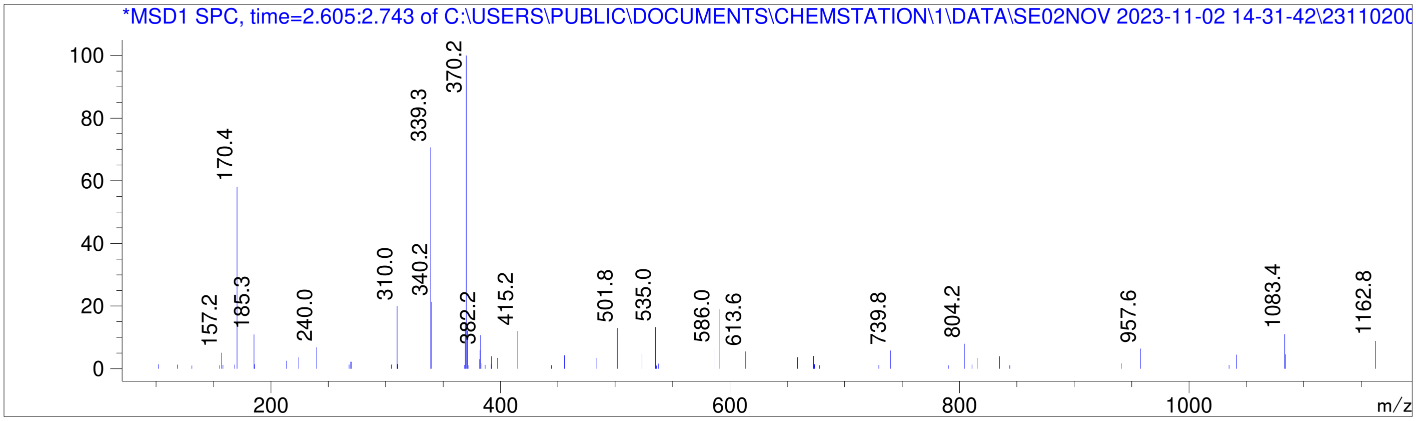
*MSD1 SPC, time=2.605:2.743 of C:\USERS\PUBLIC\DOCUMENTS\CHEMSTATION\1\DATA\SE02NOV 2023-11-02 14-31-42\2311020
370.2
339.3
170.4
157.2
185.3
240.0
310.0
340.2
382.2
415.2
501.8
535.0
586.0
613.6
739.8
804.2
957.6
1083.4
1162.8
m/z

Sample-> CPT22010446-21-C2-50deg-24h

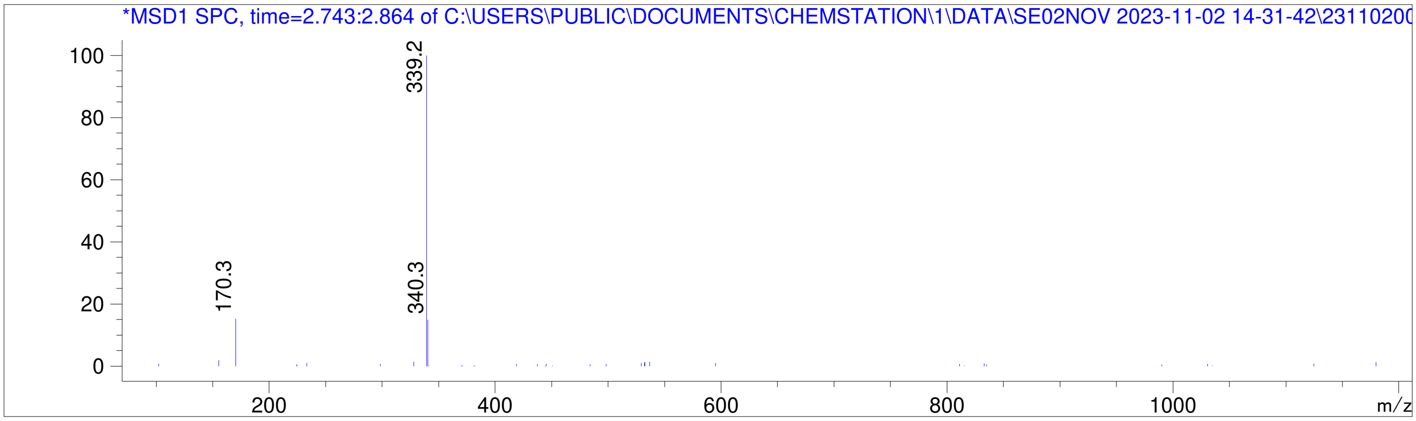

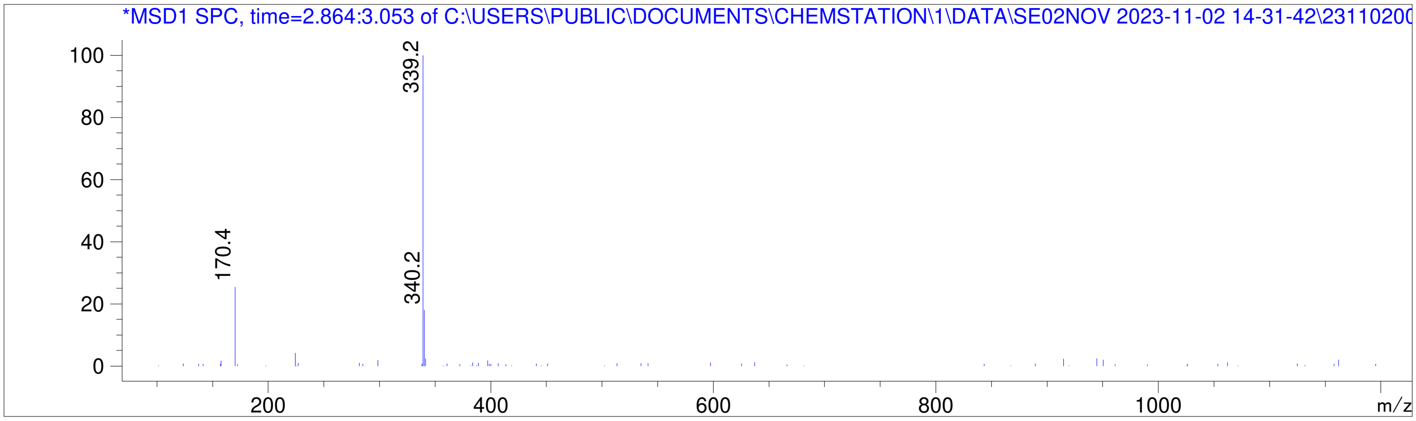

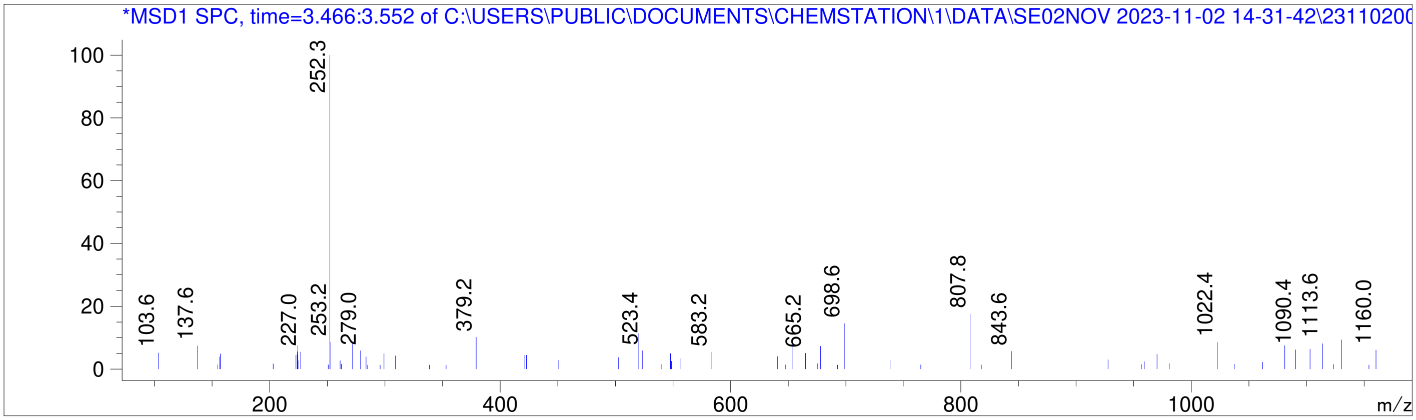

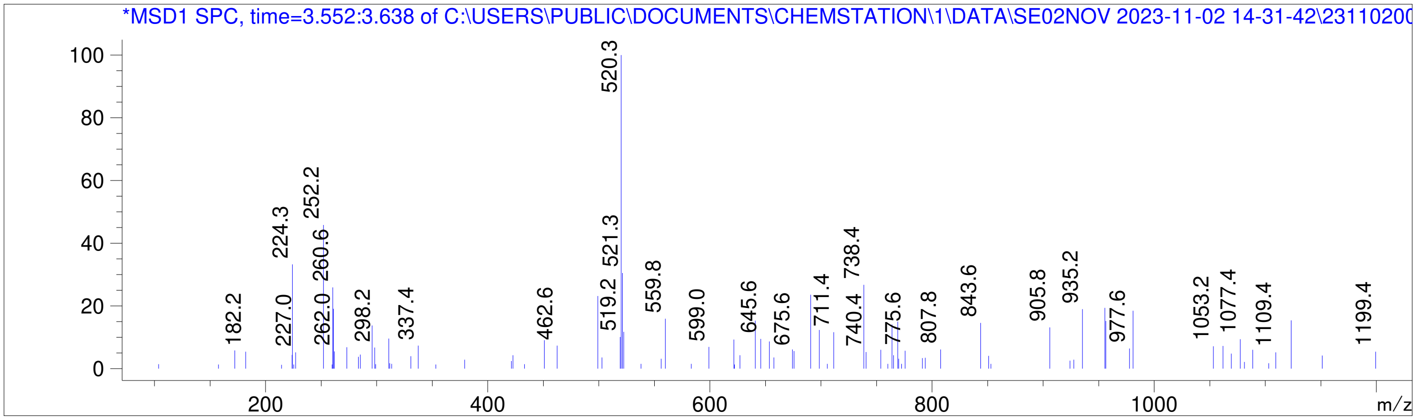

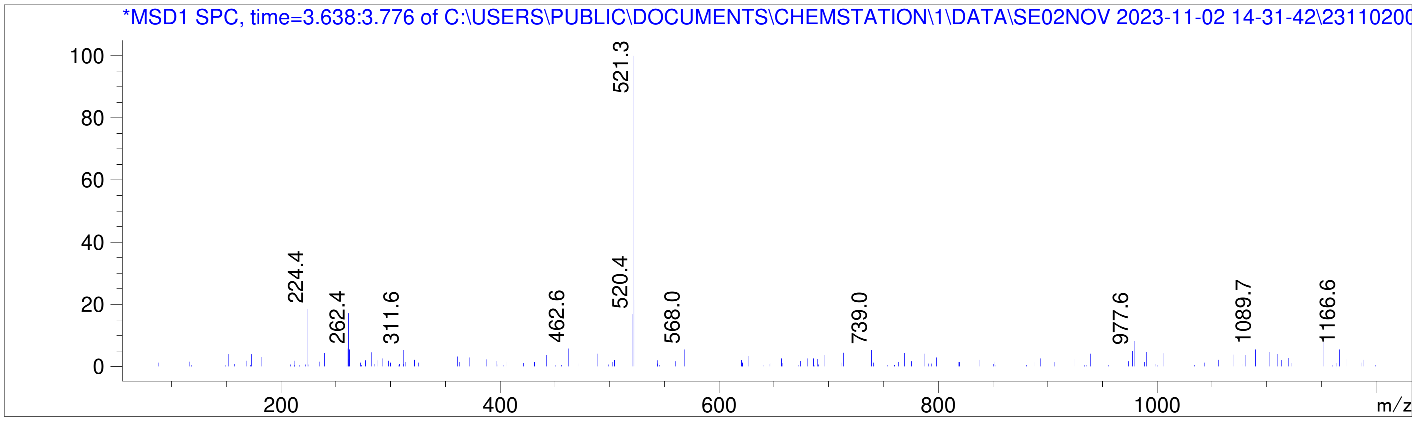
*MSD1 SPC, time=3.638:3.776 of C:\USERS\PUBLIC\DOCUMENTS\CHEMSTATION\1\DATA\SE02NOV 2023-11-02 14-31-42\2311020
521.3
520.4
224.4
262.4
311.6
462.6
568.0
739.0
977.6
1089.7
1166.6
m/z

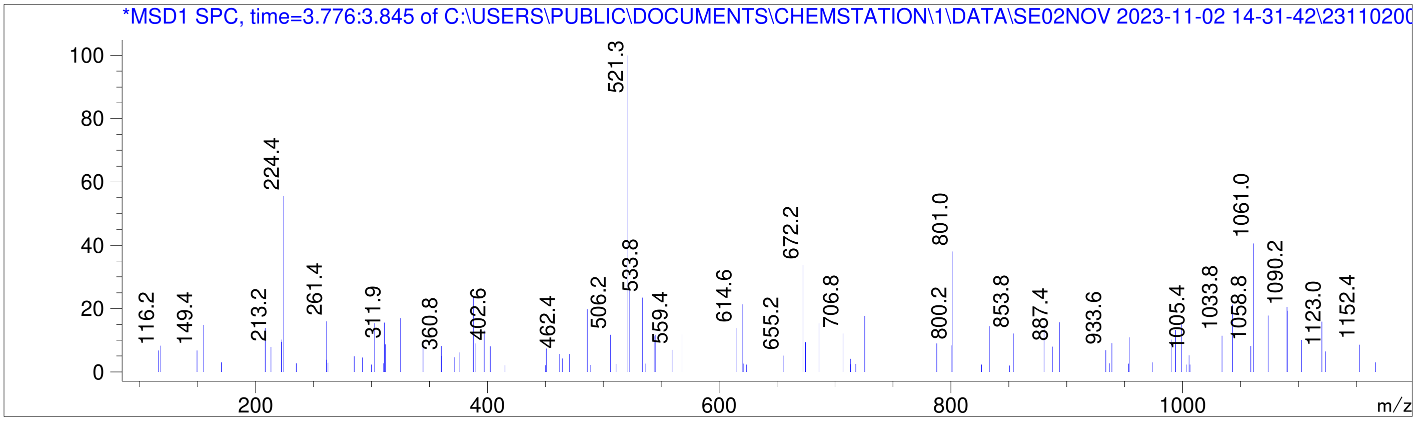
*MSD1 SPC, time=3.776:3.845 of C:\USERS\PUBLIC\DOCUMENTS\CHEMSTATION\1\DATA\SE02NOV 2023-11-02 14-31-42\2311020
521.3
224.4
116.2
149.4
213.2
261.4
311.9
360.8
402.6
462.4
506.2
533.8
559.4
614.6
655.2
672.2
706.8
800.2
801.0
853.8
887.4
933.6
1005.4
1033.8
1058.8
1061.0
1090.2
1123.0
1152.4
m/z

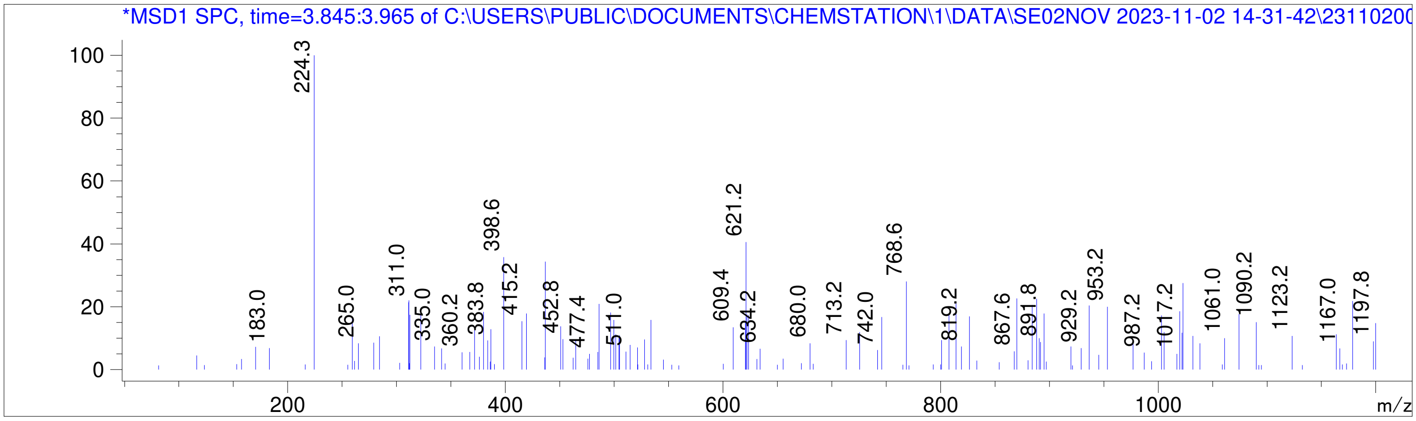
*MSD1 SPC, time=3.845:3.965 of C:\USERS\PUBLIC\DOCUMENTS\CHEMSTATION\1\DATA\SE02NOV 2023-11-02 14-31-42\2311020
224.3
183.0
265.0
311.0
335.0
360.2
383.8
398.6
415.2
452.8
477.4
511.0
609.4
621.2
634.2
680.0
713.2
742.0
768.6
819.2
867.6
891.8
929.2
953.2
987.2
1017.2
1061.0
1090.2
1123.2
1167.0
1197.8
m/z

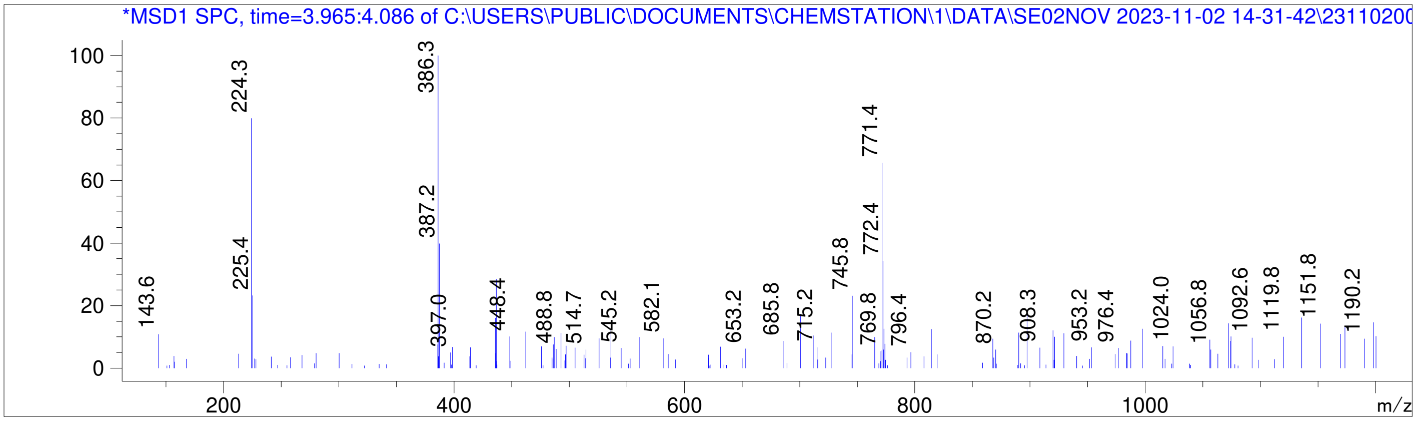
*MSD1 SPC, time=3.965:4.086 of C:\USERS\PUBLIC\DOCUMENTS\CHEMSTATION\1\DATA\SE02NOV 2023-11-02 14-31-42\2311020
143.6
224.3
225.4
386.3
387.2
397.0
448.4
488.8
514.7
545.2
582.1
653.2
685.8
715.2
745.8
769.8
771.4
772.4
796.4
870.2
908.3
953.2
976.4
1024.0
1056.8
1092.6
1119.8
1151.8
1190.2
m/z

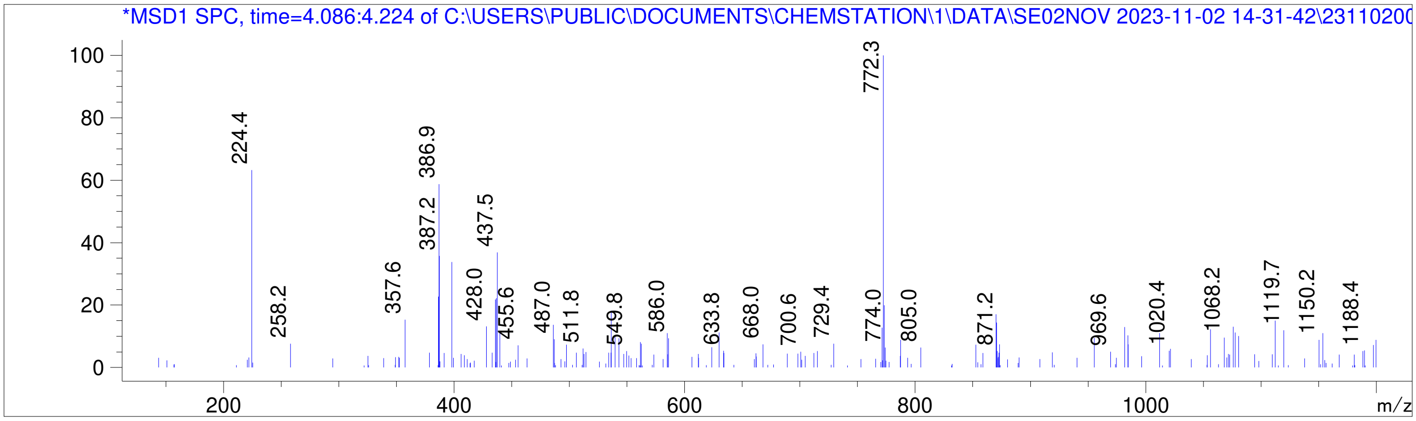
*MSD1 SPC, time=4.086:4.224 of C:\USERS\PUBLIC\DOCUMENTS\CHEMSTATION\1\DATA\SE02NOV 2023-11-02 14-31-42\2311020
224.4
258.2
357.6
386.9
387.2
428.0
437.5
455.6
487.0
511.8
549.8
586.0
633.8
668.0
700.6
729.4
772.3
774.0
805.0
871.2
969.6
1020.4
1068.2
1119.7
1150.2
1188.4
m/z

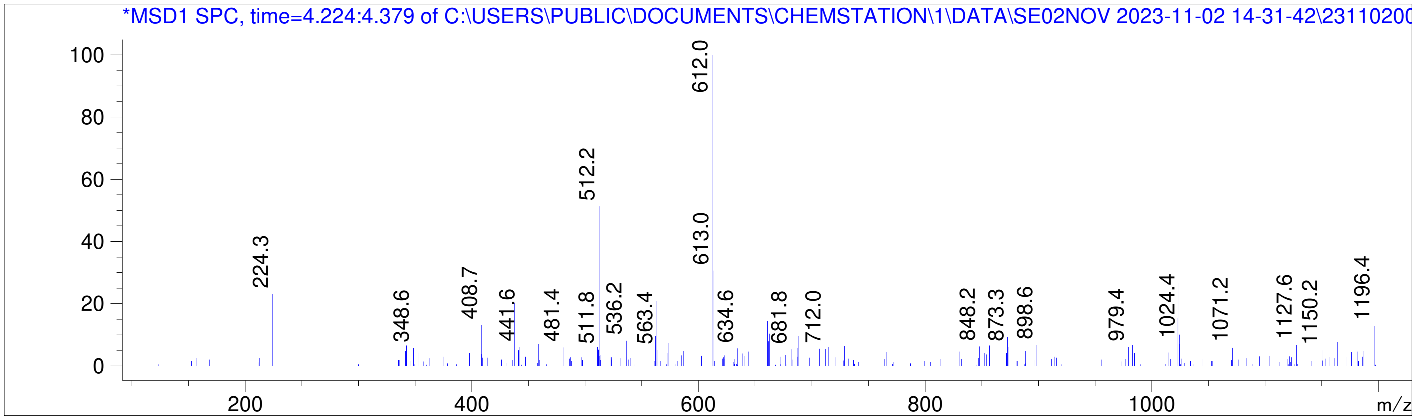
*MSD1 SPC, time=4.224:4.379 of C:\USERS\PUBLIC\DOCUMENTS\CHEMSTATION\1\DATA\SE02NOV 2023-11-02 14-31-42\2311020
224.3
348.6
408.7
441.6
481.4
511.8
512.2
536.2
563.4
612.0
613.0
634.6
681.8
712.0
848.2
873.3
898.6
979.4
1024.4
1071.2
1127.6
1150.2
1196.4
m/z

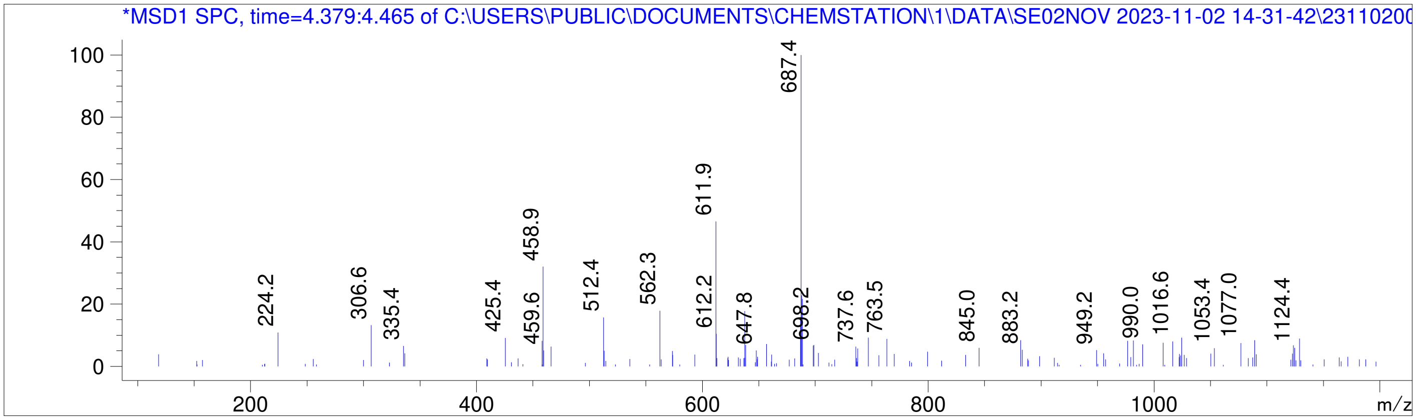
*MSD1 SPC, time=4.379:4.465 of C:\USERS\PUBLIC\DOCUMENTS\CHEMSTATION\1\DATA\SE02NOV 2023-11-02 14-31-42\2311020
224.2
306.6
335.4
425.4
458.9
459.6
512.4
562.3
611.9
612.2
647.8
687.4
698.2
737.6
763.5
845.0
883.2
949.2
990.0
1016.6
1053.4
1077.0
1124.4
m/z

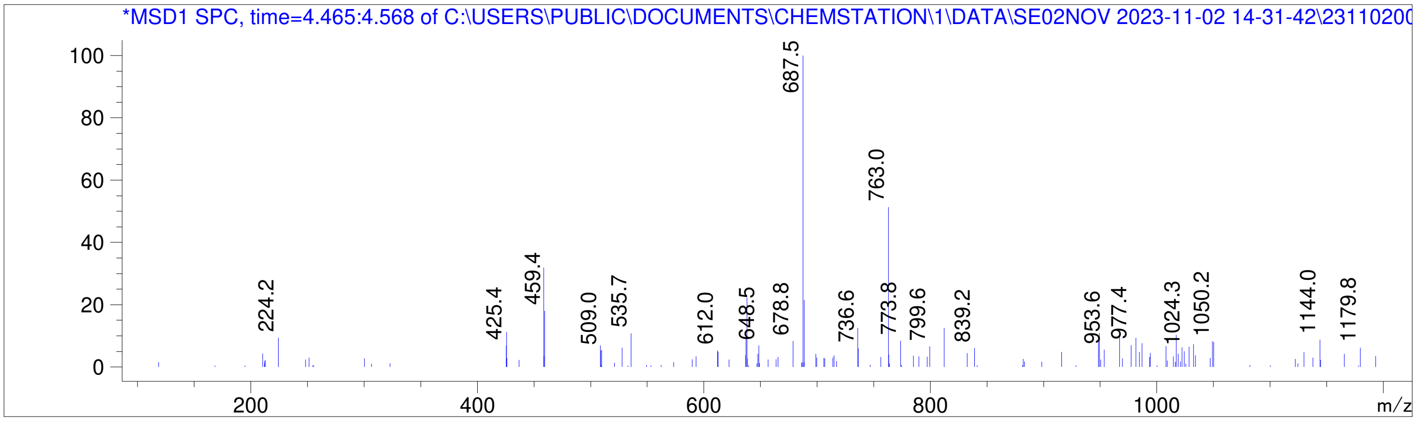
*MSD1 SPC, time=4.465:4.568 of C:\USERS\PUBLIC\DOCUMENTS\CHEMSTATION\1\DATA\SE02NOV 2023-11-02 14-31-42\2311020
100
80
60
40
20
0
224.2
425.4
459.4
509.0
535.7
612.0
648.5
678.8
687.5
736.6
763.0
773.8
799.6
839.2
953.6
977.4
1024.3
1050.2
1144.0
1179.8
200
400
600
800
1000
m/z

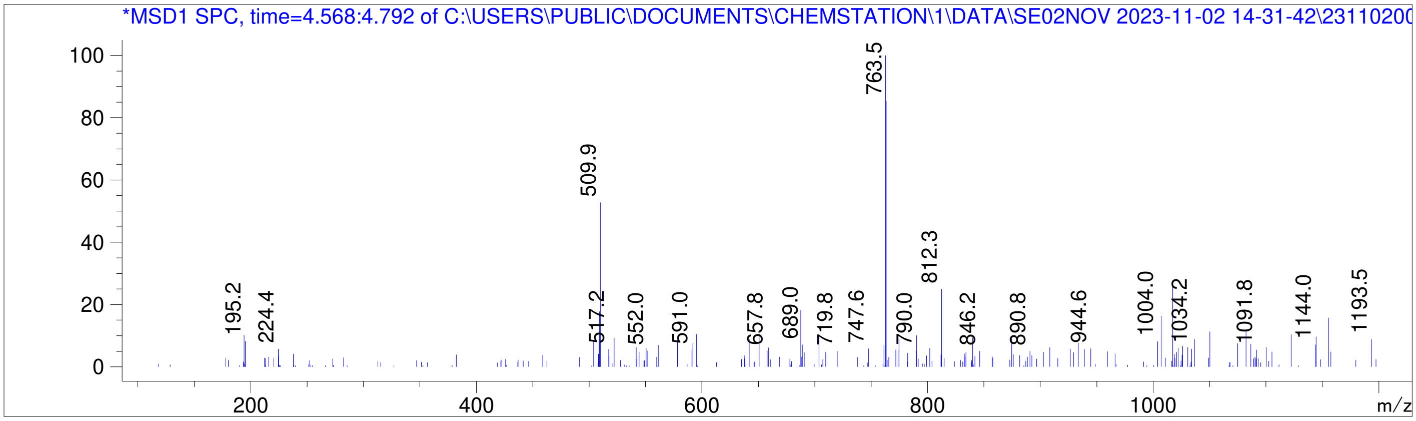
*MSD1 SPC, time=4.568:4.792 of C:\USERS\PUBLIC\DOCUMENTS\CHEMSTATION\1\DATA\SE02NOV 2023-11-02 14-31-42\2311020
100
80
60
40
20
0
195.2
224.4
509.9
517.2
552.0
591.0
657.8
689.0
719.8
747.6
763.5
790.0
812.3
846.2
890.8
944.6
1004.0
1034.2
1091.8
1144.0
1193.5
200
400
600
800
1000
m/z
