# Supplementary material for: Astrobiological implications of the stability and reactivity of peptide nucleic acid (PNA) in concentrated sulfuric acid
Source: Sci Adv. 2025 Mar 26;11(13):eadr0006. doi: 10.1126/sciadv.adr0006 (PMC11939054; doi:10.1126/sciadv.adr0006)

Injection Date : Tue, 7. Nov. 2023

Seq Line : 9
Location : 49
Inj. Vol. : 2 µl

Acq. Method : C:\Users\Public\Documents\ChemStation\1\Data\SE07NOV 2023-11-07 08-21-00\22010446 LCMS-6.M

Analysis Method : C:\Users\Public\Documents\ChemStation\1\Data\SE07NOV 2023-11-07 08-21-00\22010446 LCMS-6.M (Sequence Method)

Waters XBridge Phenyl (4.6 * 150 mm; 3.5 µm); 0.05% TFA (aq) / AcN: 100/0 (0.0 min) --> (6.0 min) --> 70/30 (0.0 min) --> (2.0 min) --> 10/90 (2.0 min); Flow: 1.0 ml/min; MSD1 = positive; MSD2 = negative

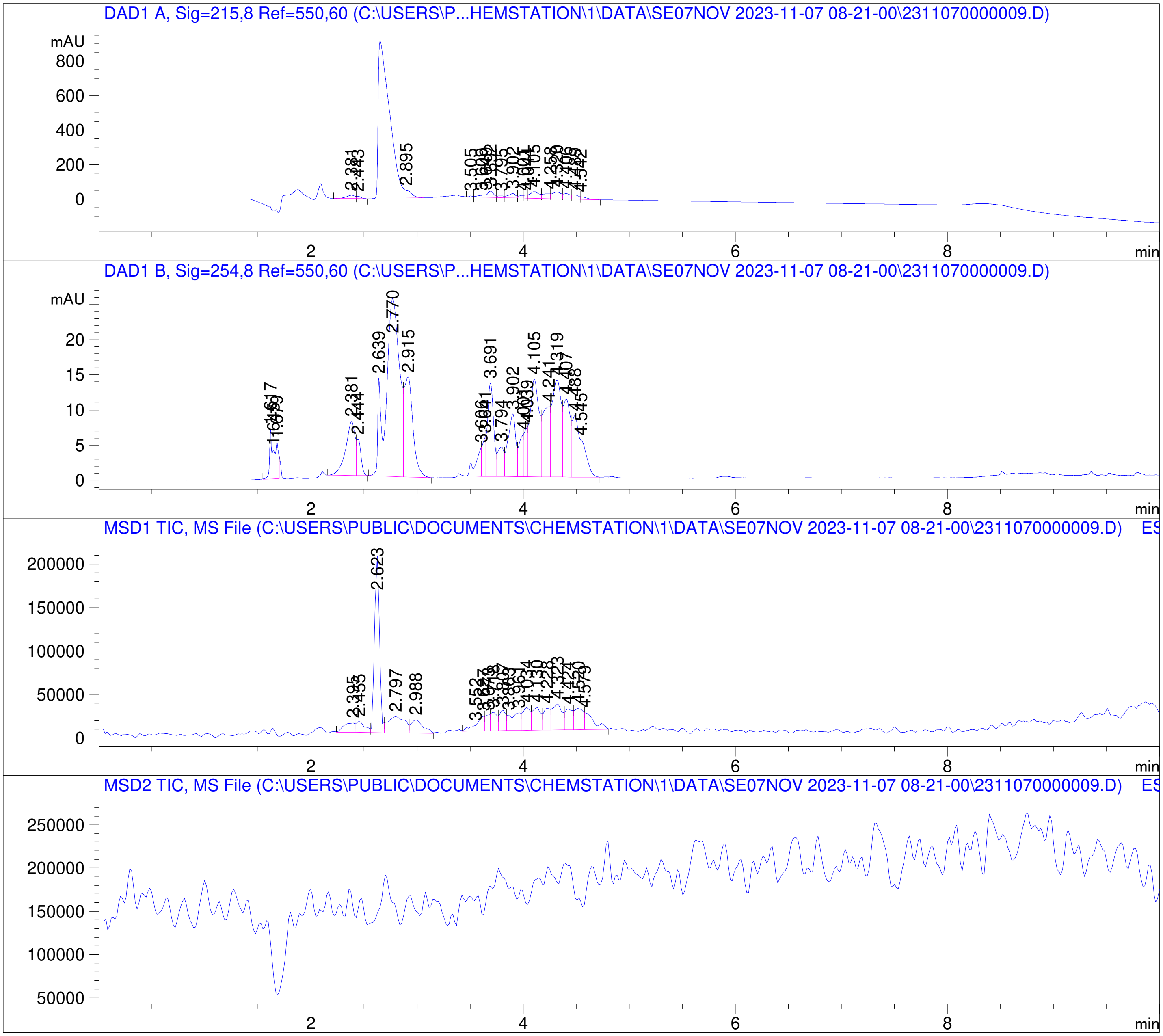

DAD1 A, Sig=215,8 Ref=550,60

Peak #	Ret. Time [min]	Area [mV *s]	Area %
1	2.381	112.052	6.722
2	2.443	35.906	2.154
3	2.895	164.667	9.878
4	3.505	11.287	0.677
5	3.609	31.841	1.910
6	3.649	29.370	1.762
7	3.692	134.883	8.091
8	3.795	42.158	2.529
9	3.902	117.342	7.039
10	4.001	43.756	2.625
11	4.044	44.472	2.668
12	4.105	225.206	13.510
13	4.258	137.207	8.231
14	4.320	229.106	13.744
15	4.406	139.540	8.371
16	4.489	110.358	6.620
17	4.542	57.856	3.471

DAD1 B, Sig=254,8 Ref=550,60

Peak #	Ret. Time [min]	Area [mV *s]	Area %
1	1.617	8.914	0.999
2	1.645	6.510	0.729
3	1.679	9.889	1.108
4	2.381	47.641	5.338
5	2.444	14.400	1.613
6	2.639	31.269	3.503
7	2.770	197.274	22.103
8	2.915	80.995	9.075
9	3.606	11.910	1.334
10	3.641	9.873	1.106
11	3.691	55.278	6.193
12	3.794	17.112	1.917
13	3.902	45.009	5.043
14	4.001	16.810	1.883
15	4.039	15.077	1.689
16	4.105	82.590	9.254
17	4.241	48.877	5.476
18	4.319	83.036	9.303
19	4.407	52.595	5.893
20	4.488	37.644	4.218
21	4.545	19.827	2.221

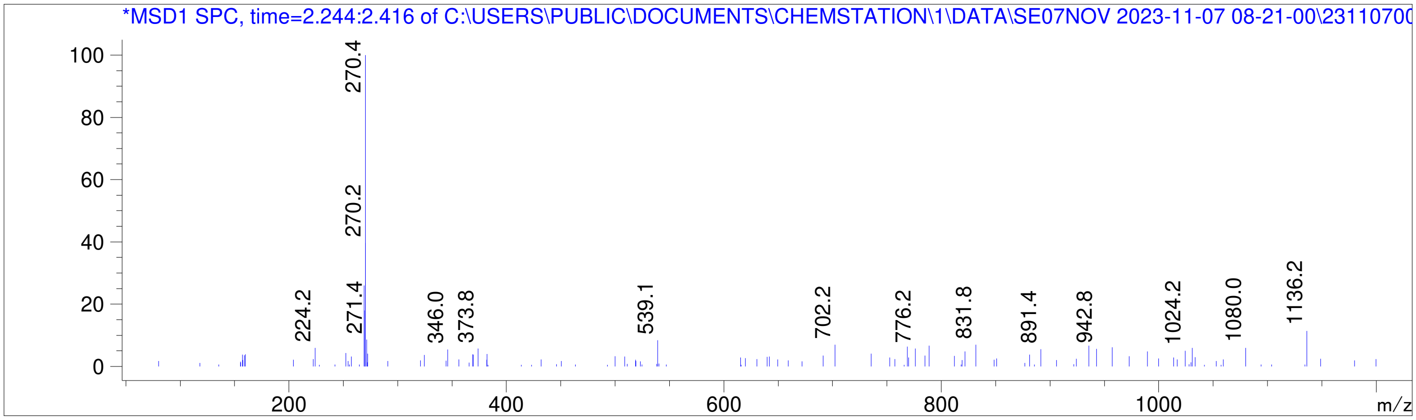
*MSD1 SPC, time=2.244:2.416 of C:\USERS\PUBLIC\DOCUMENTS\CHEMSTATION\1\DATA\SE07NOV 2023-11-07 08-21-00\2311070
270.4
270.2
224.2
271.4
346.0
373.8
539.1
702.2
776.2
831.8
891.4
942.8
1024.2
1080.0
1136.2
m/z

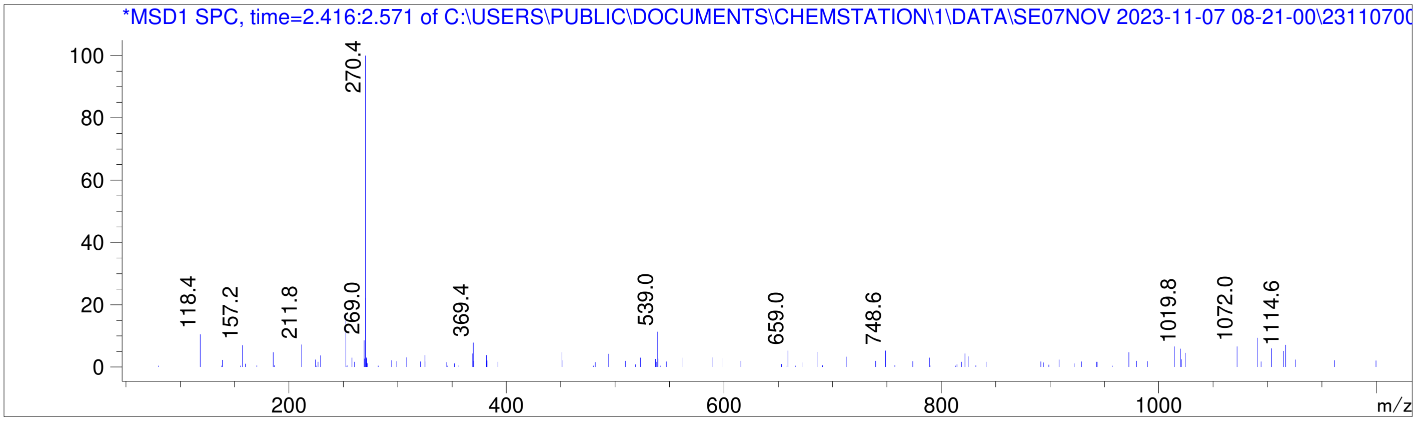
*MSD1 SPC, time=2.416:2.571 of C:\USERS\PUBLIC\DOCUMENTS\CHEMSTATION\1\DATA\SE07NOV 2023-11-07 08-21-00\2311070
270.4
118.4
157.2
211.8
269.0
369.4
539.0
659.0
748.6
1019.8
1072.0
1114.6
m/z

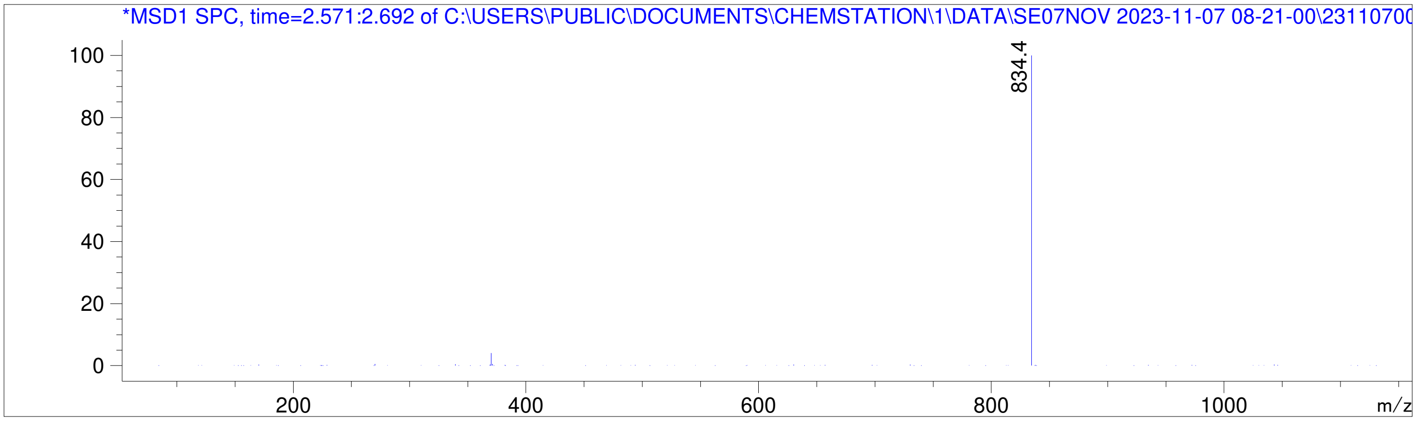
*MSD1 SPC, time=2.571:2.692 of C:\USERS\PUBLIC\DOCUMENTS\CHEMSTATION\1\DATA\SE07NOV 2023-11-07 08-21-00\2311070
834.4
m/z

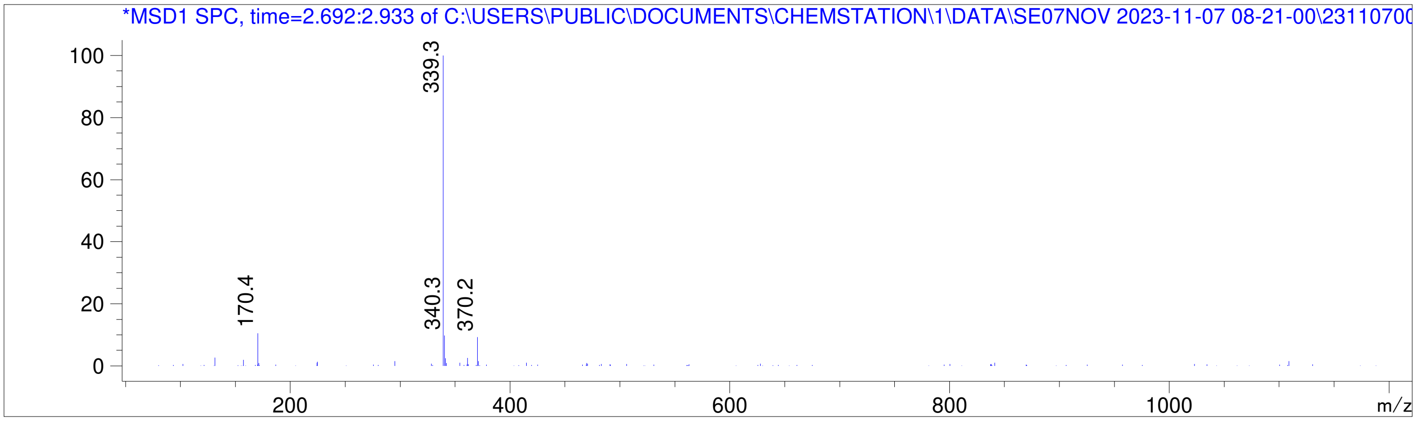
*MSD1 SPC, time=2.692:2.933 of C:\USERS\PUBLIC\DOCUMENTS\CHEMSTATION\1\DATA\SE07NOV 2023-11-07 08-21-00\2311070
339.3
170.4
340.3
370.2
m/z

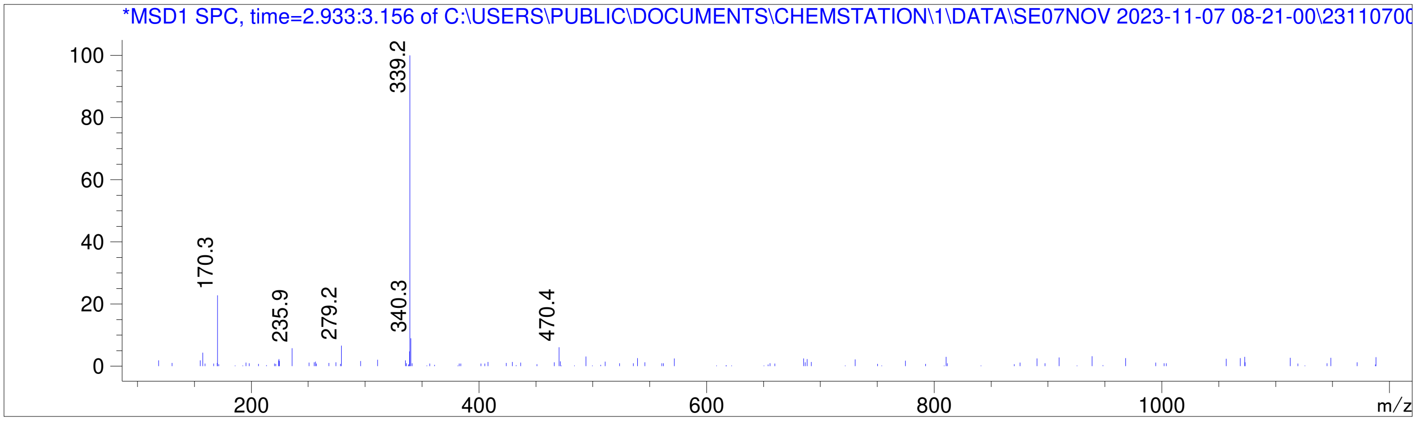
*MSD1 SPC, time=2.933:3.156 of C:\USERS\PUBLIC\DOCUMENTS\CHEMSTATION\1\DATA\SE07NOV 2023-11-07 08-21-00\2311070
339.2
170.3
235.9
279.2
340.3
470.4
m/z

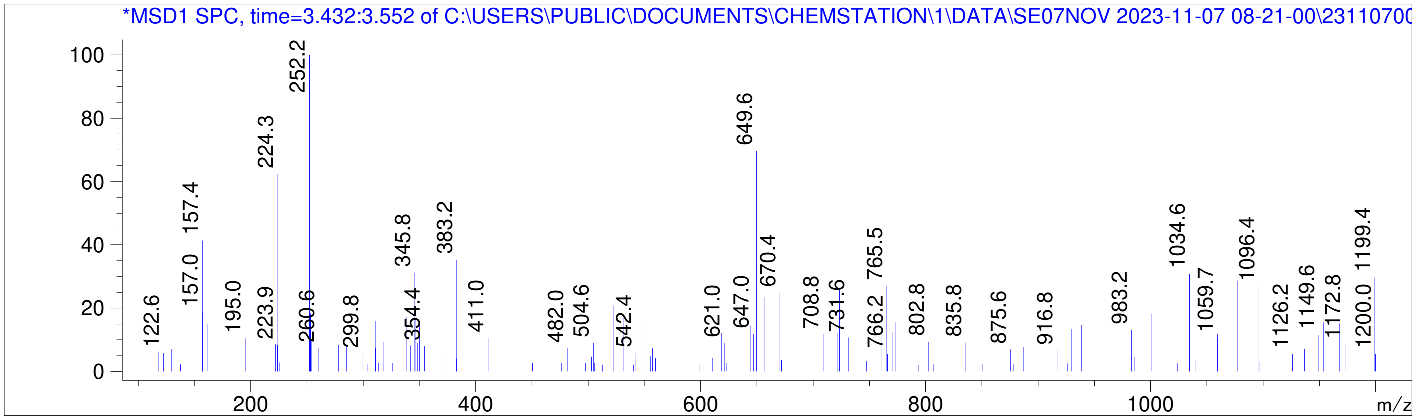
*MSD1 SPC, time=3.432:3.552 of C:\USERS\PUBLIC\DOCUMENTS\CHEMSTATION\1\DATA\SE07NOV 2023-11-07 08-21-00\2311070
252.2
224.3
649.6
157.4
345.8
383.2
670.4
765.5
1034.6
1096.4
1199.4
122.6
157.0
195.0
223.9
260.6
299.8
354.4
411.0
482.0
504.6
542.4
621.0
647.0
708.8
731.6
766.2
802.8
835.8
875.6
916.8
983.2
1059.7
1126.2
1149.6
1172.8
1200.0
m/z

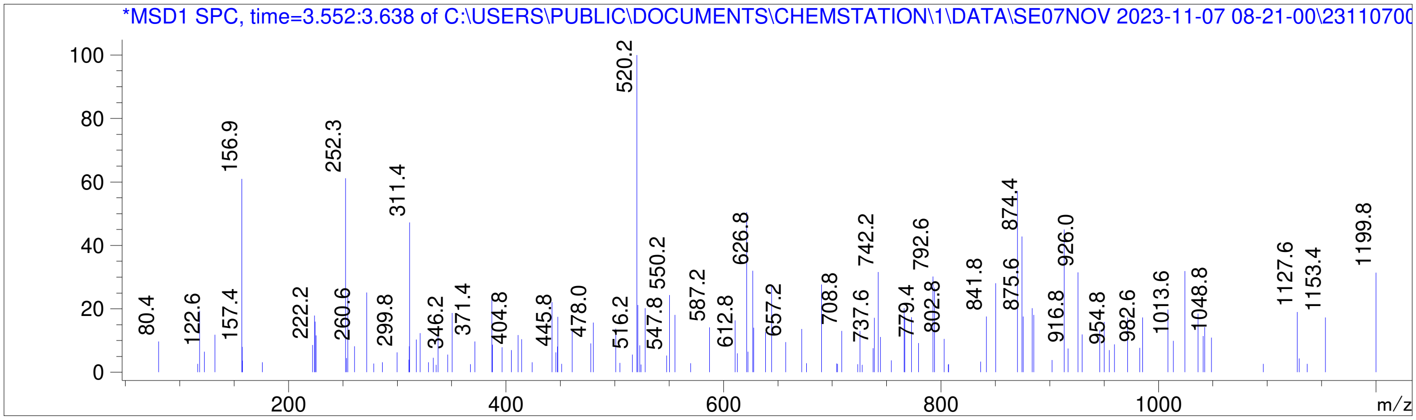
*MSD1 SPC, time=3.552:3.638 of C:\USERS\PUBLIC\DOCUMENTS\CHEMSTATION\1\DATA\SE07NOV 2023-11-07 08-21-00\2311070
520.2
156.9
252.3
311.4
874.4
550.2
626.8
742.2
792.6
926.0
1199.8
80.4
122.6
157.4
222.2
260.6
299.8
346.2
371.4
404.8
445.8
478.0
516.2
547.8
587.2
612.8
657.2
708.8
737.6
779.4
802.8
841.8
875.6
916.8
954.8
982.6
1013.6
1048.8
1127.6
1153.4
m/z

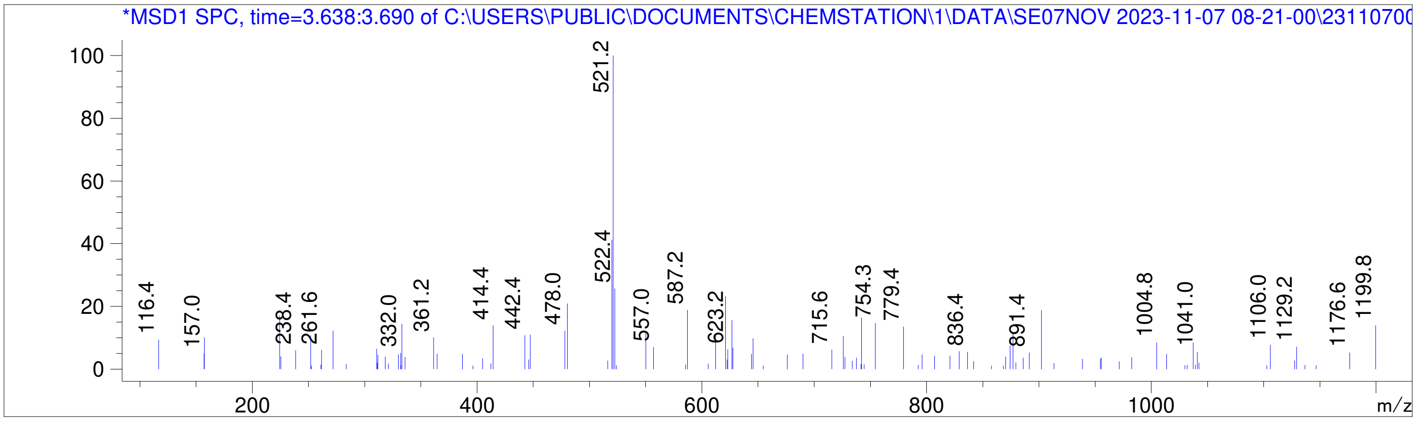
*MSD1 SPC, time=3.638:3.690 of C:\USERS\PUBLIC\DOCUMENTS\CHEMSTATION\1\DATA\SE07NOV 2023-11-07 08-21-00\2311070
521.2
522.4
116.4
157.0
238.4
261.6
332.0
361.2
414.4
442.4
478.0
557.0
587.2
623.2
715.6
754.3
779.4
836.4
891.4
1004.8
1041.0
1106.0
1129.2
1176.6
1199.8
m/z

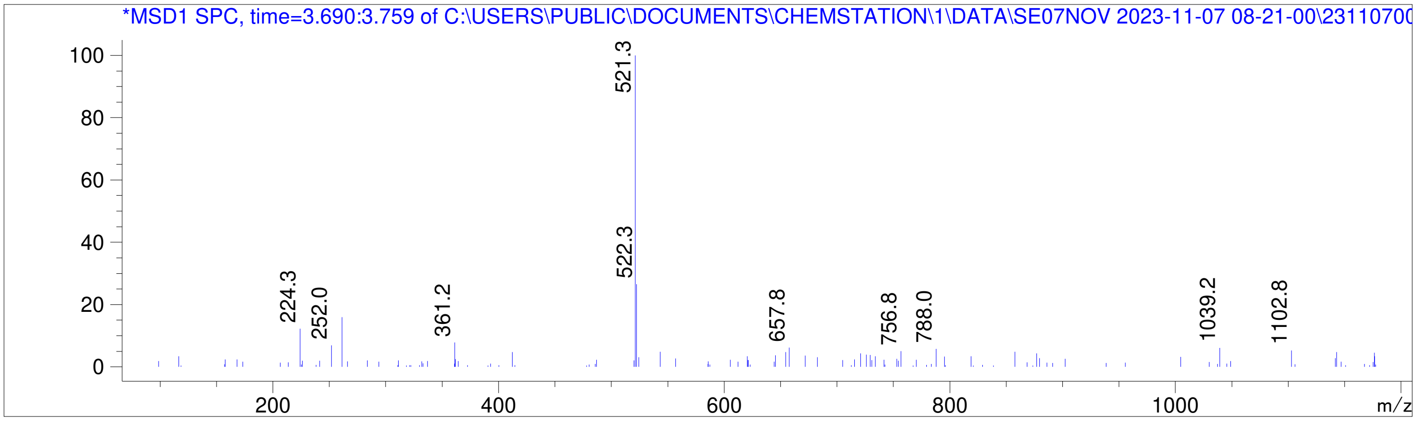
*MSD1 SPC, time=3.690:3.759 of C:\USERS\PUBLIC\DOCUMENTS\CHEMSTATION\1\DATA\SE07NOV 2023-11-07 08-21-00\2311070
521.3
522.3
224.3
252.0
361.2
657.8
756.8
788.0
1039.2
1102.8
m/z

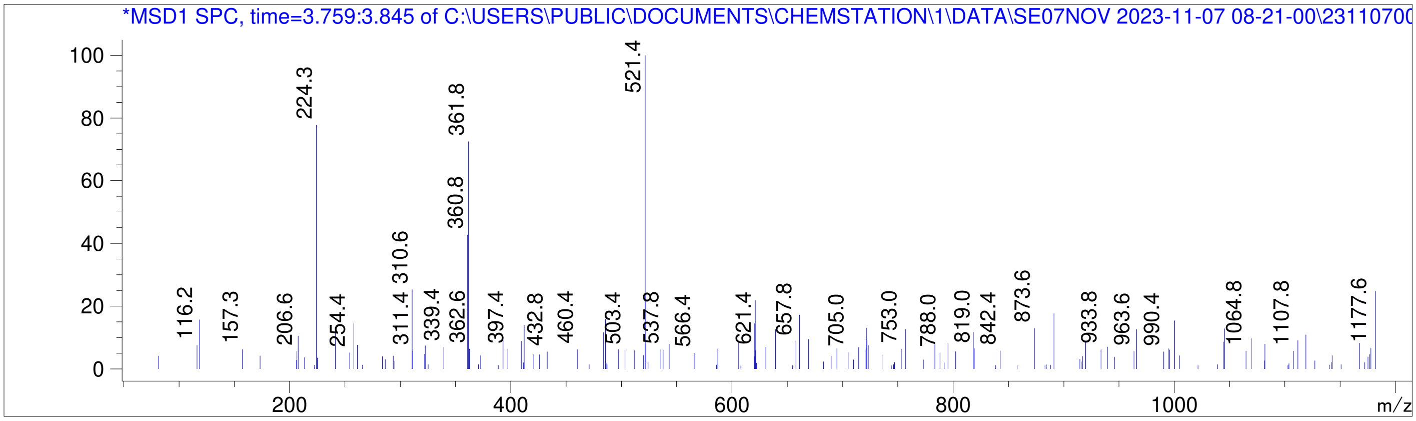
*MSD1 SPC, time=3.759:3.845 of C:\USERS\PUBLIC\DOCUMENTS\CHEMSTATION\1\DATA\SE07NOV 2023-11-07 08-21-00\2311070
521.4
224.3
361.8
360.8
310.6
116.2
157.3
206.6
254.4
311.4
339.4
362.6
397.4
432.8
460.4
503.4
537.8
566.4
621.4
657.8
705.0
753.0
788.0
819.0
842.4
873.6
933.8
963.6
990.4
1064.8
1107.8
1177.6
m/z

Data -> C:\USERS\PUBLIC\DOCUMENTS\CHEMSTATION\1\DATA\SE07NOV 2023-11-07 08-21-00\ ->
Sample-> CPT22010446-13-C2-80deg-1h

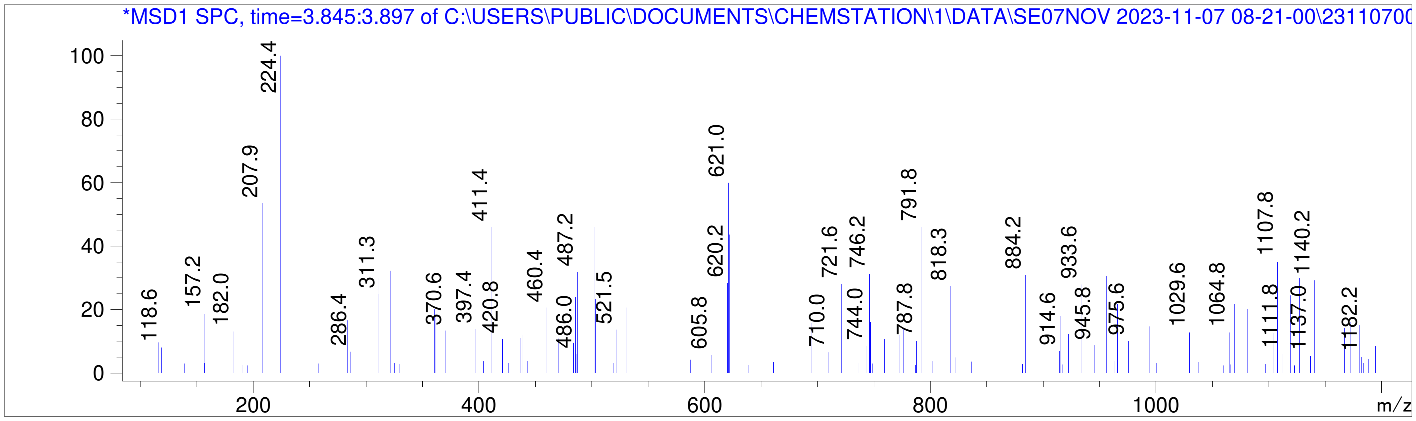

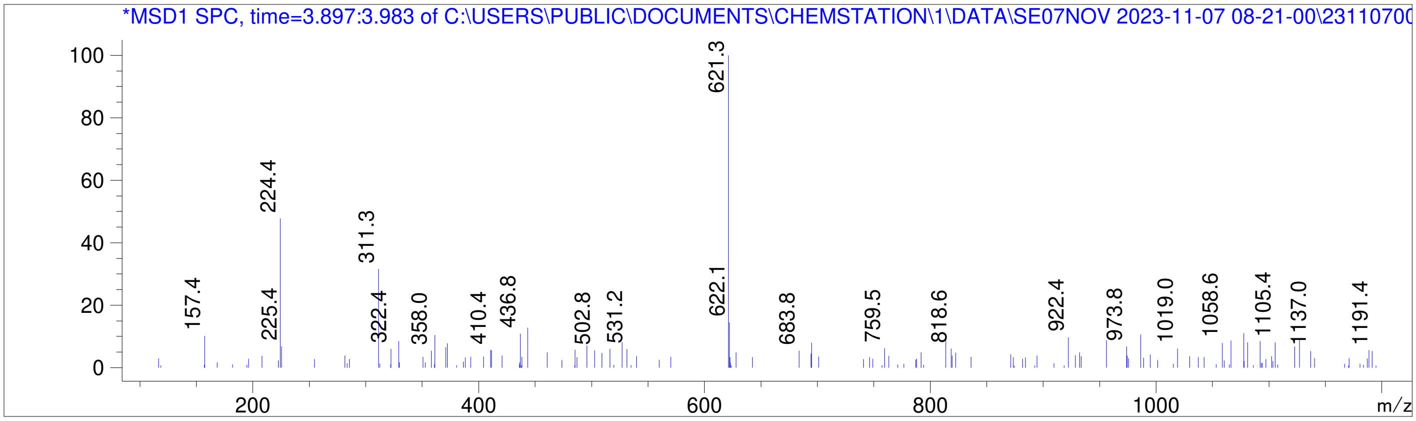

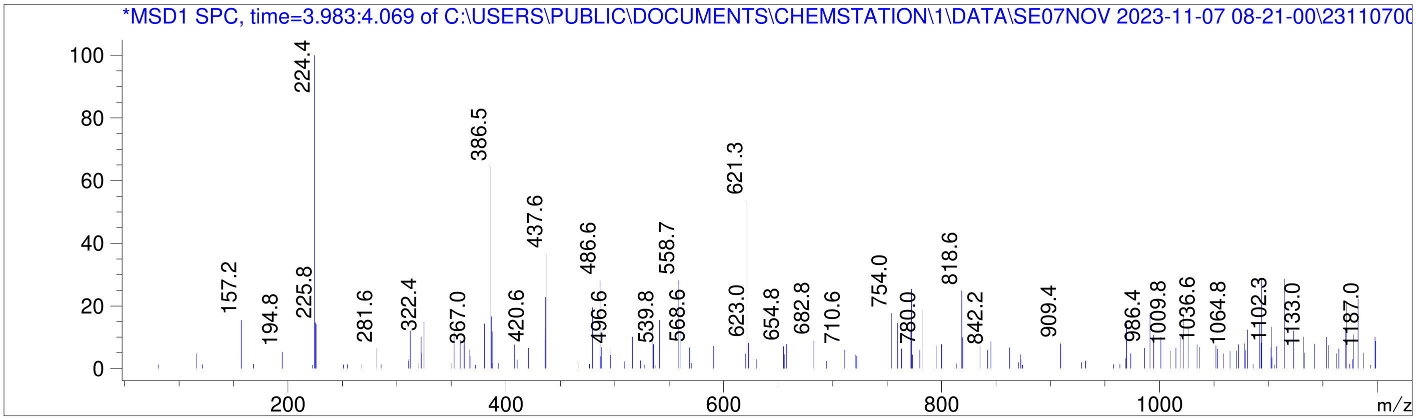

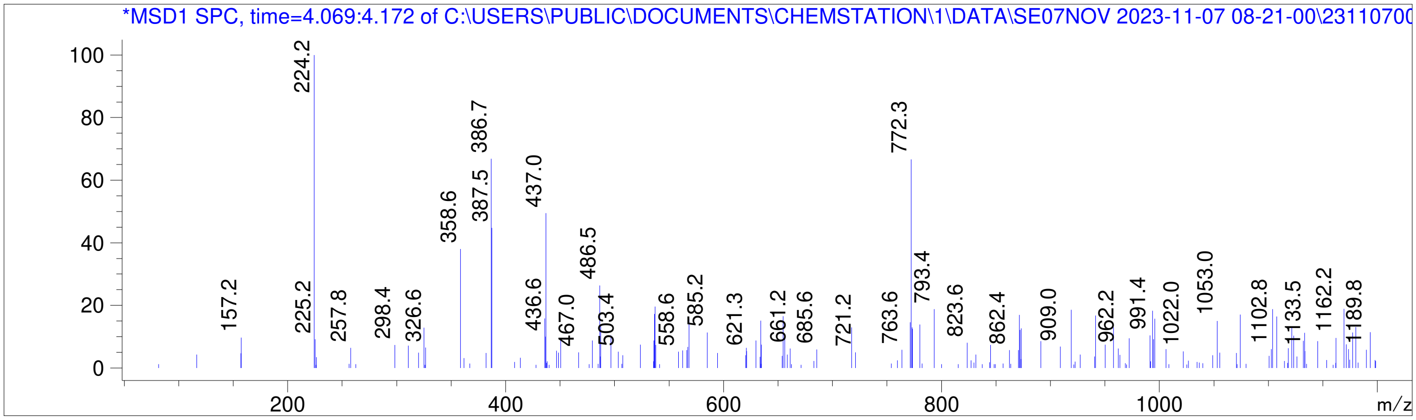

Sample-> CPT22010446-13-C2-80deg-1h

==

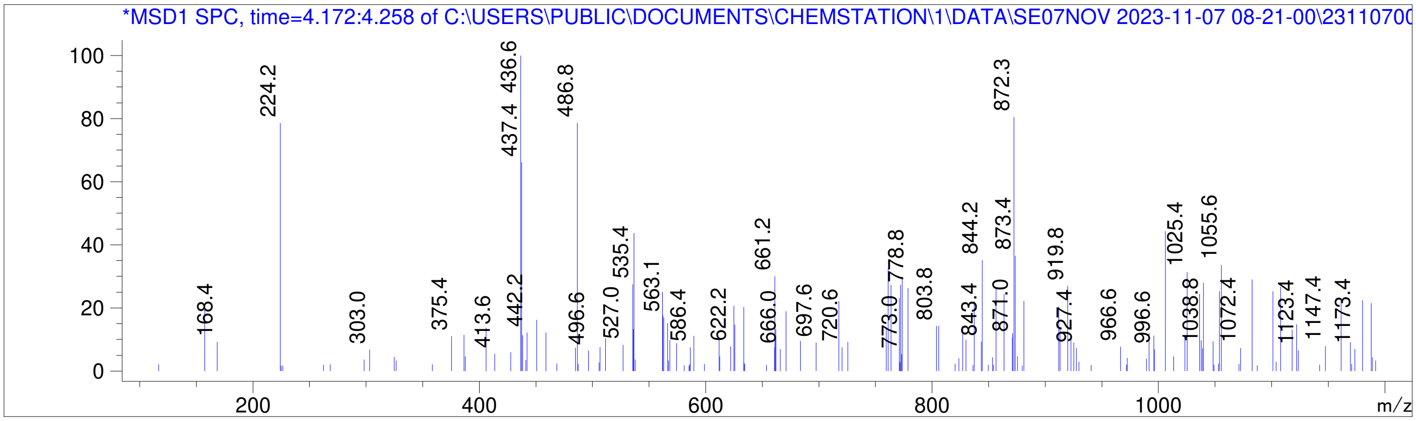

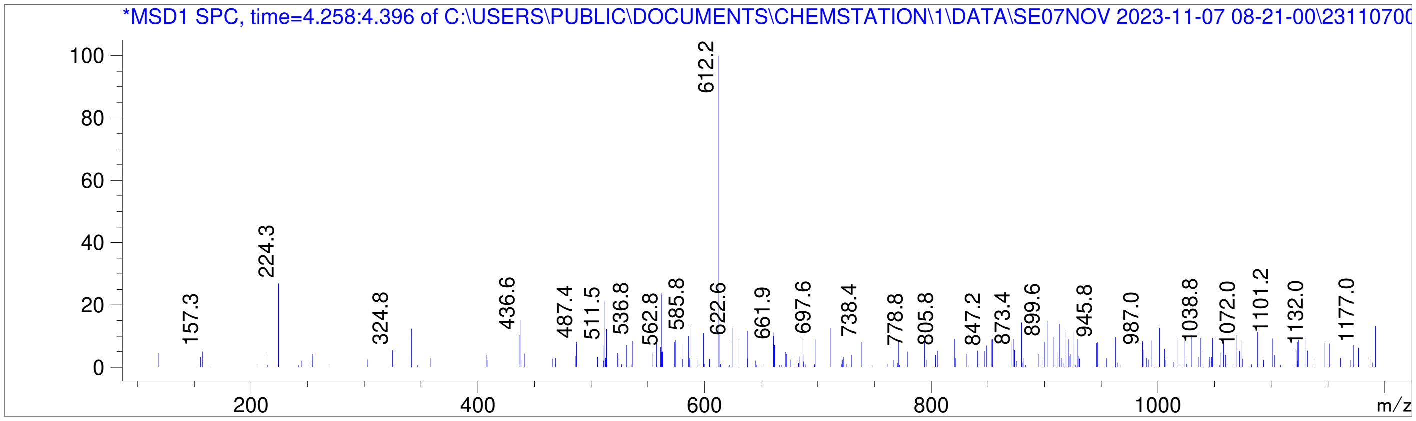

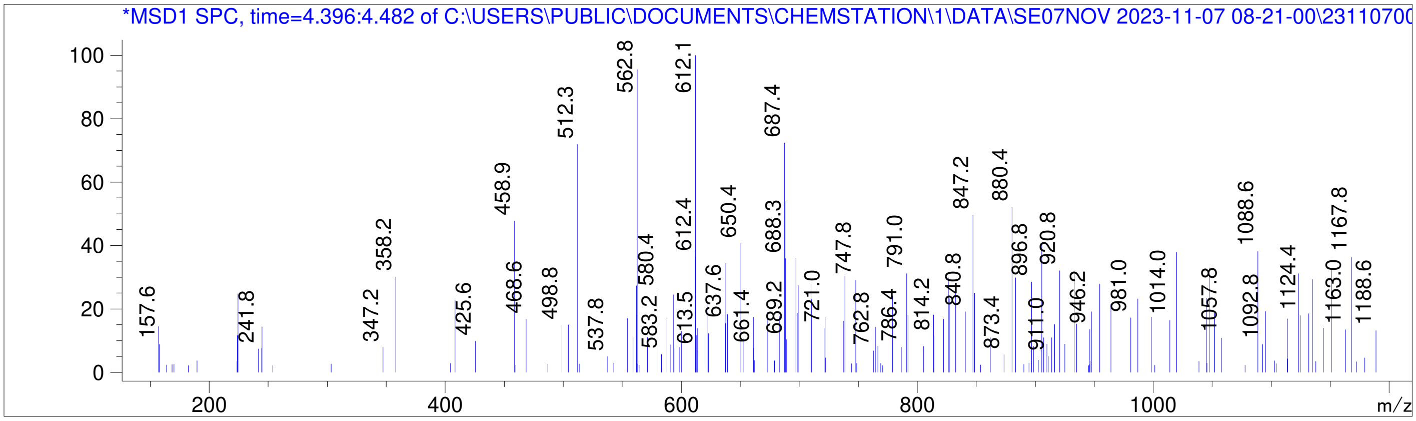

Sample-> CPT22010446-13-C2-80deg-1h

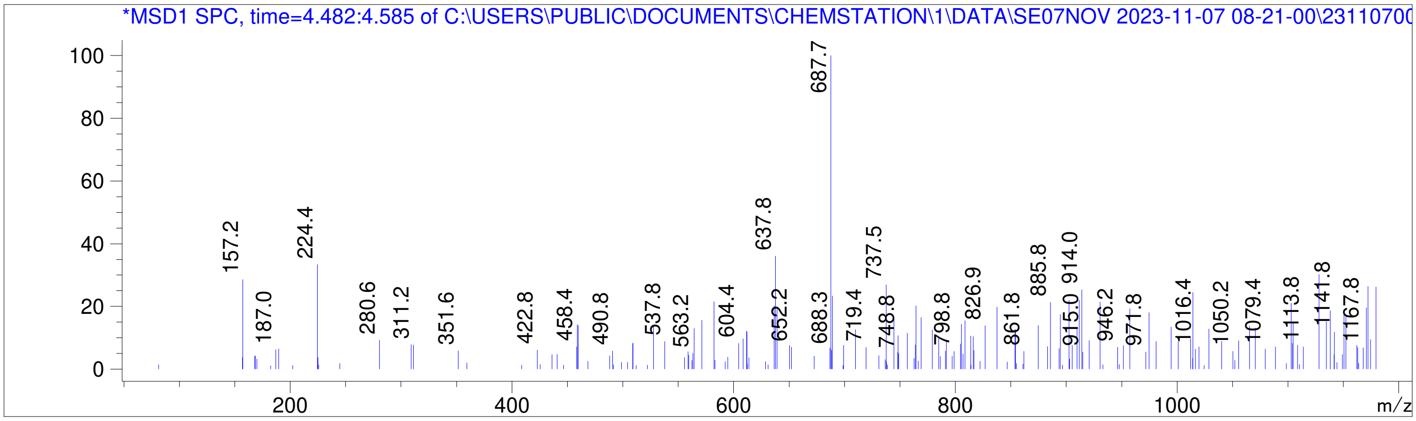

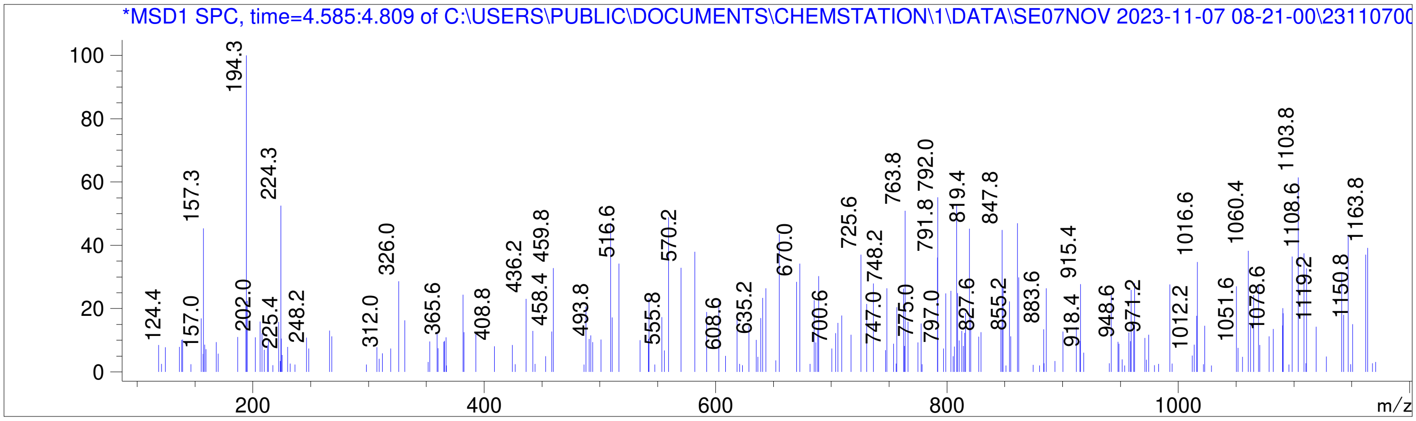

Supplement: Supplementary file 2 — Data S1 and S2 [file sciadv.adr0006_data_s1_and_s2.zip › Supplementary Dataset 1-LCMS DATA/LCMS PNA Hexamers A-T/LCMS C6 50C_80C/80C/1h/CPT22010446-13-C2-80deg-1h.pdf]
