# Supplementary material for: Astrobiological implications of the stability and reactivity of peptide nucleic acid (PNA) in concentrated sulfuric acid
Source: Sci Adv. 2025 Mar 26;11(13):eadr0006. doi: 10.1126/sciadv.adr0006 (PMC11939054; doi:10.1126/sciadv.adr0006)
Supplement: Supplementary file 2 — Data S1 and S2 [file sciadv.adr0006_data_s1_and_s2.zip › Supplementary Dataset 1-LCMS DATA/LCMS PNA Hexamers A-T/LCMS C6 50C_80C/80C/1h/CPT22010446-21-C1-80dg-1h.pdf]

===

Injection Date : Fri, 20. Oct. 2023   Seq Line   :   39
                                               Location   :   14
                                               Inj. Vol. :   2 µl

Acq. Method   : C:\Users\Public\Documents\ChemStation\1\Data\SE19OCT 2023-10-19 16-56-54\22010446 LCMS-6.M
Analysis Method : C:\Users\Public\Documents\ChemStation\1\Data\SE19OCT 2023-10-19 16-56-54\22010446 LCMS-6.M (Sequence Method)
Waters XBridge Phenyl (4.6 * 150 mm; 3.5 µm); 0.05% TFA (aq) / AcN: 100/0 (0.0 min) --> (6.0 min) --> 70/30 (0.0 min) --> (2.0 min) --> 10/90 (2.0 min); Flow: 1.0 ml/min;
MSD1 = positive; MSD2 = negative

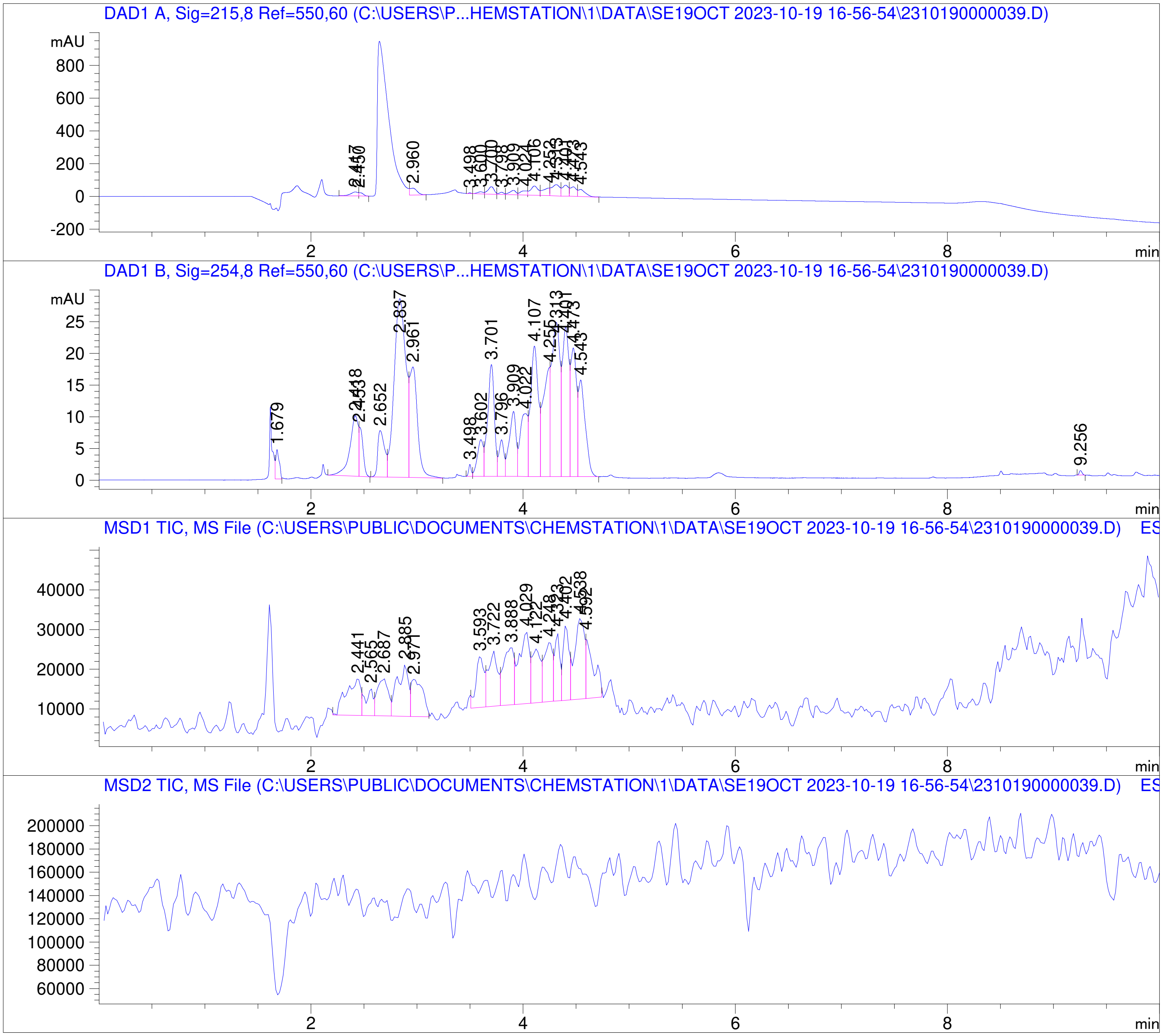

DAD1 A, Sig=215,8 Ref=550,60

Peak #	Ret. Time [min]	Area [mV *s]	Area %
1	2.417	121.148	4.908
2	2.450	55.507	2.249
3	2.960	194.706	7.888
4	3.498	7.477	0.303
5	3.600	50.893	2.062
6	3.700	184.679	7.482
7	3.798	42.103	1.706
8	3.909	111.529	4.518
9	4.024	117.806	4.773
10	4.106	284.320	11.519
11	4.252	212.129	8.594
12	4.313	362.826	14.699
13	4.401	279.409	11.320
14	4.473	237.574	9.625
15	4.543	206.271	8.357

DAD1 B, Sig=254,8 Ref=550,60

Peak #	Ret. Time [min]	Area [mV *s]	Area %
1	1.679	10.396	0.863
2	2.418	52.914	4.392
3	2.453	19.412	1.611
4	2.652	33.419	2.774
5	2.837	215.874	17.917
6	2.961	91.613	7.604
7	3.498	3.187	0.265
8	3.602	22.780	1.891
9	3.701	77.713	6.450
10	3.796	19.383	1.609
11	3.909	45.541	3.780
12	4.022	50.888	4.224
13	4.107	103.205	8.566
14	4.255	77.243	6.411
15	4.313	129.820	10.775
16	4.401	102.477	8.505
17	4.473	77.292	6.415
18	4.543	70.405	5.843
19	9.256	1.299	0.108

Data -> C:\USERS\PUBLIC\DOCUMENTS\CHEMSTATION\1\DATA\SE19OCT 2023-10-19 16-56-54\ ->
Sample-> CPT22010446-21-C1-80dg-1h
==

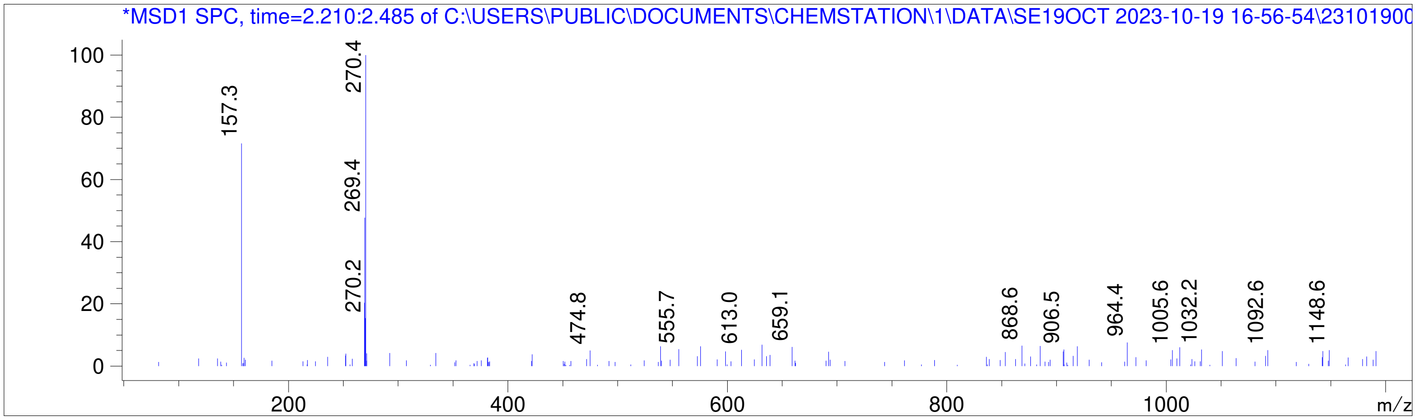

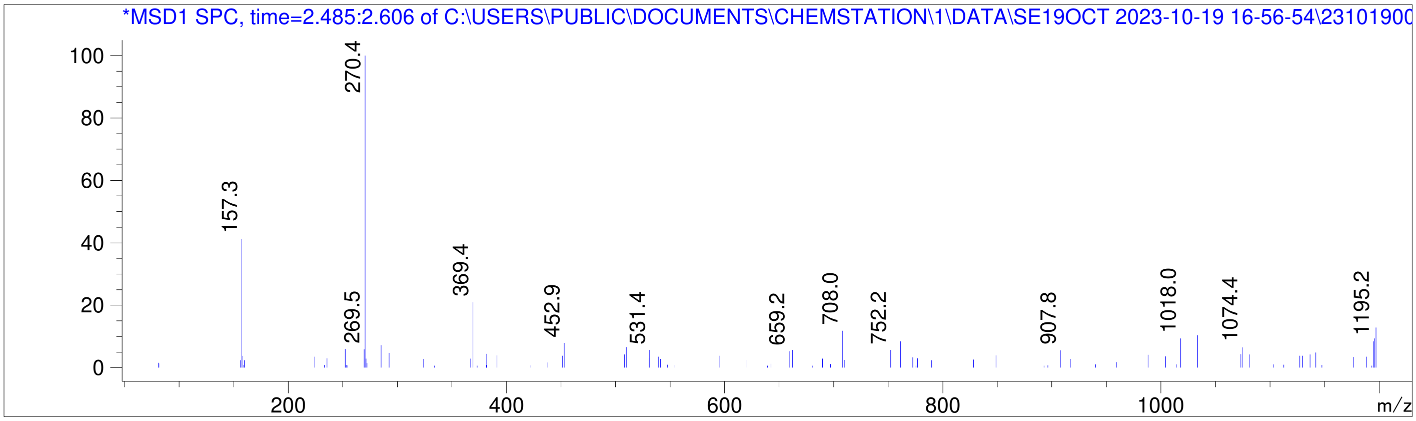

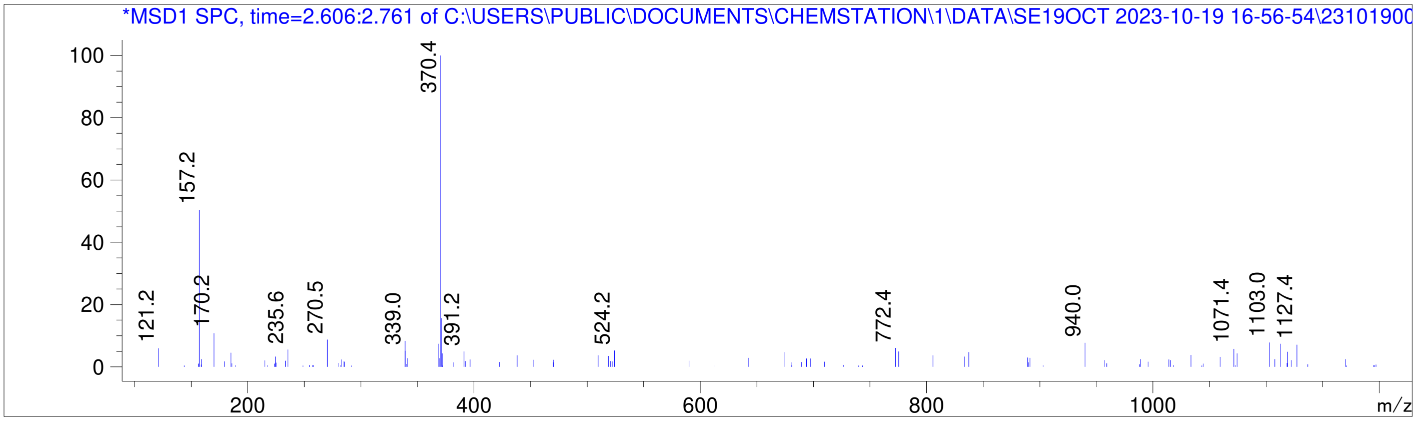

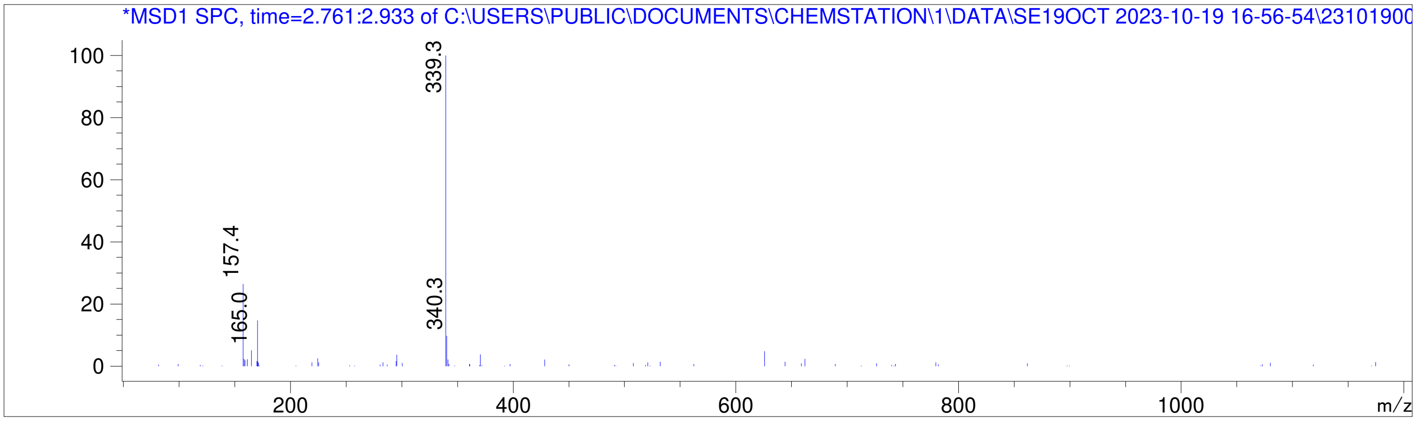
*MSD1 SPC, time=2.761:2.933 of C:\USERS\PUBLIC\DOCUMENTS\CHEMSTATION\1\DATA\SE19OCT 2023-10-19 16-56-54\23101900
339.3
157.4
165.0
340.3
m/z

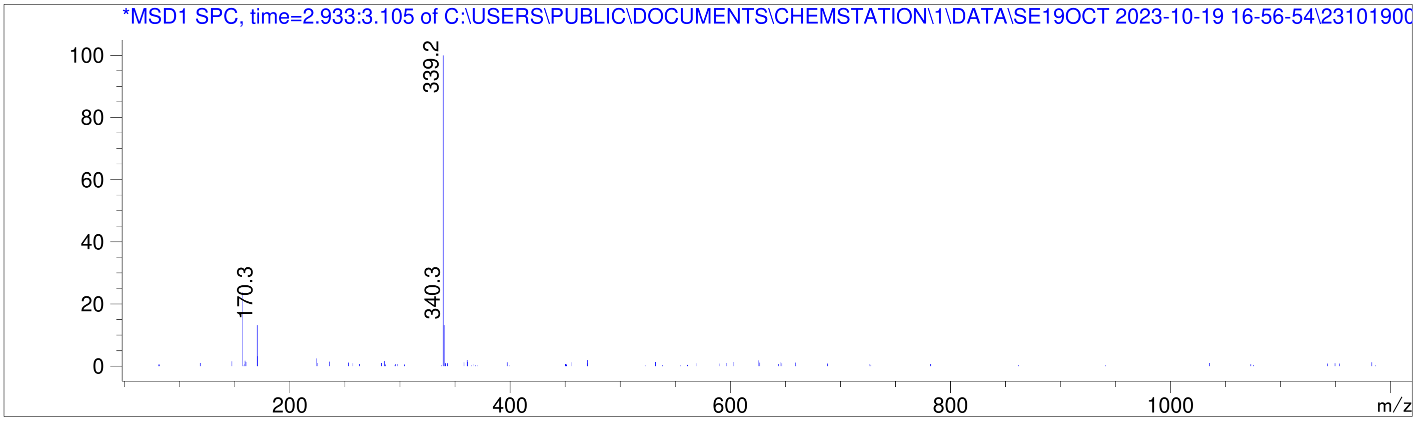
*MSD1 SPC, time=2.933:3.105 of C:\USERS\PUBLIC\DOCUMENTS\CHEMSTATION\1\DATA\SE19OCT 2023-10-19 16-56-54\23101900
339.2
170.3
340.3
m/z

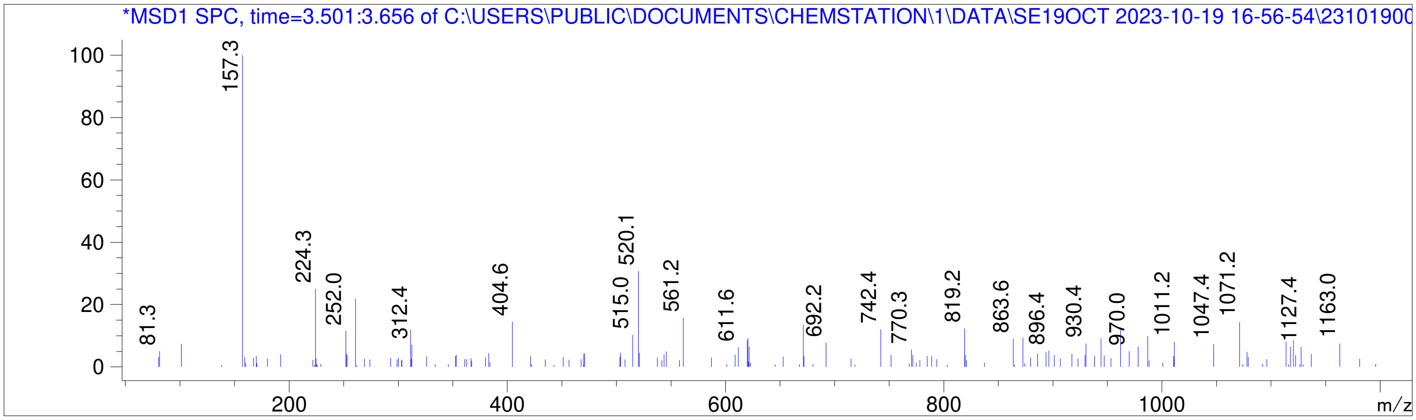
*MSD1 SPC, time=3.501:3.656 of C:\USERS\PUBLIC\DOCUMENTS\CHEMSTATION\1\DATA\SE19OCT 2023-10-19 16-56-54\23101900
157.3
81.3
224.3
252.0
312.4
404.6
515.0
520.1
561.2
611.6
692.2
742.4
770.3
819.2
863.6
896.4
930.4
970.0
1011.2
1047.4
1071.2
1127.4
1163.0
m/z

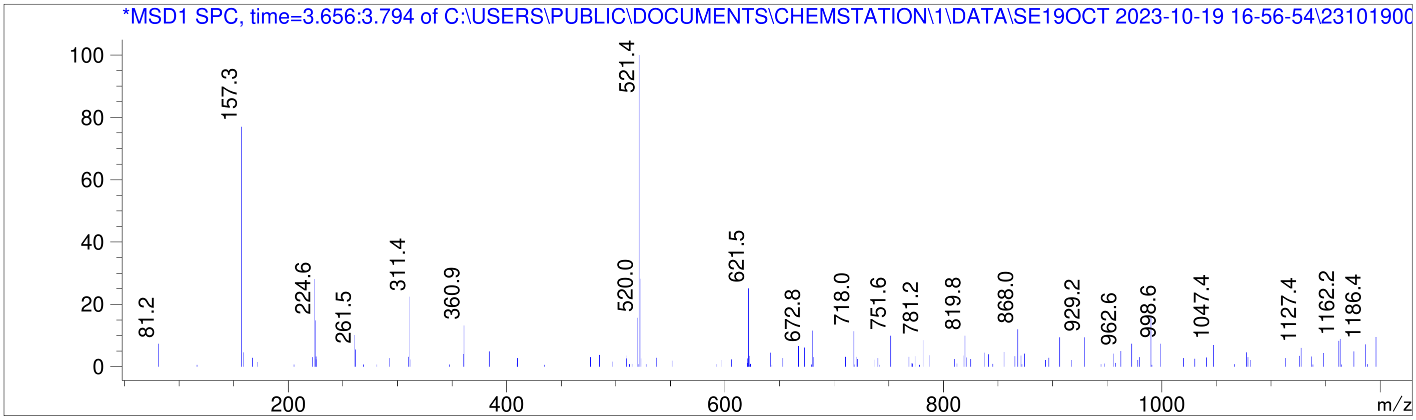
*MSD1 SPC, time=3.656:3.794 of C:\USERS\PUBLIC\DOCUMENTS\CHEMSTATION\1\DATA\SE19OCT 2023-10-19 16-56-54\23101900
521.4
157.3
81.2
224.6
261.5
311.4
360.9
520.0
621.5
672.8
718.0
751.6
781.2
819.8
868.0
929.2
962.6
998.6
1047.4
1127.4
1162.2
1186.4
m/z

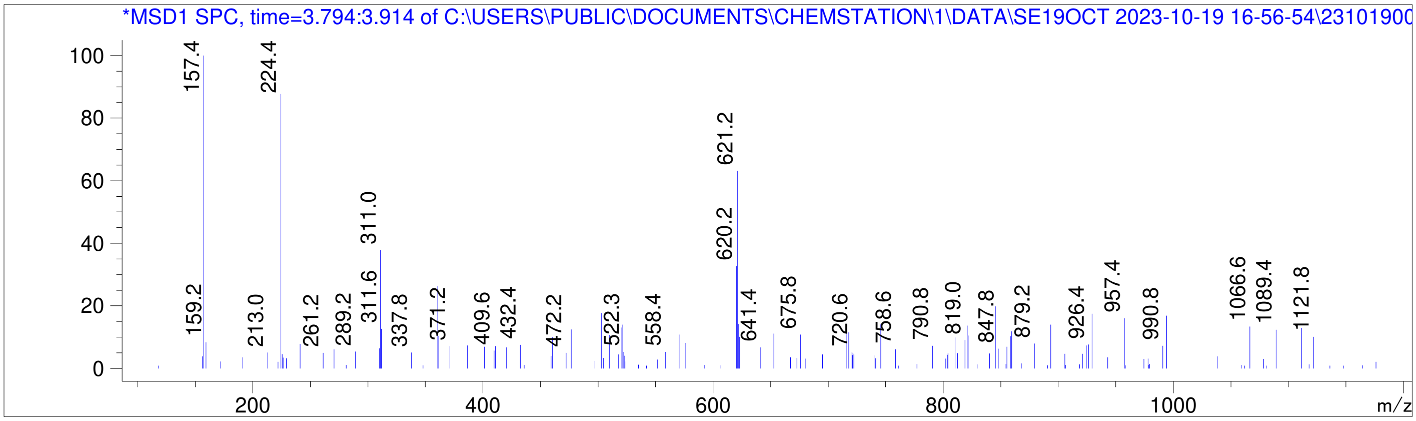
*MSD1 SPC, time=3.794:3.914 of C:\USERS\PUBLIC\DOCUMENTS\CHEMSTATION\1\DATA\SE19OCT 2023-10-19 16-56-54\23101900
100
80
60
40
20
0
157.4
224.4
159.2
213.0
261.2
289.2
311.6
311.0
337.8
371.2
409.6
432.4
472.2
522.3
558.4
620.2
621.2
641.4
675.8
720.6
758.6
790.8
819.0
847.8
879.2
926.4
957.4
990.8
1066.6
1089.4
1121.8
200
400
600
800
1000
m/z

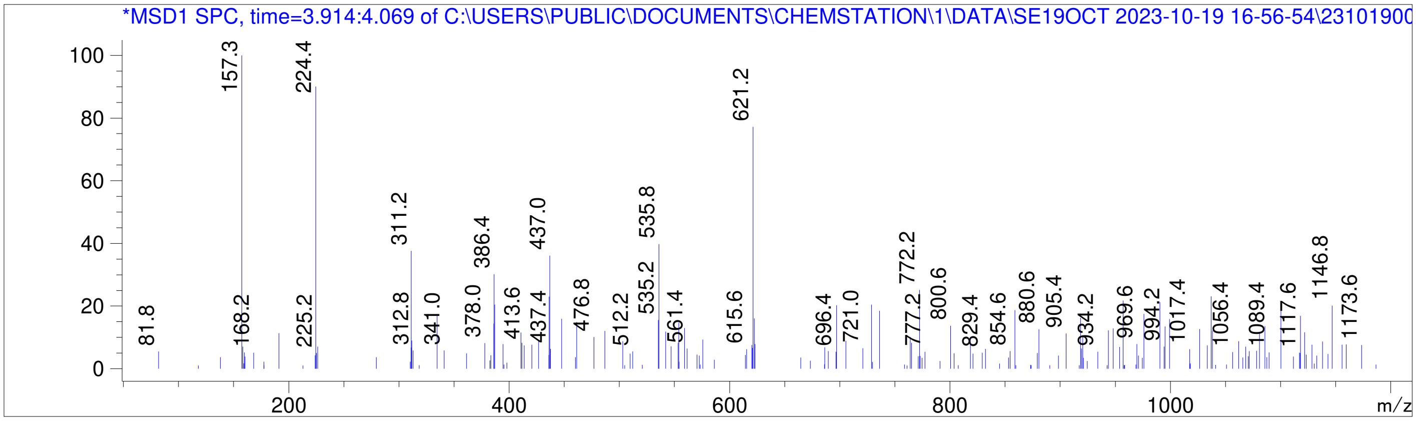
*MSD1 SPC, time=3.914:4.069 of C:\USERS\PUBLIC\DOCUMENTS\CHEMSTATION\1\DATA\SE19OCT 2023-10-19 16-56-54\23101900
100
80
60
40
20
0
81.8
157.3
168.2
224.4
225.2
311.2
312.8
341.0
378.0
386.4
413.6
437.4
437.0
476.8
512.2
535.2
535.8
561.4
615.6
621.2
696.4
721.0
777.2
772.2
800.6
829.4
854.6
880.6
905.4
934.2
969.6
994.2
1017.4
1056.4
1089.4
1117.6
1146.8
1173.6
200
400
600
800
1000
m/z

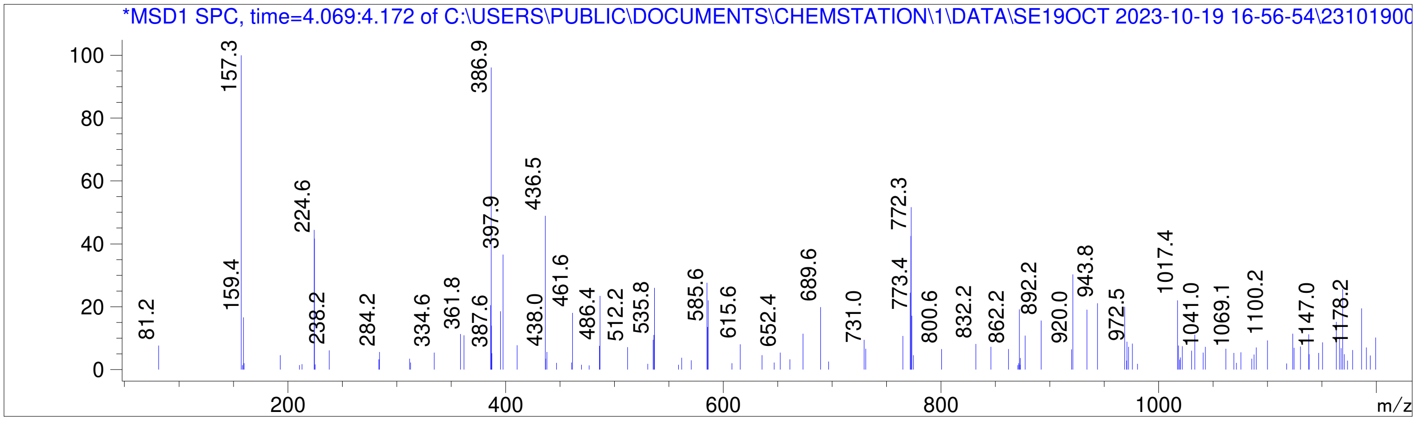
*MSD1 SPC, time=4.069:4.172 of C:\USERS\PUBLIC\DOCUMENTS\CHEMSTATION\1\DATA\SE19OCT 2023-10-19 16-56-54\23101900
100
80
60
40
20
0
81.2
157.3
159.4
224.6
238.2
284.2
334.6
361.8
387.6
386.9
397.9
438.0
436.5
461.6
486.4
512.2
535.8
585.6
615.6
652.4
689.6
731.0
773.4
772.3
800.6
832.2
862.2
892.2
920.0
943.8
972.5
1017.4
1041.0
1069.1
1100.2
1147.0
1178.2
200
400
600
800
1000
m/z

Sample-> CPT22010446-21-C1-80dg-1h

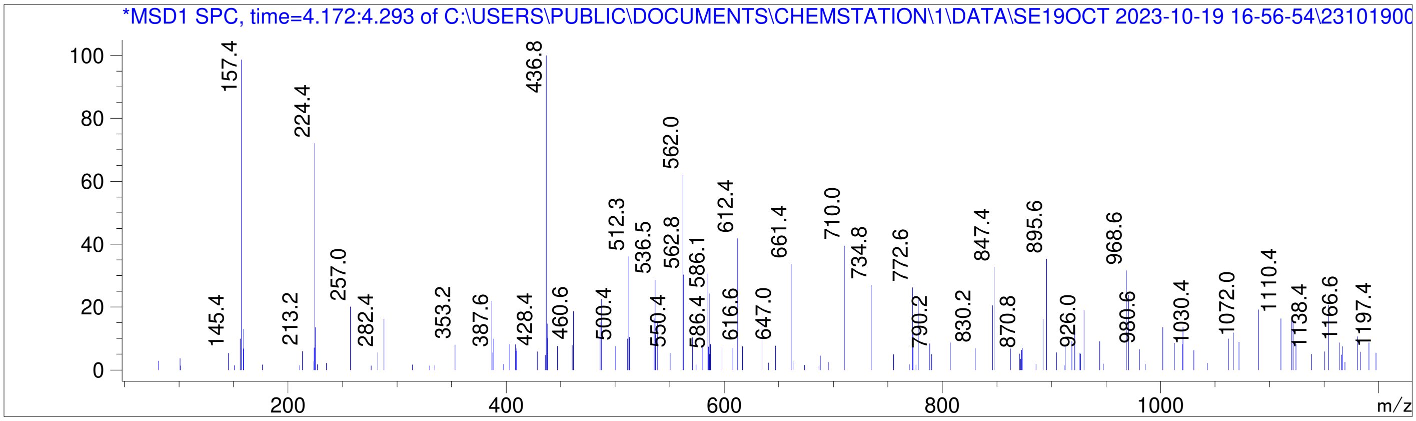

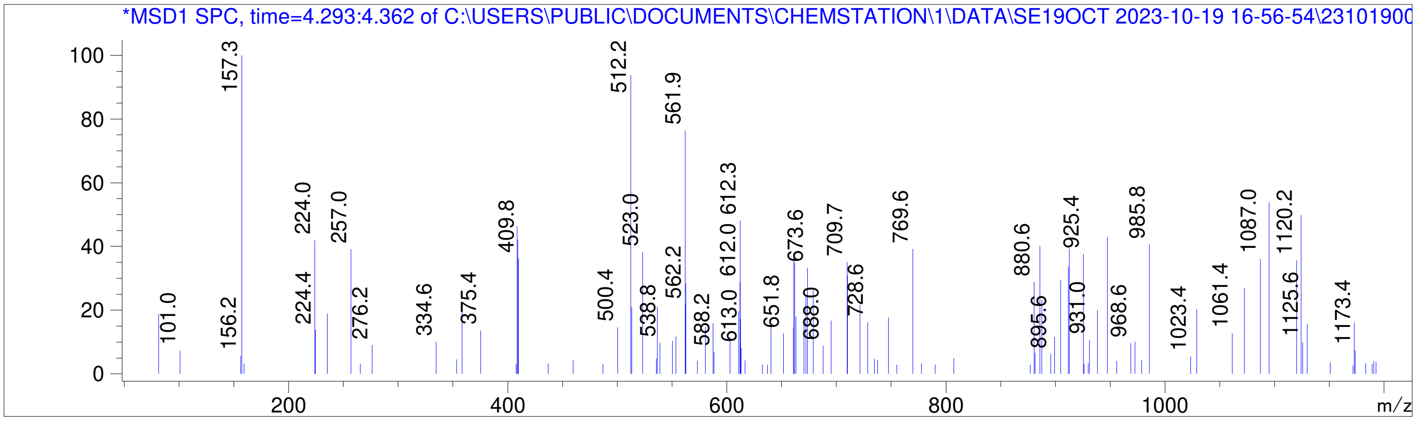

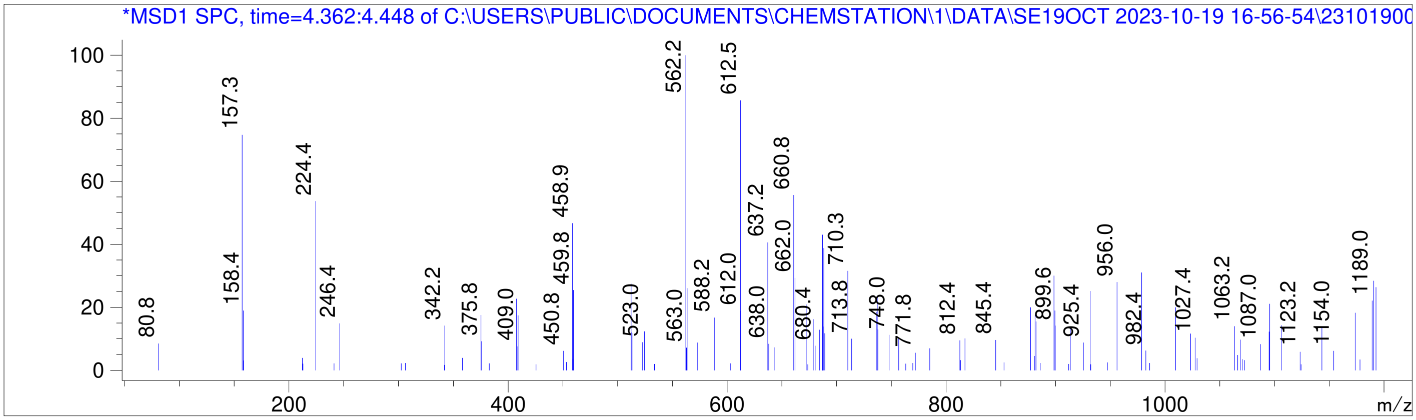

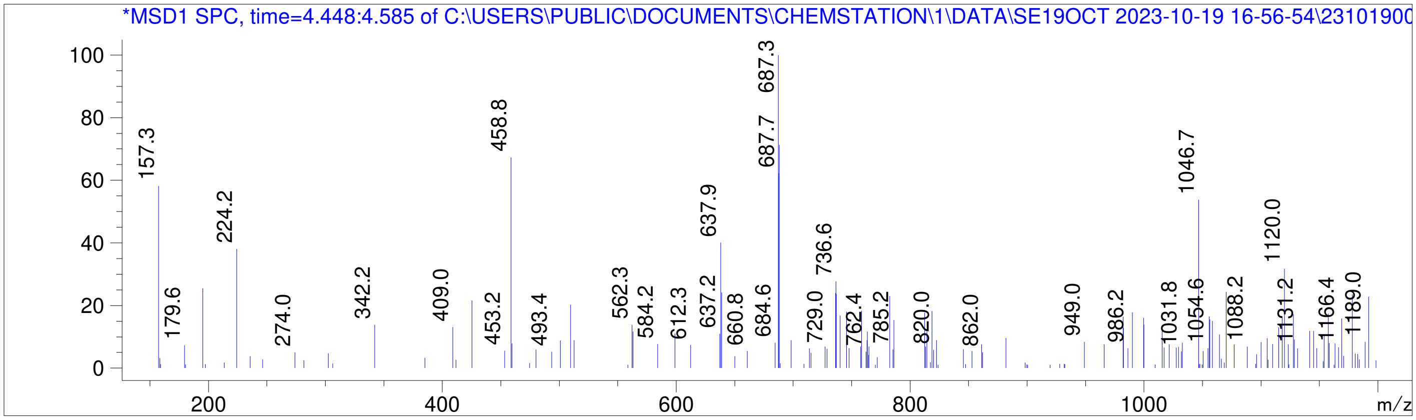

==

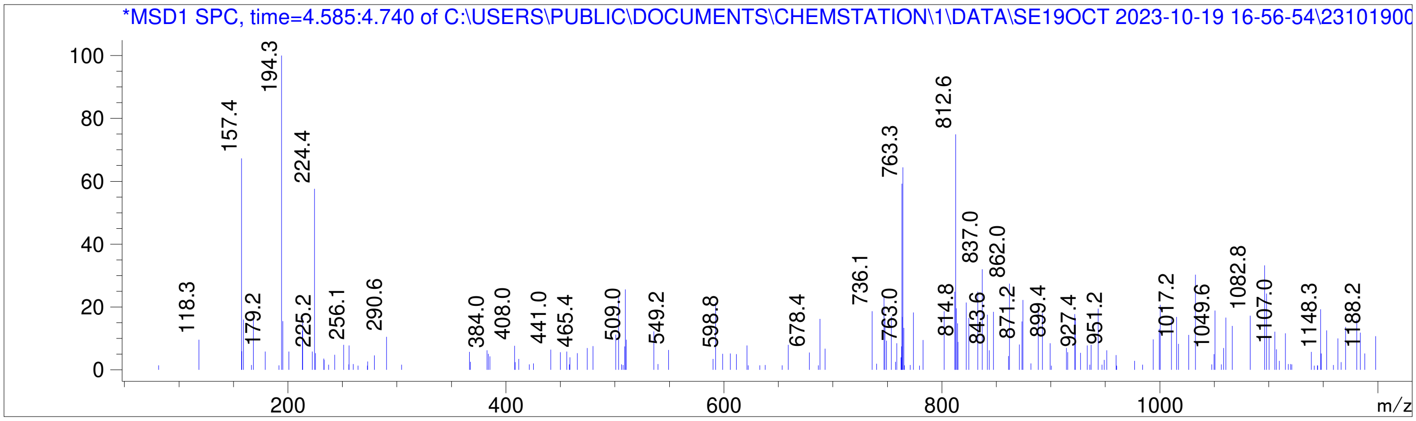
*MSD1 SPC, time=4.585:4.740 of C:\USERS\PUBLIC\DOCUMENTS\CHEMSTATION\1\DATA\SE19OCT 2023-10-19 16-56-54\23101900
100
80
60
40
20
0
118.3
157.4
179.2
194.3
224.4
225.2
256.1
290.6
384.0
408.0
441.0
465.4
509.0
549.2
598.8
678.4
736.1
763.3
763.0
812.6
814.8
837.0
843.6
862.0
871.2
899.4
927.4
951.2
1017.2
1049.6
1082.8
1107.0
1148.3
1188.2
200
400
600
800
1000
m/z
