# Supplementary material for: Astrobiological implications of the stability and reactivity of peptide nucleic acid (PNA) in concentrated sulfuric acid
Source: Sci Adv. 2025 Mar 26;11(13):eadr0006. doi: 10.1126/sciadv.adr0006 (PMC11939054; doi:10.1126/sciadv.adr0006)

Data -> C:\USERS\PUBLIC\DOCUMENTS\CHEMSTATION\1\DATA\SE19OCT 2023-10-19 16-56-54\ ->
Sample-> CPT22010446-21-C1-80dg-24h

===

Injection Date	:	Fri, 20. Oct. 2023	Seq Line	:	45
			Location	:	25
			Inj. Vol.	:	2 µl

Acq. Method : C:\Users\Public\Documents\ChemStation\1\Data\SE19OCT 2023-10-19 16-56-54\22010446 LCMS-6.M
Analysis Method : C:\Users\Public\Documents\ChemStation\1\Data\SE19OCT 2023-10-19 16-56-54\22010446 LCMS-6.M (Sequence Method)
Waters XBridge Phenyl (4.6 * 150 mm; 3.5 µm); 0.05% TFA (aq) / AcN: 100/0 (0.0 min) --> (6.0 min) --> 70/30 (0.0 min) --> (2.0 min) --> 10/90 (2.0 min); Flow: 1.0 ml/min;
MSD1 = positive; MSD2 = negative

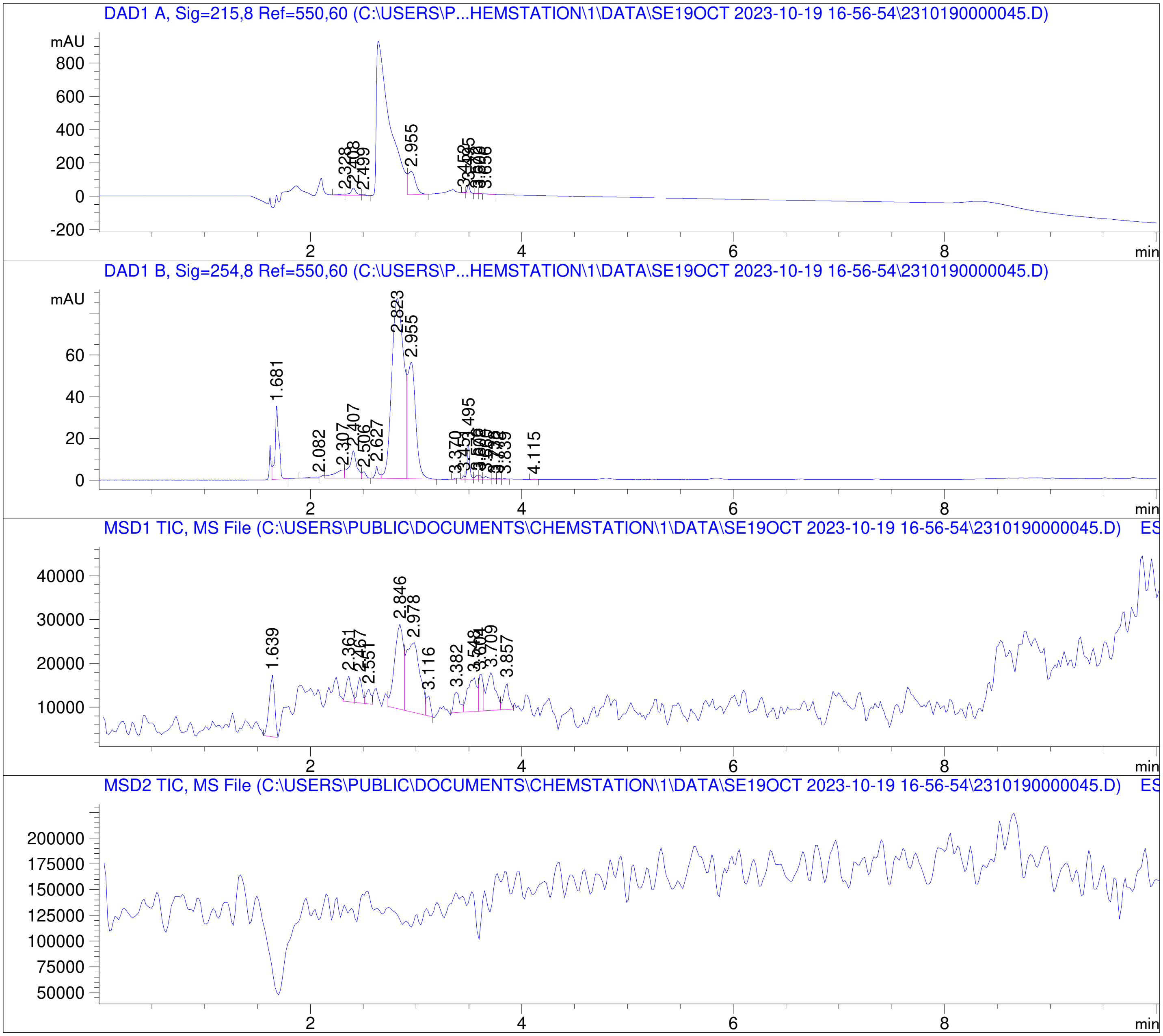

Sample-> CPT22010446-21-C1-80dg-24h

DAD1 A, Sig=215,8 Ref=550,60

Peak #	Ret. Time [min]	Area [mV *s]	Area %
1	2.328	19.577	2.014
2	2.408	153.694	15.808
3	2.499	13.288	1.367
4	2.955	693.709	71.350
5	3.452	4.573	0.470
6	3.495	75.697	7.786
7	3.572	2.398	0.247
8	3.600	3.319	0.341
9	3.656	6.006	0.618

DAD1 B, Sig=254,8 Ref=550,60

Peak #	Ret. Time [min]	Area [mV *s]	Area %
1	1.681	86.669	7.051
2	2.082	4.097	0.333
3	2.307	27.657	2.250
4	2.407	62.900	5.117
5	2.506	7.815	0.636
6	2.627	12.002	0.976
7	2.823	686.462	55.845
8	2.955	294.540	23.961
9	3.370	0.830	0.067
10	3.451	2.687	0.219
11	3.495	28.472	2.316
12	3.576	3.643	0.296
13	3.602	3.381	0.275
14	3.660	4.246	0.345
15	3.738	1.294	0.105
16	3.772	0.996	0.081
17	3.839	0.962	0.078
18	4.115	0.572	0.047

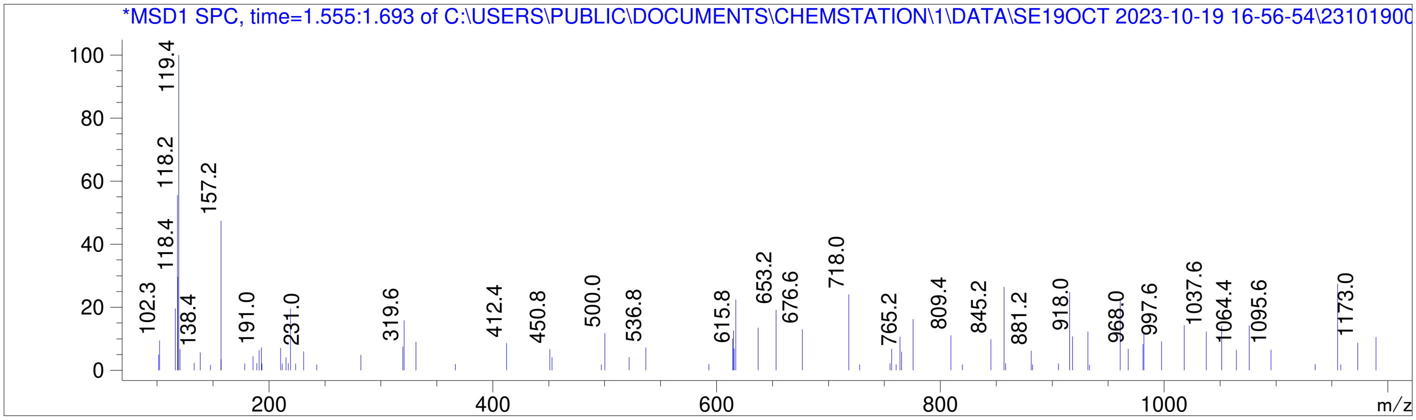
*MSD1 SPC, time=1.555:1.693 of C:\USERS\PUBLIC\DOCUMENTS\CHEMSTATION\1\DATA\SE19OCT 2023-10-19 16-56-54\23101900
119.4
118.2
157.2
118.4
102.3
138.4
191.0
231.0
319.6
412.4
450.8
500.0
536.8
615.8
653.2
676.6
718.0
765.2
809.4
845.2
881.2
918.0
968.0
997.6
1037.6
1064.4
1095.6
1173.0
m/z

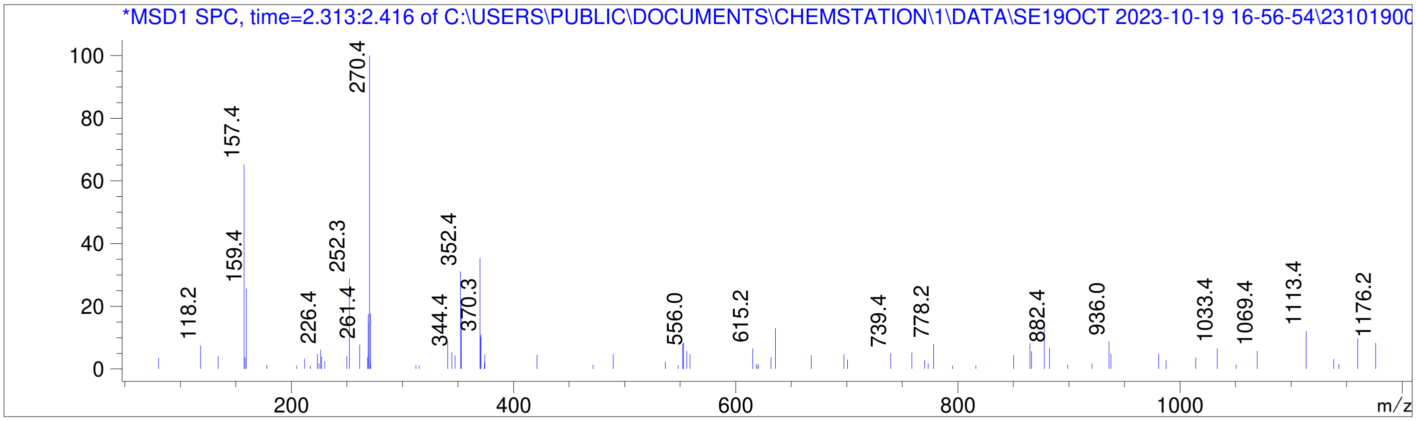
*MSD1 SPC, time=2.313:2.416 of C:\USERS\PUBLIC\DOCUMENTS\CHEMSTATION\1\DATA\SE19OCT 2023-10-19 16-56-54\23101900
270.4
157.4
159.4
252.3
352.4
118.2
226.4
261.4
344.4
370.3
556.0
615.2
739.4
778.2
882.4
936.0
1033.4
1069.4
1113.4
1176.2
m/z

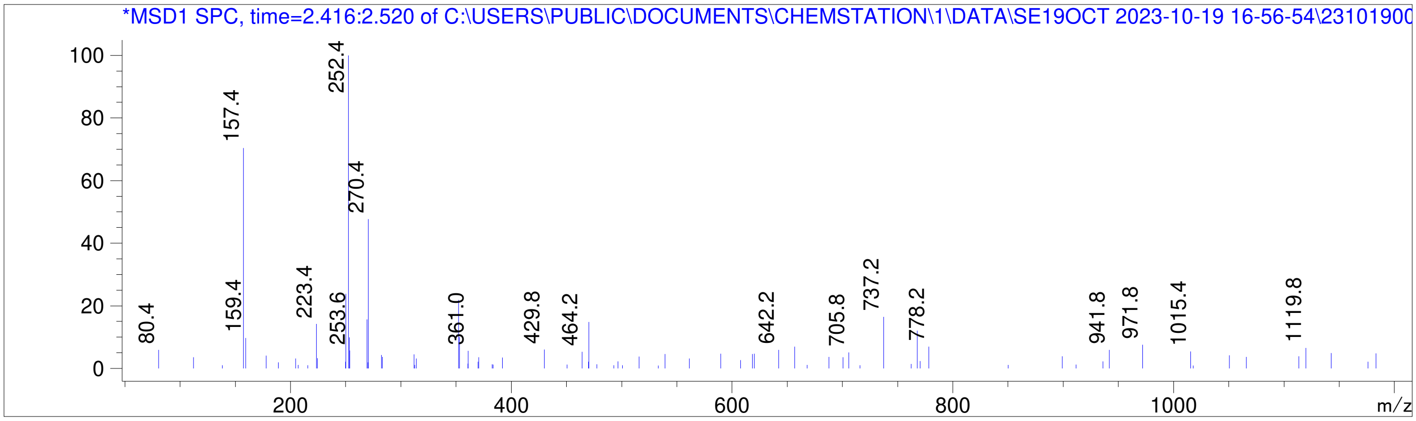
*MSD1 SPC, time=2.416:2.520 of C:\USERS\PUBLIC\DOCUMENTS\CHEMSTATION\1\DATA\SE19OCT 2023-10-19 16-56-54\23101900
252.4
157.4
270.4
80.4
159.4
223.4
253.6
361.0
429.8
464.2
642.2
705.8
737.2
778.2
941.8
971.8
1015.4
1119.8
m/z

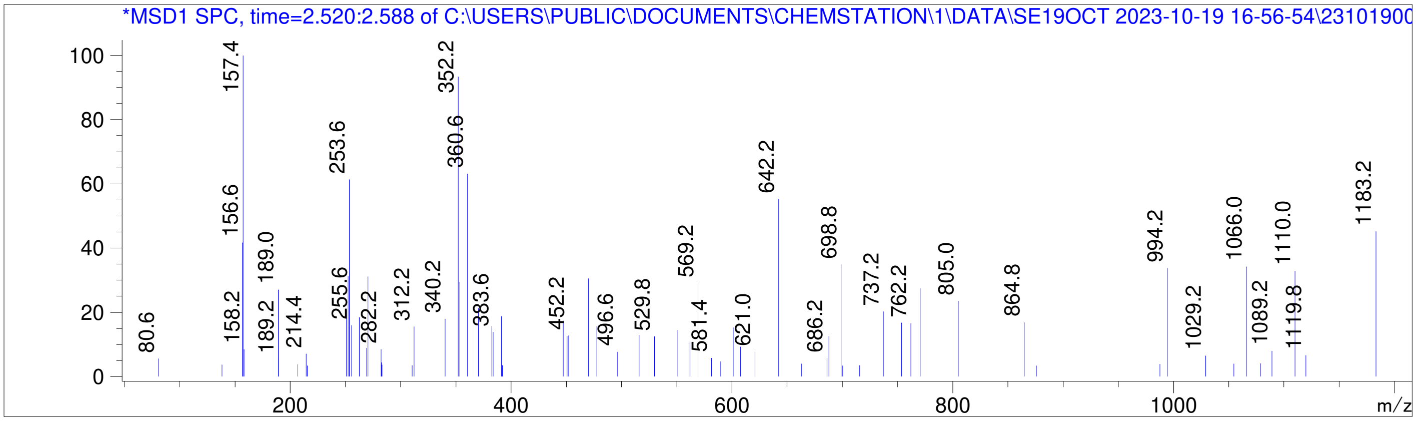
*MSD1 SPC, time=2.520:2.588 of C:\USERS\PUBLIC\DOCUMENTS\CHEMSTATION\1\DATA\SE19OCT 2023-10-19 16-56-54\23101900
157.4
352.2
360.6
253.6
642.2
156.6
189.0
698.8
994.2
1066.0
1110.0
1183.2
569.2
805.0
737.2
762.2
864.8
80.6
158.2
189.2
214.4
255.6
282.2
312.2
340.2
383.6
452.2
496.6
529.8
581.4
621.0
686.2
1029.2
1089.2
1119.8
m/z

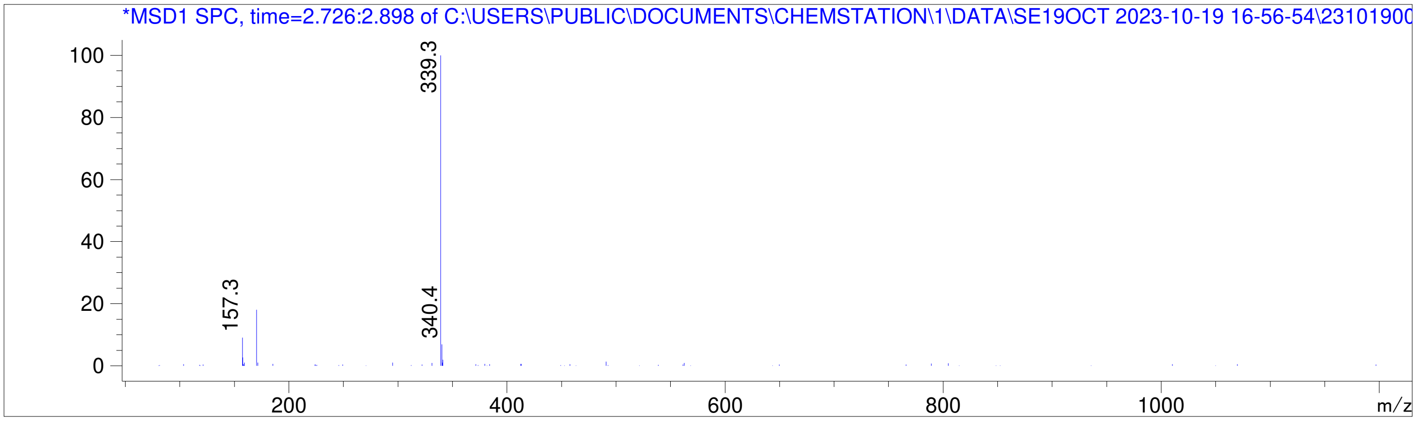
*MSD1 SPC, time=2.726:2.898 of C:\USERS\PUBLIC\DOCUMENTS\CHEMSTATION\1\DATA\SE19OCT 2023-10-19 16-56-54\23101900
339.3
157.3
340.4
m/z

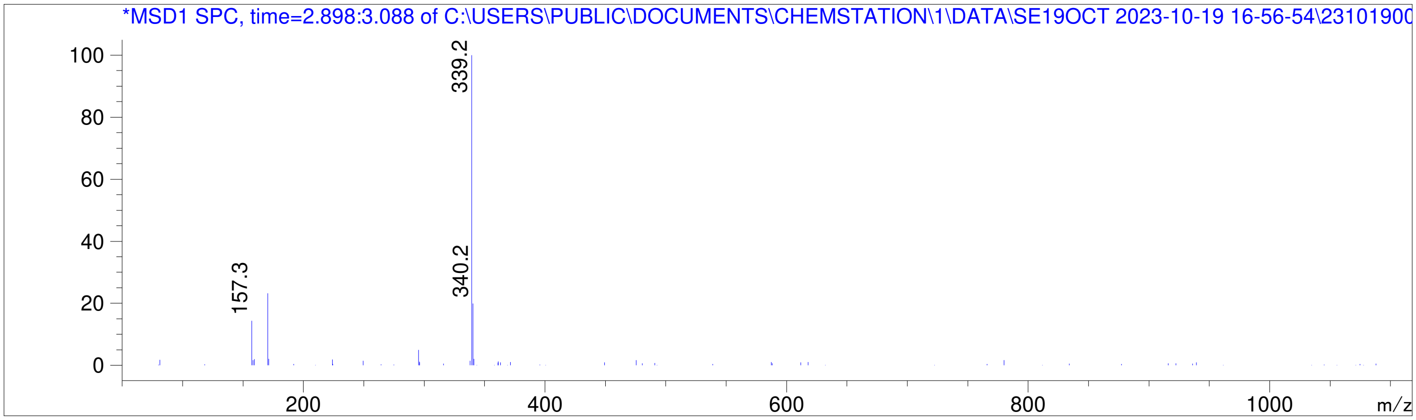
*MSD1 SPC, time=2.898:3.088 of C:\USERS\PUBLIC\DOCUMENTS\CHEMSTATION\1\DATA\SE19OCT 2023-10-19 16-56-54\23101900
339.2
157.3
340.2
m/z

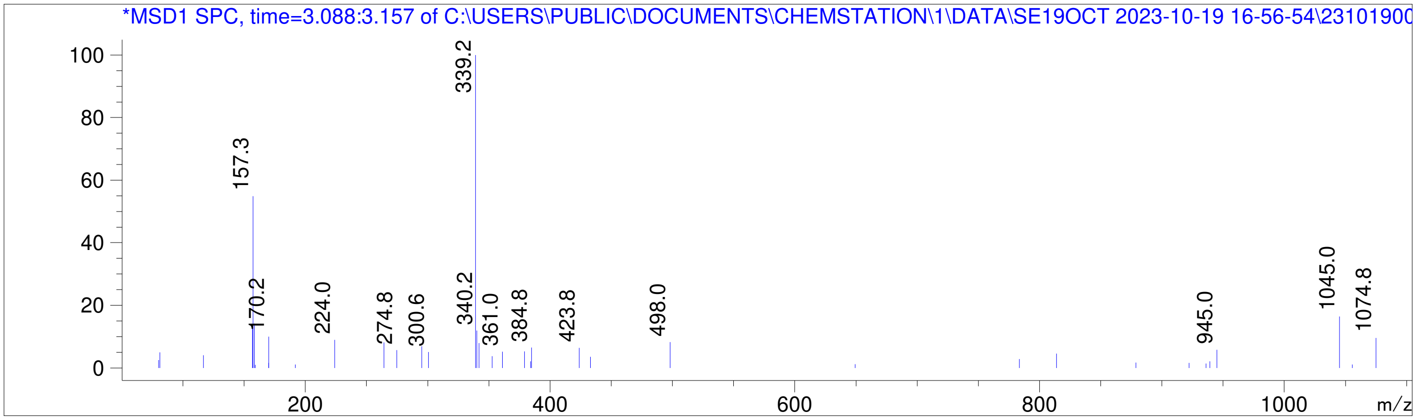
*MSD1 SPC, time=3.088:3.157 of C:\USERS\PUBLIC\DOCUMENTS\CHEMSTATION\1\DATA\SE19OCT 2023-10-19 16-56-54\23101900
339.2
157.3
170.2
224.0
274.8
300.6
340.2
361.0
384.8
423.8
498.0
945.0
1045.0
1074.8
m/z

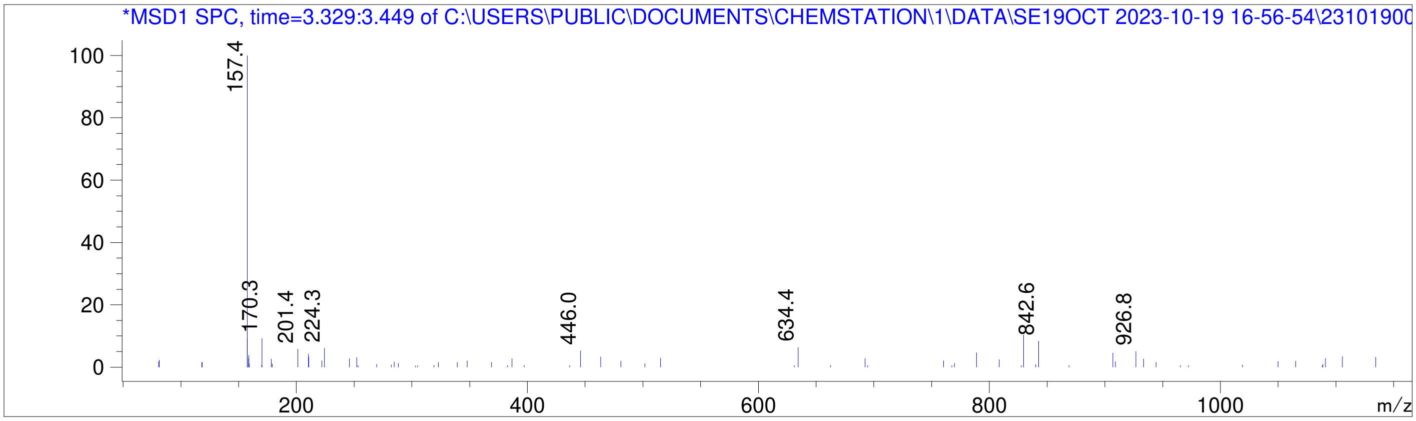
*MSD1 SPC, time=3.329:3.449 of C:\USERS\PUBLIC\DOCUMENTS\CHEMSTATION\1\DATA\SE19OCT 2023-10-19 16-56-54\23101900
157.4
170.3
201.4
224.3
446.0
634.4
842.6
926.8
m/z

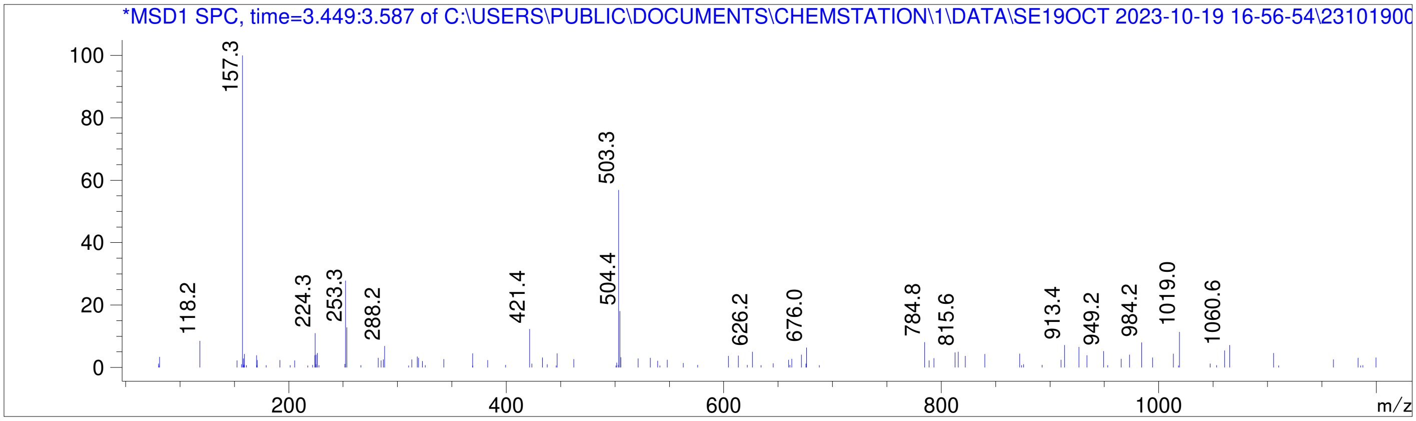
*MSD1 SPC, time=3.449:3.587 of C:\USERS\PUBLIC\DOCUMENTS\CHEMSTATION\1\DATA\SE19OCT 2023-10-19 16-56-54\23101900
157.3
118.2
224.3
253.3
288.2
421.4
503.3
504.4
626.2
676.0
784.8
815.6
913.4
949.2
984.2
1019.0
1060.6
m/z

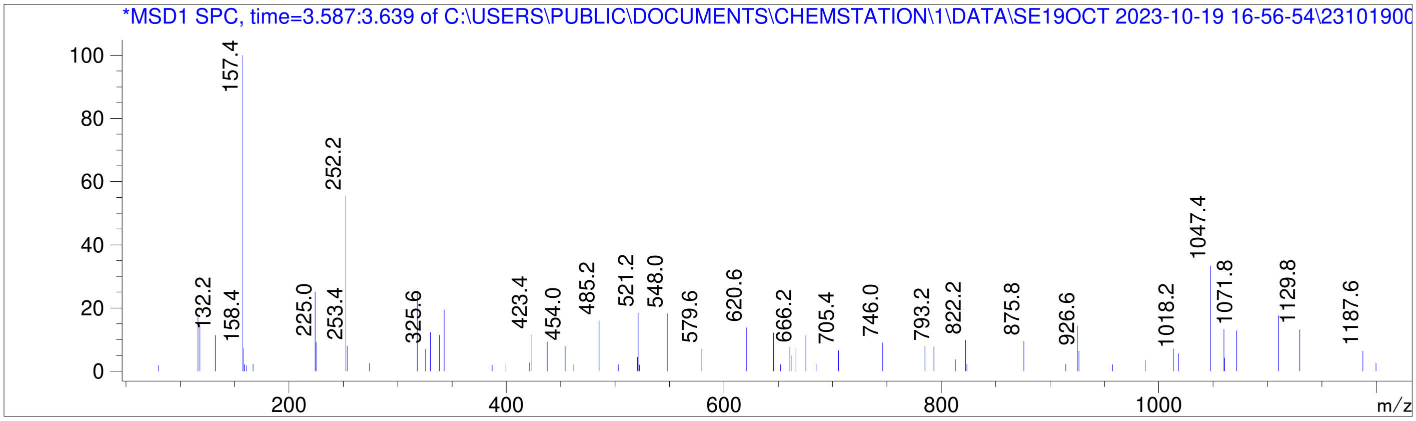
*MSD1 SPC, time=3.587:3.639 of C:\USERS\PUBLIC\DOCUMENTS\CHEMSTATION\1\DATA\SE19OCT 2023-10-19 16-56-54\23101900
157.4
132.2
158.4
225.0
252.2
253.4
325.6
423.4
454.0
485.2
521.2
548.0
579.6
620.6
666.2
705.4
746.0
793.2
822.2
875.8
926.6
1018.2
1047.4
1071.8
1129.8
1187.6
m/z

Sample-> CPT22010446-21-C1-80dg-24h

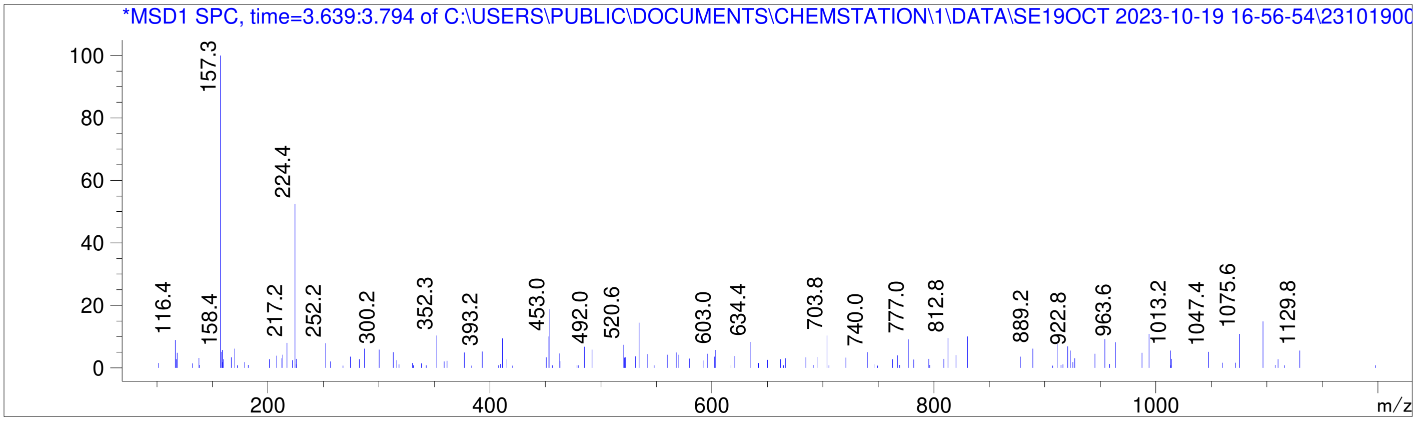

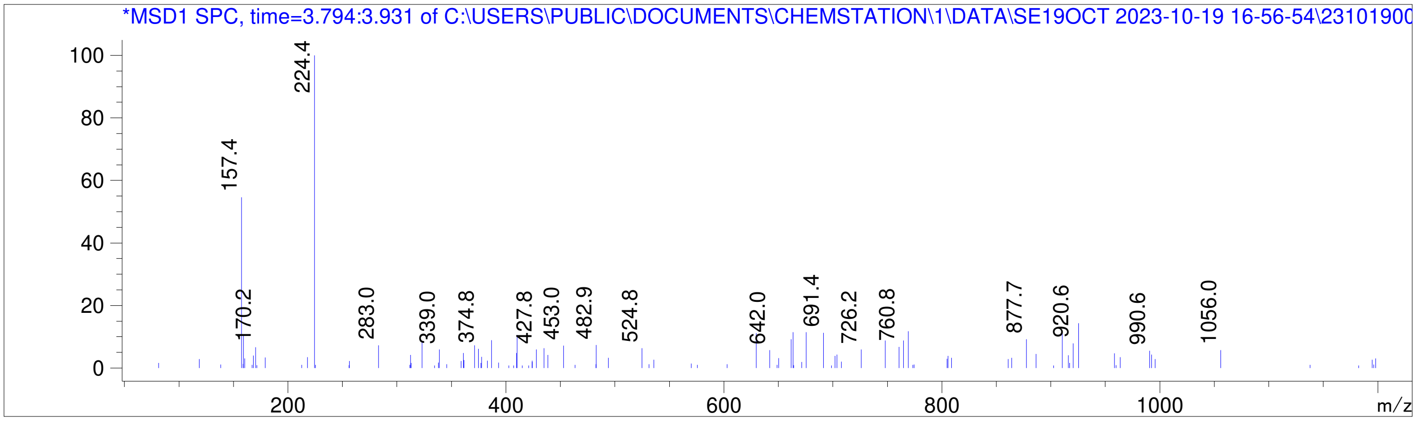

Supplement: Supplementary file 2 — Data S1 and S2 [file sciadv.adr0006_data_s1_and_s2.zip › Supplementary Dataset 1-LCMS DATA/LCMS PNA Hexamers A-T/LCMS C6 50C_80C/80C/24h/CPT22010446-21-C1-80dg-24h.pdf]
