# Supplementary material for: Astrobiological implications of the stability and reactivity of peptide nucleic acid (PNA) in concentrated sulfuric acid
Source: Sci Adv. 2025 Mar 26;11(13):eadr0006. doi: 10.1126/sciadv.adr0006 (PMC11939054; doi:10.1126/sciadv.adr0006)
Supplement: Supplementary file 2 — Data S1 and S2 [file sciadv.adr0006_data_s1_and_s2.zip › Supplementary Dataset 1-LCMS DATA/LCMS PNA Hexamers A-T/LCMS C6 50C_80C/80C/24h/CPT22010446-21-C2-80deg-24h.pdf]

Injection Date : Wed, 8. Nov. 2023 | Seq Line : 40
Location : 77
Inj. Vol. : 2 µl

Acq. Method : C:\Users\Public\Documents\ChemStation\1\Data\SE07NOV 2023-11-07 14-56-21\22010446 LCMS-6.M
Analysis Method : C:\Users\Public\Documents\ChemStation\1\Data\SE07NOV 2023-11-07 14-56-21\22010446 LCMS-6.M (Sequence Method)
Waters XBridge Phenyl (4.6 * 150 mm; 3.5 µm); 0.05% TFA (aq) / AcN: 100/0 (0.0 min) --> (6.0 min) --> 70/30 (0.0 min) --> (2.0 min) --> 10/90 (2.0 min); Flow: 1.0 ml/min;
MSD1 = positive; MSD2 = negative

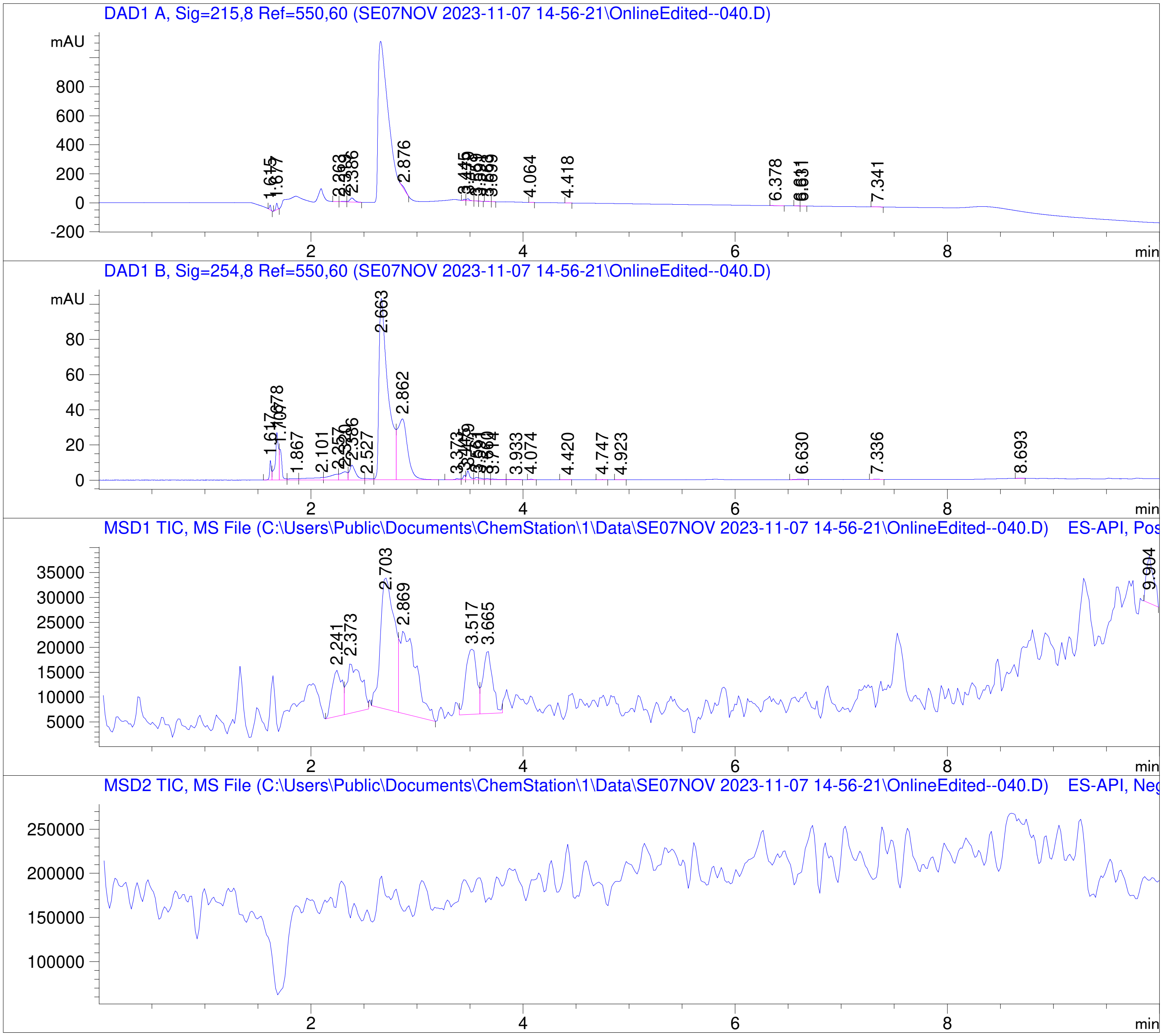

DAD1 A, Sig=215,8 Ref=550,60

Peak #	Ret. Time [min]	Area [mV *s]	Area %
1	1.615	28.967	11.365
2	1.677	53.249	20.893
3	2.263	2.518	0.988
4	2.319	11.590	4.547
5	2.386	85.080	33.382
6	2.876	25.596	10.043
7	3.445	10.655	4.180
8	3.479	23.705	9.301
9	3.559	2.679	1.051
10	3.591	0.769	0.302
11	3.663	1.994	0.782
12	3.699	0.227	0.089
13	4.064	0.656	0.257
14	4.418	0.934	0.366
15	6.378	0.727	0.285
16	6.611	1.090	0.428
17	6.631	1.313	0.515
18	7.341	3.122	1.225

DAD1 B, Sig=254,8 Ref=550,60

Peak #	Ret. Time [min]	Area [mV *s]	Area %
1	1.617	12.260	1.148
2	1.678	56.748	5.313
3	1.707	28.746	2.691
4	1.867	4.923	0.461
5	2.101	14.692	1.375
6	2.257	20.001	1.872
7	2.320	21.900	2.050
8	2.386	34.908	3.268
9	2.527	3.601	0.337
10	2.663	612.422	57.333
11	2.862	229.913	21.524
12	3.373	1.630	0.153
13	3.445	3.725	0.349
14	3.479	10.245	0.959
15	3.561	2.863	0.268
16	3.591	2.147	0.201
17	3.660	1.689	0.158
18	3.714	1.675	0.157
19	3.933	0.786	0.074
20	4.074	0.459	0.043
21	4.420	0.180	0.017
22	4.747	0.141	0.013
23	4.923	0.103	0.010
24	6.630	1.082	0.101
25	7.336	0.908	0.085
26	8.693	0.430	0.040

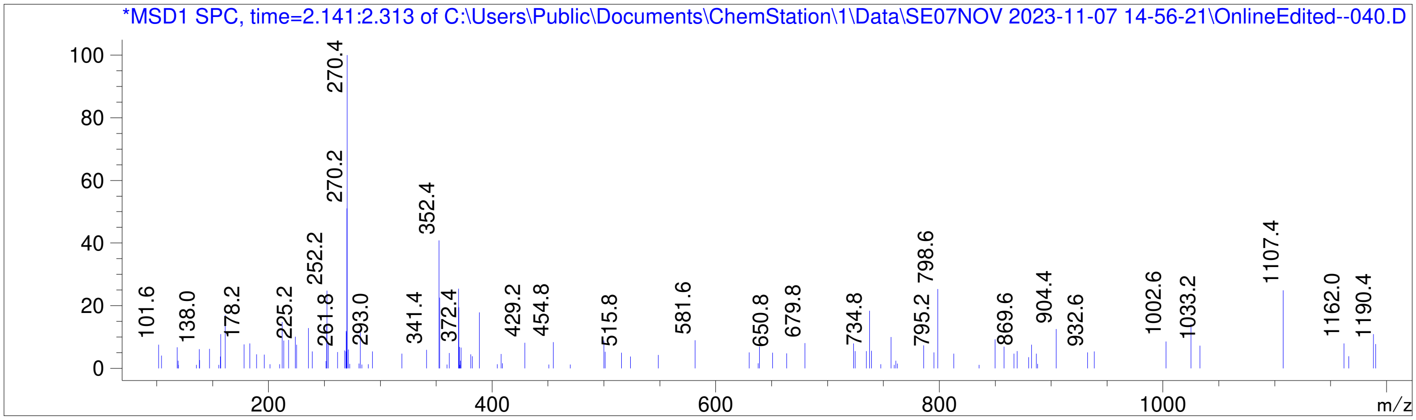
*MSD1 SPC, time=2.141:2.313 of C:\Users\Public\Documents\ChemStation\1\Data\SE07NOV 2023-11-07 14-56-21\OnlineEdited--040.D
270.4
270.2
352.4
252.2
101.6
138.0
178.2
225.2
261.8
293.0
341.4
372.4
429.2
454.8
515.8
581.6
650.8
679.8
734.8
795.2
798.6
869.6
904.4
932.6
1002.6
1033.2
1107.4
1162.0
1190.4
m/z

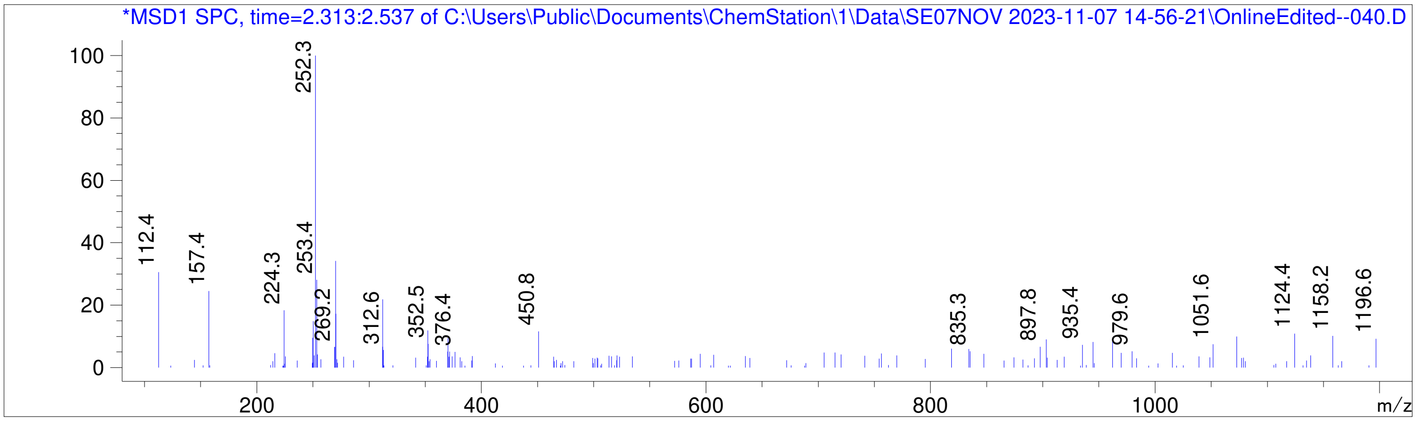
*MSD1 SPC, time=2.313:2.537 of C:\Users\Public\Documents\ChemStation\1\Data\SE07NOV 2023-11-07 14-56-21\OnlineEdited--040.D
252.3
112.4
157.4
224.3
253.4
269.2
312.6
352.5
376.4
450.8
835.3
897.8
935.4
979.6
1051.6
1124.4
1158.2
1196.6
m/z

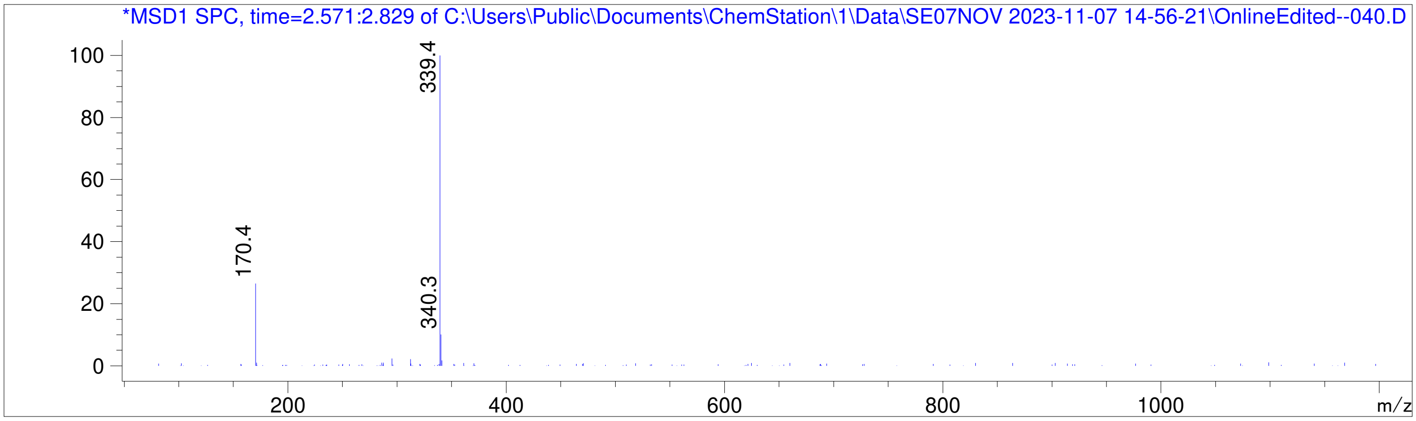
*MSD1 SPC, time=2.571:2.829 of C:\Users\Public\Documents\ChemStation\1\Data\SE07NOV 2023-11-07 14-56-21\OnlineEdited--040.D
339.4
170.4
340.3
m/z

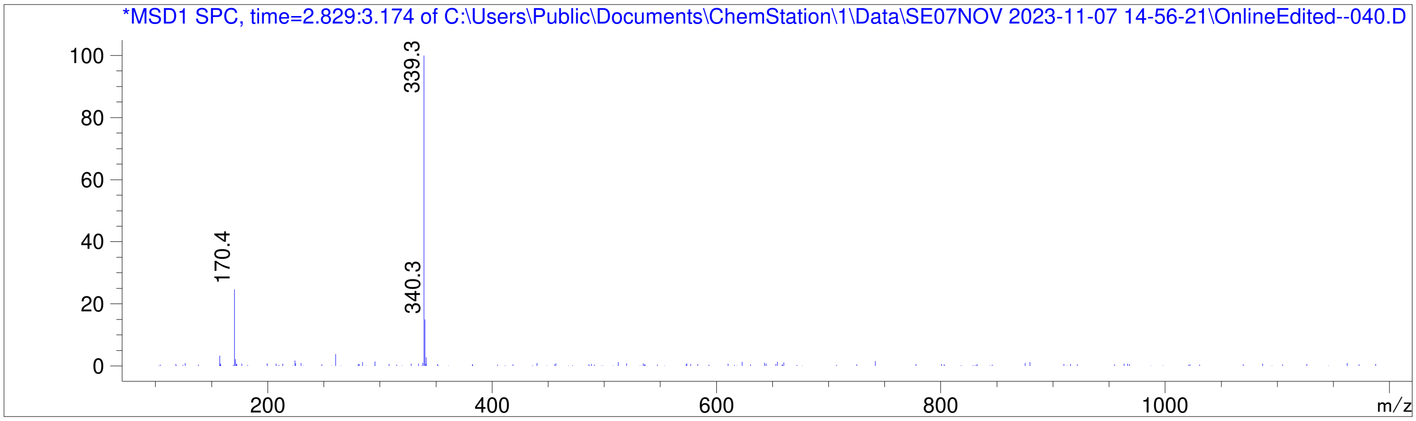
*MSD1 SPC, time=2.829:3.174 of C:\Users\Public\Documents\ChemStation\1\Data\SE07NOV 2023-11-07 14-56-21\OnlineEdited--040.D
339.3
170.4
340.3
m/z

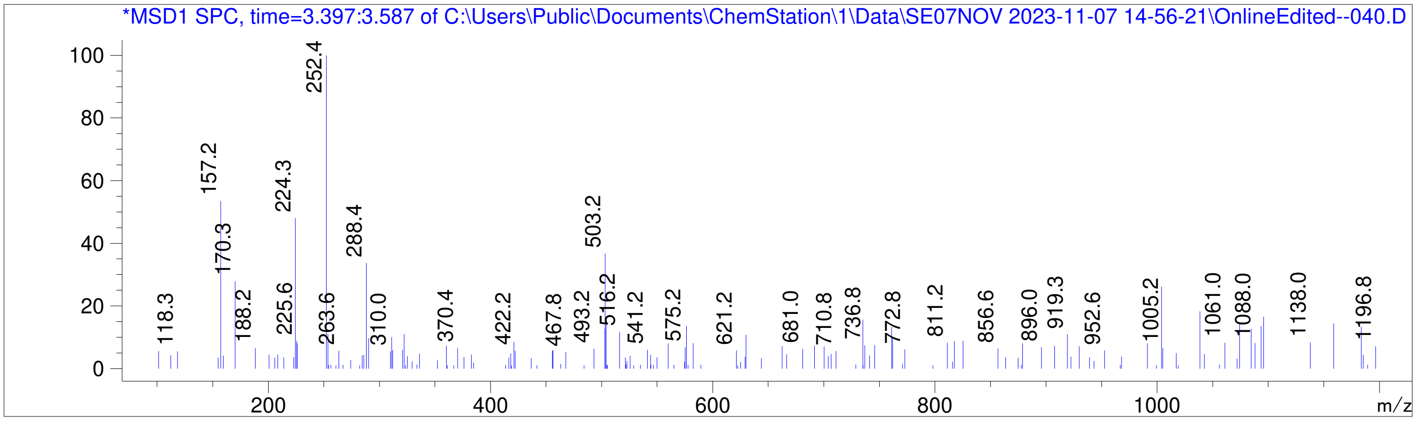
*MSD1 SPC, time=3.397:3.587 of C:\Users\Public\Documents\ChemStation\1\Data\SE07NOV 2023-11-07 14-56-21\OnlineEdited--040.D
252.4
157.2
224.3
288.4
503.2
170.3
118.3
188.2
225.6
263.6
310.0
370.4
422.2
467.8
493.2
516.2
541.2
575.2
621.2
681.0
710.8
736.8
772.8
811.2
856.6
896.0
919.3
952.6
1005.2
1061.0
1088.0
1138.0
1196.8
m/z

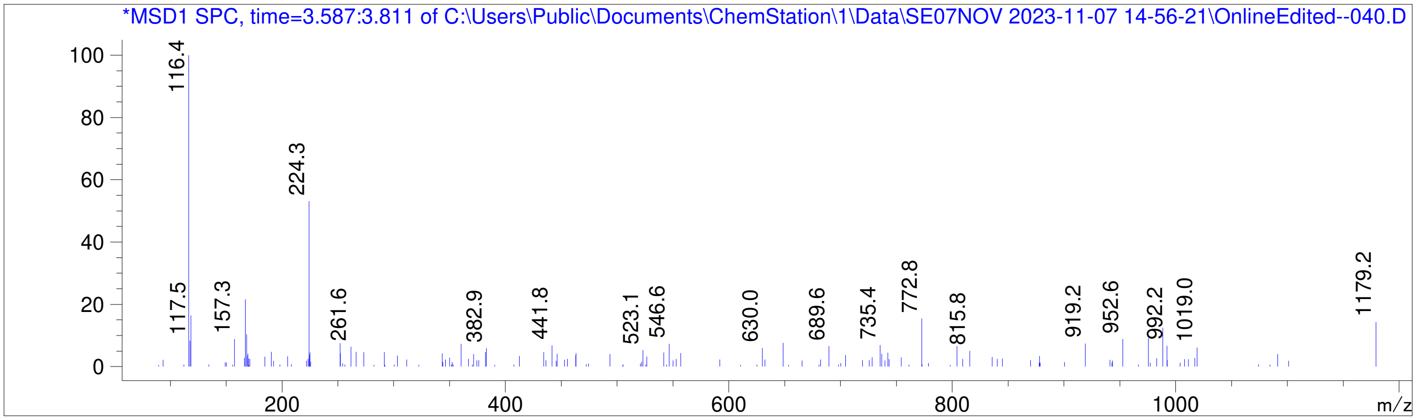
*MSD1 SPC, time=3.587:3.811 of C:\Users\Public\Documents\ChemStation\1\Data\SE07NOV 2023-11-07 14-56-21\OnlineEdited--040.D
116.4
224.3
117.5
157.3
261.6
382.9
441.8
523.1
546.6
630.0
689.6
735.4
772.8
815.8
919.2
952.6
992.2
1019.0
1179.2
m/z

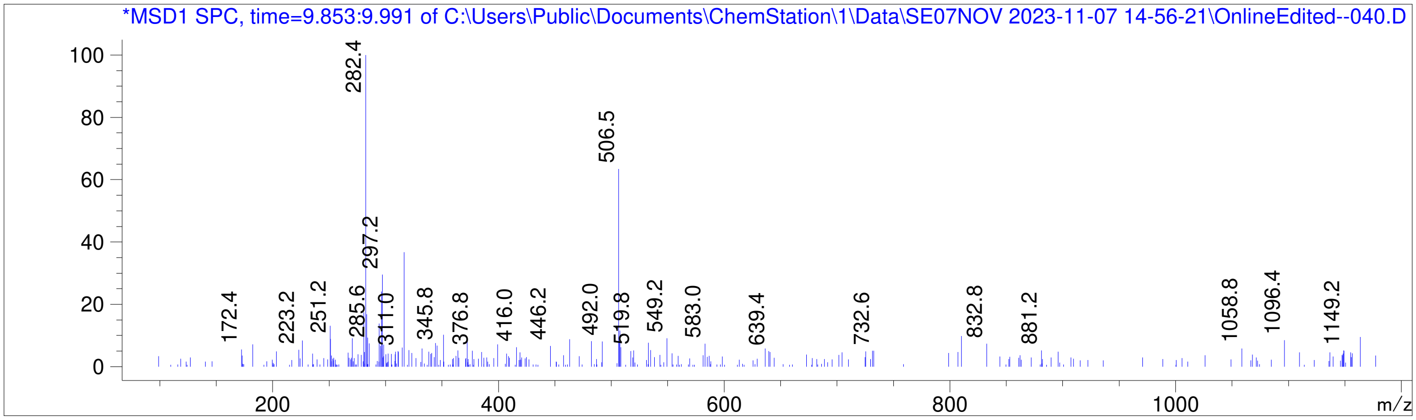
*MSD1 SPC, time=9.853:9.991 of C:\Users\Public\Documents\ChemStation\1\Data\SE07NOV 2023-11-07 14-56-21\OnlineEdited--040.D
282.4
506.5
297.2
172.4
223.2
251.2
285.6
311.0
345.8
376.8
416.0
446.2
492.0
519.8
549.2
583.0
639.4
732.6
832.8
881.2
1058.8
1096.4
1149.2
m/z
