# Supplementary material for: Astrobiological implications of the stability and reactivity of peptide nucleic acid (PNA) in concentrated sulfuric acid
Source: Sci Adv. 2025 Mar 26;11(13):eadr0006. doi: 10.1126/sciadv.adr0006 (PMC11939054; doi:10.1126/sciadv.adr0006)
Supplement: Supplementary file 2 — Data S1 and S2 [file sciadv.adr0006_data_s1_and_s2.zip › Supplementary Dataset 1-LCMS DATA/LCMS PNA Hexamers A-T/LCMS C6 RT/14d/CPT22010446-21-C-1-14d.pdf]

===

Injection Date : Mon, 9. Oct. 2023		Seq Line :	17
		Location :	86
		Inj. Vol. :	2 µl

Acq. Method : C:\Users\Public\Documents\ChemStation\1\Data\SE09OCT 2023-10-09 14-54-12\22010446 LCMS-6.M

Analysis Method : C:\Users\Public\Documents\ChemStation\1\Data\SE09OCT 2023-10-09 14-54-12\22010446 LCMS-6.M (Sequence Method)

Waters XBridge Phenyl (4.6 * 150 mm; 3.5 µm); 0.05% TFA (aq) / AcN: 100/0 (0.0 min) --> (6.0 min) --> 70/30 (0.0 min) --> (2.0 min) --> 10/90 (2.0 min); Flow: 1.0 ml/min; MSD1 = positive; MSD2 = negative

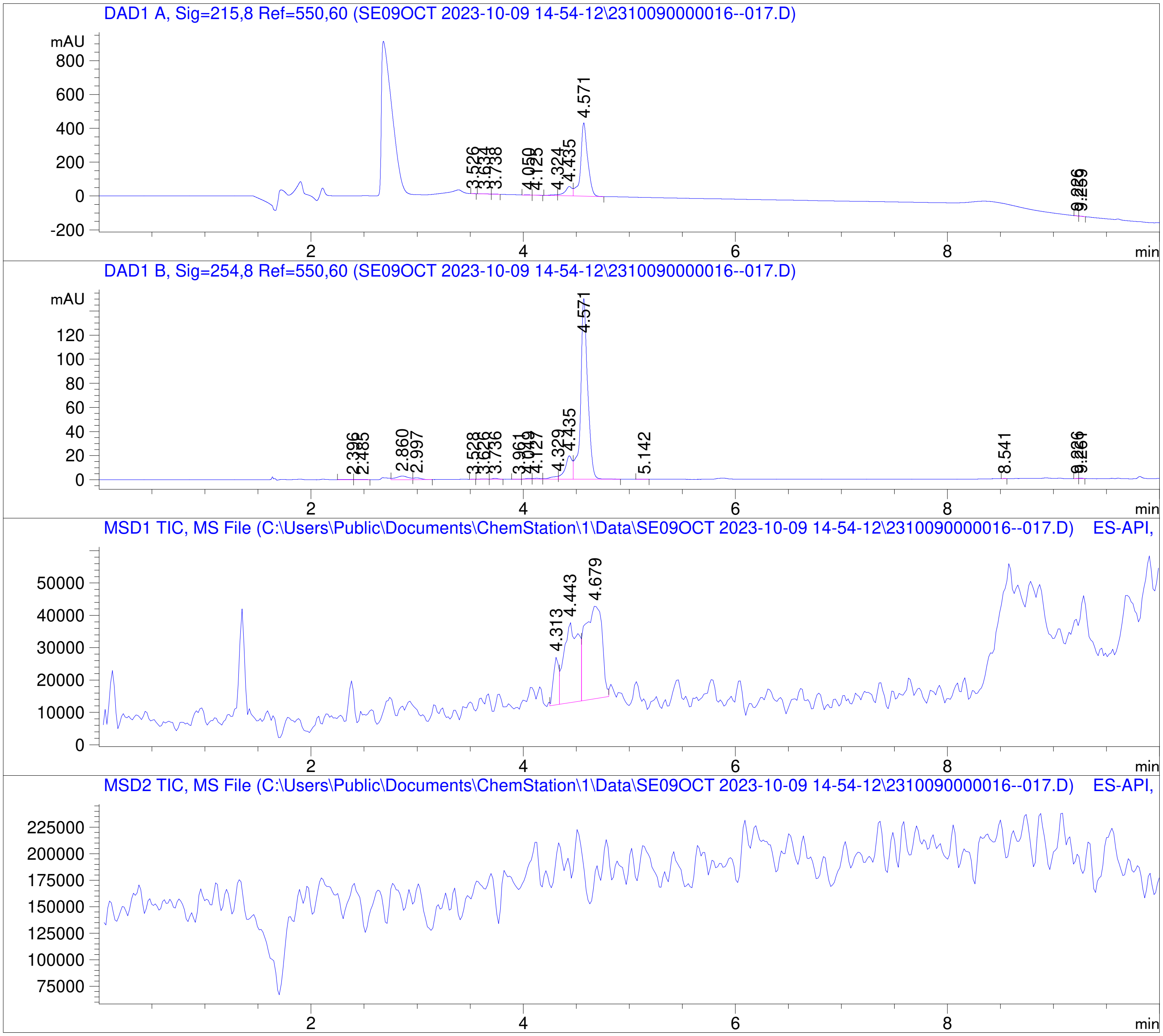

DAD1 A, Sig=215,8 Ref=550,60

Peak #	Ret. Time [min]	Area [mV *s]	Area %
1	3.526	0.604	0.026
2	3.634	5.598	0.242
3	3.738	3.492	0.151
4	4.050	4.020	0.174
5	4.125	5.136	0.222
6	4.324	25.123	1.085
7	4.435	261.069	11.271
8	4.571	2007.363	86.660
9	9.226	1.215	0.052
10	9.259	2.750	0.119

DAD1 B, Sig=254,8 Ref=550,60

Peak #	Ret. Time [min]	Area [mV *s]	Area %
1	2.396	0.955	0.113
2	2.485	1.522	0.180
3	2.860	23.942	2.830
4	2.997	7.258	0.858
5	3.528	0.207	0.024
6	3.626	1.452	0.172
7	3.736	2.567	0.303
8	3.961	0.393	0.046
9	4.049	2.942	0.348
10	4.127	3.860	0.456
11	4.329	12.064	1.426
12	4.435	91.500	10.815
13	4.571	695.361	82.190
14	5.142	0.180	0.021
15	8.541	0.307	0.036
16	9.226	0.466	0.055
17	9.261	1.069	0.126

==

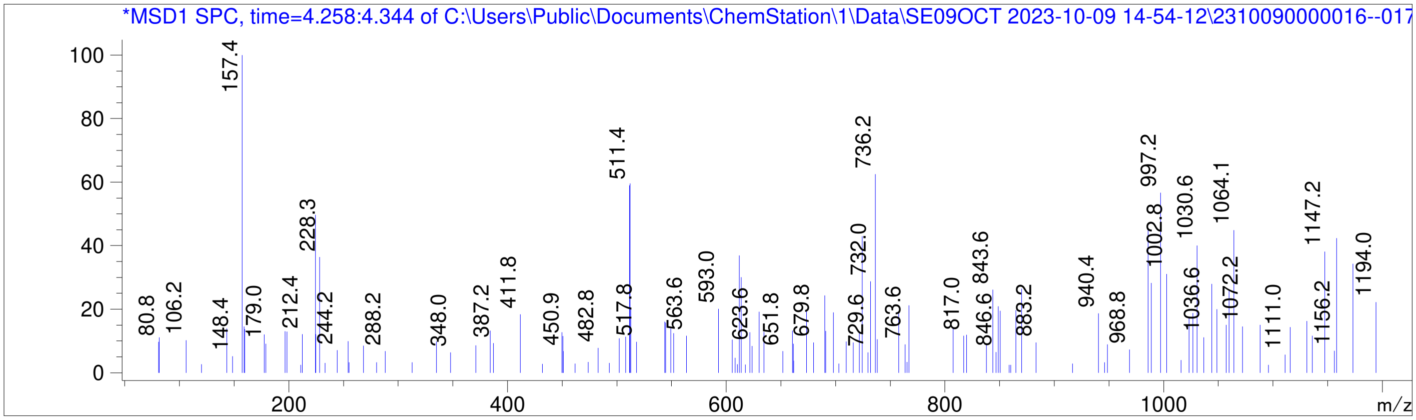
*MSD1 SPC, time=4.258:4.344 of C:\Users\Public\Documents\ChemStation\1\Data\SE09OCT 2023-10-09 14-54-12\2310090000016--017
157.4
511.4
736.2
997.2
1064.1
1030.6
1147.2
1002.8
1194.0
228.3
593.0
732.0
843.6
940.4
411.8
212.4
179.0
106.2
80.8
148.4
244.2
288.2
348.0
387.2
450.9
482.8
517.8
563.6
623.6
651.8
679.8
729.6
763.6
817.0
846.6
883.2
968.8
1036.6
1072.2
1111.0
1156.2
m/z

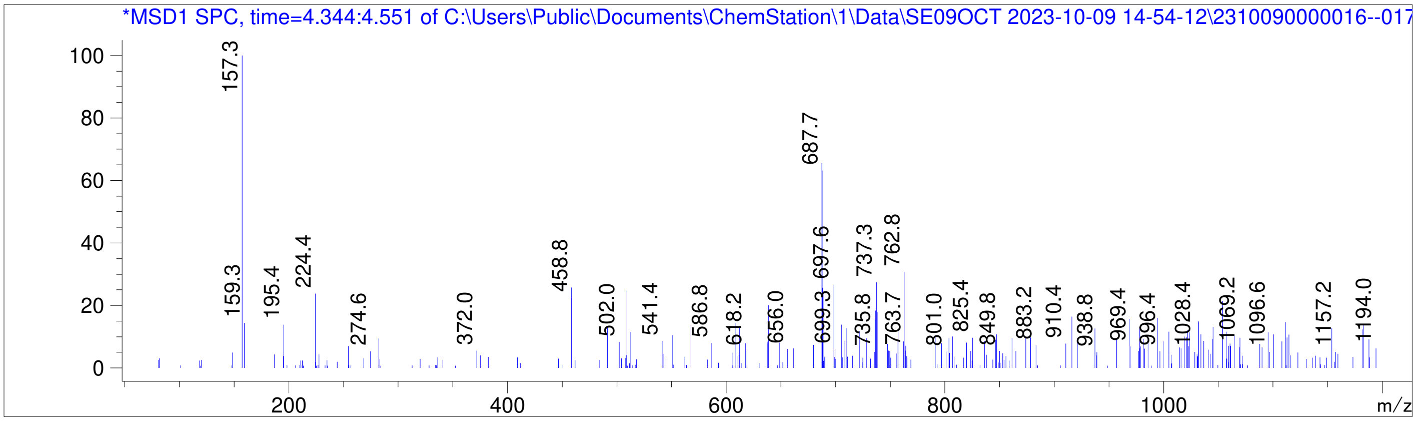
*MSD1 SPC, time=4.344:4.551 of C:\Users\Public\Documents\ChemStation\1\Data\SE09OCT 2023-10-09 14-54-12\2310090000016--017
157.3
687.7
697.6
737.3
762.8
458.8
224.4
159.3
195.4
274.6
372.0
502.0
541.4
586.8
618.2
656.0
699.3
735.8
763.7
801.0
825.4
849.8
883.2
910.4
938.8
969.4
996.4
1028.4
1069.2
1096.6
1157.2
1194.0
m/z

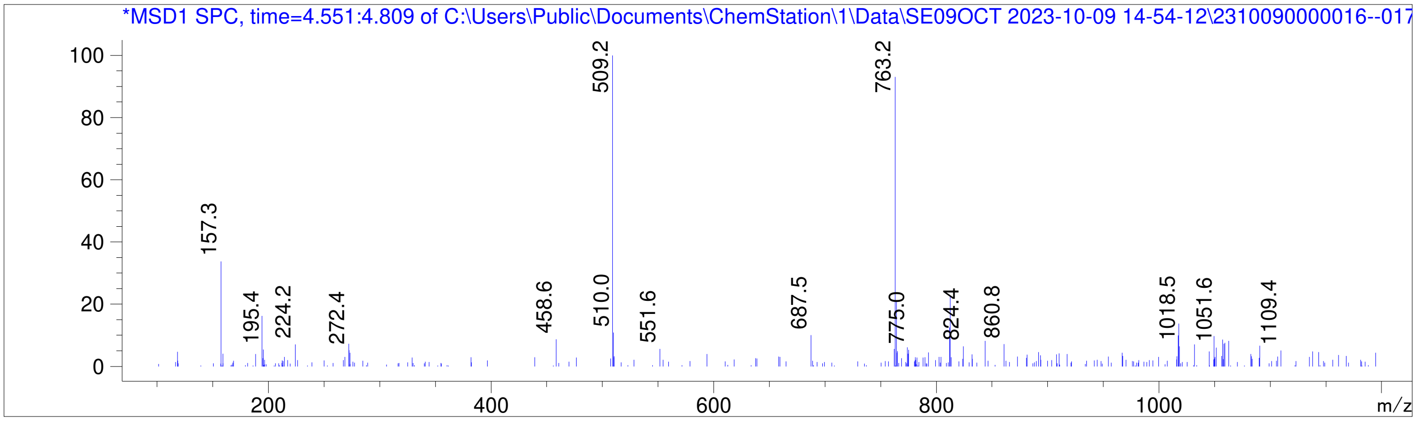
*MSD1 SPC, time=4.551:4.809 of C:\Users\Public\Documents\ChemStation\1\Data\SE09OCT 2023-10-09 14-54-12\2310090000016--017
509.2
763.2
157.3
195.4
224.2
272.4
458.6
510.0
551.6
687.5
775.0
824.4
860.8
1018.5
1051.6
1109.4
m/z
