# Supplementary material for: Astrobiological implications of the stability and reactivity of peptide nucleic acid (PNA) in concentrated sulfuric acid
Source: Sci Adv. 2025 Mar 26;11(13):eadr0006. doi: 10.1126/sciadv.adr0006 (PMC11939054; doi:10.1126/sciadv.adr0006)
Supplement: Supplementary file 2 — Data S1 and S2 [file sciadv.adr0006_data_s1_and_s2.zip › Supplementary Dataset 1-LCMS DATA/LCMS PNA Hexamers A-T/LCMS C6 RT/14d/CPT22010446-21-C2-14d.pdf]

===

Injection Date	:	Wed, 11. Oct. 2023	Seq Line	:	25
			Location	:	23
			Inj. Vol.	:	2 µl

Acq. Method : C:\Users\Public\Documents\ChemStation\1\Data\SE11OCT 2023-10-11 13-11-06\22010446 LCMS-6.M

Analysis Method : C:\Users\Public\Documents\ChemStation\1\Data\SE11OCT 2023-10-11 13-11-06\22010446 LCMS-6.M (Sequence Method)

Waters XBridge Phenyl (4.6 * 150 mm; 3.5 µm); 0.05% TFA (aq) / AcN: 100/0 (0.0 min) --> (6.0 min) --> 70/30 (0.0 min) --> (2.0 min) --> 10/90 (2.0 min); Flow: 1.0 ml/min;
MSD1 = positive; MSD2 = negative

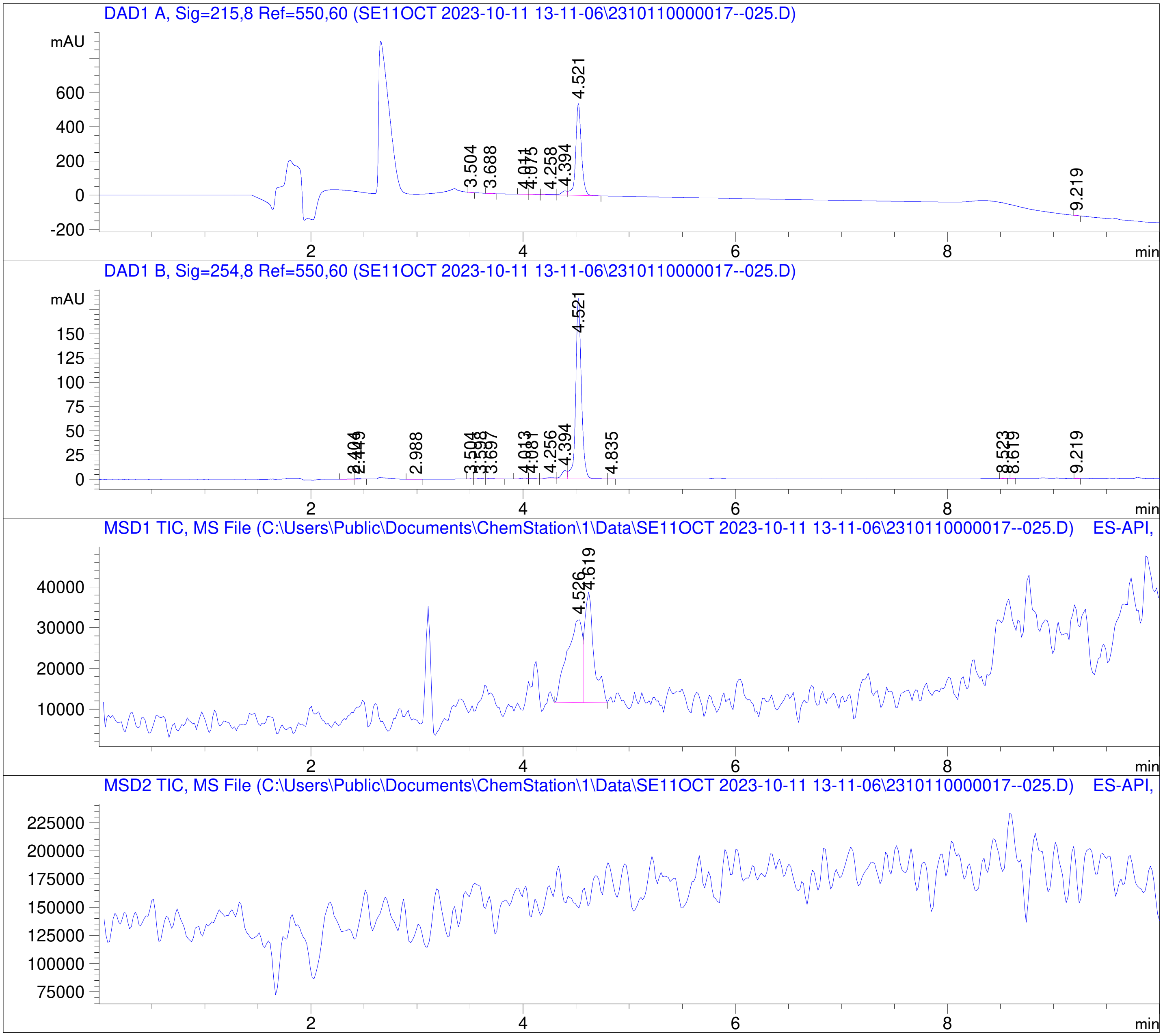

DAD1 A, Sig=215,8 Ref=550,60

Peak #	Ret. Time [min]	Area [mV *s]	Area %
1	3.504	1.719	0.080
2	3.688	6.053	0.282
3	4.011	8.624	0.402
4	4.075	7.352	0.343
5	4.258	23.540	1.097
6	4.394	94.086	4.386
7	4.521	2001.918	93.324
8	9.219	1.844	0.086

DAD1 B, Sig=254,8 Ref=550,60

Peak #	Ret. Time [min]	Area [mV *s]	Area %
1	2.404	0.872	0.116
2	2.449	1.933	0.257
3	2.988	0.465	0.062
4	3.504	0.468	0.062
5	3.598	2.165	0.287
6	3.697	2.798	0.372
7	4.013	3.817	0.507
8	4.081	2.801	0.372
9	4.256	8.528	1.133
10	4.394	33.280	4.420
11	4.521	693.895	92.150
12	4.835	0.338	0.045
13	8.523	0.682	0.091
14	8.619	0.110	0.015
15	9.219	0.857	0.114

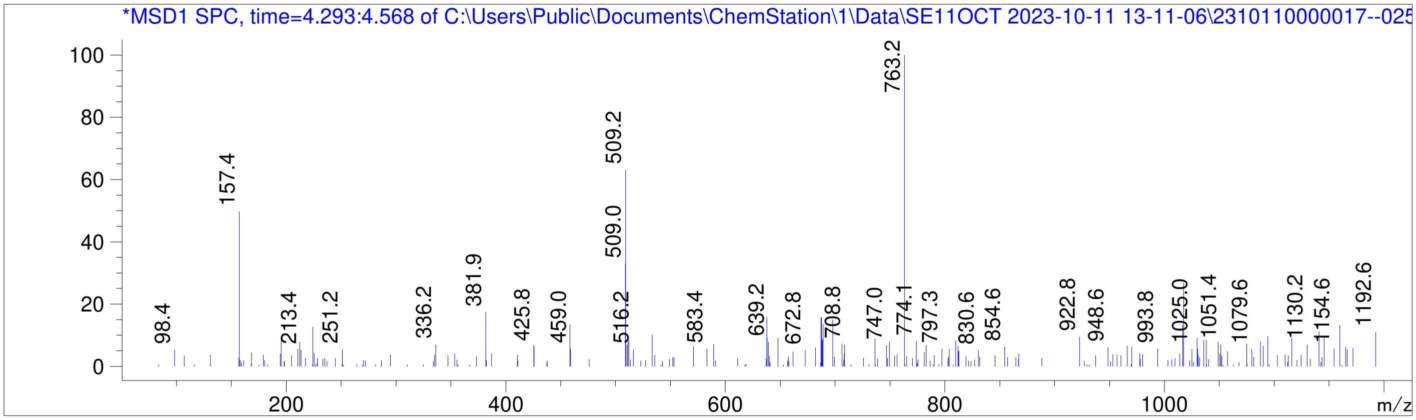
*MSD1 SPC, time=4.293:4.568 of C:\Users\Public\Documents\ChemStation\1\Data\SE11OCT 2023-10-11 13-11-06\2310110000017--025
763.2
509.2
157.4
509.0
381.9
98.4
213.4
251.2
336.2
425.8
459.0
516.2
583.4
639.2
672.8
708.8
747.0
774.1
797.3
830.6
854.6
922.8
948.6
993.8
1025.0
1051.4
1079.6
1130.2
1154.6
1192.6
m/z

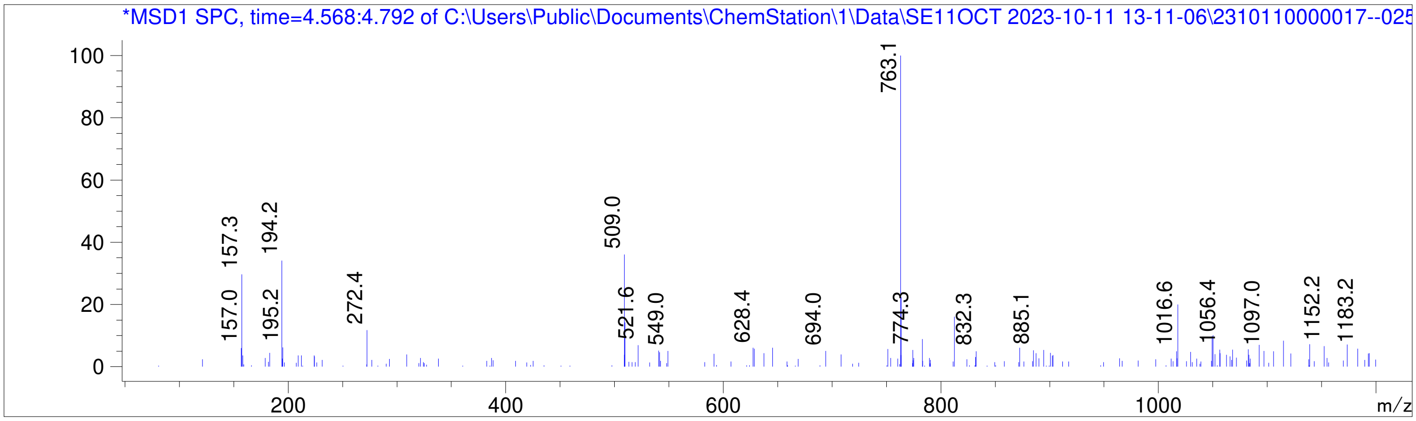
*MSD1 SPC, time=4.568:4.792 of C:\Users\Public\Documents\ChemStation\1\Data\SE11OCT 2023-10-11 13-11-06\2310110000017--025
763.1
509.0
157.3
194.2
157.0
195.2
272.4
521.6
549.0
628.4
694.0
774.3
832.3
885.1
1016.6
1056.4
1097.0
1152.2
1183.2
m/z
