# Supplementary material for: Astrobiological implications of the stability and reactivity of peptide nucleic acid (PNA) in concentrated sulfuric acid
Source: Sci Adv. 2025 Mar 26;11(13):eadr0006. doi: 10.1126/sciadv.adr0006 (PMC11939054; doi:10.1126/sciadv.adr0006)

Data -> C:\Users\Public\Documents\ChemStation\1\Data\SE17OCT 2023-10-17 07-53-29\ ->
Sample-> CPT22010446-21-C3-14d

Injection Date	: Tue, 17. Oct. 2023	Seq Line :	17
		Location :	23
		Inj. Vol. :	2 µl

Acq. Method : C:\Users\Public\Documents\ChemStation\1\Data\SE17OCT 2023-10-17 07-53-29\22010446 LCMS-6.M
Analysis Method : C:\Users\Public\Documents\ChemStation\1\Data\SE17OCT 2023-10-17 07-53-29\22010446 LCMS-6.M (Sequence Method)
Waters XBridge Phenyl (4.6 * 150 mm; 3.5 µm); 0.05% TFA (aq) / AcN: 100/0 (0.0 min) --> (6.0 min) --> 70/30 (0.0 min) --> (2.0 min) --> 10/90 (2.0 min); Flow: 1.0 ml/min;
MSD1 = positive; MSD2 = negative

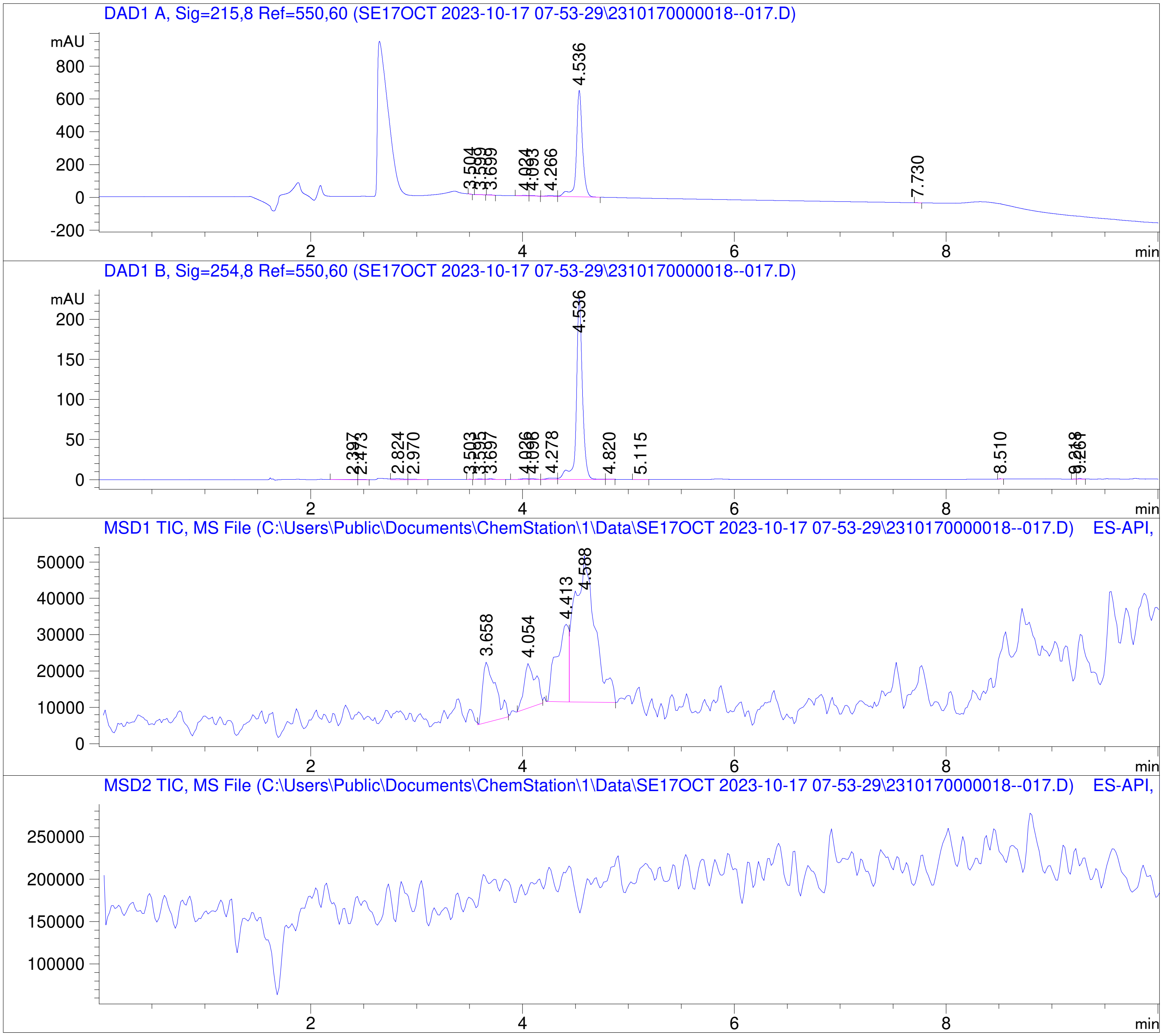

Sample-> CPT22010446-21-C3-14d

===

DAD1 A, Sig=215,8 Ref=550,60

Peak #	Ret. Time [min]	Area [mV *s]	Area %
1	3.504	0.848	0.032
2	3.599	5.135	0.193
3	3.699	3.697	0.139
4	4.024	12.698	0.476
5	4.093	9.667	0.363
6	4.266	25.083	0.941
7	4.536	2608.560	97.837
8	7.730	0.556	0.021

DAD1 B, Sig=254,8 Ref=550,60

Peak #	Ret. Time [min]	Area [mV *s]	Area %
1	2.397	2.441	0.256
2	2.473	1.050	0.110
3	2.824	7.985	0.839
4	2.970	2.674	0.281
5	3.503	0.455	0.048
6	3.595	2.178	0.229
7	3.697	3.247	0.341
8	4.026	4.612	0.484
9	4.096	3.344	0.351
10	4.278	10.589	1.112
11	4.536	909.426	95.529
12	4.820	0.734	0.077
13	5.115	0.329	0.035
14	8.510	0.541	0.057
15	9.218	0.357	0.038
16	9.261	2.028	0.213

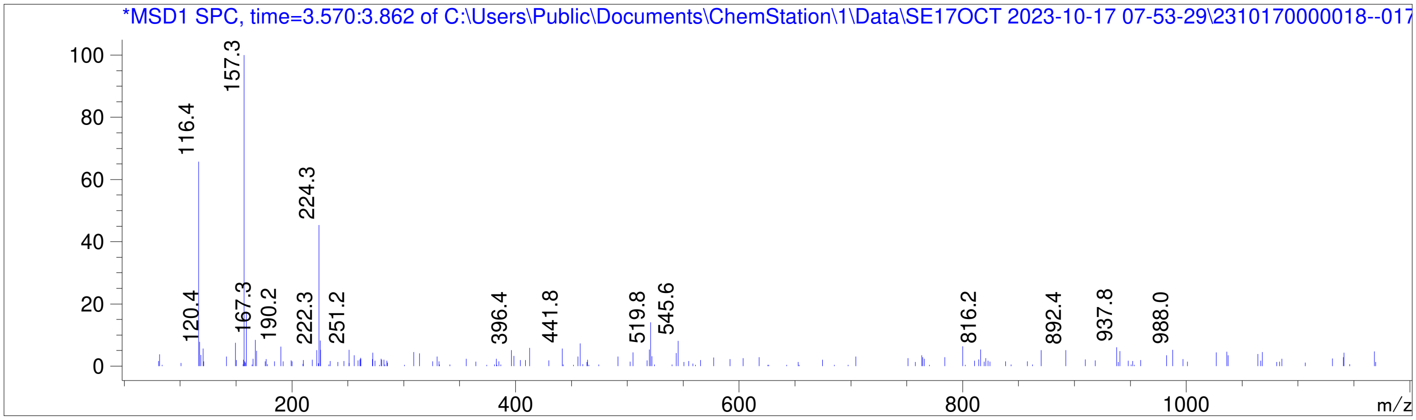
*MSD1 SPC, time=3.570:3.862 of C:\Users\Public\Documents\ChemStation\1\Data\SE17OCT 2023-10-17 07-53-29\2310170000018--017
157.3
116.4
224.3
120.4
167.3
190.2
222.3
251.2
396.4
441.8
519.8
545.6
816.2
892.4
937.8
988.0
m/z

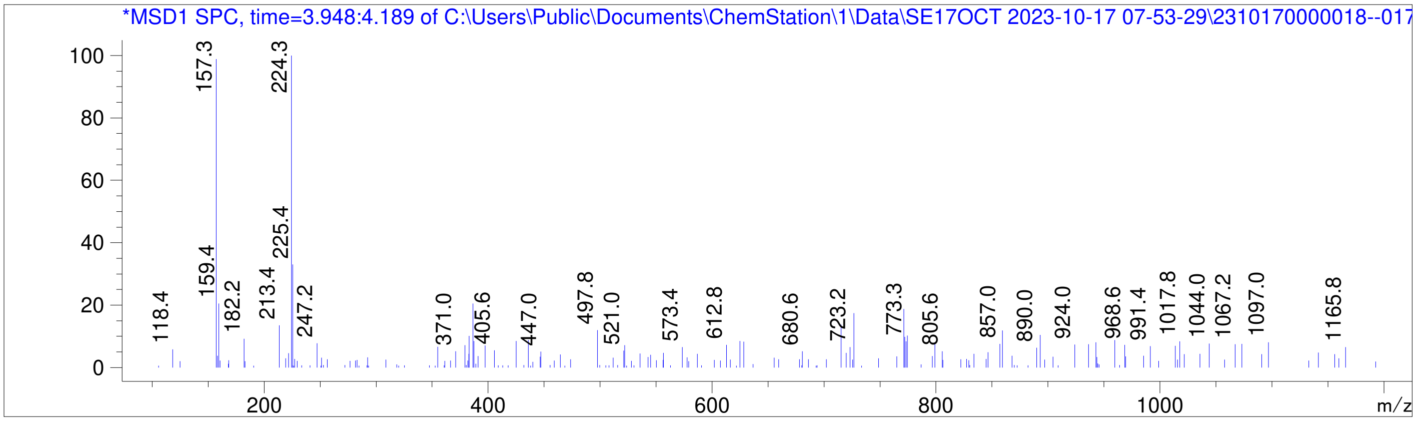
*MSD1 SPC, time=3.948:4.189 of C:\Users\Public\Documents\ChemStation\1\Data\SE17OCT 2023-10-17 07-53-29\2310170000018--017
157.3
224.3
225.4
159.4
213.4
118.4
182.2
247.2
371.0
405.6
447.0
497.8
521.0
573.4
612.8
680.6
723.2
773.3
805.6
857.0
890.0
924.0
968.6
991.4
1017.8
1044.0
1067.2
1097.0
1165.8
m/z

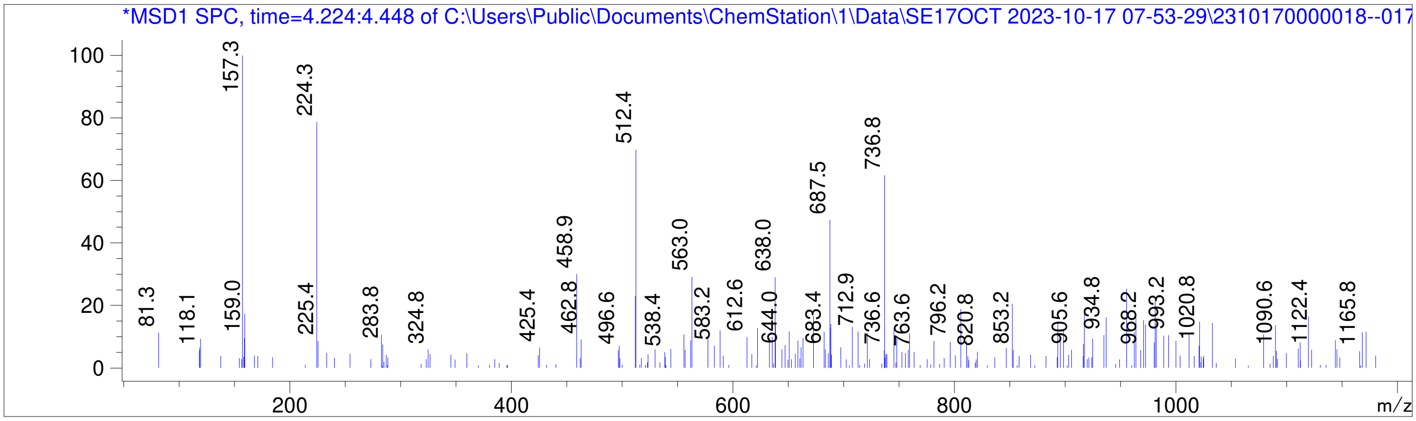
*MSD1 SPC, time=4.224:4.448 of C:\Users\Public\Documents\ChemStation\1\Data\SE17OCT 2023-10-17 07-53-29\2310170000018--017
157.3
224.3
512.4
736.8
687.5
563.0
638.0
458.9
81.3
118.1
159.0
225.4
283.8
324.8
425.4
462.8
496.6
538.4
583.2
612.6
644.0
683.4
712.9
736.6
763.6
796.2
820.8
853.2
905.6
934.8
968.2
993.2
1020.8
1090.6
1122.4
1165.8
m/z

Data -> C:\Users\Public\Documents\ChemStation\1\Data\SE17OCT 2023-10-17 07-53-29\ ->
Sample-> CPT22010446-21-C3-14d

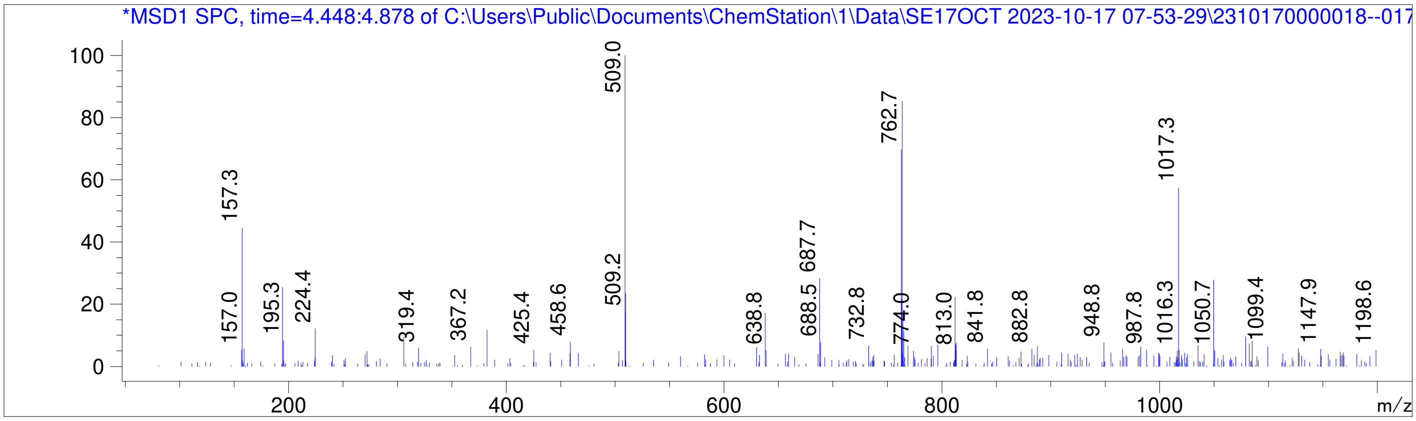

Supplement: Supplementary file 2 — Data S1 and S2 [file sciadv.adr0006_data_s1_and_s2.zip › Supplementary Dataset 1-LCMS DATA/LCMS PNA Hexamers A-T/LCMS C6 RT/14d/CPT22010446-21-C3-14d.pdf]
