# Supplementary material for: Astrobiological implications of the stability and reactivity of peptide nucleic acid (PNA) in concentrated sulfuric acid
Source: Sci Adv. 2025 Mar 26;11(13):eadr0006. doi: 10.1126/sciadv.adr0006 (PMC11939054; doi:10.1126/sciadv.adr0006)
Supplement: Supplementary file 2 — Data S1 and S2 [file sciadv.adr0006_data_s1_and_s2.zip › Supplementary Dataset 1-LCMS DATA/LCMS PNA Hexamers A-T/LCMS C6 RT/1h/CPT22010446-13-C.pdf]

===

Injection Date : Tue, 26. Sep. 2023

Seq Line : 8
Location : 60
Inj. Vol. : 2 µl

Acq. Method : C:\Users\Public\Documents\ChemStation\1\Data\SE26SEP 2023-09-26 10-10-32\22010446 LCMS-6.M
Analysis Method : C:\Users\Public\Documents\ChemStation\1\Data\SE26SEP 2023-09-26 10-10-32\22010446 LCMS-6.M (Sequence Method)
Waters XBridge Phenyl (4.6 * 150 mm; 3.5 µm); 0.05% TFA (aq) / AcN: 100/0 (0.0 min) --> (6.0 min) --> 70/30 (0.0 min) --> (2.0 min) --> 10/90 (2.0 min); Flow: 1.0 ml/min;
MSD1 = positive; MSD2 = negative

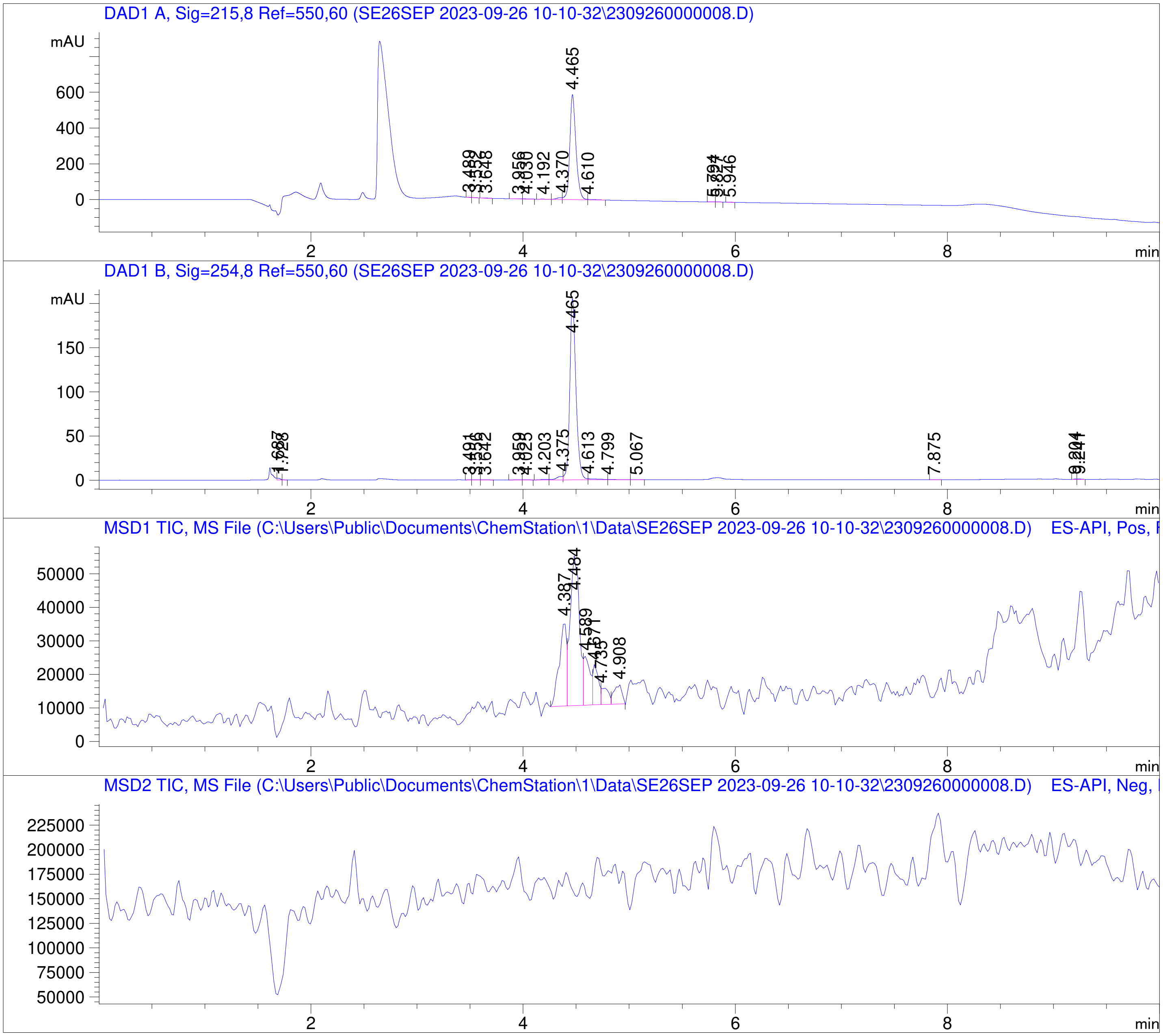

DAD1 A, Sig=215,8 Ref=550,60

Peak #	Ret. Time [min]	Area [mV *s]	Area %
1	3.489	1.760	0.072
2	3.552	1.655	0.068
3	3.648	4.304	0.176
4	3.956	4.276	0.175
5	4.030	4.175	0.171
6	4.192	5.894	0.241
7	4.370	38.225	1.565
8	4.465	2363.736	96.756
9	4.610	14.609	0.598
10	5.794	1.998	0.082
11	5.827	1.495	0.061
12	5.946	0.868	0.036

DAD1 B, Sig=254,8 Ref=550,60

Peak #	Ret. Time [min]	Area [mV *s]	Area %
1	1.687	4.002	0.462
2	1.728	0.294	0.034
3	3.491	0.404	0.047
4	3.556	0.699	0.081
5	3.642	0.920	0.106
6	3.959	1.092	0.126
7	4.025	0.880	0.102
8	4.203	2.425	0.280
9	4.375	15.999	1.848
10	4.465	824.399	95.214
11	4.613	8.990	1.038
12	4.799	2.724	0.315
13	5.067	0.444	0.051
14	7.875	0.192	0.022
15	9.204	0.720	0.083
16	9.241	1.654	0.191

==

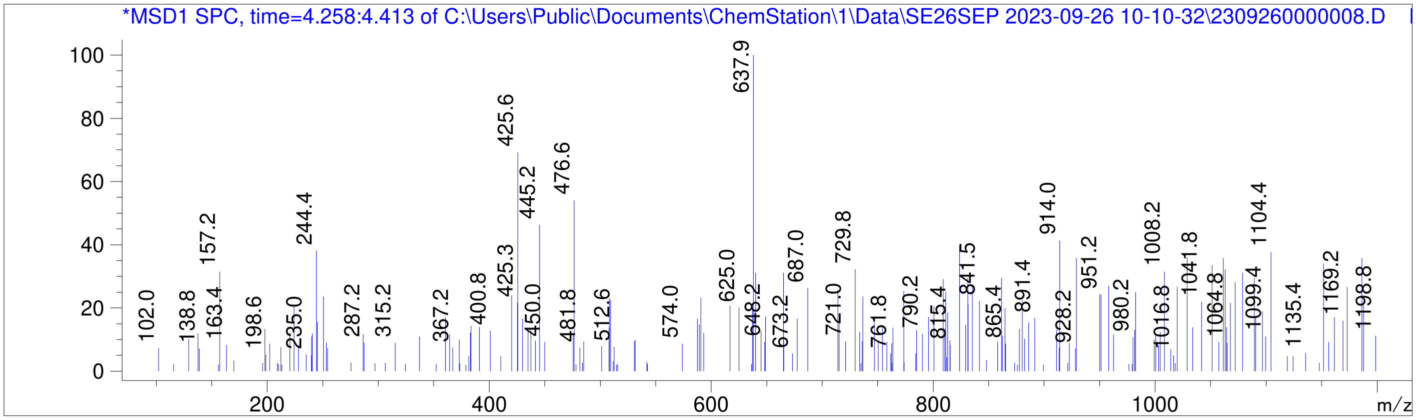
*MSD1 SPC, time=4.258:4.413 of C:\Users\Public\Documents\ChemStation\1\Data\SE26SEP 2023-09-26 10-10-32\2309260000008.D
637.9
425.6
476.6
445.2
914.0
1104.4
157.2
244.4
729.8
687.0
1008.2
951.2
1041.8
841.5
625.0
425.3
400.8
1169.2
891.4
1198.8
1064.8
1099.4
m/z

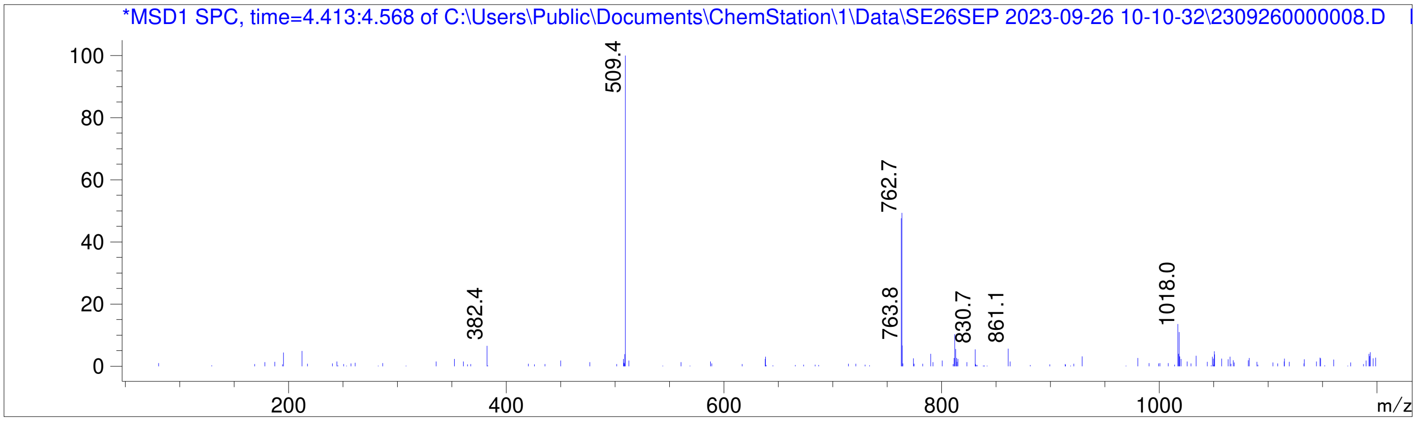
*MSD1 SPC, time=4.413:4.568 of C:\Users\Public\Documents\ChemStation\1\Data\SE26SEP 2023-09-26 10-10-32\2309260000008.D
509.4
762.7
382.4
763.8
830.7
861.1
1018.0
m/z

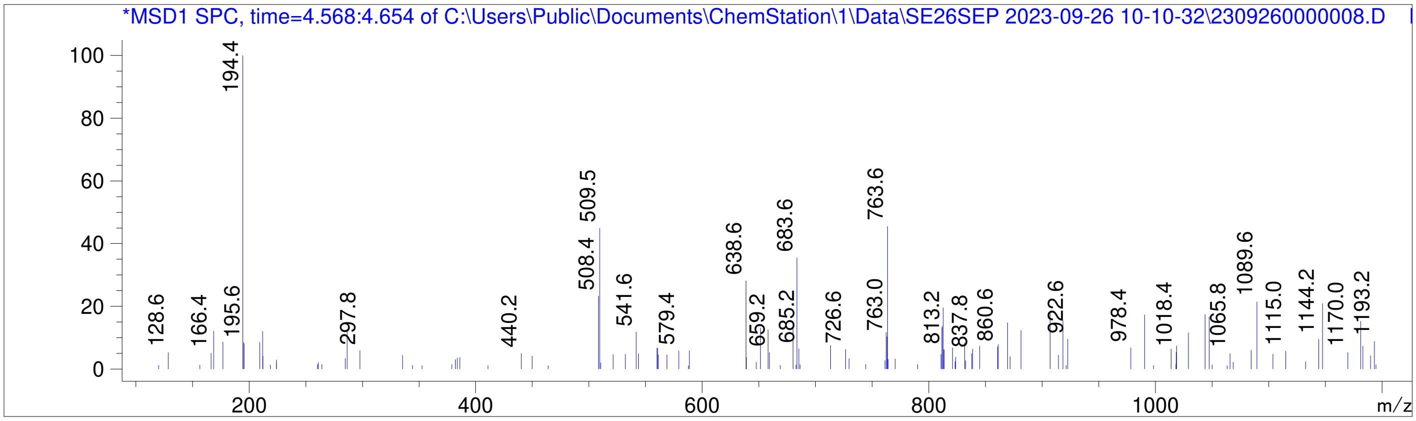
*MSD1 SPC, time=4.568:4.654 of C:\Users\Public\Documents\ChemStation\1\Data\SE26SEP 2023-09-26 10-10-32\2309260000008.D
194.4
509.5
763.6
683.6
638.6
508.4
1089.6
m/z

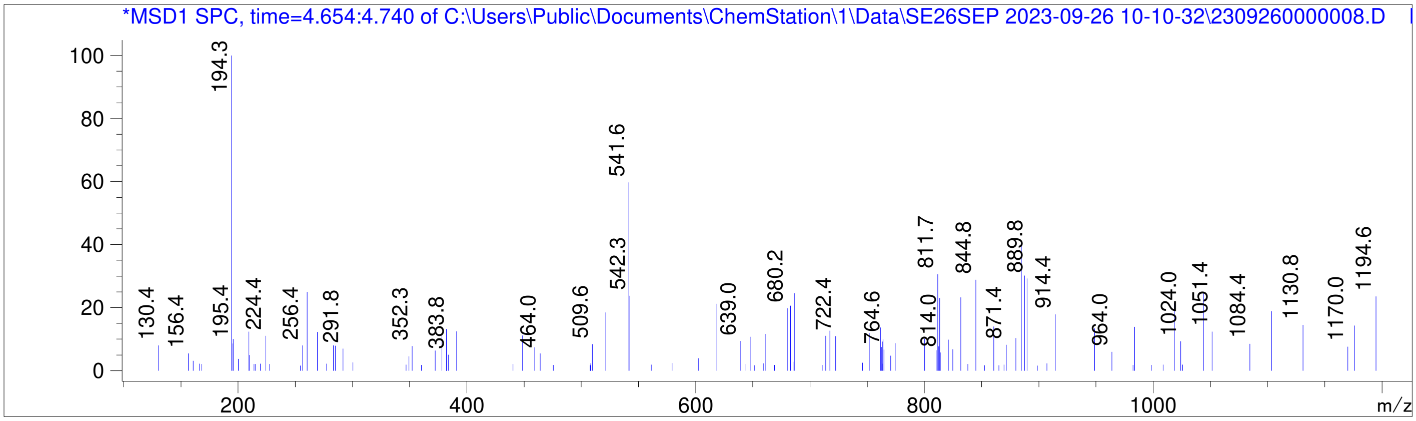
*MSD1 SPC, time=4.654:4.740 of C:\Users\Public\Documents\ChemStation\1\Data\SE26SEP 2023-09-26 10-10-32\2309260000008.D
194.3
541.6
542.3
811.7
844.8
889.8
914.4
680.2
1194.6
130.4
156.4
195.4
224.4
256.4
291.8
352.3
383.8
464.0
509.6
639.0
722.4
764.6
814.0
871.4
964.0
1024.0
1051.4
1084.4
1130.8
1170.0
m/z

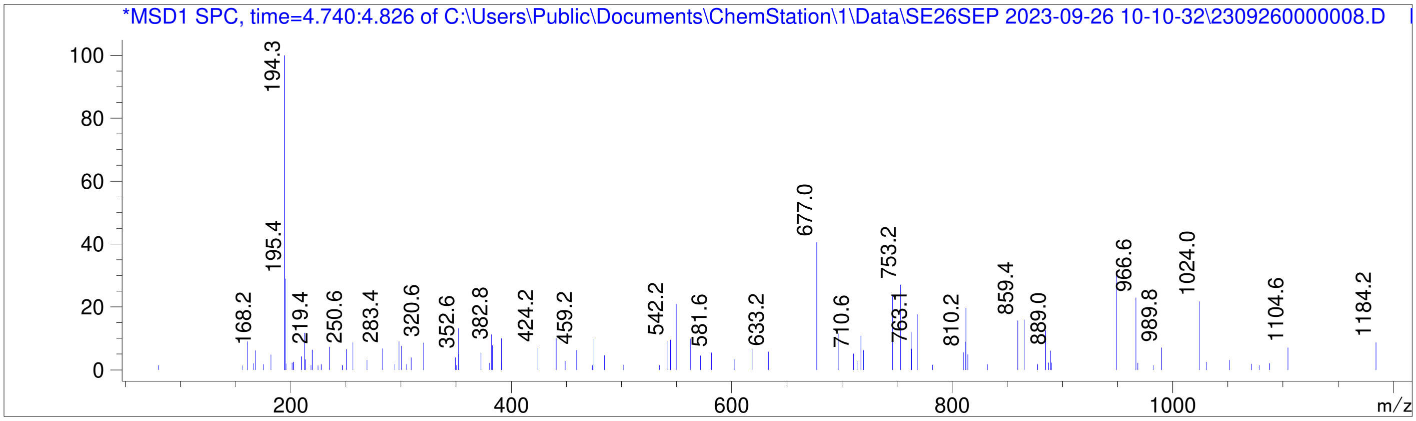
*MSD1 SPC, time=4.740:4.826 of C:\Users\Public\Documents\ChemStation\1\Data\SE26SEP 2023-09-26 10-10-32\2309260000008.D
194.3
195.4
677.0
753.2
966.6
1024.0
859.4
1184.2
1104.6
168.2
219.4
250.6
283.4
320.6
352.6
382.8
424.2
459.2
542.2
581.6
633.2
710.6
763.1
810.2
889.0
989.8
m/z

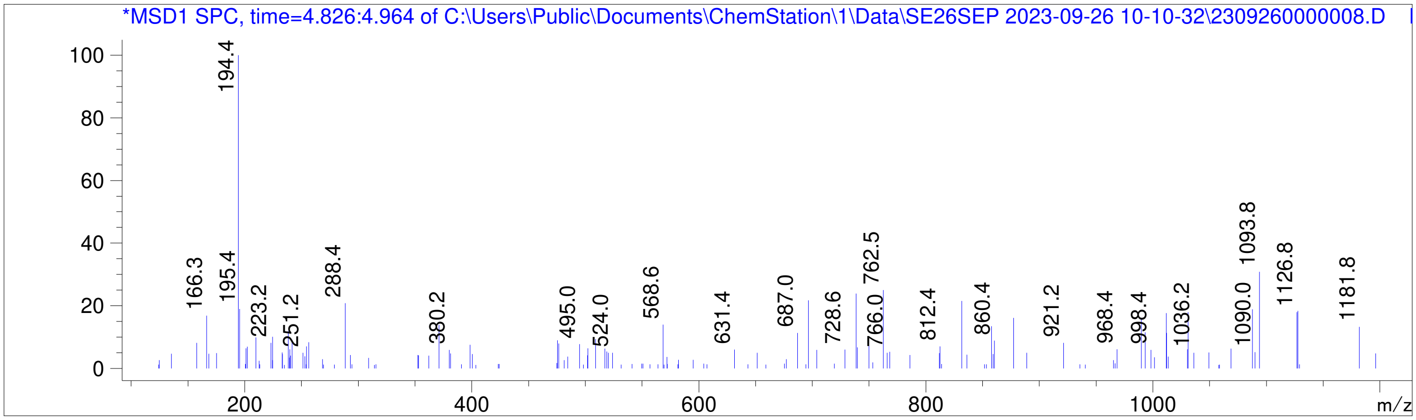
*MSD1 SPC, time=4.826:4.964 of C:\Users\Public\Documents\ChemStation\1\Data\SE26SEP 2023-09-26 10-10-32\2309260000008.D
194.4
1093.8
762.5
1126.8
1181.8
166.3
195.4
223.2
251.2
288.4
380.2
495.0
524.0
568.6
631.4
687.0
728.6
766.0
812.4
860.4
921.2
968.4
998.4
1036.2
1090.0
m/z
