# Supplementary material for: Astrobiological implications of the stability and reactivity of peptide nucleic acid (PNA) in concentrated sulfuric acid
Source: Sci Adv. 2025 Mar 26;11(13):eadr0006. doi: 10.1126/sciadv.adr0006 (PMC11939054; doi:10.1126/sciadv.adr0006)
Supplement: Supplementary file 2 — Data S1 and S2 [file sciadv.adr0006_data_s1_and_s2.zip › Supplementary Dataset 1-LCMS DATA/LCMS PNA Hexamers A-T/LCMS C6 RT/1h/CPT22010446-13-C3.pdf]

Data -> C:\Users\Public\Documents\ChemStation\1\Data\SE02OCT 2023-10-02 11-39-16\ ->
Sample-> CPT22010446-13-C3

==

Injection Date : Mon, 2. Oct. 2023 — Seq Line : 11; Location : 41; Inj. Vol. : 2 µl

Acq. Method : C:\Users\Public\Documents\ChemStation\1\Data\SE02OCT 2023-10-02 11-39-16\22010446 LCMS-6.M
Analysis Method : C:\Users\Public\Documents\ChemStation\1\Data\SE02OCT 2023-10-02 11-39-16\22010446 LCMS-6.M (Sequence Method)
Waters XBridge Phenyl (4.6 * 150 mm; 3.5 µm); 0.05% TFA (aq) / AcN: 100/0 (0.0 min) --> (6.0 min) --> 70/30 (0.0 min) --> (2.0 min) --> 10/90 (2.0 min); Flow: 1.0 ml/min;
MSD1 = positive; MSD2 = negative

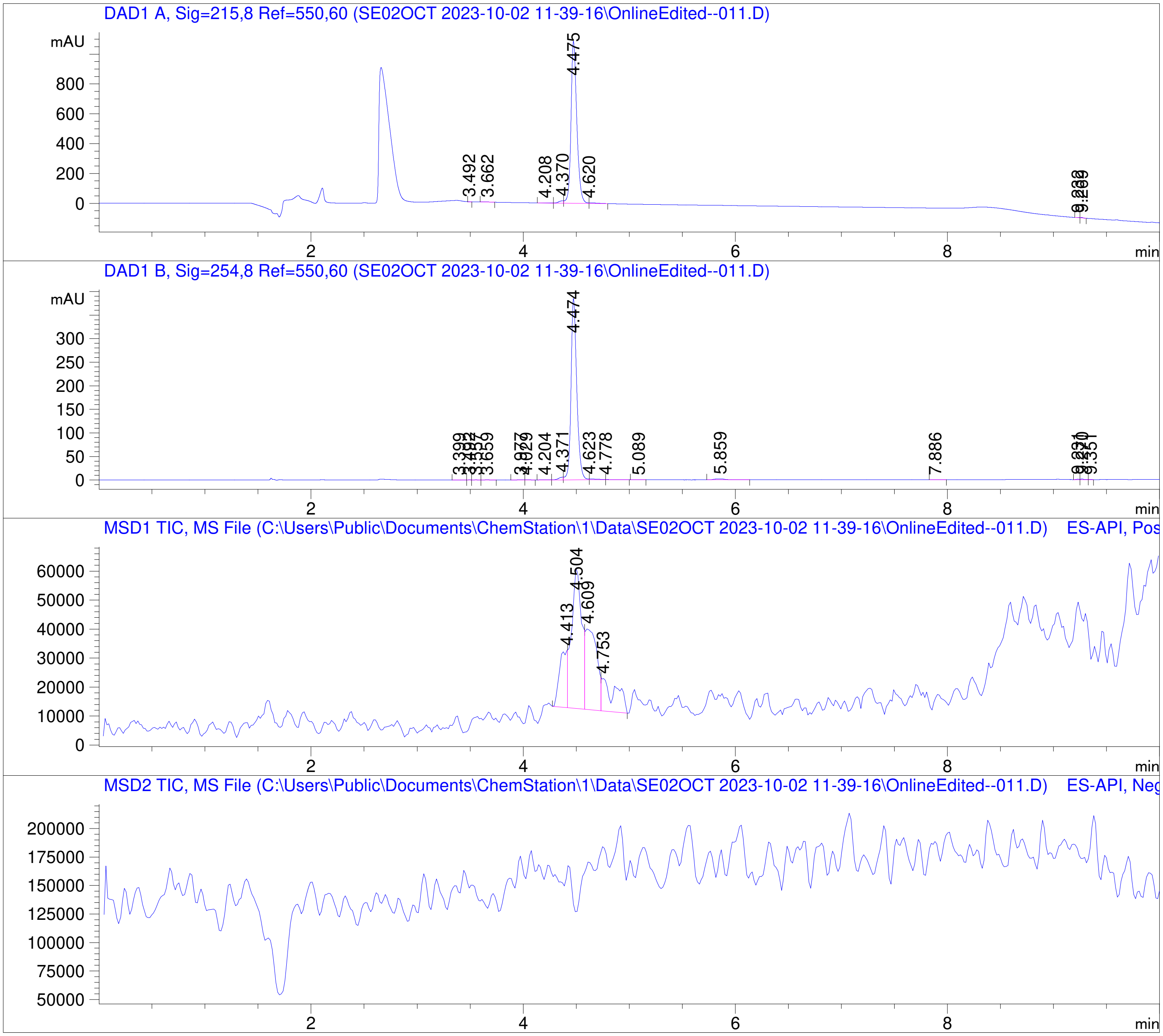

Sample-> CPT22010446-13-C3

DAD1 A, Sig=215,8 Ref=550,60

Peak #	Ret. Time [min]	Area [mV *s]	Area %
1	3.492	0.386	0.009
2	3.662	4.131	0.098
3	4.208	5.791	0.137
4	4.370	49.359	1.167
5	4.475	4137.754	97.841
6	4.620	22.991	0.544
7	9.232	3.837	0.091
8	9.269	4.805	0.114

DAD1 B, Sig=254,8 Ref=550,60

Peak #	Ret. Time [min]	Area [mV *s]	Area %
1	3.399	0.877	0.058
2	3.492	0.521	0.034
3	3.557	0.803	0.053
4	3.659	0.903	0.060
5	3.977	0.975	0.064
6	4.029	0.639	0.042
7	4.204	2.399	0.158
8	4.371	17.333	1.144
9	4.474	1455.874	96.050
10	4.623	11.894	0.785
11	4.778	3.382	0.223
12	5.089	0.462	0.031
13	5.859	14.859	0.980
14	7.886	0.301	0.020
15	9.231	1.642	0.108
16	9.270	2.603	0.172
17	9.351	0.280	0.018

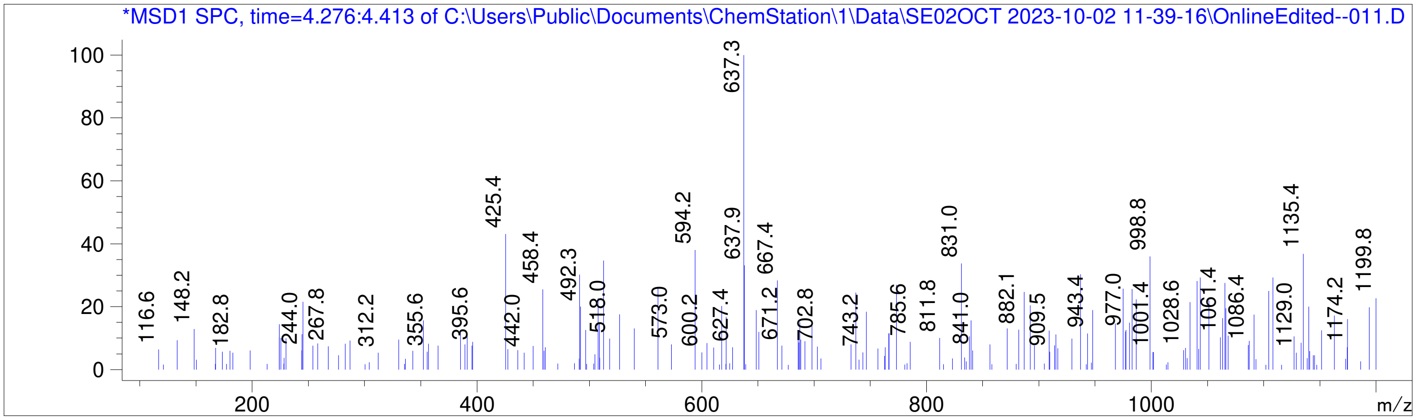
*MSD1 SPC, time=4.276:4.413 of C:\Users\Public\Documents\ChemStation\1\Data\SE02OCT 2023-10-02 11-39-16\OnlineEdited--011.D
116.6
148.2
182.8
244.0
267.8
312.2
355.6
395.6
425.4
442.0
458.4
492.3
518.0
573.0
594.2
600.2
627.4
637.3
637.9
667.4
671.2
702.8
743.2
785.6
811.8
831.0
841.0
882.1
909.5
943.4
977.0
998.8
1001.4
1028.6
1061.4
1086.4
1129.0
1135.4
1174.2
1199.8
m/z

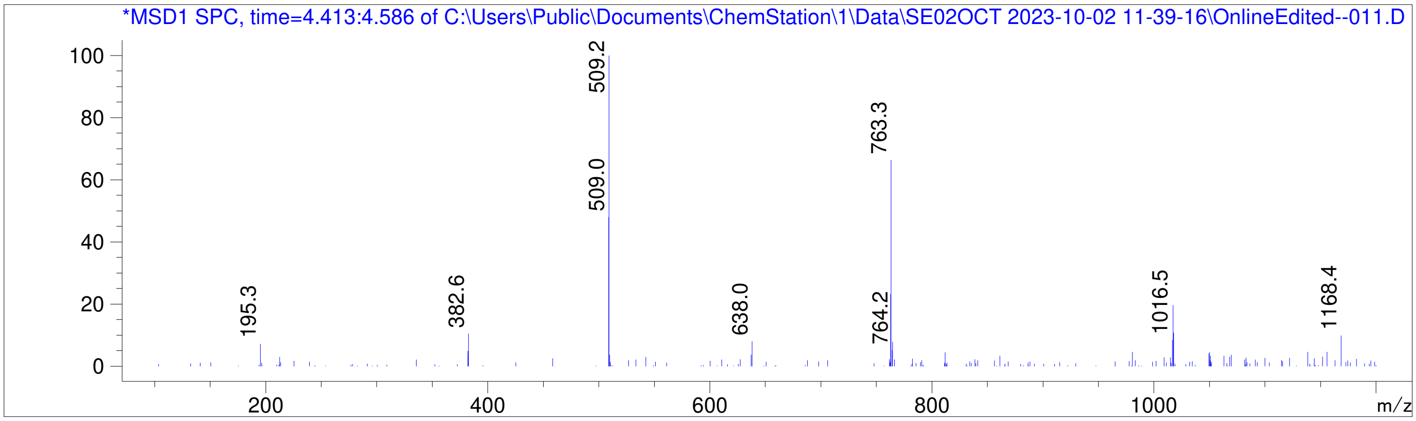
*MSD1 SPC, time=4.413:4.586 of C:\Users\Public\Documents\ChemStation\1\Data\SE02OCT 2023-10-02 11-39-16\OnlineEdited--011.D
195.3
382.6
509.2
509.0
638.0
763.3
764.2
1016.5
1168.4
m/z

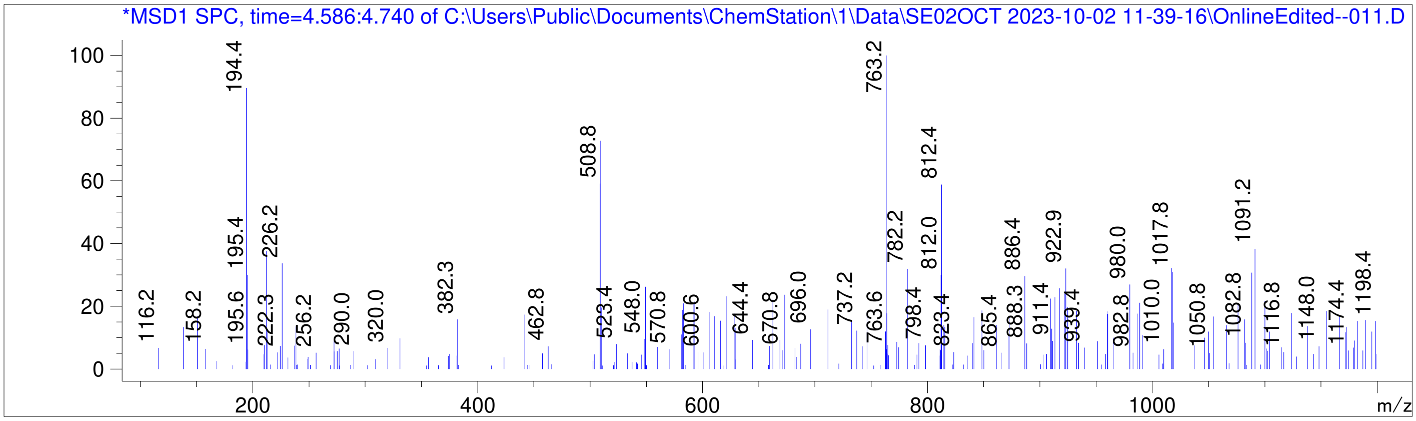
*MSD1 SPC, time=4.586:4.740 of C:\Users\Public\Documents\ChemStation\1\Data\SE02OCT 2023-10-02 11-39-16\OnlineEdited--011.D
116.2
158.2
194.4
195.4
195.6
222.3
226.2
256.2
290.0
320.0
382.3
462.8
508.8
523.4
548.0
570.8
600.6
644.4
670.8
696.0
737.2
763.2
763.6
782.2
798.4
812.4
812.0
823.4
865.4
886.4
888.3
911.4
922.9
939.4
980.0
982.8
1010.0
1017.8
1050.8
1082.8
1091.2
1116.8
1148.0
1174.4
1198.4
m/z

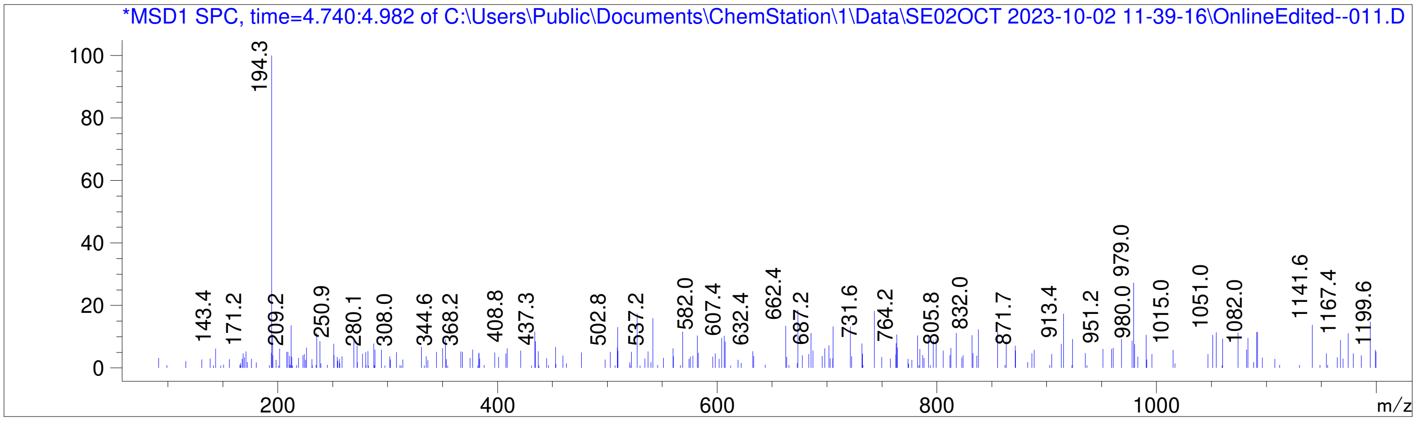
*MSD1 SPC, time=4.740:4.982 of C:\Users\Public\Documents\ChemStation\1\Data\SE02OCT 2023-10-02 11-39-16\OnlineEdited--011.D
194.3
979.0
143.4
171.2
209.2
250.9
280.1
308.0
344.6
368.2
408.8
437.3
502.8
537.2
582.0
607.4
632.4
662.4
687.2
731.6
764.2
805.8
832.0
871.7
913.4
951.2
980.0
1015.0
1051.0
1082.0
1141.6
1167.4
1199.6
m/z
