# Supplementary material for: Astrobiological implications of the stability and reactivity of peptide nucleic acid (PNA) in concentrated sulfuric acid
Source: Sci Adv. 2025 Mar 26;11(13):eadr0006. doi: 10.1126/sciadv.adr0006 (PMC11939054; doi:10.1126/sciadv.adr0006)
Supplement: Supplementary file 2 — Data S1 and S2 [file sciadv.adr0006_data_s1_and_s2.zip › Supplementary Dataset 1-LCMS DATA/LCMS PNA Hexamers A-T/LCMS C6 RT/1h/CPT22010446-19-A-2.pdf]

Data -> C:\Users\Public\Documents\ChemStation\1\Data\SE26SEP 2023-09-26 10-10-32\ ->
Sample-> CPT22010446-19-A-2

===

Injection Date : Tue, 26. Sep. 2023

Seq Line : 22
Location : 72
Inj. Vol. : 2 µl

Acq. Method : C:\Users\Public\Documents\ChemStation\1\Data\SE26SEP 2023-09-26 10-10-32\22010446 LCMS-6.M
Analysis Method : C:\Users\Public\Documents\ChemStation\1\Data\SE26SEP 2023-09-26 10-10-32\22010446 LCMS-6.M (Sequence Method)
Waters XBridge Phenyl (4.6 * 150 mm; 3.5 µm); 0.05% TFA (aq) / AcN: 100/0 (0.0 min) --> (6.0 min) --> 70/30 (0.0 min) --> (2.0 min) --> 10/90 (2.0 min); Flow: 1.0 ml/min;
MSD1 = positive; MSD2 = negative

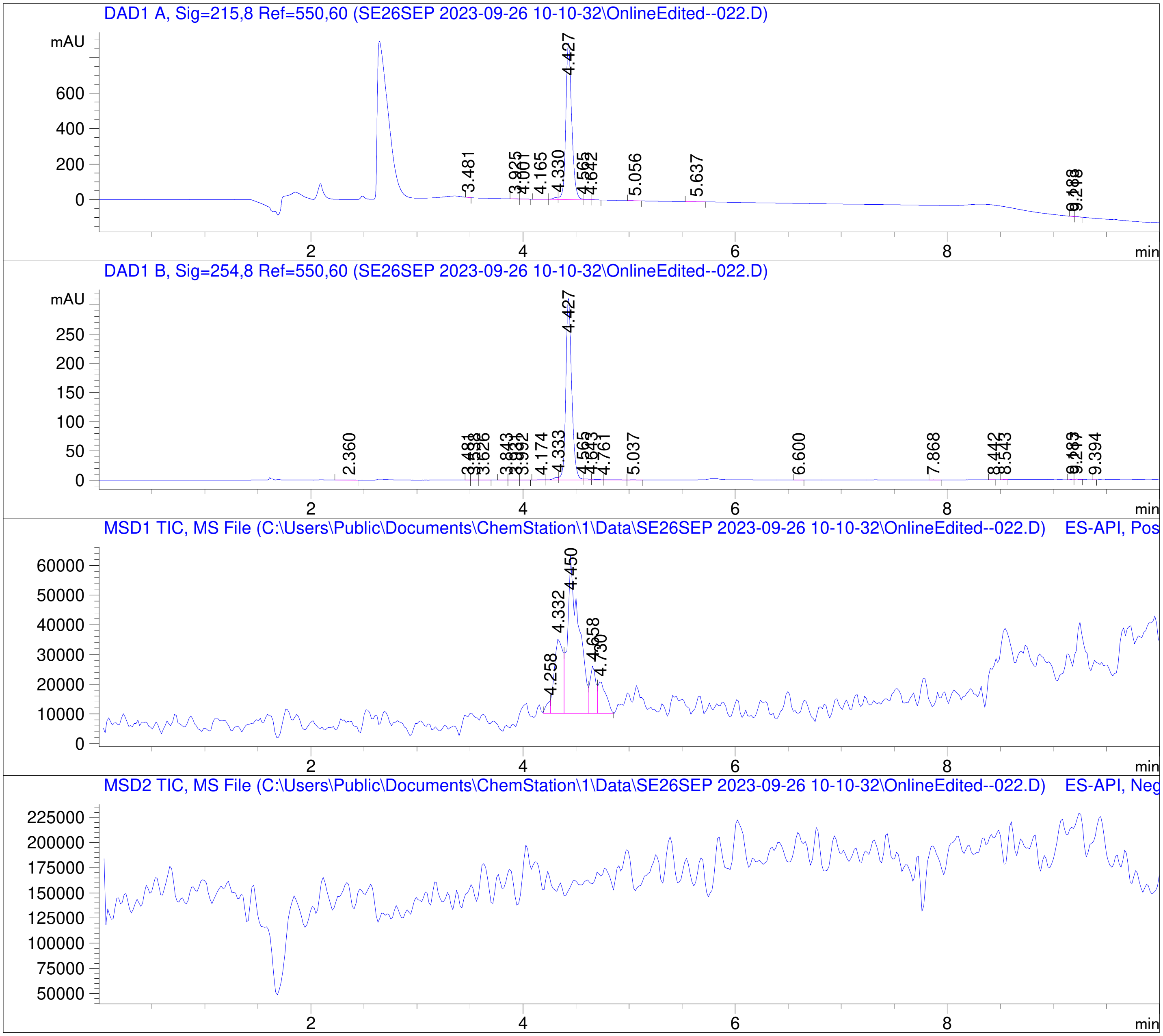

DAD1 A, Sig=215,8 Ref=550,60

Peak #	Ret. Time [min]	Area [mV *s]	Area %
1	3.481	0.940	0.027
2	3.925	1.608	0.046
3	4.001	1.646	0.047
4	4.165	5.011	0.144
5	4.330	36.147	1.037
6	4.427	3407.409	97.801
7	4.565	14.228	0.408
8	4.642	6.204	0.178
9	5.056	1.386	0.040
10	5.637	2.503	0.072
11	9.183	2.096	0.060
12	9.218	4.837	0.139

DAD1 B, Sig=254,8 Ref=550,60

Peak #	Ret. Time [min]	Area [mV *s]	Area %
1	2.360	0.270	0.022
2	3.481	0.269	0.022
3	3.538	0.476	0.039
4	3.626	0.595	0.048
5	3.843	0.167	0.013
6	3.931	0.821	0.066
7	3.992	0.556	0.045
8	4.174	1.792	0.145
9	4.333	14.922	1.208
10	4.427	1194.533	96.670
11	4.565	7.027	0.569
12	4.643	5.700	0.461
13	4.761	3.506	0.284
14	5.037	0.827	0.067
15	6.600	0.104	0.008
16	7.868	0.184	0.015
17	8.442	0.111	0.009
18	8.543	0.158	0.013
19	9.183	1.031	0.083
20	9.217	2.562	0.207
21	9.394	0.073	0.006

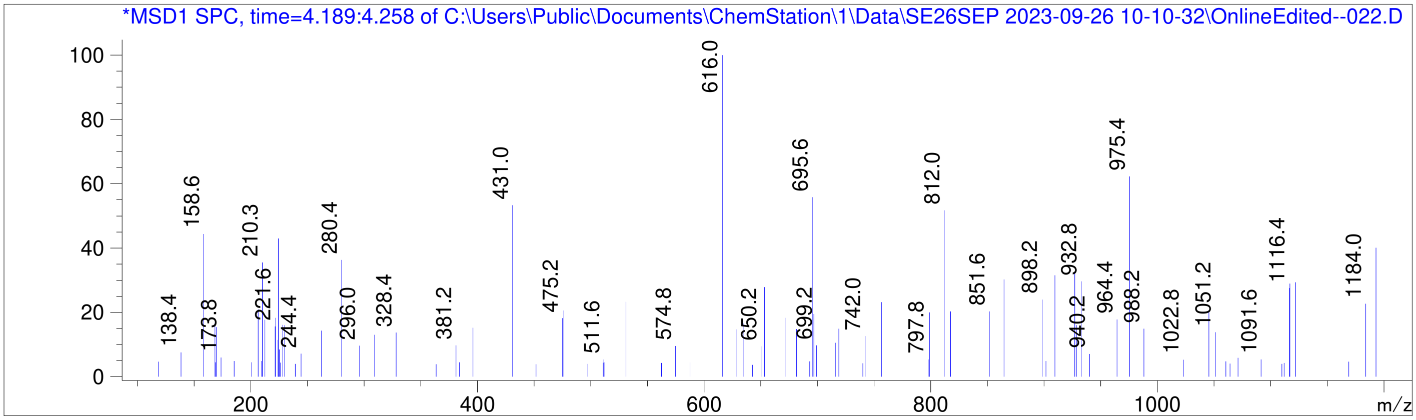
*MSD1 SPC, time=4.189:4.258 of C:\Users\Public\Documents\ChemStation\1\Data\SE26SEP 2023-09-26 10-10-32\OnlineEdited--022.D
138.4
158.6
173.8
210.3
221.6
244.4
280.4
296.0
328.4
381.2
431.0
475.2
511.6
574.8
616.0
650.2
695.6
699.2
742.0
797.8
812.0
851.6
898.2
932.8
940.2
964.4
975.4
988.2
1022.8
1051.2
1091.6
1116.4
1184.0
m/z

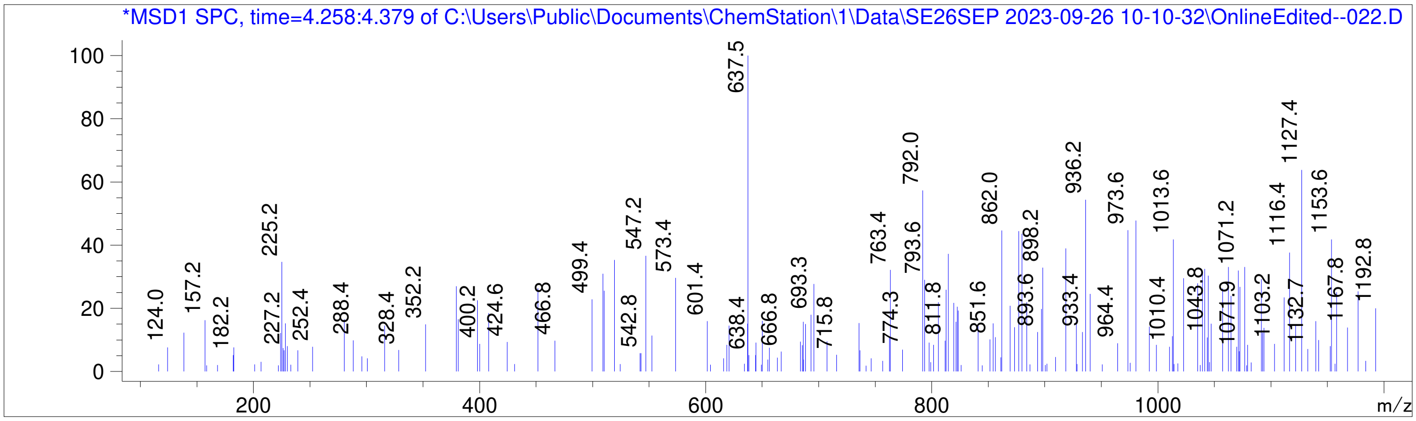
*MSD1 SPC, time=4.258:4.379 of C:\Users\Public\Documents\ChemStation\1\Data\SE26SEP 2023-09-26 10-10-32\OnlineEdited--022.D
124.0
157.2
182.2
225.2
227.2
252.4
288.4
328.4
352.2
400.2
424.6
466.8
499.4
542.8
547.2
573.4
601.4
637.5
638.4
666.8
693.3
715.8
763.4
774.3
792.0
793.6
811.8
851.6
862.0
893.6
898.2
933.4
936.2
964.4
973.6
1010.4
1013.6
1043.8
1071.2
1071.9
1103.2
1116.4
1127.4
1132.7
1153.6
1167.8
1192.8
m/z

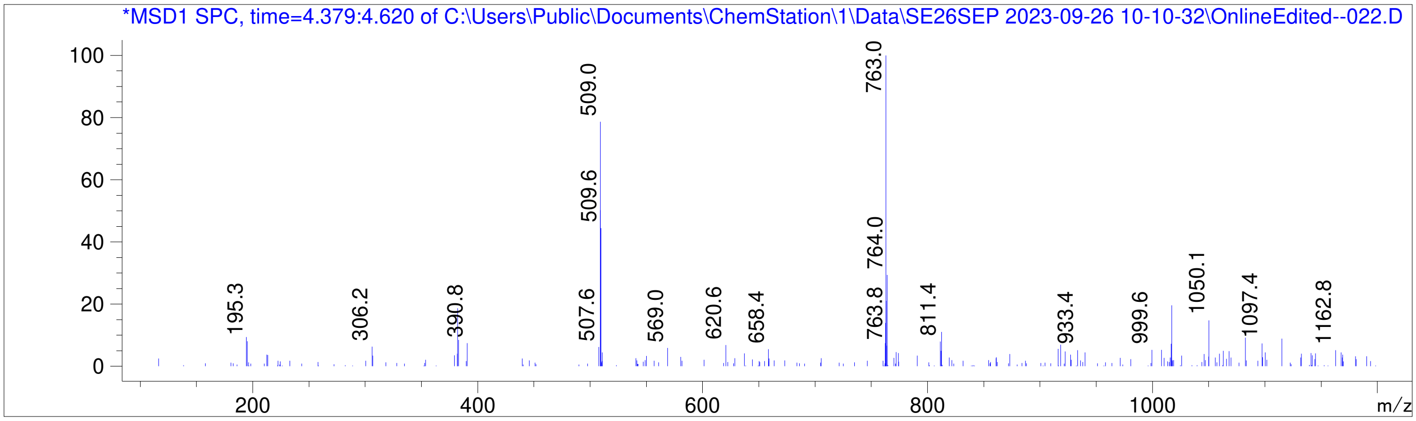
*MSD1 SPC, time=4.379:4.620 of C:\Users\Public\Documents\ChemStation\1\Data\SE26SEP 2023-09-26 10-10-32\OnlineEdited--022.D
195.3
306.2
390.8
507.6
509.0
509.6
569.0
620.6
658.4
763.0
763.8
764.0
811.4
933.4
999.6
1050.1
1097.4
1162.8
m/z

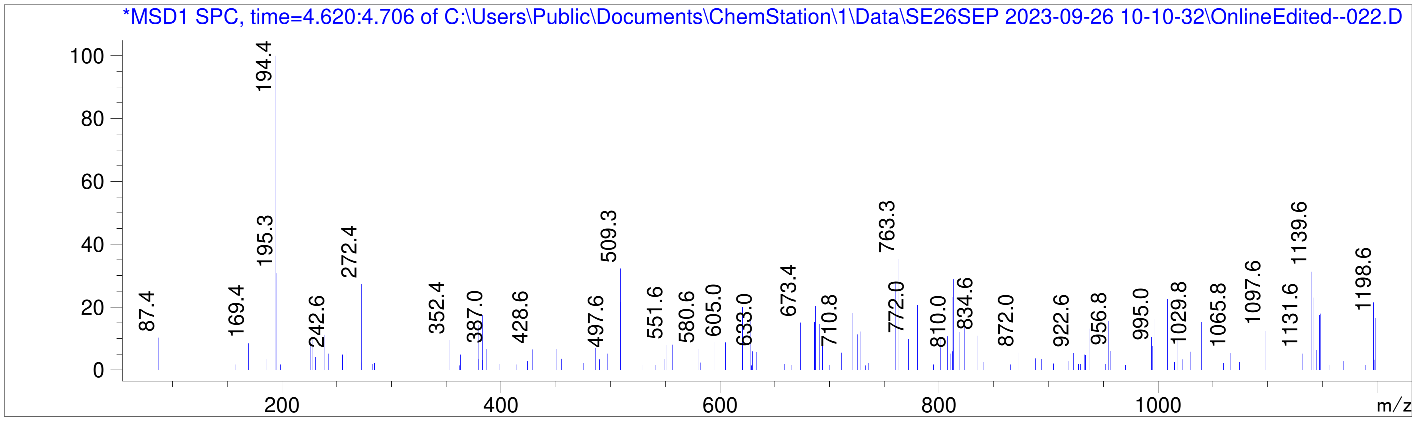
*MSD1 SPC, time=4.620:4.706 of C:\Users\Public\Documents\ChemStation\1\Data\SE26SEP 2023-09-26 10-10-32\OnlineEdited--022.D
194.4
195.3
272.4
509.3
763.3
1139.6
1198.6
87.4
169.4
242.6
352.4
387.0
428.6
497.6
551.6
580.6
605.0
633.0
673.4
710.8
772.0
810.0
834.6
872.0
922.6
956.8
995.0
1029.8
1065.8
1097.6
1131.6
m/z

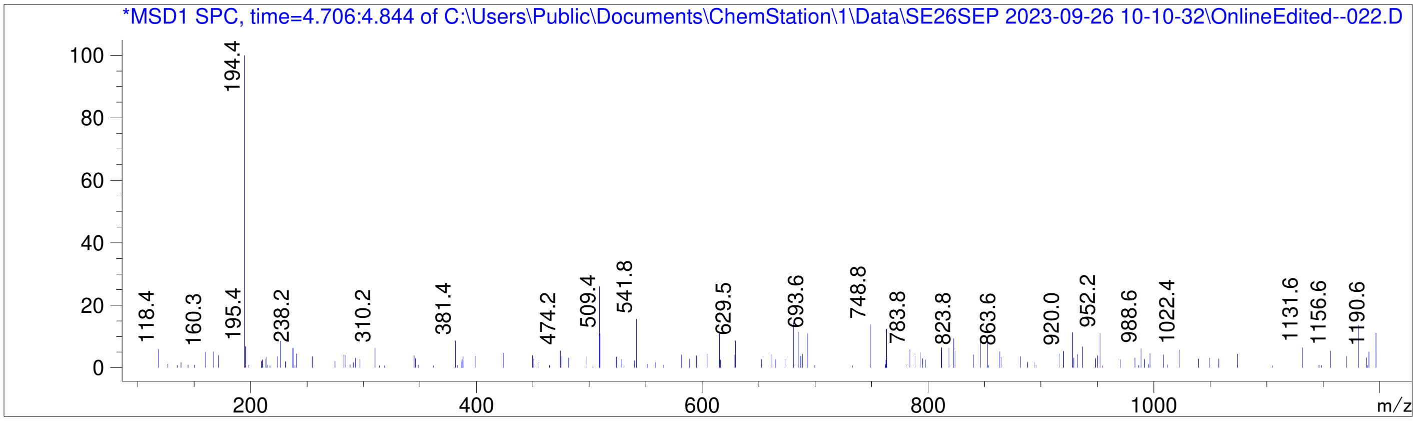
*MSD1 SPC, time=4.706:4.844 of C:\Users\Public\Documents\ChemStation\1\Data\SE26SEP 2023-09-26 10-10-32\OnlineEdited--022.D
194.4
118.4
160.3
195.4
238.2
310.2
381.4
474.2
509.4
541.8
629.5
693.6
748.8
783.8
823.8
863.6
920.0
952.2
988.6
1022.4
1131.6
1156.6
1190.6
m/z
