# Supplementary material for: Astrobiological implications of the stability and reactivity of peptide nucleic acid (PNA) in concentrated sulfuric acid
Source: Sci Adv. 2025 Mar 26;11(13):eadr0006. doi: 10.1126/sciadv.adr0006 (PMC11939054; doi:10.1126/sciadv.adr0006)

Sample-> CPT22010446-13-C3-24h

Injection Date : Tue, 3. Oct. 2023		Seq Line :	8
		Location :	64
		Inj. Vol. :	2 µl

Acq. Method : C:\Users\Public\Documents\ChemStation\1\Data\SE03OCT 2023-10-03 13-55-25\22010446 LCMS-6.M

Analysis Method : C:\Users\Public\Documents\ChemStation\1\Data\SE03OCT 2023-10-03 13-55-25\22010446 LCMS-6.M (Sequence Method)

Waters XBridge Phenyl (4.6 * 150 mm; 3.5 µm); 0.05% TFA (aq) / AcN: 100/0 (0.0 min) --> (6.0 min) --> 70/30 (0.0 min) --> (2.0 min) --> 10/90 (2.0 min); Flow: 1.0 ml/min; MSD1 = positive; MSD2 = negative

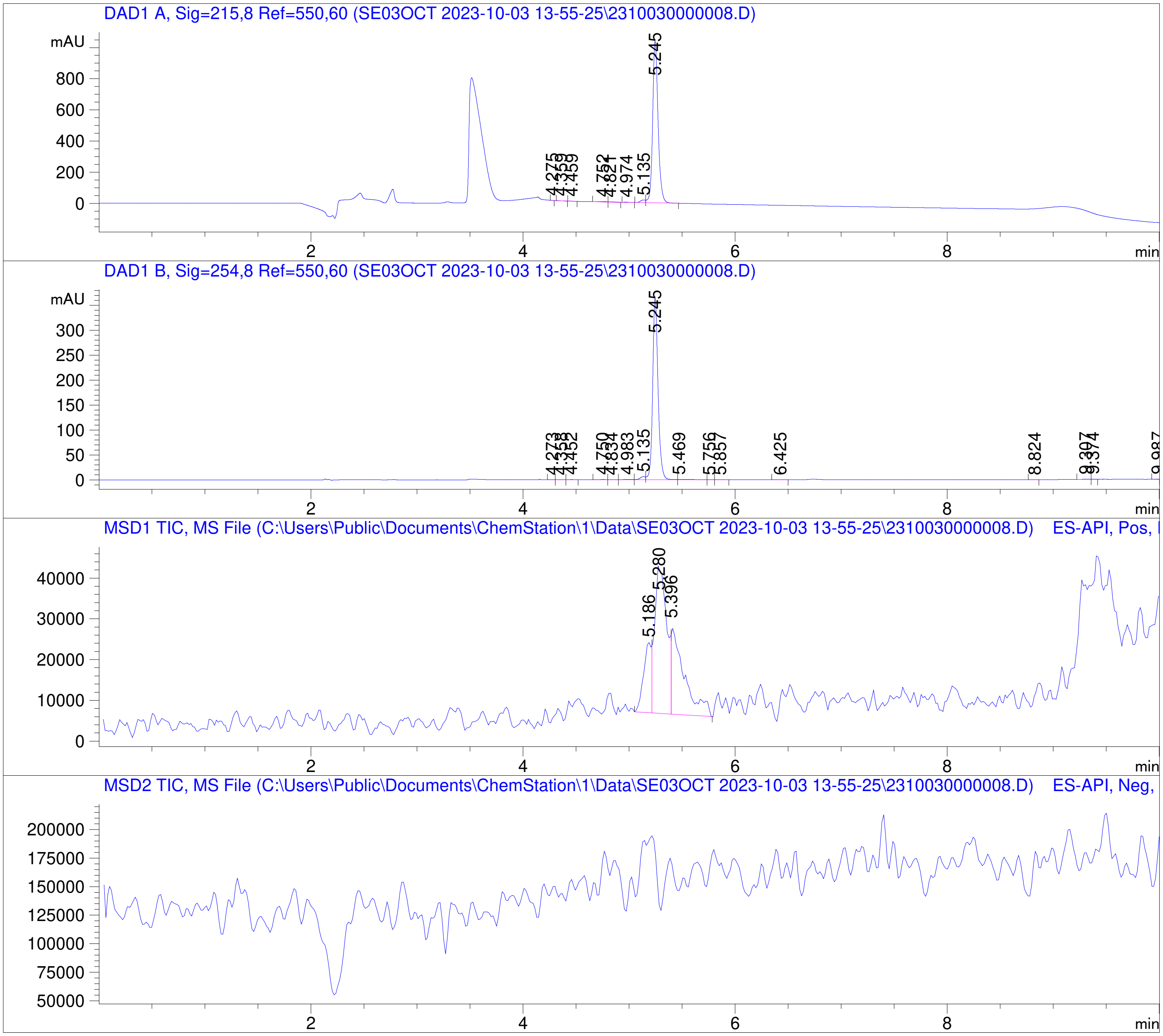

DAD1 A, Sig=215,8 Ref=550,60

Peak #	Ret. Time [min]	Area [mV *s]	Area %
1	4.275	0.372	0.010
2	4.359	1.881	0.050
3	4.459	1.103	0.029
4	4.752	9.999	0.266
5	4.821	6.151	0.164
6	4.974	3.032	0.081
7	5.135	54.135	1.440
8	5.245	3683.298	97.961

DAD1 B, Sig=254,8 Ref=550,60

Peak #	Ret. Time [min]	Area [mV *s]	Area %
1	4.273	0.354	0.027
2	4.358	0.733	0.056
3	4.452	0.782	0.059
4	4.750	1.274	0.097
5	4.834	0.790	0.060
6	4.983	2.930	0.223
7	5.135	20.854	1.585
8	5.245	1281.207	97.358
9	5.469	3.075	0.234
10	5.756	0.124	0.009
11	5.857	0.340	0.026
12	6.425	0.409	0.031
13	8.824	0.152	0.012
14	9.307	1.035	0.079
15	9.374	0.233	0.018
16	9.987	1.685	0.128

Sample-> CPT22010446-13-C3-24h

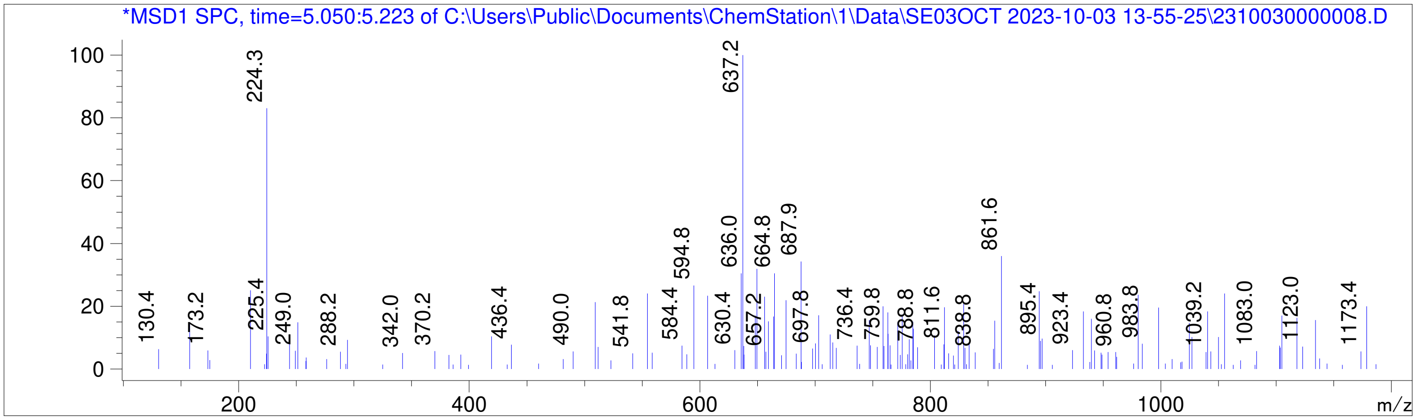

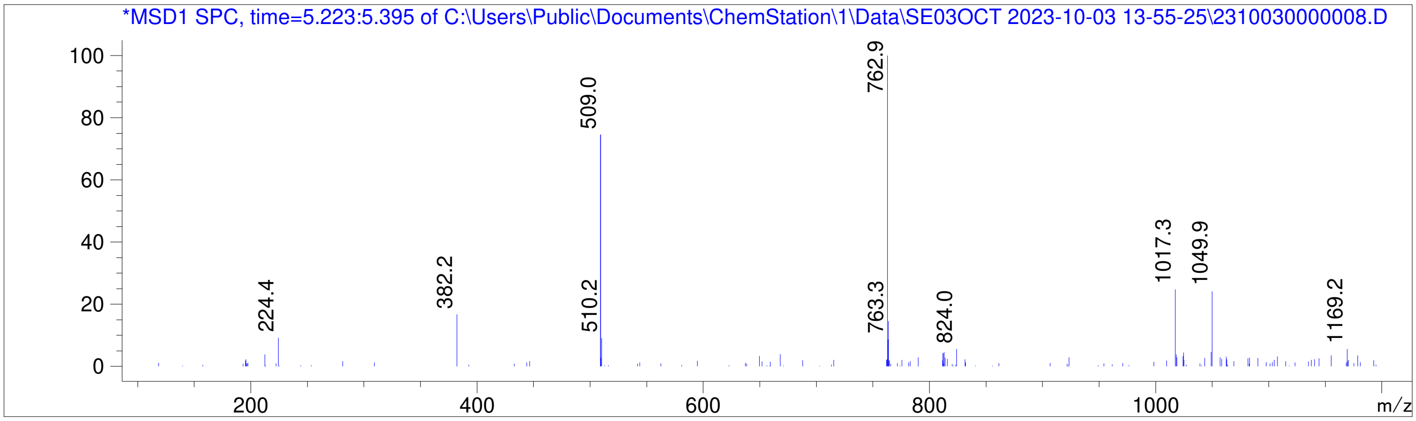

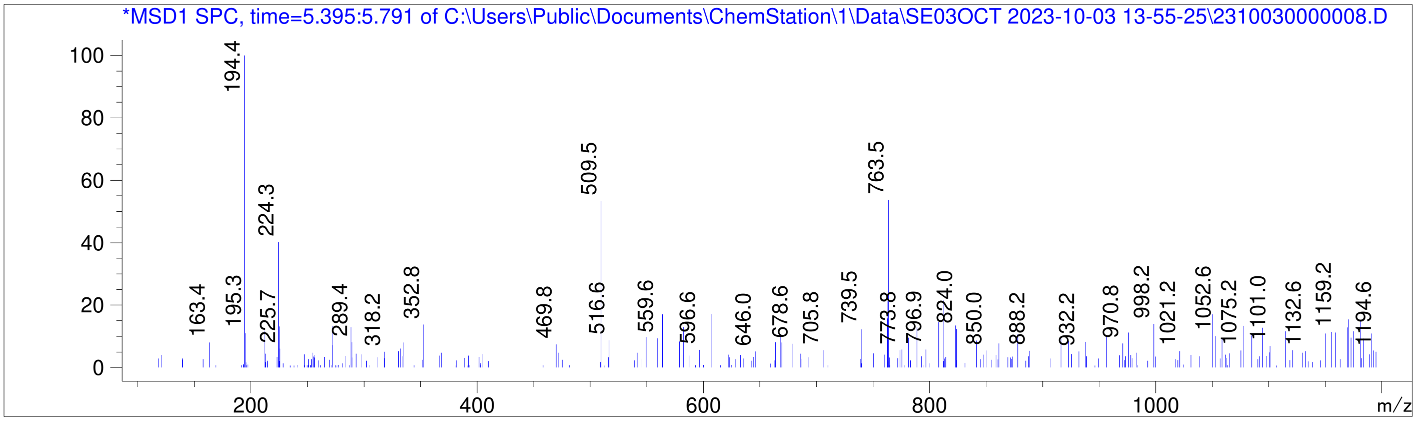

Supplement: Supplementary file 2 — Data S1 and S2 [file sciadv.adr0006_data_s1_and_s2.zip › Supplementary Dataset 1-LCMS DATA/LCMS PNA Hexamers A-T/LCMS C6 RT/24h/CPT22010446-13-C3-24h.pdf]
