# Supplementary material for: Astrobiological implications of the stability and reactivity of peptide nucleic acid (PNA) in concentrated sulfuric acid
Source: Sci Adv. 2025 Mar 26;11(13):eadr0006. doi: 10.1126/sciadv.adr0006 (PMC11939054; doi:10.1126/sciadv.adr0006)
Supplement: Supplementary file 2 — Data S1 and S2 [file sciadv.adr0006_data_s1_and_s2.zip › Supplementary Dataset 1-LCMS DATA/LCMS PNA Hexamers A-T/LCMS C6 RT/24h/CPT22010446-21-A2-24h.pdf]

Data -> C:\Users\Public\Documents\ChemStation\1\Data\SE27SEP 2023-09-27 12-47-15\ ->
Sample-> CPT22010446-21-A2-24h

===

Injection Date : Wed, 27. Sep. 2023

Seq Line : 8
Location : 7
Inj. Vol. : 2 µl

Acq. Method : C:\Users\Public\Documents\ChemStation\1\Data\SE27SEP 2023-09-27 12-47-15\22010446 LCMS-6.M
Analysis Method : C:\Users\Public\Documents\ChemStation\1\Data\SE27SEP 2023-09-27 12-47-15\22010446 LCMS-6.M (Sequence Method)
Waters XBridge Phenyl (4.6 * 150 mm; 3.5 µm); 0.05% TFA (aq) / AcN: 100/0 (0.0 min) --> (6.0 min) --> 70/30 (0.0 min) --> (2.0 min) --> 10/90 (2.0 min); Flow: 1.0 ml/min;
MSD1 = positive; MSD2 = negative

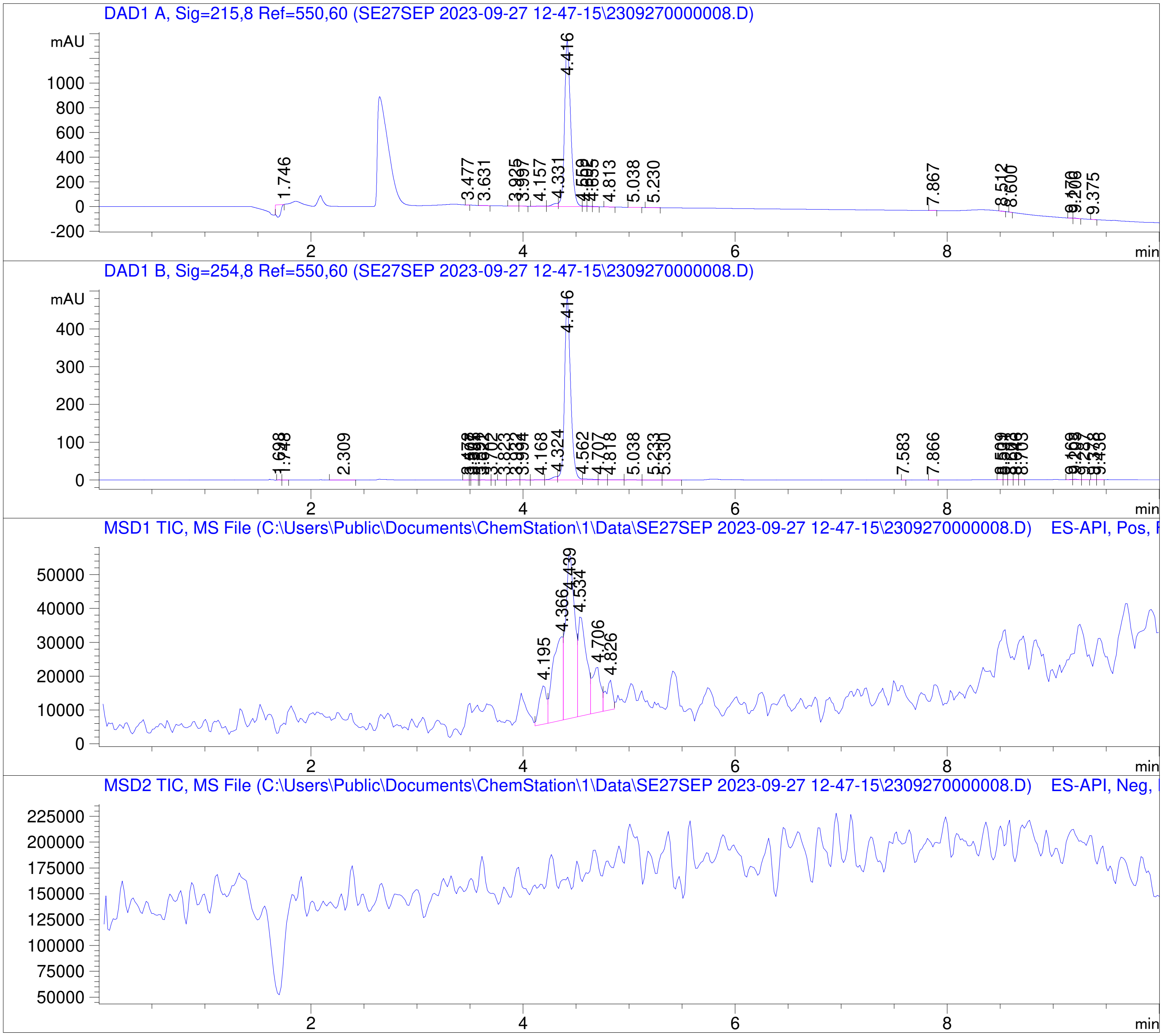

DAD1 A, Sig=215,8 Ref=550,60

Peak #	Ret. Time [min]	Area [mV *s]	Area %
1	1.746	0.019	0.000
2	3.477	0.803	0.015
3	3.631	4.227	0.076
4	3.925	5.628	0.102
5	3.997	3.431	0.062
6	4.157	14.015	0.253
7	4.331	94.954	1.717
8	4.416	5358.695	96.917
9	4.559	12.901	0.233
10	4.602	11.322	0.205
11	4.655	4.276	0.077
12	4.813	1.371	0.025
13	5.038	2.291	0.041
14	5.230	1.569	0.028
15	7.867	0.445	0.008
16	8.512	1.859	0.034
17	8.600	0.391	0.007
18	9.170	3.294	0.060
19	9.206	6.550	0.118
20	9.375	1.108	0.020

DAD1 B, Sig=254,8 Ref=550,60

Peak #	Ret. Time [min]	Area [mV *s]	Area %
1	1.698	1.187	0.060
2	1.748	0.288	0.014
3	2.309	0.961	0.048
4	3.478	0.448	0.023
5	3.507	0.156	0.008
6	3.535	1.088	0.055
7	3.591	0.129	0.006
8	3.622	1.341	0.068
9	3.702	0.033	0.002
10	3.823	0.091	0.005
11	3.932	2.262	0.114
12	3.994	1.536	0.077
13	4.168	4.728	0.238
14	4.324	32.212	1.624
15	4.416	1901.767	95.867
16	4.562	16.460	0.830
17	4.707	4.079	0.206
18	4.818	3.699	0.186
19	5.038	2.198	0.111
20	5.233	1.411	0.071
21	5.330	0.656	0.033
22	7.583	0.033	0.002
23	7.866	0.282	0.014
24	8.509	0.329	0.017
25	8.531	0.288	0.015
26	8.603	0.265	0.013
27	8.646	0.199	0.010
28	8.703	0.123	0.006

Peak #	Ret. Time [min]	Area [mV *s]	Area %
29	9.169	1.317	0.066
30	9.205	3.259	0.164
31	9.287	0.488	0.025
32	9.378	0.160	0.008
33	9.436	0.278	0.014

*MSD1 SPC, time=4.120:4.241 of C:\Users\Public\Documents\ChemStation\1\Data\SE27SEP 2023-09-27 12-47-15\2309270000008.D

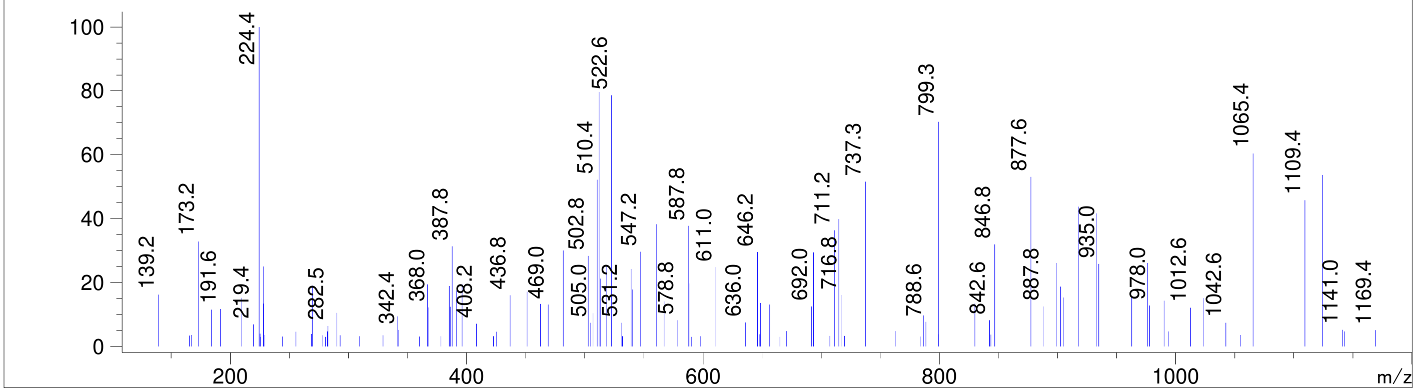

*MSD1 SPC, time=4.241:4.379 of C:\Users\Public\Documents\ChemStation\1\Data\SE27SEP 2023-09-27 12-47-15\2309270000008.D

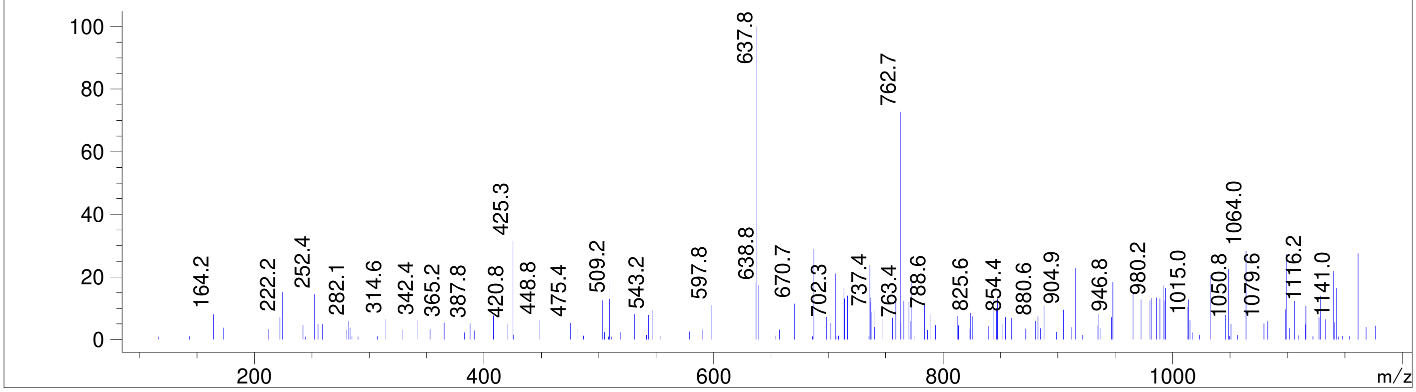

*MSD1 SPC, time=4.379:4.516 of C:\Users\Public\Documents\ChemStation\1\Data\SE27SEP 2023-09-27 12-47-15\2309270000008.D

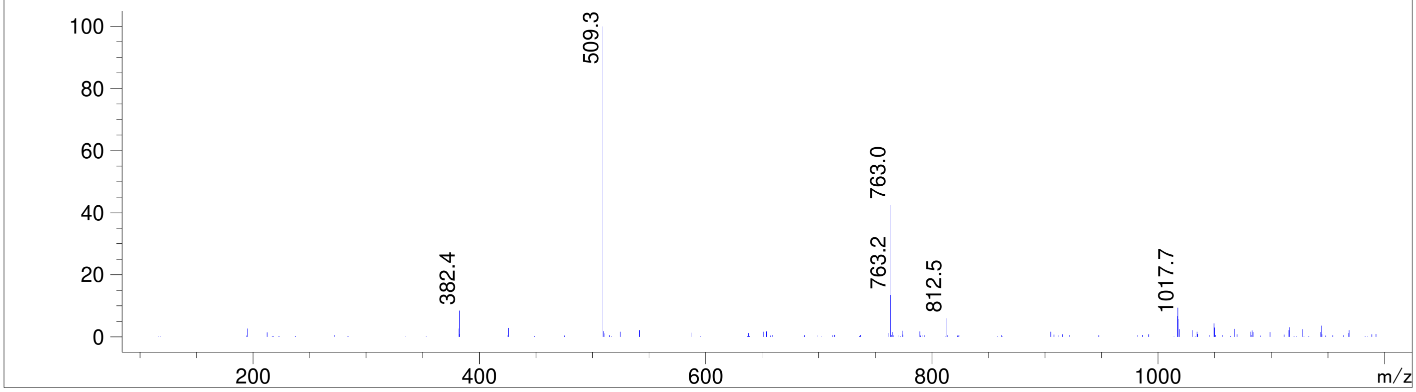

==

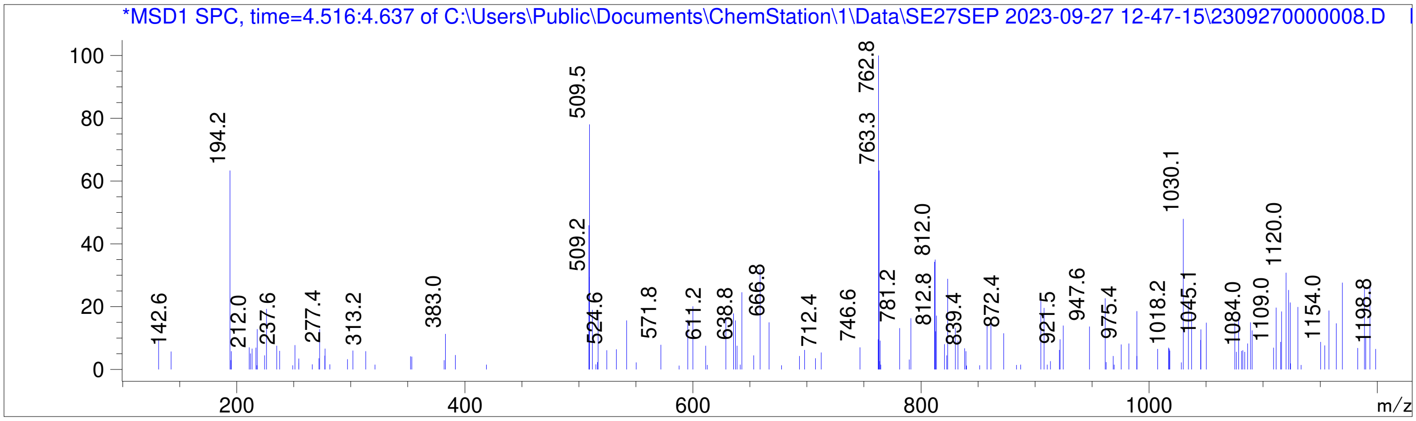
*MSD1 SPC, time=4.516:4.637 of C:\Users\Public\Documents\ChemStation\1\Data\SE27SEP 2023-09-27 12-47-15\2309270000008.D
100
80
60
40
20
0
142.6
194.2
212.0
237.6
277.4
313.2
383.0
509.5
509.2
524.6
571.8
611.2
638.8
666.8
712.4
746.6
762.8
763.3
781.2
812.0
812.8
839.4
872.4
921.5
947.6
975.4
1018.2
1030.1
1045.1
1084.0
1109.0
1120.0
1154.0
1198.8
200
400
600
800
1000
m/z

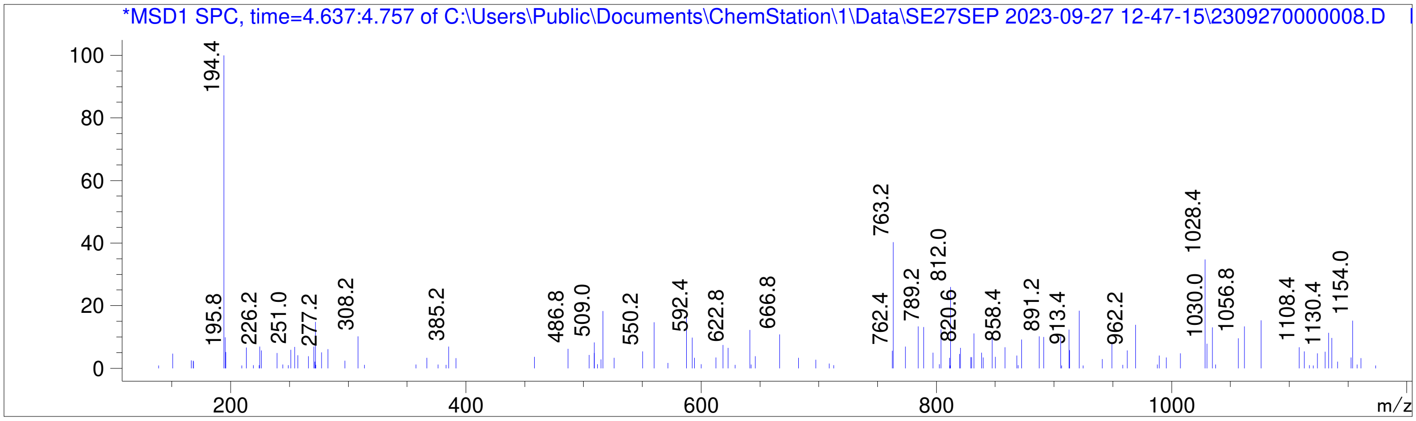
*MSD1 SPC, time=4.637:4.757 of C:\Users\Public\Documents\ChemStation\1\Data\SE27SEP 2023-09-27 12-47-15\2309270000008.D
100
80
60
40
20
0
194.4
195.8
226.2
251.0
277.2
308.2
385.2
486.8
509.0
550.2
592.4
622.8
666.8
762.4
763.2
789.2
812.0
820.6
858.4
891.2
913.4
962.2
1028.4
1030.0
1056.8
1108.4
1130.4
1154.0
200
400
600
800
1000
m/z

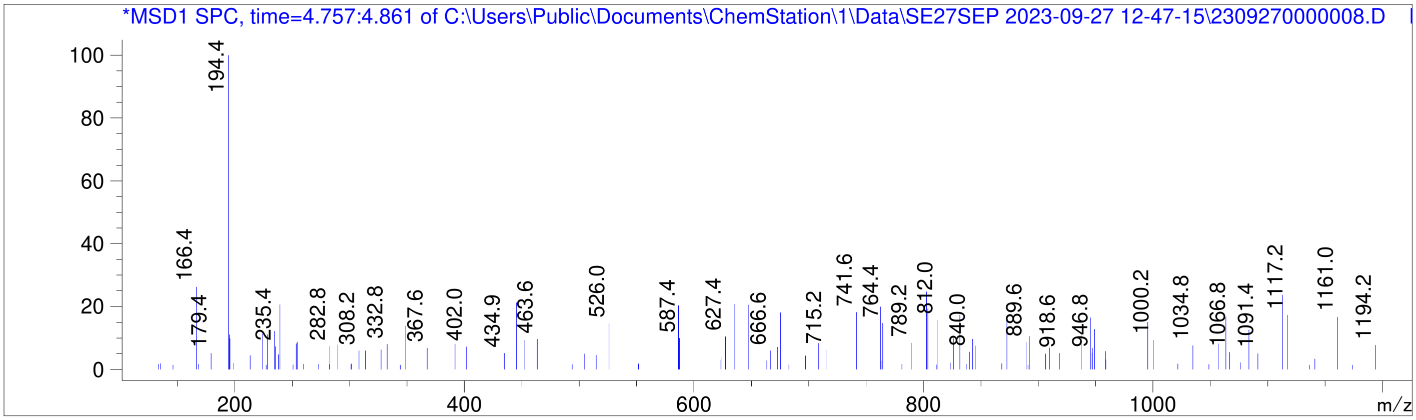
*MSD1 SPC, time=4.757:4.861 of C:\Users\Public\Documents\ChemStation\1\Data\SE27SEP 2023-09-27 12-47-15\2309270000008.D
100
80
60
40
20
0
194.4
166.4
179.4
235.4
282.8
308.2
332.8
367.6
402.0
434.9
463.6
526.0
587.4
627.4
666.6
715.2
741.6
764.4
789.2
812.0
840.0
889.6
918.6
946.8
1000.2
1034.8
1066.8
1091.4
1117.2
1161.0
1194.2
200
400
600
800
1000
m/z
