# Supplementary material for: Astrobiological implications of the stability and reactivity of peptide nucleic acid (PNA) in concentrated sulfuric acid
Source: Sci Adv. 2025 Mar 26;11(13):eadr0006. doi: 10.1126/sciadv.adr0006 (PMC11939054; doi:10.1126/sciadv.adr0006)
Supplement: Supplementary file 2 — Data S1 and S2 [file sciadv.adr0006_data_s1_and_s2.zip › Supplementary Dataset 1-LCMS DATA/LCMS PNA Hexamers A-T/LCMS C6 RT/24h/CPT22010446-21-A-24h (LCMS-6).pdf]

Data -> C:\Users\Public\Documents\ChemStation\1\Data\09. September\SE26SEP\SE26SEP 202->
Sample-> CPT22010446-21-A-24h

===

Injection Date	:	Tue, 26. Sep. 2023	Seq Line	:	29
			Location	:	84
			Inj. Vol.	:	2 µl

Acq. Method : C:\Users\Public\Documents\ChemStation\1\Data\SE26SEP 2023-09-26 16-24-18\22010446 LCMS-6.M
Analysis Method : C:\Users\Public\Documents\ChemStation\1\Data\09. September\SE26SEP\SE26SEP 2023-09-26 16-24-18\22010446 LCMS-6.M (Sequence->
Waters XBridge Phenyl (4.6 * 150 mm; 3.5 µm); 0.05% TFA (aq) / AcN: 100/0 (0.0 min) --> (6.0 min) --> 70/30 (0.0 min) --> (2.0 min) --> 10/90 (2.0 min); Flow: 1.0 ml/min;
MSD1 = positive; MSD2 = negative

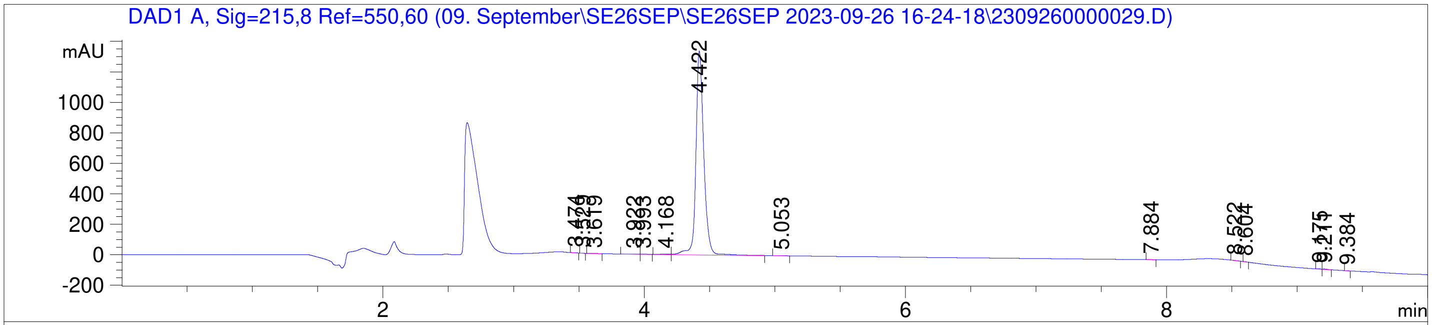

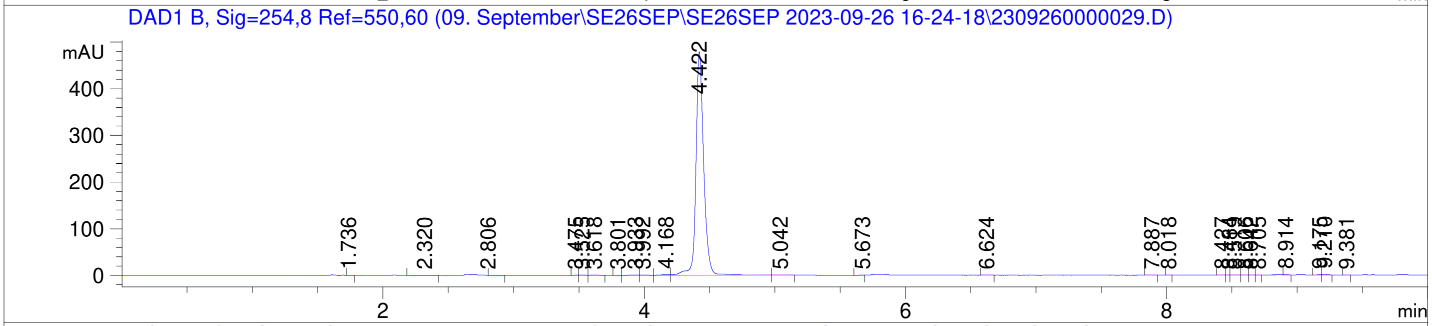

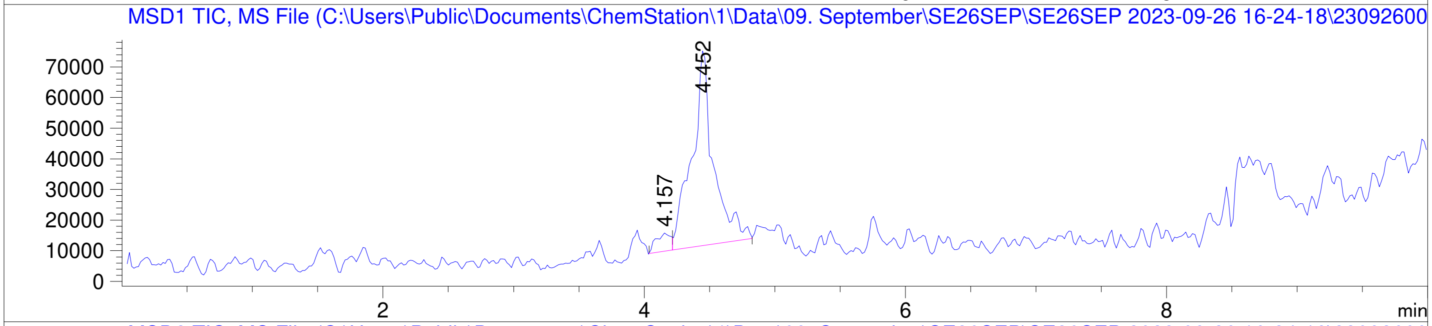

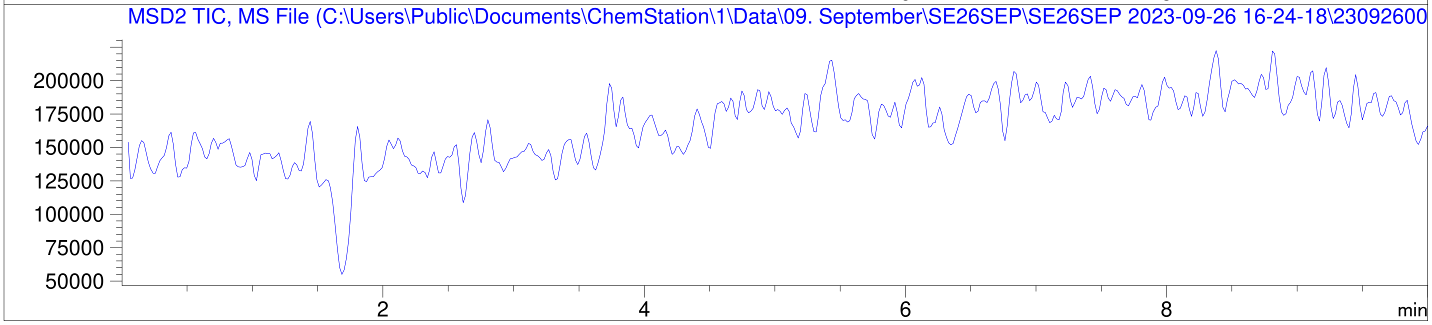

DAD1 A, Sig=215,8 Ref=550,60

Peak #	Ret. Time [min]	Area [mV *s]	Area %
1	3.474	1.834	0.033
2	3.529	0.544	0.010
3	3.619	5.833	0.104
4	3.922	7.627	0.136
5	3.993	4.096	0.073
6	4.168	14.851	0.264
7	4.422	5564.728	99.098
8	5.053	2.281	0.041
9	7.884	0.507	0.009
10	8.522	1.906	0.034
11	8.604	0.548	0.010
12	9.175	3.156	0.056
13	9.211	7.148	0.127
14	9.384	0.334	0.006

DAD1 B, Sig=254,8 Ref=550,60

Peak #	Ret. Time [min]	Area [mV *s]	Area %
1	1.736	0.227	0.011
2	2.320	1.264	0.063
3	2.806	1.046	0.052
4	3.475	0.650	0.033
5	3.525	1.398	0.070
6	3.618	1.865	0.094
7	3.801	0.075	0.004
8	3.933	2.670	0.134
9	3.992	1.956	0.098
10	4.168	5.239	0.263
11	4.422	1968.923	98.729
12	5.042	1.055	0.053
13	5.673	0.196	0.010
14	6.624	0.169	0.008
15	7.887	0.270	0.014
16	8.018	0.062	0.003
17	8.427	0.241	0.012
18	8.484	0.094	0.005
19	8.519	0.779	0.039
20	8.606	0.386	0.019
21	8.642	0.231	0.012
22	8.705	0.123	0.006
23	8.914	0.480	0.024
24	9.175	1.336	0.067
25	9.210	3.420	0.172
26	9.381	0.126	0.006

==

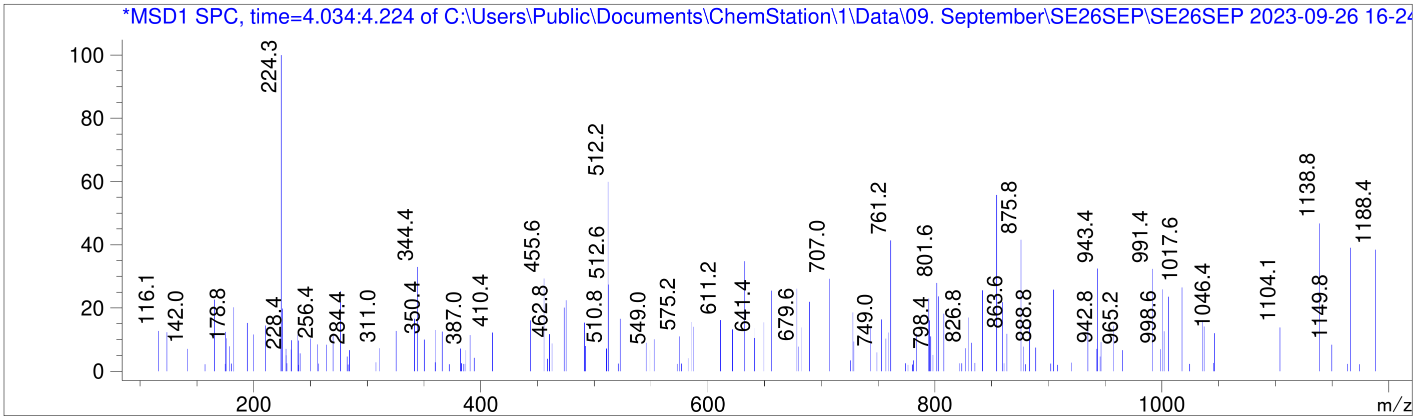
*MSD1 SPC, time=4.034:4.224 of C:\Users\Public\Documents\ChemStation\1\Data\09. September\SE26SEP\SE26SEP 2023-09-26 16-2
224.3
512.2
761.2
875.8
1138.8
1188.4
344.4
455.6
512.6
707.0
801.6
943.4
991.4
1017.6
116.1
142.0
178.8
228.4
256.4
284.4
311.0
350.4
387.0
410.4
462.8
510.8
549.0
575.2
611.2
641.4
679.6
749.0
798.4
826.8
863.6
888.8
942.8
965.2
998.6
1046.4
1104.1
1149.8
m/z

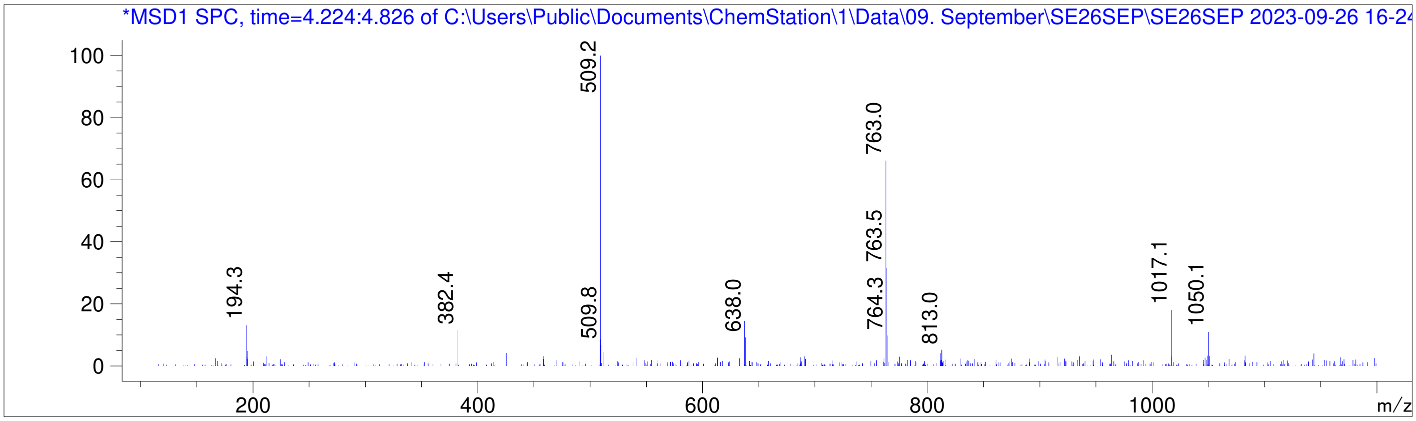
*MSD1 SPC, time=4.224:4.826 of C:\Users\Public\Documents\ChemStation\1\Data\09. September\SE26SEP\SE26SEP 2023-09-26 16-2
509.2
763.0
763.5
194.3
382.4
509.8
638.0
764.3
813.0
1017.1
1050.1
m/z
