# Supplementary material for: Astrobiological implications of the stability and reactivity of peptide nucleic acid (PNA) in concentrated sulfuric acid
Source: Sci Adv. 2025 Mar 26;11(13):eadr0006. doi: 10.1126/sciadv.adr0006 (PMC11939054; doi:10.1126/sciadv.adr0006)
Supplement: Supplementary file 2 — Data S1 and S2 [file sciadv.adr0006_data_s1_and_s2.zip › Supplementary Dataset 1-LCMS DATA/LCMS PNA Hexamers A-T/LCMS G6 50C_80C/50C/1h/CPT22010446-20-B1-50deg-1h.pdf]

Data -> C:\Users\Public\Documents\ChemStation\1\Data\SE17OCT 2023-10-17 07-53-29\ ->
Sample-> CPT22010446-20-B1-50deg-1h

==

Injection Date : Tue, 17. Oct. 2023   Seq Line : 21
Location : 30
Inj. Vol. : 2 µl

Acq. Method : C:\Users\Public\Documents\ChemStation\1\Data\SE17OCT 2023-10-17 07-53-29\22010446 LCMS-6.M
Analysis Method : C:\Users\Public\Documents\ChemStation\1\Data\SE17OCT 2023-10-17 07-53-29\22010446 LCMS-6.M (Sequence Method)
Waters XBridge Phenyl (4.6 * 150 mm; 3.5 µm); 0.05% TFA (aq) / AcN: 100/0 (0.0 min) --> (6.0 min) --> 70/30 (0.0 min) --> (2.0 min) --> 10/90 (2.0 min); Flow: 1.0 ml/min;
MSD1 = positive; MSD2 = negative

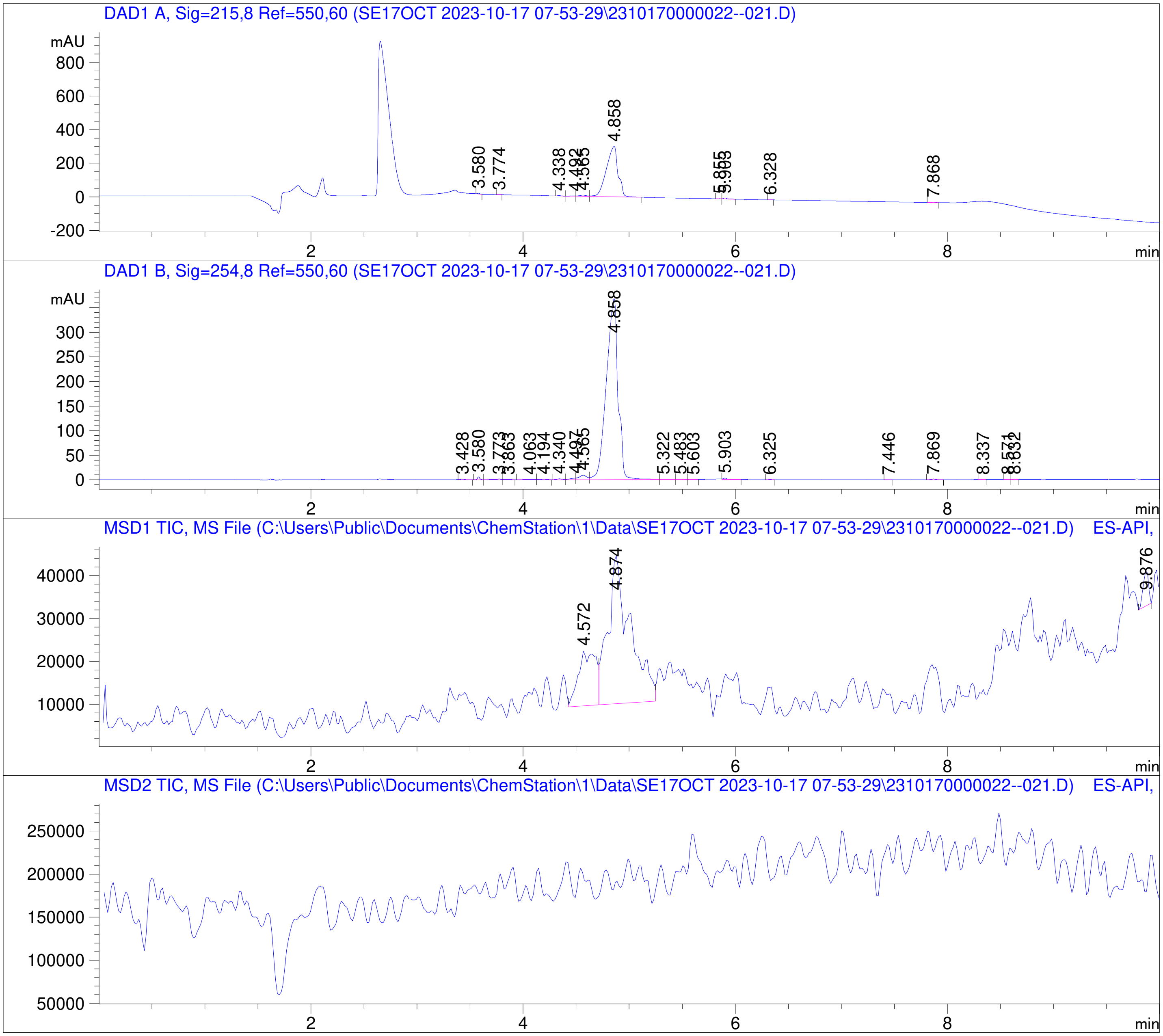

DAD1 A, Sig=215,8 Ref=550,60

Peak #	Ret. Time [min]	Area [mV *s]	Area %
1	3.580	5.483	0.231
2	3.774	1.520	0.064
3	4.338	5.540	0.233
4	4.492	5.882	0.248
5	4.565	35.062	1.476
6	4.858	2299.209	96.810
7	5.855	2.331	0.098
8	5.903	13.702	0.577
9	6.328	0.450	0.019
10	7.868	5.790	0.244

DAD1 B, Sig=254,8 Ref=550,60

Peak #	Ret. Time [min]	Area [mV *s]	Area %
1	3.428	3.441	0.117
2	3.580	8.856	0.301
3	3.773	5.430	0.185
4	3.863	2.707	0.092
5	4.063	3.695	0.126
6	4.194	4.003	0.136
7	4.340	5.631	0.192
8	4.497	10.570	0.360
9	4.565	46.092	1.569
10	4.858	2826.171	96.211
11	5.322	5.577	0.190
12	5.483	2.555	0.087
13	5.603	0.601	0.020
14	5.903	7.939	0.270
15	6.325	0.239	0.008
16	7.446	0.137	0.005
17	7.869	3.021	0.103
18	8.337	0.123	0.004
19	8.571	0.318	0.011
20	8.632	0.378	0.013

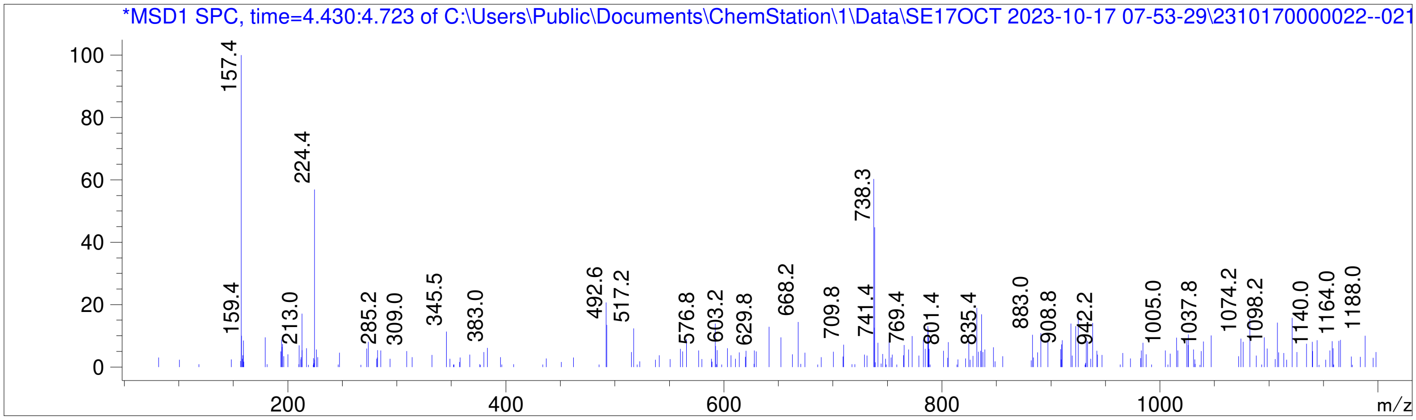
*MSD1 SPC, time=4.430:4.723 of C:\Users\Public\Documents\ChemStation\1\Data\SE17OCT 2023-10-17 07-53-29\2310170000022--021
157.4
224.4
738.3
159.4
213.0
285.2
309.0
345.5
383.0
492.6
517.2
576.8
603.2
629.8
668.2
709.8
741.4
769.4
801.4
835.4
883.0
908.8
942.2
1005.0
1037.8
1074.2
1098.2
1140.0
1164.0
1188.0
m/z

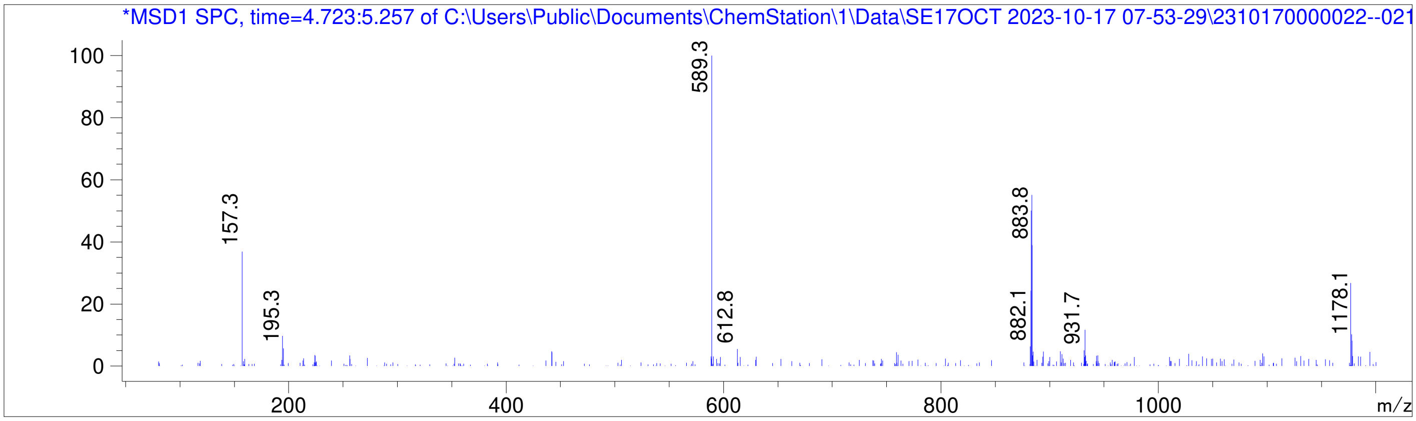
*MSD1 SPC, time=4.723:5.257 of C:\Users\Public\Documents\ChemStation\1\Data\SE17OCT 2023-10-17 07-53-29\2310170000022--021
589.3
157.3
195.3
612.8
883.8
882.1
931.7
1178.1
m/z

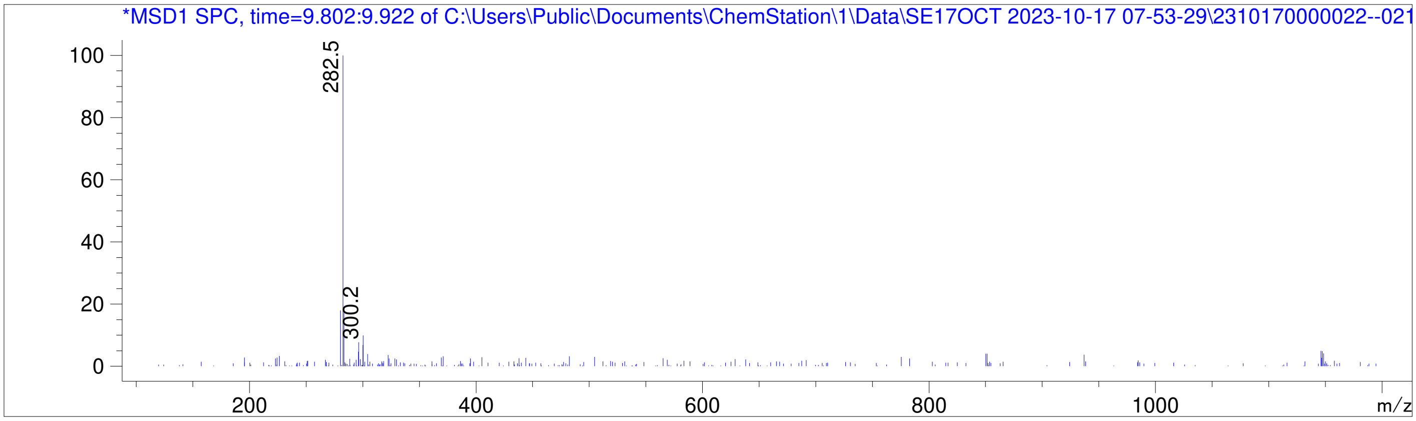
*MSD1 SPC, time=9.802:9.922 of C:\Users\Public\Documents\ChemStation\1\Data\SE17OCT 2023-10-17 07-53-29\2310170000022--021
282.5
300.2
m/z
