# Supplementary material for: Astrobiological implications of the stability and reactivity of peptide nucleic acid (PNA) in concentrated sulfuric acid
Source: Sci Adv. 2025 Mar 26;11(13):eadr0006. doi: 10.1126/sciadv.adr0006 (PMC11939054; doi:10.1126/sciadv.adr0006)
Supplement: Supplementary file 2 — Data S1 and S2 [file sciadv.adr0006_data_s1_and_s2.zip › Supplementary Dataset 1-LCMS DATA/LCMS PNA Hexamers A-T/LCMS G6 50C_80C/50C/1h/CPT22010446-20-B2-50deg-1h.pdf]

Data -> C:\Users\Public\Documents\ChemStation\1\Data\SE01NOV 2023-11-01 15-33-53\ ->
Sample-> CPT22010446-20-B2-50deg-1h

===

Injection Date :	Thu, 2. Nov. 2023	Seq Line :	48
		Location :	18
		Inj. Vol. :	2 µl

Acq. Method : C:\Users\Public\Documents\ChemStation\1\Data\SE01NOV 2023-11-01 15-33-53\22010446 LCMS-6.M

Analysis Method : C:\Users\Public\Documents\ChemStation\1\Data\SE01NOV 2023-11-01 15-33-53\22010446 LCMS-6.M (Sequence Method)

Waters XBridge Phenyl (4.6 * 150 mm; 3.5 µm); 0.05% TFA (aq) / AcN: 100/0 (0.0 min) --> (6.0 min) --> 70/30 (0.0 min) --> (2.0 min) --> 10/90 (2.0 min); Flow: 1.0 ml/min;
MSD1 = positive; MSD2 = negative

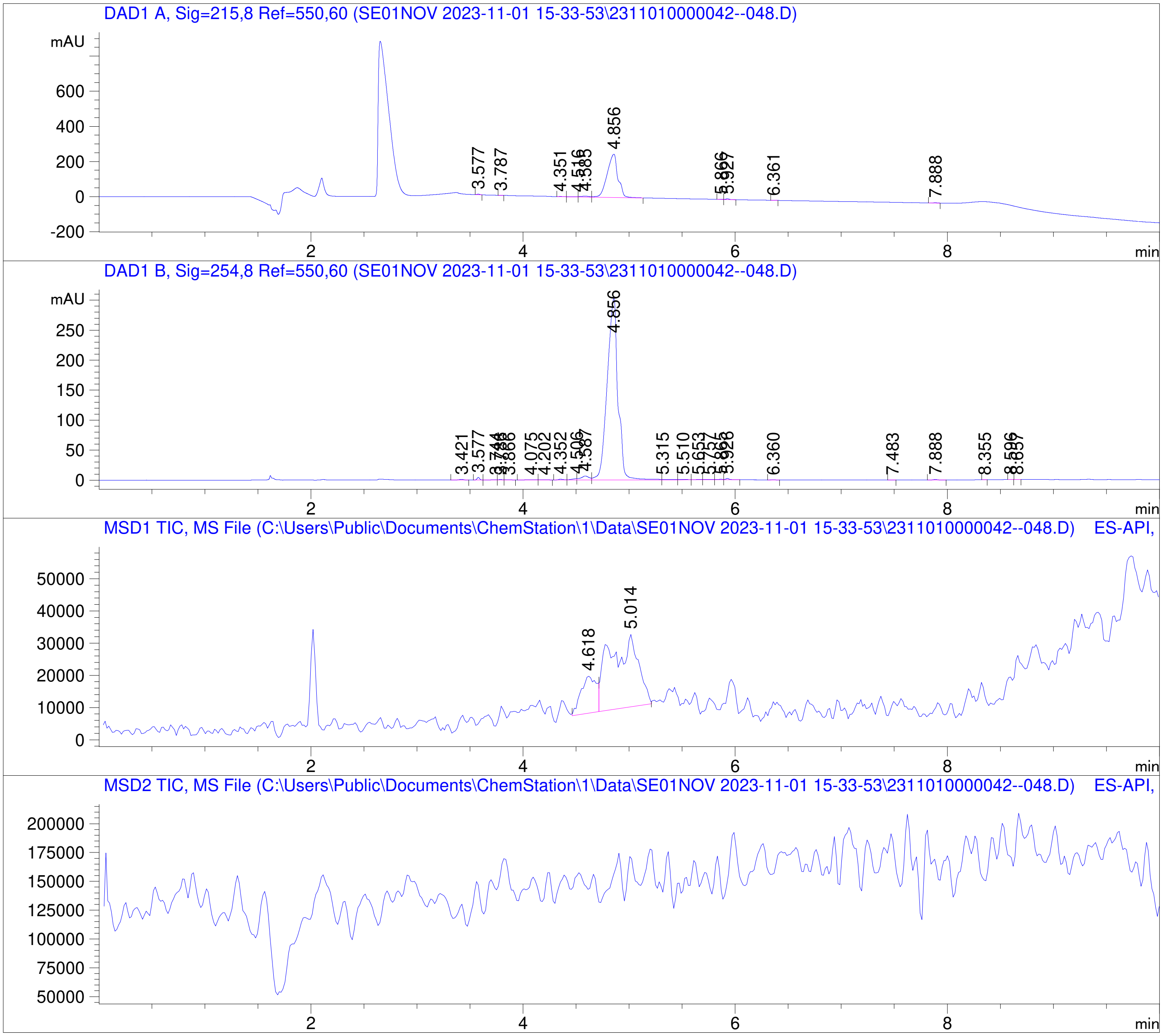

DAD1 A, Sig=215,8 Ref=550,60

Peak #	Ret. Time [min]	Area [mV *s]	Area %
1	3.577	5.437	0.303
2	3.787	0.773	0.043
3	4.351	3.401	0.189
4	4.516	8.373	0.466
5	4.585	29.823	1.660
6	4.856	1733.669	96.469
7	5.866	1.553	0.086
8	5.927	9.521	0.530
9	6.361	0.524	0.029
10	7.888	4.048	0.225

DAD1 B, Sig=254,8 Ref=550,60

Peak #	Ret. Time [min]	Area [mV *s]	Area %
1	3.421	3.235	0.145
2	3.577	7.063	0.316
3	3.744	1.749	0.078
4	3.786	2.215	0.099
5	3.866	2.105	0.094
6	4.075	3.088	0.138
7	4.202	3.187	0.143
8	4.352	4.103	0.184
9	4.506	6.808	0.305
10	4.587	35.284	1.578
11	4.856	2136.444	95.574
12	5.315	6.721	0.301
13	5.510	4.008	0.179
14	5.653	2.615	0.117
15	5.757	3.681	0.165
16	5.865	3.729	0.167
17	5.926	6.101	0.273
18	6.360	0.298	0.013
19	7.483	0.131	0.006
20	7.888	2.198	0.098
21	8.355	0.103	0.005
22	8.596	0.255	0.011
23	8.657	0.259	0.012

==

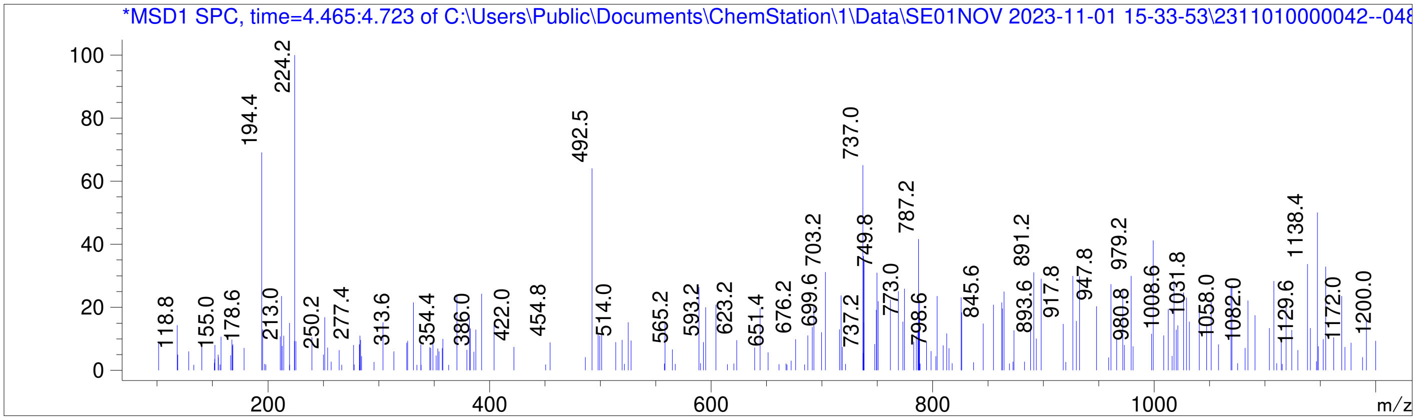
*MSD1 SPC, time=4.465:4.723 of C:\Users\Public\Documents\ChemStation\1\Data\SE01NOV 2023-11-01 15-33-53\2311010000042--048
118.8
155.0
178.6
194.4
213.0
224.2
250.2
277.4
313.6
354.4
386.0
422.0
454.8
492.5
514.0
565.2
593.2
623.2
651.4
676.2
699.6
703.2
737.2
737.0
749.8
773.0
787.2
798.6
845.6
893.6
891.2
917.8
947.8
980.8
979.2
1008.6
1031.8
1058.0
1082.0
1129.6
1138.4
1172.0
1200.0
m/z

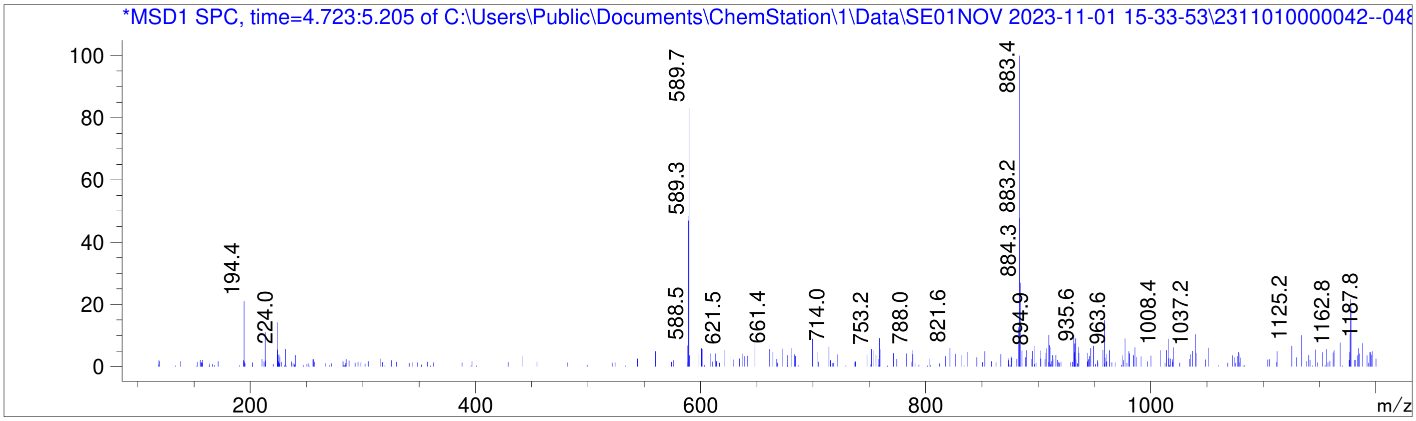
*MSD1 SPC, time=4.723:5.205 of C:\Users\Public\Documents\ChemStation\1\Data\SE01NOV 2023-11-01 15-33-53\2311010000042--048
194.4
224.0
588.5
589.3
589.7
621.5
661.4
714.0
753.2
788.0
821.6
884.3
883.2
883.4
894.9
935.6
963.6
1008.4
1037.2
1125.2
1162.8
1187.8
m/z
