# Supplementary material for: Astrobiological implications of the stability and reactivity of peptide nucleic acid (PNA) in concentrated sulfuric acid
Source: Sci Adv. 2025 Mar 26;11(13):eadr0006. doi: 10.1126/sciadv.adr0006 (PMC11939054; doi:10.1126/sciadv.adr0006)

Data -> C:\Users\Public\Documents\ChemStation\1\Data\SE17OCT 2023-10-17 16-51-41\ ->
Sample-> CPT22010446-20-B1-50deg-1h

Injection Date : Tue, 17. Oct. 2023	Seq Line	:	6
	Location	:	50
	Inj. Vol.	:	2 µl

Acq. Method : C:\Users\Public\Documents\ChemStation\1\Data\SE17OCT 2023-10-17 16-51-41\22010446 LCMS-6.M
Analysis Method : C:\Users\Public\Documents\ChemStation\1\Data\SE17OCT 2023-10-17 16-51-41\22010446 LCMS-6.M (Sequence Method)
Waters XBridge Phenyl (4.6 * 150 mm; 3.5 µm); 0.05% TFA (aq) / AcN: 100/0 (0.0 min) --> (6.0 min) --> 70/30 (0.0 min) --> (2.0 min) --> 10/90 (2.0 min); Flow: 1.0 ml/min;
MSD1 = positive; MSD2 = negative

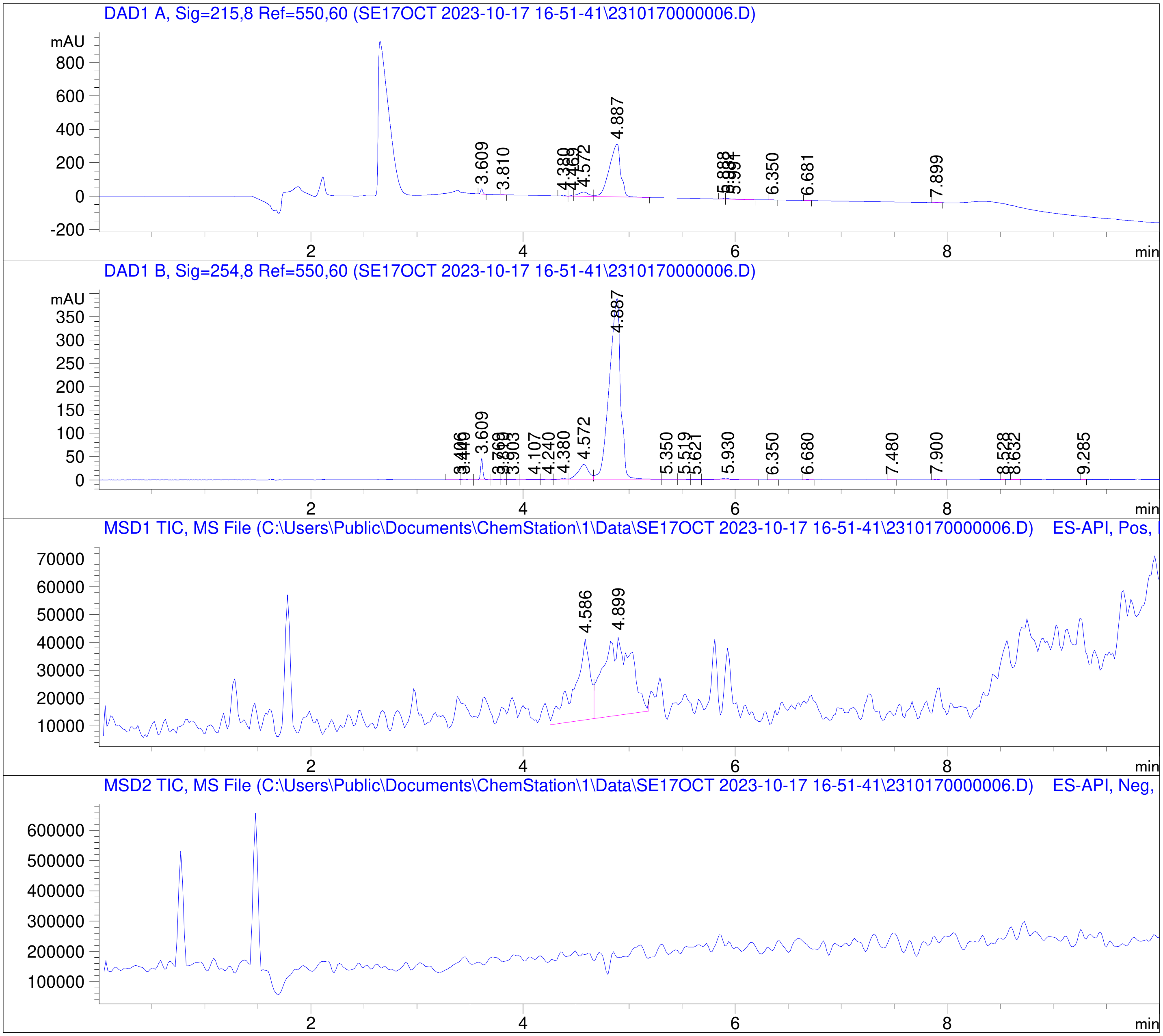

DAD1 A, Sig=215,8 Ref=550,60

Peak #	Ret. Time [min]	Area [mV *s]	Area %
1	3.609	44.657	1.706
2	3.810	1.343	0.051
3	4.380	9.033	0.345
4	4.469	10.138	0.387
5	4.572	170.852	6.528
6	4.887	2353.240	89.909
7	5.888	6.188	0.236
8	5.932	9.807	0.375
9	5.991	6.757	0.258
10	6.350	0.652	0.025
11	6.681	1.123	0.043
12	7.899	3.555	0.136

DAD1 B, Sig=254,8 Ref=550,60

Peak #	Ret. Time [min]	Area [mV *s]	Area %
1	3.406	1.084	0.033
2	3.440	4.203	0.128
3	3.609	67.436	2.060
4	3.769	2.543	0.078
5	3.810	2.767	0.085
6	3.903	3.220	0.098
7	4.107	5.586	0.171
8	4.240	5.914	0.181
9	4.380	13.425	0.410
10	4.572	230.116	7.031
11	4.887	2898.770	88.570
12	5.350	8.451	0.258
13	5.519	5.034	0.154
14	5.621	2.575	0.079
15	5.930	18.414	0.563
16	6.350	0.250	0.008
17	6.680	0.402	0.012
18	7.480	0.114	0.003
19	7.900	1.804	0.055
20	8.528	0.163	0.005
21	8.632	0.373	0.011
22	9.285	0.216	0.007

Sample-> CPT22010446-20-B1-50deg-1h

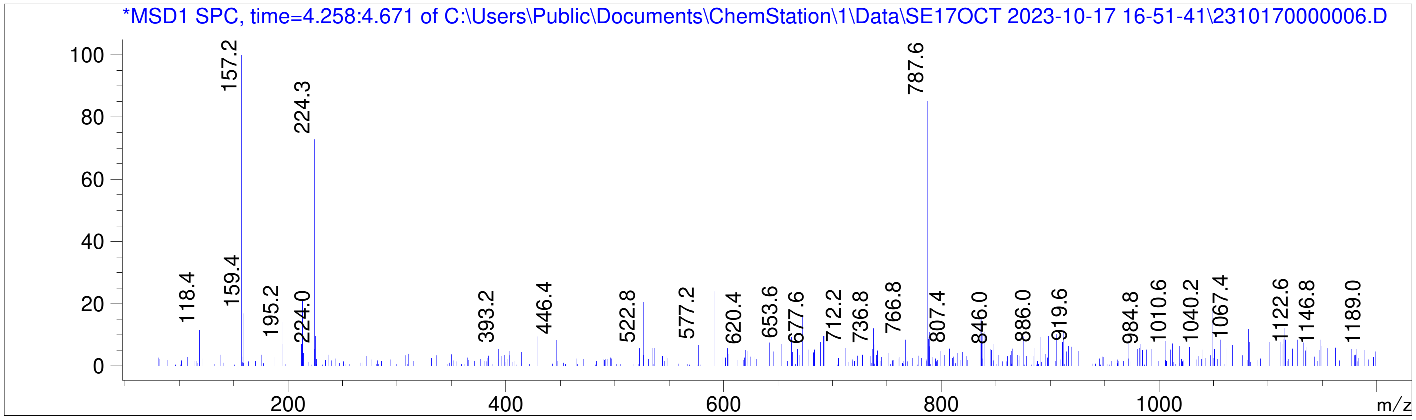

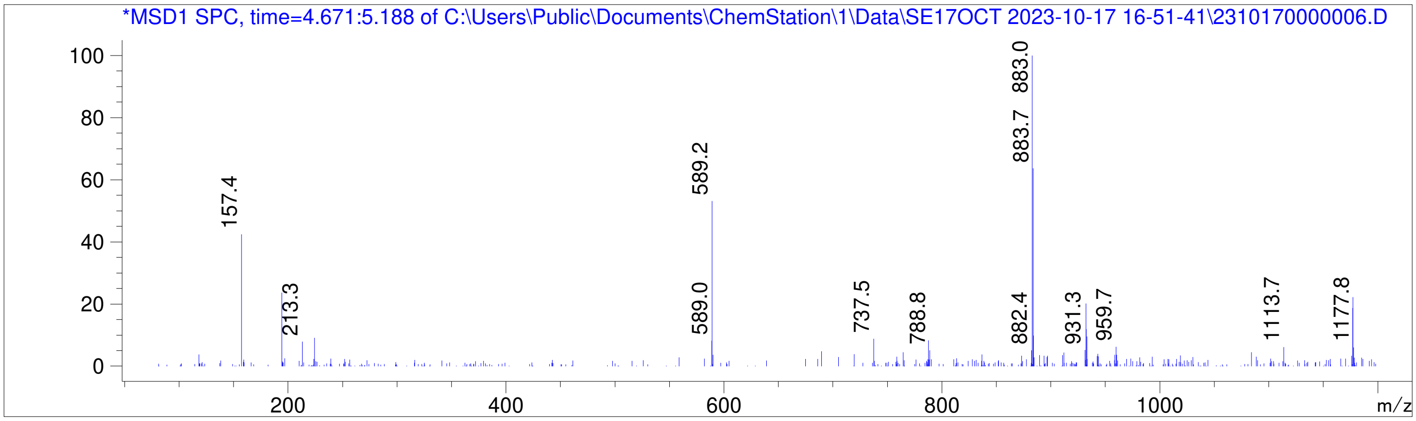

Supplement: Supplementary file 2 — Data S1 and S2 [file sciadv.adr0006_data_s1_and_s2.zip › Supplementary Dataset 1-LCMS DATA/LCMS PNA Hexamers A-T/LCMS G6 50C_80C/50C/24h/CPT22010446-20-B1-50deg-24h.pdf]
