# Supplementary material for: Astrobiological implications of the stability and reactivity of peptide nucleic acid (PNA) in concentrated sulfuric acid
Source: Sci Adv. 2025 Mar 26;11(13):eadr0006. doi: 10.1126/sciadv.adr0006 (PMC11939054; doi:10.1126/sciadv.adr0006)

Data -> C:\USERS\PUBLIC\DOCUMENTS\CHEMSTATION\1\DATA\SE02NOV 2023-11-02 14-31-42\ ->
Sample-> CPT22010446-20-B2-50deg-24h

Injection Date	:	Thu, 2. Nov. 2023	Seq Line	:	13
			Location	:	14
			Inj. Vol.	:	2 µl

Acq. Method : C:\Users\Public\Documents\ChemStation\1\Data\SE02NOV 2023-11-02 14-31-42\22010446 LCMS-6.M
Analysis Method : C:\Users\Public\Documents\ChemStation\1\Data\SE02NOV 2023-11-02 14-31-42\22010446 LCMS-6.M (Sequence Method)
Waters XBridge Phenyl (4.6 * 150 mm; 3.5 µm); 0.05% TFA (aq) / AcN: 100/0 (0.0 min) --> (6.0 min) --> 70/30 (0.0 min) --> (2.0 min) --> 10/90 (2.0 min); Flow: 1.0 ml/min;
MSD1 = positive; MSD2 = negative

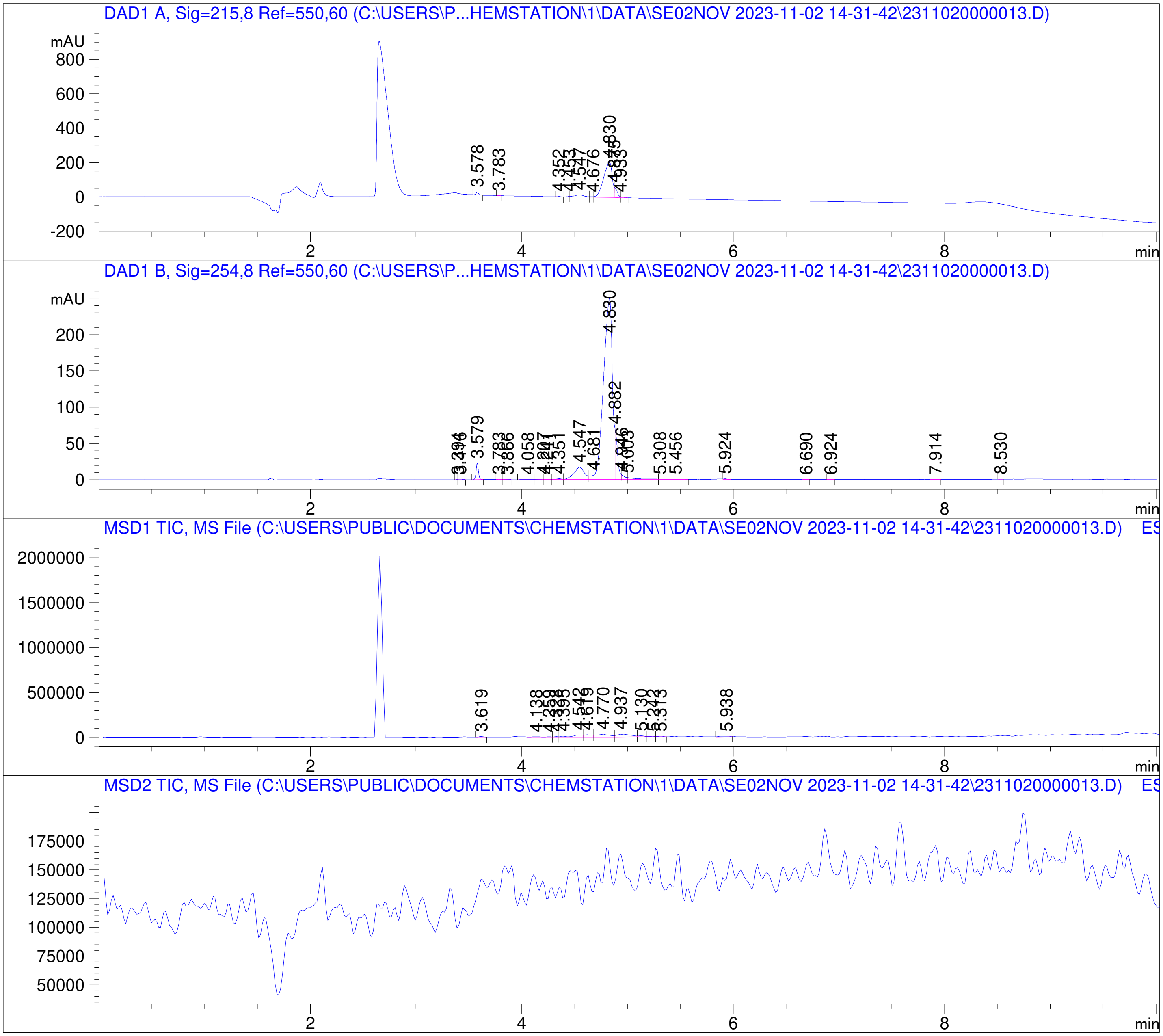

DAD1 A, Sig=215,8 Ref=550,60

Peak #	Ret. Time [min]	Area [mV *s]	Area %
1	3.578	26.029	1.875
2	3.783	0.472	0.034
3	4.352	3.753	0.270
4	4.453	4.548	0.328
5	4.547	82.107	5.915
6	4.676	6.508	0.469
7	4.830	1147.846	82.685
8	4.875	108.429	7.811
9	4.933	8.527	0.614

DAD1 B, Sig=254,8 Ref=550,60

Peak #	Ret. Time [min]	Area [mV *s]	Area %
1	3.394	0.345	0.019
2	3.416	1.872	0.105
3	3.579	34.944	1.969
4	3.783	0.688	0.039
5	3.866	0.729	0.041
6	4.058	1.657	0.093
7	4.207	1.436	0.081
8	4.241	2.272	0.128
9	4.351	4.953	0.279
10	4.547	115.569	6.511
11	4.681	16.480	0.928
12	4.830	1439.651	81.105
13	4.882	115.421	6.502
14	4.946	13.391	0.754
15	5.003	17.507	0.986
16	5.308	3.452	0.194
17	5.456	1.384	0.078
18	5.924	1.837	0.103
19	6.690	0.212	0.012
20	6.924	0.249	0.014
21	7.914	0.789	0.044
22	8.530	0.210	0.012

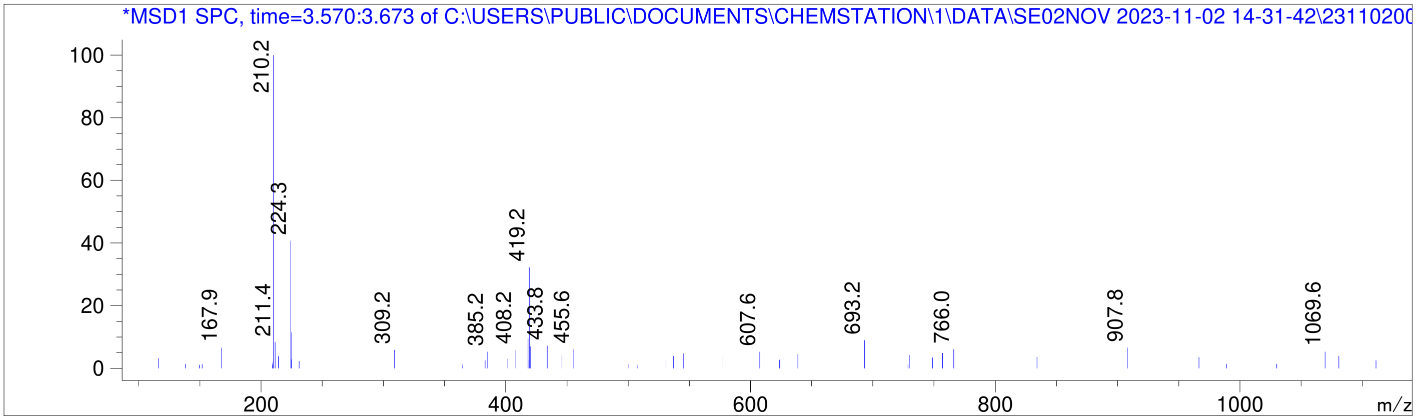
*MSD1 SPC, time=3.570:3.673 of C:\USERS\PUBLIC\DOCUMENTS\CHEMSTATION\1\DATA\SE02NOV 2023-11-02 14-31-42\2311020
210.2
224.3
419.2
167.9
211.4
309.2
385.2
408.2
433.8
455.6
607.6
693.2
766.0
907.8
1069.6
m/z

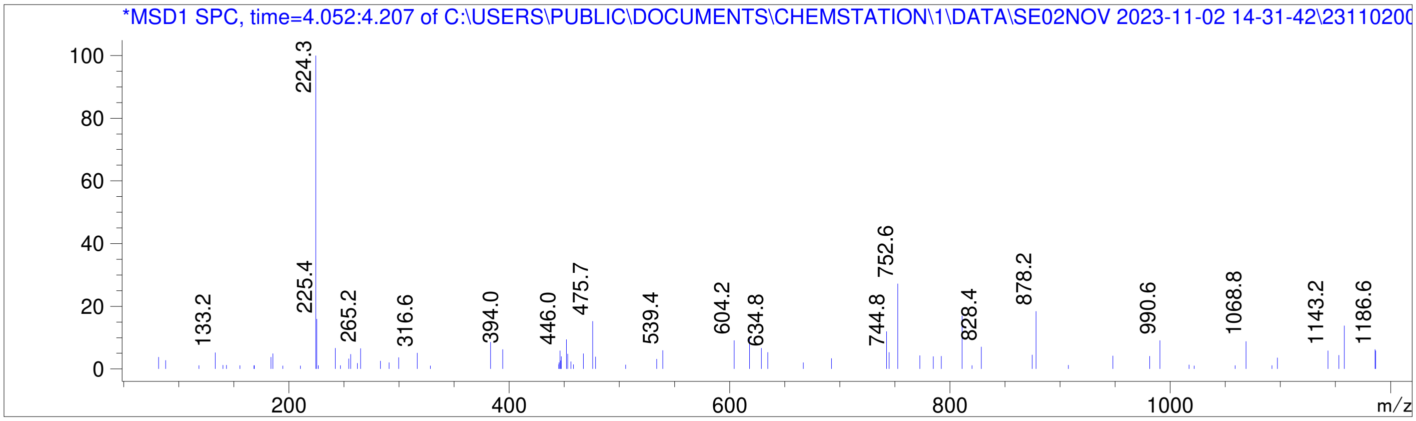
*MSD1 SPC, time=4.052:4.207 of C:\USERS\PUBLIC\DOCUMENTS\CHEMSTATION\1\DATA\SE02NOV 2023-11-02 14-31-42\2311020
224.3
752.6
225.4
133.2
265.2
316.6
394.0
446.0
475.7
539.4
604.2
634.8
744.8
828.4
878.2
990.6
1068.8
1143.2
1186.6
m/z

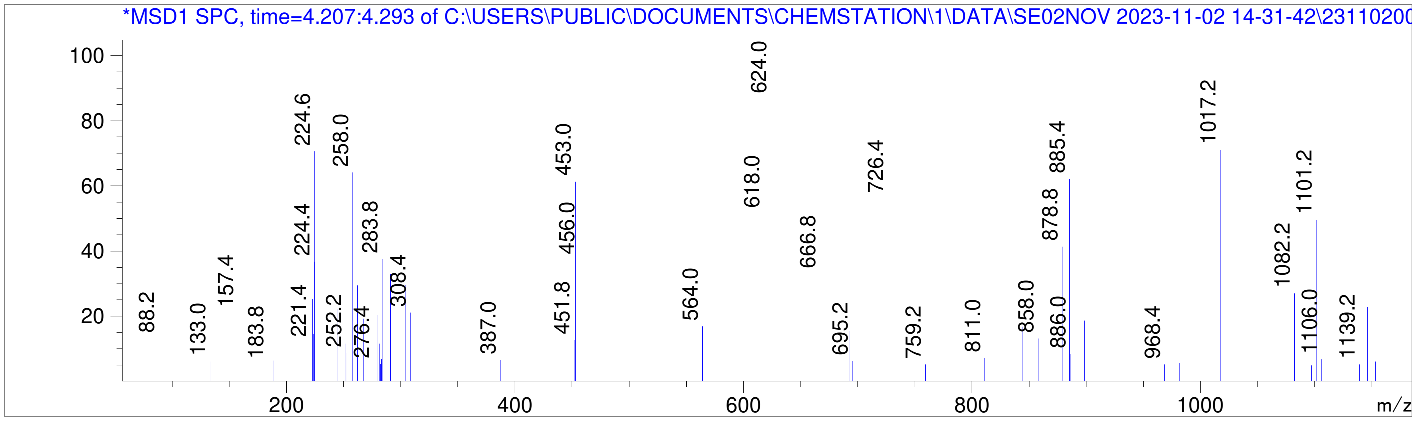
*MSD1 SPC, time=4.207:4.293 of C:\USERS\PUBLIC\DOCUMENTS\CHEMSTATION\1\DATA\SE02NOV 2023-11-02 14-31-42\2311020
624.0
1017.2
224.6
258.0
453.0
885.4
726.4
1101.2
618.0
878.8
224.4
283.8
456.0
666.8
1082.2
88.2
133.0
157.4
183.8
221.4
252.2
276.4
308.4
387.0
451.8
564.0
695.2
759.2
811.0
858.0
886.0
968.4
1106.0
1139.2
m/z

Sample-> CPT22010446-20-B2-50deg-24h

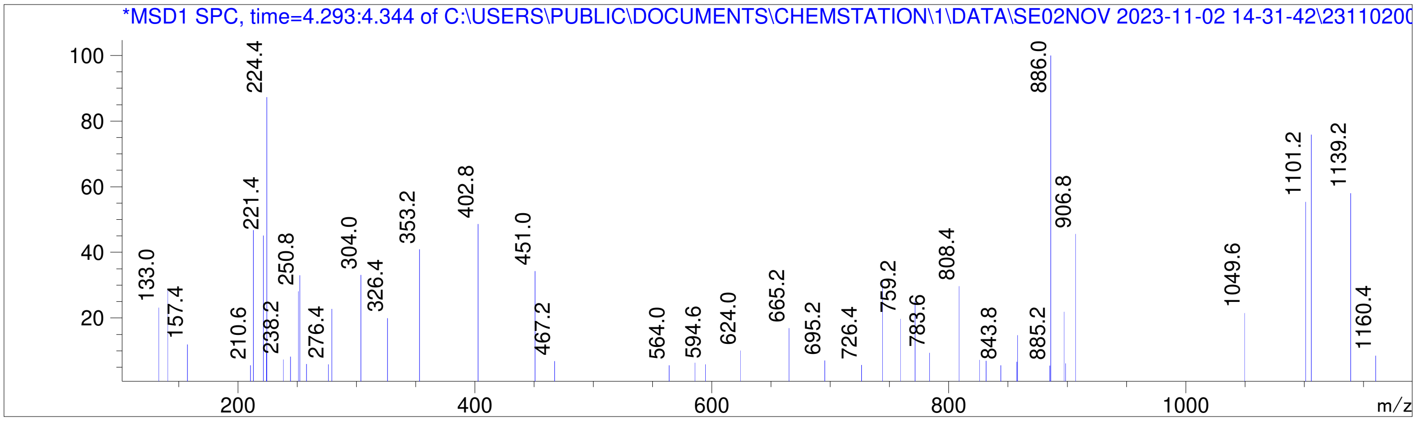

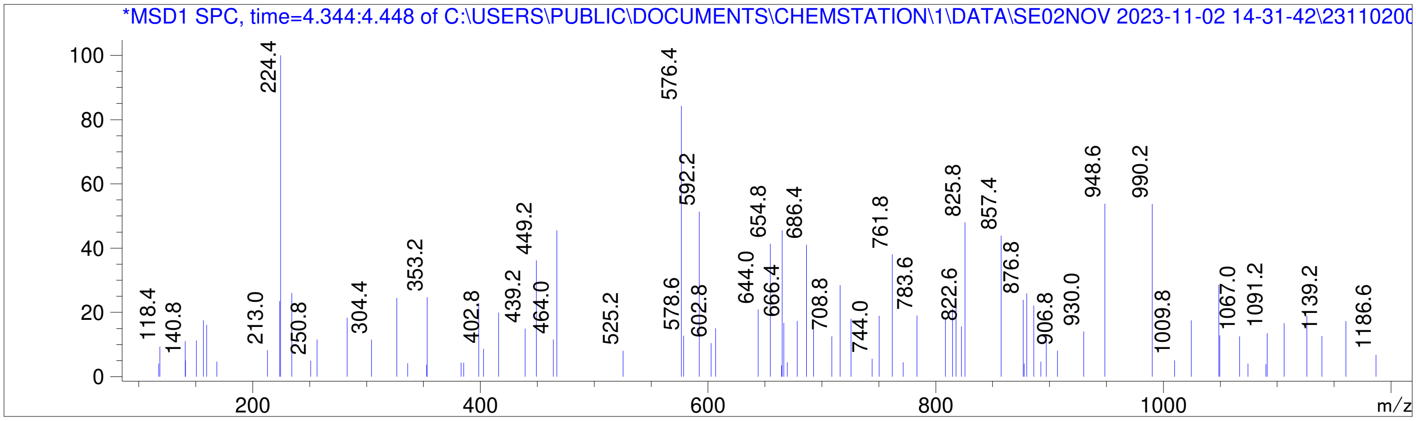

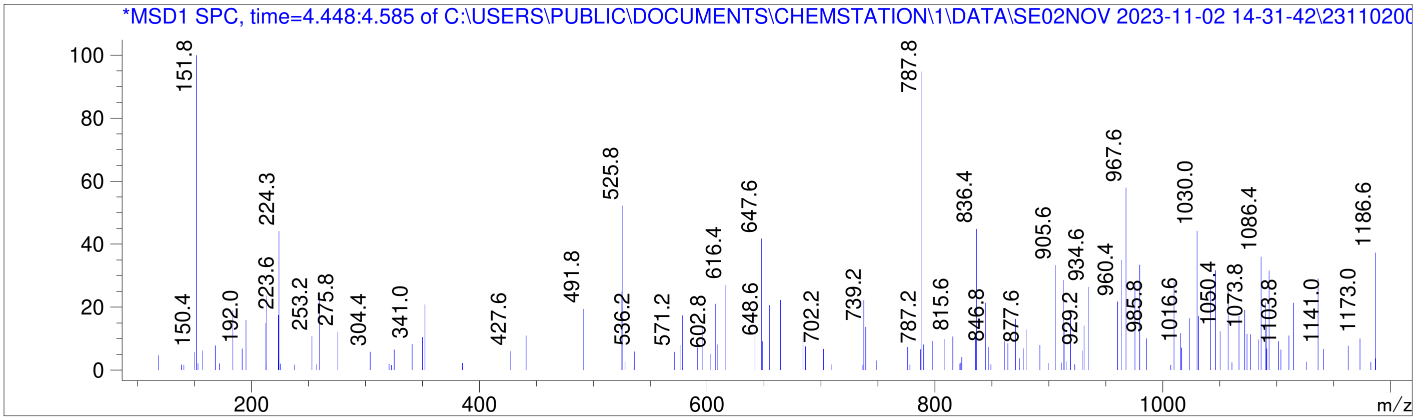

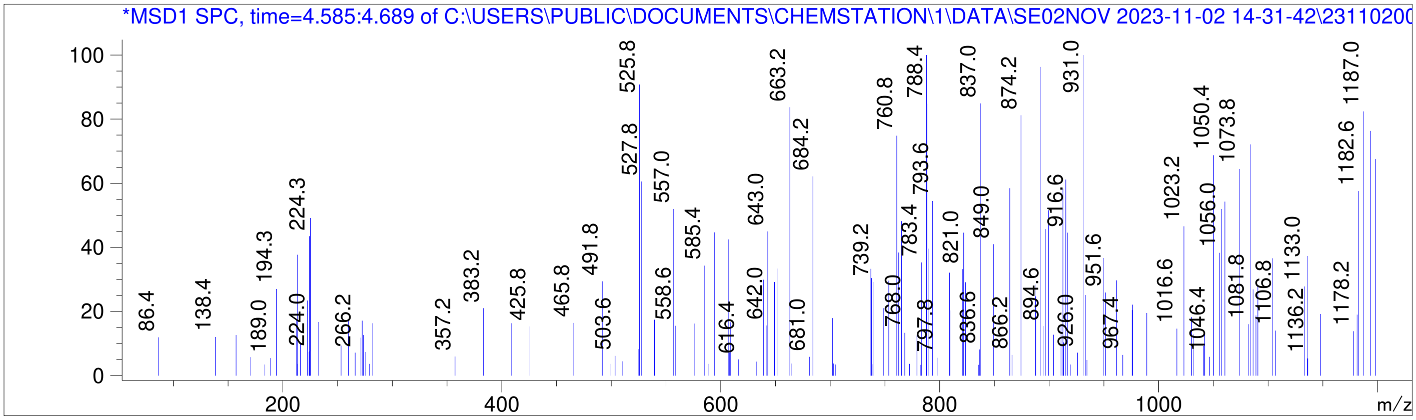

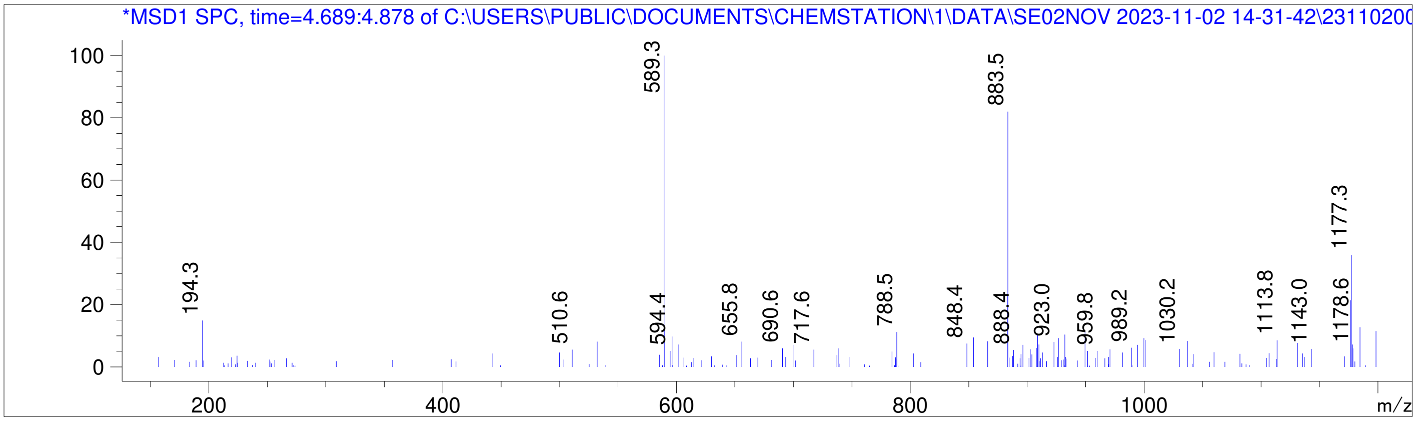
*MSD1 SPC, time=4.689:4.878 of C:\USERS\PUBLIC\DOCUMENTS\CHEMSTATION\1\DATA\SE02NOV 2023-11-02 14-31-42\2311020
194.3
510.6
589.3
594.4
655.8
690.6
717.6
788.5
848.4
883.5
888.4
923.0
959.8
989.2
1030.2
1113.8
1143.0
1177.3
1178.6
m/z

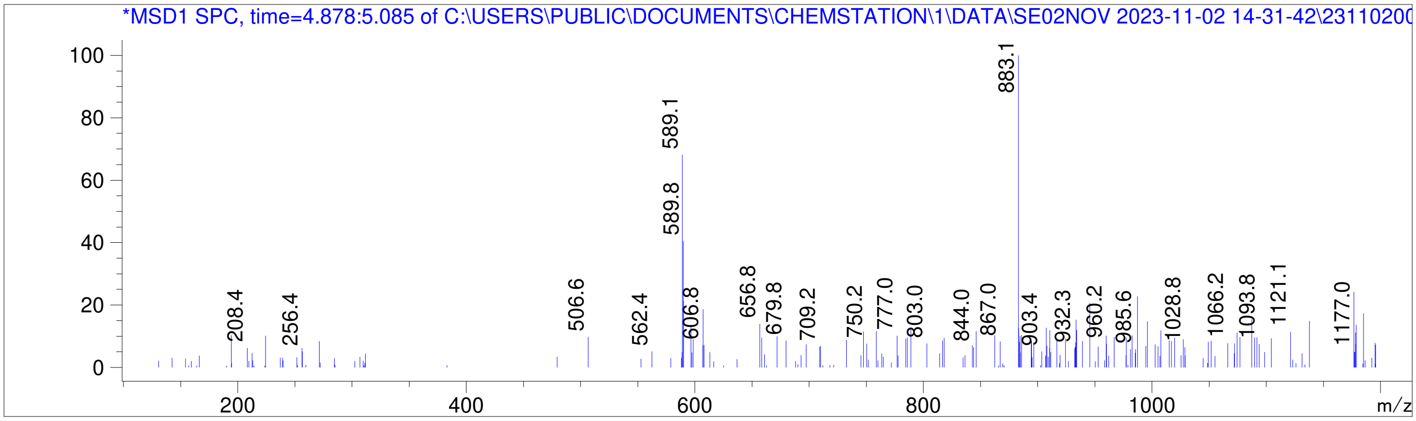
*MSD1 SPC, time=4.878:5.085 of C:\USERS\PUBLIC\DOCUMENTS\CHEMSTATION\1\DATA\SE02NOV 2023-11-02 14-31-42\2311020
208.4
256.4
506.6
562.4
589.1
589.8
606.8
656.8
679.8
709.2
750.2
777.0
803.0
844.0
867.0
883.1
903.4
932.3
960.2
985.6
1028.8
1066.2
1093.8
1121.1
1177.0
m/z

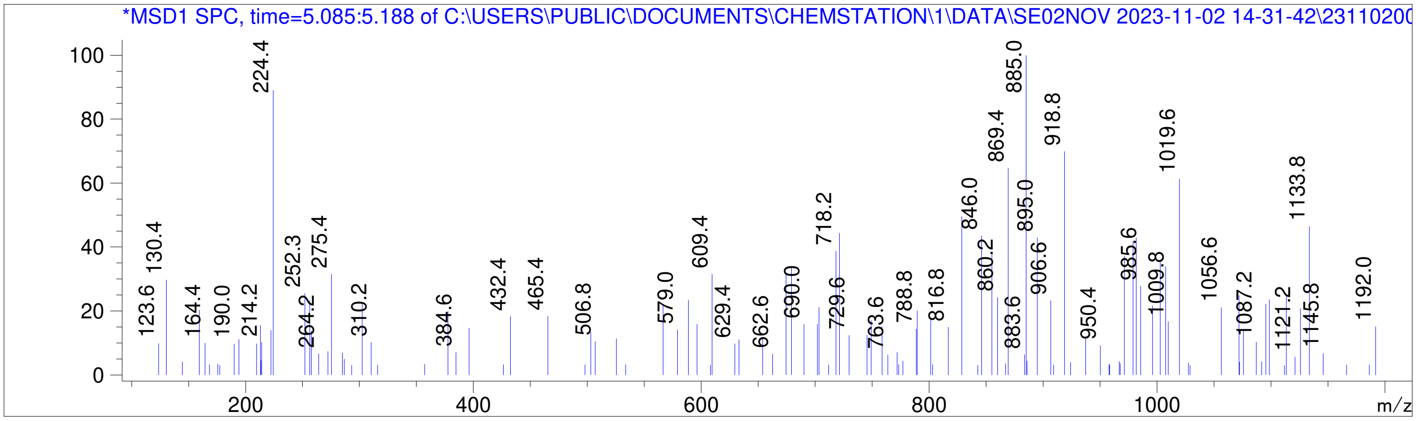
*MSD1 SPC, time=5.085:5.188 of C:\USERS\PUBLIC\DOCUMENTS\CHEMSTATION\1\DATA\SE02NOV 2023-11-02 14-31-42\2311020
123.6
130.4
164.4
190.0
214.2
224.4
252.3
264.2
275.4
310.2
384.6
432.4
465.4
506.8
579.0
609.4
629.4
662.6
690.0
718.2
729.6
763.6
788.8
816.8
846.0
860.2
869.4
883.6
885.0
895.0
906.6
918.8
950.4
985.6
1009.8
1019.6
1056.6
1087.2
1121.2
1133.8
1145.8
1192.0
m/z

Data -> C:\USERS\PUBLIC\DOCUMENTS\CHEMSTATION\1\DATA\SE02NOV 2023-11-02 14-31-42\   ->
Sample-> CPT22010446-20-B2-50deg-24h
==

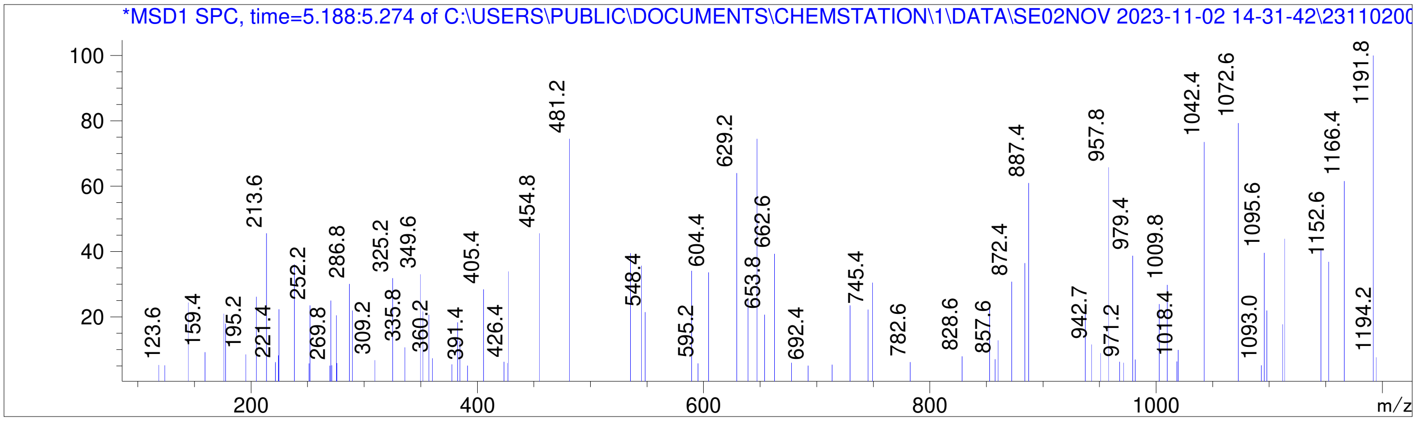

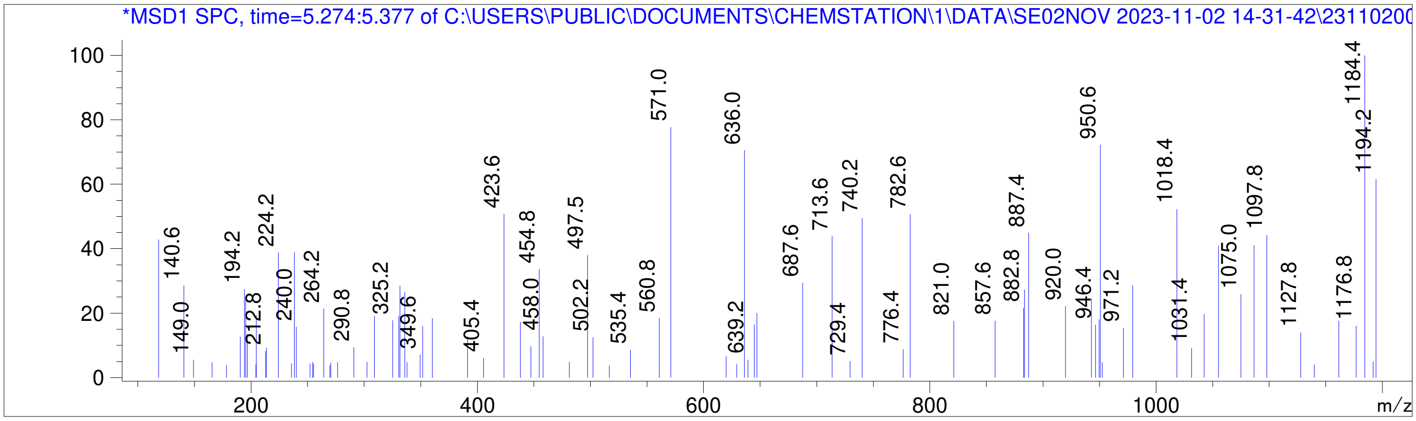

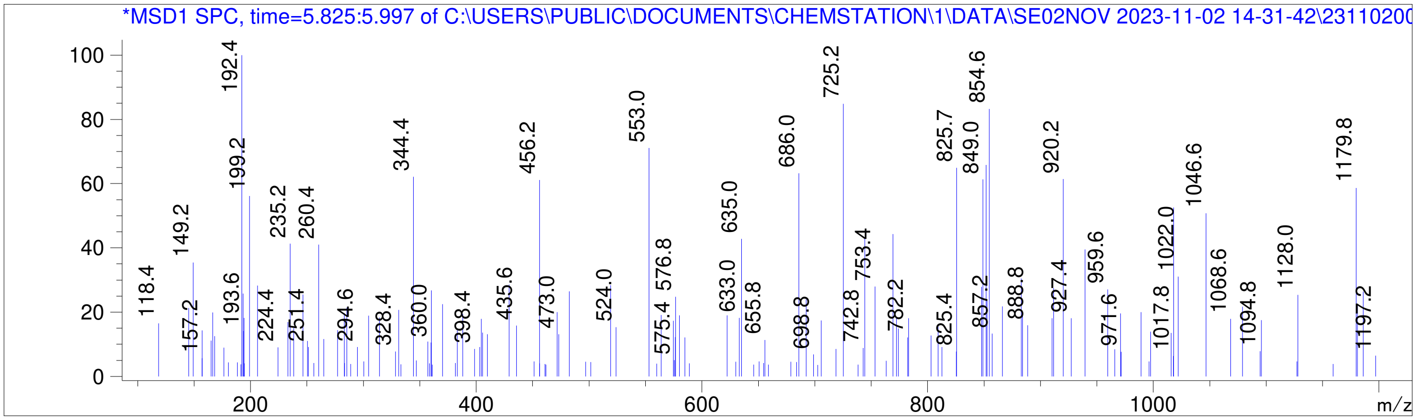

Supplement: Supplementary file 2 — Data S1 and S2 [file sciadv.adr0006_data_s1_and_s2.zip › Supplementary Dataset 1-LCMS DATA/LCMS PNA Hexamers A-T/LCMS G6 50C_80C/50C/24h/CPT22010446-20-B2-50deg-24h.pdf]
