# Supplementary material for: Astrobiological implications of the stability and reactivity of peptide nucleic acid (PNA) in concentrated sulfuric acid
Source: Sci Adv. 2025 Mar 26;11(13):eadr0006. doi: 10.1126/sciadv.adr0006 (PMC11939054; doi:10.1126/sciadv.adr0006)
Supplement: Supplementary file 2 — Data S1 and S2 [file sciadv.adr0006_data_s1_and_s2.zip › Supplementary Dataset 1-LCMS DATA/LCMS PNA Hexamers A-T/LCMS G6 50C_80C/80C/1h/CPT22010446-13-B2-80deg-1h.pdf]

Data -> C:\USERS\PUBLIC\DOCUMENTS\CHEMSTATION\1\DATA\SE07NOV 2023-11-07 08-21-00\ ->
Sample-> CPT22010446-13-B2-80deg-1h

==

Injection Date	:	Tue, 7. Nov. 2023	Seq Line	:	7
			Location	:	47
			Inj. Vol.	:	2 µl

Acq. Method : C:\Users\Public\Documents\ChemStation\1\Data\SE07NOV 2023-11-07 08-21-00\22010446 LCMS-6.M
Analysis Method : C:\Users\Public\Documents\ChemStation\1\Data\SE07NOV 2023-11-07 08-21-00\22010446 LCMS-6.M (Sequence Method)
Waters XBridge Phenyl (4.6 * 150 mm; 3.5 µm); 0.05% TFA (aq) / AcN: 100/0 (0.0 min) --> (6.0 min) --> 70/30 (0.0 min) --> (2.0 min) --> 10/90 (2.0 min); Flow: 1.0 ml/min;
MSD1 = positive; MSD2 = negative

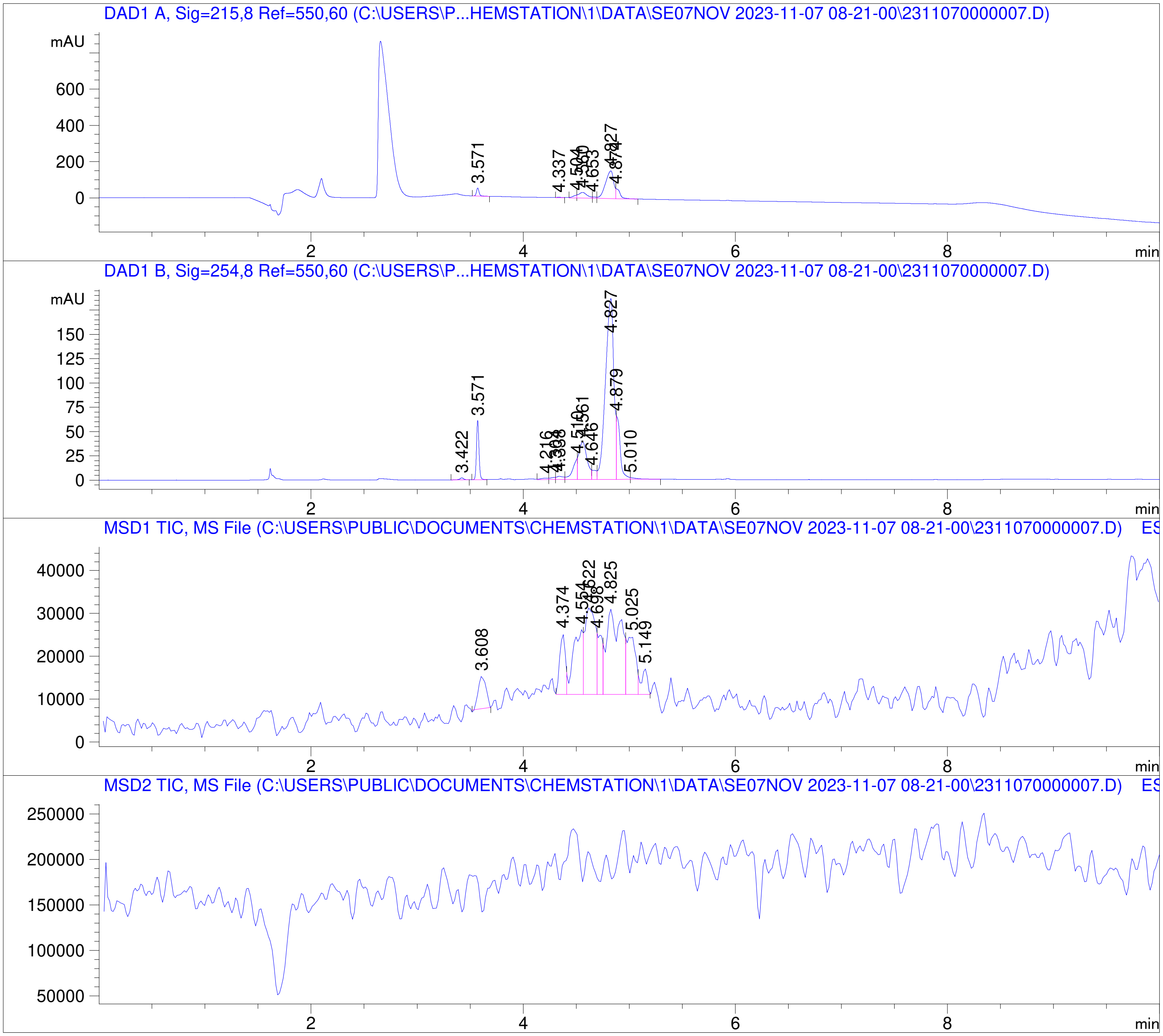

DAD1 A, Sig=215,8 Ref=550,60

Peak #	Ret. Time [min]	Area [mV *s]	Area %
1	3.571	76.050	5.895
2	4.337	9.989	0.774
3	4.504	37.894	2.937
4	4.560	174.780	13.548
5	4.653	16.969	1.315
6	4.827	824.069	63.878
7	4.874	150.322	11.652

DAD1 B, Sig=254,8 Ref=550,60

Peak #	Ret. Time [min]	Area [mV *s]	Area %
1	3.422	5.284	0.324
2	3.571	100.166	6.148
3	4.216	7.517	0.461
4	4.304	7.094	0.435
5	4.338	15.143	0.929
6	4.510	64.558	3.962
7	4.561	207.929	12.762
8	4.646	28.303	1.737
9	4.827	1023.823	62.839
10	4.879	160.842	9.872
11	5.010	8.624	0.529

==

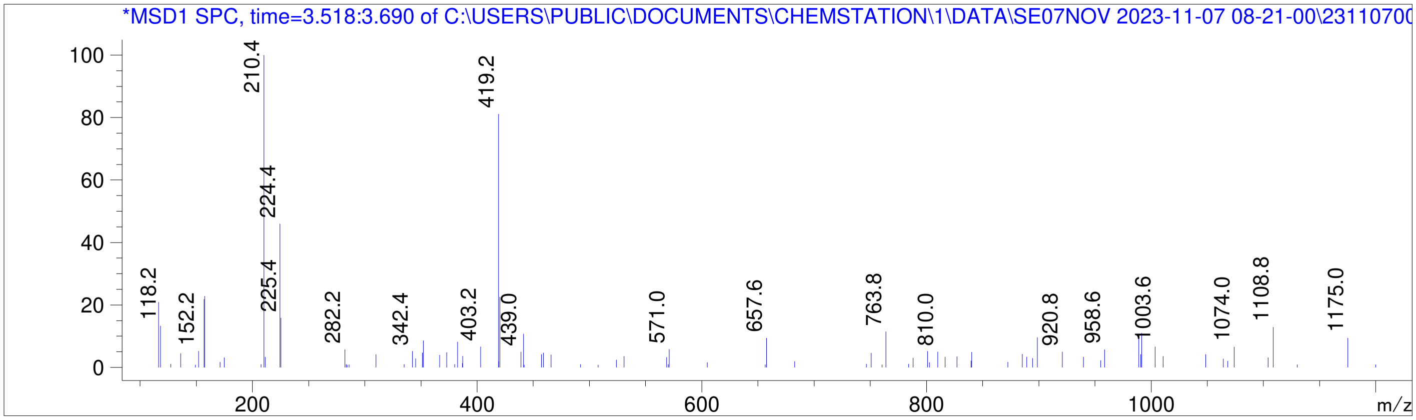
*MSD1 SPC, time=3.518:3.690 of C:\USERS\PUBLIC\DOCUMENTS\CHEMSTATION\1\DATA\SE07NOV 2023-11-07 08-21-00\2311070
210.4
419.2
224.4
225.4
118.2
152.2
282.2
342.4
403.2
439.0
571.0
657.6
763.8
810.0
920.8
958.6
1003.6
1074.0
1108.8
1175.0
m/z

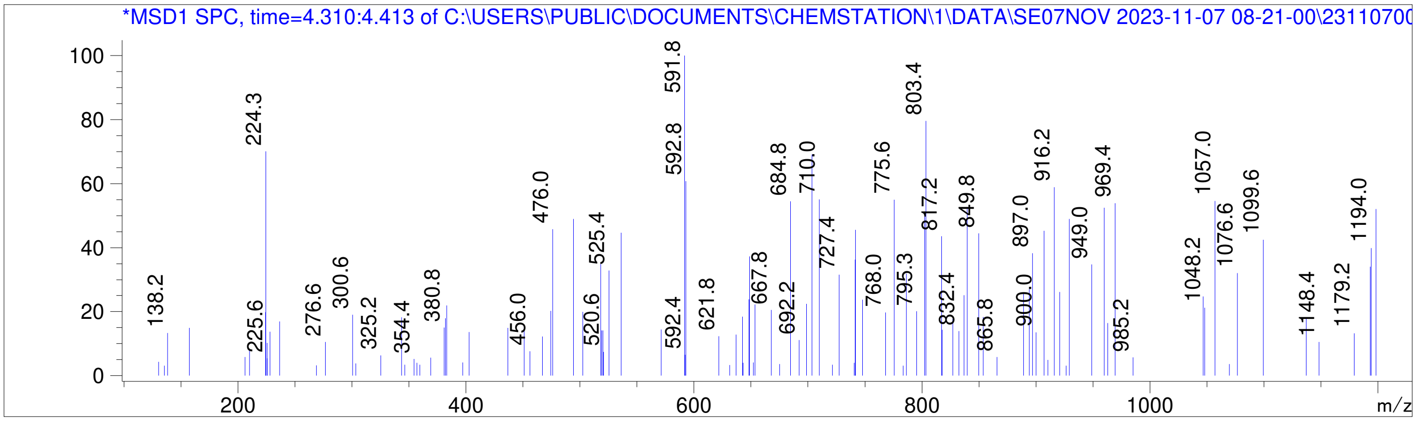
*MSD1 SPC, time=4.310:4.413 of C:\USERS\PUBLIC\DOCUMENTS\CHEMSTATION\1\DATA\SE07NOV 2023-11-07 08-21-00\2311070
591.8
224.3
592.8
803.4
684.8
710.0
775.6
476.0
525.4
817.2
849.8
897.0
916.2
969.4
1057.0
1076.6
1099.6
1194.0
727.4
949.0
1048.2
1179.2
1148.4
138.2
225.6
276.6
300.6
325.2
354.4
380.8
456.0
520.6
592.4
621.8
667.8
692.2
768.0
795.3
832.4
865.8
900.0
985.2
m/z

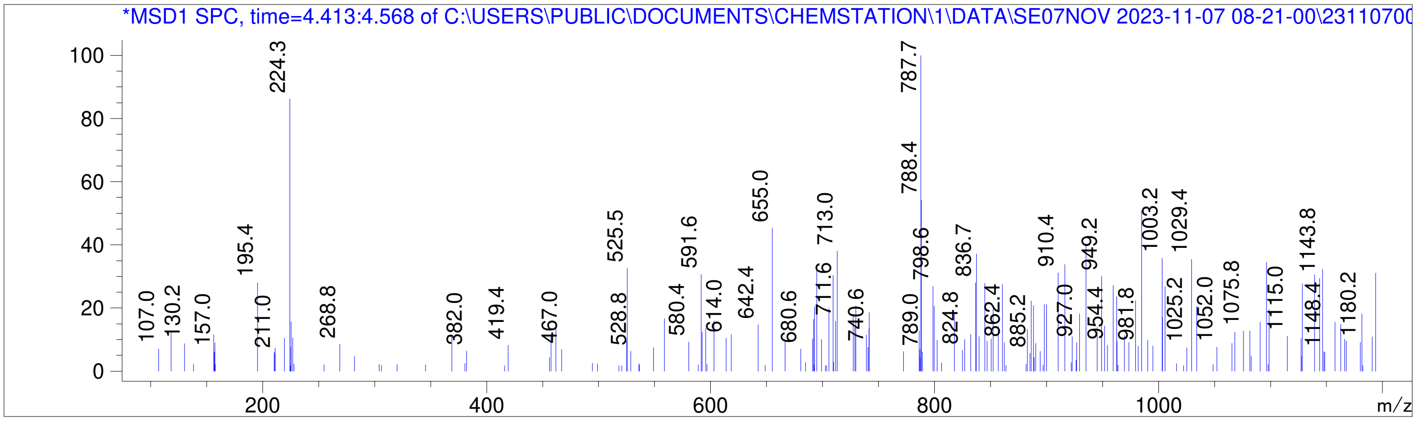
*MSD1 SPC, time=4.413:4.568 of C:\USERS\PUBLIC\DOCUMENTS\CHEMSTATION\1\DATA\SE07NOV 2023-11-07 08-21-00\2311070
787.7
224.3
788.4
655.0
713.0
525.5
591.6
195.4
642.4
711.6
798.6
836.7
910.4
949.2
1003.2
1029.4
1075.8
1143.8
107.0
130.2
157.0
211.0
268.8
382.0
419.4
467.0
528.8
580.4
614.0
680.6
740.6
789.0
824.8
862.4
885.2
927.0
954.4
981.8
1025.2
1052.0
1115.0
1148.4
1180.2
m/z

Sample-> CPT22010446-13-B2-80deg-1h

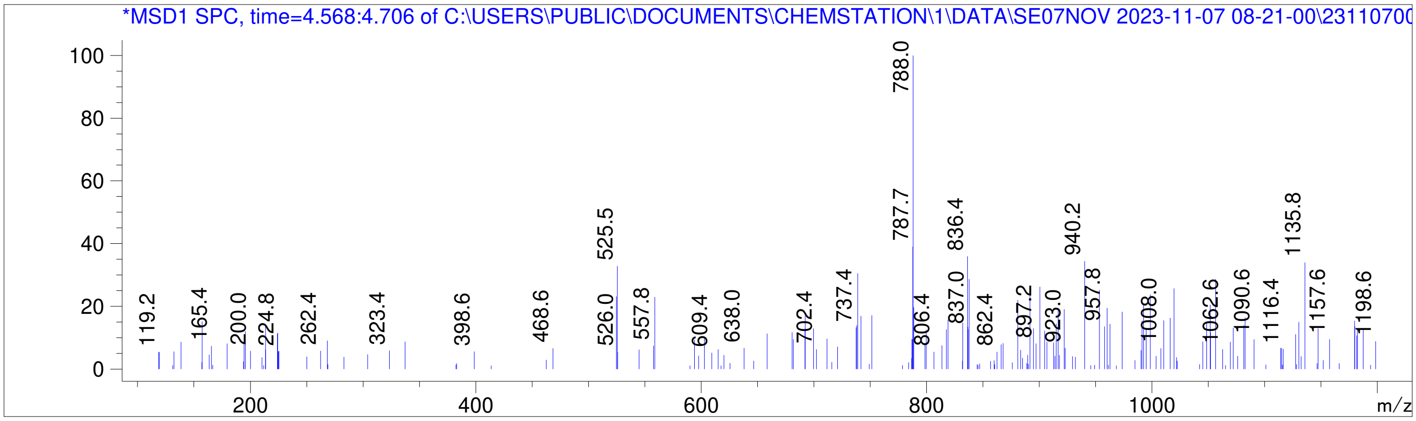

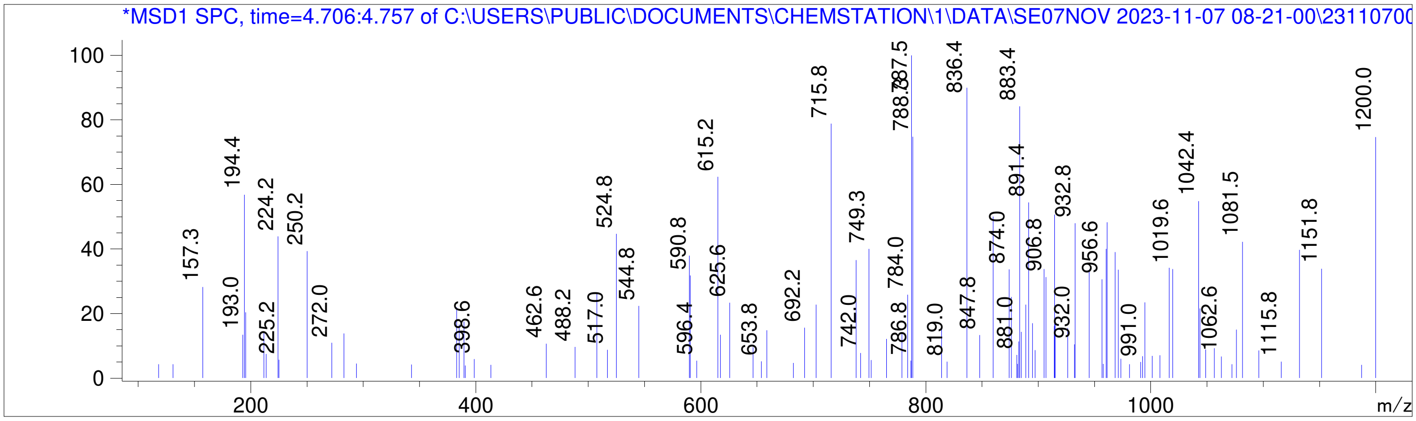

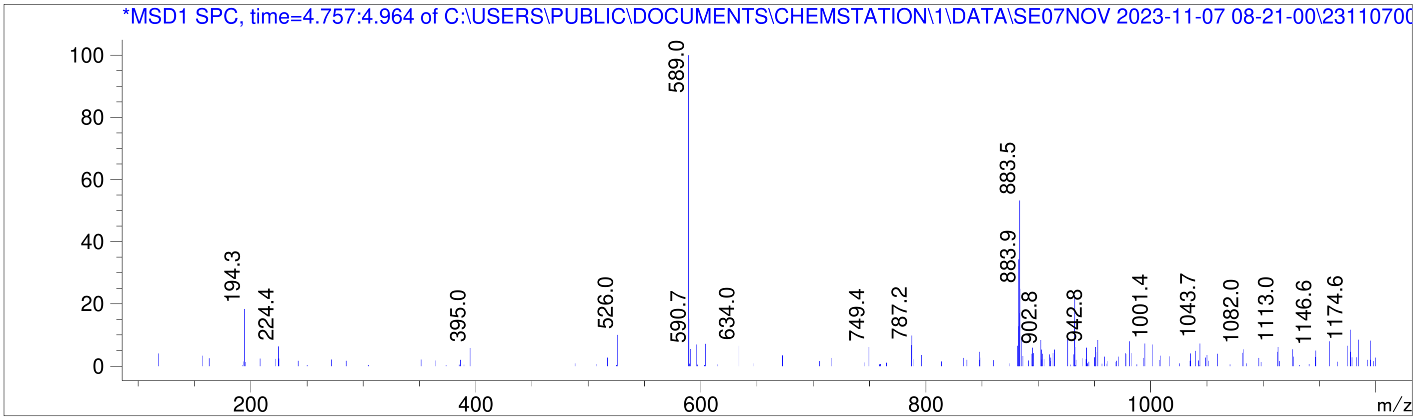

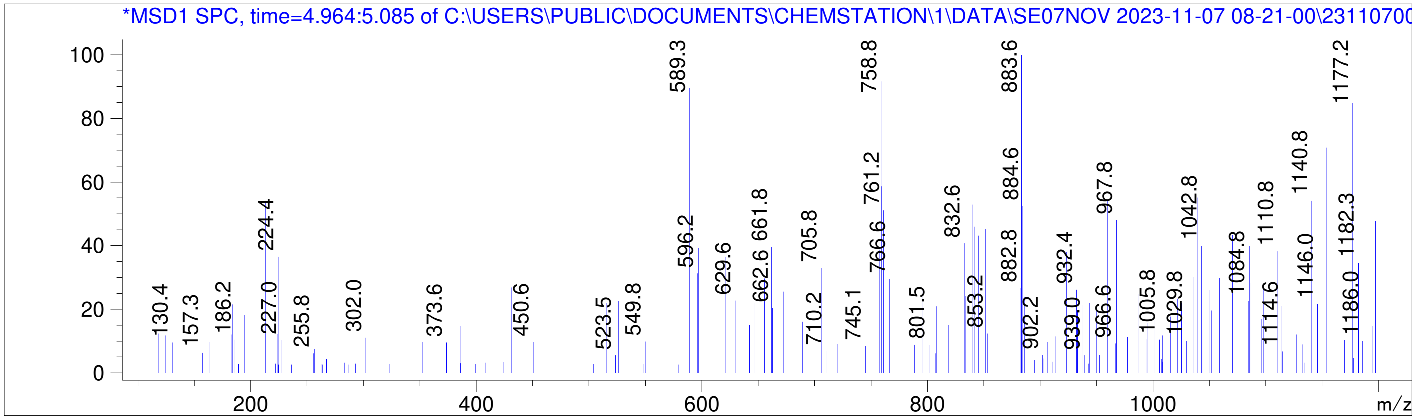

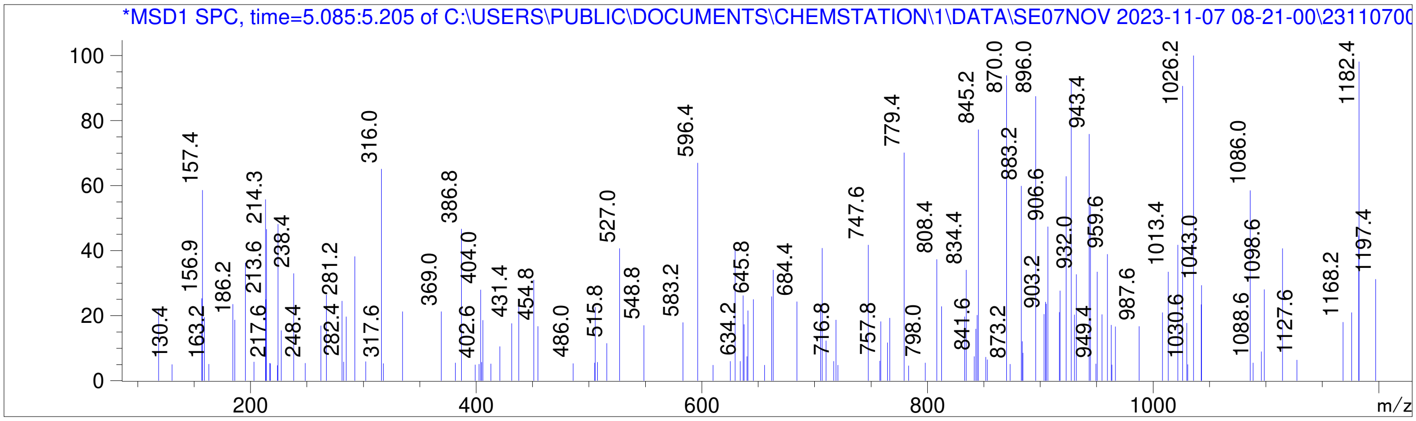
*MSD1 SPC, time=5.085:5.205 of C:\USERS\PUBLIC\DOCUMENTS\CHEMSTATION\1\DATA\SE07NOV 2023-11-07 08-21-00\23110700
m/z
