# Supplementary material for: Astrobiological implications of the stability and reactivity of peptide nucleic acid (PNA) in concentrated sulfuric acid
Source: Sci Adv. 2025 Mar 26;11(13):eadr0006. doi: 10.1126/sciadv.adr0006 (PMC11939054; doi:10.1126/sciadv.adr0006)

Injection Date : Fri, 20. Oct. 2023		Seq Line :	37
		Location :	12
		Inj. Vol. :	2 µl

Acq. Method : C:\Users\Public\Documents\ChemStation\1\Data\SE19OCT 2023-10-19 16-56-54\22010446 LCMS-6.M
Analysis Method : C:\Users\Public\Documents\ChemStation\1\Data\SE19OCT 2023-10-19 16-56-54\22010446 LCMS-6.M (Sequence Method)
Waters XBridge Phenyl (4.6 * 150 mm; 3.5 µm); 0.05% TFA (aq) / AcN: 100/0 (0.0 min) --> (6.0 min) --> 70/30 (0.0 min) --> (2.0 min) --> 10/90 (2.0 min); Flow: 1.0 ml/min;
MSD1 = positive; MSD2 = negative

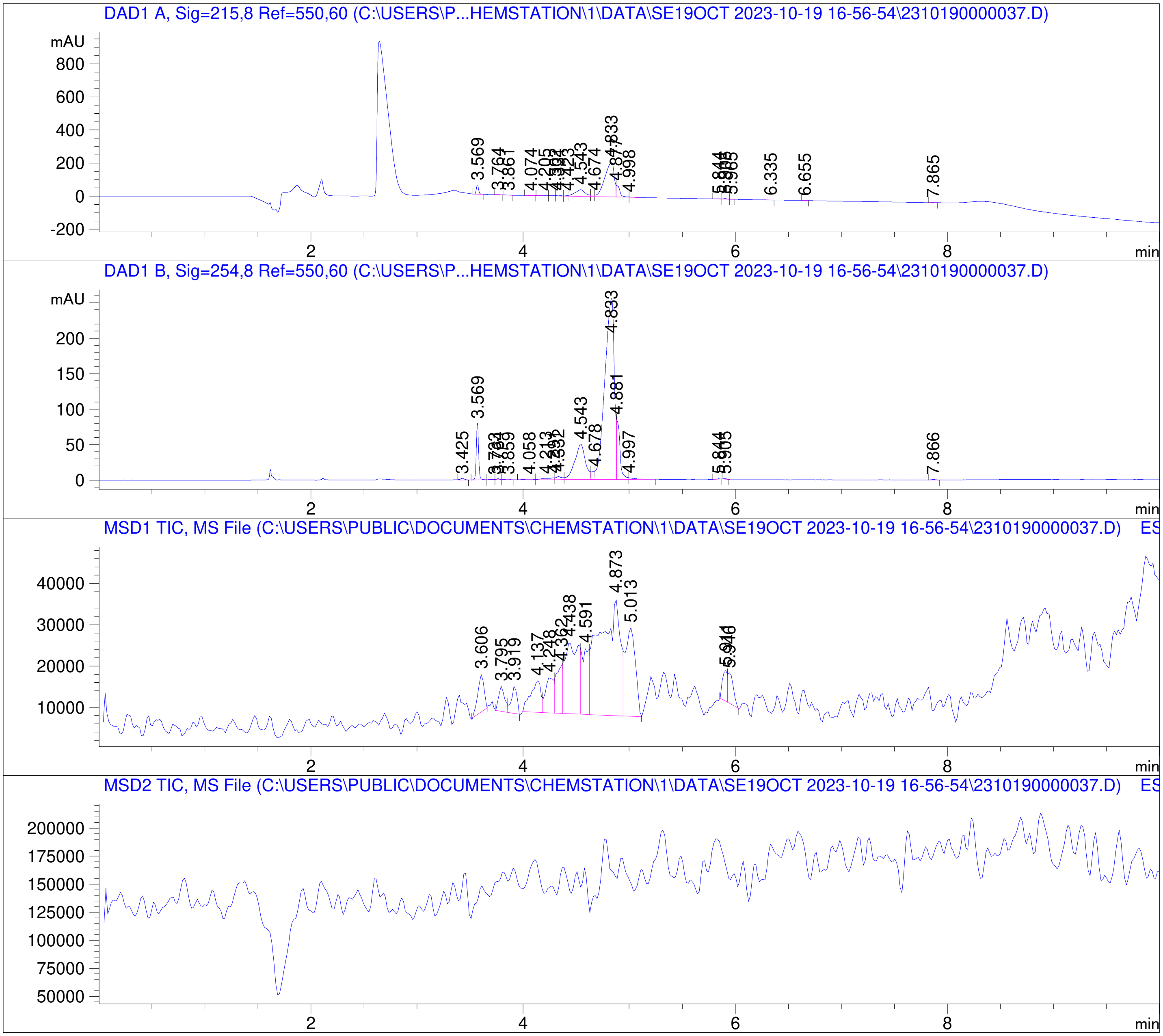

DAD1 A, Sig=215,8 Ref=550,60

Peak #	Ret. Time [min]	Area [mV *s]	Area %
1	3.569	80.520	4.282
2	3.764	2.739	0.146
3	3.861	1.970	0.105
4	4.074	2.349	0.125
5	4.205	3.333	0.177
6	4.302	3.614	0.192
7	4.331	12.250	0.651
8	4.423	6.955	0.370
9	4.543	270.729	14.397
10	4.674	19.830	1.054
11	4.833	1287.449	68.463
12	4.877	167.913	8.929
13	4.998	4.399	0.234
14	5.844	3.823	0.203
15	5.905	8.150	0.433
16	5.965	0.645	0.034
17	6.335	0.559	0.030
18	6.655	0.639	0.034
19	7.865	2.629	0.140

DAD1 B, Sig=254,8 Ref=550,60

Peak #	Ret. Time [min]	Area [mV *s]	Area %
1	3.425	5.521	0.237
2	3.569	117.667	5.060
3	3.733	1.972	0.085
4	3.764	3.299	0.142
5	3.859	3.285	0.141
6	4.058	4.662	0.200
7	4.213	8.068	0.347
8	4.291	7.190	0.309
9	4.332	18.314	0.788
10	4.543	348.050	14.967
11	4.678	26.583	1.143
12	4.833	1581.996	68.030
13	4.881	184.280	7.925
14	4.997	9.337	0.402
15	5.844	1.440	0.062
16	5.905	2.347	0.101
17	7.866	1.433	0.062

Data -> C:\USERS\PUBLIC\DOCUMENTS\CHEMSTATION\1\DATA\SE19OCT 2023-10-19 16-56-54\   ->
Sample-> CPT22010446-20-B1-80dg-1h
==

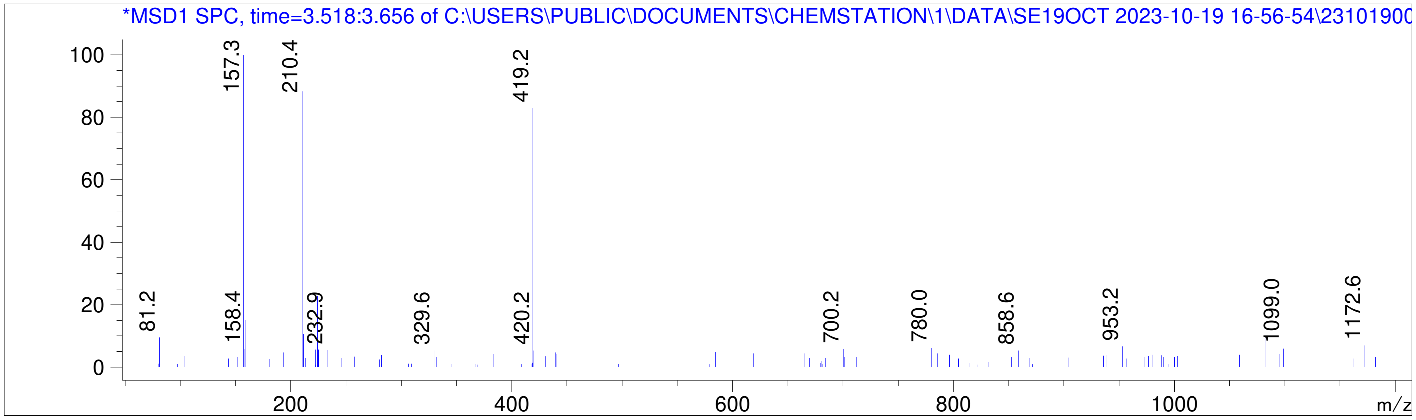

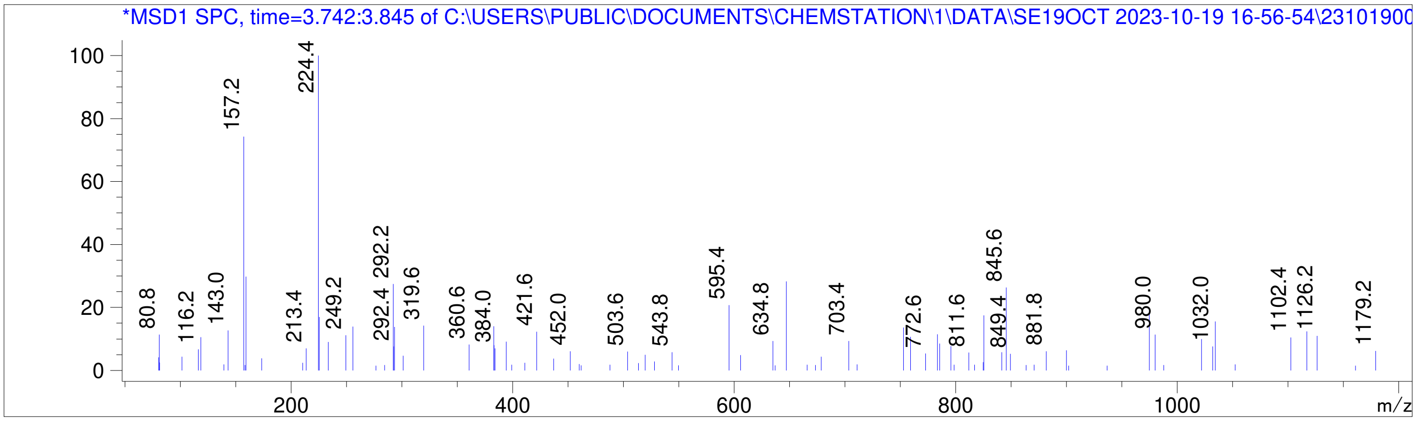

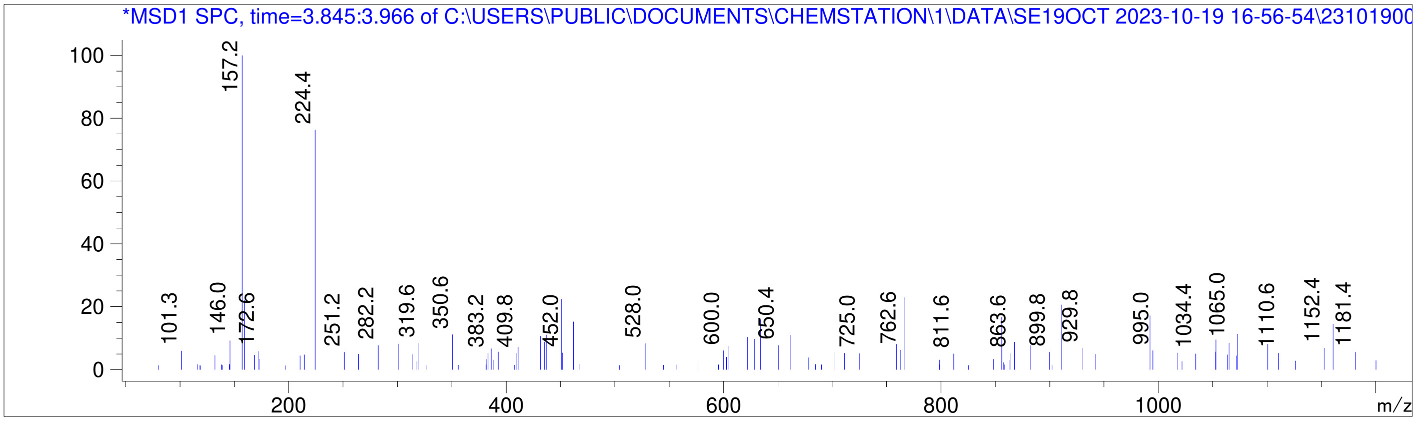

Data -> C:\USERS\PUBLIC\DOCUMENTS\CHEMSTATION\1\DATA\SE19OCT 2023-10-19 16-56-54\ ->
Sample-> CPT22010446-20-B1-80dg-1h

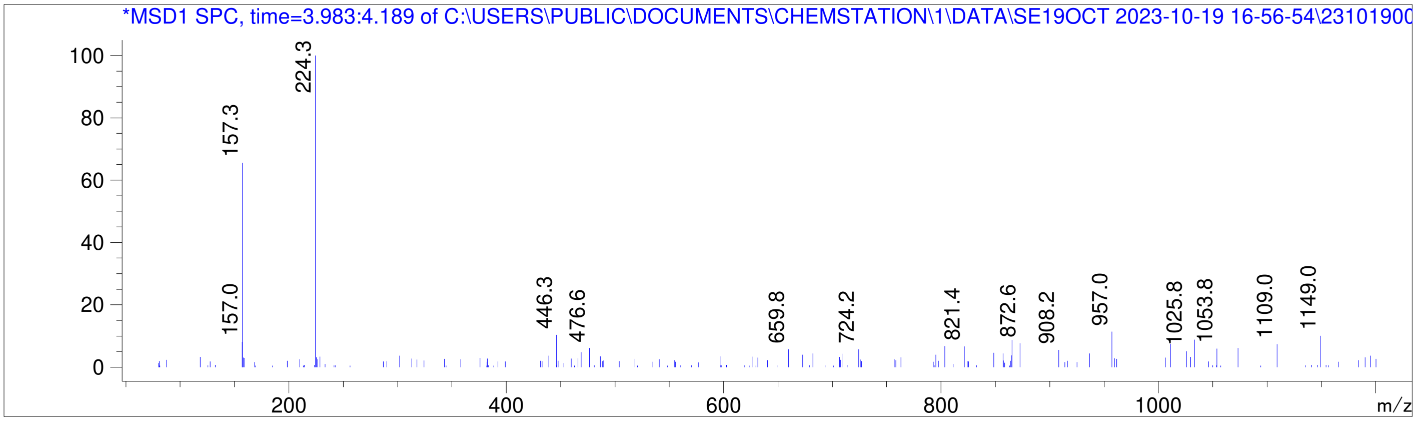

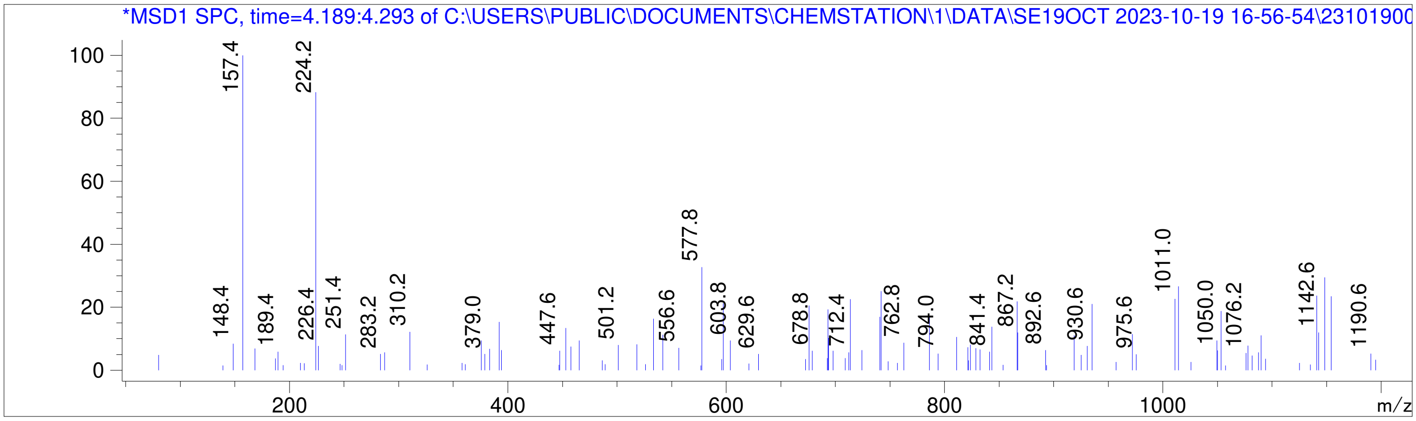

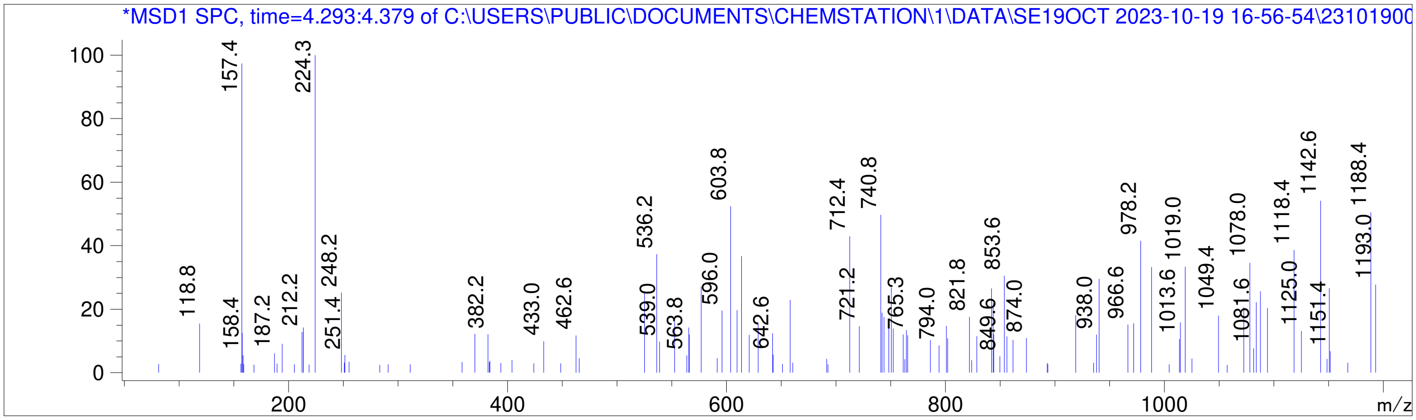

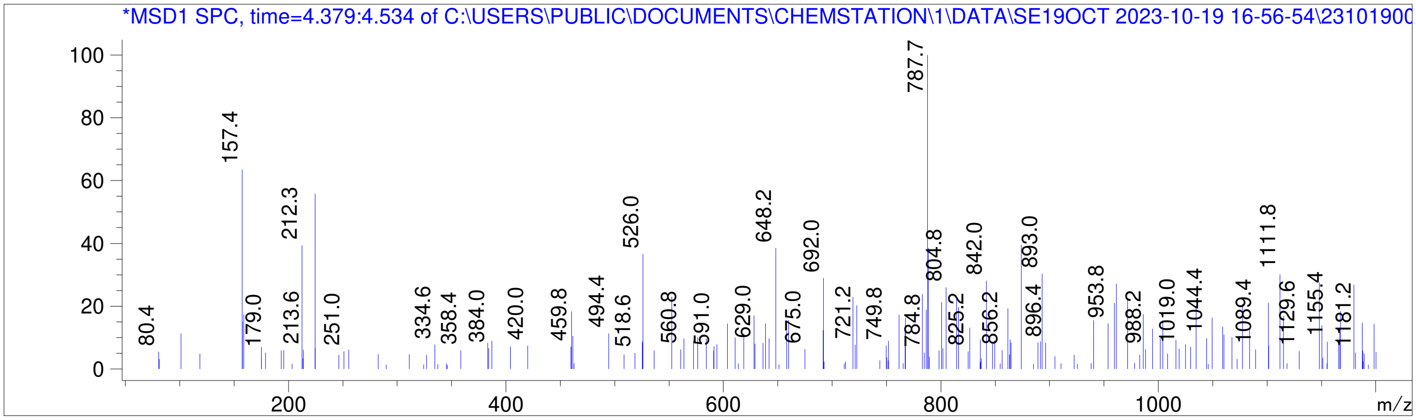

Data -> C:\USERS\PUBLIC\DOCUMENTS\CHEMSTATION\1\DATA\SE19OCT 2023-10-19 16-56-54\   ->
Sample-> CPT22010446-20-B1-80dg-1h
==

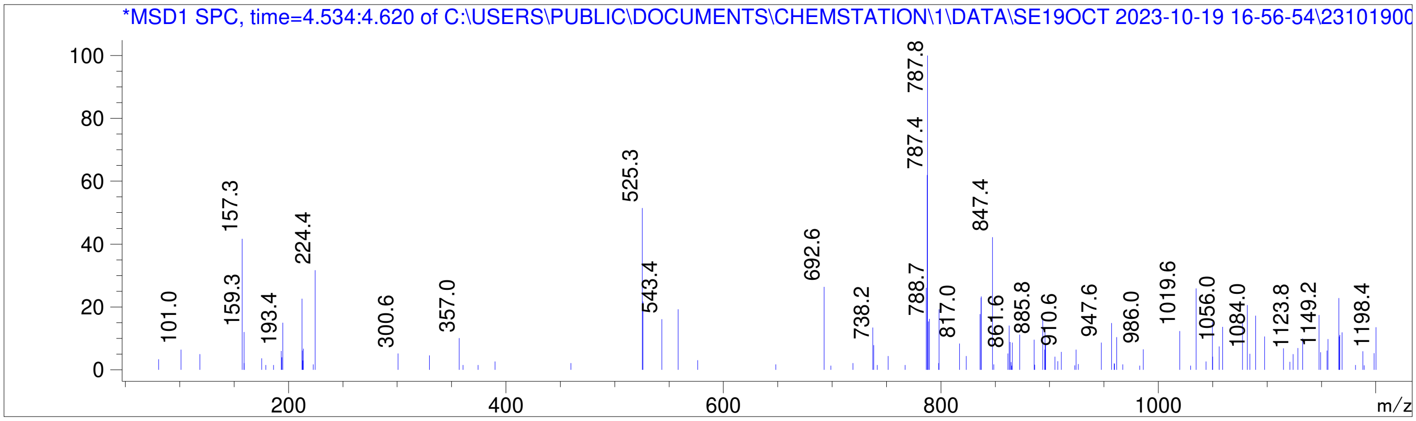

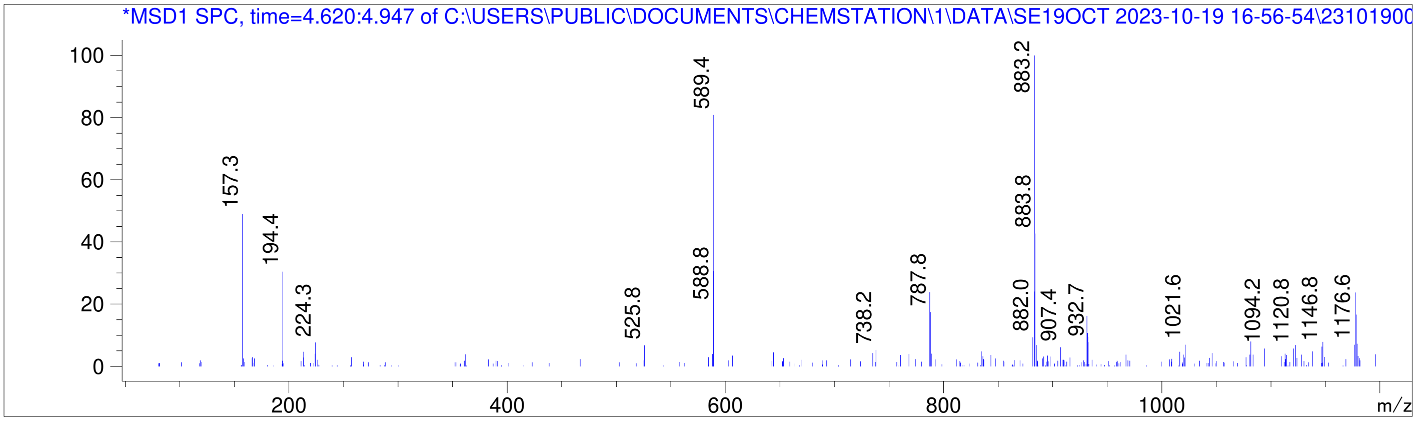

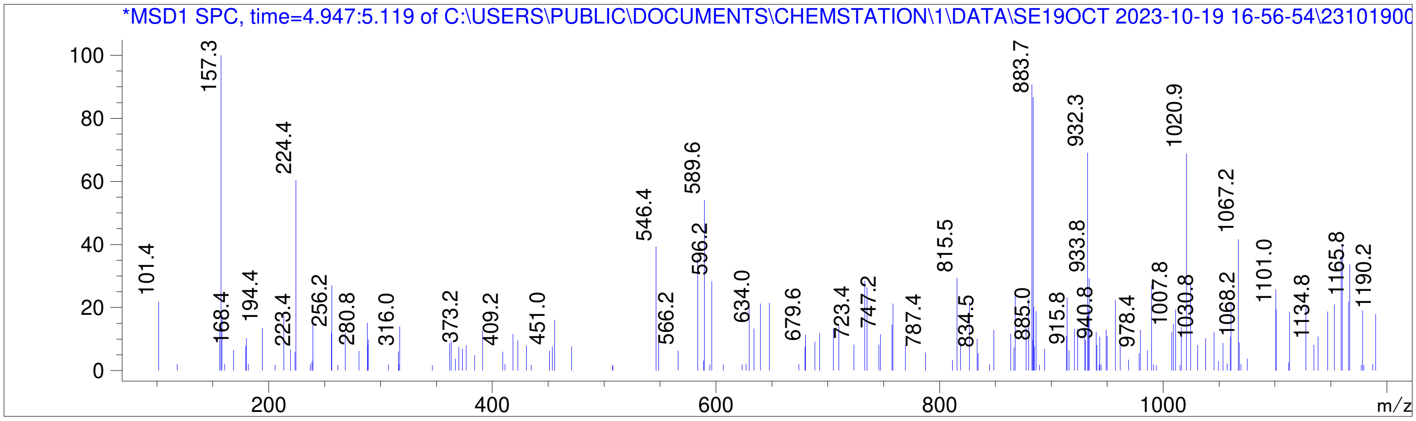

*MSD1 SPC, time=5.859:5.928 of C:\USERS\PUBLIC\DOCUMENTS\CHEMSTATION\1\DATA\SE19OCT 2023-10-19 16-56-54\23101900

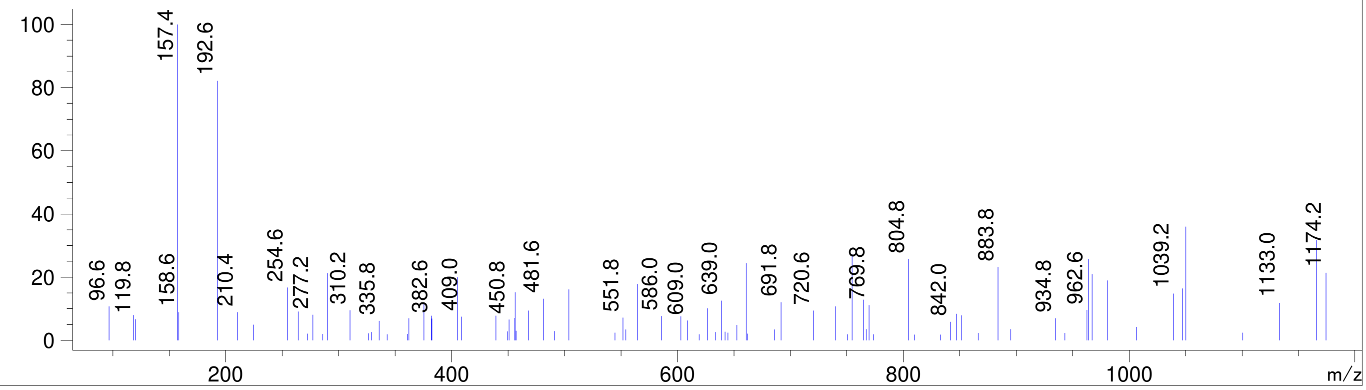

*MSD1 SPC, time=5.928:6.032 of C:\USERS\PUBLIC\DOCUMENTS\CHEMSTATION\1\DATA\SE19OCT 2023-10-19 16-56-54\23101900

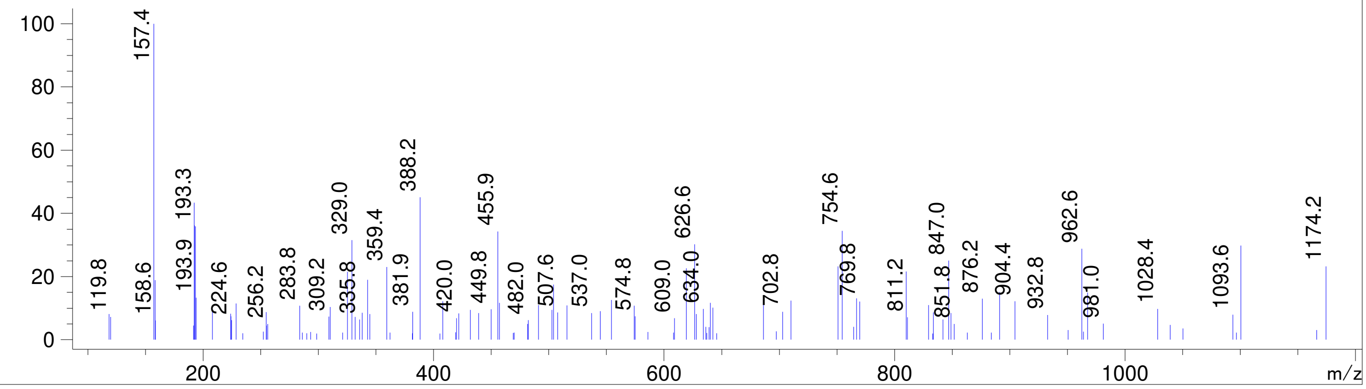

Supplement: Supplementary file 2 — Data S1 and S2 [file sciadv.adr0006_data_s1_and_s2.zip › Supplementary Dataset 1-LCMS DATA/LCMS PNA Hexamers A-T/LCMS G6 50C_80C/80C/1h/CPT22010446-20-B1-80dg-1h.pdf]
