# Supplementary material for: Astrobiological implications of the stability and reactivity of peptide nucleic acid (PNA) in concentrated sulfuric acid
Source: Sci Adv. 2025 Mar 26;11(13):eadr0006. doi: 10.1126/sciadv.adr0006 (PMC11939054; doi:10.1126/sciadv.adr0006)
Supplement: Supplementary file 2 — Data S1 and S2 [file sciadv.adr0006_data_s1_and_s2.zip › Supplementary Dataset 1-LCMS DATA/LCMS PNA Hexamers A-T/LCMS G6 50C_80C/80C/24h/CPT22010446-20-B1-80dg-24h.pdf]

Data -> C:\USERS\PUBLIC\DOCUMENTS\CHEMSTATION\1\DATA\SE19OCT 2023-10-19 16-56-54\ ->
Sample-> CPT22010446-20-B1-80dg-24h

===

Injection Date :	Fri, 20. Oct. 2023	Seq Line :	43
		Location :	23
		Inj. Vol. :	2 µl

Acq. Method : C:\Users\Public\Documents\ChemStation\1\Data\SE19OCT 2023-10-19 16-56-54\22010446 LCMS-6.M
Analysis Method : C:\Users\Public\Documents\ChemStation\1\Data\SE19OCT 2023-10-19 16-56-54\22010446 LCMS-6.M (Sequence Method)
Waters XBridge Phenyl (4.6 * 150 mm; 3.5 µm); 0.05% TFA (aq) / AcN: 100/0 (0.0 min) --> (6.0 min) --> 70/30 (0.0 min) --> (2.0 min) --> 10/90 (2.0 min); Flow: 1.0 ml/min;
MSD1 = positive; MSD2 = negative

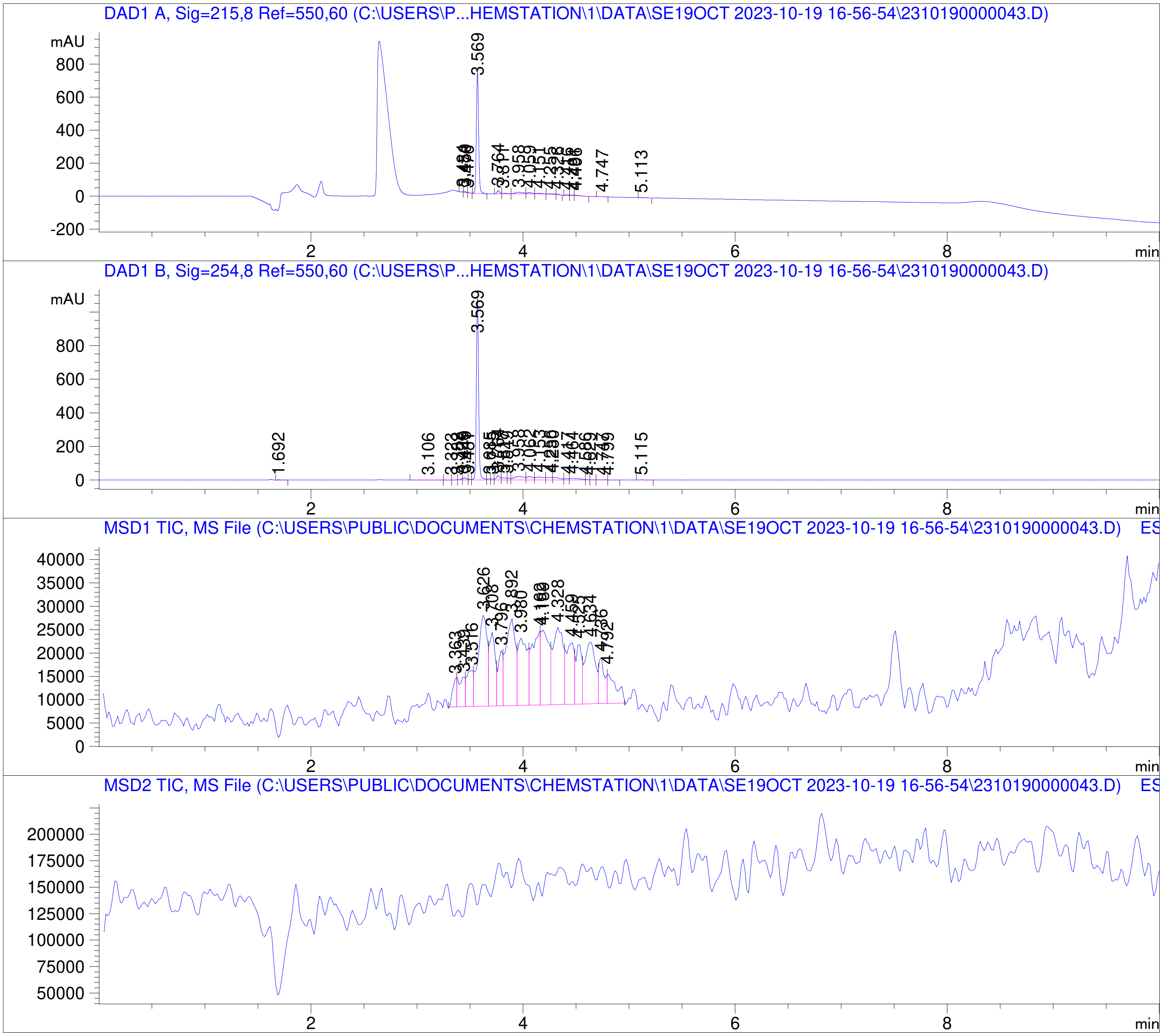

DAD1 A, Sig=215,8 Ref=550,60

Peak #	Ret. Time [min]	Area [mV *s]	Area %
1	3.434	2.841	0.222
2	3.450	8.460	0.662
3	3.476	2.154	0.169
4	3.569	1069.028	83.659
5	3.764	34.115	2.670
6	3.811	12.315	0.964
7	3.958	47.512	3.718
8	4.059	24.389	1.909
9	4.151	15.052	1.178
10	4.255	15.621	1.222
11	4.326	9.992	0.782
12	4.416	10.137	0.793
13	4.481	7.441	0.582
14	4.496	12.688	0.993
15	4.747	2.257	0.177
16	5.113	3.839	0.300

DAD1 B, Sig=254,8 Ref=550,60

Peak #	Ret. Time [min]	Area [mV *s]	Area %
1	1.692	5.211	0.220
2	3.106	2.320	0.098
3	3.323	0.892	0.038
4	3.382	2.479	0.105
5	3.428	10.404	0.440
6	3.449	27.080	1.144
7	3.481	8.386	0.354
8	3.569	1587.782	67.102
9	3.685	12.157	0.514
10	3.715	11.927	0.504
11	3.764	61.711	2.608
12	3.817	39.491	1.669
13	3.849	21.013	0.888
14	3.958	141.538	5.982
15	4.062	82.724	3.496
16	4.153	100.392	4.243
17	4.256	61.729	2.609
18	4.280	71.197	3.009
19	4.417	24.052	1.016
20	4.464	63.809	2.697
21	4.586	6.205	0.262
22	4.629	5.978	0.253
23	4.747	10.294	0.435
24	4.799	2.786	0.118
25	5.115	4.653	0.197

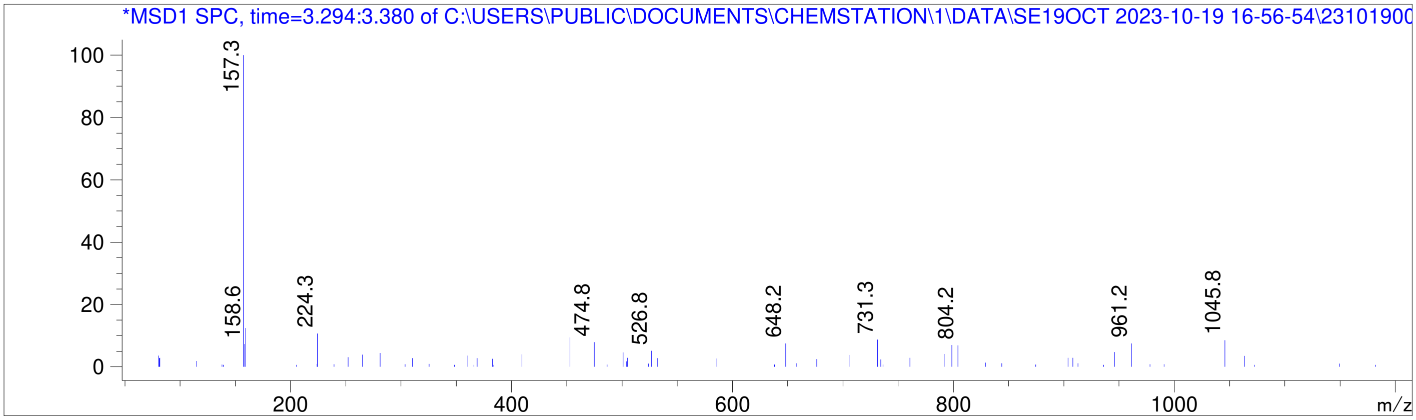
*MSD1 SPC, time=3.294:3.380 of C:\USERS\PUBLIC\DOCUMENTS\CHEMSTATION\1\DATA\SE19OCT 2023-10-19 16-56-54\23101900
157.3
158.6
224.3
474.8
526.8
648.2
731.3
804.2
961.2
1045.8
m/z

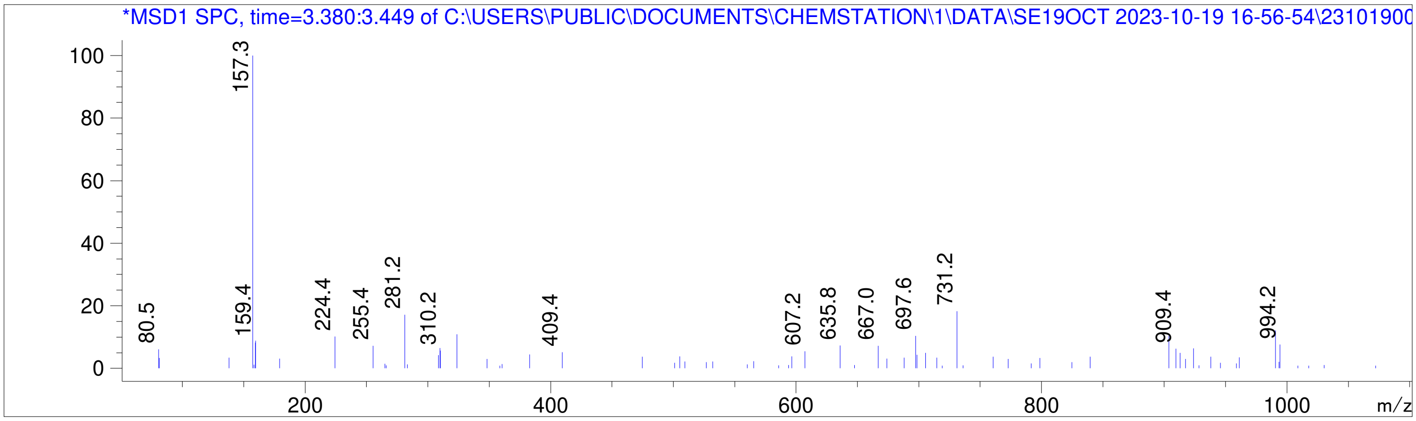
*MSD1 SPC, time=3.380:3.449 of C:\USERS\PUBLIC\DOCUMENTS\CHEMSTATION\1\DATA\SE19OCT 2023-10-19 16-56-54\23101900
157.3
80.5
159.4
224.4
255.4
281.2
310.2
409.4
607.2
635.8
667.0
697.6
731.2
909.4
994.2
m/z

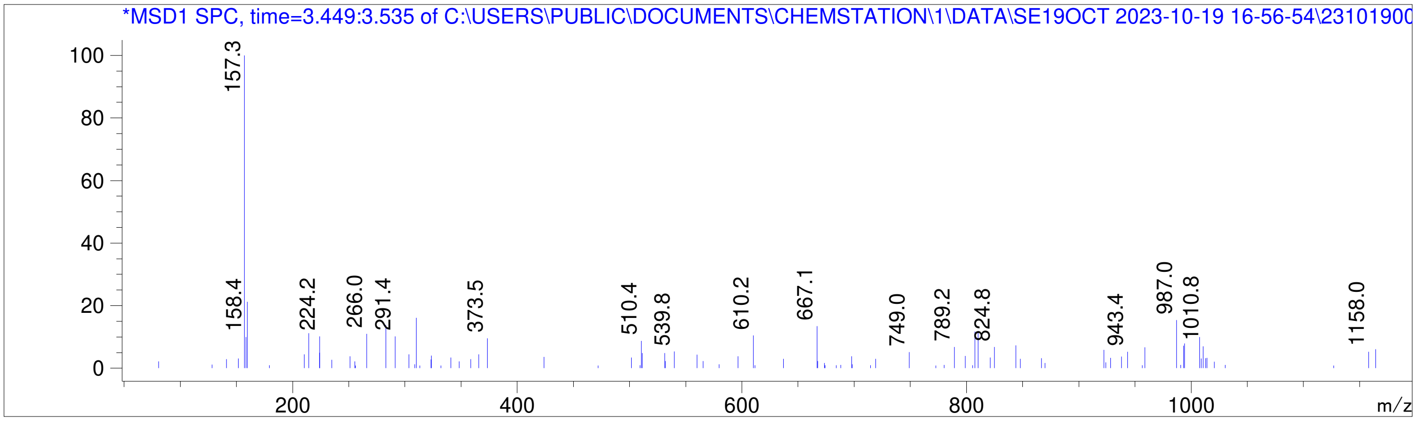
*MSD1 SPC, time=3.449:3.535 of C:\USERS\PUBLIC\DOCUMENTS\CHEMSTATION\1\DATA\SE19OCT 2023-10-19 16-56-54\23101900
157.3
158.4
224.2
266.0
291.4
373.5
510.4
539.8
610.2
667.1
749.0
789.2
824.8
943.4
987.0
1010.8
1158.0
m/z

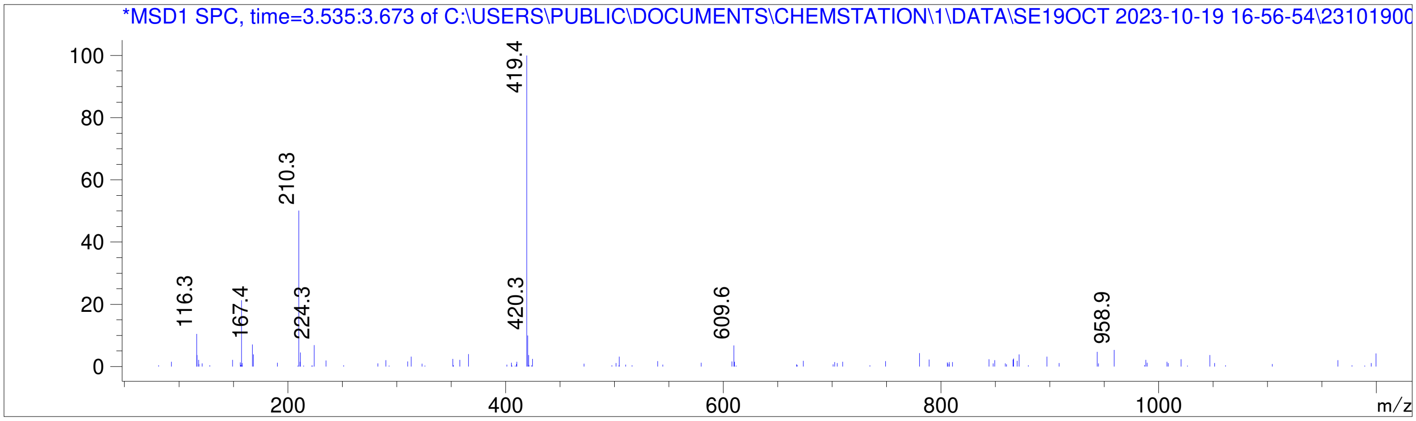
*MSD1 SPC, time=3.535:3.673 of C:\USERS\PUBLIC\DOCUMENTS\CHEMSTATION\1\DATA\SE19OCT 2023-10-19 16-56-54\23101900
419.4
210.3
116.3
167.4
224.3
420.3
609.6
958.9
m/z

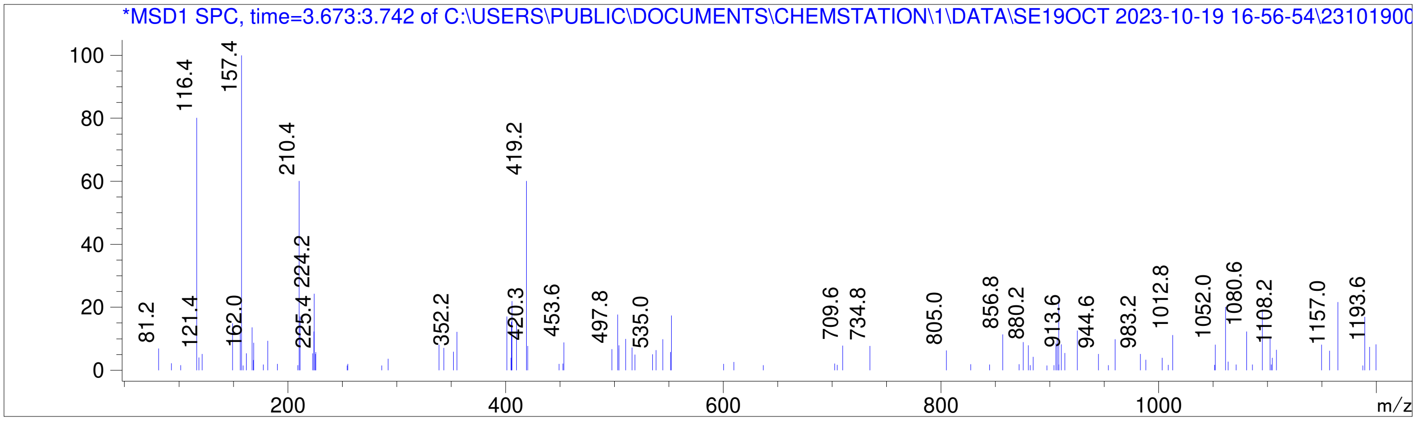
*MSD1 SPC, time=3.673:3.742 of C:\USERS\PUBLIC\DOCUMENTS\CHEMSTATION\1\DATA\SE19OCT 2023-10-19 16-56-54\23101900
157.4
116.4
210.4
419.2
224.2
81.2
121.4
162.0
225.4
352.2
420.3
453.6
497.8
535.0
709.6
734.8
805.0
856.8
880.2
913.6
944.6
983.2
1012.8
1052.0
1080.6
1108.2
1157.0
1193.6
m/z

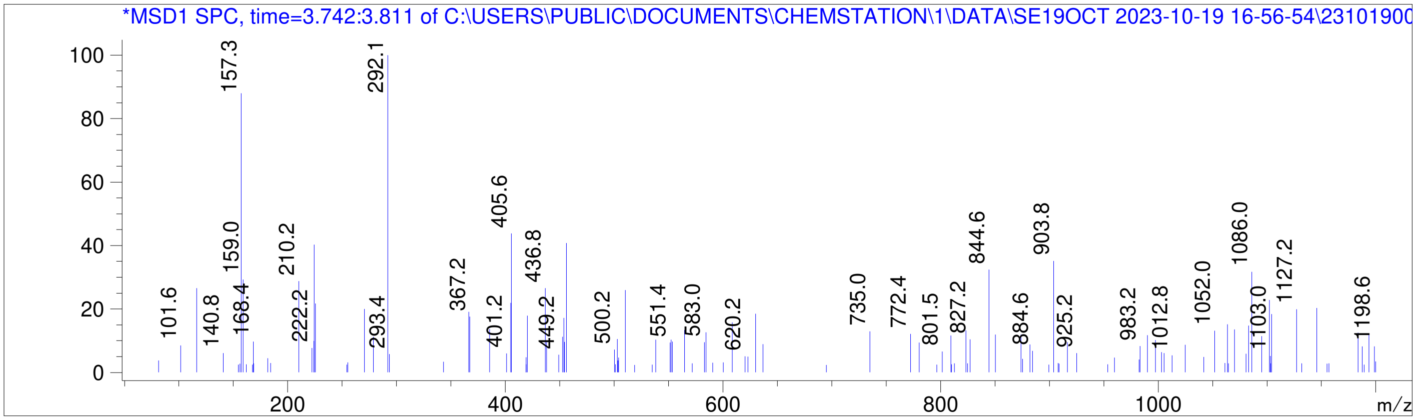
*MSD1 SPC, time=3.742:3.811 of C:\USERS\PUBLIC\DOCUMENTS\CHEMSTATION\1\DATA\SE19OCT 2023-10-19 16-56-54\23101900
157.3
292.1
405.6
436.8
159.0
210.2
101.6
140.8
168.4
222.2
293.4
367.2
401.2
449.2
500.2
551.4
583.0
620.2
735.0
772.4
801.5
827.2
844.6
884.6
903.8
925.2
983.2
1012.8
1052.0
1086.0
1103.0
1127.2
1198.6
m/z

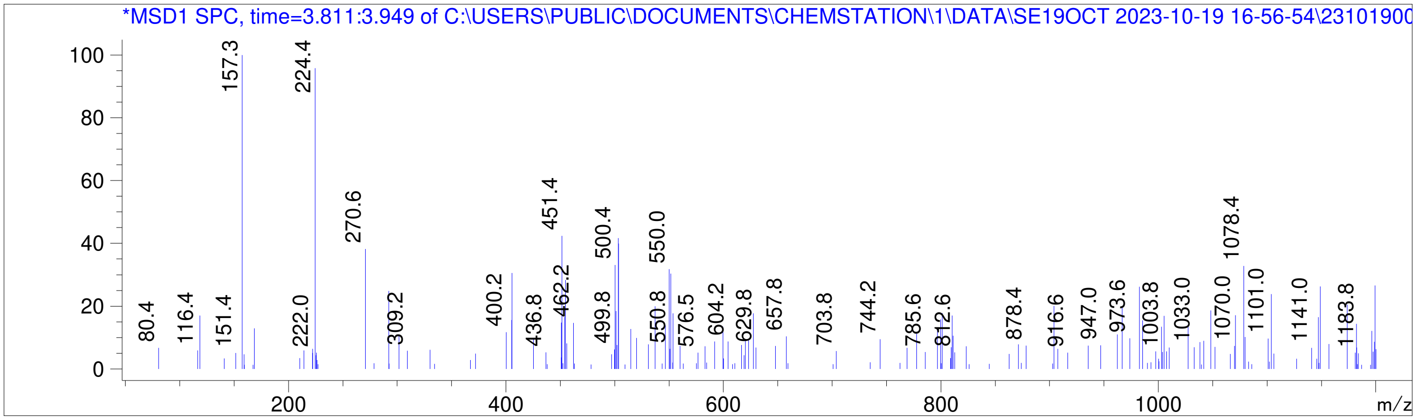
*MSD1 SPC, time=3.811:3.949 of C:\USERS\PUBLIC\DOCUMENTS\CHEMSTATION\1\DATA\SE19OCT 2023-10-19 16-56-54\23101900
157.3
224.4
270.6
451.4
500.4
550.0
1078.4
80.4
116.4
151.4
222.0
309.2
400.2
436.8
462.2
499.8
550.8
576.5
604.2
629.8
657.8
703.8
744.2
785.6
812.6
878.4
916.6
947.0
973.6
1003.8
1033.0
1070.0
1101.0
1141.0
1183.8
m/z

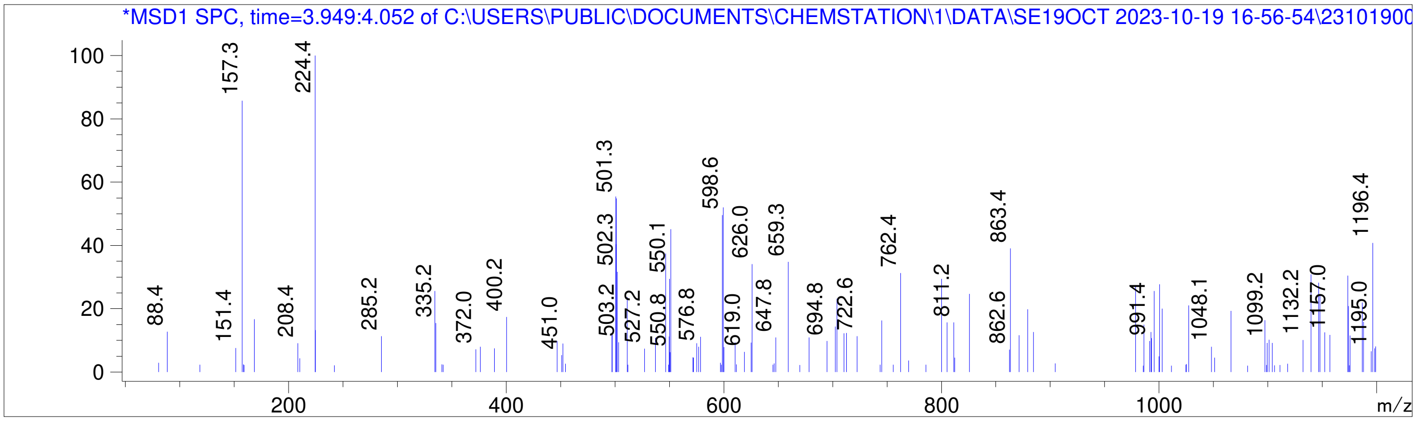
*MSD1 SPC, time=3.949:4.052 of C:\USERS\PUBLIC\DOCUMENTS\CHEMSTATION\1\DATA\SE19OCT 2023-10-19 16-56-54\23101900
88.4
151.4
157.3
208.4
224.4
285.2
335.2
372.0
400.2
451.0
503.2
502.3
501.3
527.2
550.8
550.1
576.8
598.6
619.0
626.0
647.8
659.3
694.8
722.6
762.4
811.2
862.6
863.4
991.4
1048.1
1099.2
1132.2
1157.0
1195.0
1196.4
m/z

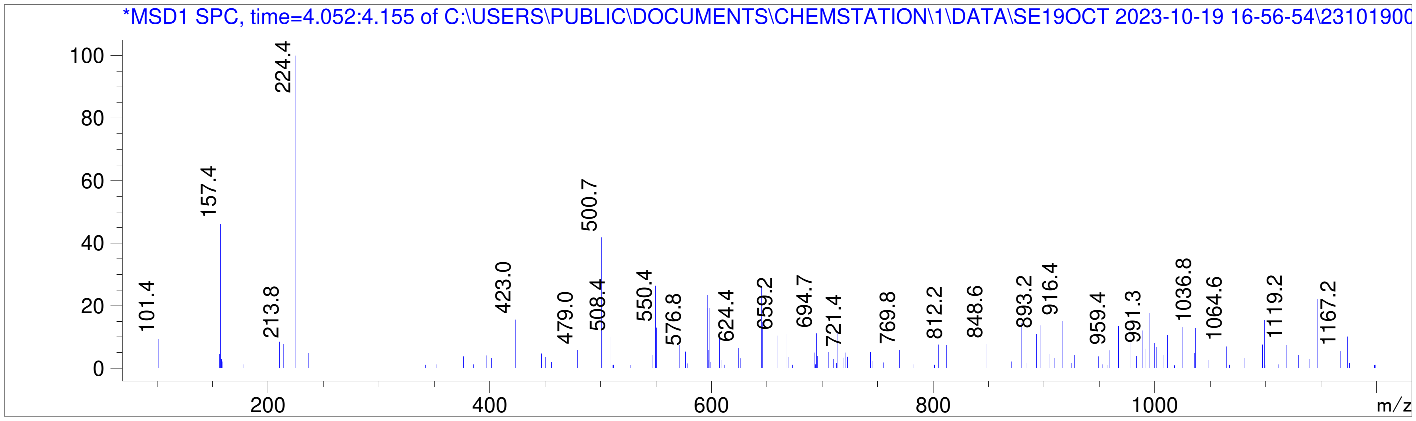
*MSD1 SPC, time=4.052:4.155 of C:\USERS\PUBLIC\DOCUMENTS\CHEMSTATION\1\DATA\SE19OCT 2023-10-19 16-56-54\23101900
101.4
157.4
213.8
224.4
423.0
479.0
508.4
500.7
550.4
576.8
624.4
659.2
694.7
721.4
769.8
812.2
848.6
893.2
916.4
959.4
991.3
1036.8
1064.6
1119.2
1167.2
m/z

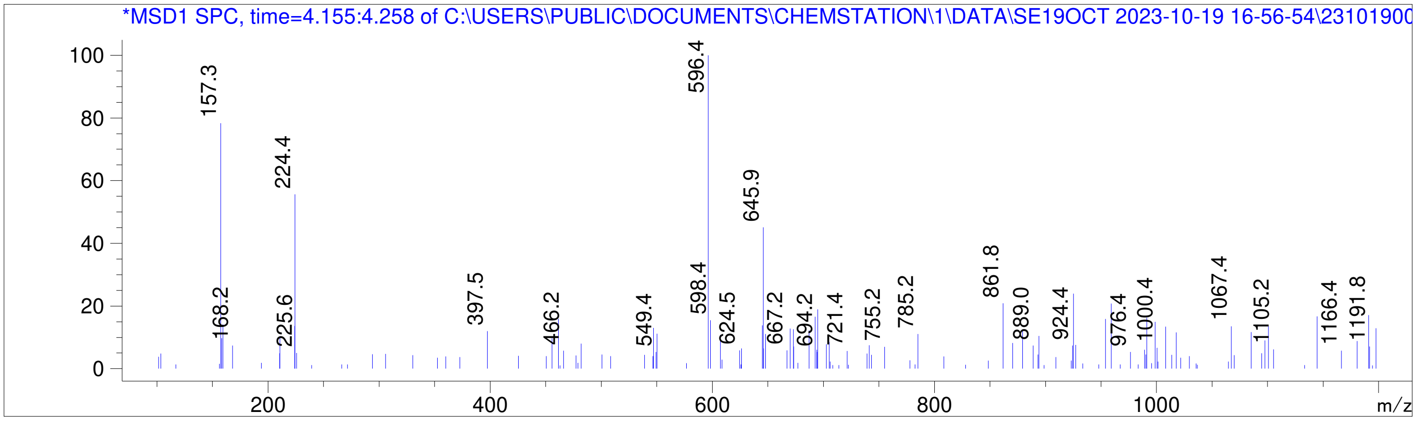
*MSD1 SPC, time=4.155:4.258 of C:\USERS\PUBLIC\DOCUMENTS\CHEMSTATION\1\DATA\SE19OCT 2023-10-19 16-56-54\23101900
157.3
168.2
225.6
224.4
397.5
466.2
549.4
598.4
596.4
624.5
645.9
667.2
694.2
721.4
755.2
785.2
861.8
889.0
924.4
976.4
1000.4
1067.4
1105.2
1166.4
1191.8
m/z

Sample-> CPT22010446-20-B1-80dg-24h

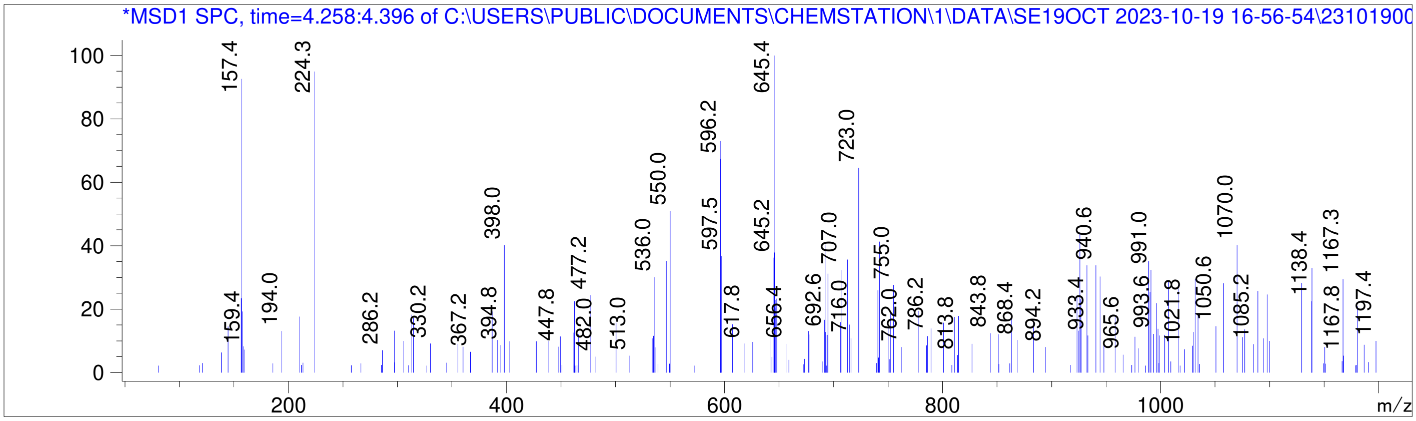

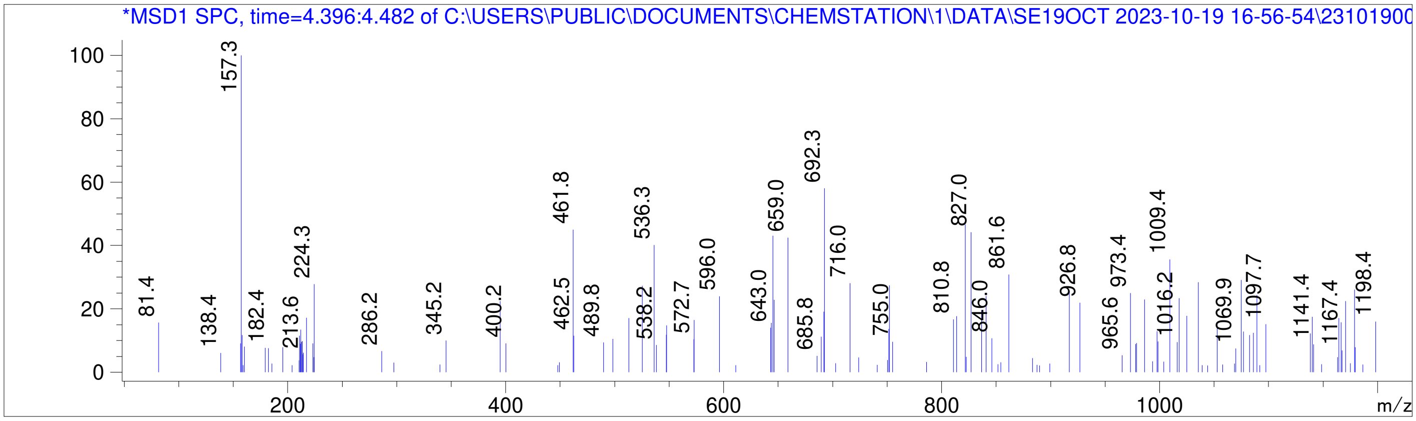

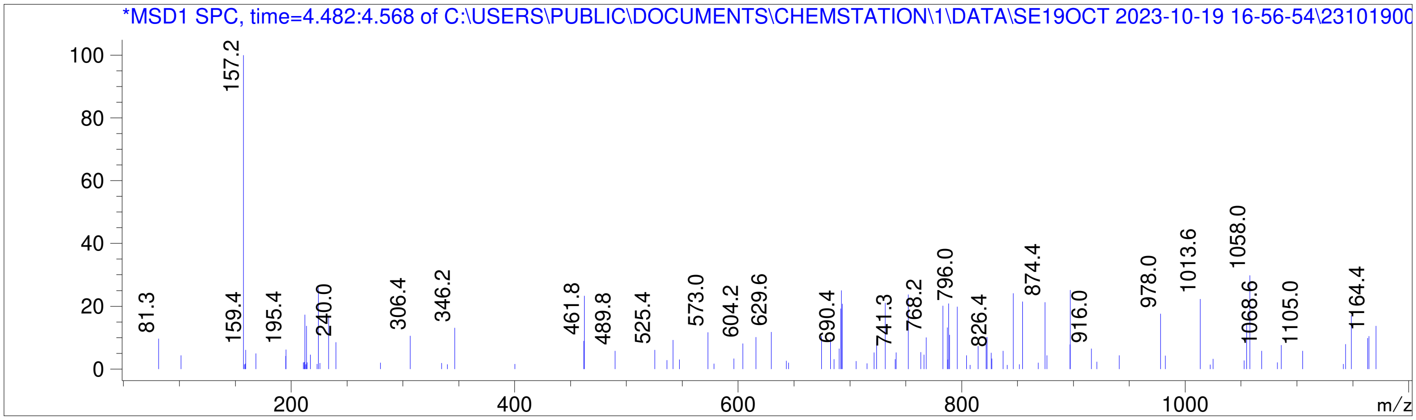

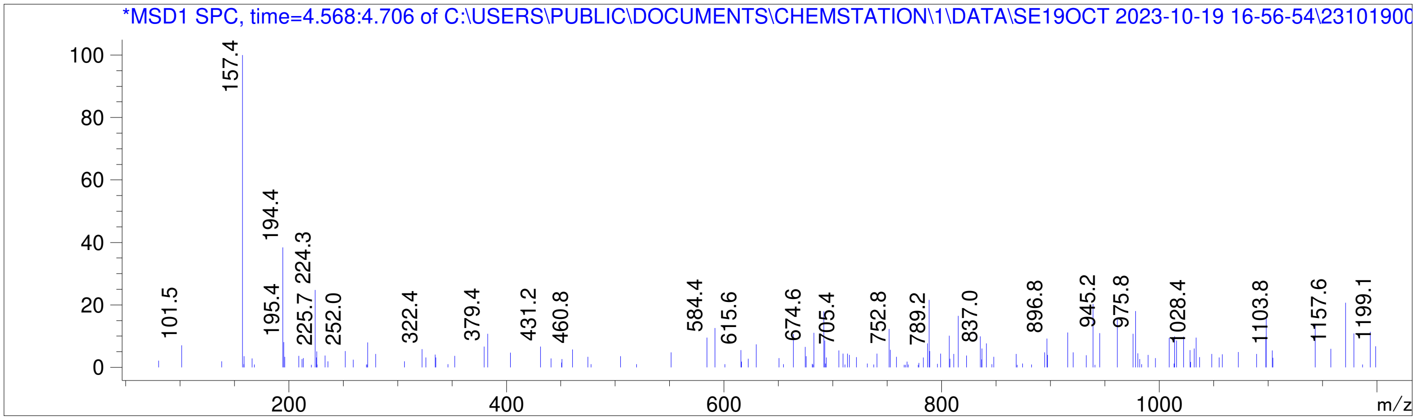

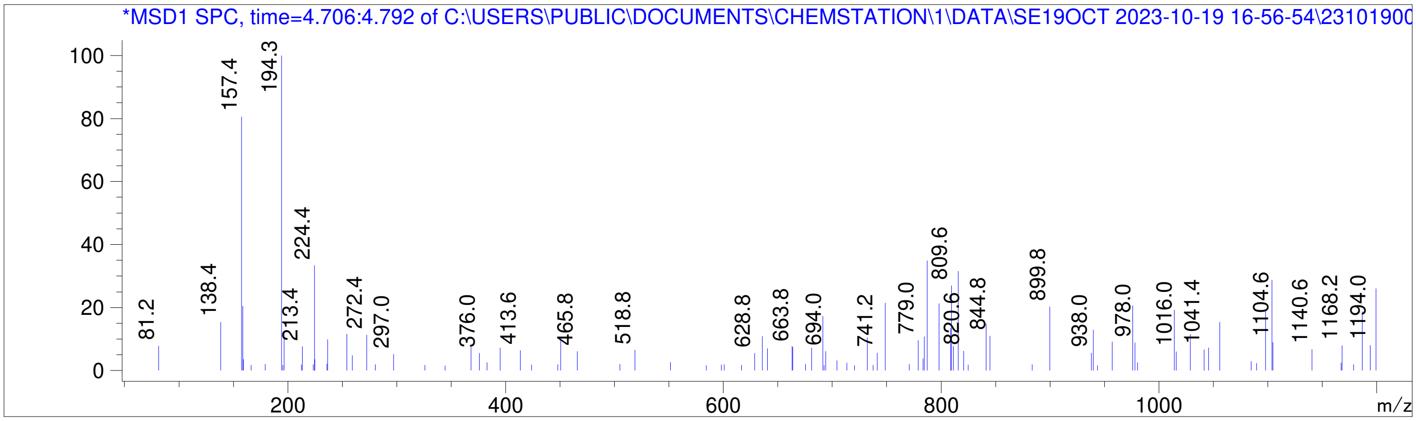
*MSD1 SPC, time=4.706:4.792 of C:\USERS\PUBLIC\DOCUMENTS\CHEMSTATION\1\DATA\SE19OCT 2023-10-19 16-56-54\23101900
100
80
60
40
20
0
81.2
138.4
157.4
194.3
213.4
224.4
272.4
297.0
376.0
413.6
465.8
518.8
628.8
663.8
694.0
741.2
779.0
809.6
820.6
844.8
899.8
938.0
978.0
1016.0
1041.4
1104.6
1140.6
1168.2
1194.0
200
400
600
800
1000
m/z

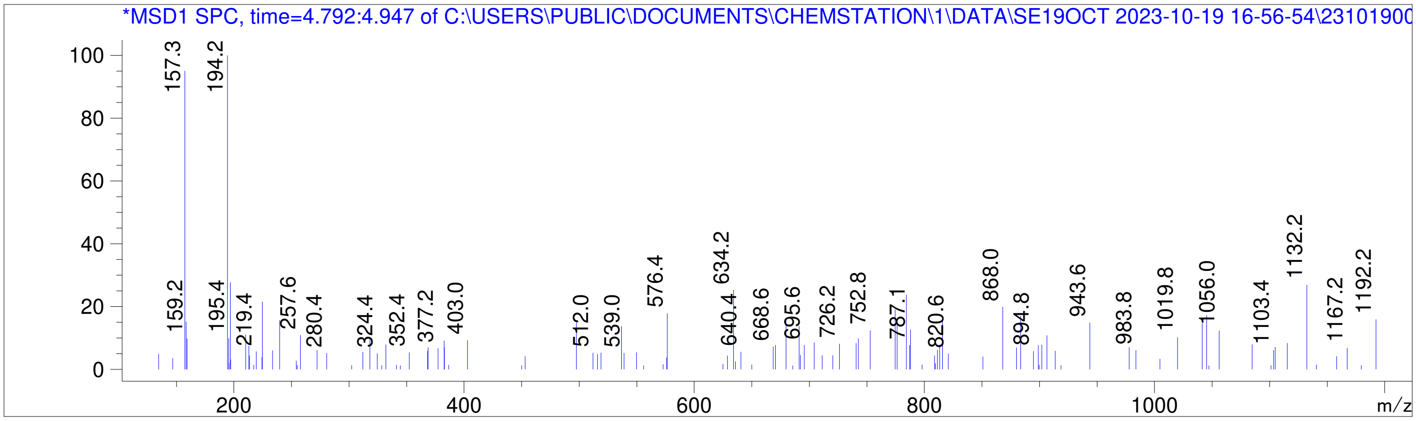
*MSD1 SPC, time=4.792:4.947 of C:\USERS\PUBLIC\DOCUMENTS\CHEMSTATION\1\DATA\SE19OCT 2023-10-19 16-56-54\23101900
100
80
60
40
20
0
157.3
159.2
194.2
195.4
219.4
257.6
280.4
324.4
352.4
377.2
403.0
512.0
539.0
576.4
634.2
640.4
668.6
695.6
726.2
752.8
787.1
820.6
868.0
894.8
943.6
983.8
1019.8
1056.0
1103.4
1132.2
1167.2
1192.2
200
400
600
800
1000
m/z
