# Supplementary material for: Astrobiological implications of the stability and reactivity of peptide nucleic acid (PNA) in concentrated sulfuric acid
Source: Sci Adv. 2025 Mar 26;11(13):eadr0006. doi: 10.1126/sciadv.adr0006 (PMC11939054; doi:10.1126/sciadv.adr0006)

Data -> C:\Users\Public\Documents\ChemStation\1\Data\SE07NOV 2023-11-07 14-56-21\ ->
Sample-> CPT22010446-20-B2-80deg-24h

===

Injection Date	:	Wed, 8. Nov. 2023	Seq Line	:	38
			Location	:	75
			Inj. Vol.	:	2 µl

Acq. Method : C:\Users\Public\Documents\ChemStation\1\Data\SE07NOV 2023-11-07 14-56-21\22010446 LCMS-6.M
Analysis Method : C:\Users\Public\Documents\ChemStation\1\Data\SE07NOV 2023-11-07 14-56-21\22010446 LCMS-6.M (Sequence Method)
Waters XBridge Phenyl (4.6 * 150 mm; 3.5 µm); 0.05% TFA (aq) / AcN: 100/0 (0.0 min) --> (6.0 min) --> 70/30 (0.0 min) --> (2.0 min) --> 10/90 (2.0 min); Flow: 1.0 ml/min;
MSD1 = positive; MSD2 = negative

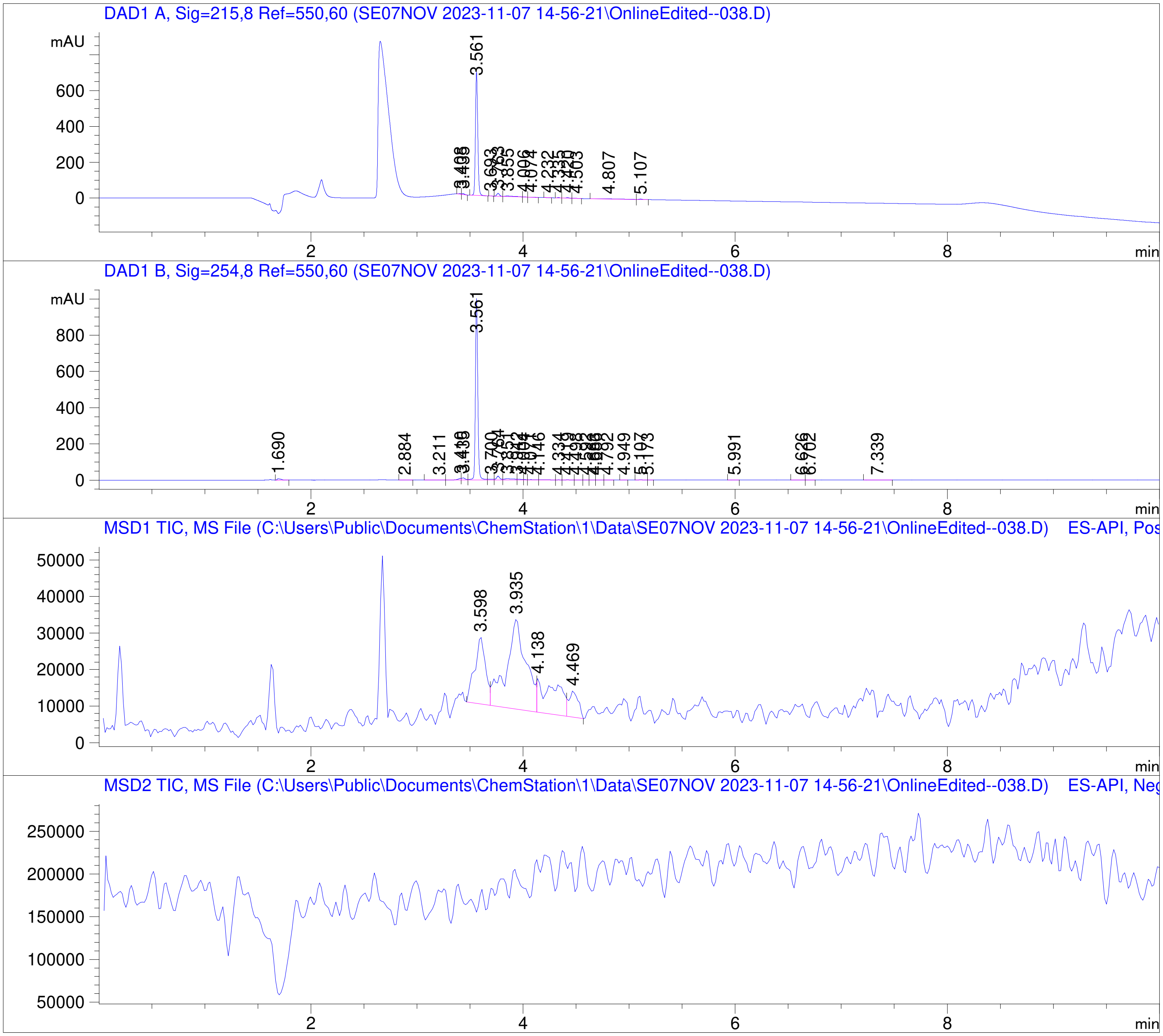

DAD1 A, Sig=215,8 Ref=550,60

Peak #	Ret. Time [min]	Area [mV *s]	Area %
1	3.408	5.508	0.477
2	3.435	8.052	0.698
3	3.561	1048.205	90.817
4	3.693	0.769	0.067
5	3.763	28.968	2.510
6	3.855	21.625	1.874
7	4.006	0.951	0.082
8	4.074	1.196	0.104
9	4.232	1.021	0.088
10	4.335	3.729	0.323
11	4.420	7.426	0.643
12	4.503	1.943	0.168
13	4.807	19.434	1.684
14	5.107	5.372	0.465

DAD1 B, Sig=254,8 Ref=550,60

Peak #	Ret. Time [min]	Area [mV *s]	Area %
1	1.690	19.497	1.081
2	2.884	0.457	0.025
3	3.211	2.518	0.140
4	3.410	20.772	1.151
5	3.435	27.980	1.551
6	3.561	1547.591	85.773
7	3.700	17.940	0.994
8	3.764	51.157	2.835
9	3.851	48.767	2.703
10	3.942	14.999	0.831
11	4.004	5.619	0.311
12	4.071	14.194	0.787
13	4.146	13.398	0.743
14	4.334	3.233	0.179
15	4.419	5.474	0.303
16	4.498	0.950	0.053
17	4.592	0.503	0.028
18	4.663	0.478	0.027
19	4.686	0.336	0.019
20	4.792	0.158	0.009
21	4.949	0.166	0.009
22	5.107	5.605	0.311
23	5.173	0.138	0.008
24	5.991	0.217	0.012
25	6.626	0.745	0.041
26	6.702	0.139	0.008
27	7.339	1.255	0.070

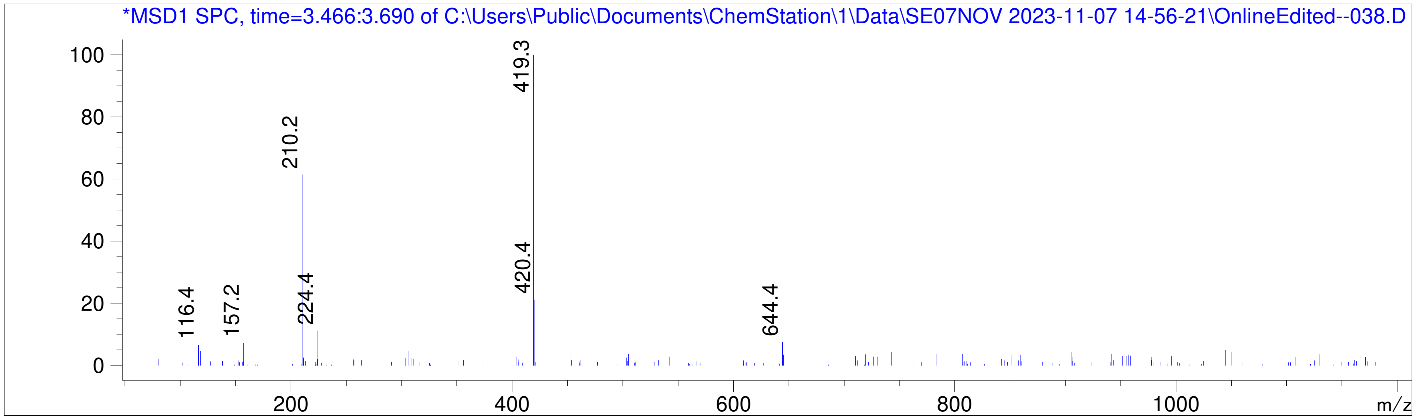
*MSD1 SPC, time=3.466:3.690 of C:\Users\Public\Documents\ChemStation\1\Data\SE07NOV 2023-11-07 14-56-21\OnlineEdited--038.D
419.3
210.2
420.4
116.4
157.2
224.4
644.4
m/z

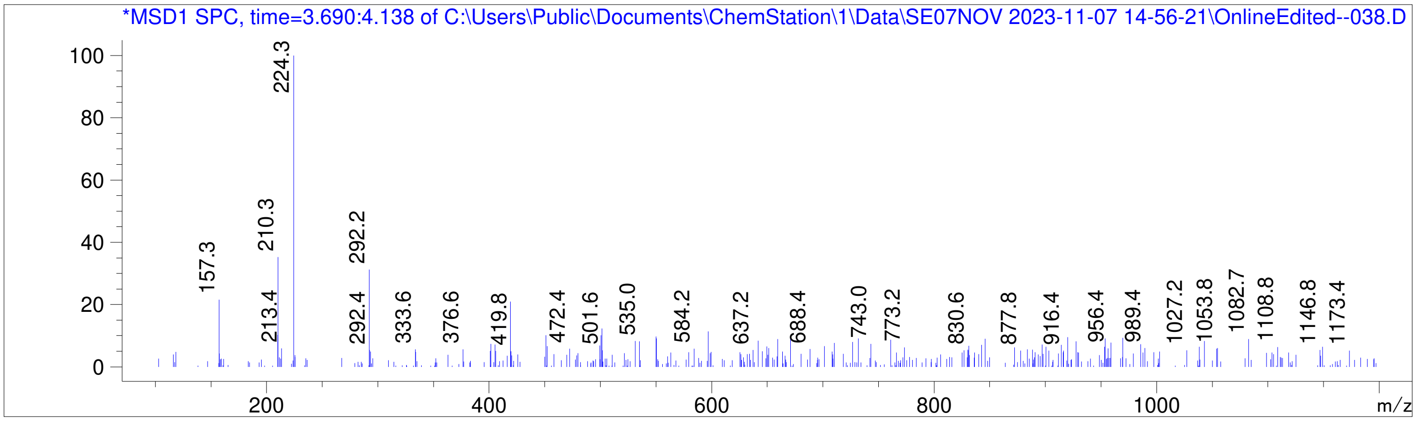
*MSD1 SPC, time=3.690:4.138 of C:\Users\Public\Documents\ChemStation\1\Data\SE07NOV 2023-11-07 14-56-21\OnlineEdited--038.D
224.3
210.3
292.2
157.3
213.4
292.4
333.6
376.6
419.8
472.4
501.6
535.0
584.2
637.2
688.4
743.0
773.2
830.6
877.8
916.4
956.4
989.4
1027.2
1053.8
1082.7
1108.8
1146.8
1173.4
m/z

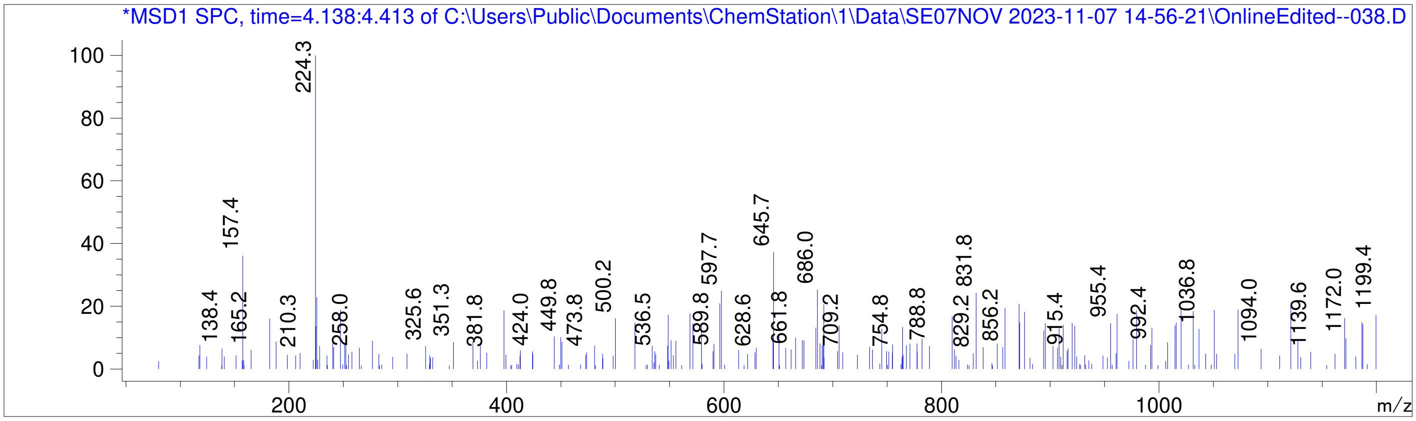
*MSD1 SPC, time=4.138:4.413 of C:\Users\Public\Documents\ChemStation\1\Data\SE07NOV 2023-11-07 14-56-21\OnlineEdited--038.D
224.3
157.4
645.7
686.0
597.7
831.8
138.4
165.2
210.3
258.0
325.6
351.3
381.8
424.0
449.8
473.8
500.2
536.5
589.8
628.6
661.8
709.2
754.8
788.8
829.2
856.2
915.4
955.4
992.4
1036.8
1094.0
1139.6
1172.0
1199.4
m/z

Sample-> CPT22010446-20-B2-80deg-24h

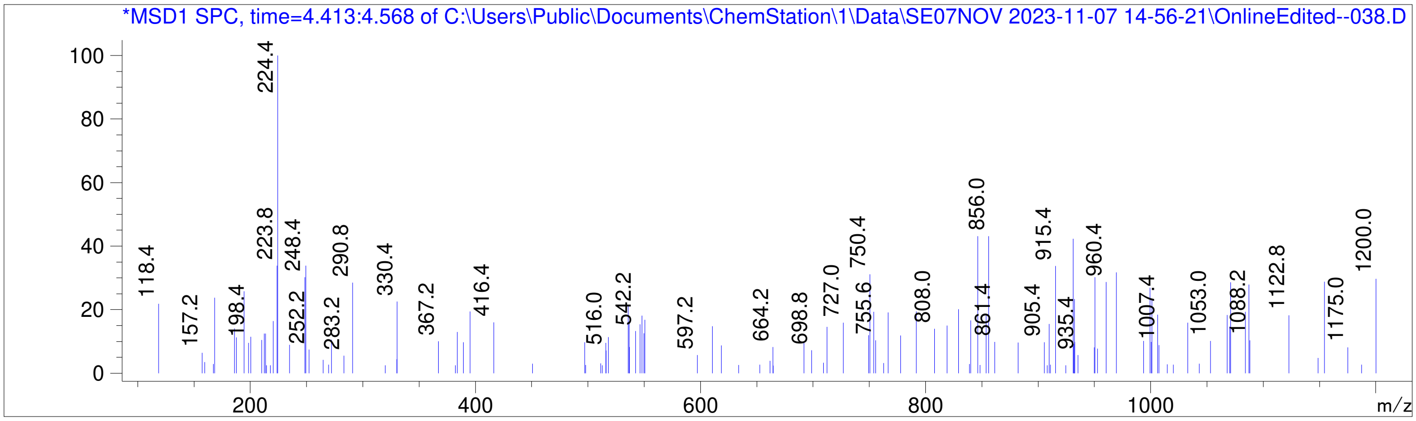

Supplement: Supplementary file 2 — Data S1 and S2 [file sciadv.adr0006_data_s1_and_s2.zip › Supplementary Dataset 1-LCMS DATA/LCMS PNA Hexamers A-T/LCMS G6 50C_80C/80C/24h/CPT22010446-20-B2-80deg-24h.pdf]
