# Supplementary material for: Astrobiological implications of the stability and reactivity of peptide nucleic acid (PNA) in concentrated sulfuric acid
Source: Sci Adv. 2025 Mar 26;11(13):eadr0006. doi: 10.1126/sciadv.adr0006 (PMC11939054; doi:10.1126/sciadv.adr0006)
Supplement: Supplementary file 2 — Data S1 and S2 [file sciadv.adr0006_data_s1_and_s2.zip › Supplementary Dataset 1-LCMS DATA/LCMS PNA Hexamers A-T/LCMS G6 RT/14d/CPT22010446-20-B-1-14d.pdf]

===

Injection Date : Mon, 9. Oct. 2023

Seq Line : 15
Location : 84
Inj. Vol. : 2 µl

Acq. Method : C:\Users\Public\Documents\ChemStation\1\Data\SE09OCT 2023-10-09 14-54-12\22010446 LCMS-6.M
Analysis Method : C:\Users\Public\Documents\ChemStation\1\Data\SE09OCT 2023-10-09 14-54-12\22010446 LCMS-6.M (Sequence Method)
Waters XBridge Phenyl (4.6 * 150 mm; 3.5 µm); 0.05% TFA (aq) / AcN: 100/0 (0.0 min) --> (6.0 min) --> 70/30 (0.0 min) --> (2.0 min) --> 10/90 (2.0 min); Flow: 1.0 ml/min;
MSD1 = positive; MSD2 = negative

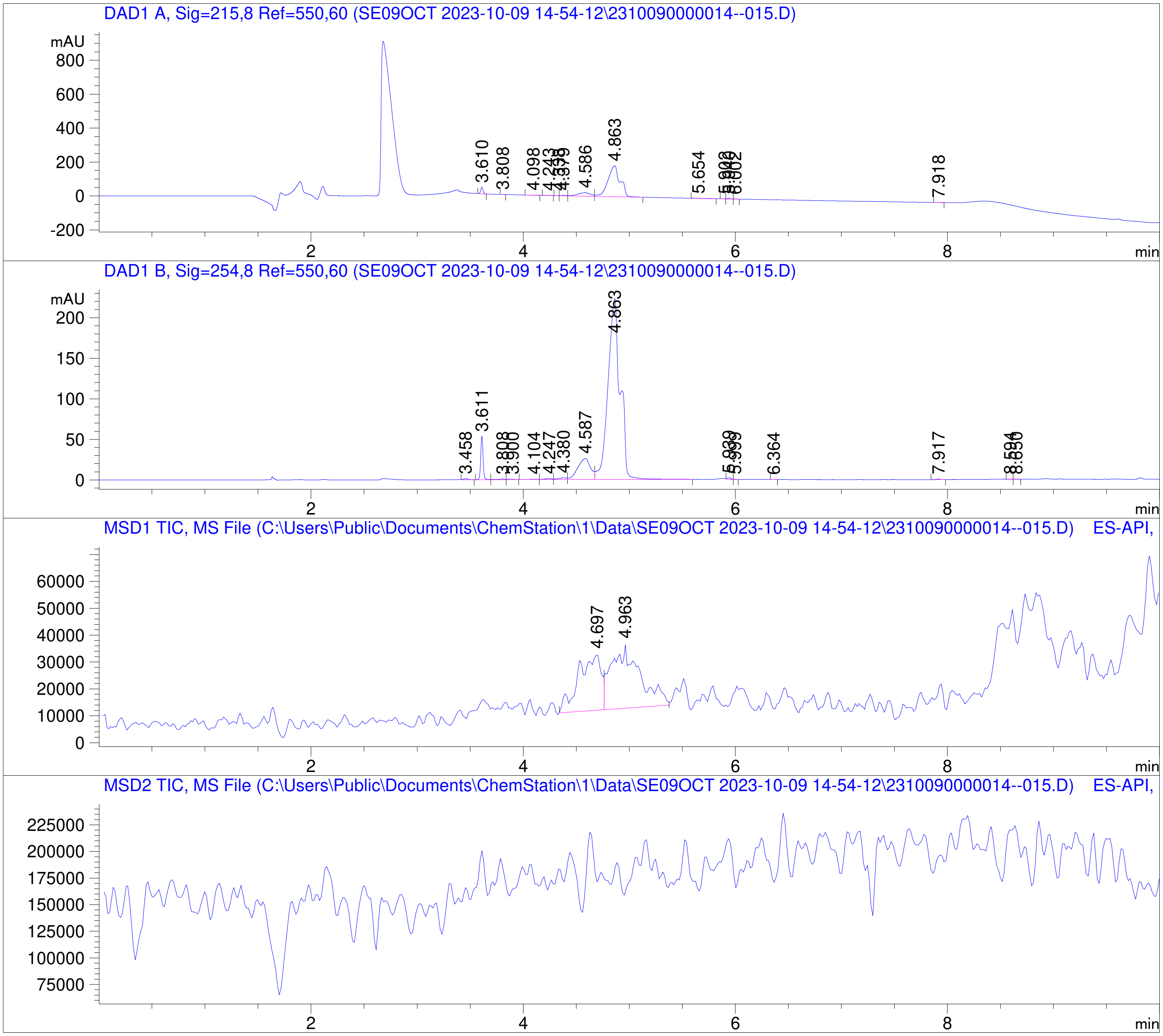

DAD1 A, Sig=215,8 Ref=550,60

Peak #	Ret. Time [min]	Area [mV *s]	Area %
1	3.610	54.542	3.210
2	3.808	0.861	0.051
3	4.098	2.690	0.158
4	4.243	0.927	0.055
5	4.338	0.608	0.036
6	4.379	7.126	0.419
7	4.586	165.623	9.748
8	4.863	1448.246	85.240
9	5.654	3.533	0.208
10	5.902	1.675	0.099
11	5.940	8.745	0.515
12	6.002	0.692	0.041
13	7.918	3.749	0.221

DAD1 B, Sig=254,8 Ref=550,60

Peak #	Ret. Time [min]	Area [mV *s]	Area %
1	3.458	3.613	0.171
2	3.611	81.545	3.858
3	3.808	3.451	0.163
4	3.900	2.512	0.119
5	4.104	2.586	0.122
6	4.247	5.429	0.257
7	4.380	11.529	0.546
8	4.587	215.021	10.174
9	4.863	1780.158	84.231
10	5.939	4.800	0.227
11	5.999	0.305	0.014
12	6.364	0.102	0.005
13	7.917	1.946	0.092
14	8.594	0.181	0.009
15	8.650	0.251	0.012

===

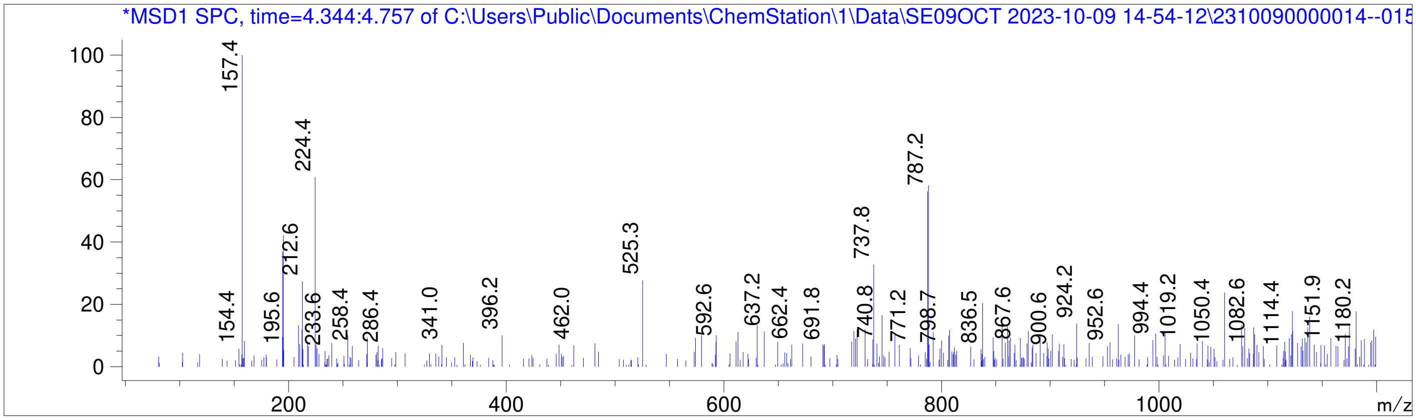
*MSD1 SPC, time=4.344:4.757 of C:\Users\Public\Documents\ChemStation\1\Data\SE09OCT 2023-10-09 14-54-12\2310090000014--015
157.4
224.4
212.6
787.2
737.8
525.3
154.4
195.6
233.6
258.4
286.4
341.0
396.2
462.0
592.6
637.2
662.4
691.8
740.8
771.2
798.7
836.5
867.6
900.6
924.2
952.6
994.4
1019.2
1050.4
1082.6
1114.4
1151.9
1180.2
m/z

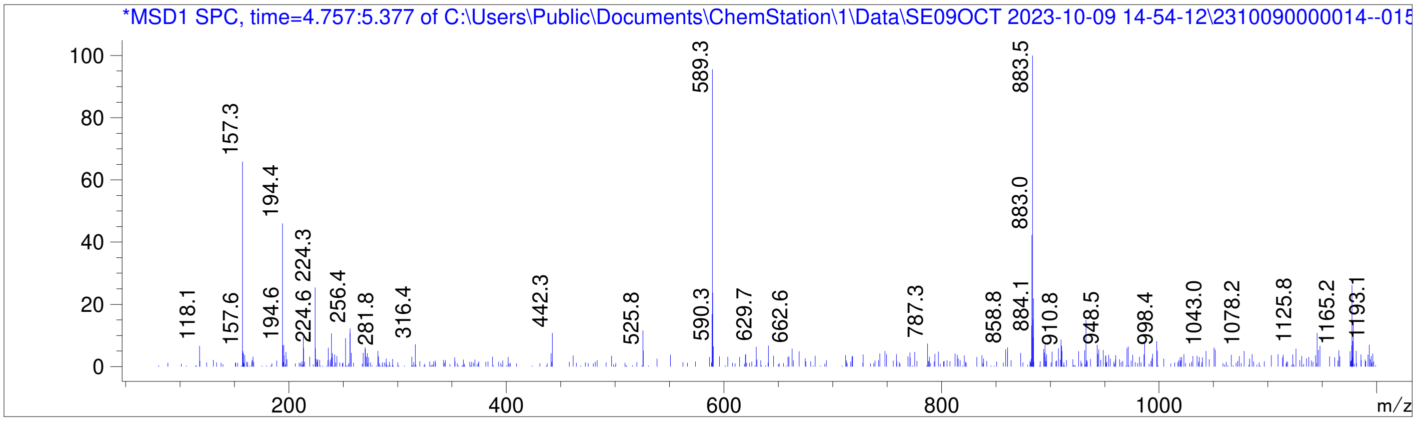
*MSD1 SPC, time=4.757:5.377 of C:\Users\Public\Documents\ChemStation\1\Data\SE09OCT 2023-10-09 14-54-12\2310090000014--015
589.3
883.5
157.3
194.4
883.0
224.3
118.1
157.6
194.6
224.6
256.4
281.8
316.4
442.3
525.8
590.3
629.7
662.6
787.3
858.8
884.1
910.8
948.5
998.4
1043.0
1078.2
1125.8
1165.2
1193.1
m/z
