# Supplementary material for: Astrobiological implications of the stability and reactivity of peptide nucleic acid (PNA) in concentrated sulfuric acid
Source: Sci Adv. 2025 Mar 26;11(13):eadr0006. doi: 10.1126/sciadv.adr0006 (PMC11939054; doi:10.1126/sciadv.adr0006)
Supplement: Supplementary file 2 — Data S1 and S2 [file sciadv.adr0006_data_s1_and_s2.zip › Supplementary Dataset 1-LCMS DATA/LCMS PNA Hexamers A-T/LCMS G6 RT/14d/CPT22010446-20-B2-14d.pdf]

Injection Date : Wed, 11. Oct. 2023

Seq Line : 23
Location : 21
Inj. Vol. : 2 µl

Acq. Method : C:\Users\Public\Documents\ChemStation\1\Data\SE11OCT 2023-10-11 13-11-06\22010446 LCMS-6.M
Analysis Method : C:\Users\Public\Documents\ChemStation\1\Data\SE11OCT 2023-10-11 13-11-06\22010446 LCMS-6.M (Sequence Method)
Waters XBridge Phenyl (4.6 * 150 mm; 3.5 µm); 0.05% TFA (aq) / AcN: 100/0 (0.0 min) --> (6.0 min) --> 70/30 (0.0 min) --> (2.0 min) --> 10/90 (2.0 min); Flow: 1.0 ml/min;
MSD1 = positive; MSD2 = negative

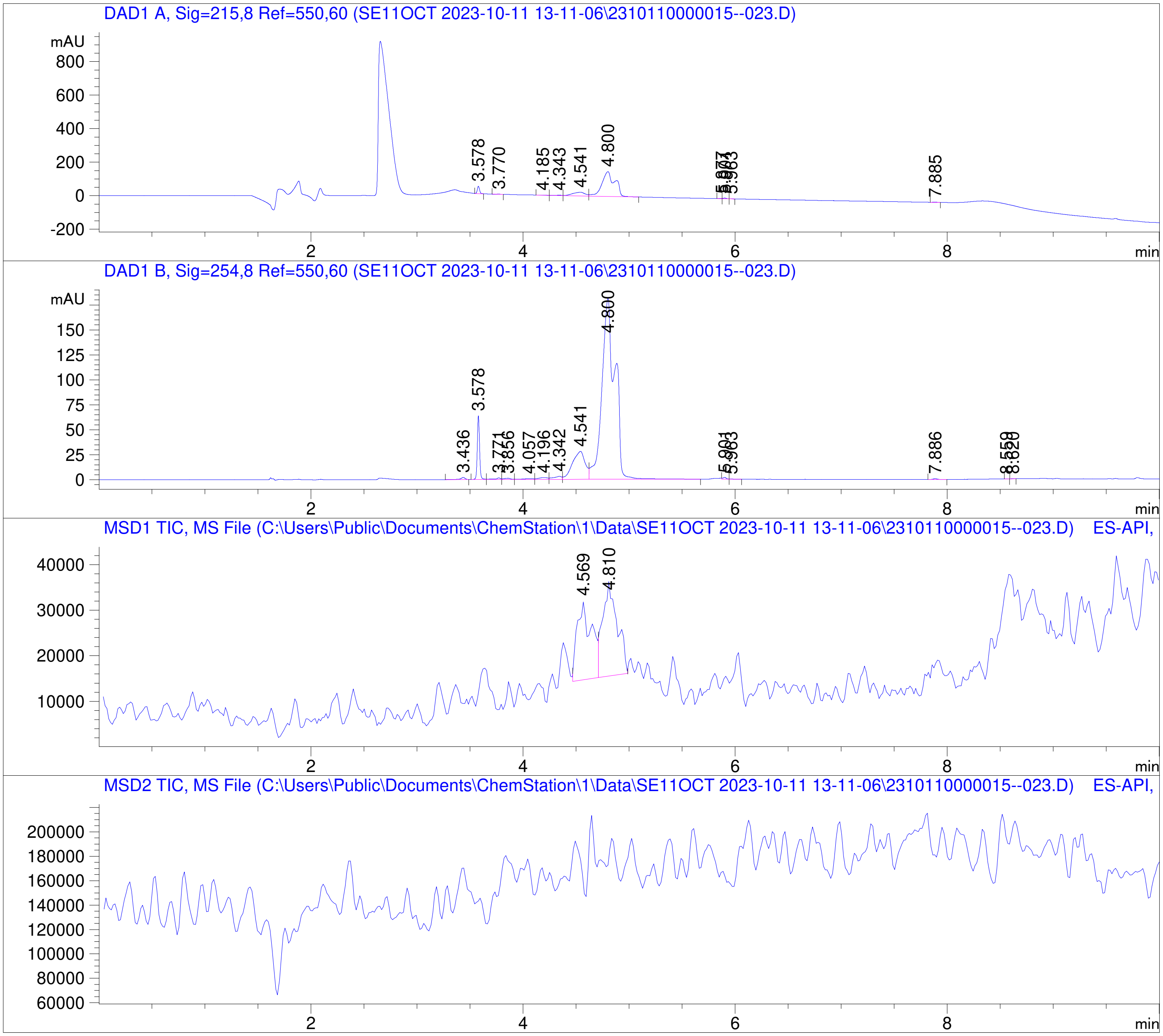

DAD1 A, Sig=215,8 Ref=550,60

Peak #	Ret. Time [min]	Area [mV *s]	Area %
1	3.578	62.106	4.067
2	3.770	2.763	0.181
3	4.185	2.455	0.161
4	4.343	8.575	0.562
5	4.541	177.864	11.648
6	4.800	1258.683	82.428
7	5.877	1.659	0.109
8	5.901	8.218	0.538
9	5.963	1.206	0.079
10	7.885	3.478	0.228

DAD1 B, Sig=254,8 Ref=550,60

Peak #	Ret. Time [min]	Area [mV *s]	Area %
1	3.436	5.835	0.301
2	3.578	93.467	4.824
3	3.771	5.386	0.278
4	3.856	4.808	0.248
5	4.057	5.148	0.266
6	4.196	10.504	0.542
7	4.342	16.306	0.842
8	4.541	233.340	12.044
9	4.800	1555.034	80.261
10	5.901	4.559	0.235
11	5.963	0.816	0.042
12	7.886	1.910	0.099
13	8.559	0.147	0.008
14	8.620	0.207	0.011

==

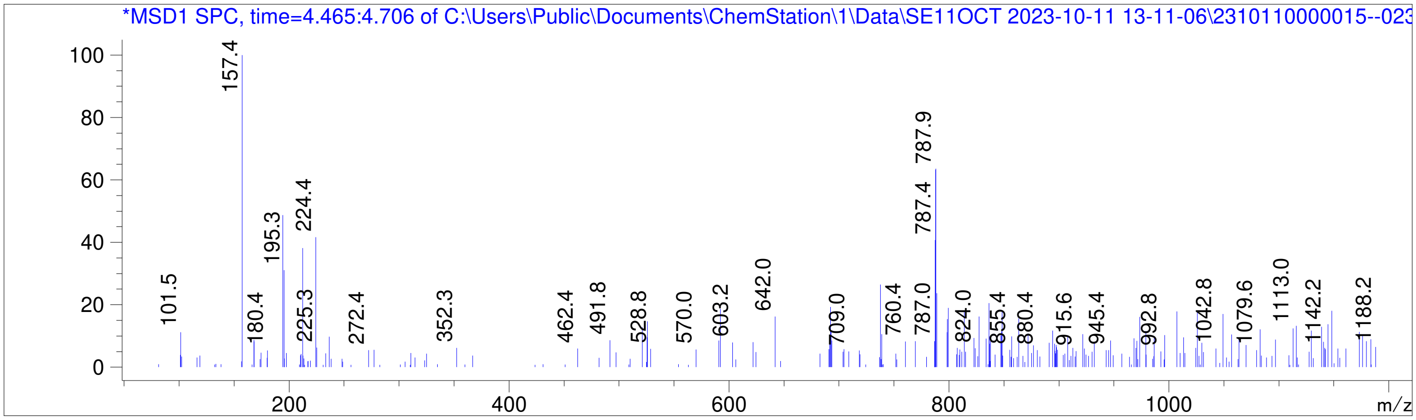
*MSD1 SPC, time=4.465:4.706 of C:\Users\Public\Documents\ChemStation\1\Data\SE11OCT 2023-10-11 13-11-06\2310110000015--023
157.4
787.9
787.4
224.4
195.3
101.5
180.4
225.3
272.4
352.3
462.4
491.8
528.8
570.0
603.2
642.0
709.0
760.4
787.0
824.0
855.4
880.4
915.6
945.4
992.8
1042.8
1079.6
1113.0
1142.2
1188.2
m/z

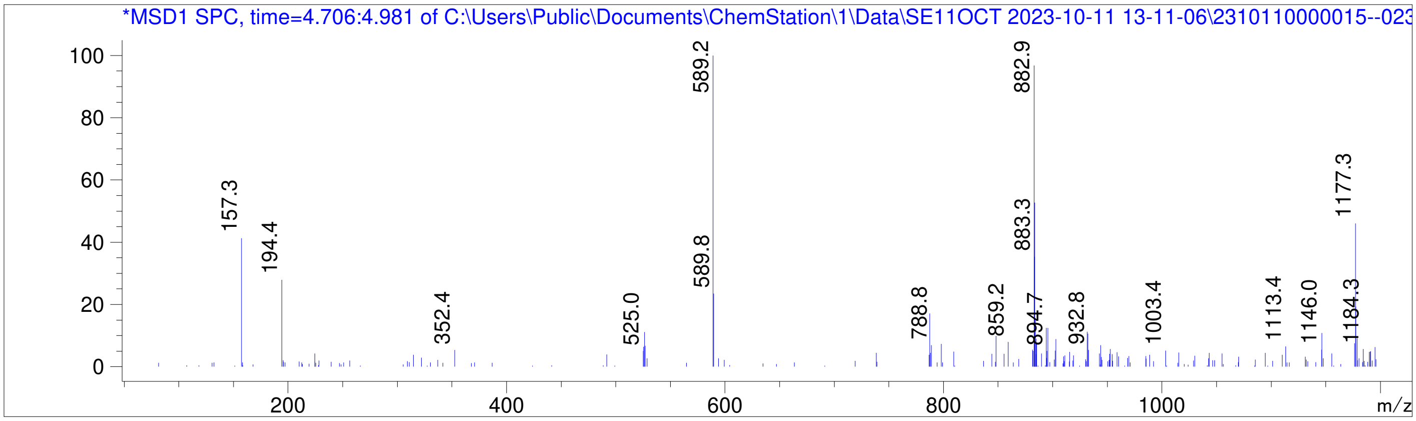
*MSD1 SPC, time=4.706:4.981 of C:\Users\Public\Documents\ChemStation\1\Data\SE11OCT 2023-10-11 13-11-06\2310110000015--023
589.2
882.9
157.3
194.4
883.3
589.8
1177.3
352.4
525.0
788.8
859.2
894.7
932.8
1003.4
1113.4
1146.0
1184.3
m/z
