# Supplementary material for: Astrobiological implications of the stability and reactivity of peptide nucleic acid (PNA) in concentrated sulfuric acid
Source: Sci Adv. 2025 Mar 26;11(13):eadr0006. doi: 10.1126/sciadv.adr0006 (PMC11939054; doi:10.1126/sciadv.adr0006)
Supplement: Supplementary file 2 — Data S1 and S2 [file sciadv.adr0006_data_s1_and_s2.zip › Supplementary Dataset 1-LCMS DATA/LCMS PNA Hexamers A-T/LCMS G6 RT/14d/CPT22010446-20-B3-14d.pdf]

Data -> C:\Users\Public\Documents\ChemStation\1\Data\SE17OCT 2023-10-17 07-53-29\   ->
Sample-> CPT22010446-20-B3-14d

Injection Date	:	Tue, 17. Oct. 2023	Seq Line	:	15
			Location	:	21
			Inj. Vol.	:	2 µl

Acq. Method   : C:\Users\Public\Documents\ChemStation\1\Data\SE17OCT 2023-10-17 07-53-29\22010446 LCMS-6.M

Analysis Method : C:\Users\Public\Documents\ChemStation\1\Data\SE17OCT 2023-10-17 07-53-29\22010446 LCMS-6.M (Sequence Method)

Waters XBridge Phenyl (4.6 * 150 mm; 3.5 µm); 0.05% TFA (aq) / AcN: 100/0 (0.0 min) --> (6.0 min) --> 70/30 (0.0 min) --> (2.0 min) --> 10/90 (2.0 min); Flow: 1.0 ml/min; MSD1 = positive; MSD2 = negative

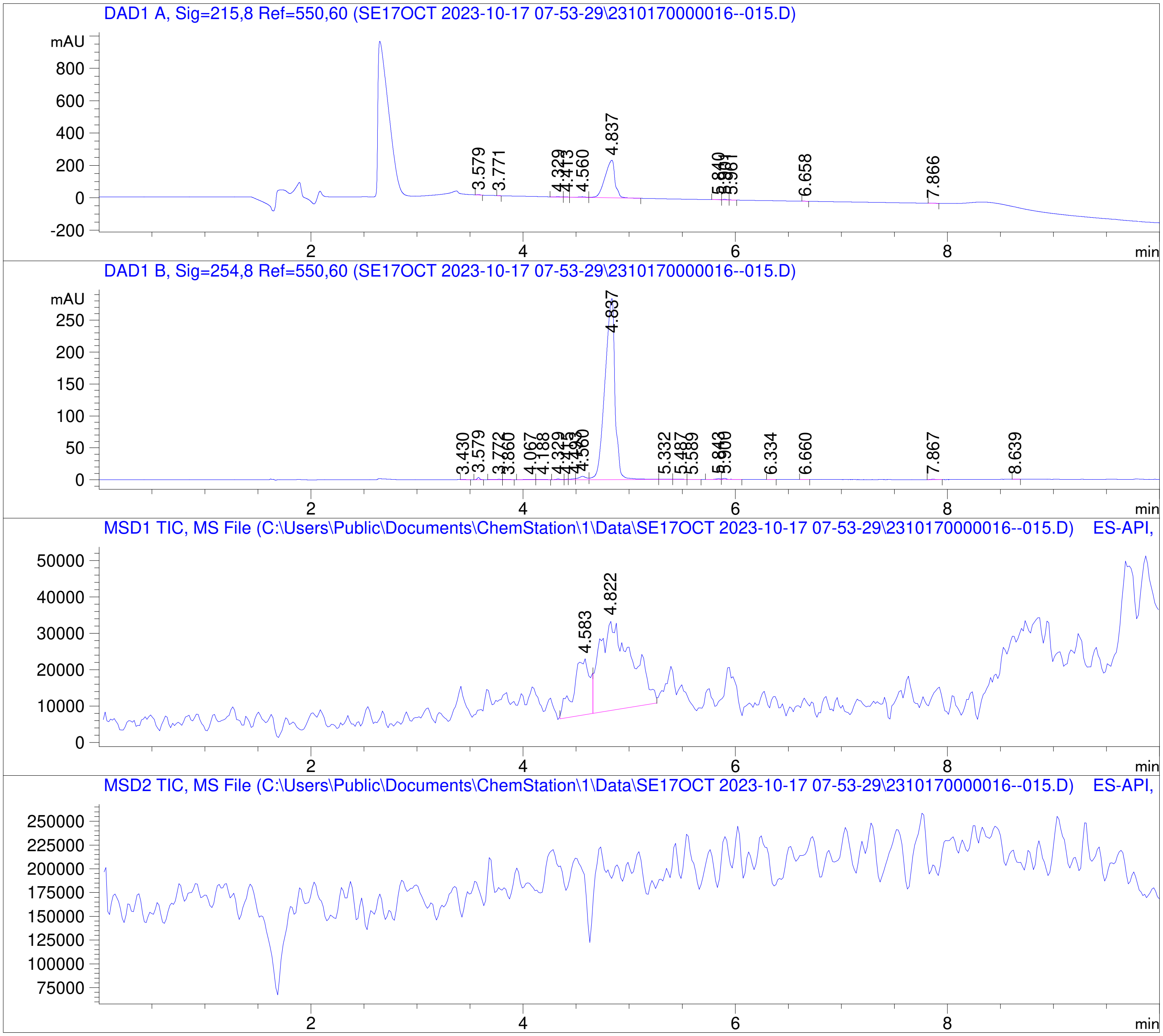

Sample-> CPT22010446-20-B3-14d

DAD1 A, Sig=215,8 Ref=550,60

Peak #	Ret. Time [min]	Area [mV *s]	Area %
1	3.579	4.763	0.303
2	3.771	0.544	0.035
3	4.329	4.514	0.287
4	4.413	1.856	0.118
5	4.560	18.820	1.197
6	4.837	1526.265	97.050
7	5.840	3.342	0.213
8	5.901	7.719	0.491
9	5.961	1.147	0.073
10	6.658	0.394	0.025
11	7.866	3.291	0.209

DAD1 B, Sig=254,8 Ref=550,60

Peak #	Ret. Time [min]	Area [mV *s]	Area %
1	3.430	1.164	0.060
2	3.579	5.081	0.263
3	3.772	1.658	0.086
4	3.860	0.686	0.035
5	4.067	1.444	0.075
6	4.188	1.196	0.062
7	4.329	2.810	0.145
8	4.415	1.076	0.056
9	4.493	4.107	0.213
10	4.560	25.286	1.309
11	4.837	1868.231	96.678
12	5.332	3.918	0.203
13	5.487	2.955	0.153
14	5.589	0.912	0.047
15	5.843	4.131	0.214
16	5.900	5.503	0.285
17	6.334	0.138	0.007
18	6.660	0.195	0.010
19	7.867	1.697	0.088
20	8.639	0.230	0.012

==

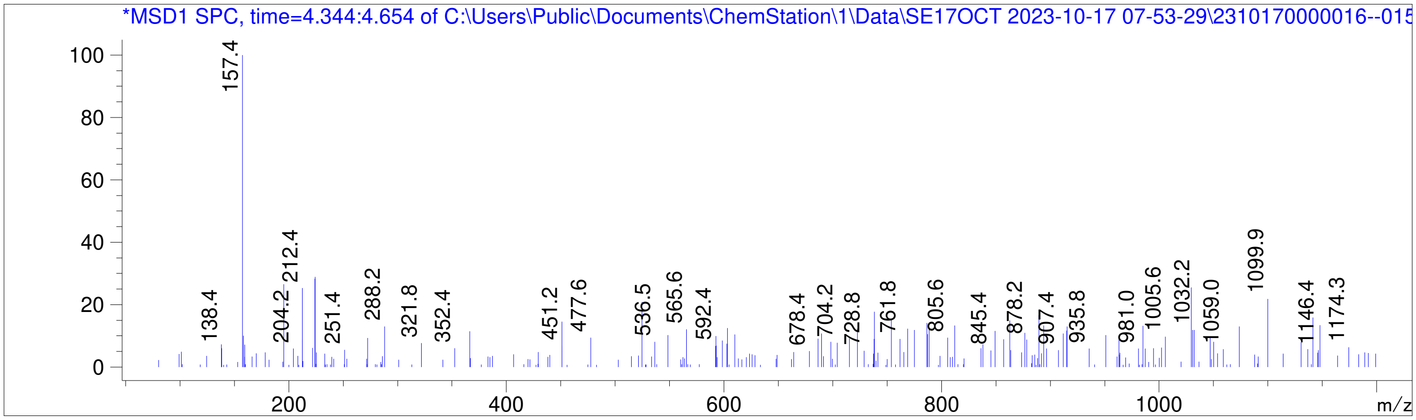
*MSD1 SPC, time=4.344:4.654 of C:\Users\Public\Documents\ChemStation\1\Data\SE17OCT 2023-10-17 07-53-29\2310170000016--015
157.4
138.4
204.2
212.4
251.4
288.2
321.8
352.4
451.2
477.6
536.5
565.6
592.4
678.4
704.2
728.8
761.8
805.6
845.4
878.2
907.4
935.8
981.0
1005.6
1032.2
1059.0
1099.9
1146.4
1174.3
m/z

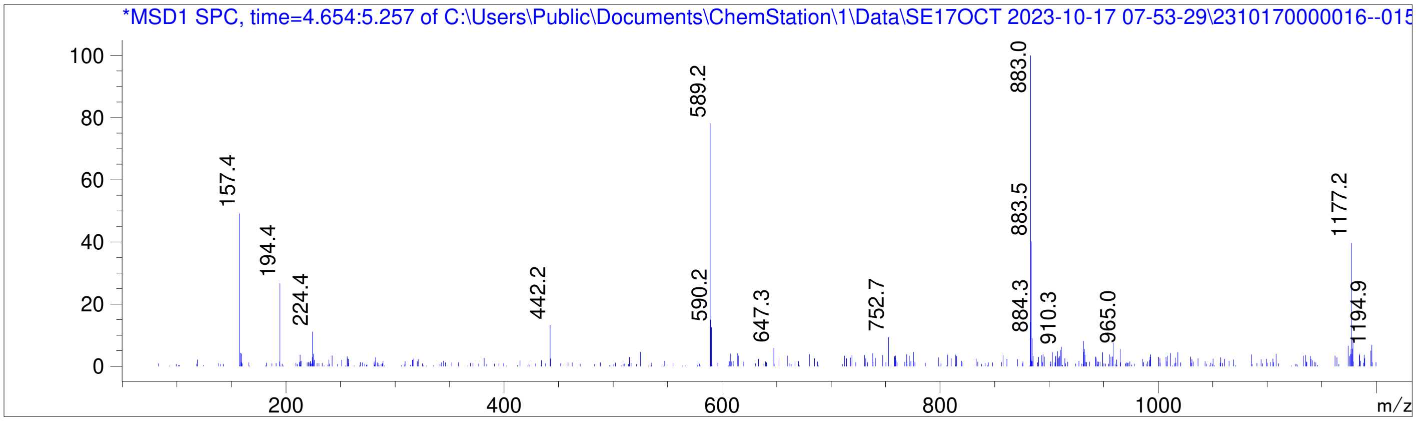
*MSD1 SPC, time=4.654:5.257 of C:\Users\Public\Documents\ChemStation\1\Data\SE17OCT 2023-10-17 07-53-29\2310170000016--015
157.4
194.4
224.4
442.2
589.2
590.2
647.3
752.7
883.0
883.5
884.3
910.3
965.0
1177.2
1194.9
m/z
