# Supplementary material for: Astrobiological implications of the stability and reactivity of peptide nucleic acid (PNA) in concentrated sulfuric acid
Source: Sci Adv. 2025 Mar 26;11(13):eadr0006. doi: 10.1126/sciadv.adr0006 (PMC11939054; doi:10.1126/sciadv.adr0006)
Supplement: Supplementary file 2 — Data S1 and S2 [file sciadv.adr0006_data_s1_and_s2.zip › Supplementary Dataset 1-LCMS DATA/LCMS PNA Hexamers A-T/LCMS G6 RT/1h/LCMS-6_CPT22010446-13-B.pdf]

Data -> C:\Users\Public\Documents\ChemStation\1\Data\09. September\SE26SEP\SE26SEP 202->
Sample-> CPT22010446-13-B

===

Injection Date :	Tue, 26. Sep. 2023	Seq Line :	12
		Location :	58
		Inj. Vol. :	4 µl

Acq. Method : C:\Users\Public\Documents\ChemStation\1\Data\SE26SEP 2023-09-26 10-10-32\22010446 LCMS-6.M

Analysis Method : C:\Users\Public\Documents\ChemStation\1\Data\09. September\ SE26SEP\SE26SEP 2023-09-26 10-10-32\22010446 LCMS-6.M (Sequence->

Waters XBridge Phenyl (4.6 * 150 mm; 3.5 µm); 0.05% TFA (aq) / AcN: 100/0 (0.0 min) --> (6.0 min) --> 70/30 (0.0 min) --> (2.0 min) --> 10/90 (2.0 min); Flow: 1.0 ml/min; MSD1 = positive; MSD2 = negative

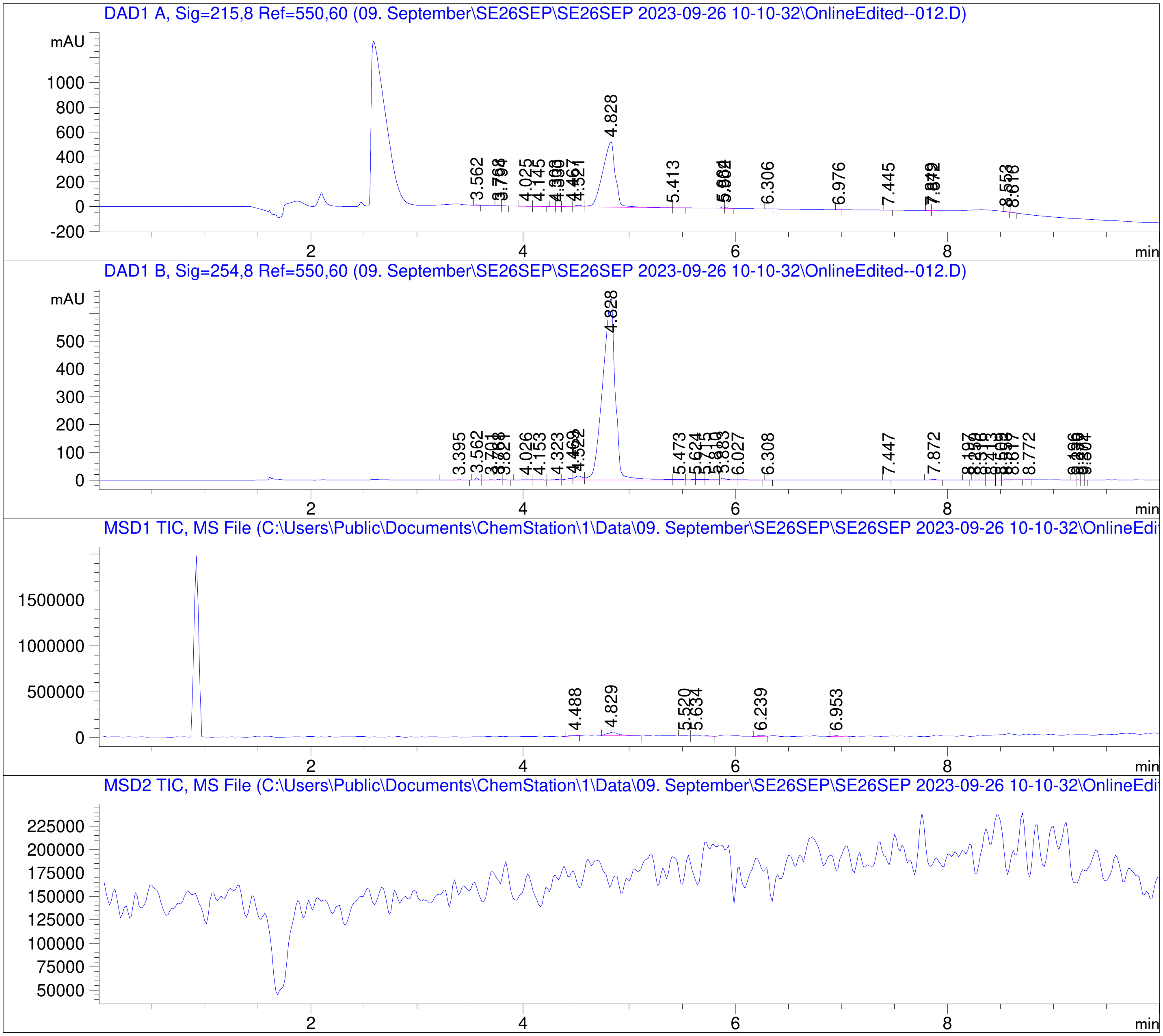

DAD1 A, Sig=215,8 Ref=550,60

Peak #	Ret. Time [min]	Area [mV *s]	Area %
1	3.562	8.173	0.181
2	3.768	5.269	0.117
3	3.794	2.573	0.057
4	4.025	5.464	0.121
5	4.145	6.401	0.142
6	4.303	2.179	0.048
7	4.330	3.411	0.076
8	4.467	16.700	0.370
9	4.521	56.974	1.262
10	4.828	4344.530	96.265
11	5.413	3.378	0.075
12	5.884	23.005	0.510
13	5.902	11.881	0.263
14	6.306	1.252	0.028
15	6.976	0.545	0.012
16	7.445	0.621	0.014
17	7.849	3.472	0.077
18	7.872	12.121	0.269
19	8.553	2.914	0.065
20	8.616	2.215	0.049

DAD1 B, Sig=254,8 Ref=550,60

Peak #	Ret. Time [min]	Area [mV *s]	Area %
1	3.395	5.493	0.098
2	3.562	13.150	0.236
3	3.701	4.693	0.084
4	3.768	6.374	0.114
5	3.821	3.157	0.057
6	4.026	7.006	0.126
7	4.153	7.587	0.136
8	4.323	8.474	0.152
9	4.469	24.808	0.445
10	4.522	71.088	1.274
11	4.828	5343.604	95.753
12	5.473	11.619	0.208
13	5.624	6.720	0.120
14	5.715	8.686	0.156
15	5.810	21.528	0.386
16	5.883	22.659	0.406
17	6.027	2.250	0.040
18	6.308	0.309	0.006
19	7.447	0.305	0.005
20	7.872	7.227	0.129
21	8.197	0.083	0.001
22	8.259	0.049	0.001
23	8.316	0.282	0.005
24	8.413	0.401	0.007
25	8.509	0.156	0.003
26	8.553	0.901	0.016
27	8.617	1.010	0.018
28	8.772	0.102	0.002

Peak #	Ret. Time [min]	Area [mV *s]	Area %
29	9.196	0.238	0.004
30	9.230	0.331	0.006
31	9.272	0.280	0.005
32	9.301	0.049	0.001

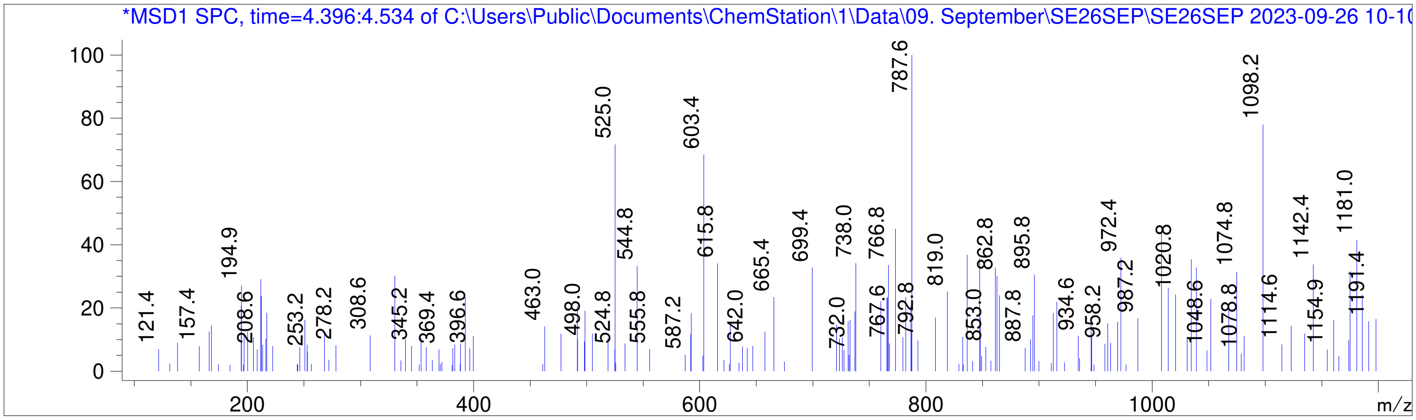
*MSD1 SPC, time=4.396:4.534 of C:\Users\Public\Documents\ChemStation\1\Data\09. September\SE26SEP\SE26SEP 2023-09-26 10-1
121.4
157.4
194.9
208.6
253.2
278.2
308.6
345.2
369.4
396.6
463.0
498.0
524.8
525.0
544.8
555.8
587.2
603.4
615.8
642.0
665.4
699.4
732.0
738.0
766.8
767.6
787.6
792.8
819.0
853.0
862.8
887.8
895.8
934.6
958.2
972.4
987.2
1020.8
1048.6
1074.8
1078.8
1098.2
1114.6
1142.4
1154.9
1181.0
1191.4
m/z

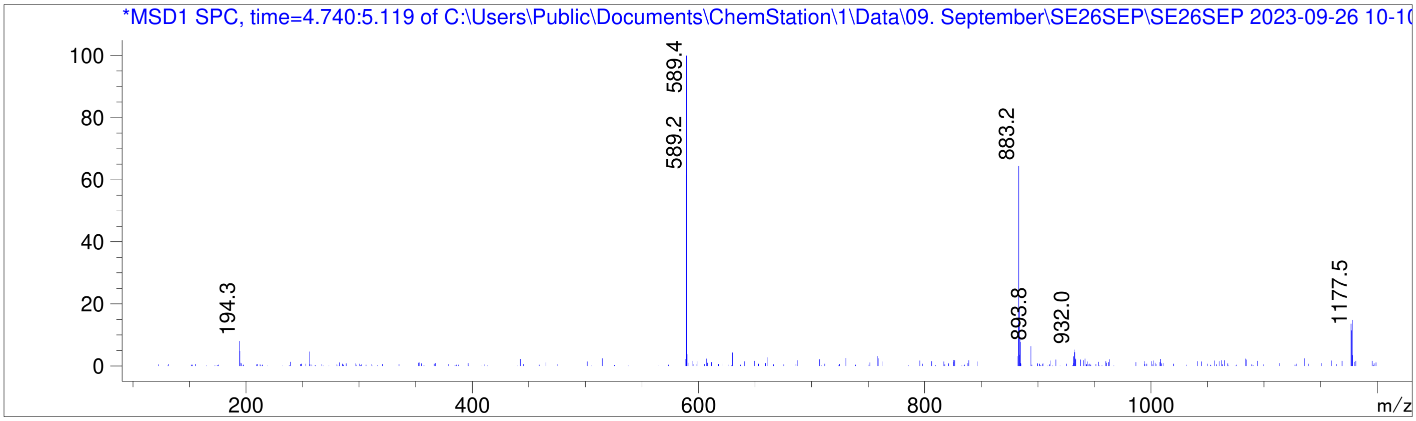
*MSD1 SPC, time=4.740:5.119 of C:\Users\Public\Documents\ChemStation\1\Data\09. September\SE26SEP\SE26SEP 2023-09-26 10-1
194.3
589.4
589.2
883.2
893.8
932.0
1177.5
m/z

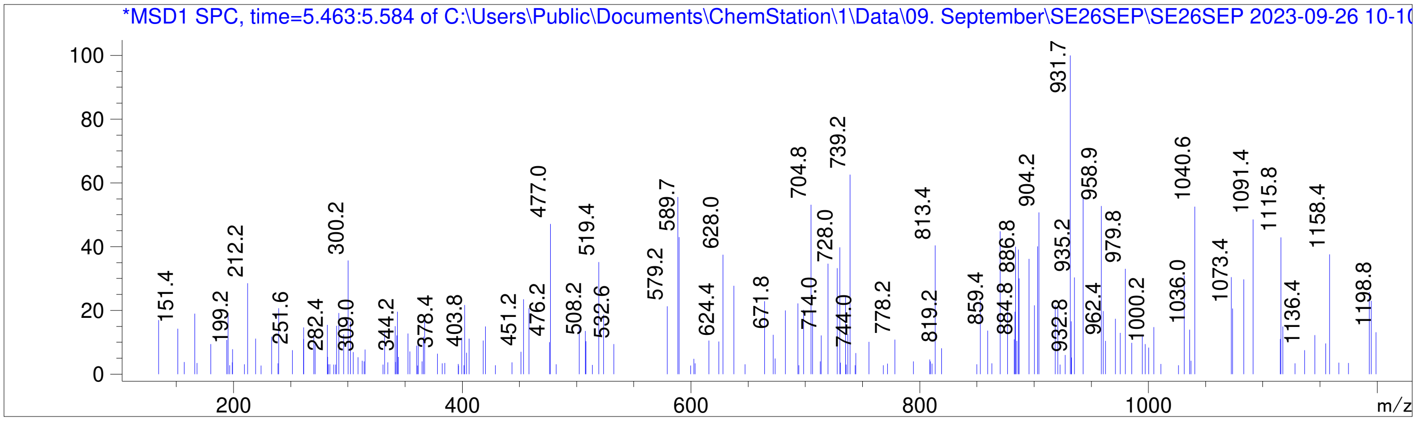
*MSD1 SPC, time=5.463:5.584 of C:\Users\Public\Documents\ChemStation\1\Data\09. September\SE26SEP\SE26SEP 2023-09-26 10-1
151.4
199.2
212.2
251.6
282.4
300.2
309.0
344.2
378.4
403.8
451.2
476.2
477.0
508.2
519.4
532.6
579.2
589.7
624.4
628.0
671.8
704.8
714.0
728.0
739.2
744.0
778.2
813.4
819.2
859.4
884.8
886.8
904.2
932.8
935.2
931.7
958.9
962.4
979.8
1000.2
1036.0
1040.6
1073.4
1091.4
1115.8
1136.4
1158.4
1198.8
m/z

===

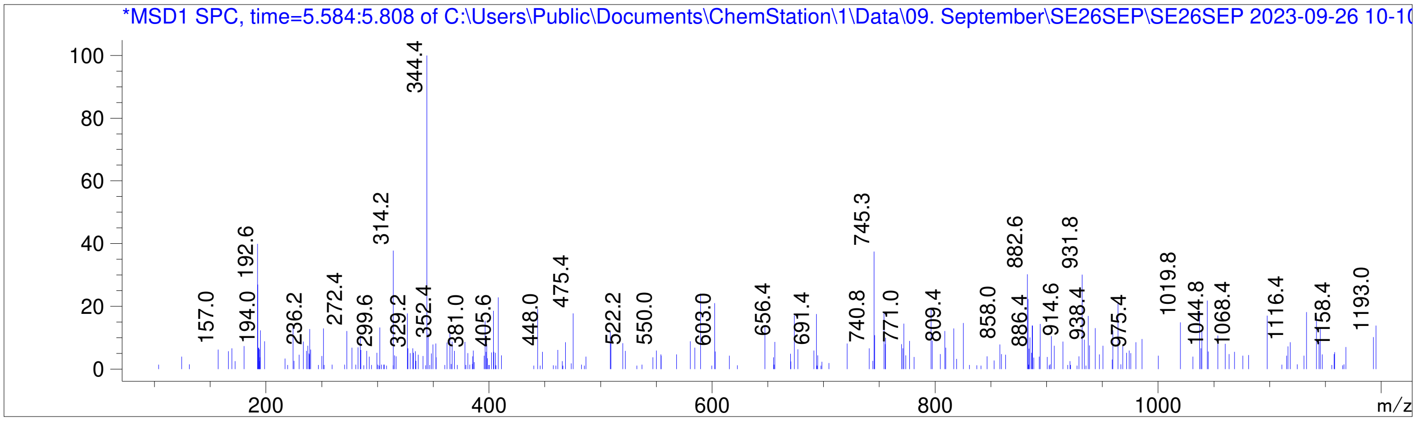
*MSD1 SPC, time=5.584:5.808 of C:\Users\Public\Documents\ChemStation\1\Data\09. September\SE26SEP\SE26SEP 2023-09-26 10-1
344.4
192.6
314.2
745.3
882.6
931.8
m/z

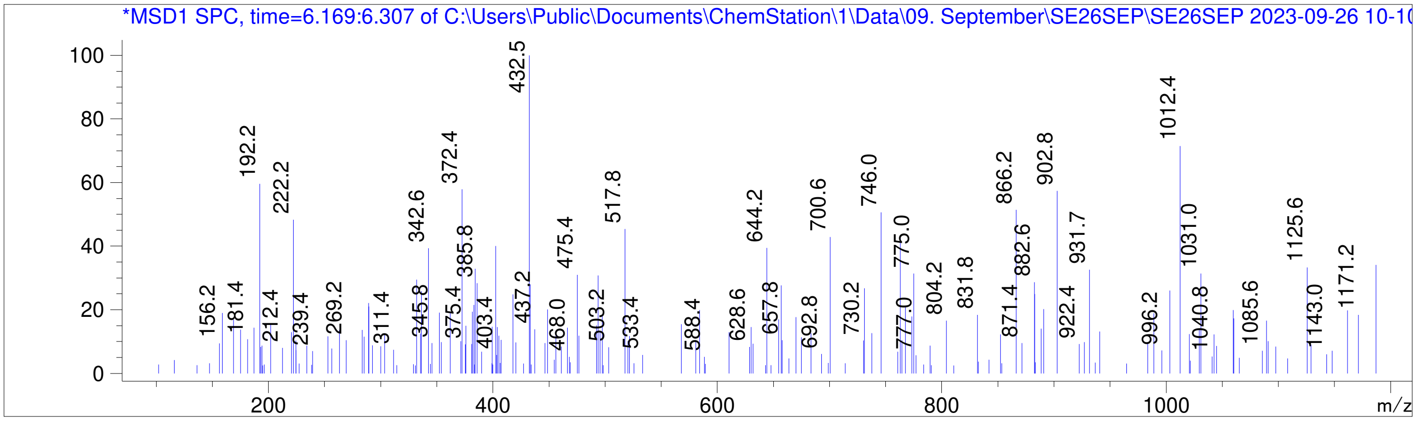
*MSD1 SPC, time=6.169:6.307 of C:\Users\Public\Documents\ChemStation\1\Data\09. September\SE26SEP\SE26SEP 2023-09-26 10-1
432.5
1012.4
192.2
902.8
372.4
866.2
m/z

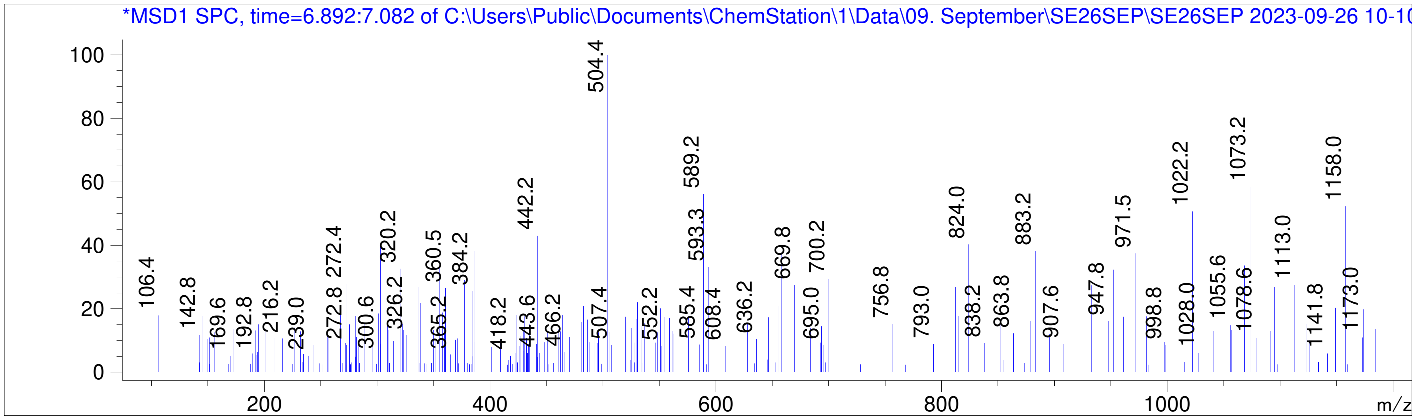
*MSD1 SPC, time=6.892:7.082 of C:\Users\Public\Documents\ChemStation\1\Data\09. September\SE26SEP\SE26SEP 2023-09-26 10-1
504.4
1073.2
1022.2
589.2
1158.0
442.2
m/z
