# Supplementary material for: Astrobiological implications of the stability and reactivity of peptide nucleic acid (PNA) in concentrated sulfuric acid
Source: Sci Adv. 2025 Mar 26;11(13):eadr0006. doi: 10.1126/sciadv.adr0006 (PMC11939054; doi:10.1126/sciadv.adr0006)

Data -> C:\Users\Public\Documents\ChemStation\1\Data\Se02Oct\SE02OCT 2023-10-02 11-39-->
Sample-> CPT22010446-13-B3

==

Injection Date : Mon, 2. Oct. 2023

Seq Line : 9
Location : 39
Inj. Vol. : 2 µl

Acq. Method : C:\Users\Public\Documents\ChemStation\1\Data\SE02OCT 2023-10-02 11-39-16\22010446 LCMS-6.M
Analysis Method : C:\Users\Public\Documents\ChemStation\1\Data\Se02Oct\SE02OCT 2023-10-02 11-39-16\22010446 LCMS-6.M (Sequence Method)
Waters XBridge Phenyl (4.6 * 150 mm; 3.5 µm); 0.05% TFA (aq) / AcN: 100/0 (0.0 min) --> (6.0 min) --> 70/30 (0.0 min) --> (2.0 min) --> 10/90 (2.0 min); Flow: 1.0 ml/min;
MSD1 = positive; MSD2 = negative

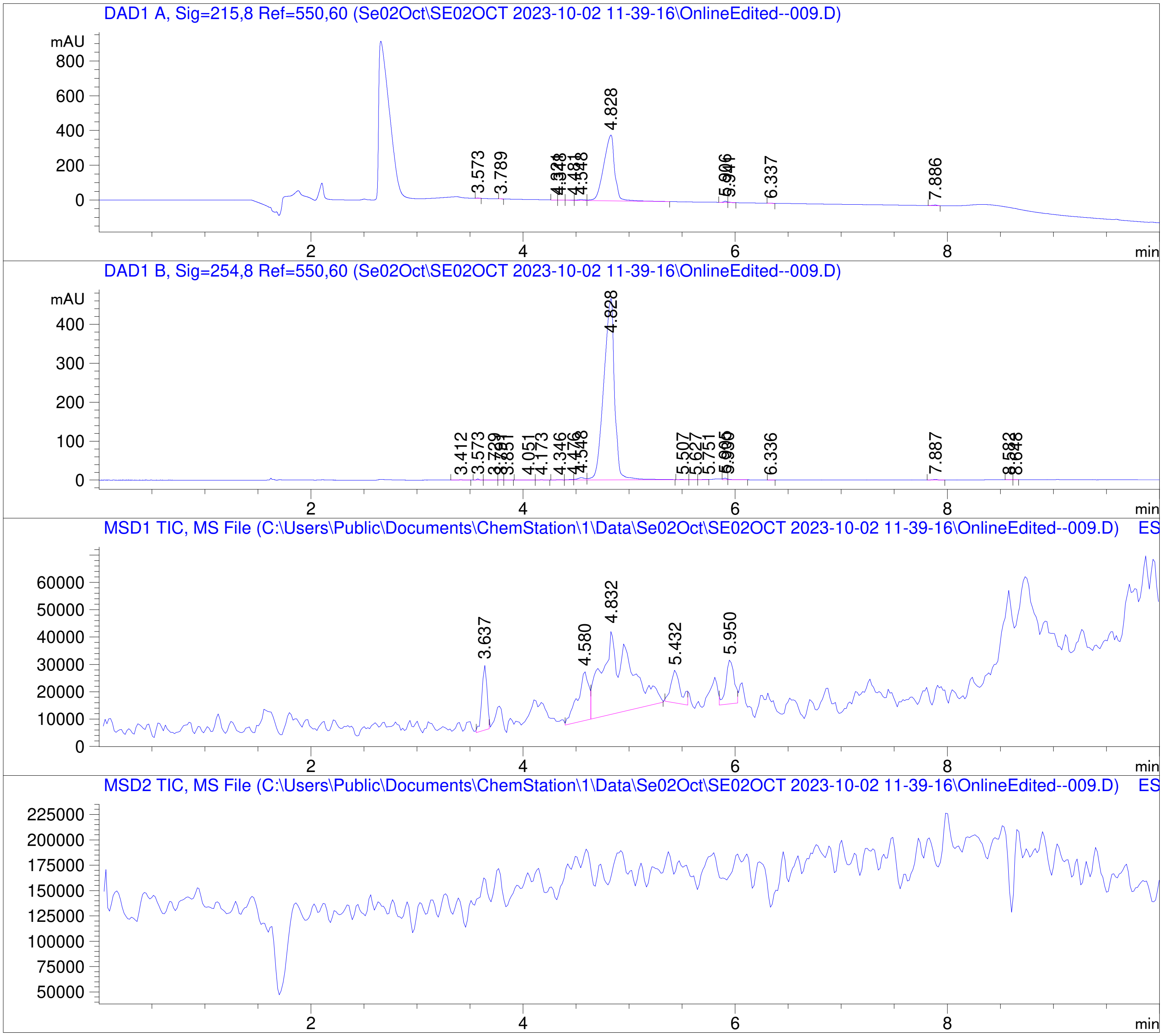

DAD1 A, Sig=215,8 Ref=550,60

Peak #	Ret. Time [min]	Area [mV *s]	Area %
1	3.573	2.570	0.093
2	3.789	0.778	0.028
3	4.321	1.199	0.044
4	4.348	1.917	0.070
5	4.481	3.695	0.134
6	4.548	22.069	0.802
7	4.828	2689.476	97.774
8	5.906	14.485	0.527
9	5.941	4.838	0.176
10	6.337	0.568	0.021
11	7.886	9.099	0.331

DAD1 B, Sig=254,8 Ref=550,60

Peak #	Ret. Time [min]	Area [mV *s]	Area %
1	3.412	2.249	0.067
2	3.573	4.212	0.126
3	3.729	1.640	0.049
4	3.791	1.748	0.052
5	3.851	1.263	0.038
6	4.051	2.702	0.081
7	4.173	2.379	0.071
8	4.346	2.556	0.076
9	4.476	4.808	0.144
10	4.548	27.739	0.830
11	4.828	3268.594	97.775
12	5.507	0.994	0.030
13	5.627	0.515	0.015
14	5.751	3.014	0.090
15	5.905	9.995	0.299
16	5.930	3.288	0.098
17	6.336	0.200	0.006
18	7.887	4.380	0.131
19	8.582	0.386	0.012
20	8.648	0.325	0.010

Data -> C:\Users\Public\Documents\ChemStation\1\Data\Se02Oct\SE02OCT 2023-10-02 11-39-->
Sample-> CPT22010446-13-B3

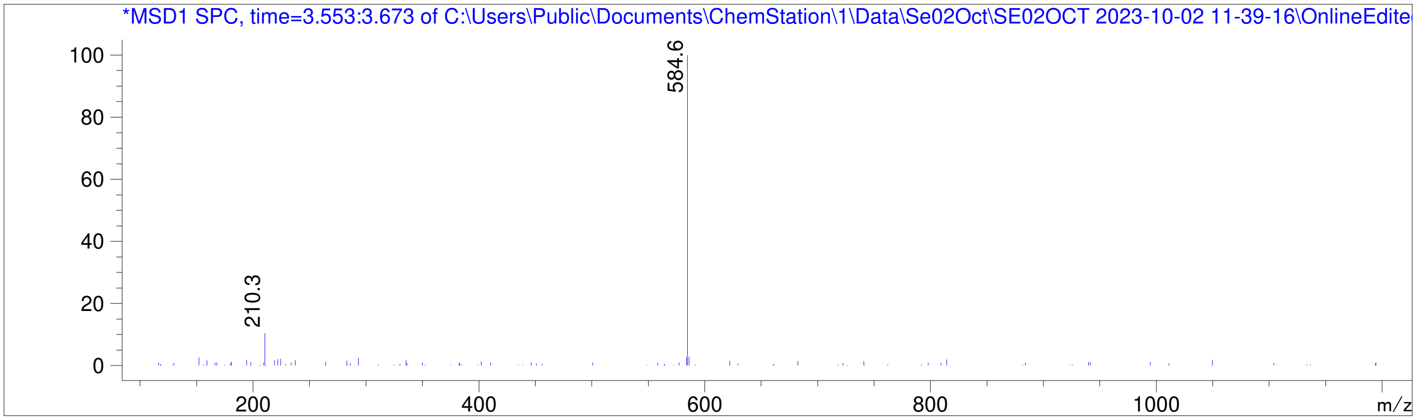

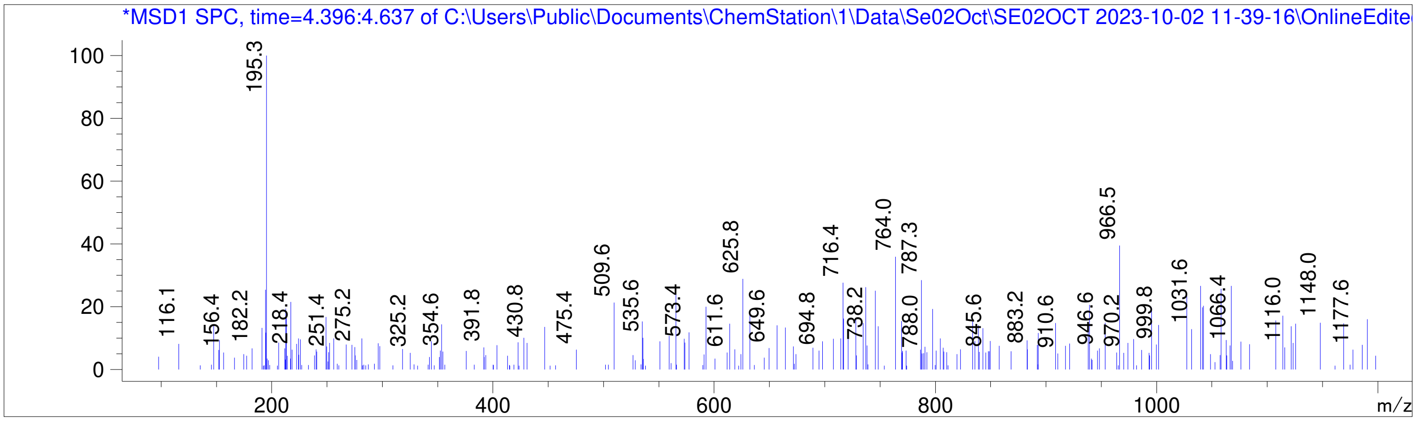

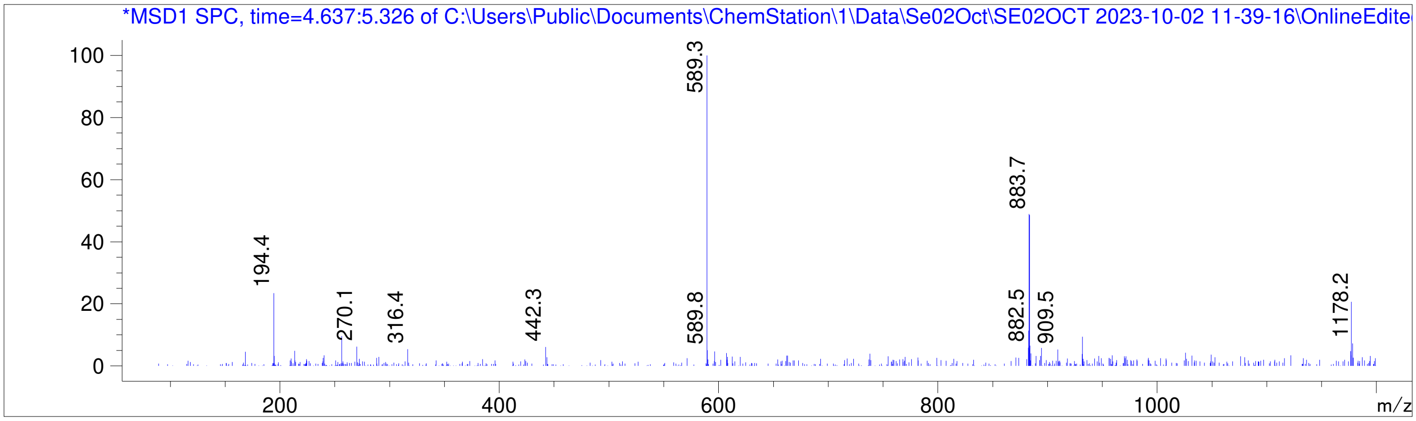

Data -> C:\Users\Public\Documents\ChemStation\1\Data\Se02Oct\SE02OCT 2023-10-02 11-39-->
Sample-> CPT22010446-13-B3

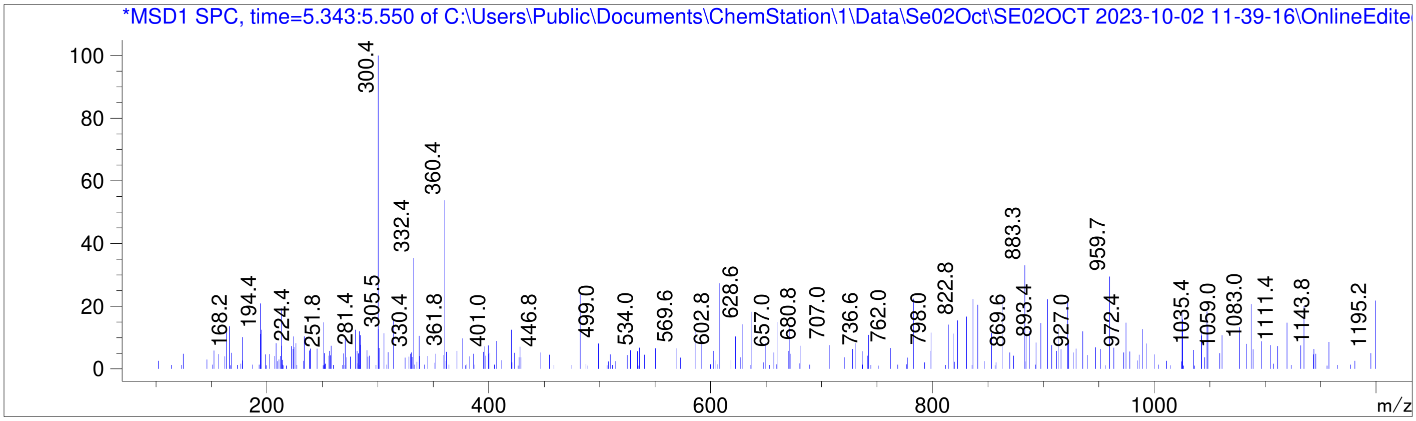

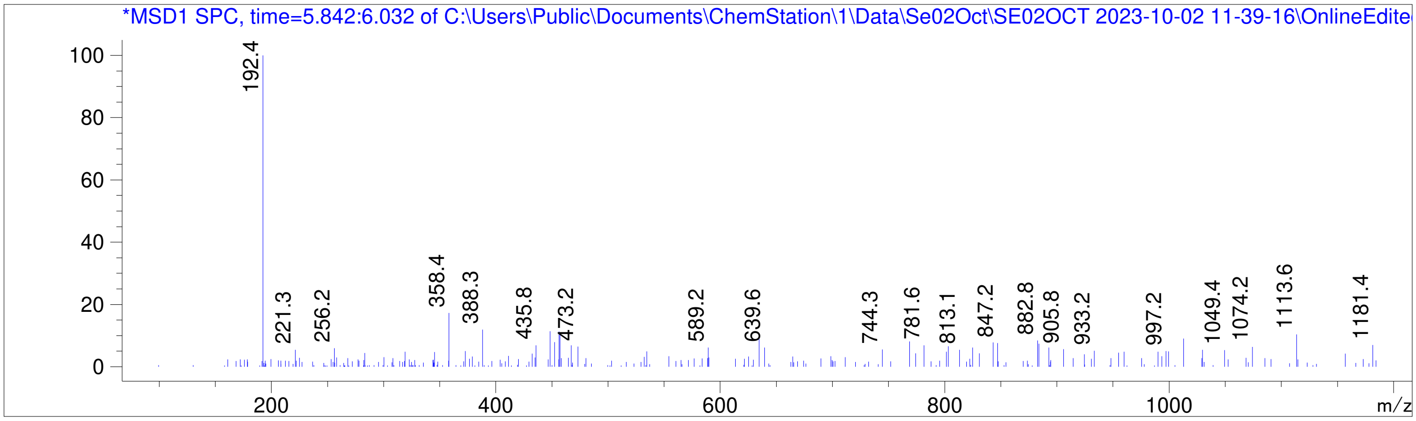

Supplement: Supplementary file 2 — Data S1 and S2 [file sciadv.adr0006_data_s1_and_s2.zip › Supplementary Dataset 1-LCMS DATA/LCMS PNA Hexamers A-T/LCMS G6 RT/1h/LCMS-6_CPT22010446-13-B3.pdf]
