# Supplementary material for: Astrobiological implications of the stability and reactivity of peptide nucleic acid (PNA) in concentrated sulfuric acid
Source: Sci Adv. 2025 Mar 26;11(13):eadr0006. doi: 10.1126/sciadv.adr0006 (PMC11939054; doi:10.1126/sciadv.adr0006)

===

Injection Date : Tue, 26. Sep. 2023	Seq Line	:	21
	Location	:	68
	Inj. Vol.	:	4 µl

Acq. Method : C:\Users\Public\Documents\ChemStation\1\Data\SE26SEP 2023-09-26 16-24-18\22010446 LCMS-6.M

Analysis Method : C:\Users\Public\Documents\ChemStation\1\Data\09. September\SE26SEP\SE26SEP 2023-09-26 16-24-18\22010446 LCMS-6.M (Sequence->

Waters XBridge Phenyl (4.6 * 150 mm; 3.5 µm); 0.05% TFA (aq) / AcN: 100/0 (0.0 min) --> (6.0 min) --> 70/30 (0.0 min) --> (2.0 min) --> 10/90 (2.0 min); Flow: 1.0 ml/min; MSD1 = positive; MSD2 = negative

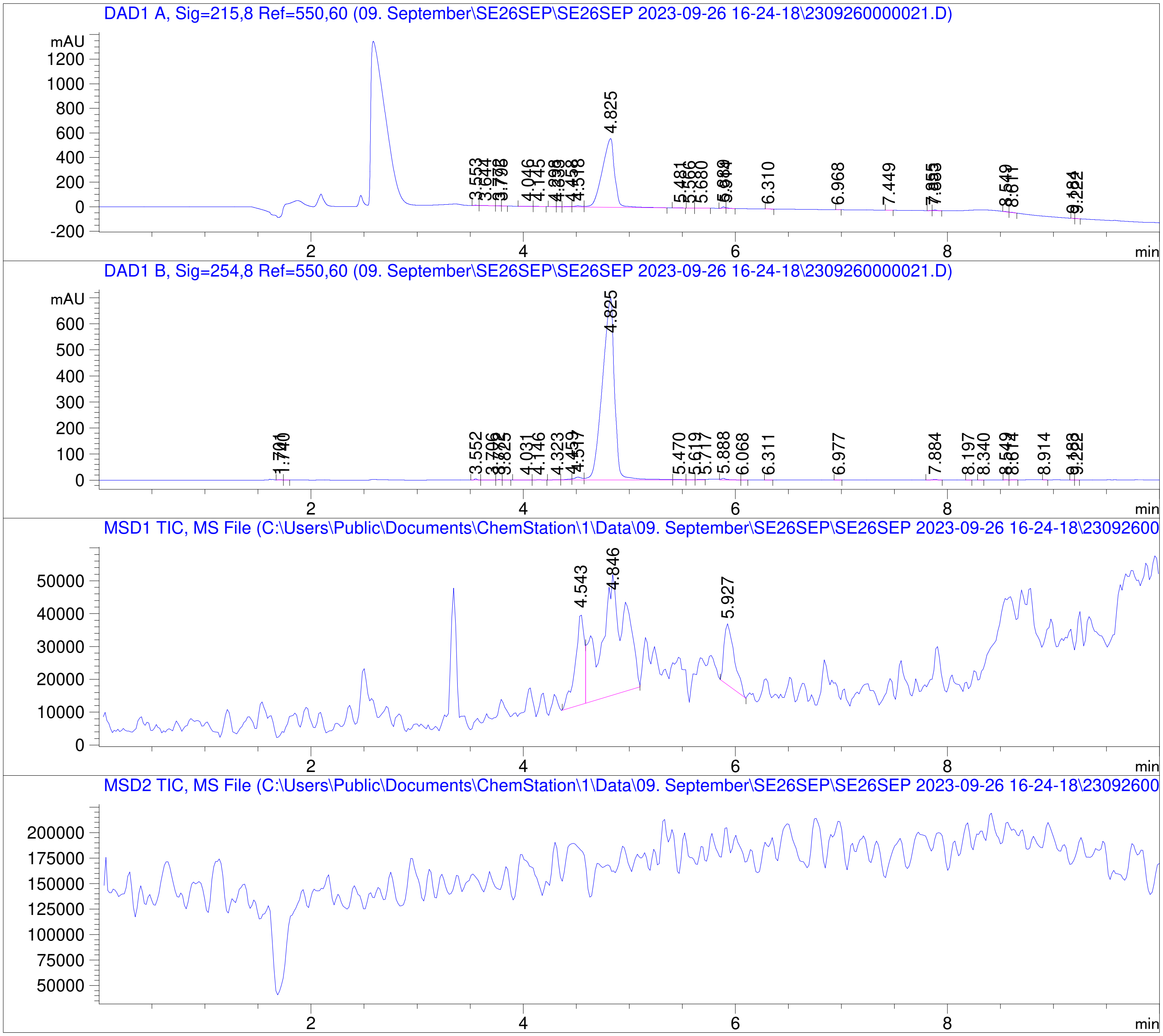

DAD1 A, Sig=215,8 Ref=550,60

Peak #	Ret. Time [min]	Area [mV *s]	Area %
1	3.553	6.695	0.145
2	3.644	7.578	0.164
3	3.772	3.938	0.085
4	3.795	1.557	0.034
5	4.046	2.658	0.057
6	4.145	3.016	0.065
7	4.298	2.084	0.045
8	4.333	2.074	0.045
9	4.458	8.381	0.181
10	4.518	40.585	0.878
11	4.825	4479.733	96.907
12	5.481	1.859	0.040
13	5.566	0.546	0.012
14	5.680	3.125	0.068
15	5.889	24.159	0.523
16	5.914	8.098	0.175
17	6.310	1.246	0.027
18	6.968	0.319	0.007
19	7.449	0.694	0.015
20	7.855	2.421	0.052
21	7.883	12.931	0.280
22	8.549	3.402	0.074
23	8.611	3.908	0.085
24	9.184	0.597	0.013
25	9.222	1.112	0.024

DAD1 B, Sig=254,8 Ref=550,60

Peak #	Ret. Time [min]	Area [mV *s]	Area %
1	1.701	4.879	0.085
2	1.740	0.383	0.007
3	3.552	8.593	0.151
4	3.706	3.113	0.055
5	3.772	4.097	0.072
6	3.825	2.132	0.037
7	4.031	4.868	0.085
8	4.146	4.543	0.080
9	4.323	5.451	0.095
10	4.459	14.908	0.261
11	4.517	53.905	0.944
12	4.825	5546.279	97.147
13	5.470	10.339	0.181
14	5.619	5.255	0.092
15	5.717	9.269	0.162
16	5.888	20.589	0.361
17	6.068	0.318	0.006
18	6.311	0.275	0.005
19	6.977	0.108	0.002
20	7.884	7.318	0.128
21	8.197	0.139	0.002
22	8.340	0.103	0.002
23	8.549	0.675	0.012

Peak #	Ret. Time [min]	Area [mV *s]	Area %
24	8.614	0.747	0.013
25	8.914	0.296	0.005
26	9.183	0.212	0.004
27	9.222	0.372	0.007

Data -> C:\Users\Public\Documents\ChemStation\1\Data\09. September\SE26SEP\SE26SEP 202->
Sample-> CPT22010446-20-A2
==

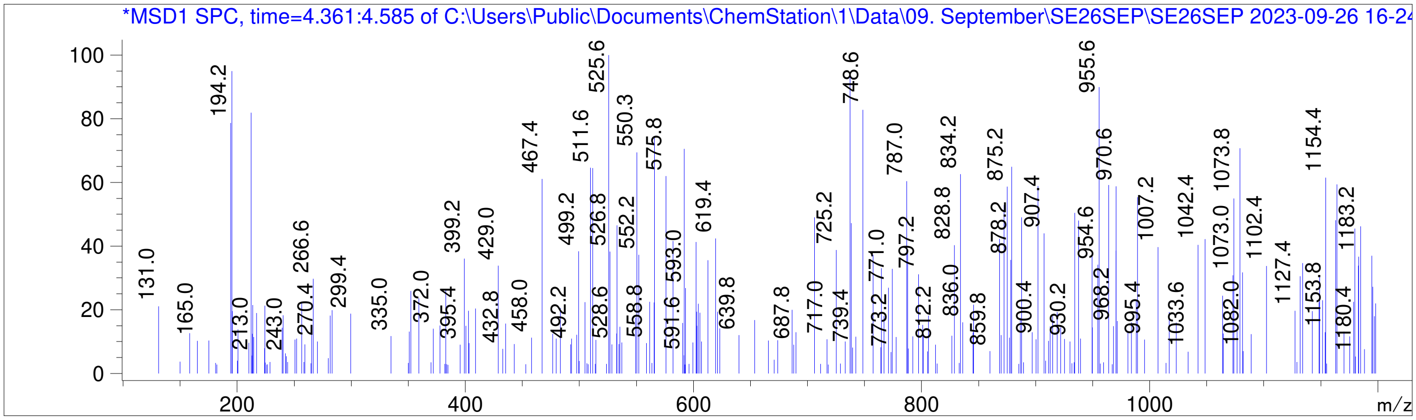

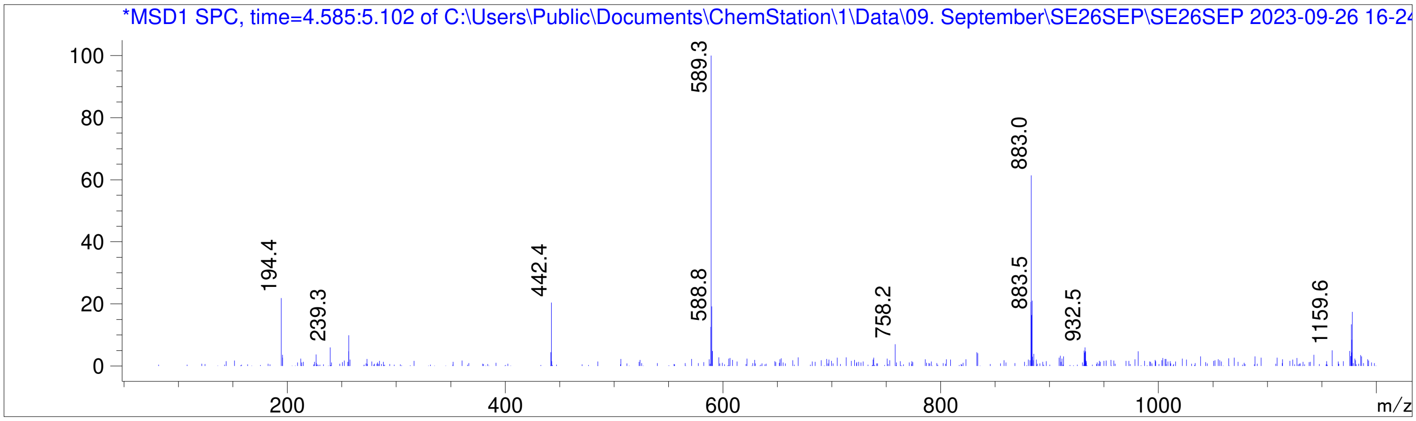

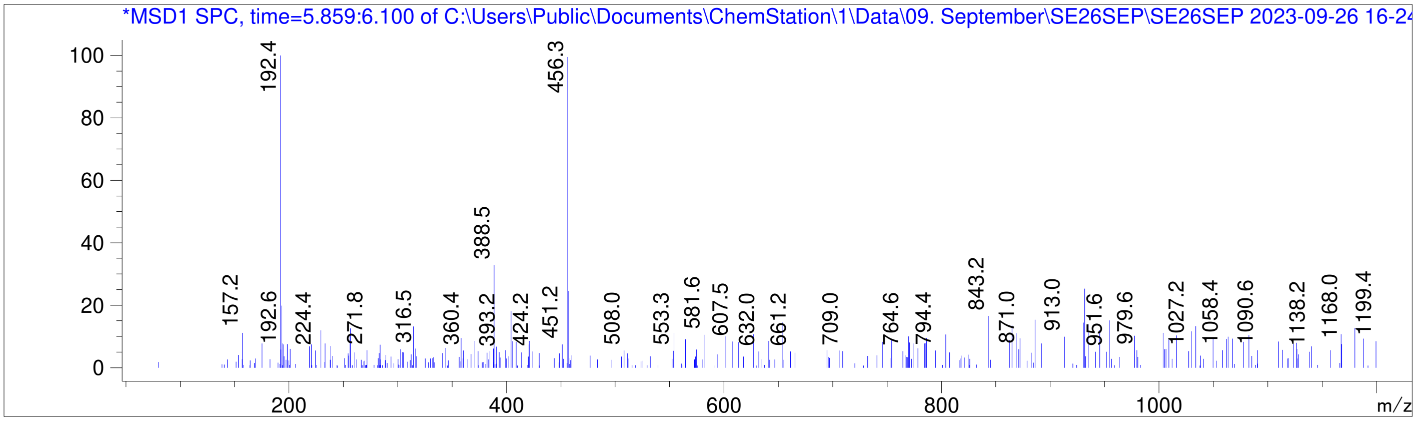

Supplement: Supplementary file 2 — Data S1 and S2 [file sciadv.adr0006_data_s1_and_s2.zip › Supplementary Dataset 1-LCMS DATA/LCMS PNA Hexamers A-T/LCMS G6 RT/1h/LCMS-6_CPT22010446-20-A-2.pdf]
