# Supplementary material for: Astrobiological implications of the stability and reactivity of peptide nucleic acid (PNA) in concentrated sulfuric acid
Source: Sci Adv. 2025 Mar 26;11(13):eadr0006. doi: 10.1126/sciadv.adr0006 (PMC11939054; doi:10.1126/sciadv.adr0006)

===

Injection Date	:	Tue, 3. Oct. 2023	Seq Line :	6
			Location :	62
			Inj. Vol. :	2 µl

Acq. Method : C:\Users\Public\Documents\ChemStation\1\Data\SE03OCT 2023-10-03 13-55-25\22010446 LCMS-6.M

Analysis Method : C:\Users\Public\Documents\ChemStation\1\Data\Se03Oct\SE03OCT 2023-10-03 13-55-25\22010446 LCMS-6.M (Sequence Method)

Waters XBridge Phenyl (4.6 * 150 mm; 3.5 µm); 0.05% TFA (aq) / AcN: 100/0 (0.0 min) --> (6.0 min) --> 70/30 (0.0 min) --> (2.0 min) --> 10/90 (2.0 min); Flow: 1.0 ml/min; MSD1 = positive; MSD2 = negative

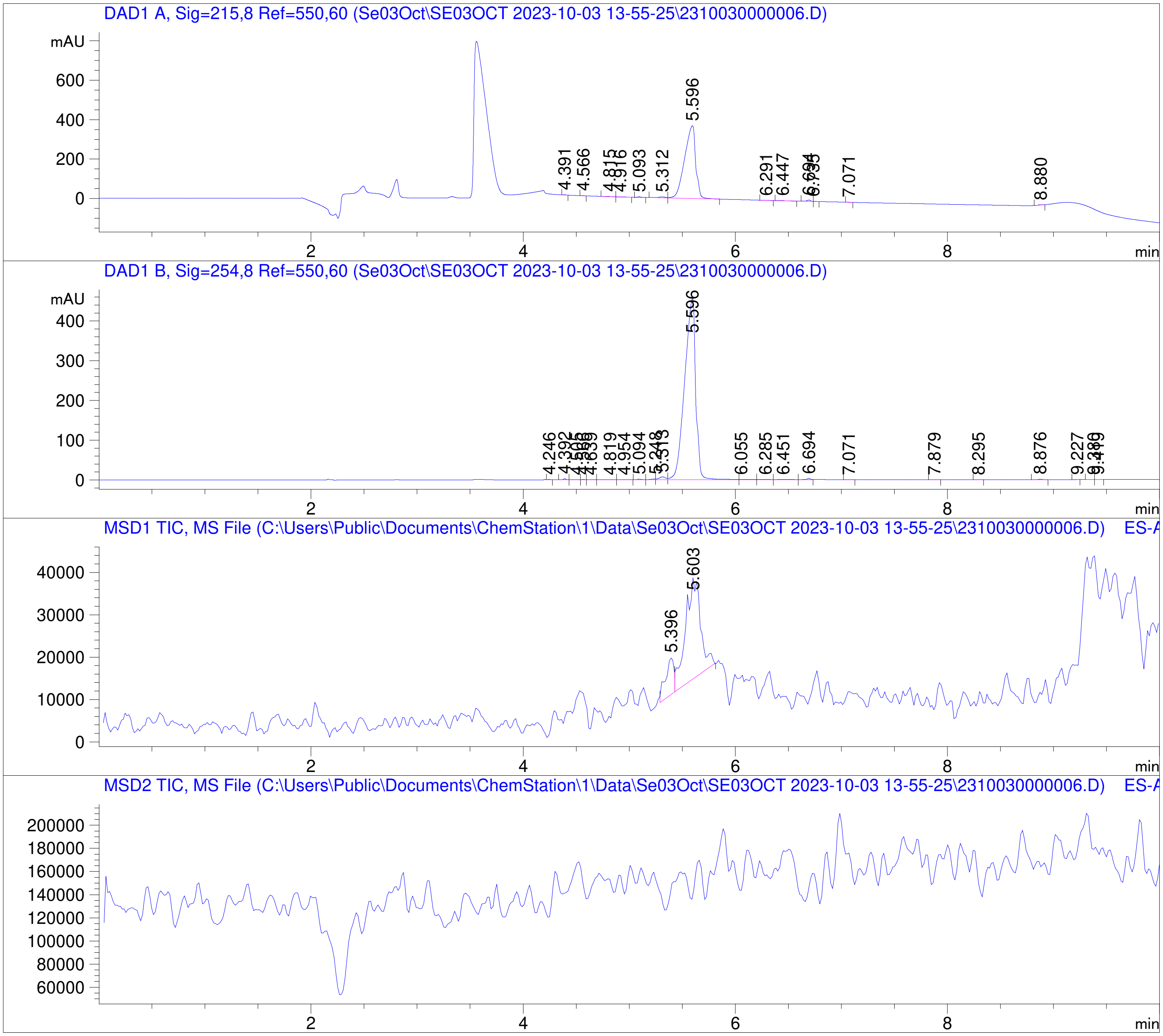

DAD1 A, Sig=215,8 Ref=550,60

Peak #	Ret. Time [min]	Area [mV *s]	Area %
1	4.391	2.342	0.085
2	4.566	1.013	0.037
3	4.815	2.705	0.099
4	4.916	2.603	0.095
5	5.093	6.945	0.253
6	5.312	23.487	0.856
7	5.596	2676.313	97.585
8	6.291	0.832	0.030
9	6.447	3.294	0.120
10	6.694	15.913	0.580
11	6.735	0.925	0.034
12	7.071	0.723	0.026
13	8.880	5.455	0.199

DAD1 B, Sig=254,8 Ref=550,60

Peak #	Ret. Time [min]	Area [mV *s]	Area %
1	4.246	0.902	0.026
2	4.392	4.757	0.139
3	4.505	1.777	0.052
4	4.566	1.941	0.057
5	4.639	1.610	0.047
6	4.819	2.537	0.074
7	4.954	2.587	0.076
8	5.094	4.929	0.145
9	5.248	6.421	0.188
10	5.313	32.518	0.953
11	5.596	3314.659	97.173
12	6.055	7.774	0.228
13	6.285	5.453	0.160
14	6.451	7.997	0.234
15	6.694	9.263	0.272
16	7.071	0.276	0.008
17	7.879	0.200	0.006
18	8.295	0.223	0.007
19	8.876	3.879	0.114
20	9.227	0.188	0.006
21	9.380	0.577	0.017
22	9.419	0.639	0.019

Data -> C:\Users\Public\Documents\ChemStation\1\Data\Se03Oct\SE03OCT 2023-10-03 13-55-->
Sample-> CPT22010446-13-B3-24h
==

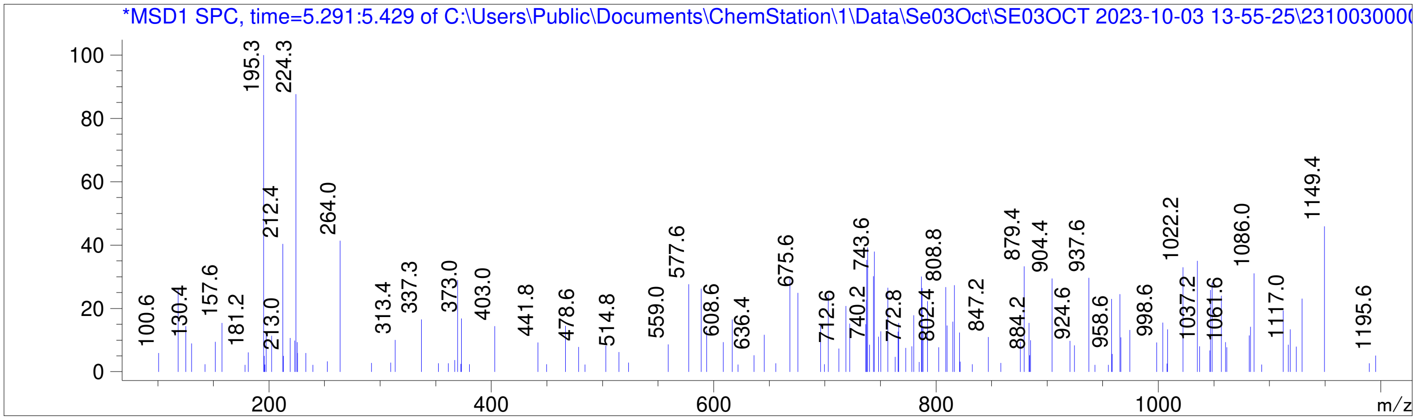

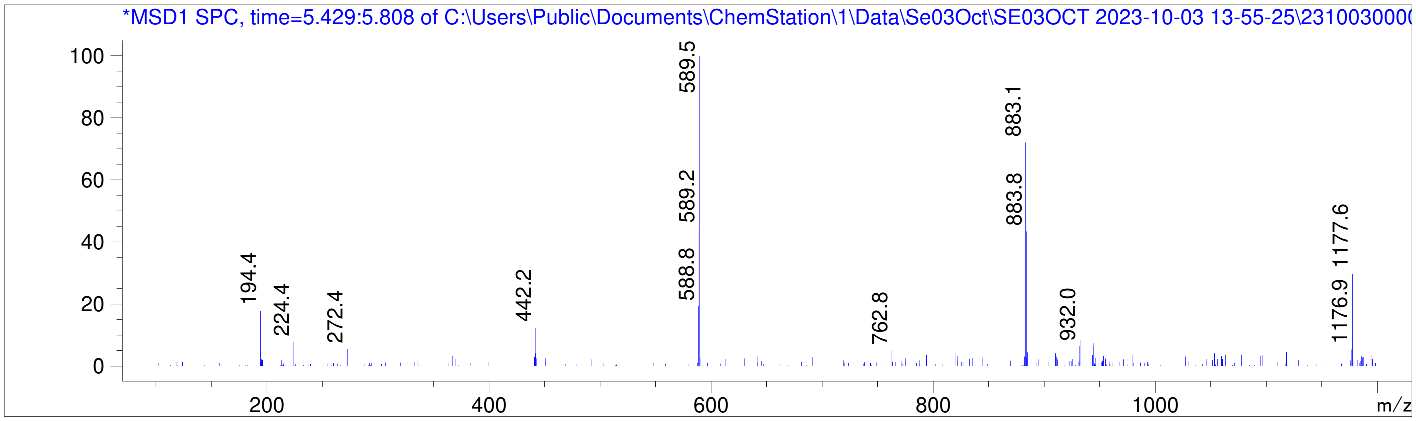

Supplement: Supplementary file 2 — Data S1 and S2 [file sciadv.adr0006_data_s1_and_s2.zip › Supplementary Dataset 1-LCMS DATA/LCMS PNA Hexamers A-T/LCMS G6 RT/24h/LCMS-6_CPT22010446-13-B3-24h.pdf]
