# Supplementary material for: Astrobiological implications of the stability and reactivity of peptide nucleic acid (PNA) in concentrated sulfuric acid
Source: Sci Adv. 2025 Mar 26;11(13):eadr0006. doi: 10.1126/sciadv.adr0006 (PMC11939054; doi:10.1126/sciadv.adr0006)

Data -> C:\USERS\PUBLIC\DOCUMENTS\CHEMSTATION\1\DATA\SE27SEP 2023-09-27 12-47-15\ ->
Sample-> CPT22010446-20-A2-24h

=====================================================================

| | | | | | |
|---|---|---|---|---|---|
| Injection Date | : | Wed, 27. Sep. 2023 | Seq Line | : | 6 |
| | | | Location | : | 5 |
| | | | Inj. Vol. | : | 2 µl |

Acq. Method : C:\Users\Public\Documents\ChemStation\1\Data\SE27SEP 2023-09-27 12-47-15\22010446 LCMS-6.M
Analysis Method : C:\Users\Public\Documents\ChemStation\1\Data\SE27SEP 2023-09-27 12-47-15\22010446 LCMS-6.M (Sequence Method)
Waters XBridge Phenyl (4.6 * 150 mm; 3.5 µm); 0.05% TFA (aq) / AcN: 100/0 (0.0 min) --> (6.0 min) --> 70/30 (0.0 min) --> (2.0 min) --> 10/90 (2.0 min); Flow: 1.0 ml/min;
MSD1 = positive; MSD2 = negative

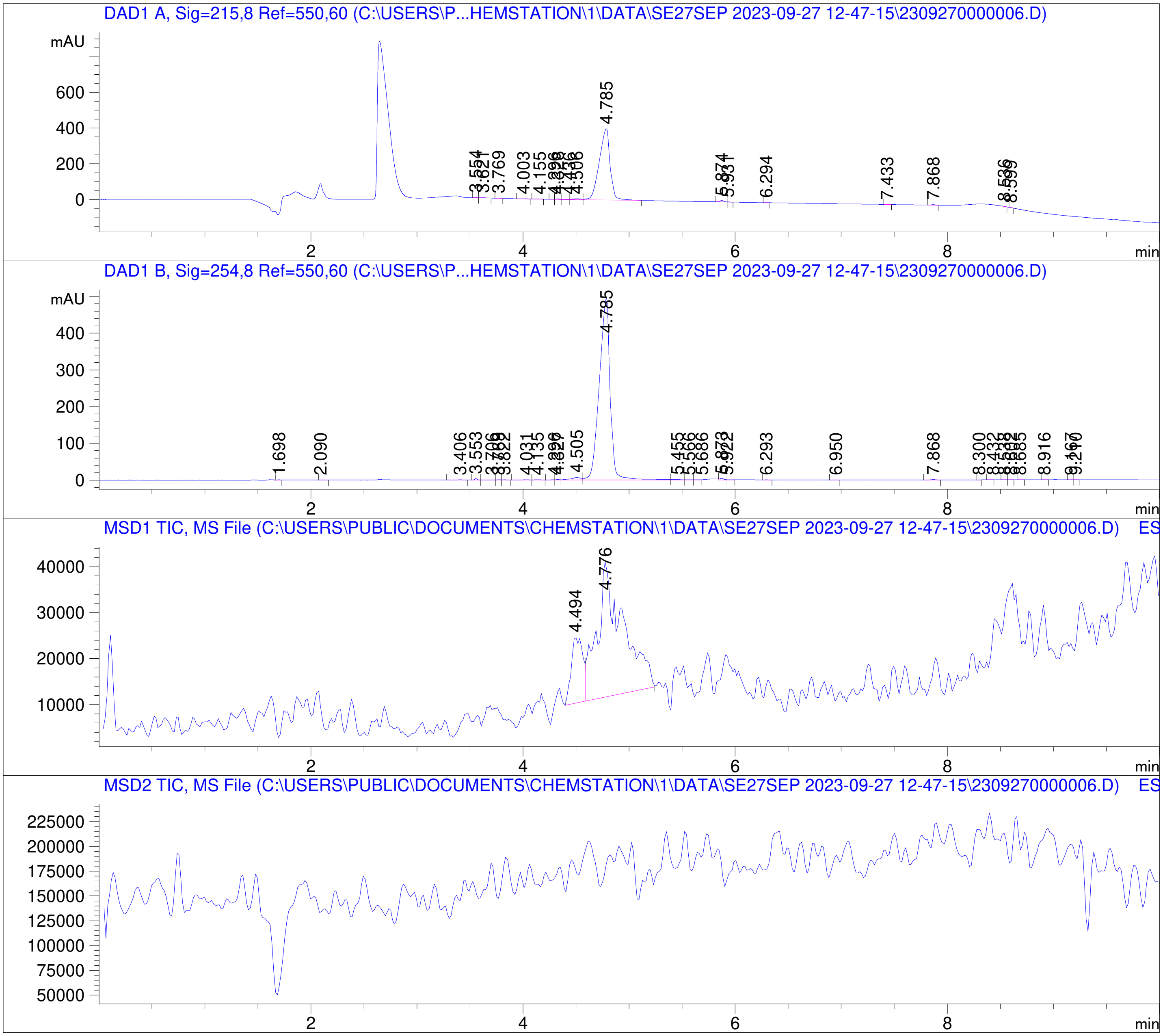

DAD1 A, Sig=215,8 Ref=550,60

| Peak # | Ret. Time [min] | Area [mV *s] | Area % |
|---|---|---|---|
| 1 | 3.554 | 5.480 | 0.190 |
| 2 | 3.621 | 4.890 | 0.169 |
| 3 | 3.769 | 1.973 | 0.068 |
| 4 | 4.003 | 1.759 | 0.061 |
| 5 | 4.155 | 2.140 | 0.074 |
| 6 | 4.296 | 0.760 | 0.026 |
| 7 | 4.328 | 5.623 | 0.195 |
| 8 | 4.436 | 3.827 | 0.132 |
| 9 | 4.506 | 24.702 | 0.855 |
| 10 | 4.785 | 2809.304 | 97.196 |
| 11 | 5.874 | 18.574 | 0.643 |
| 12 | 5.931 | 0.640 | 0.022 |
| 13 | 6.294 | 0.573 | 0.020 |
| 14 | 7.433 | 0.590 | 0.020 |
| 15 | 7.868 | 7.349 | 0.254 |
| 16 | 8.536 | 1.042 | 0.036 |
| 17 | 8.599 | 1.138 | 0.039 |

DAD1 B, Sig=254,8 Ref=550,60

| Peak # | Ret. Time [min] | Area [mV *s] | Area % |
|---|---|---|---|
| 1 | 1.698 | 1.596 | 0.044 |
| 2 | 2.090 | 1.502 | 0.042 |
| 3 | 3.406 | 2.480 | 0.069 |
| 4 | 3.553 | 6.408 | 0.177 |
| 5 | 3.706 | 2.134 | 0.059 |
| 6 | 3.769 | 2.118 | 0.059 |
| 7 | 3.822 | 1.509 | 0.042 |
| 8 | 4.031 | 3.227 | 0.089 |
| 9 | 4.135 | 2.696 | 0.075 |
| 10 | 4.296 | 1.618 | 0.045 |
| 11 | 4.327 | 3.803 | 0.105 |
| 12 | 4.505 | 44.916 | 1.242 |
| 13 | 4.785 | 3509.415 | 97.015 |
| 14 | 5.455 | 7.229 | 0.200 |
| 15 | 5.566 | 3.134 | 0.087 |
| 16 | 5.686 | 3.461 | 0.096 |
| 17 | 5.873 | 11.799 | 0.326 |
| 18 | 5.922 | 2.043 | 0.056 |
| 19 | 6.293 | 0.237 | 0.007 |
| 20 | 6.950 | 0.141 | 0.004 |
| 21 | 7.868 | 3.735 | 0.103 |
| 22 | 8.300 | 0.055 | 0.002 |
| 23 | 8.432 | 0.122 | 0.003 |
| 24 | 8.538 | 0.417 | 0.012 |
| 25 | 8.602 | 0.504 | 0.014 |
| 26 | 8.685 | 0.160 | 0.004 |
| 27 | 8.916 | 0.587 | 0.016 |
| 28 | 9.167 | 0.134 | 0.004 |
| 29 | 9.210 | 0.196 | 0.005 |

Sample-> CPT22010446-20-A2-24h

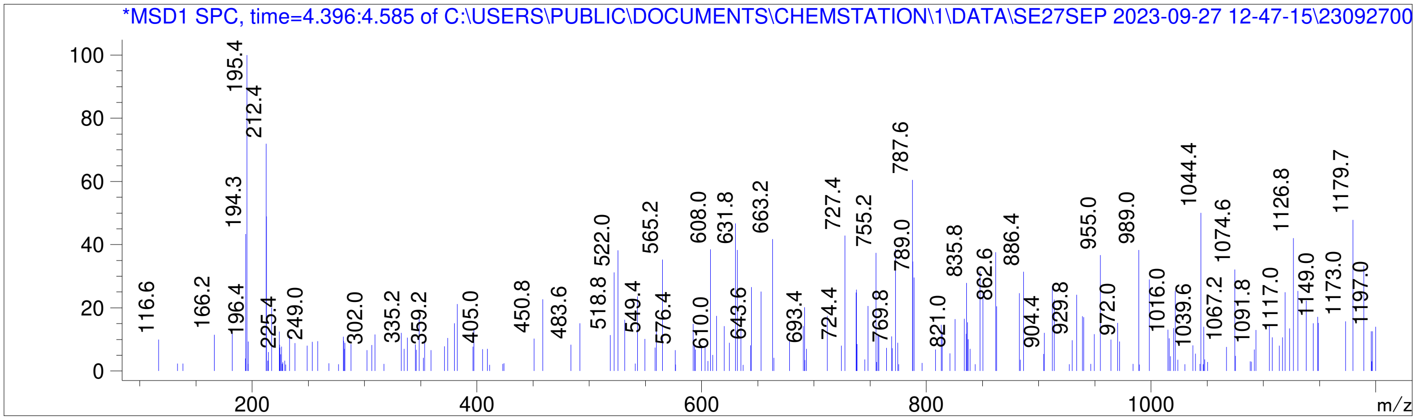


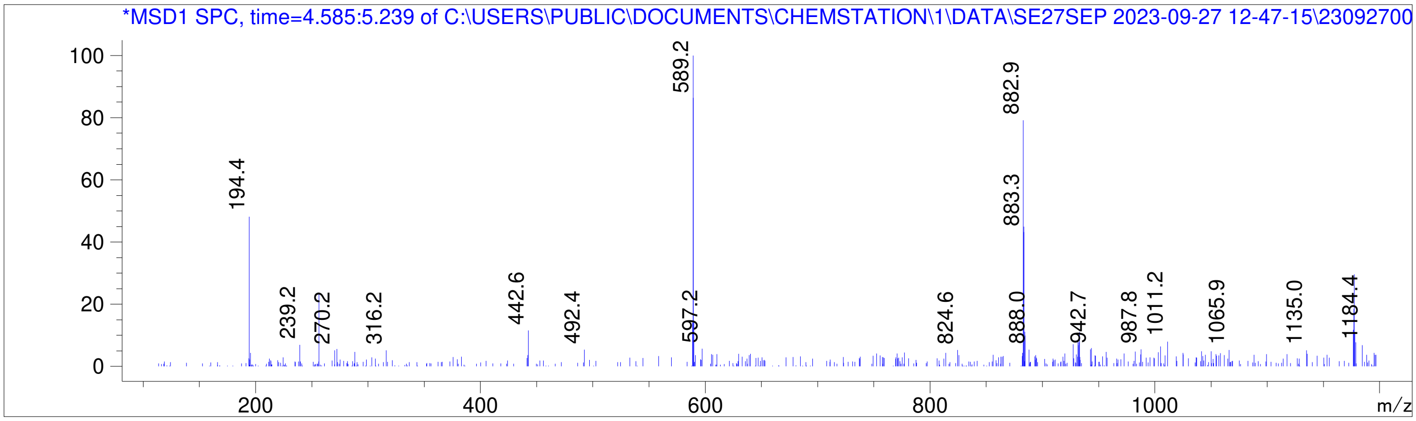

Supplement: Supplementary file 2 — Data S1 and S2 [file sciadv.adr0006_data_s1_and_s2.zip › Supplementary Dataset 1-LCMS DATA/LCMS PNA Hexamers A-T/LCMS G6 RT/24h/LCMS-6_CPT22010446-20-A2-24h.pdf]
