# Supplementary material for: Astrobiological implications of the stability and reactivity of peptide nucleic acid (PNA) in concentrated sulfuric acid
Source: Sci Adv. 2025 Mar 26;11(13):eadr0006. doi: 10.1126/sciadv.adr0006 (PMC11939054; doi:10.1126/sciadv.adr0006)
Supplement: Supplementary file 2 — Data S1 and S2 [file sciadv.adr0006_data_s1_and_s2.zip › Supplementary Dataset 1-LCMS DATA/LCMS PNA Hexamers A-T/LCMS G6 RT/24h/LCMS-6_CPT22010446-20-A-24h.pdf]

Data -> C:\Users\Public\Documents\ChemStation\1\Data\09. September\SE26SEP\SE26SEP 202->
Sample-> CPT22010446-20-A-24h

===

Injection Date	: Tue, 26. Sep. 2023	Seq Line	:	27
		Location	:	82
		Inj. Vol.	:	2 µl

Acq. Method : C:\Users\Public\Documents\ChemStation\1\Data\SE26SEP 2023-09-26 16-24-18\22010446 LCMS-6.M

Analysis Method : C:\Users\Public\Documents\ChemStation\1\Data\09. September\SE26SEP\SE26SEP 2023-09-26 16-24-18\22010446 LCMS-6.M (Sequence->

Waters XBridge Phenyl (4.6 * 150 mm; 3.5 µm); 0.05% TFA (aq) / AcN: 100/0 (0.0 min) --> (6.0 min) --> 70/30 (0.0 min) --> (2.0 min) --> 10/90 (2.0 min); Flow: 1.0 ml/min;
MSD1 = positive; MSD2 = negative

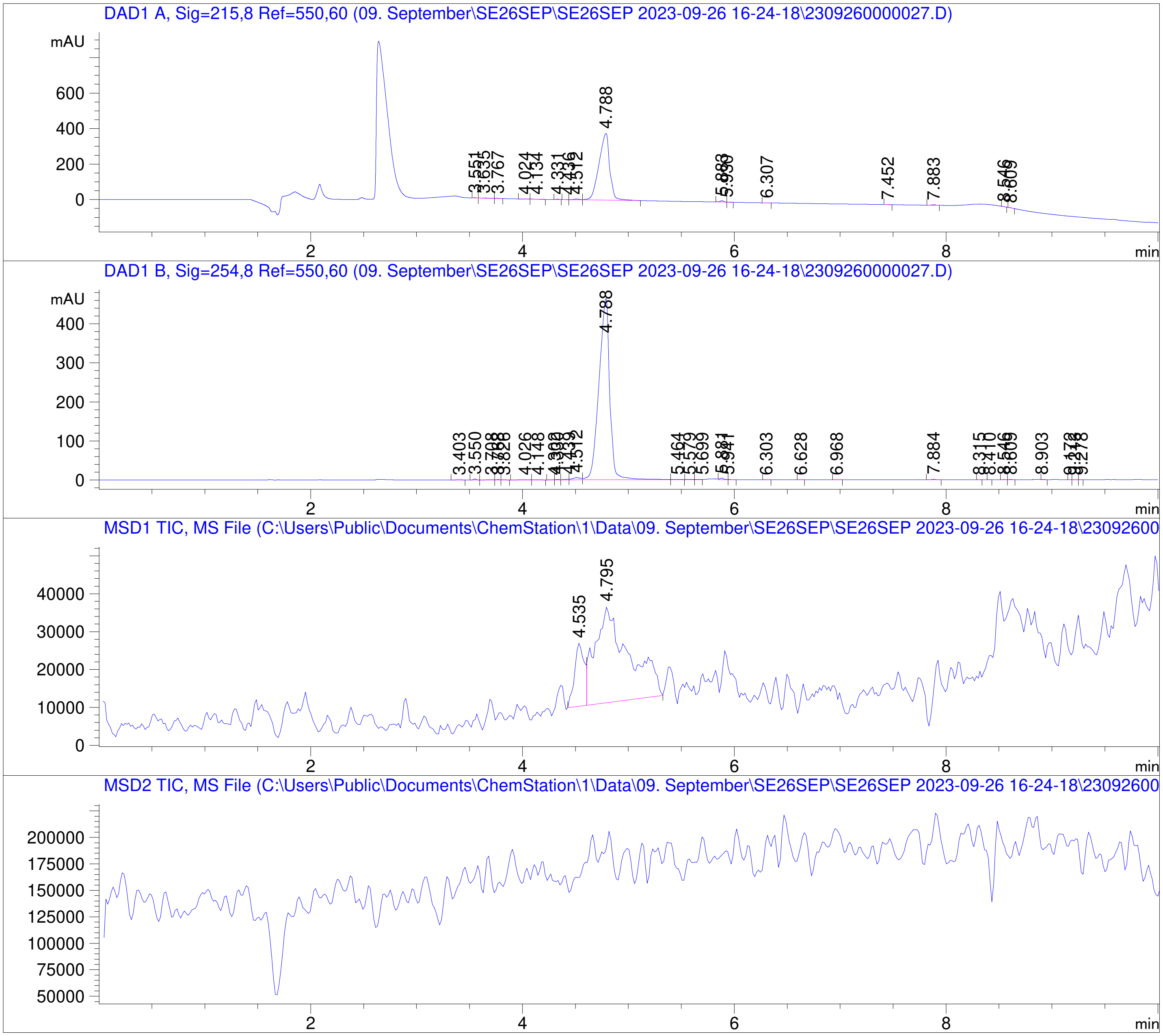

===

DAD1 A, Sig=215,8 Ref=550,60

Peak #	Ret. Time [min]	Area [mV *s]	Area %
1	3.551	3.066	0.113
2	3.635	4.150	0.154
3	3.767	2.060	0.076
4	4.024	1.846	0.068
5	4.134	2.894	0.107
6	4.331	4.459	0.165
7	4.436	1.944	0.072
8	4.512	19.873	0.735
9	4.788	2631.823	97.352
10	5.883	17.325	0.641
11	5.930	1.305	0.048
12	6.307	0.972	0.036
13	7.452	0.731	0.027
14	7.883	7.225	0.267
15	8.546	1.504	0.056
16	8.609	2.239	0.083

DAD1 B, Sig=254,8 Ref=550,60

Peak #	Ret. Time [min]	Area [mV *s]	Area %
1	3.403	1.976	0.059
2	3.550	4.276	0.127
3	3.708	1.806	0.054
4	3.768	2.109	0.063
5	3.826	1.265	0.038
6	4.026	2.913	0.087
7	4.148	2.356	0.070
8	4.302	1.416	0.042
9	4.330	3.352	0.100
10	4.439	5.354	0.159
11	4.512	30.684	0.913
12	4.788	3275.382	97.486
13	5.464	5.286	0.157
14	5.579	2.239	0.067
15	5.699	2.139	0.064
16	5.881	10.693	0.318
17	5.941	0.785	0.023
18	6.303	0.201	0.006
19	6.628	0.111	0.003
20	6.968	0.110	0.003
21	7.884	3.477	0.103
22	8.315	0.080	0.002
23	8.410	0.082	0.002
24	8.546	0.352	0.010
25	8.609	0.410	0.012
26	8.903	0.347	0.010
27	9.172	0.125	0.004
28	9.213	0.259	0.008
29	9.278	0.259	0.008

==

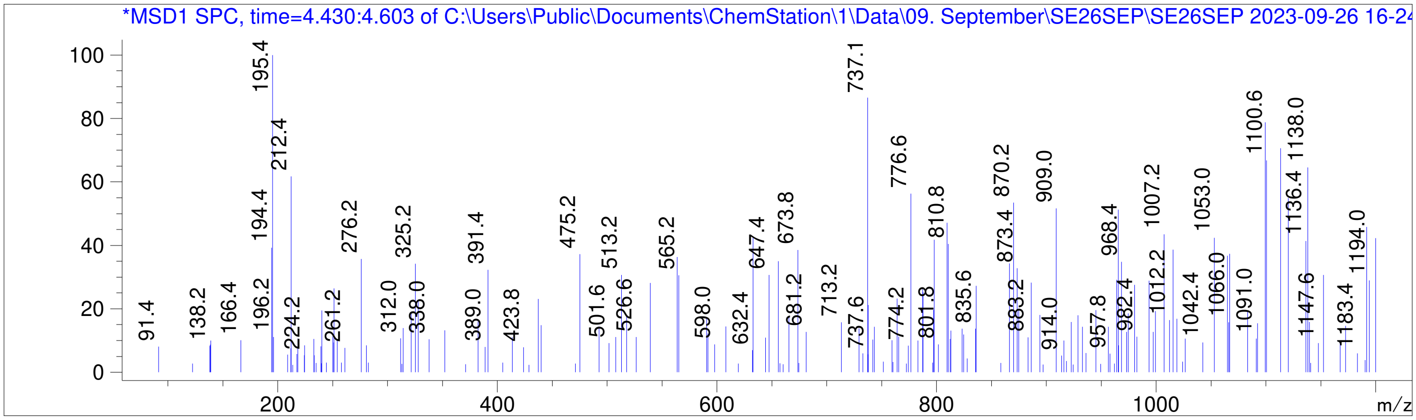
*MSD1 SPC, time=4.430:4.603 of C:\Users\Public\Documents\ChemStation\1\Data\09. September\SE26SEP\SE26SEP 2023-09-26 16-24
195.4
737.1
1100.6
1138.0
212.4
776.6
870.2
909.0
1007.2
1053.0
1136.4
194.4
276.2
325.2
391.4
475.2
513.2
565.2
647.4
673.8
810.8
873.4
968.4
1194.0
m/z

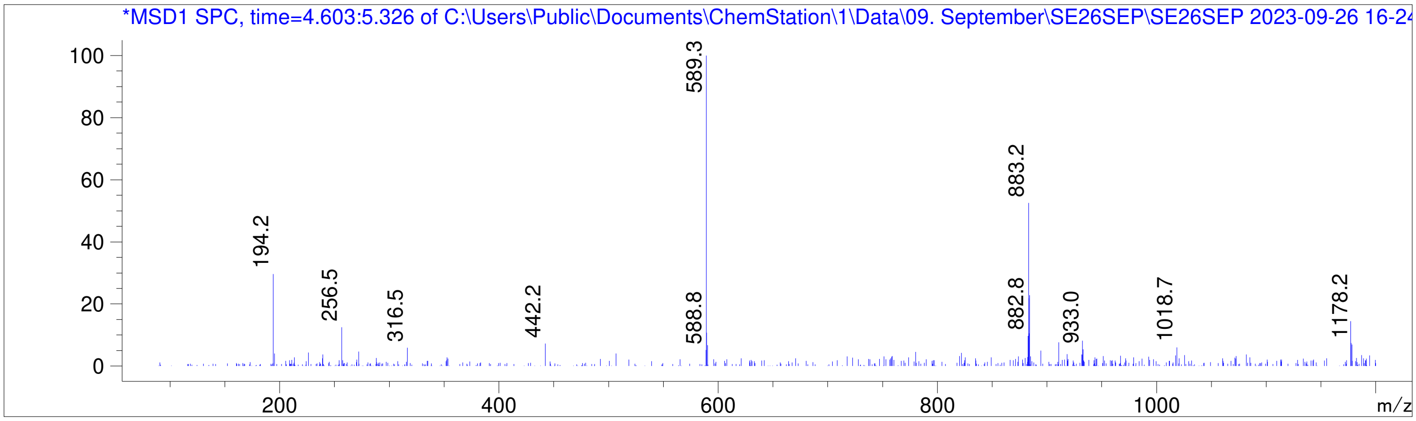
*MSD1 SPC, time=4.603:5.326 of C:\Users\Public\Documents\ChemStation\1\Data\09. September\SE26SEP\SE26SEP 2023-09-26 16-24
589.3
883.2
194.2
256.5
316.5
442.2
588.8
882.8
933.0
1018.7
1178.2
m/z
