# Supplementary material for: Astrobiological implications of the stability and reactivity of peptide nucleic acid (PNA) in concentrated sulfuric acid
Source: Sci Adv. 2025 Mar 26;11(13):eadr0006. doi: 10.1126/sciadv.adr0006 (PMC11939054; doi:10.1126/sciadv.adr0006)

Sample-> CPT22010446-19-D1-50deg-1h

Injection Date : Tue, 17. Oct. 2023

Seq Line : 4
Location : 34
Inj. Vol. : 2 µl

Acq. Method : C:\Users\Public\Documents\ChemStation\1\Data\SE17OCT 2023-10-17 14-37-22\22010446C LCMS-6#.M

Analysis Method : C:\Users\Public\Documents\ChemStation\1\Data\SE17OCT 2023-10-17 14-37-22\22010446C LCMS-6#.M (Sequence Method)

Waters XBridge BEH Amide (4.6 x 150 mm, 2.5 µm); PN# 186006726
Mobile Phase A: 20mM Ammonium Acetate (aq) pH 8.2
Mobile Phase B: AcN
Mobile Phase A / Mobile Phase B: 5/95 (0 min) --> (10 min) --> 60/40 (5 min); Flow: 1.0 ml/min; MSD1 = positive; MSD2 = negative

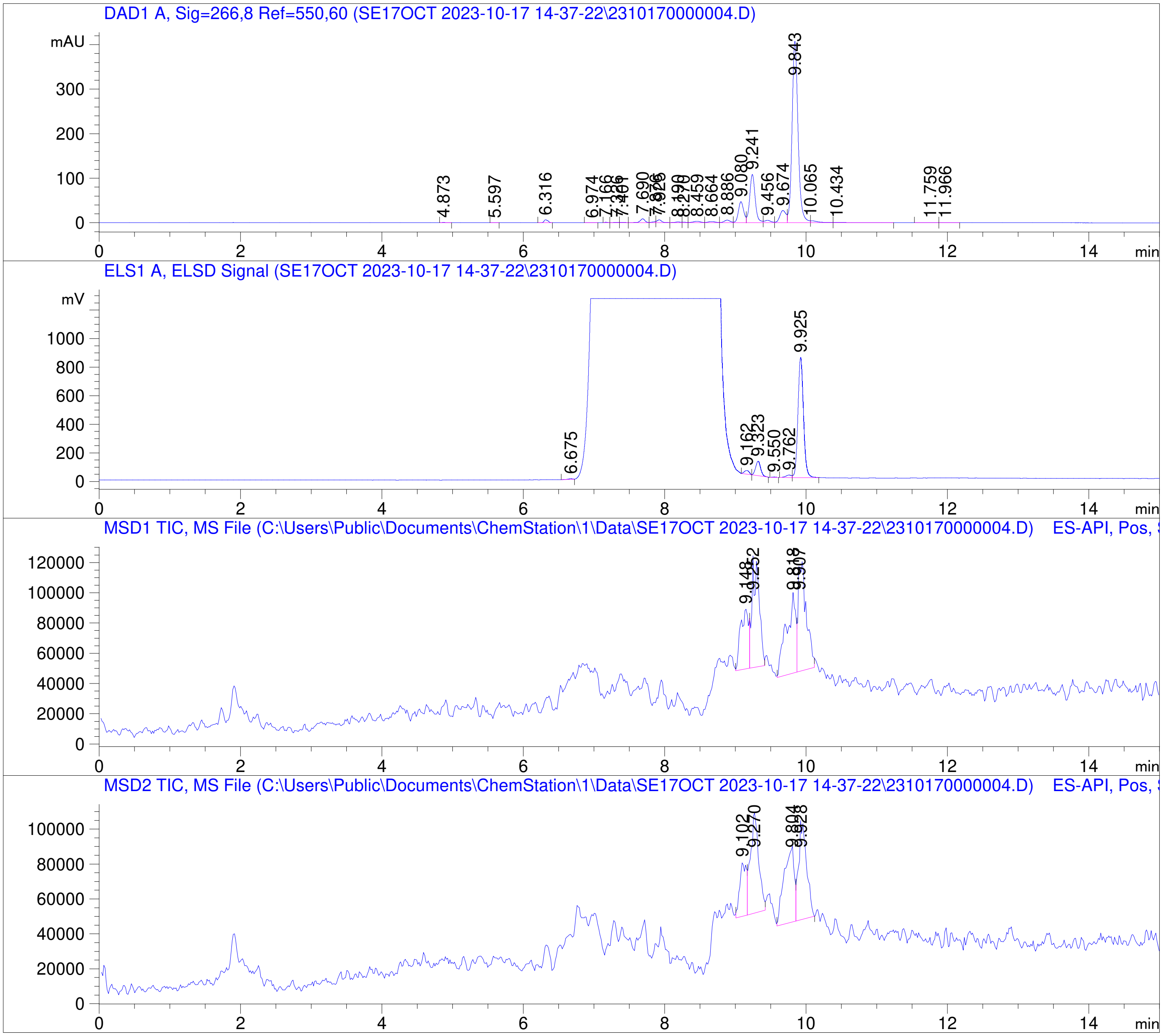

Sample-> CPT22010446-19-D1-50deg-1h

DAD1 A, Sig=266,8 Ref=550,60

Peak #	Ret. Time [min]	Area [mV *s]	Area %
1	4.873	2.402	0.065
2	5.597	0.605	0.016
3	6.316	24.671	0.667
4	6.974	1.218	0.033
5	7.166	3.220	0.087
6	7.326	4.032	0.109
7	7.401	4.930	0.133
8	7.690	43.748	1.182
9	7.876	9.156	0.247
10	7.925	31.278	0.845
11	8.190	10.948	0.296
12	8.270	4.894	0.132
13	8.459	25.018	0.676
14	8.664	20.100	0.543
15	8.886	34.781	0.940
16	9.080	248.301	6.711
17	9.241	581.843	15.725
18	9.456	33.761	0.912
19	9.674	166.599	4.503
20	9.843	2404.325	64.982
21	10.065	32.577	0.880
22	10.434	10.036	0.271
23	11.759	0.799	0.022
24	11.966	0.760	0.021

ELS1 A, ELSD Signal

Peak #	Ret. Time [min]	Area [mV *s]	Area %
1	6.675	19.448	0.364
2	9.162	120.817	2.261
3	9.323	505.393	9.457
4	9.550	11.080	0.207
5	9.762	97.041	1.816
6	9.925	4590.542	85.896

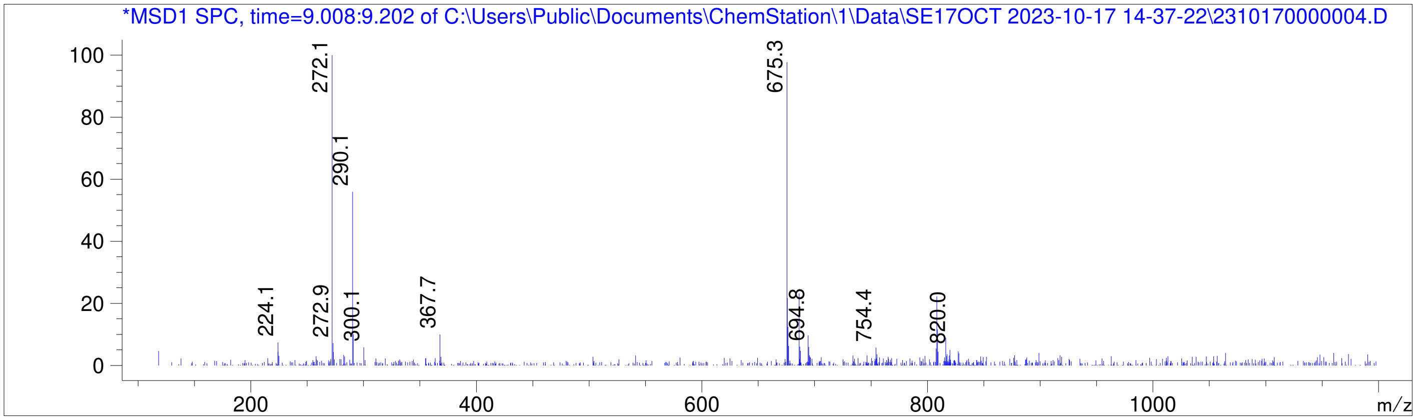
*MSD1 SPC, time=9.008:9.202 of C:\Users\Public\Documents\ChemStation\1\Data\SE17OCT 2023-10-17 14-37-22\2310170000004.D
100
80
60
40
20
0
272.1
675.3
290.1
224.1
272.9
300.1
367.7
694.8
754.4
820.0
200
400
600
800
1000
m/z

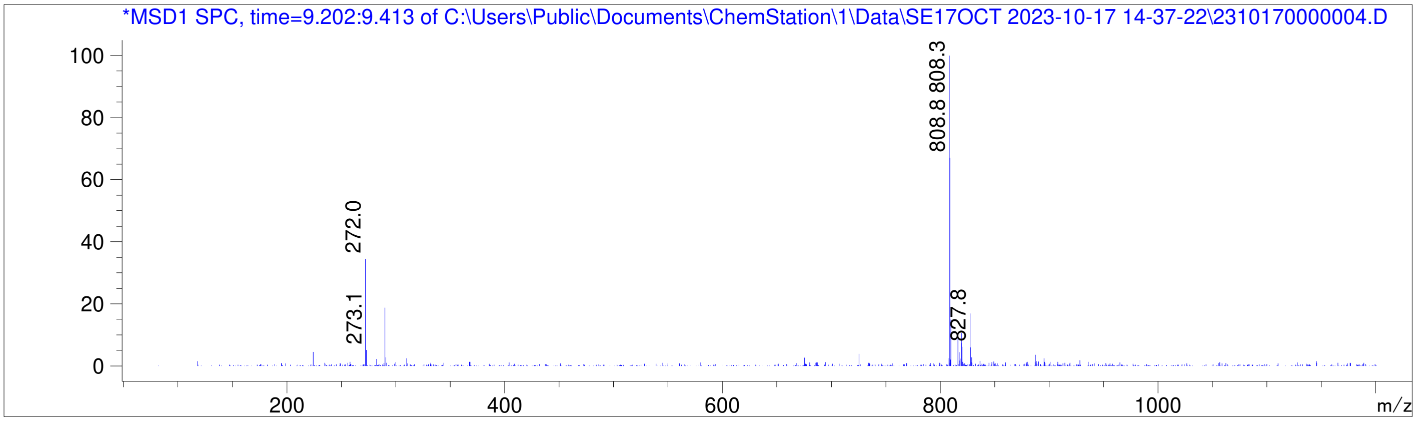
*MSD1 SPC, time=9.202:9.413 of C:\Users\Public\Documents\ChemStation\1\Data\SE17OCT 2023-10-17 14-37-22\2310170000004.D
100
80
60
40
20
0
808.8 808.3
272.0
273.1
827.8
200
400
600
800
1000
m/z

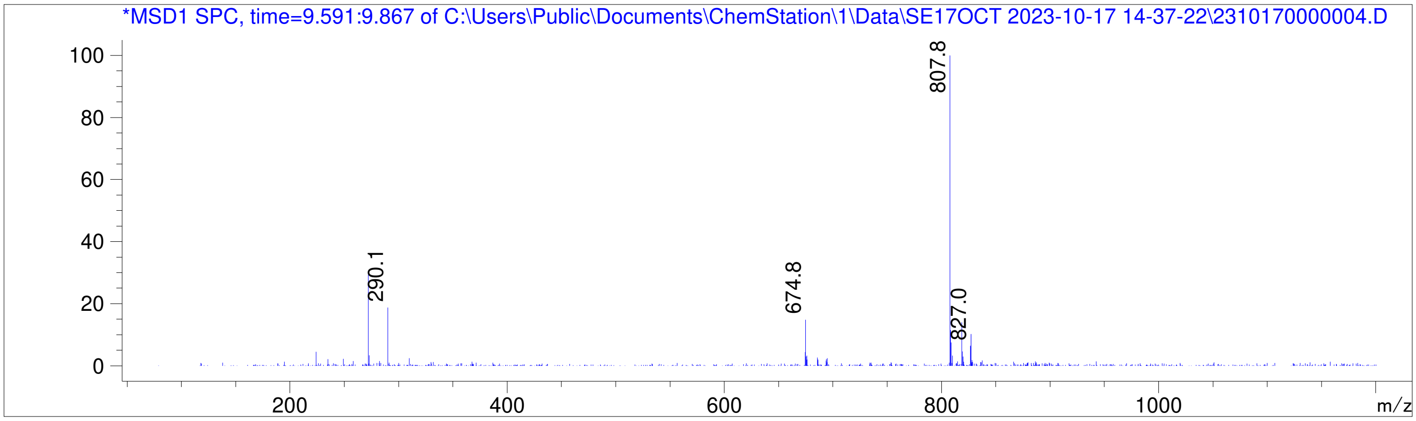
*MSD1 SPC, time=9.591:9.867 of C:\Users\Public\Documents\ChemStation\1\Data\SE17OCT 2023-10-17 14-37-22\2310170000004.D
100
80
60
40
20
0
807.8
290.1
674.8
827.0
200
400
600
800
1000
m/z

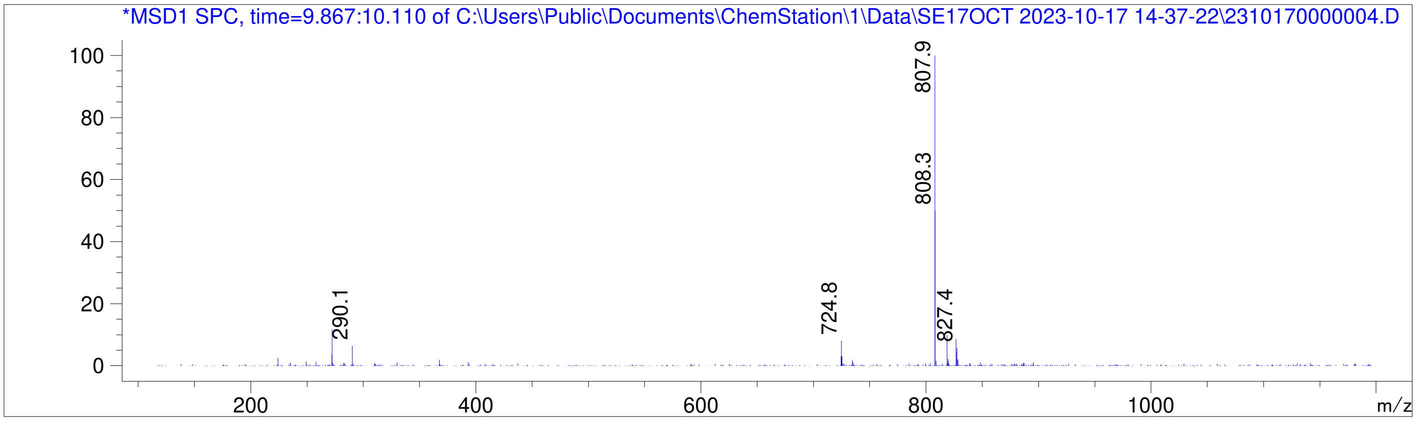
*MSD1 SPC, time=9.867:10.110 of C:\Users\Public\Documents\ChemStation\1\Data\SE17OCT 2023-10-17 14-37-22\2310170000004.D
100
80
60
40
20
0
807.9
808.3
290.1
724.8
827.4
200
400
600
800
1000
m/z

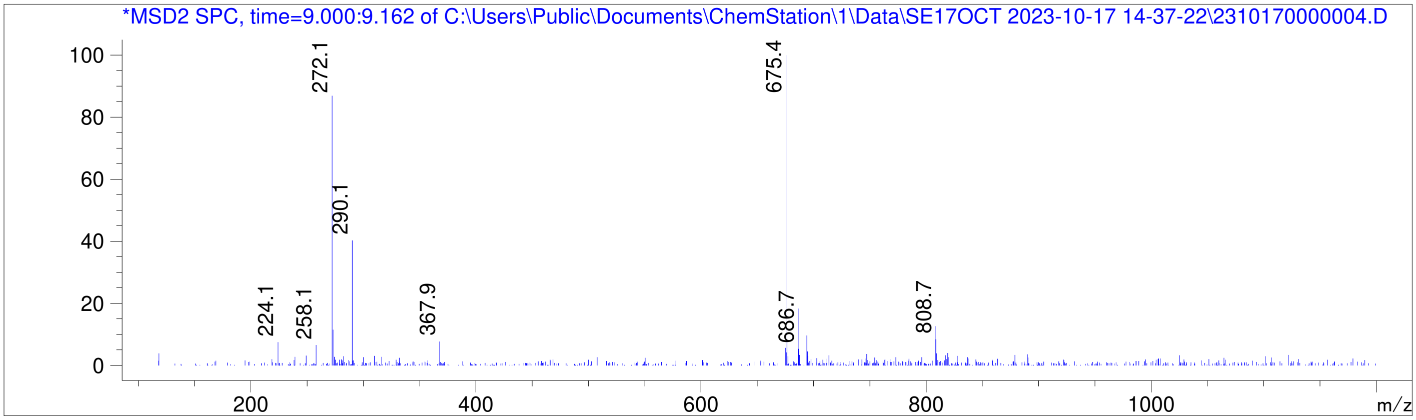
*MSD2 SPC, time=9.000:9.162 of C:\Users\Public\Documents\ChemStation\1\Data\SE17OCT 2023-10-17 14-37-22\2310170000004.D
272.1
675.4
290.1
224.1
258.1
367.9
686.7
808.7
m/z

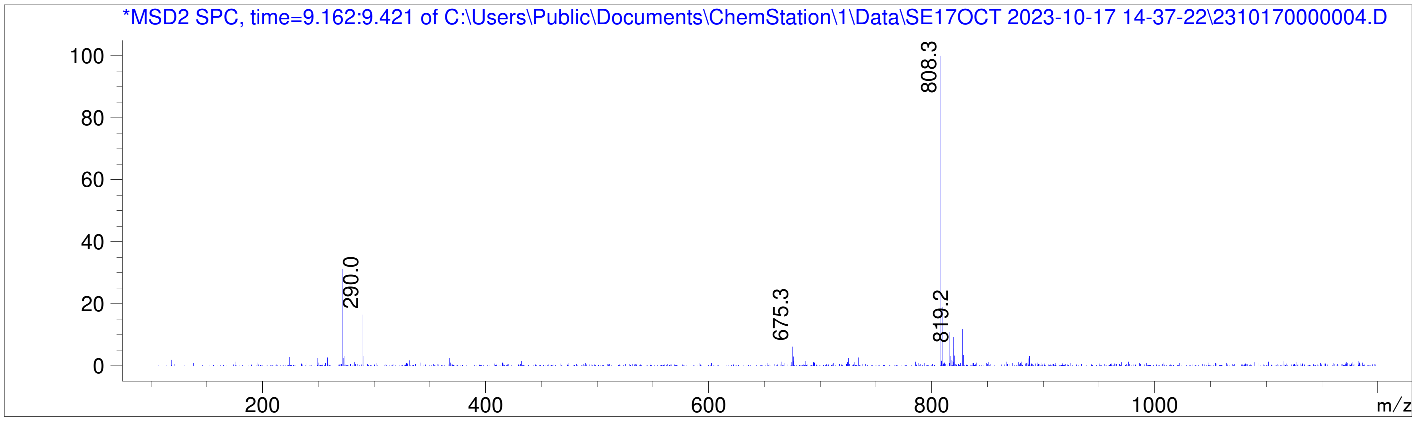
*MSD2 SPC, time=9.162:9.421 of C:\Users\Public\Documents\ChemStation\1\Data\SE17OCT 2023-10-17 14-37-22\2310170000004.D
808.3
290.0
675.3
819.2
m/z

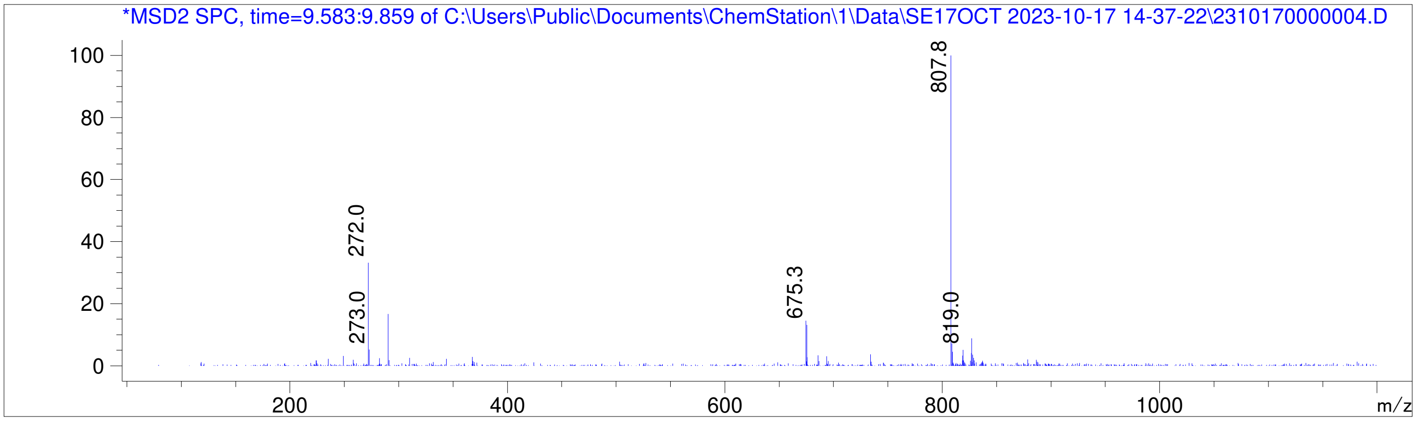
*MSD2 SPC, time=9.583:9.859 of C:\Users\Public\Documents\ChemStation\1\Data\SE17OCT 2023-10-17 14-37-22\2310170000004.D
807.8
272.0
675.3
273.0
819.0
m/z

Sample-> CPT22010446-19-D1-50deg-1h

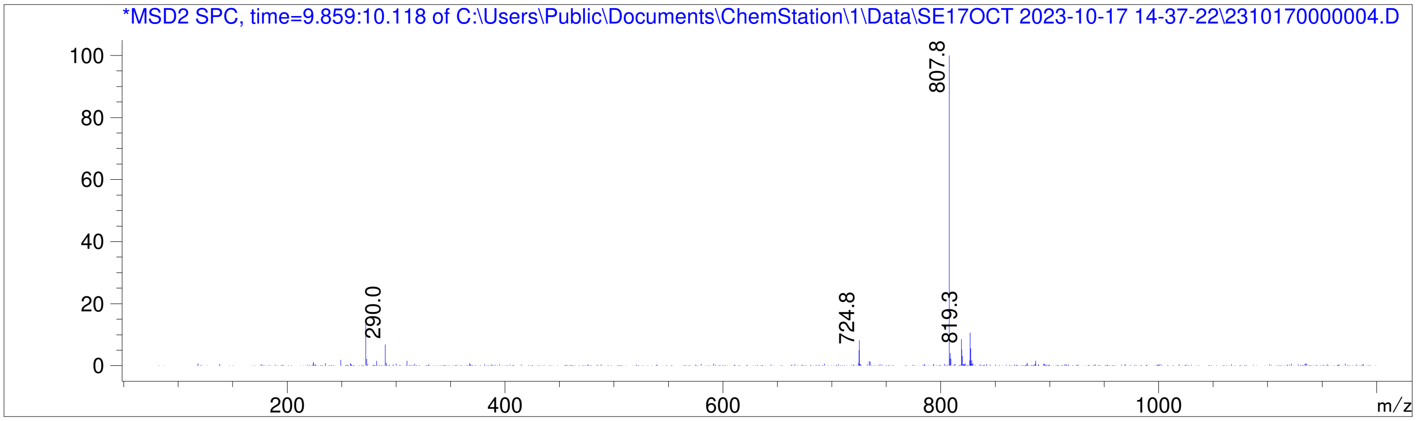

Supplement: Supplementary file 2 — Data S1 and S2 [file sciadv.adr0006_data_s1_and_s2.zip › Supplementary Dataset 1-LCMS DATA/LCMS PNA Hexamers A-T/LCMS T6 50C_80C/50C/1h/CPT22010446-19-D1-50deg-1h.pdf]
