# Supplementary material for: Astrobiological implications of the stability and reactivity of peptide nucleic acid (PNA) in concentrated sulfuric acid
Source: Sci Adv. 2025 Mar 26;11(13):eadr0006. doi: 10.1126/sciadv.adr0006 (PMC11939054; doi:10.1126/sciadv.adr0006)
Supplement: Supplementary file 2 — Data S1 and S2 [file sciadv.adr0006_data_s1_and_s2.zip › Supplementary Dataset 1-LCMS DATA/LCMS PNA Hexamers A-T/LCMS T6 50C_80C/50C/1h/CPT22010446-19-D2-50deg-1h.pdf]

Sample-> CPT22010446-19-D2-50deg-1h

=====================================================================================

| | | | | | |
|---|---|---|---|---|---|
| Injection Date | : | Thu, 2. Nov. 2023 | Seq Line | : | 32 |
| | | | Location | : | 10 |
| | | | Inj. Vol. | : | 2 µl |

Acq. Method : C:\Users\Public\Documents\ChemStation\1\Data\SE02NOV 2023-11-02 14-31-42\22010446C LCMS-6#.M

Analysis Method : C:\Users\Public\Documents\ChemStation\1\Data\SE02NOV 2023-11-02 14-31-42\22010446C LCMS-6#.M (Sequence Method)

Waters XBridge BEH Amide (4.6 x 150 mm, 2.5 µm); PN# 186006726

Mobile Phase A: 20mM Ammonium Acetate (aq) pH 8.2

Mobile Phase B: AcN

Mobile Phase A / Mobile Phase B: 5/95 (0 min) --> (10 min) --> 60/40 (5 min); Flow: 1.0 ml/min; MSD1 = positive; MSD2 = negative

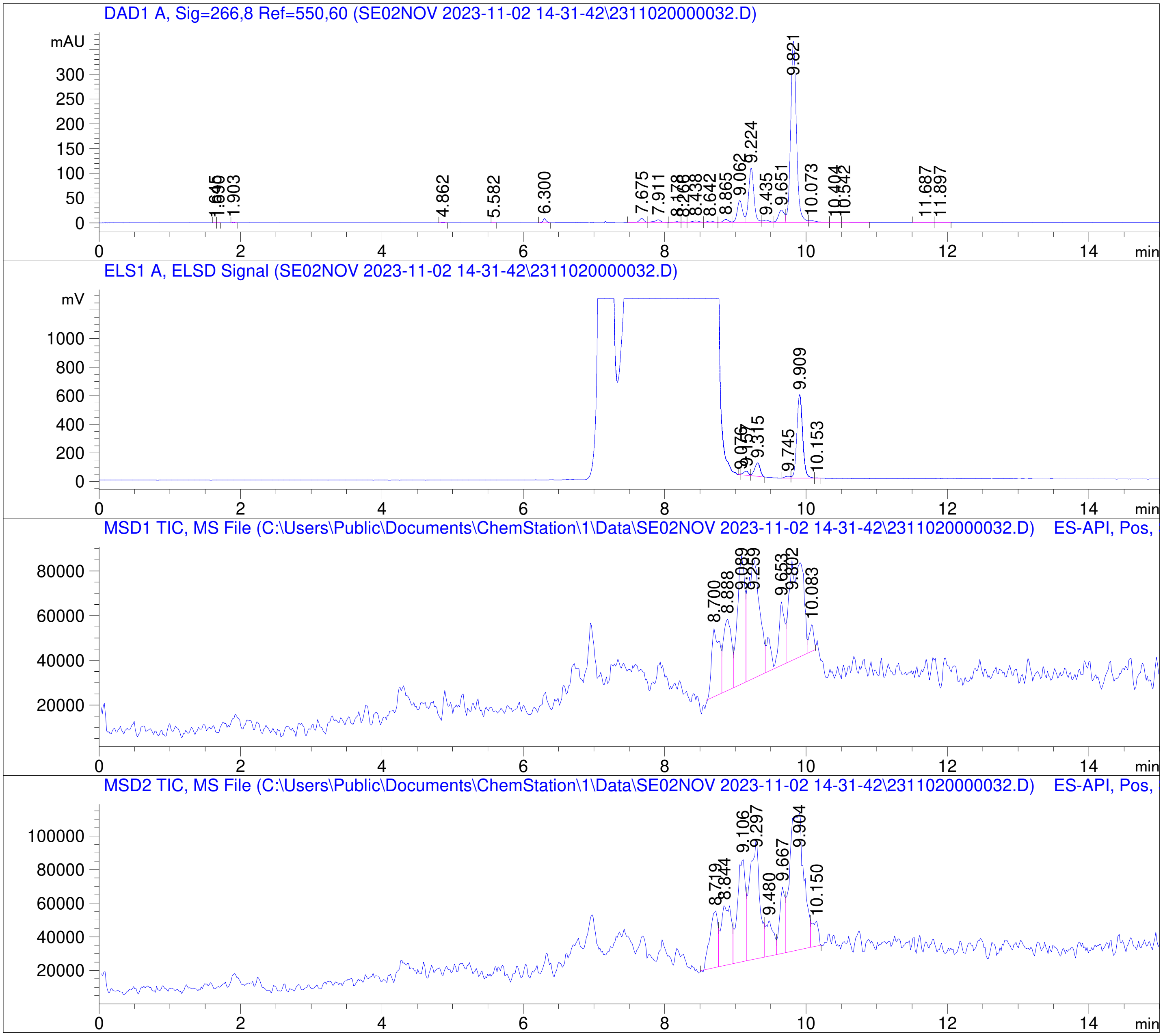

DAD1 A, Sig=266,8 Ref=550,60

| Peak # | Ret. Time [min] | Area [mV *s] | Area % |
|---|---|---|---|
| 1 | 1.645 | 0.182 | 0.006 |
| 2 | 1.690 | 0.372 | 0.011 |
| 3 | 1.903 | 1.996 | 0.061 |
| 4 | 4.862 | 2.430 | 0.075 |
| 5 | 5.582 | 0.402 | 0.012 |
| 6 | 6.300 | 23.140 | 0.712 |
| 7 | 7.675 | 36.079 | 1.110 |
| 8 | 7.911 | 31.107 | 0.957 |
| 9 | 8.178 | 6.424 | 0.198 |
| 10 | 8.266 | 3.381 | 0.104 |
| 11 | 8.438 | 20.486 | 0.630 |
| 12 | 8.642 | 15.805 | 0.486 |
| 13 | 8.865 | 31.159 | 0.959 |
| 14 | 9.062 | 225.373 | 6.935 |
| 15 | 9.224 | 571.569 | 17.589 |
| 16 | 9.435 | 27.893 | 0.858 |
| 17 | 9.651 | 146.689 | 4.514 |
| 18 | 9.821 | 2068.813 | 63.663 |
| 19 | 10.073 | 27.415 | 0.844 |
| 20 | 10.404 | 3.921 | 0.121 |
| 21 | 10.542 | 3.307 | 0.102 |
| 22 | 11.687 | 0.942 | 0.029 |
| 23 | 11.897 | 0.766 | 0.024 |

ELS1 A, ELSD Signal

| Peak # | Ret. Time [min] | Area [mV *s] | Area % |
|---|---|---|---|
| 1 | 9.076 | 6.705 | 0.165 |
| 2 | 9.157 | 142.522 | 3.506 |
| 3 | 9.315 | 512.769 | 12.614 |
| 4 | 9.745 | 68.008 | 1.673 |
| 5 | 9.909 | 3328.510 | 81.879 |
| 6 | 10.153 | 6.658 | 0.164 |

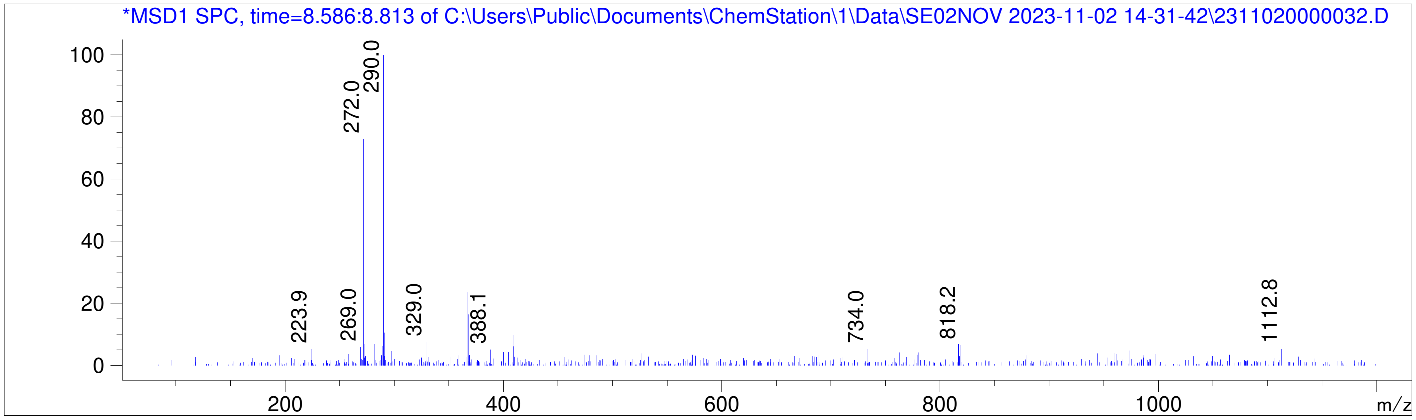
*MSD1 SPC, time=8.586:8.813 of C:\Users\Public\Documents\ChemStation\1\Data\SE02NOV 2023-11-02 14-31-42\2311020000032.D
100
80
60
40
20
0
290.0
272.0
223.9
269.0
329.0
388.1
734.0
818.2
1112.8
200
400
600
800
1000
m/z

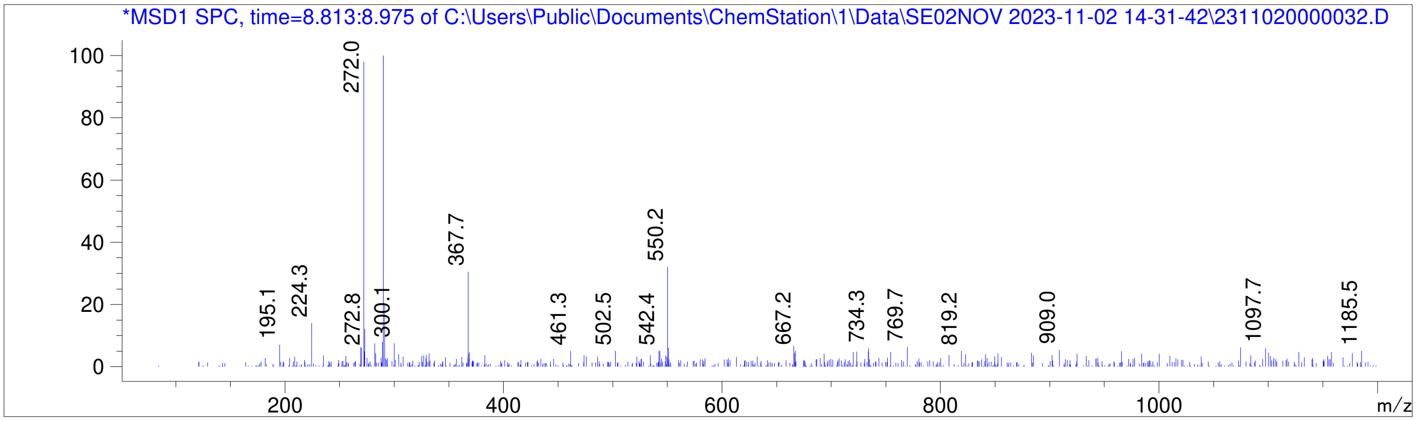
*MSD1 SPC, time=8.813:8.975 of C:\Users\Public\Documents\ChemStation\1\Data\SE02NOV 2023-11-02 14-31-42\2311020000032.D
100
80
60
40
20
0
272.0
367.7
550.2
195.1
224.3
272.8
300.1
461.3
502.5
542.4
667.2
734.3
769.7
819.2
909.0
1097.7
1185.5
200
400
600
800
1000
m/z

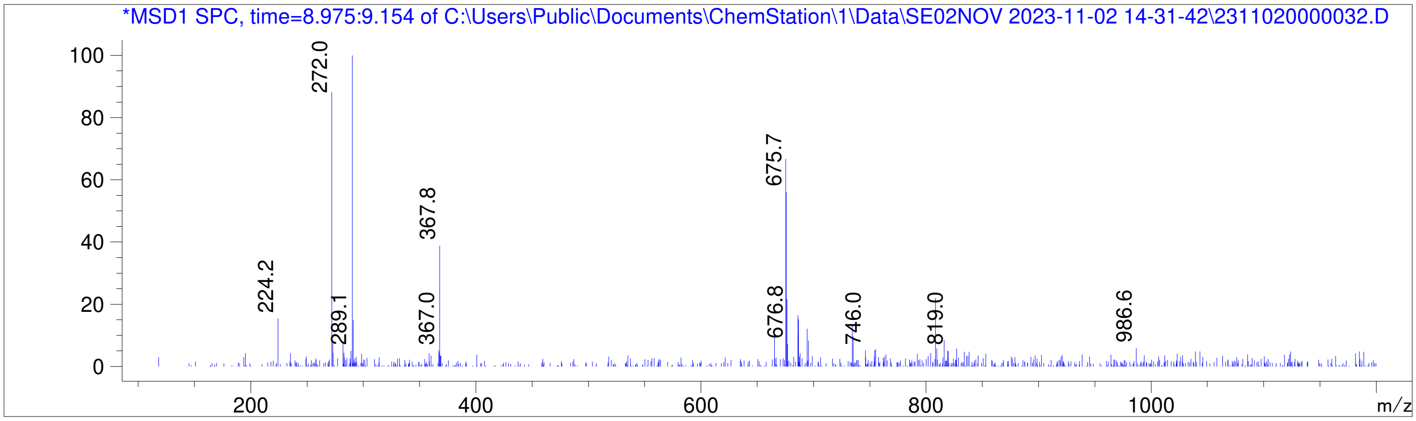
*MSD1 SPC, time=8.975:9.154 of C:\Users\Public\Documents\ChemStation\1\Data\SE02NOV 2023-11-02 14-31-42\2311020000032.D
100
80
60
40
20
0
272.0
675.7
367.8
224.2
289.1
367.0
676.8
746.0
819.0
986.6
200
400
600
800
1000
m/z

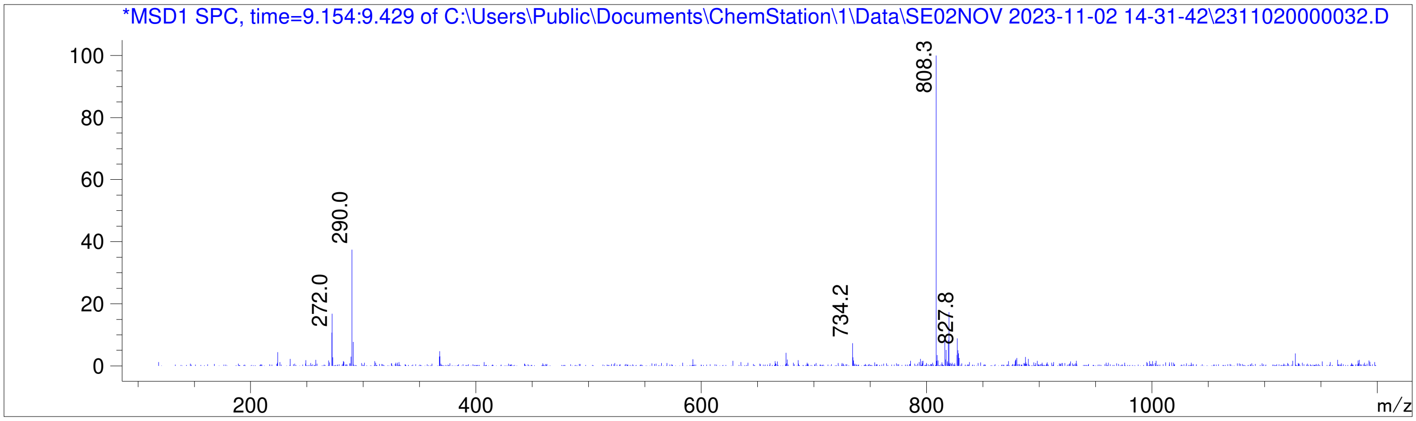
*MSD1 SPC, time=9.154:9.429 of C:\Users\Public\Documents\ChemStation\1\Data\SE02NOV 2023-11-02 14-31-42\231102000032.D
808.3
290.0
272.0
734.2
827.8
m/z

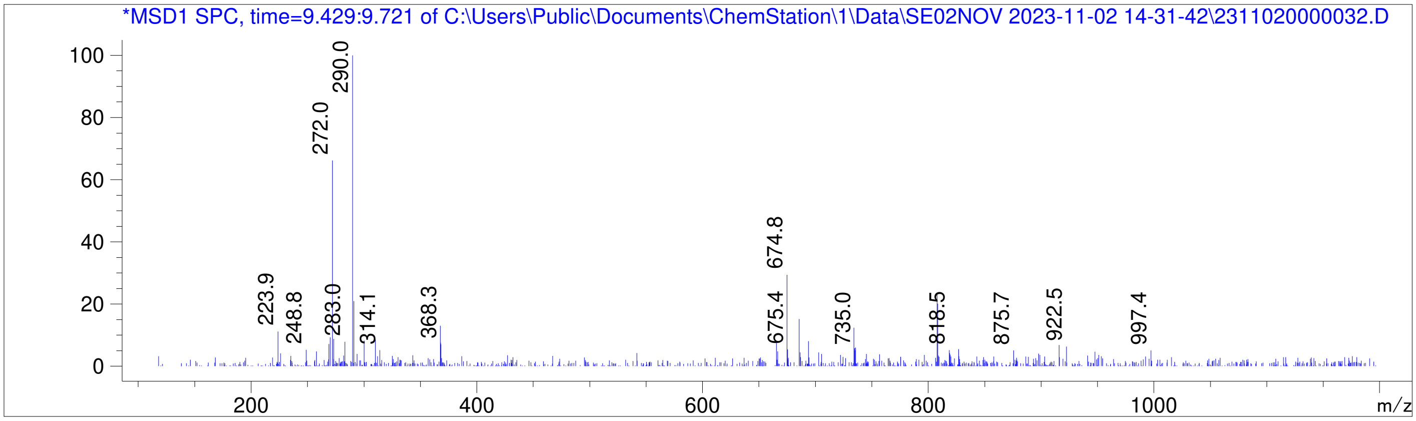
*MSD1 SPC, time=9.429:9.721 of C:\Users\Public\Documents\ChemStation\1\Data\SE02NOV 2023-11-02 14-31-42\231102000032.D
290.0
272.0
674.8
223.9
248.8
283.0
314.1
368.3
675.4
735.0
818.5
875.7
922.5
997.4
m/z

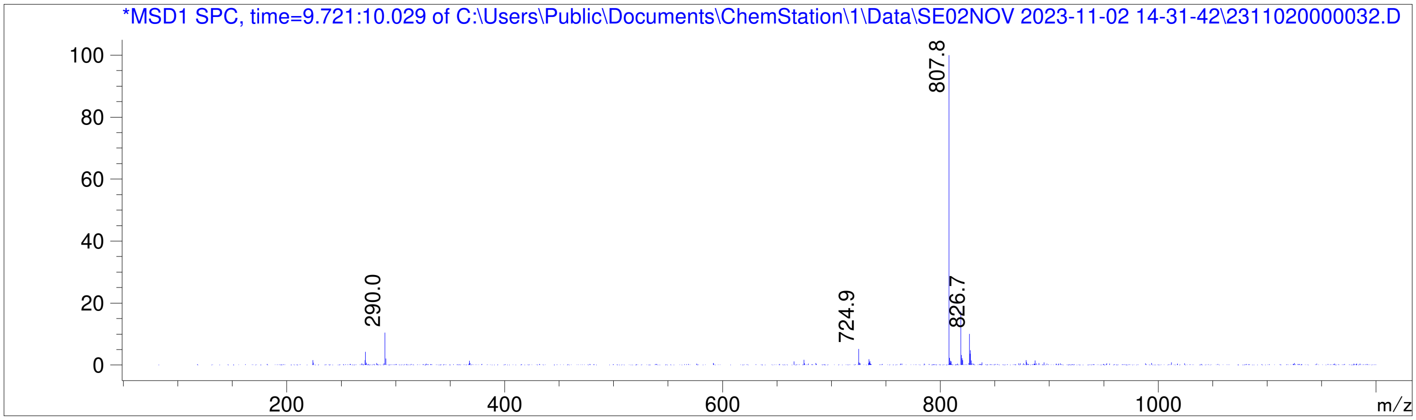
*MSD1 SPC, time=9.721:10.029 of C:\Users\Public\Documents\ChemStation\1\Data\SE02NOV 2023-11-02 14-31-42\231102000032.D
807.8
290.0
724.9
826.7
m/z

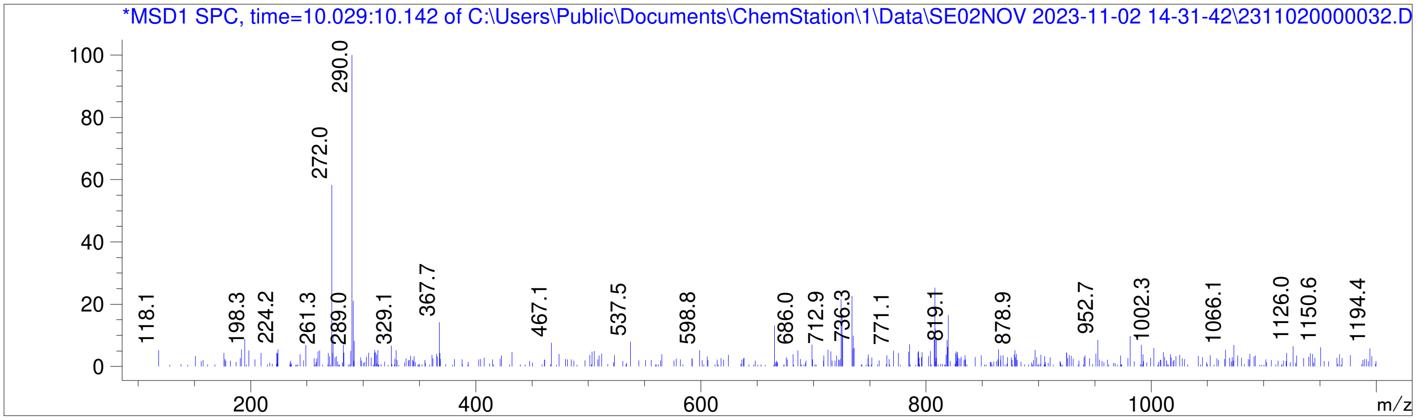
*MSD1 SPC, time=10.029:10.142 of C:\Users\Public\Documents\ChemStation\1\Data\SE02NOV 2023-11-02 14-31-42\231102000032.D
290.0
272.0
118.1
198.3
224.2
261.3
289.0
329.1
367.7
467.1
537.5
598.8
686.0
712.9
736.3
771.1
819.1
878.9
952.7
1002.3
1066.1
1126.0
1150.6
1194.4
m/z

================================================================================

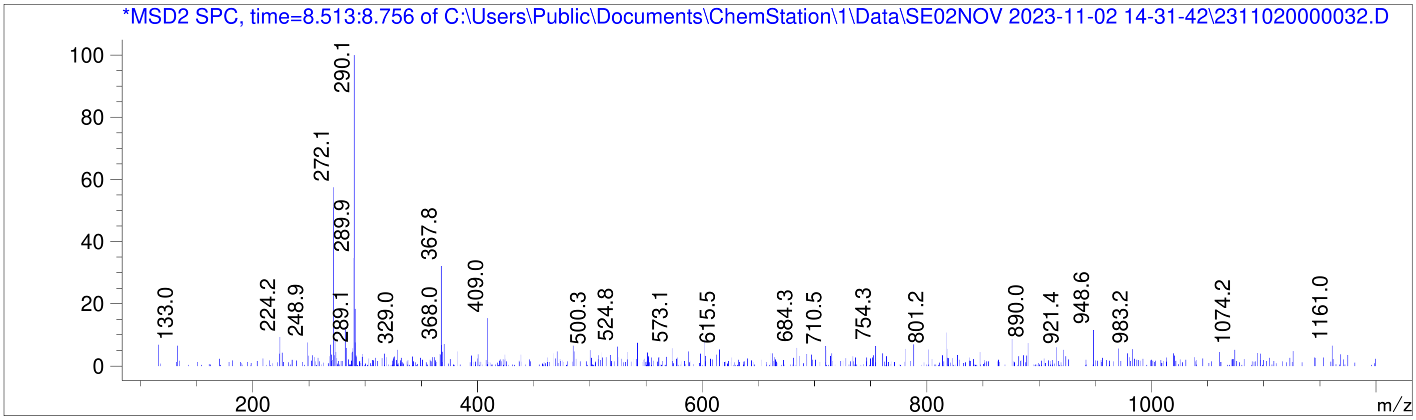
*MSD2 SPC, time=8.513:8.756 of C:\Users\Public\Documents\ChemStation\1\Data\SE02NOV 2023-11-02 14-31-42\2311020000032.D
290.1
272.1
289.9
367.8
409.0
133.0
224.2
248.9
289.1
329.0
368.0
500.3
524.8
573.1
615.5
684.3
710.5
754.3
801.2
890.0
921.4
948.6
983.2
1074.2
1161.0
m/z

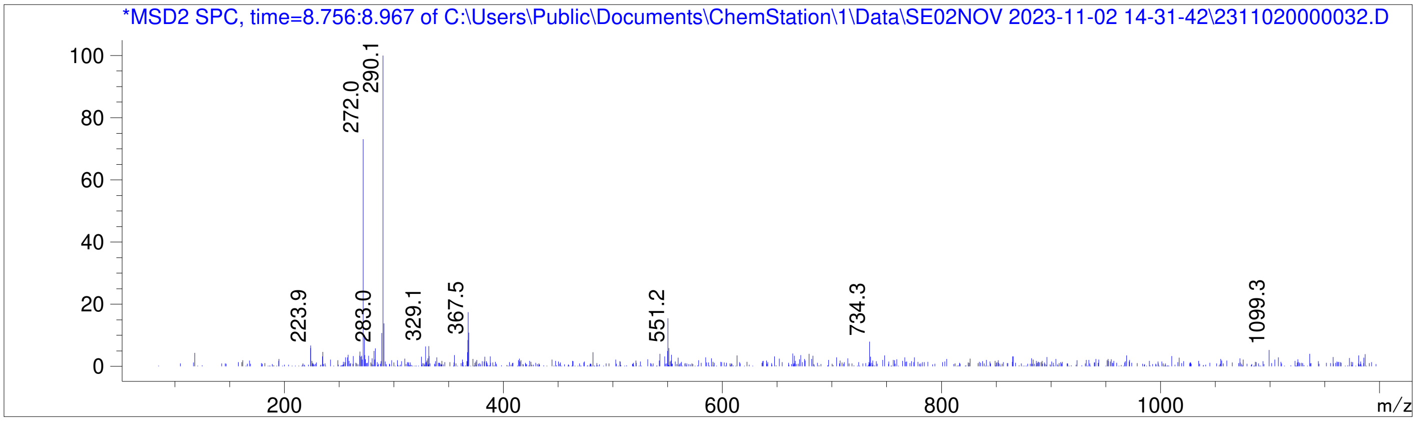
*MSD2 SPC, time=8.756:8.967 of C:\Users\Public\Documents\ChemStation\1\Data\SE02NOV 2023-11-02 14-31-42\2311020000032.D
290.1
272.0
223.9
283.0
329.1
367.5
551.2
734.3
1099.3
m/z

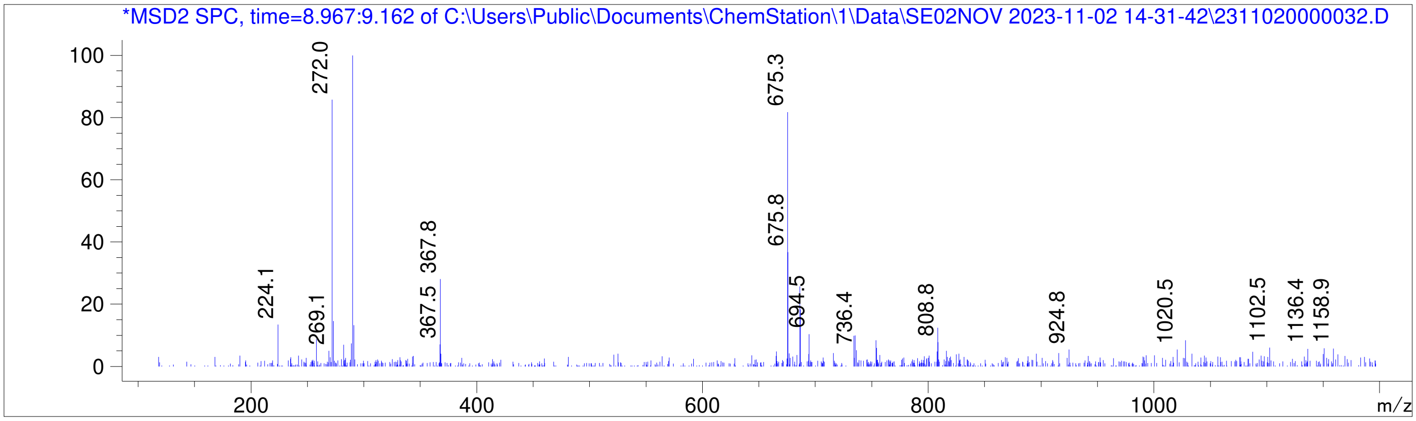
*MSD2 SPC, time=8.967:9.162 of C:\Users\Public\Documents\ChemStation\1\Data\SE02NOV 2023-11-02 14-31-42\2311020000032.D
272.0
675.3
675.8
367.8
224.1
269.1
367.5
694.5
736.4
808.8
924.8
1020.5
1102.5
1136.4
1158.9
m/z

===============================================================================

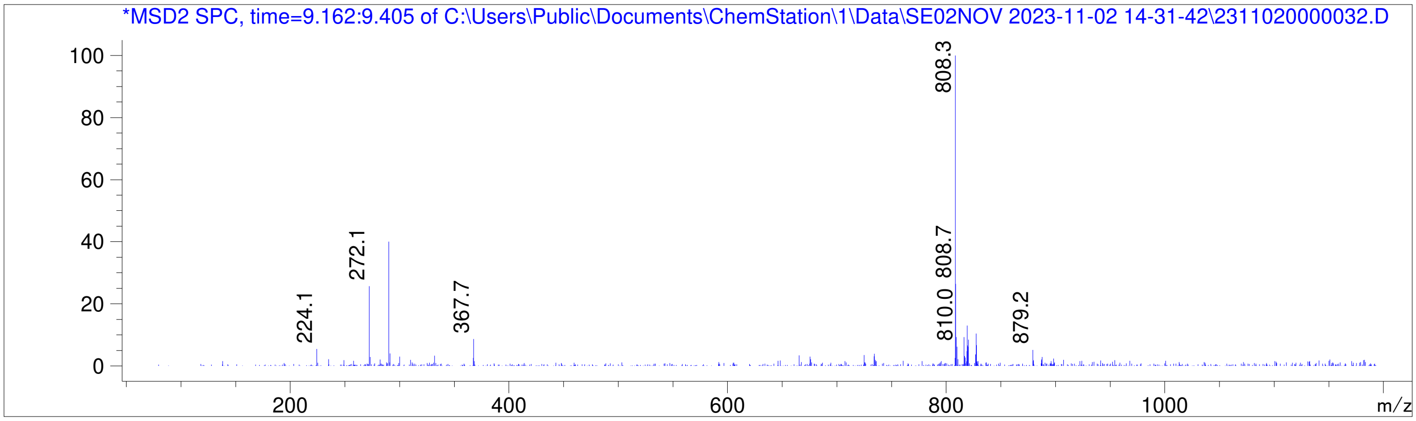
*MSD2 SPC, time=9.162:9.405 of C:\Users\Public\Documents\ChemStation\1\Data\SE02NOV 2023-11-02 14-31-42\231102000032.D
808.3
808.7
810.0
879.2
272.1
224.1
367.7
m/z

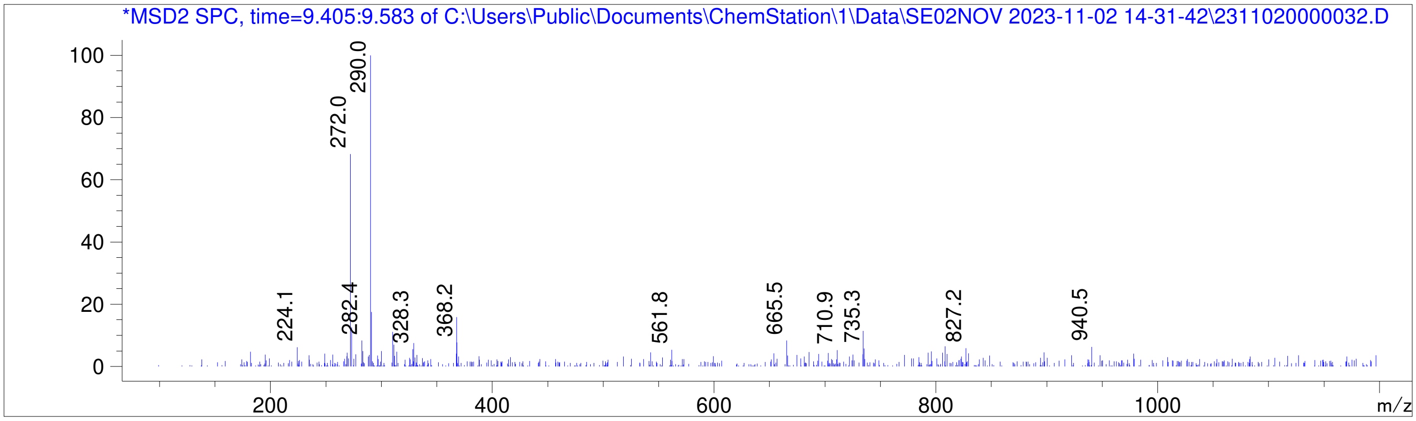
*MSD2 SPC, time=9.405:9.583 of C:\Users\Public\Documents\ChemStation\1\Data\SE02NOV 2023-11-02 14-31-42\231102000032.D
290.0
272.0
224.1
282.4
328.3
368.2
561.8
665.5
710.9
735.3
827.2
940.5
m/z

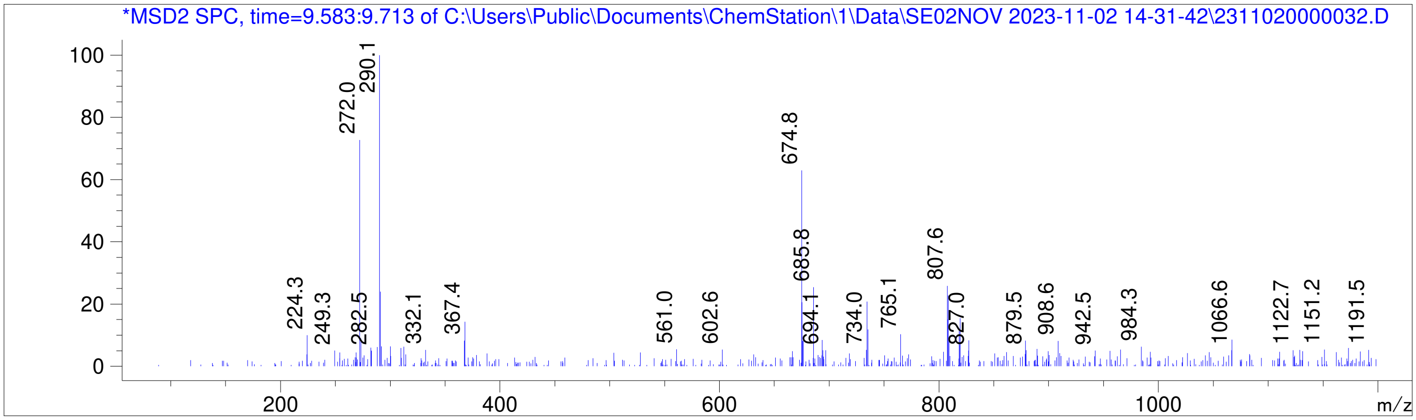
*MSD2 SPC, time=9.583:9.713 of C:\Users\Public\Documents\ChemStation\1\Data\SE02NOV 2023-11-02 14-31-42\231102000032.D
290.1
272.0
674.8
685.8
807.6
224.3
249.3
282.5
332.1
367.4
561.0
602.6
694.1
734.0
765.1
827.0
879.5
908.6
942.5
984.3
1066.6
1122.7
1151.2
1191.5
m/z

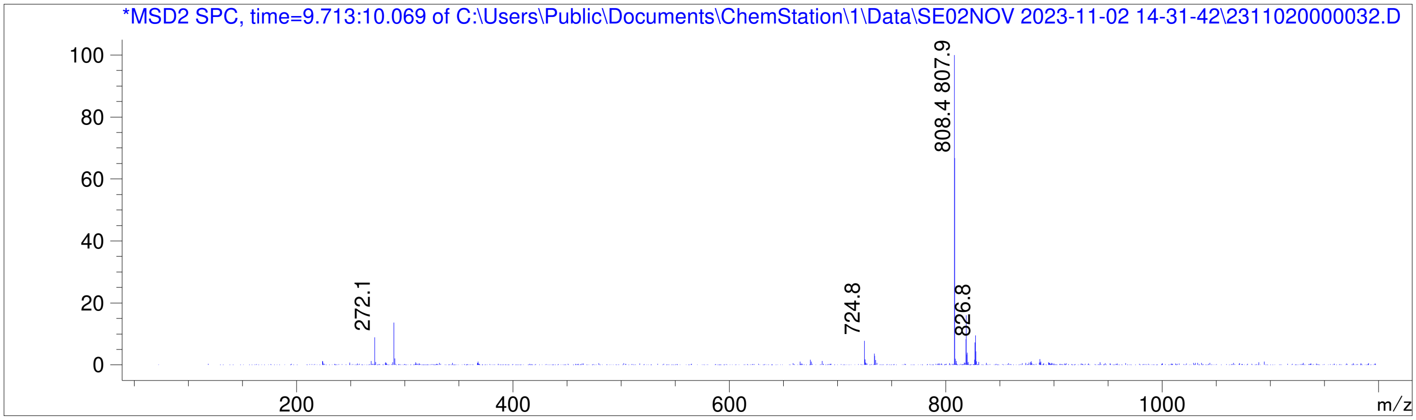
*MSD2 SPC, time=9.713:10.069 of C:\Users\Public\Documents\ChemStation\1\Data\SE02NOV 2023-11-02 14-31-42\231102000032.D
808.4 807.9
272.1
724.8
826.8
m/z

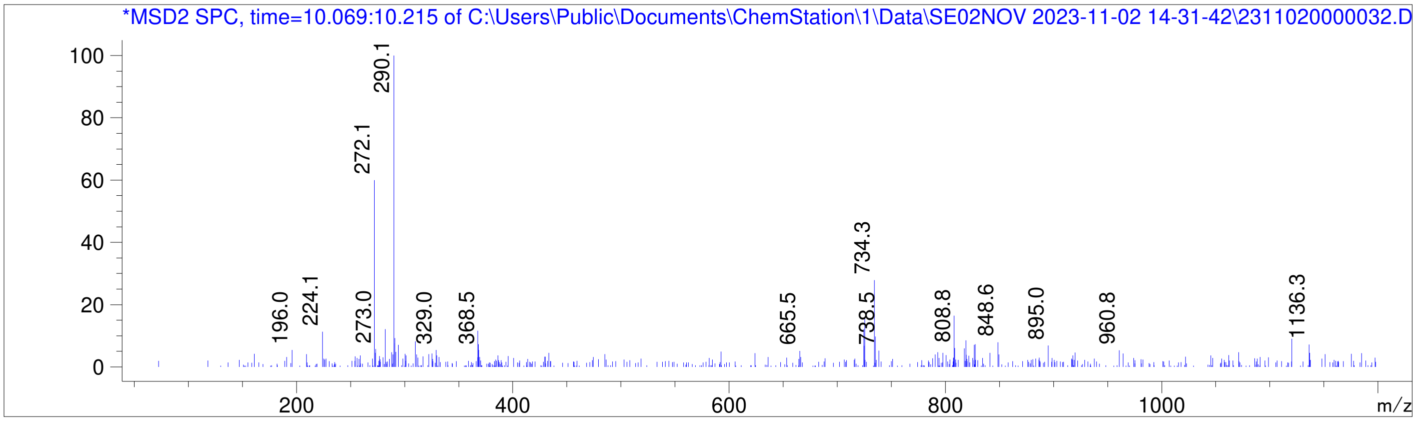
*MSD2 SPC, time=10.069:10.215 of C:\Users\Public\Documents\ChemStation\1\Data\SE02NOV 2023-11-02 14-31-42\2311020000032.D
290.1
272.1
734.3
196.0
224.1
273.0
329.0
368.5
665.5
738.5
808.8
848.6
895.0
960.8
1136.3
m/z
