# Supplementary material for: Astrobiological implications of the stability and reactivity of peptide nucleic acid (PNA) in concentrated sulfuric acid
Source: Sci Adv. 2025 Mar 26;11(13):eadr0006. doi: 10.1126/sciadv.adr0006 (PMC11939054; doi:10.1126/sciadv.adr0006)

Data -> C:\Users\Public\Documents\ChemStation\1\Data\SE18OCT 2023-10-18 09-07-12\ ->
Sample-> CPT22010446-19-D1-50deg-24h

Injection Date	:	Wed, 18. Oct. 2023	Seq Line	:	4
			Location	:	46
			Inj. Vol.	:	2 µl

Acq. Method : C:\Users\Public\Documents\ChemStation\1\Data\SE18OCT 2023-10-18 09-07-12\22010446C LCMS-6#.M
Analysis Method : C:\Users\Public\Documents\ChemStation\1\Data\SE18OCT 2023-10-18 09-07-12\22010446C LCMS-6#.M (Sequence Method)
Waters XBridge BEH Amide (4.6 x 150 mm, 2.5 µm); PN# 186006726
Mobile Phase A: 20mM Ammonium Acetate (aq) pH 8.2
Mobile Phase B: AcN
Mobile Phase A / Mobile Phase B: 5/95 (0 min) --> (10 min) --> 60/40 (5 min); Flow: 1.0 ml/min; MSD1 = positive; MSD2 = negative

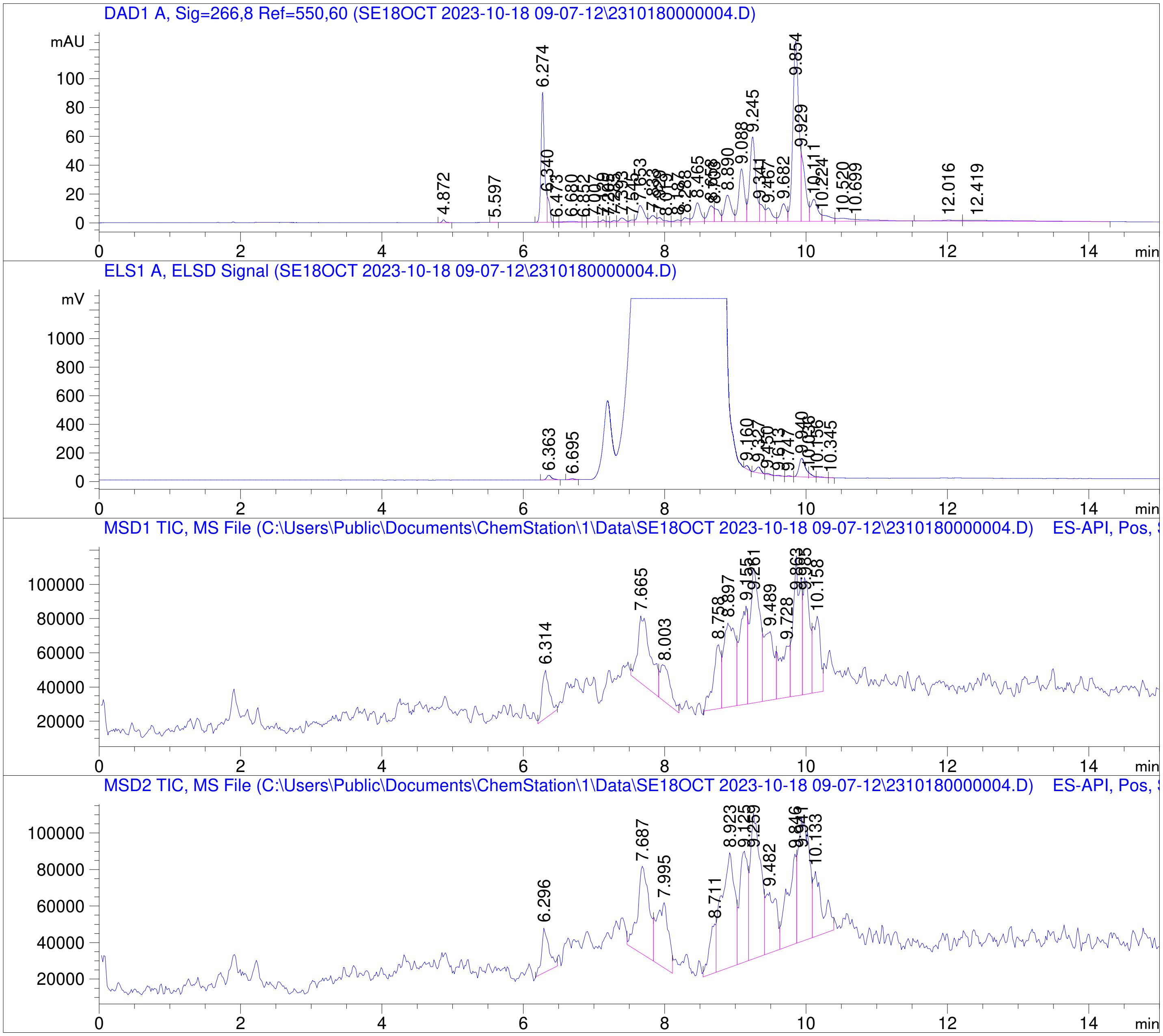

DAD1 A, Sig=266,8 Ref=550,60

Peak #	Ret. Time [min]	Area [mV *s]	Area %
1	4.872	5.447	0.197
2	5.597	0.376	0.014
3	6.274	309.569	11.169
4	6.340	41.122	1.484
5	6.473	0.884	0.032
6	6.680	8.401	0.303
7	6.852	0.798	0.029
8	7.007	2.466	0.089
9	7.129	5.328	0.192
10	7.205	1.281	0.046
11	7.285	3.692	0.133
12	7.393	15.405	0.556
13	7.545	5.997	0.216
14	7.653	71.509	2.580
15	7.833	25.132	0.907
16	7.929	13.476	0.486
17	8.019	3.491	0.126
18	8.187	6.827	0.246
19	8.288	16.003	0.577
20	8.465	82.045	2.960
21	8.658	64.170	2.315
22	8.703	45.233	1.632
23	8.890	124.195	4.481
24	9.088	206.534	7.452
25	9.245	346.793	12.512
26	9.341	52.679	1.901
27	9.467	58.310	2.104
28	9.682	73.001	2.634
29	9.854	735.672	26.543
30	9.929	195.889	7.068
31	10.111	107.893	3.893
32	10.224	36.394	1.313
33	10.520	30.241	1.091
34	10.699	24.755	0.893
35	12.016	13.541	0.489
36	12.419	37.079	1.338

ELS1 A, ELSD Signal

Peak #	Ret. Time [min]	Area [mV *s]	Area %
1	6.363	154.263	10.602
2	6.695	30.703	2.110
3	9.160	82.619	5.678
4	9.327	194.527	13.369
5	9.450	31.440	2.161
6	9.613	15.278	1.050
7	9.747	15.967	1.097
8	9.940	795.968	54.703
9	10.036	98.037	6.738
10	10.156	30.969	2.128
11	10.345	5.302	0.364

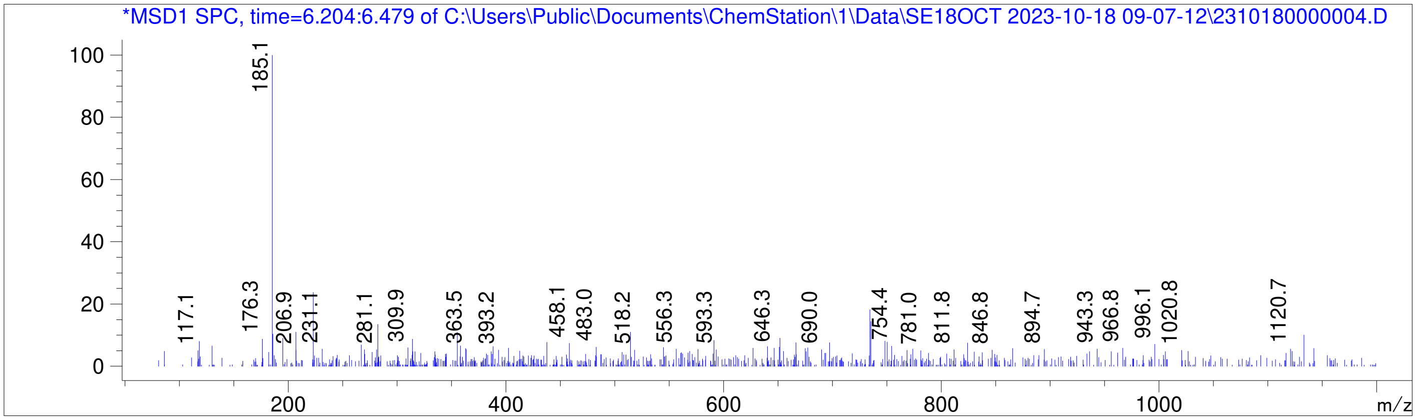
*MSD1 SPC, time=6.204:6.479 of C:\Users\Public\Documents\ChemStation\1\Data\SE18OCT 2023-10-18 09-07-12\2310180000004.D
185.1
117.1
176.3
206.9
231.1
281.1
309.9
363.5
393.2
458.1
483.0
518.2
556.3
593.3
646.3
690.0
754.4
781.0
811.8
846.8
894.7
943.3
966.8
996.1
1020.8
1120.7
m/z

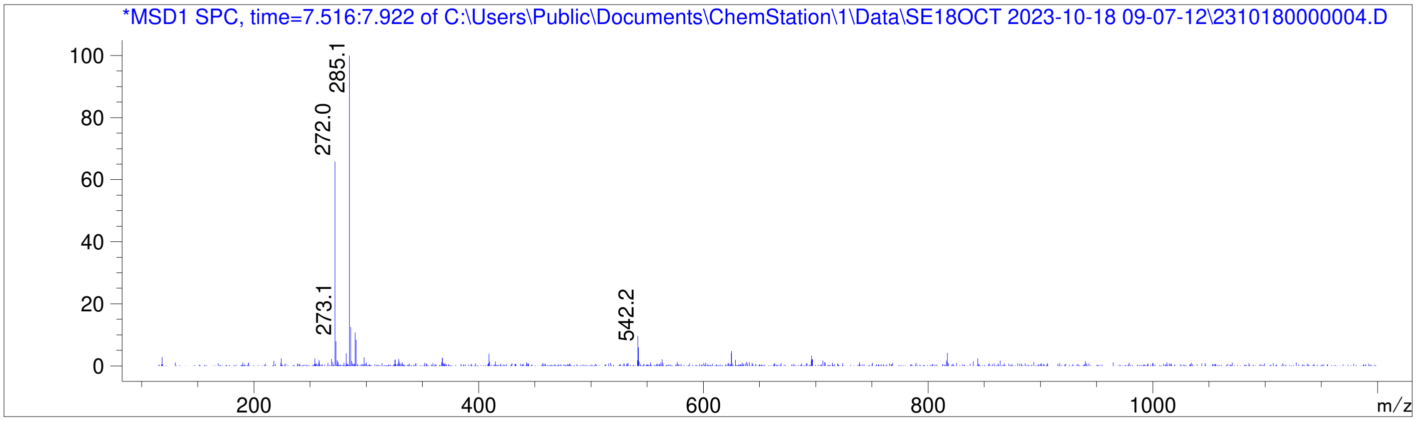
*MSD1 SPC, time=7.516:7.922 of C:\Users\Public\Documents\ChemStation\1\Data\SE18OCT 2023-10-18 09-07-12\2310180000004.D
285.1
272.0
273.1
542.2
m/z

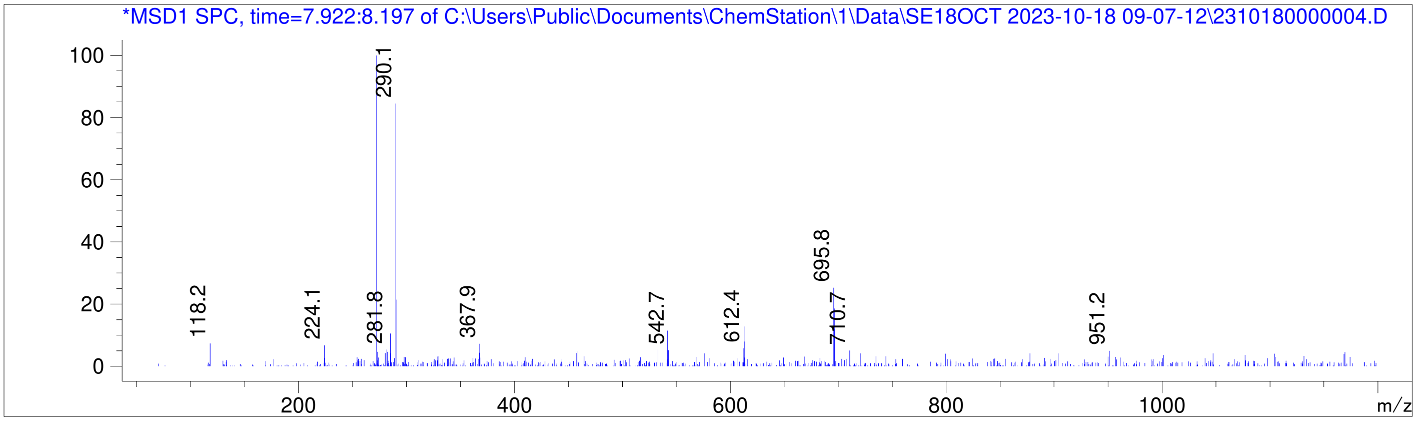
*MSD1 SPC, time=7.922:8.197 of C:\Users\Public\Documents\ChemStation\1\Data\SE18OCT 2023-10-18 09-07-12\2310180000004.D
290.1
695.8
118.2
224.1
281.8
367.9
542.7
612.4
710.7
951.2
m/z

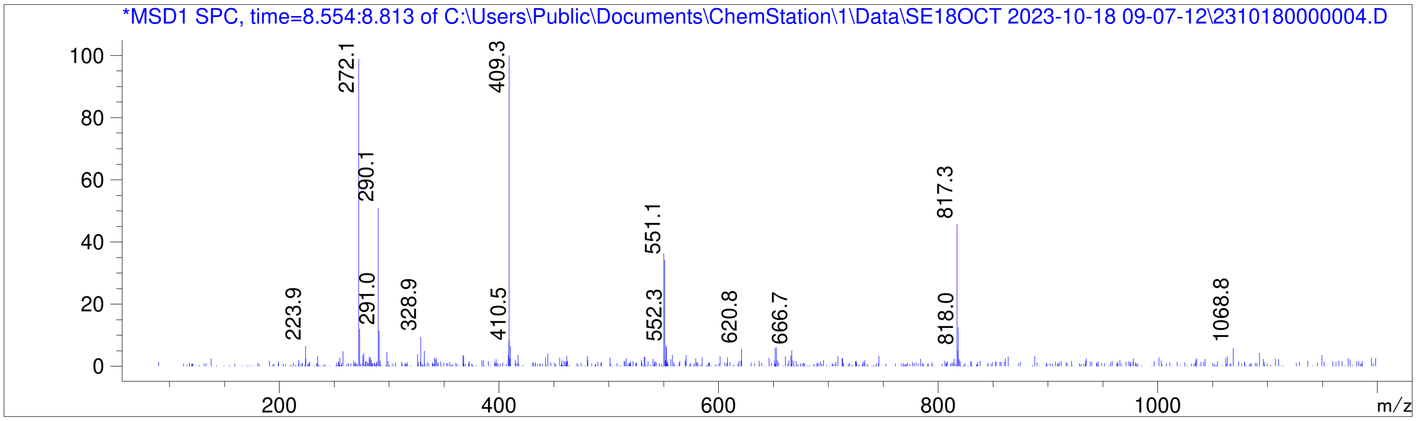
*MSD1 SPC, time=8.554:8.813 of C:\Users\Public\Documents\ChemStation\1\Data\SE18OCT 2023-10-18 09-07-12\2310180000004.D
272.1
409.3
290.1
817.3
551.1
223.9
291.0
328.9
410.5
552.3
620.8
666.7
818.0
1068.8
m/z

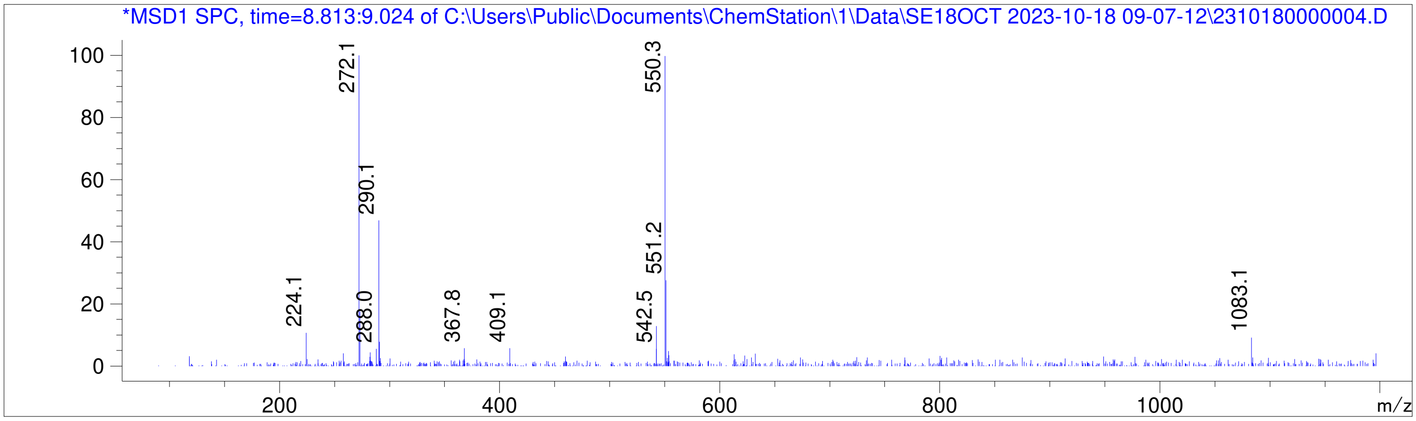
*MSD1 SPC, time=8.813:9.024 of C:\Users\Public\Documents\ChemStation\1\Data\SE18OCT 2023-10-18 09-07-12\2310180000004.D
272.1
550.3
290.1
551.2
224.1
288.0
367.8
409.1
542.5
1083.1
m/z

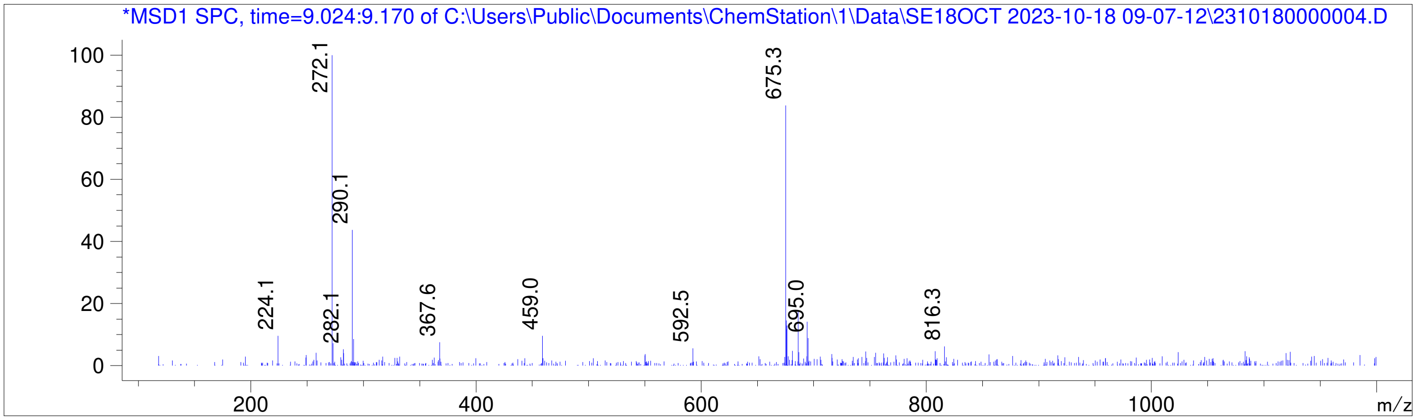
*MSD1 SPC, time=9.024:9.170 of C:\Users\Public\Documents\ChemStation\1\Data\SE18OCT 2023-10-18 09-07-12\2310180000004.D
272.1
675.3
290.1
224.1
282.1
367.6
459.0
592.5
695.0
816.3
m/z

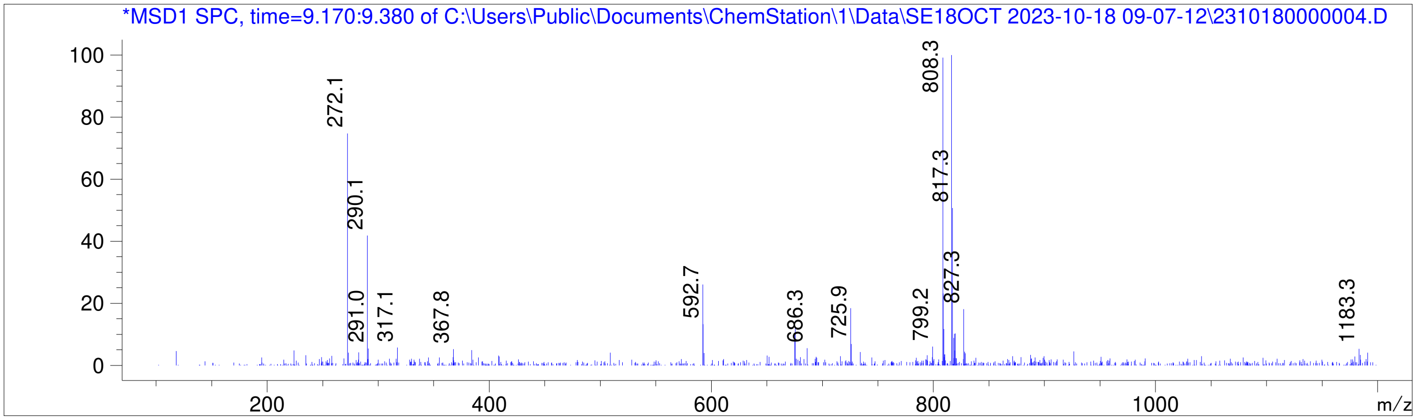
*MSD1 SPC, time=9.170:9.380 of C:\Users\Public\Documents\ChemStation\1\Data\SE18OCT 2023-10-18 09-07-12\2310180000004.D
808.3
272.1
817.3
290.1
827.3
592.7
291.0
317.1
367.8
686.3
725.9
799.2
1183.3
m/z

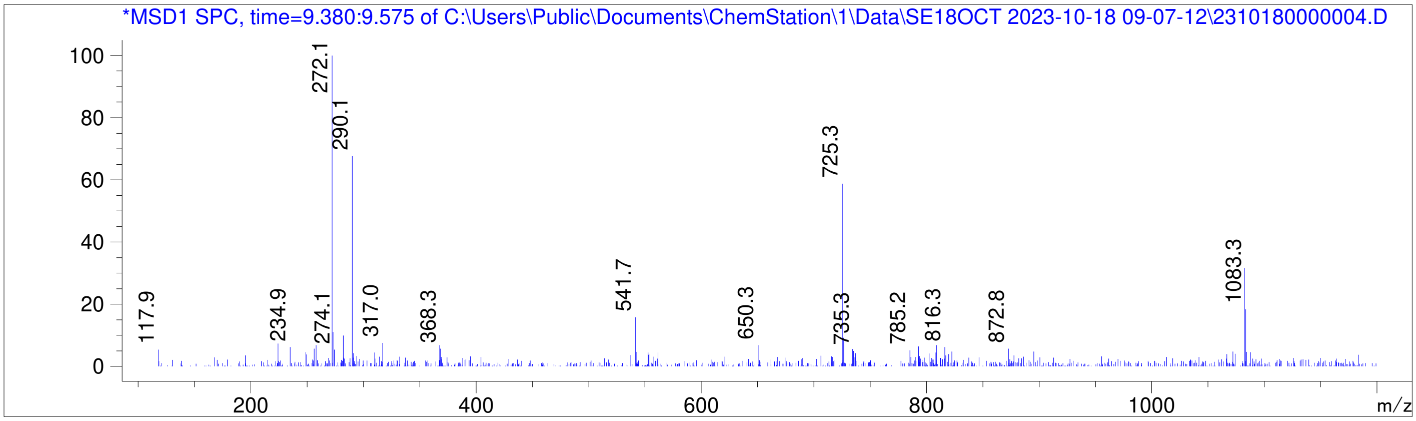
*MSD1 SPC, time=9.380:9.575 of C:\Users\Public\Documents\ChemStation\1\Data\SE18OCT 2023-10-18 09-07-12\2310180000004.D
272.1
290.1
725.3
1083.3
541.7
117.9
234.9
274.1
317.0
368.3
650.3
735.3
785.2
816.3
872.8
m/z

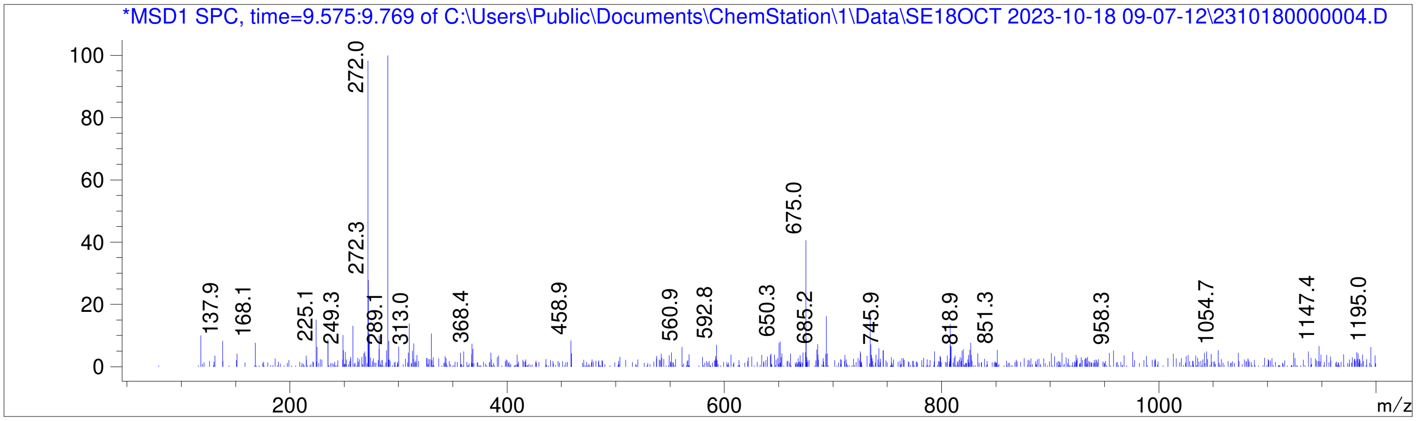
*MSD1 SPC, time=9.575:9.769 of C:\Users\Public\Documents\ChemStation\1\Data\SE18OCT 2023-10-18 09-07-12\2310180000004.D
272.0
675.0
272.3
137.9
168.1
225.1
249.3
289.1
313.0
368.4
458.9
560.9
592.8
650.3
685.2
745.9
818.9
851.3
958.3
1054.7
1147.4
1195.0
m/z

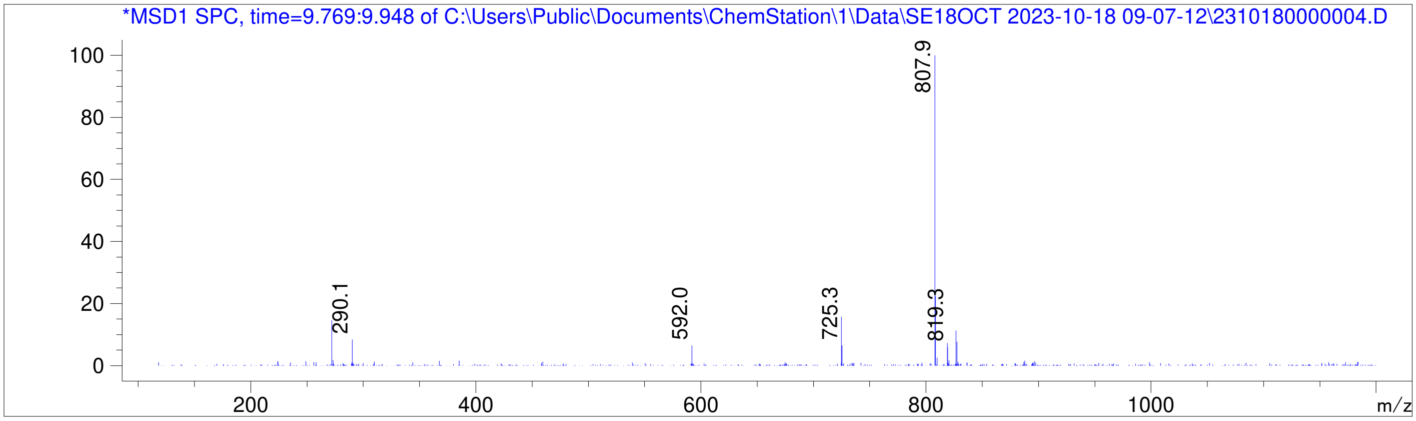
*MSD1 SPC, time=9.769:9.948 of C:\Users\Public\Documents\ChemStation\1\Data\SE18OCT 2023-10-18 09-07-12\2310180000004.D
807.9
290.1
592.0
725.3
819.3
m/z

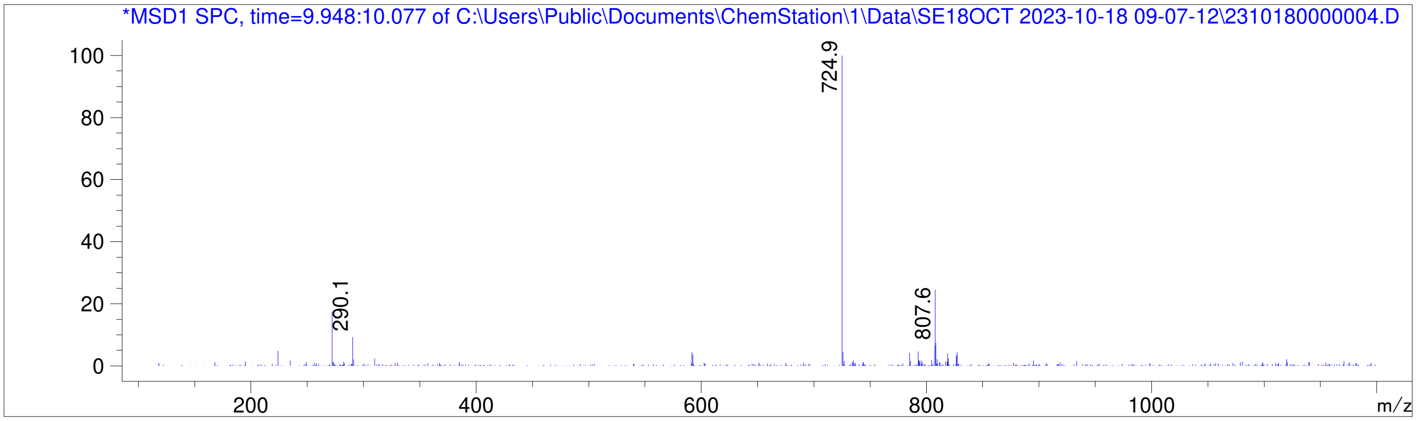
*MSD1 SPC, time=9.948:10.077 of C:\Users\Public\Documents\ChemStation\1\Data\SE18OCT 2023-10-18 09-07-12\2310180000004.D
100
80
60
40
20
0
724.9
290.1
807.6
200
400
600
800
1000
m/z

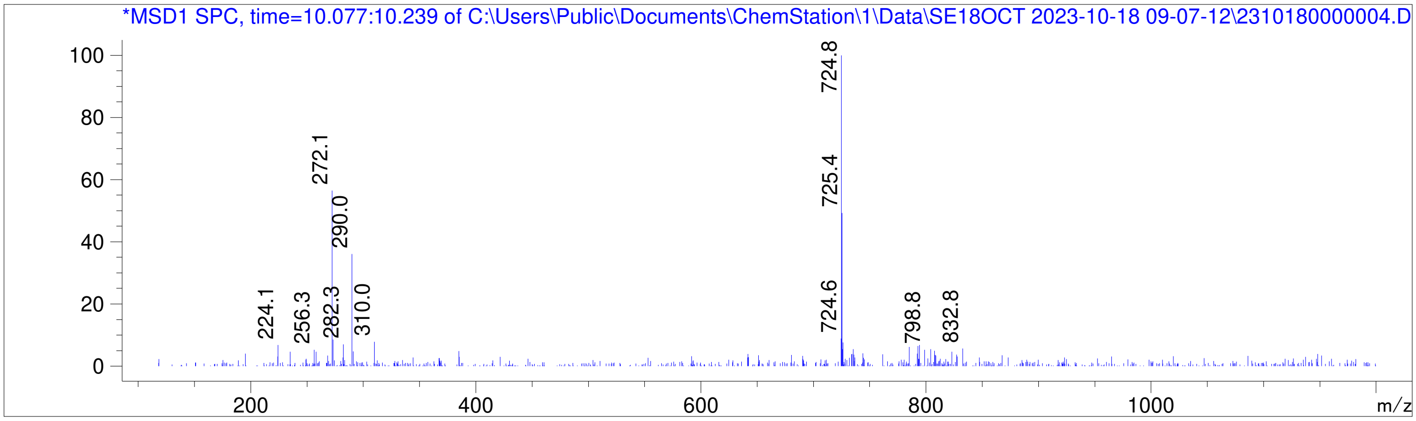
*MSD1 SPC, time=10.077:10.239 of C:\Users\Public\Documents\ChemStation\1\Data\SE18OCT 2023-10-18 09-07-12\2310180000004.D
100
80
60
40
20
0
724.8
725.4
272.1
290.0
224.1
256.3
282.3
310.0
724.6
798.8
832.8
200
400
600
800
1000
m/z

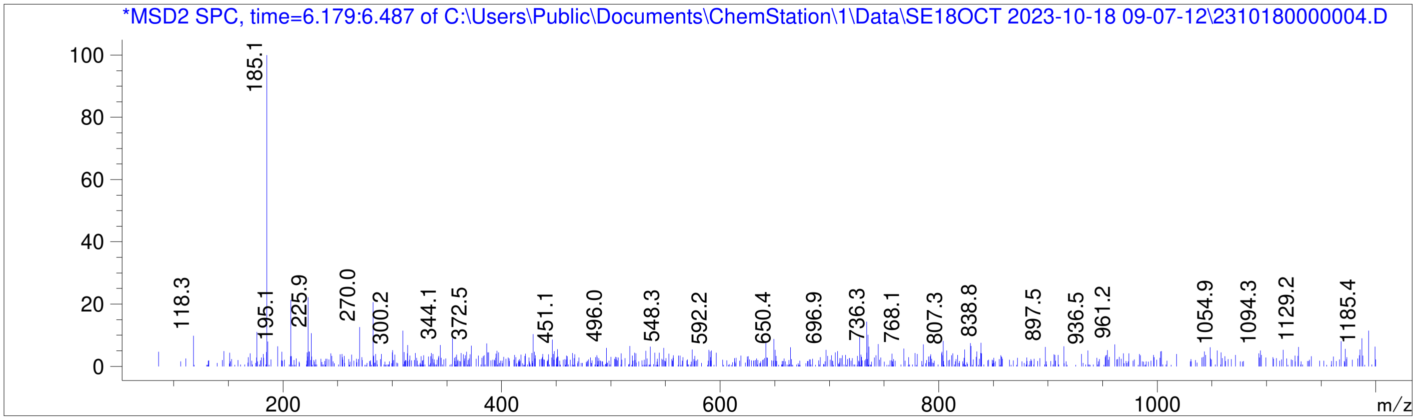
*MSD2 SPC, time=6.179:6.487 of C:\Users\Public\Documents\ChemStation\1\Data\SE18OCT 2023-10-18 09-07-12\2310180000004.D
185.1
118.3
195.1
225.9
270.0
300.2
344.1
372.5
451.1
496.0
548.3
592.2
650.4
696.9
736.3
768.1
807.3
838.8
897.5
936.5
961.2
1054.9
1094.3
1129.2
1185.4
m/z

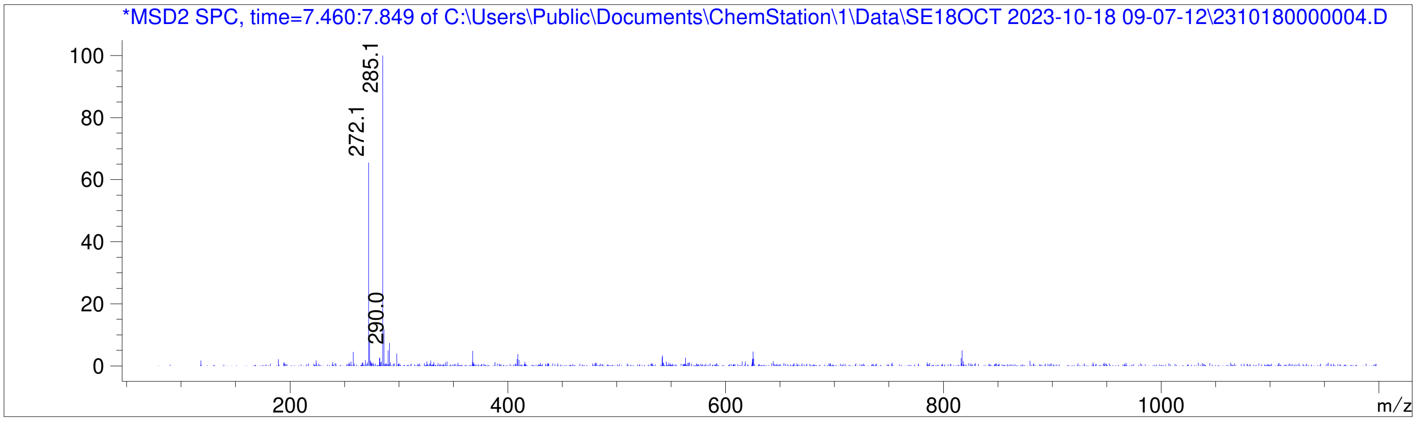
*MSD2 SPC, time=7.460:7.849 of C:\Users\Public\Documents\ChemStation\1\Data\SE18OCT 2023-10-18 09-07-12\2310180000004.D
285.1
272.1
290.0
m/z

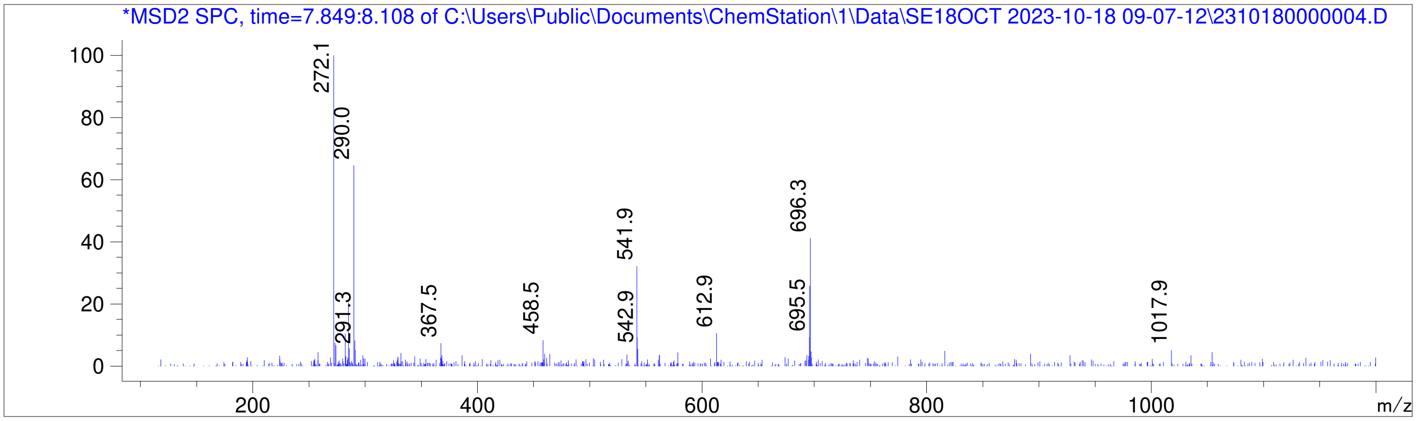
*MSD2 SPC, time=7.849:8.108 of C:\Users\Public\Documents\ChemStation\1\Data\SE18OCT 2023-10-18 09-07-12\2310180000004.D
272.1
290.0
291.3
367.5
458.5
541.9
542.9
612.9
696.3
695.5
1017.9
m/z

*MSD2 SPC, time=8.546:8.724 of C:\Users\Public\Documents\ChemStation\1\Data\SE18OCT 2023-10-18 09-07-12\2310180000004.D

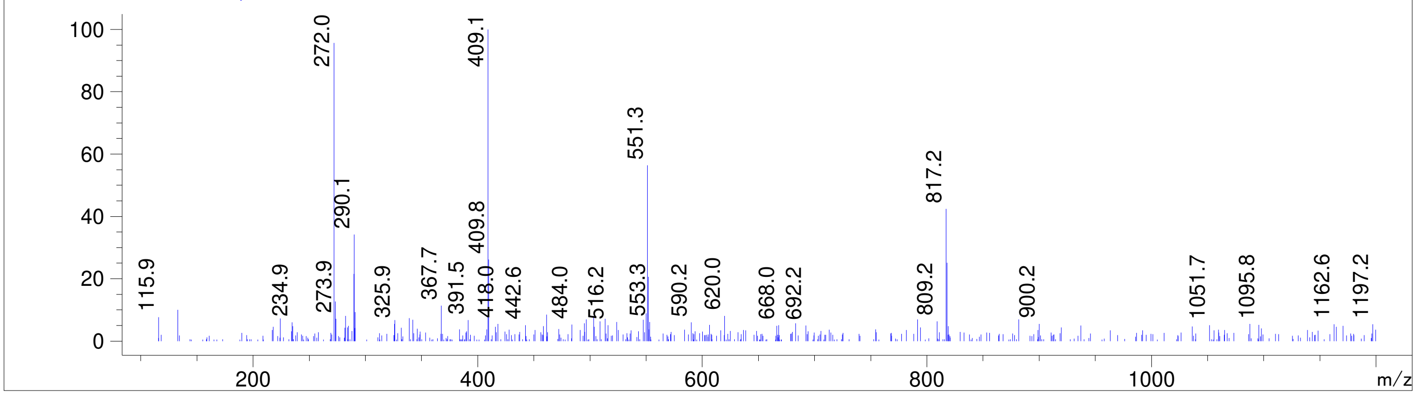

*MSD2 SPC, time=8.724:9.032 of C:\Users\Public\Documents\ChemStation\1\Data\SE18OCT 2023-10-18 09-07-12\2310180000004.D

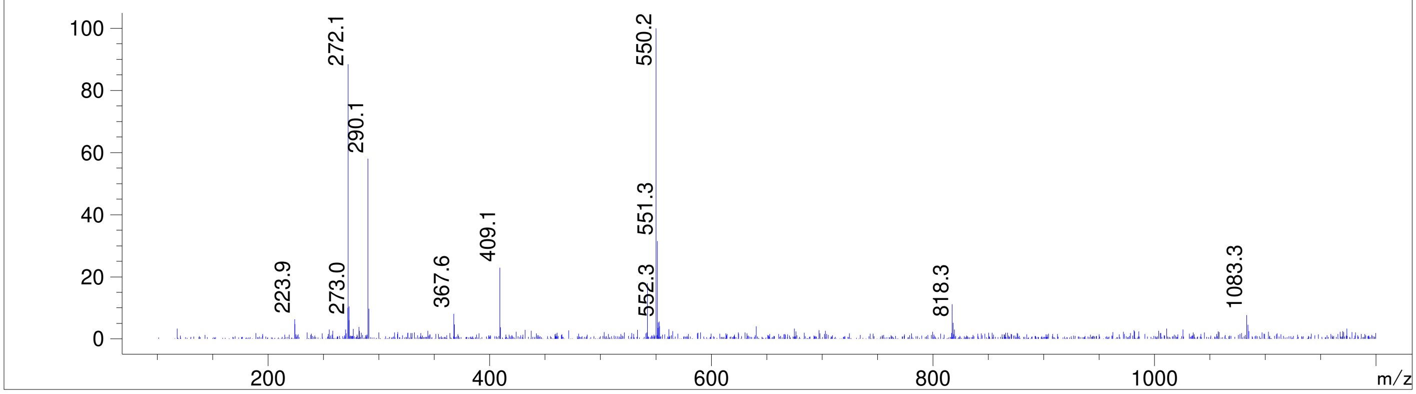

*MSD2 SPC, time=9.032:9.194 of C:\Users\Public\Documents\ChemStation\1\Data\SE18OCT 2023-10-18 09-07-12\2310180000004.D

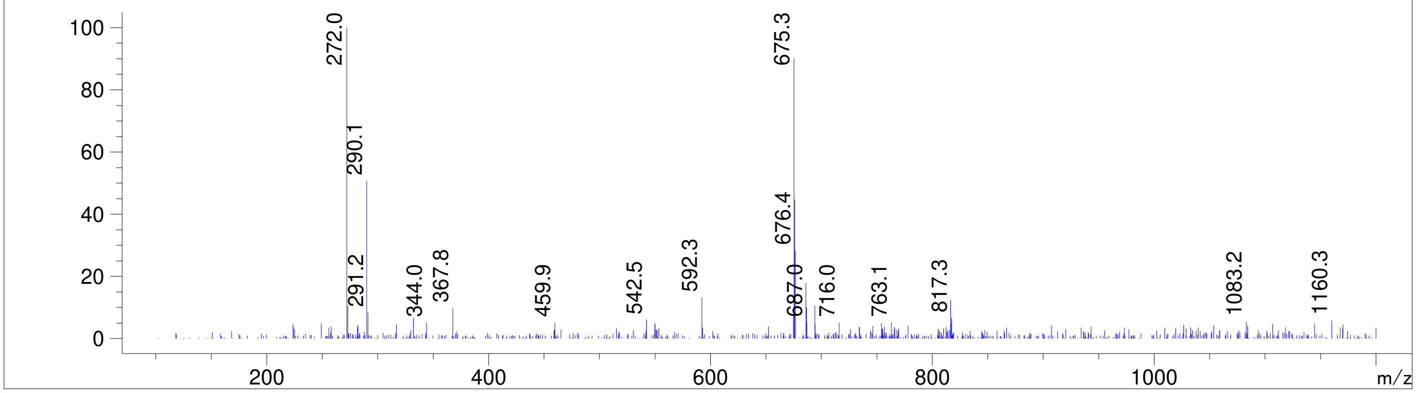

*MSD2 SPC, time=9.194:9.405 of C:\Users\Public\Documents\ChemStation\1\Data\SE18OCT 2023-10-18 09-07-12\2310180000004.D

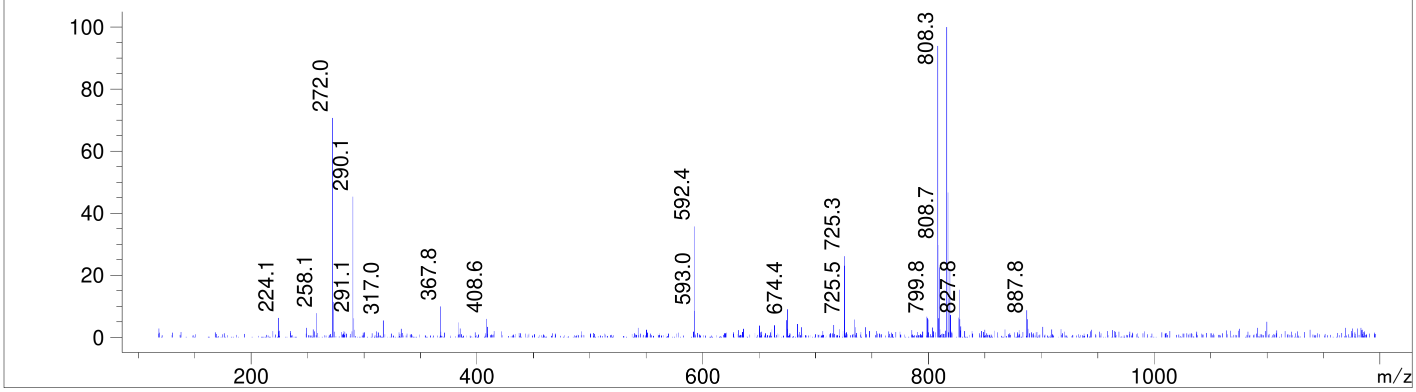

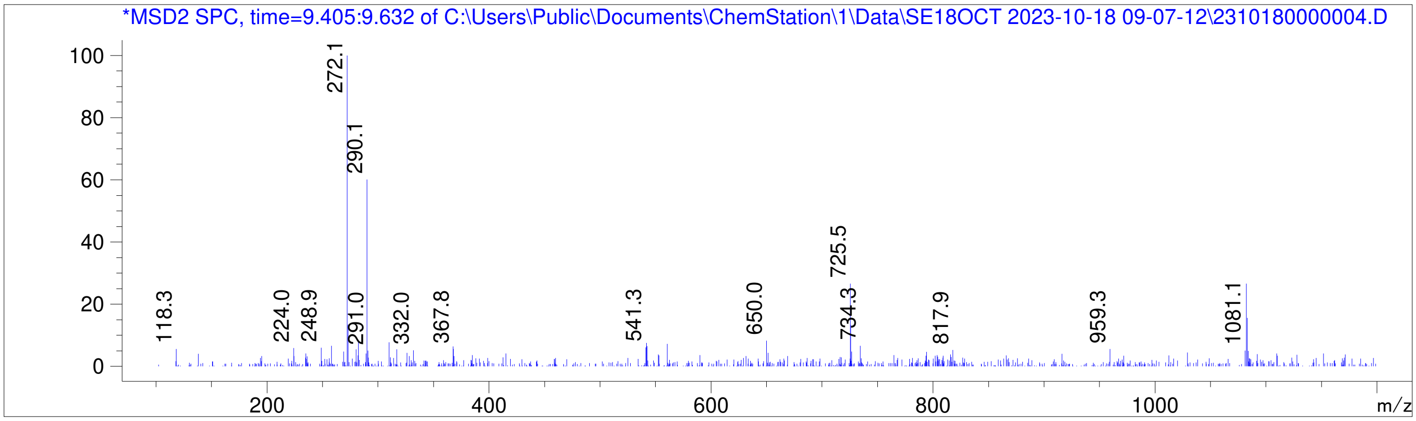
*MSD2 SPC, time=9.405:9.632 of C:\Users\Public\Documents\ChemStation\1\Data\SE18OCT 2023-10-18 09-07-12\2310180000004.D
272.1
290.1
725.5
118.3
224.0
248.9
291.0
332.0
367.8
541.3
650.0
734.3
817.9
959.3
1081.1
m/z

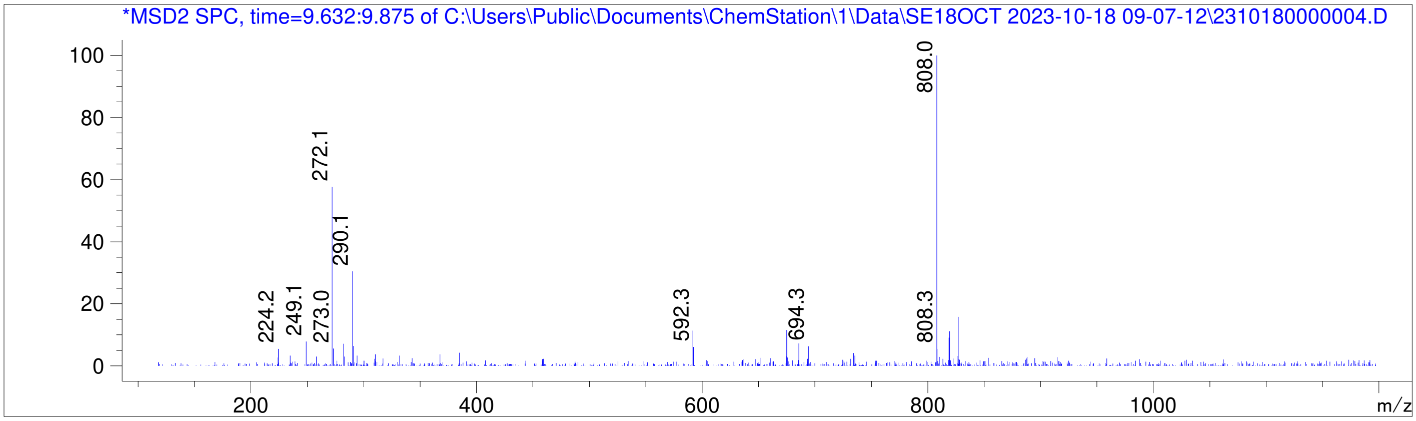
*MSD2 SPC, time=9.632:9.875 of C:\Users\Public\Documents\ChemStation\1\Data\SE18OCT 2023-10-18 09-07-12\2310180000004.D
808.0
272.1
290.1
224.2
249.1
273.0
592.3
694.3
808.3
m/z

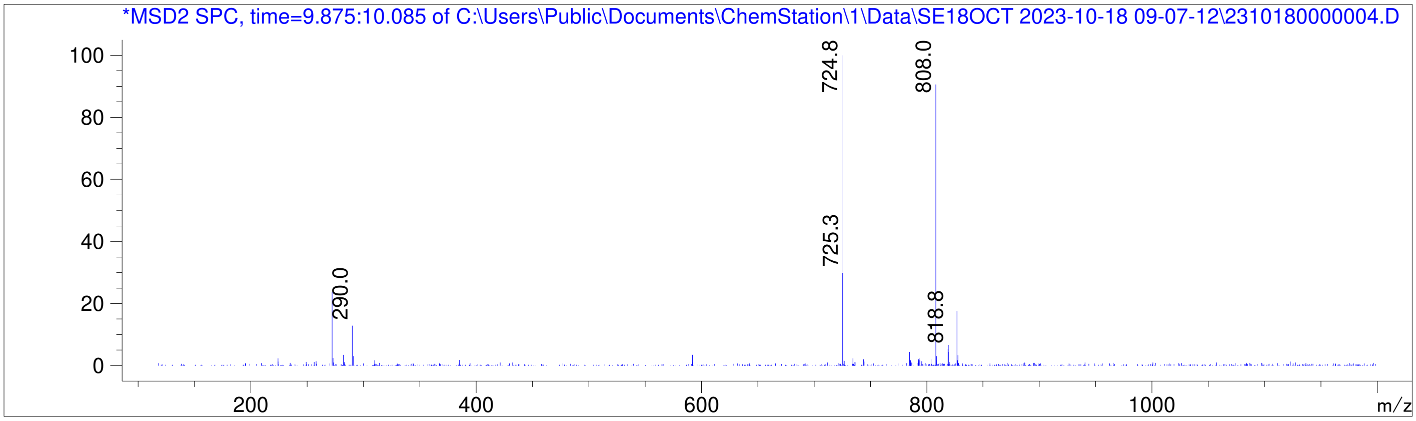
*MSD2 SPC, time=9.875:10.085 of C:\Users\Public\Documents\ChemStation\1\Data\SE18OCT 2023-10-18 09-07-12\2310180000004.D
724.8
808.0
725.3
290.0
818.8
m/z

Sample-> CPT22010446-19-D1-50deg-24h

==

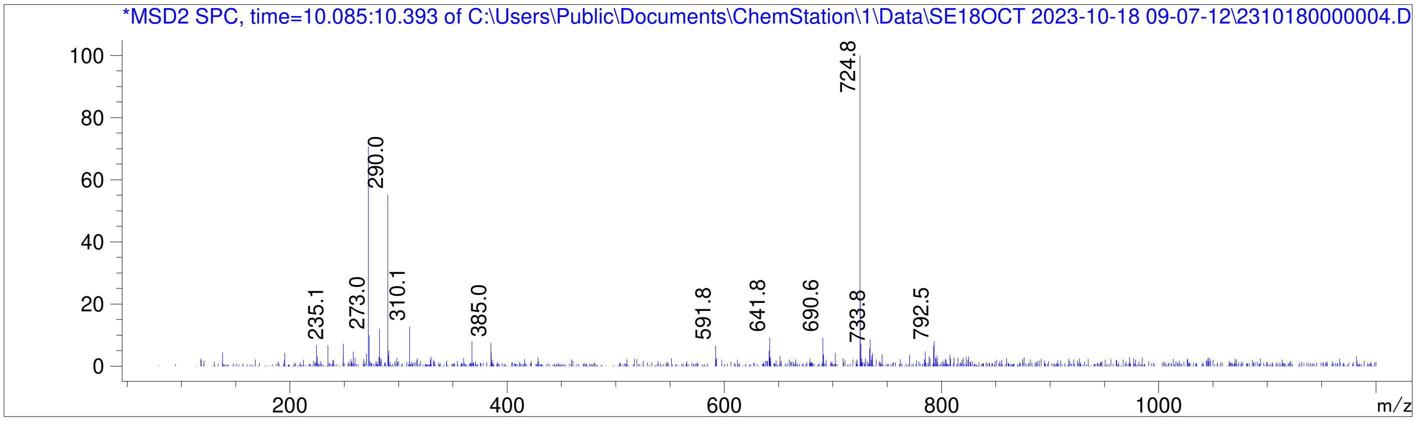

Supplement: Supplementary file 2 — Data S1 and S2 [file sciadv.adr0006_data_s1_and_s2.zip › Supplementary Dataset 1-LCMS DATA/LCMS PNA Hexamers A-T/LCMS T6 50C_80C/50C/24h/CPT22010446-19-D1-50deg-24h.pdf]
