# Supplementary material for: Astrobiological implications of the stability and reactivity of peptide nucleic acid (PNA) in concentrated sulfuric acid
Source: Sci Adv. 2025 Mar 26;11(13):eadr0006. doi: 10.1126/sciadv.adr0006 (PMC11939054; doi:10.1126/sciadv.adr0006)
Supplement: Supplementary file 2 — Data S1 and S2 [file sciadv.adr0006_data_s1_and_s2.zip › Supplementary Dataset 1-LCMS DATA/LCMS PNA Hexamers A-T/LCMS T6 50C_80C/50C/24h/CPT22010446-19-D2-50deg-24h.pdf]

Data -> C:\Users\Public\Documents\ChemStation\1\Data\SE02NOV 2023-11-02 14-31-42\   ->
Sample-> CPT22010446-19-D2-50deg-24h

===

Injection Date	:	Thu, 2. Nov. 2023	Seq Line	:	34
			Location	:	20
			Inj. Vol.	:	2 µl

Acq. Method   : C:\Users\Public\Documents\ChemStation\1\Data\SE02NOV 2023-11-02 14-31-42\22010446C LCMS-6#.M
Analysis Method : C:\Users\Public\Documents\ChemStation\1\Data\SE02NOV 2023-11-02 14-31-42\22010446C LCMS-6#.M (Sequence Method)
Waters XBridge BEH Amide (4.6 x 150 mm, 2.5 µm); PN# 186006726
Mobile Phase A: 20mM Ammonium Acetate (aq) pH 8.2
Mobile Phase B: AcN
Mobile Phase A / Mobile Phase B: 5/95 (0 min) --> (10 min) --> 60/40 (5 min); Flow: 1.0 ml/min; MSD1 = positive; MSD2 = negative

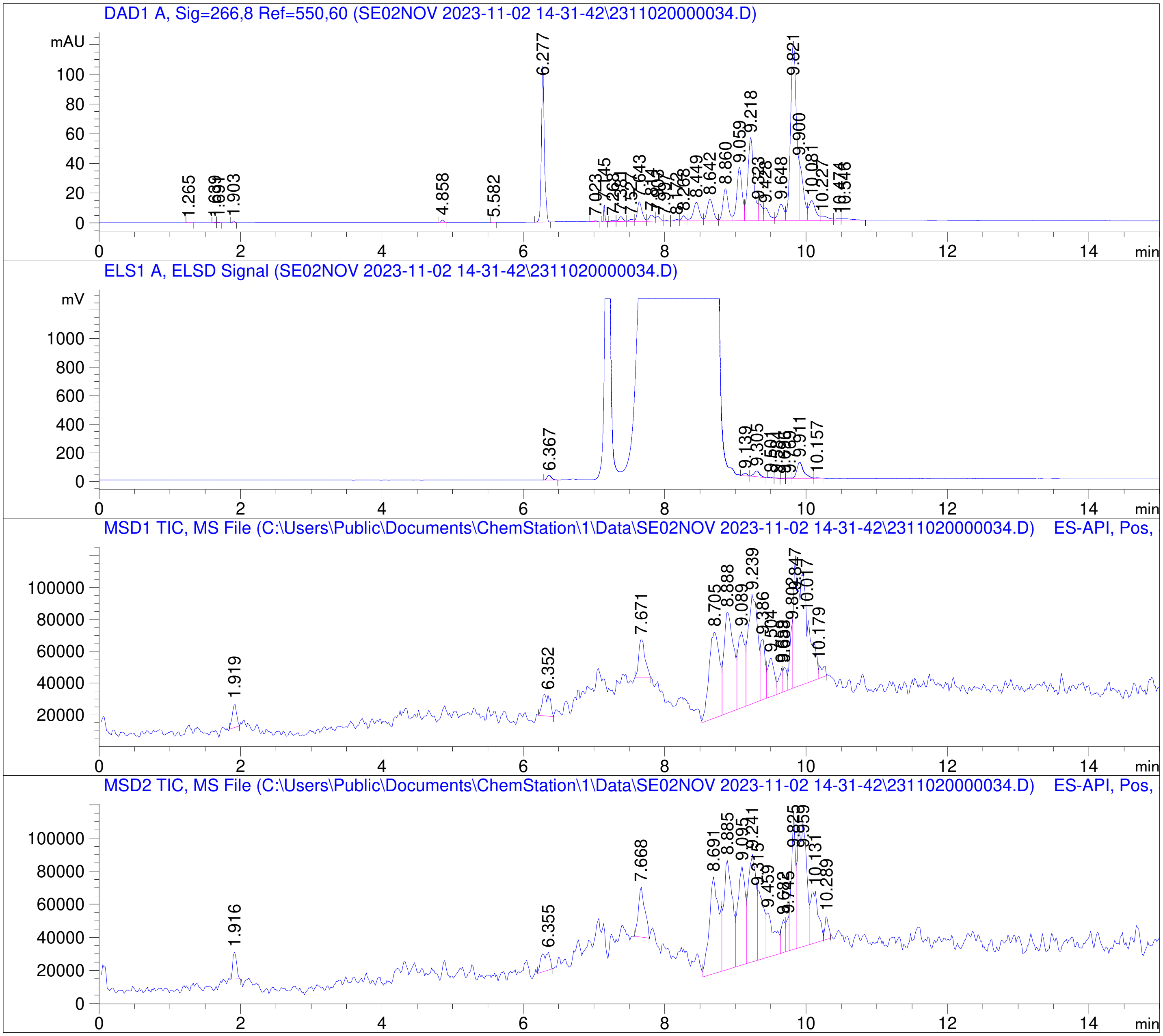

DAD1 A, Sig=266,8 Ref=550,60

Peak #	Ret. Time [min]	Area [mV *s]	Area %
1	1.265	0.314	0.013
2	1.639	0.257	0.011
3	1.691	0.453	0.019
4	1.903	1.744	0.073
5	4.858	4.076	0.169
6	5.582	0.242	0.010
7	6.277	306.100	12.726
8	7.023	1.791	0.074
9	7.145	13.433	0.559
10	7.268	3.548	0.148
11	7.381	13.421	0.558
12	7.527	5.993	0.249
13	7.643	60.630	2.521
14	7.814	20.097	0.836
15	7.903	13.493	0.561
16	7.997	2.248	0.093
17	8.172	3.911	0.163
18	8.268	14.357	0.597
19	8.449	73.541	3.058
20	8.642	84.748	3.524
21	8.860	111.872	4.651
22	9.059	188.849	7.852
23	9.218	335.493	13.949
24	9.323	44.355	1.844
25	9.428	47.109	1.959
26	9.648	64.005	2.661
27	9.821	695.357	28.910
28	9.900	165.823	6.894
29	10.081	92.014	3.826
30	10.227	20.282	0.843
31	10.474	5.759	0.239
32	10.546	9.908	0.412

ELS1 A, ELSD Signal

Peak #	Ret. Time [min]	Area [mV *s]	Area %
1	6.367	135.957	10.807
2	9.139	71.538	5.687
3	9.305	219.262	17.429
4	9.501	14.376	1.143
5	9.584	8.502	0.676
6	9.696	7.600	0.604
7	9.769	21.645	1.721
8	9.911	760.595	60.459
9	10.157	18.549	1.474

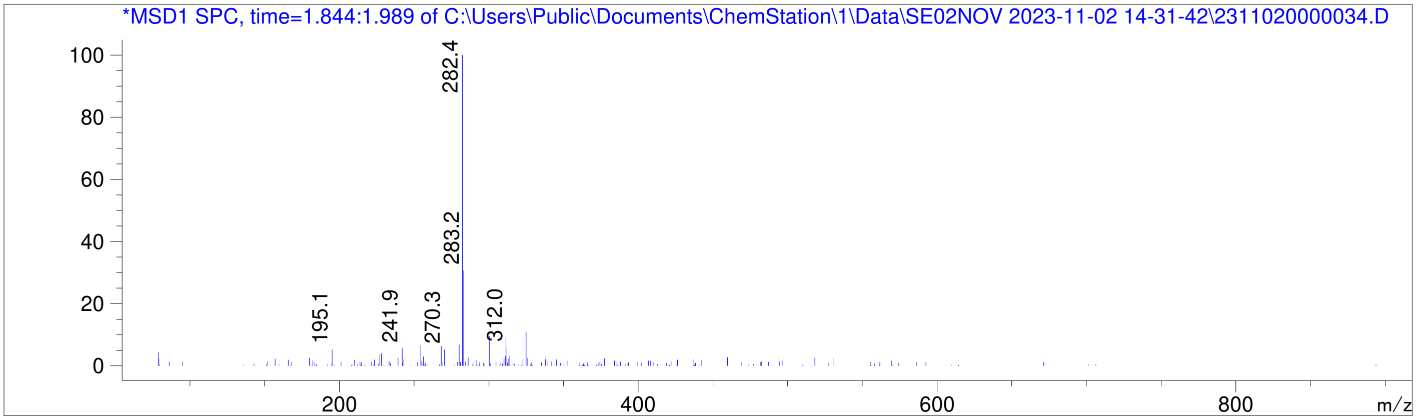
*MSD1 SPC, time=1.844:1.989 of C:\Users\Public\Documents\ChemStation\1\Data\SE02NOV 2023-11-02 14-31-42\2311020000034.D
282.4
283.2
195.1
241.9
270.3
312.0
m/z

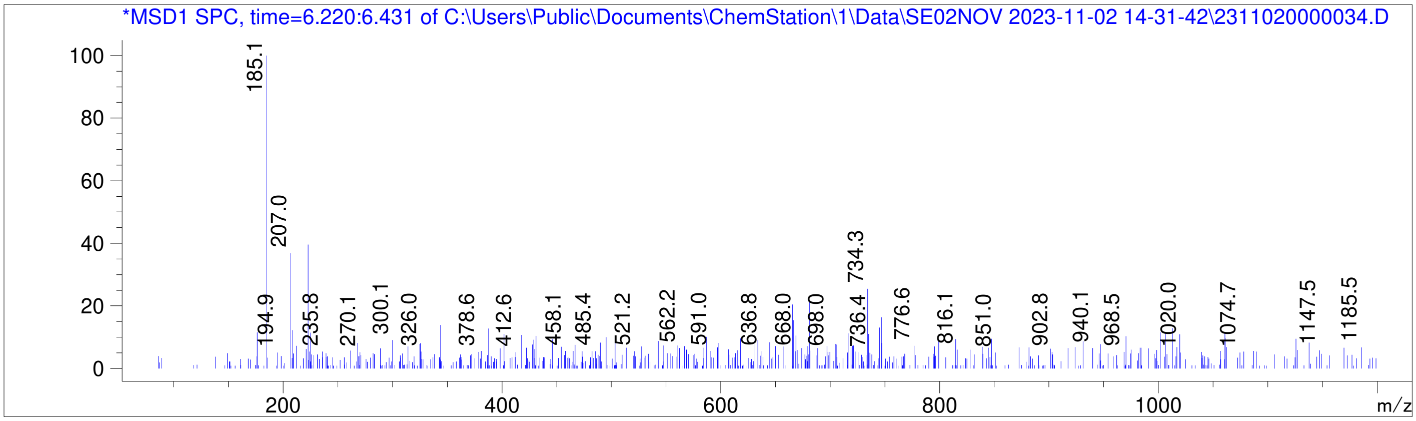
*MSD1 SPC, time=6.220:6.431 of C:\Users\Public\Documents\ChemStation\1\Data\SE02NOV 2023-11-02 14-31-42\2311020000034.D
185.1
207.0
194.9
235.8
270.1
300.1
326.0
378.6
412.6
458.1
485.4
521.2
562.2
591.0
636.8
668.0
698.0
734.3
736.4
776.6
816.1
851.0
902.8
940.1
968.5
1020.0
1074.7
1147.5
1185.5
m/z

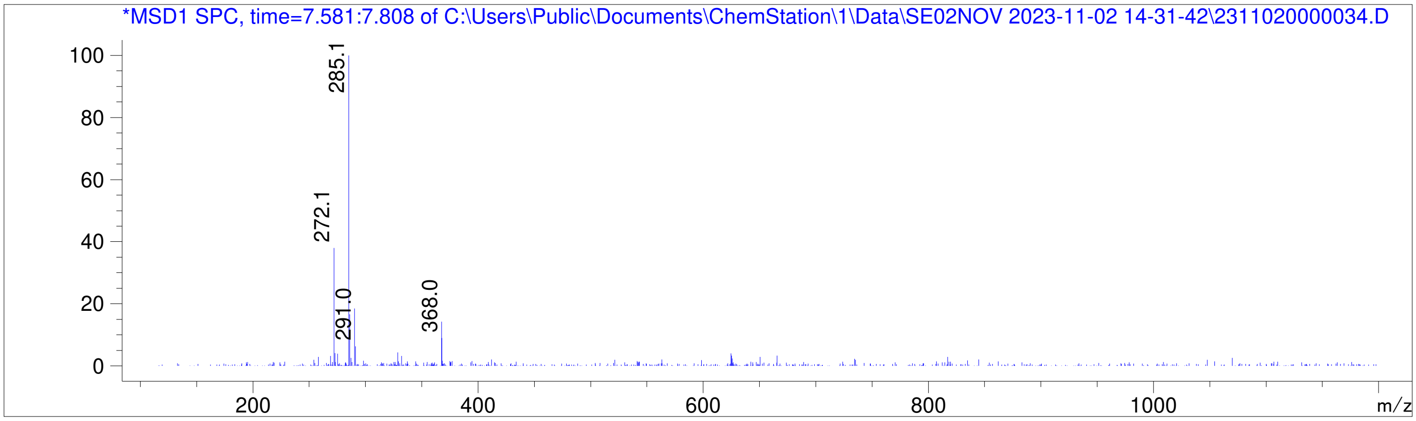
*MSD1 SPC, time=7.581:7.808 of C:\Users\Public\Documents\ChemStation\1\Data\SE02NOV 2023-11-02 14-31-42\2311020000034.D
285.1
272.1
291.0
368.0
m/z

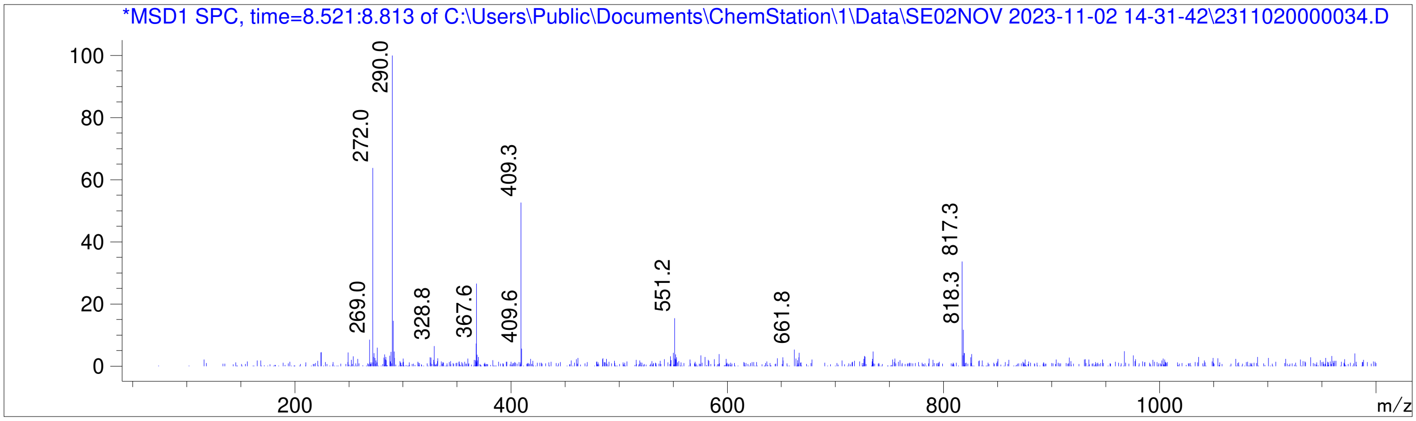
*MSD1 SPC, time=8.521:8.813 of C:\Users\Public\Documents\ChemStation\1\Data\SE02NOV 2023-11-02 14-31-42\231102000034.D
290.0
272.0
409.3
817.3
551.2
269.0
328.8
367.6
409.6
661.8
818.3
m/z

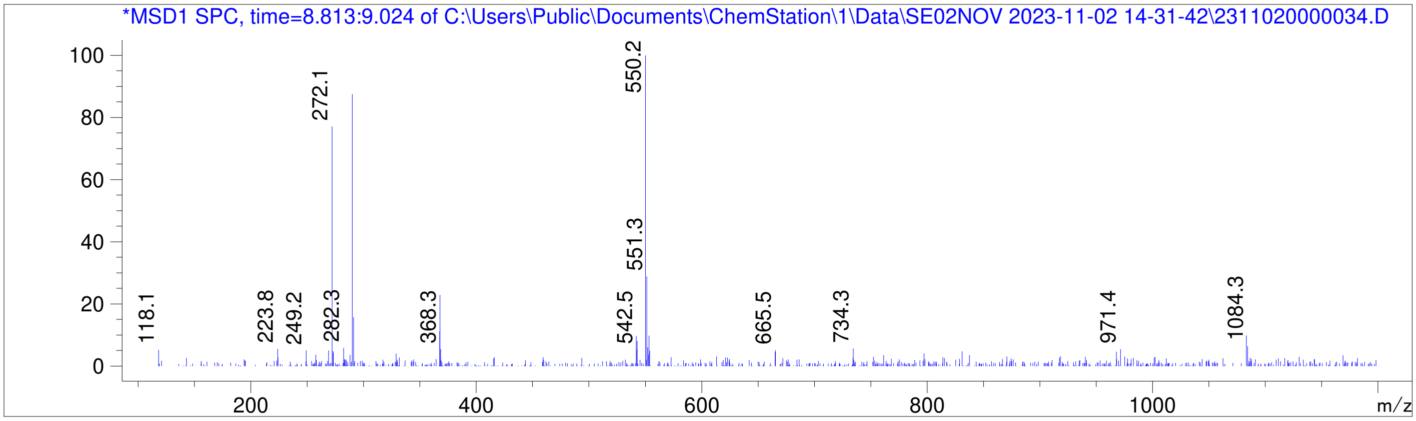
*MSD1 SPC, time=8.813:9.024 of C:\Users\Public\Documents\ChemStation\1\Data\SE02NOV 2023-11-02 14-31-42\231102000034.D
550.2
272.1
551.3
118.1
223.8
249.2
282.3
368.3
542.5
665.5
734.3
971.4
1084.3
m/z

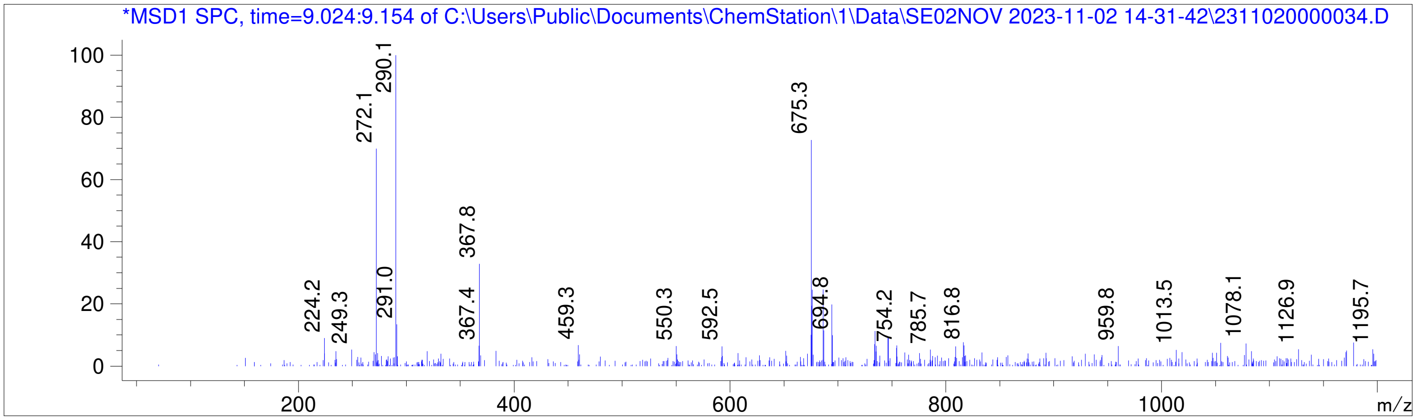
*MSD1 SPC, time=9.024:9.154 of C:\Users\Public\Documents\ChemStation\1\Data\SE02NOV 2023-11-02 14-31-42\231102000034.D
290.1
272.1
675.3
367.8
224.2
249.3
291.0
367.4
459.3
550.3
592.5
694.8
754.2
785.7
816.8
959.8
1013.5
1078.1
1126.9
1195.7
m/z

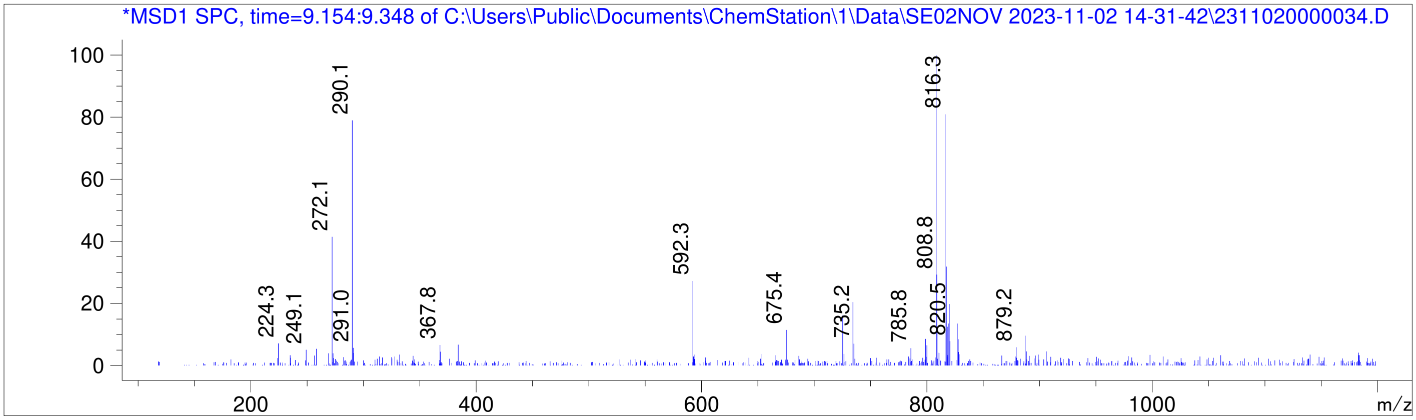
*MSD1 SPC, time=9.154:9.348 of C:\Users\Public\Documents\ChemStation\1\Data\SE02NOV 2023-11-02 14-31-42\231102000034.D
816.3
290.1
272.1
808.8
592.3
224.3
249.1
291.0
367.8
675.4
735.2
785.8
820.5
879.2
m/z

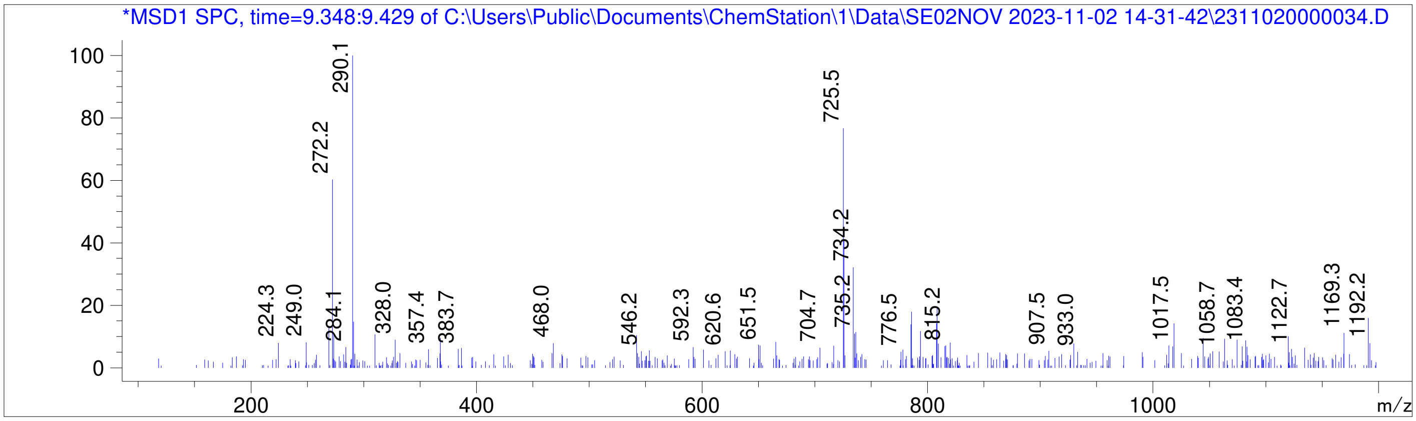
*MSD1 SPC, time=9.348:9.429 of C:\Users\Public\Documents\ChemStation\1\Data\SE02NOV 2023-11-02 14-31-42\2311020000034.D
290.1
725.5
272.2
734.2
224.3
249.0
284.1
328.0
357.4
383.7
468.0
546.2
592.3
620.6
651.5
704.7
735.2
776.5
815.2
907.5
933.0
1017.5
1058.7
1083.4
1122.7
1169.3
1192.2
m/z

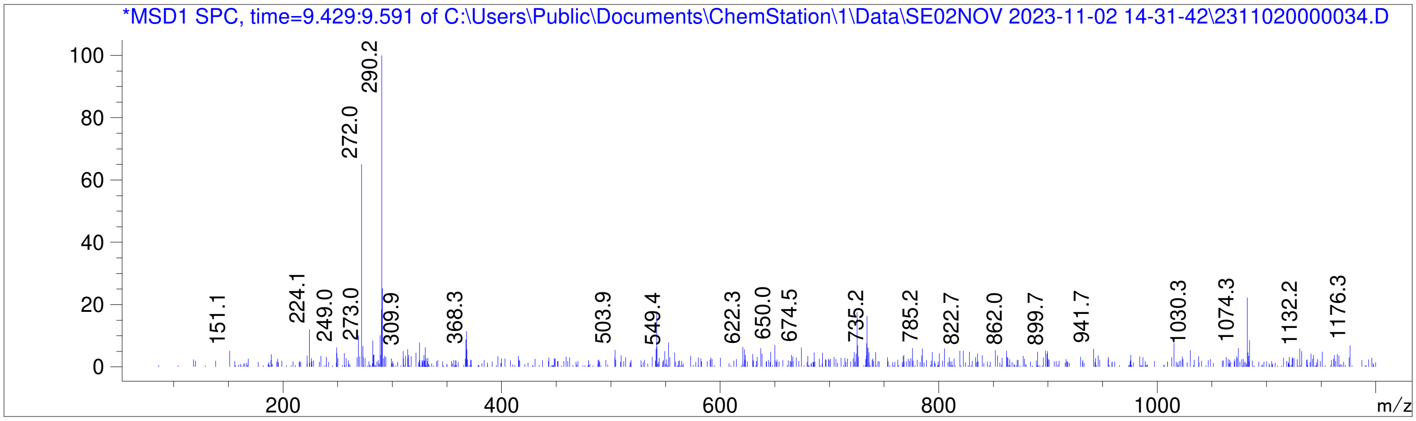
*MSD1 SPC, time=9.429:9.591 of C:\Users\Public\Documents\ChemStation\1\Data\SE02NOV 2023-11-02 14-31-42\2311020000034.D
290.2
272.0
151.1
224.1
249.0
273.0
309.9
368.3
503.9
549.4
622.3
650.0
674.5
735.2
785.2
822.7
862.0
899.7
941.7
1030.3
1074.3
1132.2
1176.3
m/z

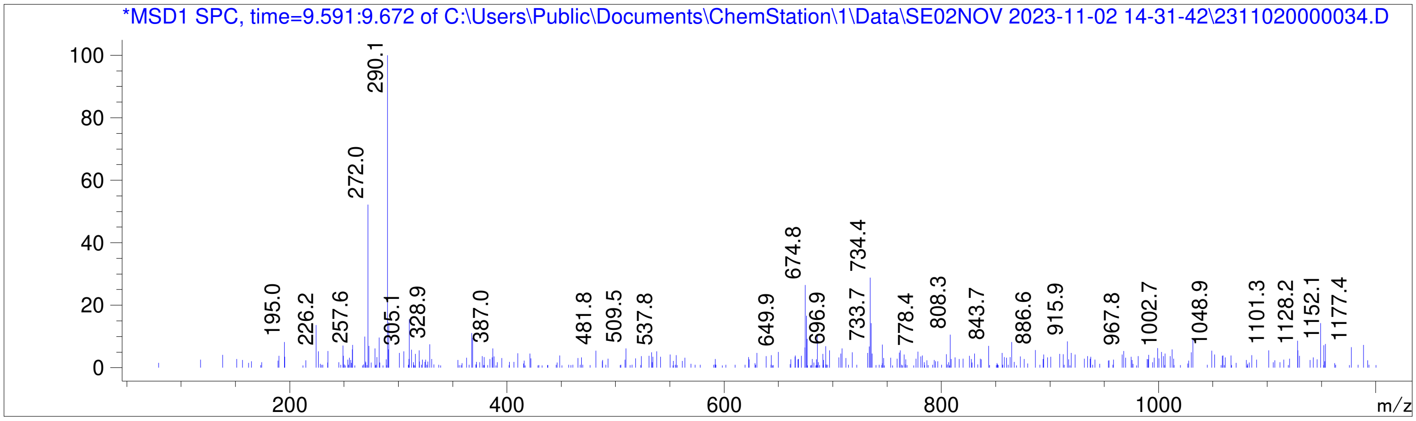
*MSD1 SPC, time=9.591:9.672 of C:\Users\Public\Documents\ChemStation\1\Data\SE02NOV 2023-11-02 14-31-42\2311020000034.D
290.1
272.0
674.8
734.4
195.0
226.2
257.6
305.1
328.9
387.0
481.8
509.5
537.8
649.9
696.9
733.7
778.4
808.3
843.7
886.6
915.9
967.8
1002.7
1048.9
1101.3
1128.2
1152.1
1177.4
m/z

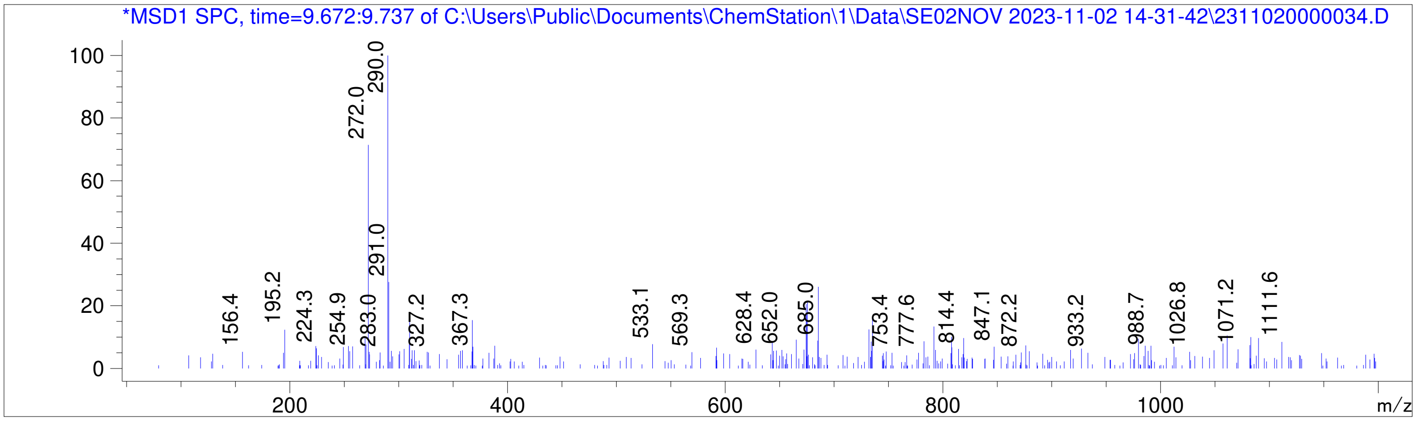
*MSD1 SPC, time=9.672:9.737 of C:\Users\Public\Documents\ChemStation\1\Data\SE02NOV 2023-11-02 14-31-42\2311020000034.D
100
80
60
40
20
0
156.4
195.2
224.3
254.9
272.0
283.0
290.0
291.0
327.2
367.3
533.1
569.3
628.4
652.0
685.0
753.4
777.6
814.4
847.1
872.2
933.2
988.7
1026.8
1071.2
1111.6
200
400
600
800
1000
m/z

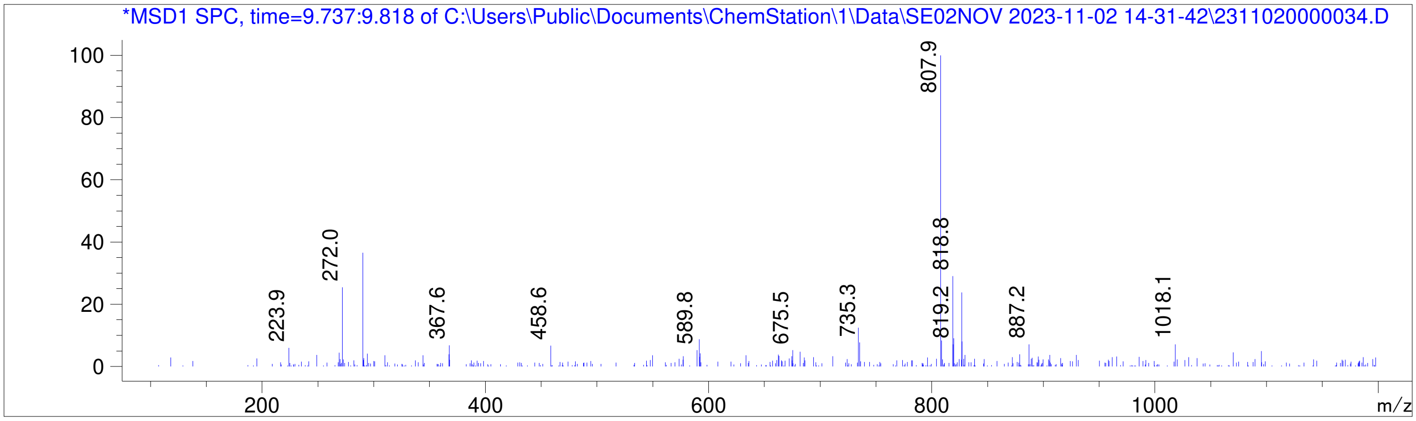
*MSD1 SPC, time=9.737:9.818 of C:\Users\Public\Documents\ChemStation\1\Data\SE02NOV 2023-11-02 14-31-42\2311020000034.D
100
80
60
40
20
0
223.9
272.0
367.6
458.6
589.8
675.5
735.3
807.9
818.8
819.2
887.2
1018.1
200
400
600
800
1000
m/z

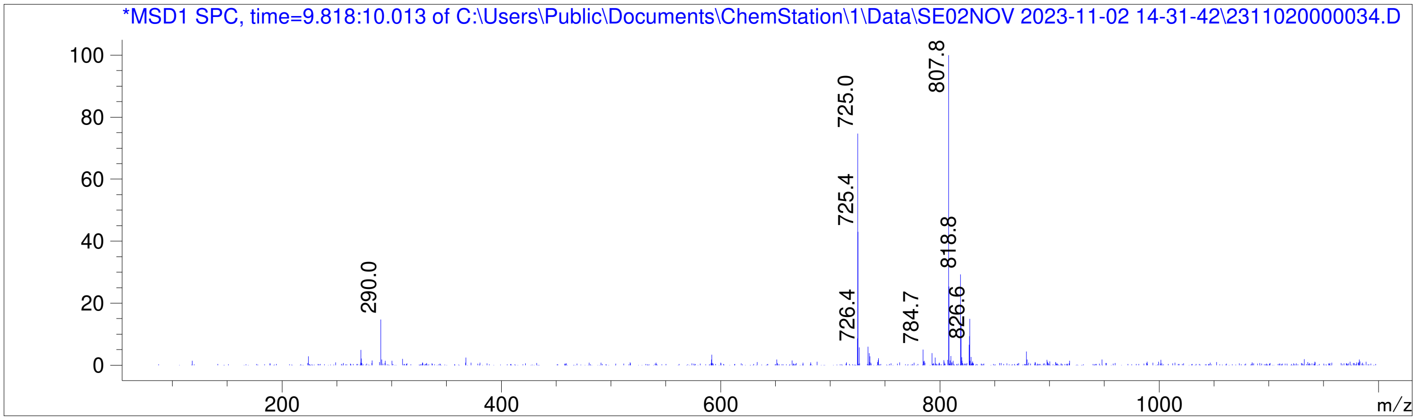
*MSD1 SPC, time=9.818:10.013 of C:\Users\Public\Documents\ChemStation\1\Data\SE02NOV 2023-11-02 14-31-42\2311020000034.D
100
80
60
40
20
0
290.0
725.0
725.4
726.4
784.7
807.8
818.8
826.6
200
400
600
800
1000
m/z

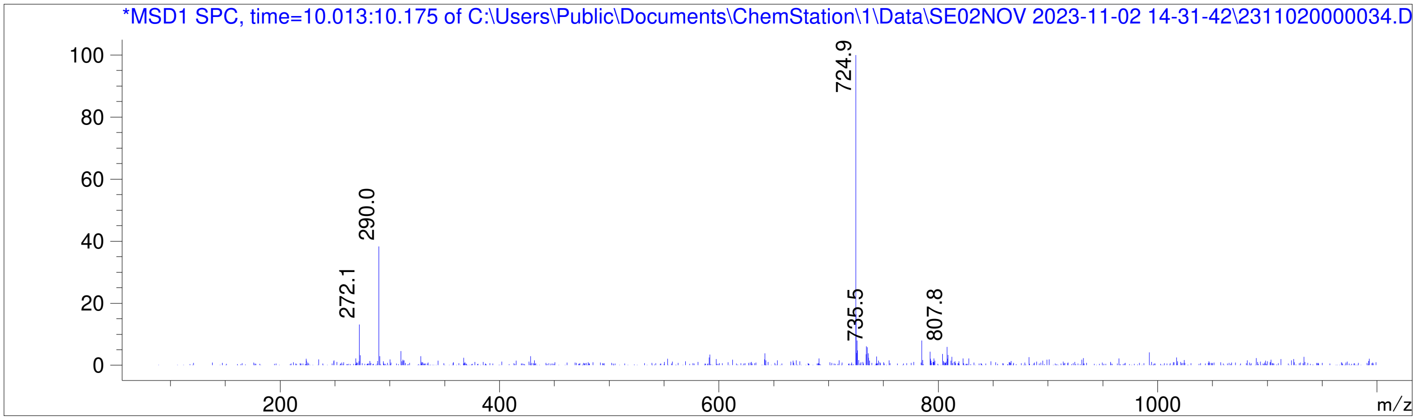
*MSD1 SPC, time=10.013:10.175 of C:\Users\Public\Documents\ChemStation\1\Data\SE02NOV 2023-11-02 14-31-42\2311020000034.D
100
80
60
40
20
0
272.1
290.0
724.9
735.5
807.8
200
400
600
800
1000
m/z

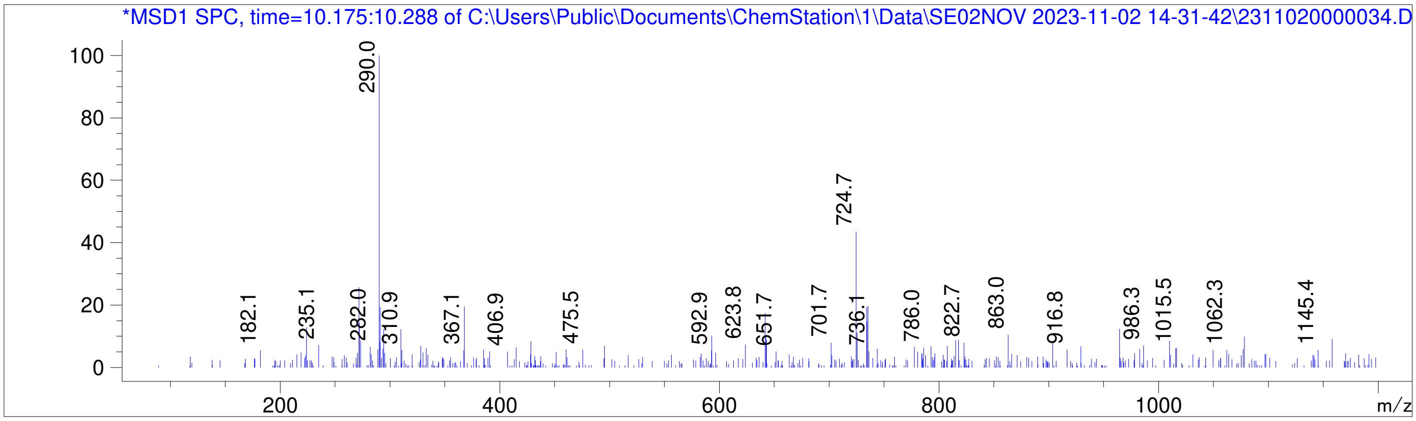
*MSD1 SPC, time=10.175:10.288 of C:\Users\Public\Documents\ChemStation\1\Data\SE02NOV 2023-11-02 14-31-42\2311020000034.D
100
80
60
40
20
0
290.0
724.7
182.1
235.1
282.0
310.9
367.1
406.9
475.5
592.9
623.8
651.7
701.7
736.1
786.0
822.7
863.0
916.8
986.3
1015.5
1062.3
1145.4
200
400
600
800
1000
m/z

==

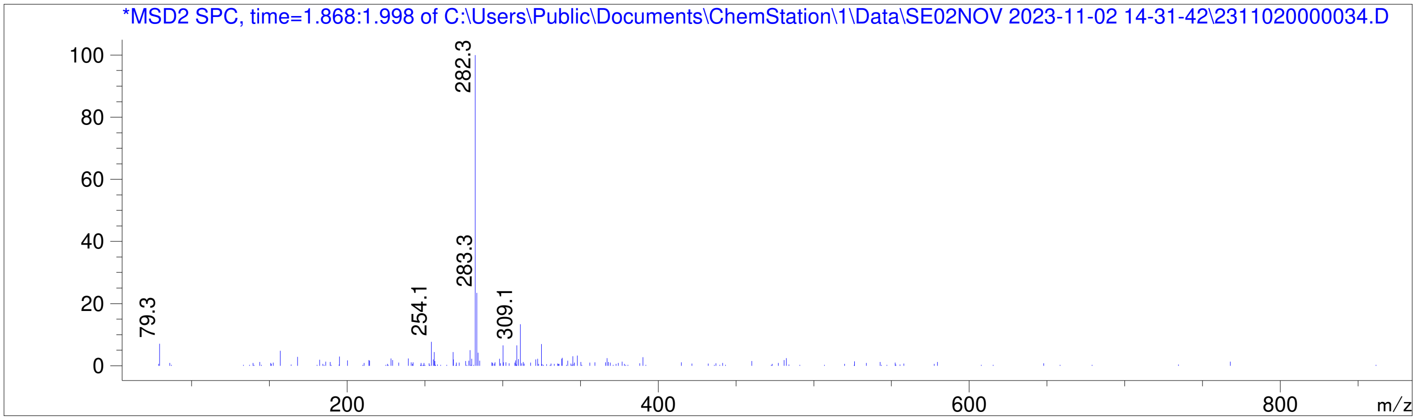
*MSD2 SPC, time=1.868:1.998 of C:\Users\Public\Documents\ChemStation\1\Data\SE02NOV 2023-11-02 14-31-42\2311020000034.D
282.3
283.3
254.1
309.1
79.3
m/z

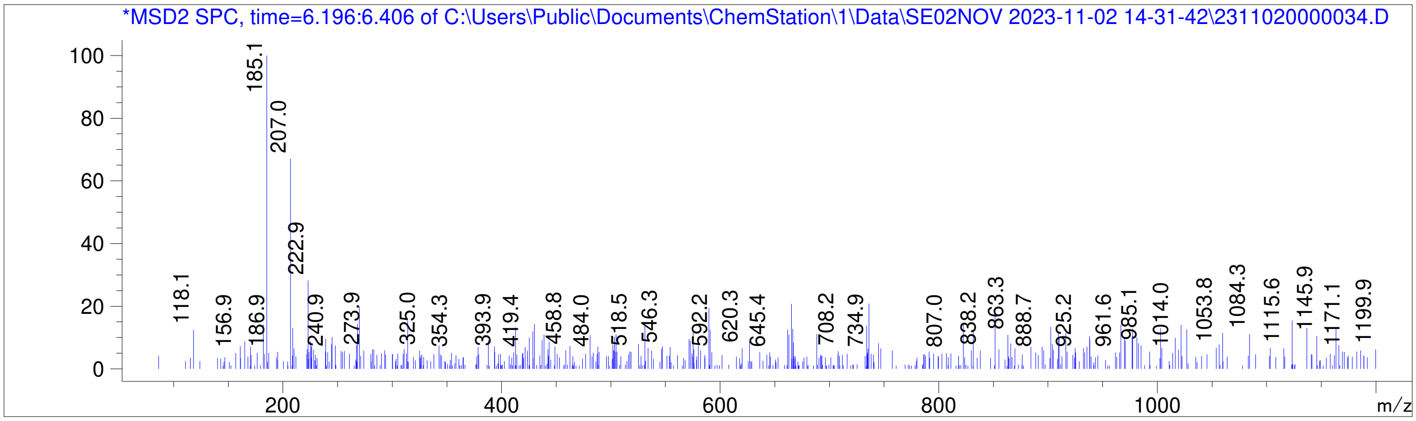
*MSD2 SPC, time=6.196:6.406 of C:\Users\Public\Documents\ChemStation\1\Data\SE02NOV 2023-11-02 14-31-42\2311020000034.D
185.1
207.0
222.9
118.1
156.9
186.9
240.9
273.9
325.0
354.3
393.9
419.4
458.8
484.0
518.5
546.3
592.2
620.3
645.4
708.2
734.9
807.0
838.2
863.3
888.7
925.2
961.6
985.1
1014.0
1053.8
1084.3
1115.6
1145.9
1171.1
1199.9
m/z

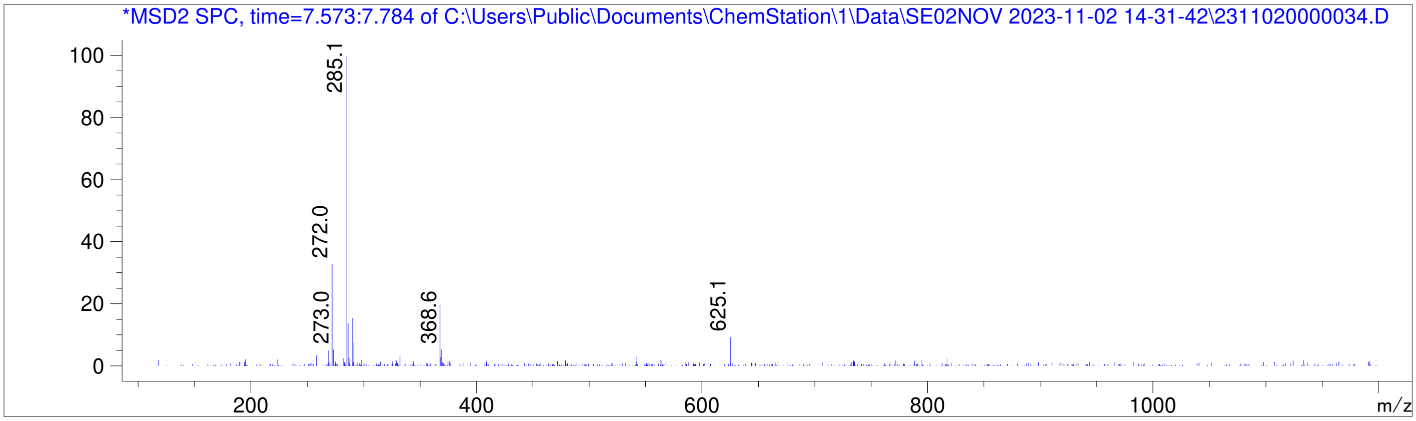
*MSD2 SPC, time=7.573:7.784 of C:\Users\Public\Documents\ChemStation\1\Data\SE02NOV 2023-11-02 14-31-42\2311020000034.D
285.1
272.0
273.0
368.6
625.1
m/z

*MSD2 SPC, time=8.546:8.805 of C:\Users\Public\Documents\ChemStation\1\Data\SE02NOV 2023-11-02 14-31-42\231102000034.D

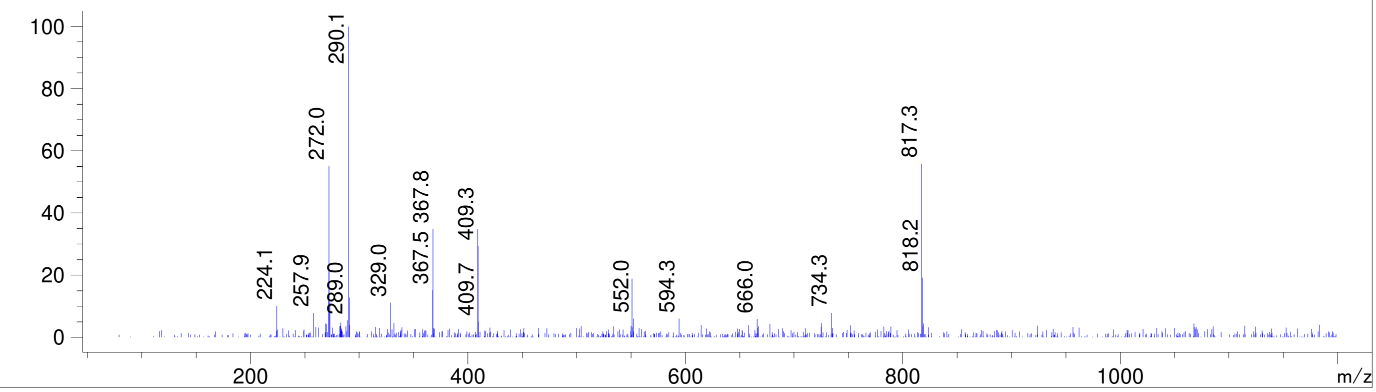

*MSD2 SPC, time=8.805:9.000 of C:\Users\Public\Documents\ChemStation\1\Data\SE02NOV 2023-11-02 14-31-42\231102000034.D

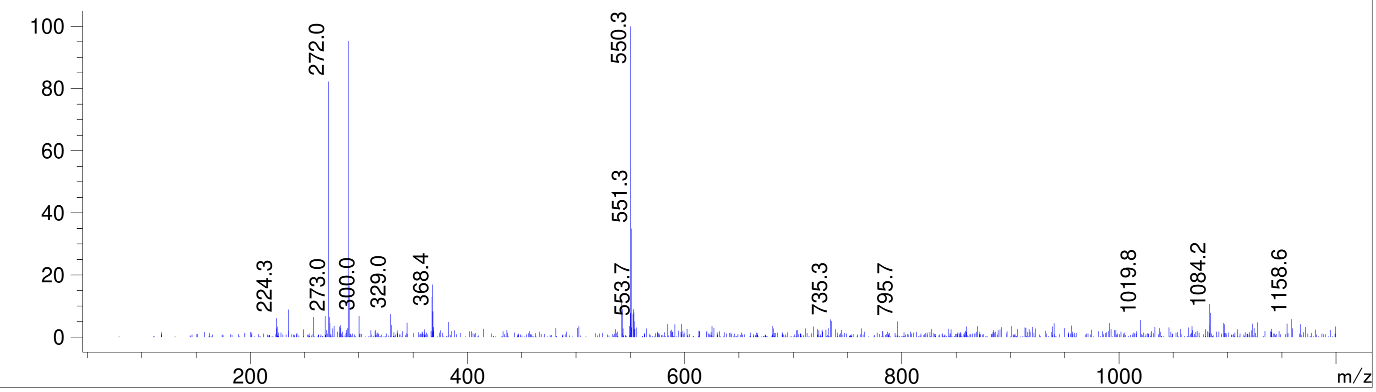

*MSD2 SPC, time=9.000:9.162 of C:\Users\Public\Documents\ChemStation\1\Data\SE02NOV 2023-11-02 14-31-42\231102000034.D

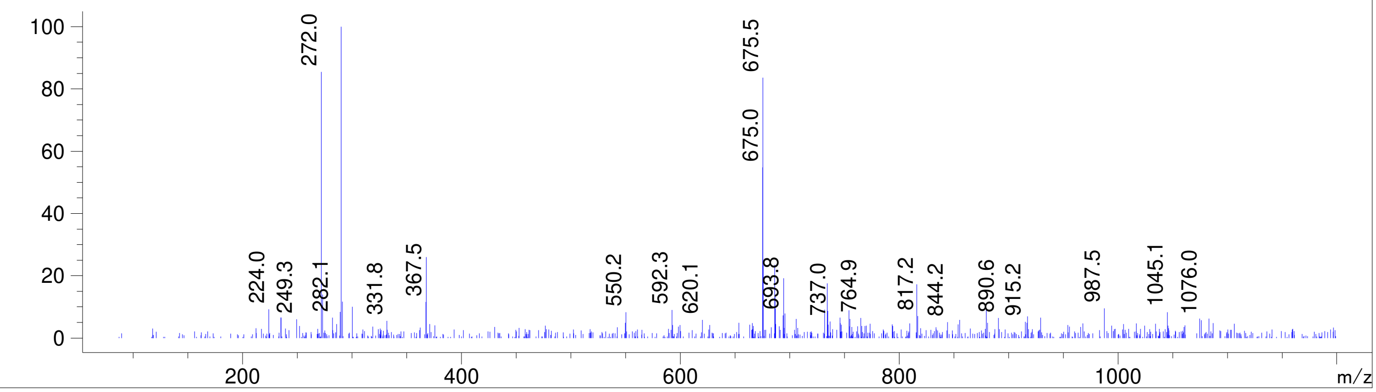

*MSD2 SPC, time=9.162:9.308 of C:\Users\Public\Documents\ChemStation\1\Data\SE02NOV 2023-11-02 14-31-42\231102000034.D

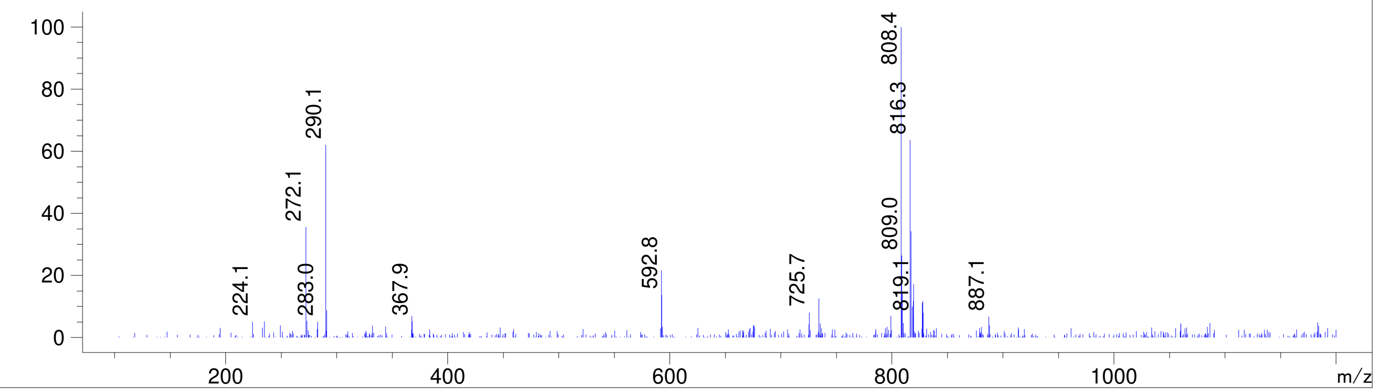

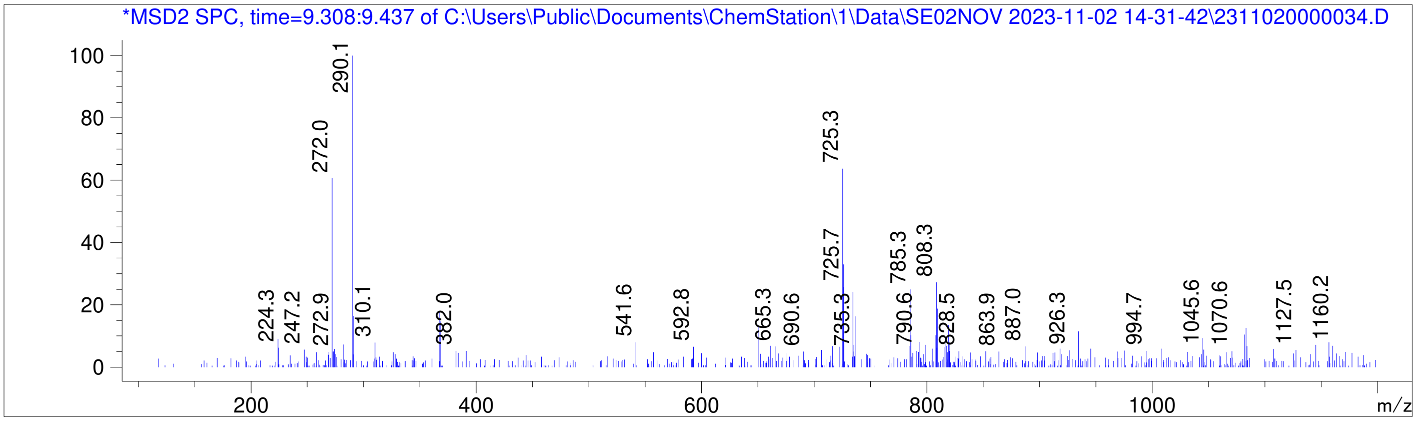
*MSD2 SPC, time=9.308:9.437 of C:\Users\Public\Documents\ChemStation\1\Data\SE02NOV 2023-11-02 14-31-42\231102000034.D
100
80
60
40
20
0
290.1
272.0
725.3
725.7
785.3
808.3
224.3
247.2
272.9
310.1
382.0
541.6
592.8
665.3
690.6
735.3
790.6
828.5
863.9
887.0
926.3
994.7
1045.6
1070.6
1127.5
1160.2
200
400
600
800
1000
m/z

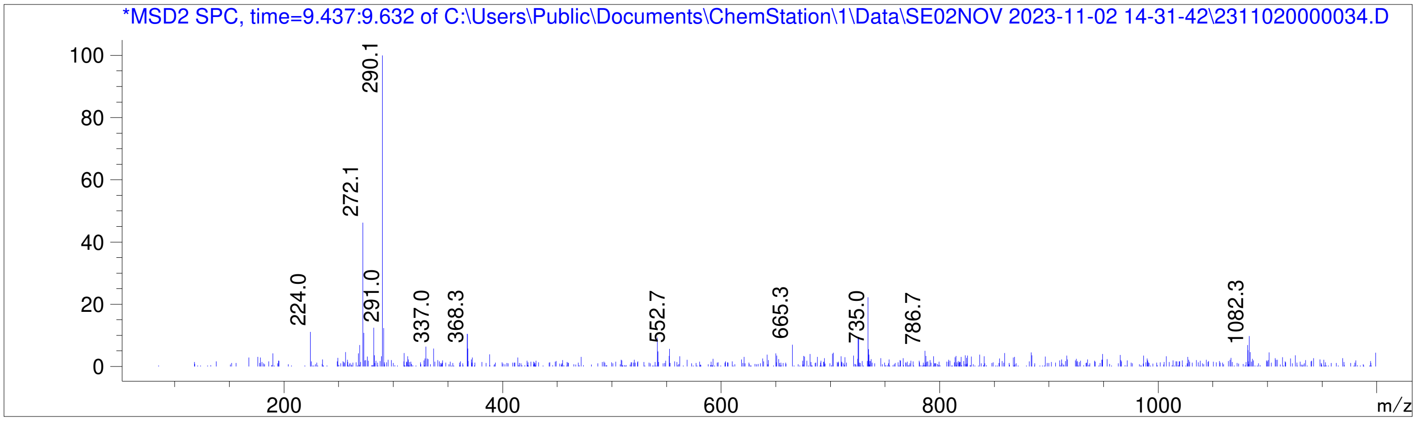
*MSD2 SPC, time=9.437:9.632 of C:\Users\Public\Documents\ChemStation\1\Data\SE02NOV 2023-11-02 14-31-42\231102000034.D
100
80
60
40
20
0
290.1
272.1
224.0
291.0
337.0
368.3
552.7
665.3
735.0
786.7
1082.3
200
400
600
800
1000
m/z

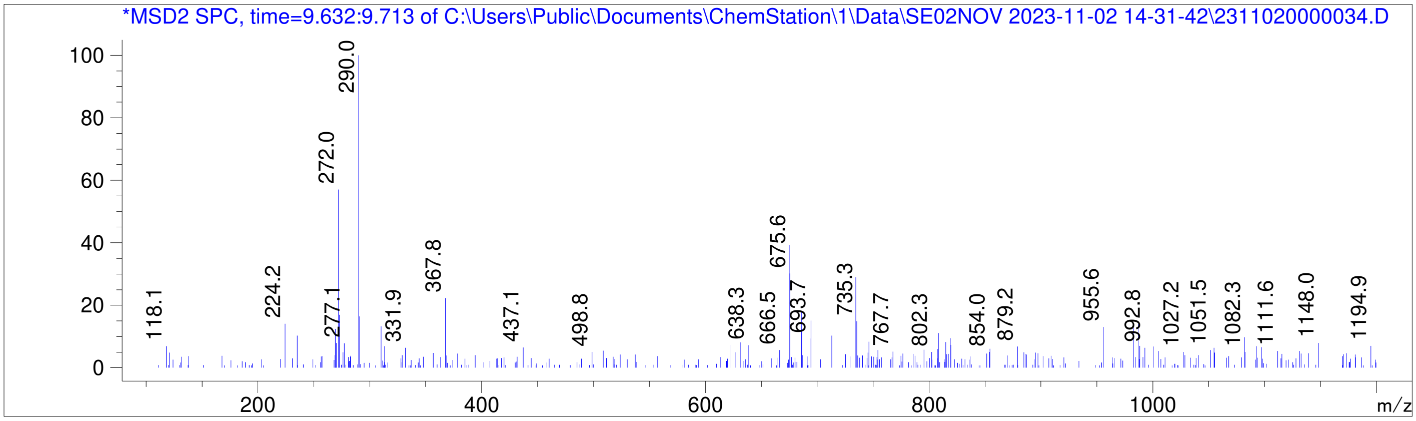
*MSD2 SPC, time=9.632:9.713 of C:\Users\Public\Documents\ChemStation\1\Data\SE02NOV 2023-11-02 14-31-42\231102000034.D
100
80
60
40
20
0
290.0
272.0
675.6
118.1
224.2
277.1
331.9
367.8
437.1
498.8
638.3
666.5
693.7
735.3
767.7
802.3
854.0
879.2
955.6
992.8
1027.2
1051.5
1082.3
1111.6
1148.0
1194.9
200
400
600
800
1000
m/z

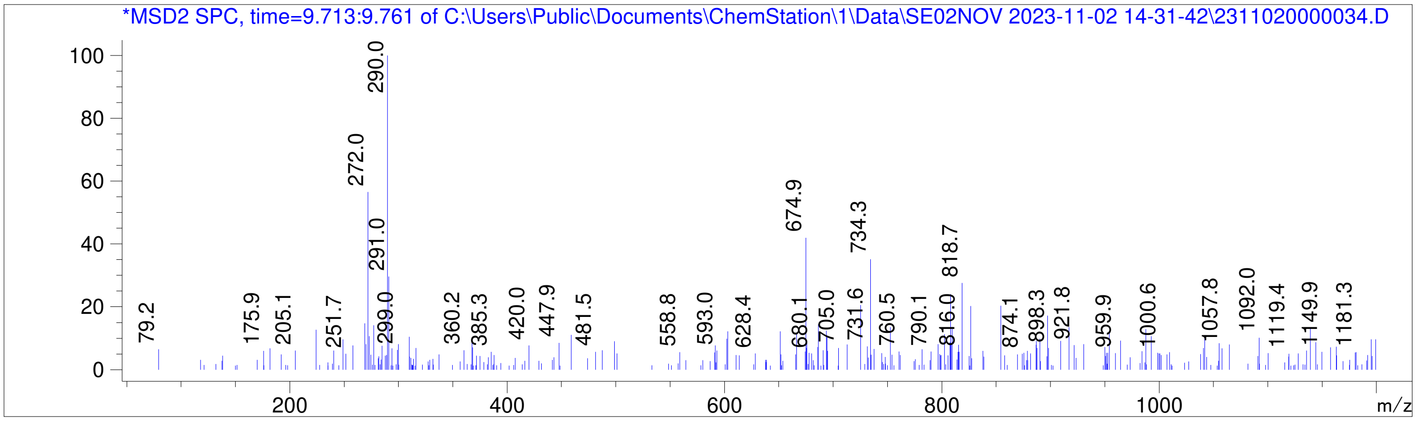

*MSD2 SPC, time=9.713:9.761 of C:\Users\Public\Documents\ChemStation\1\Data\SE02NOV 2023-11-02 14-31-42\2311020000034.D
290.0
272.0
291.0
674.9
734.3
818.7
79.2
175.9
205.1
251.7
299.0
360.2
385.3
420.0
447.9
481.5
558.8
593.0
628.4
680.1
705.0
731.6
760.5
790.1
816.0
874.1
898.3
921.8
959.9
1000.6
1057.8
1092.0
1119.4
1149.9
1181.3
m/z

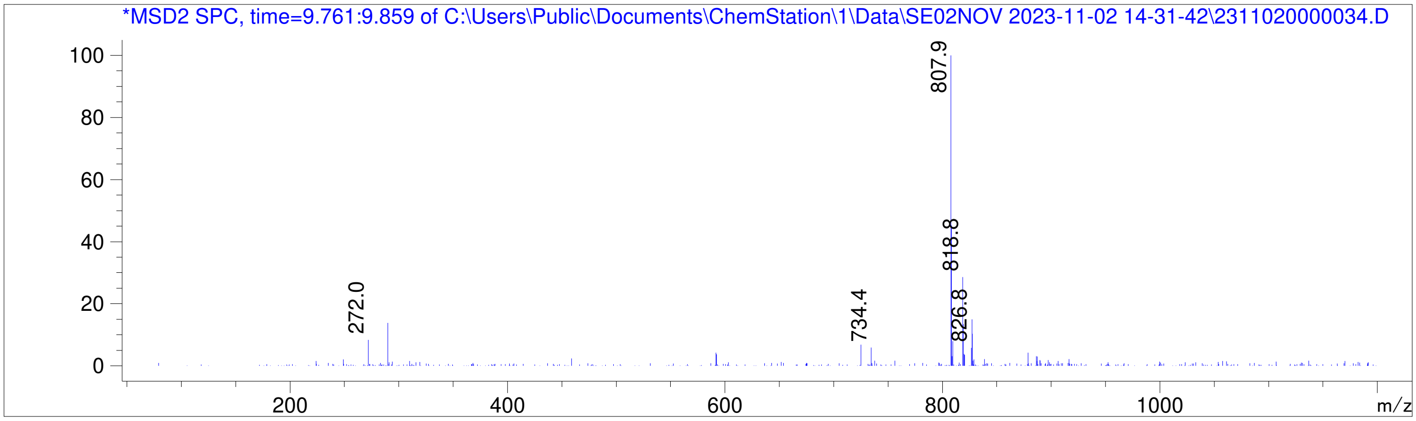

*MSD2 SPC, time=9.761:9.859 of C:\Users\Public\Documents\ChemStation\1\Data\SE02NOV 2023-11-02 14-31-42\2311020000034.D
807.9
818.8
272.0
734.4
826.8
m/z

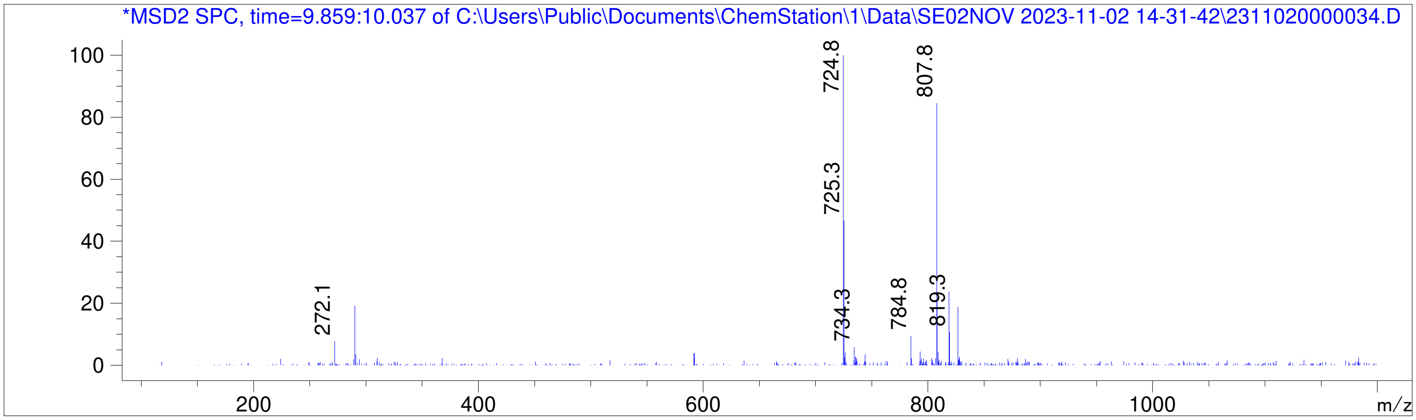

*MSD2 SPC, time=9.859:10.037 of C:\Users\Public\Documents\ChemStation\1\Data\SE02NOV 2023-11-02 14-31-42\2311020000034.D
724.8
807.8
725.3
272.1
734.3
784.8
819.3
m/z

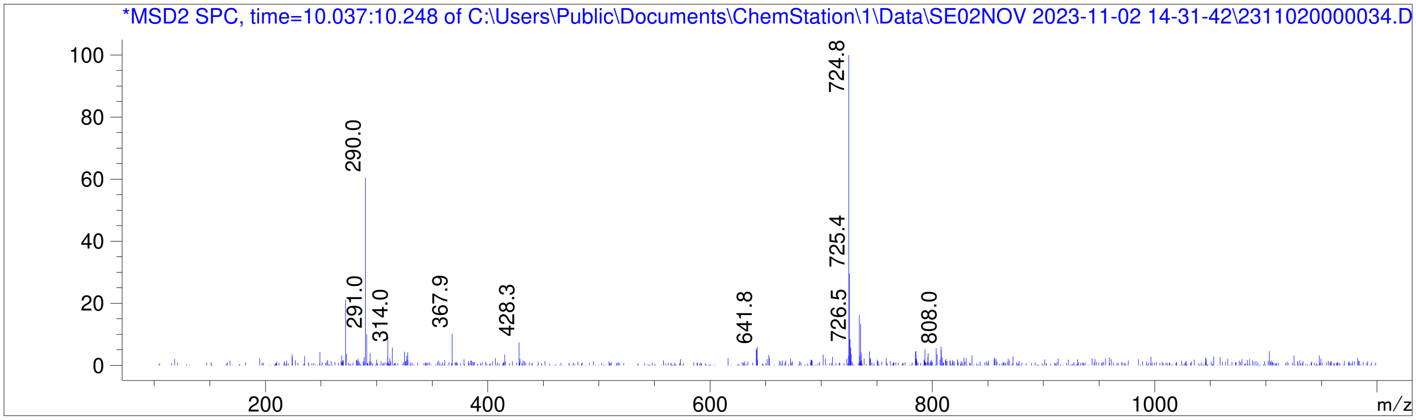

*MSD2 SPC, time=10.037:10.248 of C:\Users\Public\Documents\ChemStation\1\Data\SE02NOV 2023-11-02 14-31-42\2311020000034.D
724.8
290.0
725.4
291.0
314.0
367.9
428.3
641.8
726.5
808.0
m/z

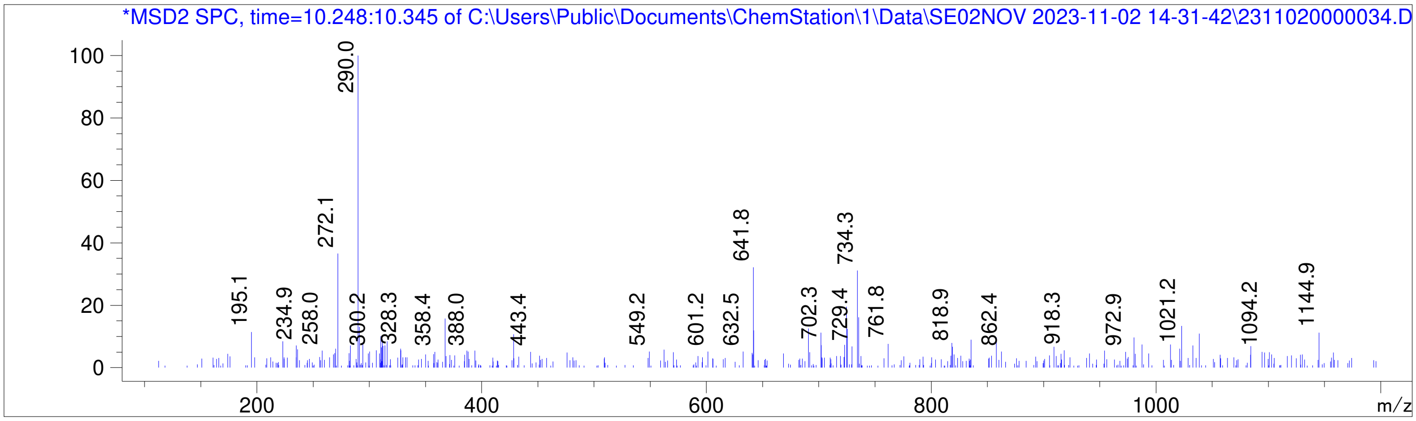
*MSD2 SPC, time=10.248:10.345 of C:\Users\Public\Documents\ChemStation\1\Data\SE02NOV 2023-11-02 14-31-42\2311020000034.D
100
80
60
40
20
0
290.0
272.1
195.1
234.9
258.0
300.2
328.3
358.4
388.0
443.4
549.2
601.2
632.5
641.8
702.3
729.4
734.3
761.8
818.9
862.4
918.3
972.9
1021.2
1094.2
1144.9
200
400
600
800
1000
m/z
