# Supplementary material for: Astrobiological implications of the stability and reactivity of peptide nucleic acid (PNA) in concentrated sulfuric acid
Source: Sci Adv. 2025 Mar 26;11(13):eadr0006. doi: 10.1126/sciadv.adr0006 (PMC11939054; doi:10.1126/sciadv.adr0006)

Data -> C:\USERS\PUBLIC\DOCUMENTS\CHEMSTATION\1\DATA\SE07NOV 2023-11-07 08-21-00\ ->
Sample-> CPT22010446-13-D2-80deg-1h

===

Injection Date : Tue, 7. Nov. 2023

Seq Line : 11
Location : 51
Inj. Vol. : 2 µl

Acq. Method : C:\Users\Public\Documents\ChemStation\1\Data\SE07NOV 2023-11-07 08-21-00\22010446 LCMS-6.M
Analysis Method : C:\Users\Public\Documents\ChemStation\1\Data\SE07NOV 2023-11-07 08-21-00\22010446 LCMS-6.M (Sequence Method)
Waters XBridge Phenyl (4.6 * 150 mm; 3.5 µm); 0.05% TFA (aq) / AcN: 100/0 (0.0 min) --> (6.0 min) --> 70/30 (0.0 min) --> (2.0 min) --> 10/90 (2.0 min); Flow: 1.0 ml/min;
MSD1 = positive; MSD2 = negative

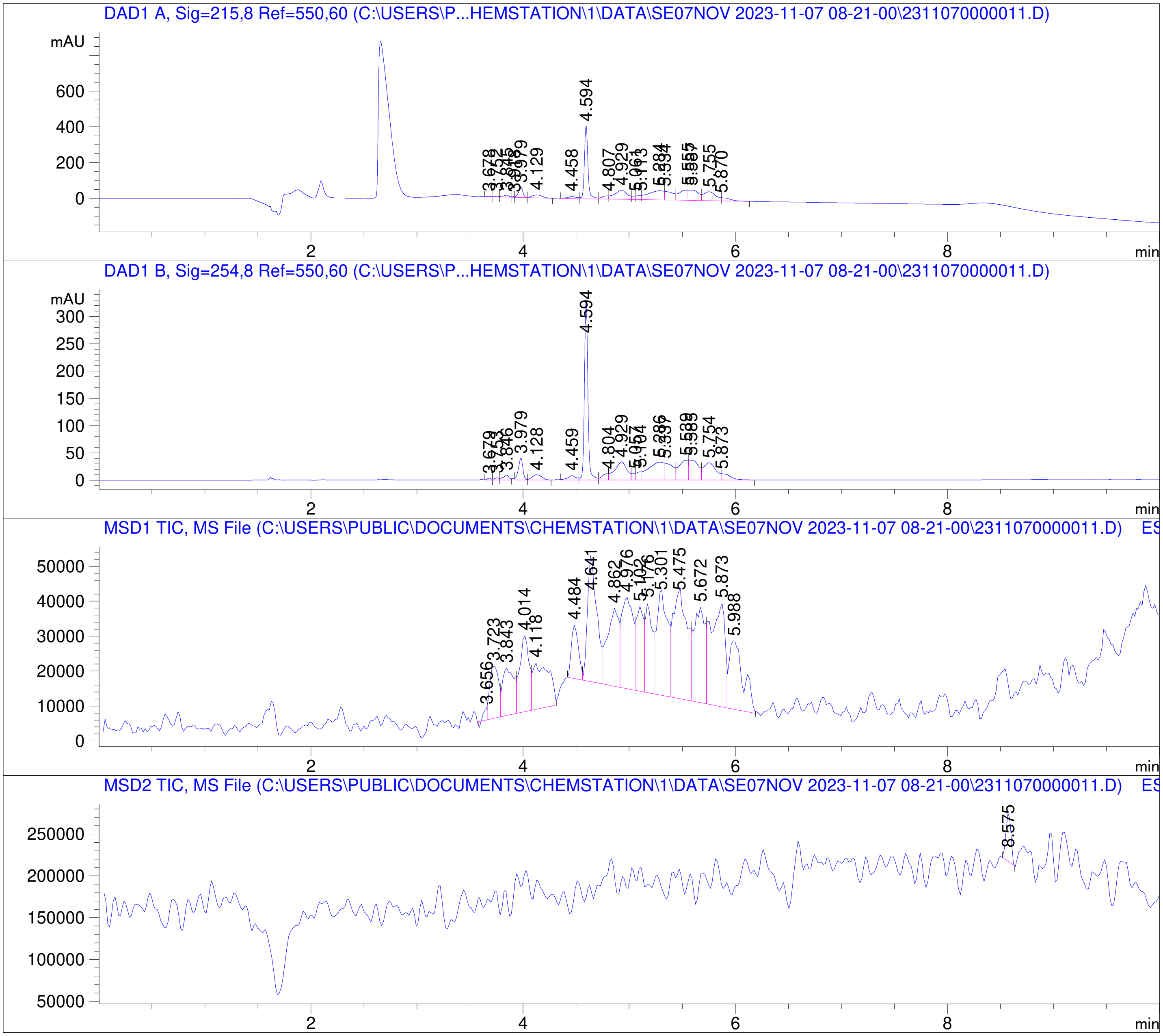

DAD1 A, Sig=215,8 Ref=550,60

Peak #	Ret. Time [min]	Area [mV *s]	Area %
1	3.678	8.470	0.213
2	3.752	14.493	0.365
3	3.845	45.932	1.155
4	3.918	5.767	0.145
5	3.979	164.026	4.126
6	4.129	102.877	2.588
7	4.458	65.093	1.637
8	4.594	936.371	23.555
9	4.807	76.167	1.916
10	4.929	415.599	10.455
11	5.061	47.241	1.188
12	5.113	68.385	1.720
13	5.284	532.885	13.405
14	5.334	269.864	6.789
15	5.555	333.511	8.390
16	5.587	375.601	9.449
17	5.755	411.621	10.355
18	5.870	101.317	2.549

DAD1 B, Sig=254,8 Ref=550,60

Peak #	Ret. Time [min]	Area [mV *s]	Area %
1	3.679	8.408	0.312
2	3.753	11.246	0.417
3	3.846	34.979	1.297
4	3.979	117.722	4.366
5	4.128	59.528	2.208
6	4.459	41.724	1.547
7	4.594	751.417	27.866
8	4.804	45.911	1.703
9	4.929	268.479	9.957
10	5.057	27.563	1.022
11	5.104	44.460	1.649
12	5.286	345.798	12.824
13	5.337	166.446	6.173
14	5.539	213.831	7.930
15	5.585	235.463	8.732
16	5.754	258.650	9.592
17	5.873	64.871	2.406

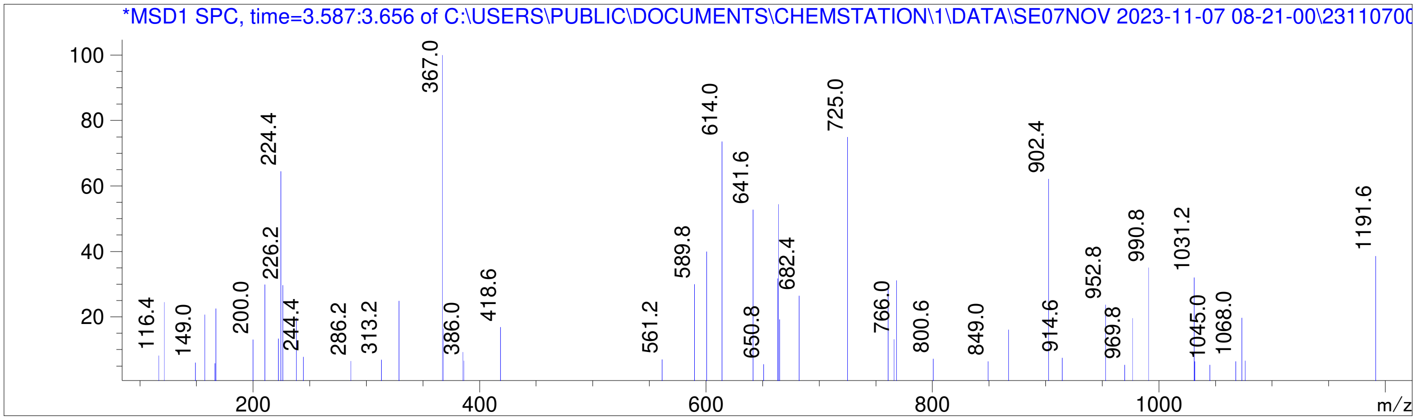
*MSD1 SPC, time=3.587:3.656 of C:\USERS\PUBLIC\DOCUMENTS\CHEMSTATION\1\DATA\SE07NOV 2023-11-07 08-21-00\23110700
116.4
149.0
200.0
224.4
226.2
244.4
286.2
313.2
367.0
386.0
418.6
561.2
589.8
614.0
641.6
650.8
682.4
725.0
766.0
800.6
849.0
902.4
914.6
952.8
969.8
990.8
1031.2
1045.0
1068.0
1191.6
m/z

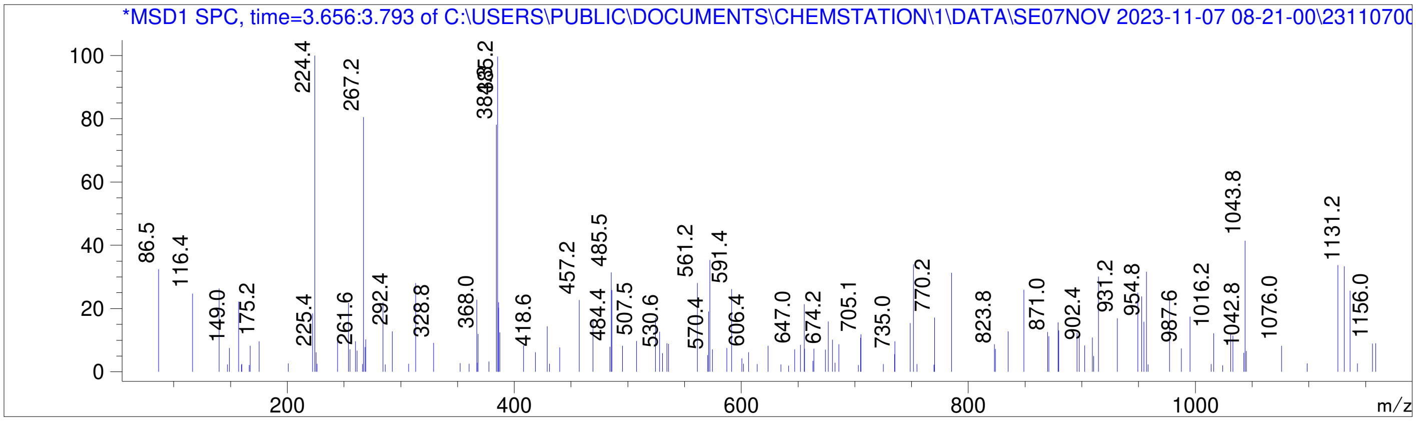
*MSD1 SPC, time=3.656:3.793 of C:\USERS\PUBLIC\DOCUMENTS\CHEMSTATION\1\DATA\SE07NOV 2023-11-07 08-21-00\23110700
86.5
116.4
149.0
175.2
224.4
225.4
261.6
267.2
292.4
328.8
368.0
418.6
457.2
484.4
485.5
507.5
530.6
561.2
570.4
591.4
606.4
647.0
674.2
705.1
735.0
770.2
823.8
871.0
902.4
931.2
954.8
987.6
1016.2
1042.8
1043.8
1076.0
1131.2
1156.0
m/z

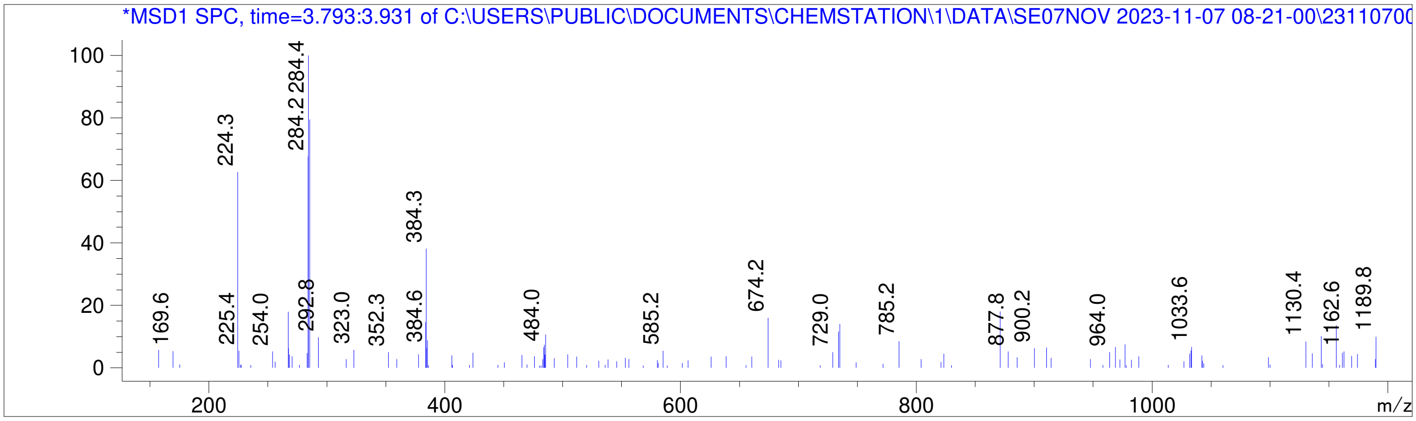
*MSD1 SPC, time=3.793:3.931 of C:\USERS\PUBLIC\DOCUMENTS\CHEMSTATION\1\DATA\SE07NOV 2023-11-07 08-21-00\23110700
169.6
224.3
225.4
254.0
284.2
284.4
292.8
323.0
352.3
384.3
384.6
484.0
585.2
674.2
729.0
785.2
877.8
900.2
964.0
1033.6
1130.4
1162.6
1189.8
m/z

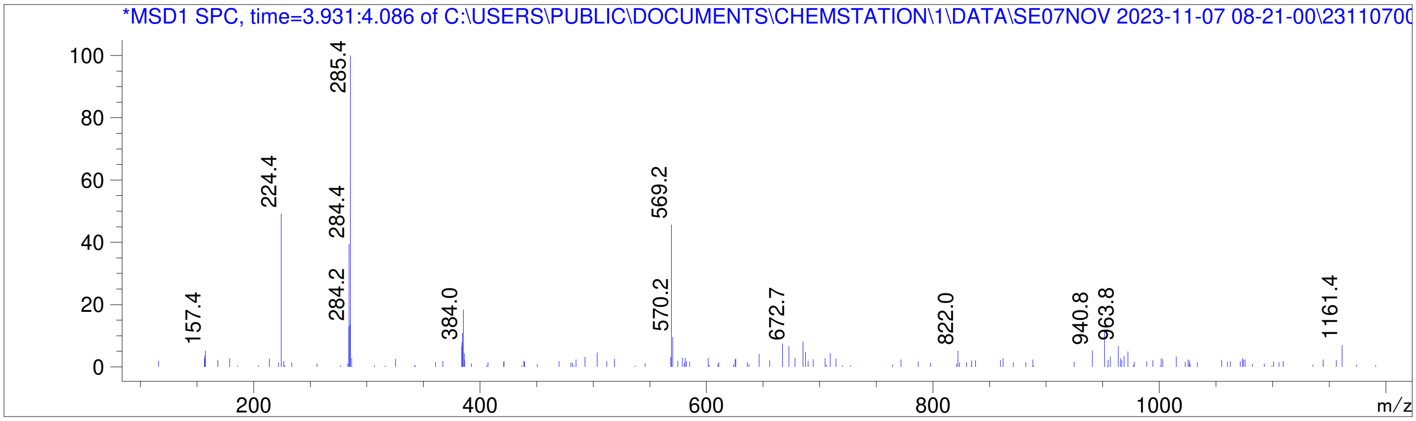
*MSD1 SPC, time=3.931:4.086 of C:\USERS\PUBLIC\DOCUMENTS\CHEMSTATION\1\DATA\SE07NOV 2023-11-07 08-21-00\2311070
285.4
224.4
284.4
569.2
284.2
570.2
384.0
157.4
672.7
822.0
940.8
963.8
1161.4
m/z

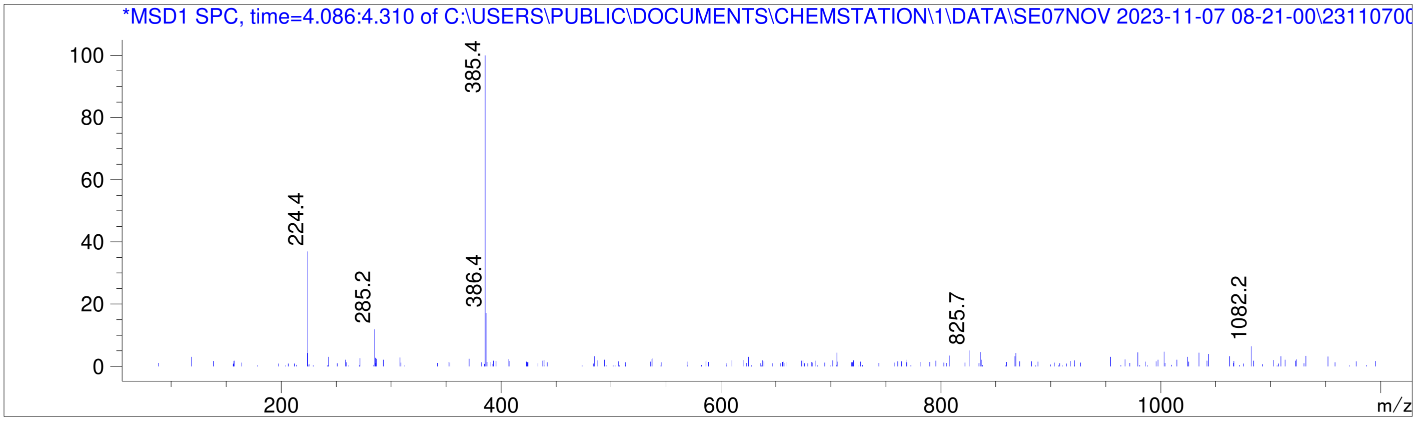
*MSD1 SPC, time=4.086:4.310 of C:\USERS\PUBLIC\DOCUMENTS\CHEMSTATION\1\DATA\SE07NOV 2023-11-07 08-21-00\2311070
385.4
224.4
386.4
285.2
825.7
1082.2
m/z

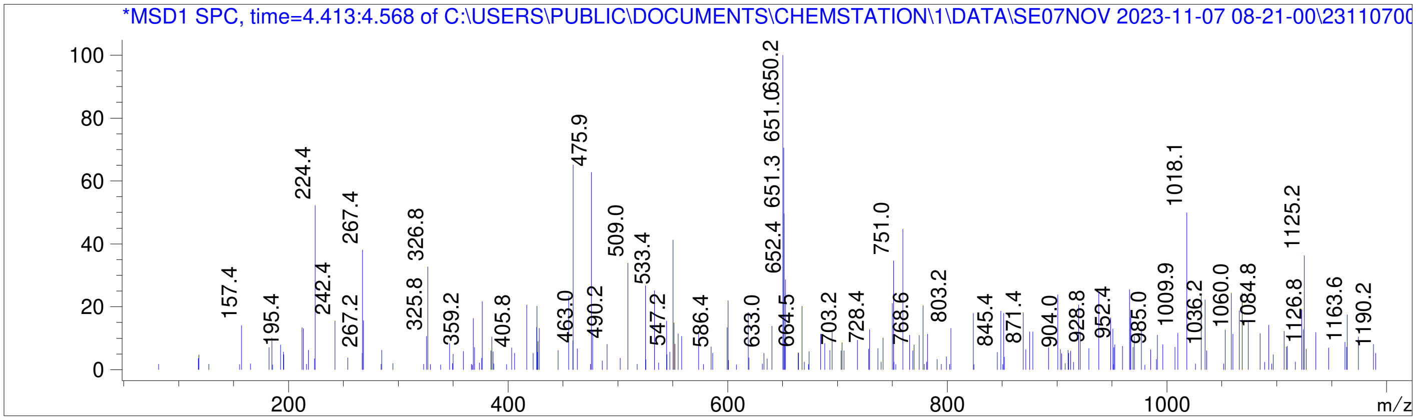
*MSD1 SPC, time=4.413:4.568 of C:\USERS\PUBLIC\DOCUMENTS\CHEMSTATION\1\DATA\SE07NOV 2023-11-07 08-21-00\2311070
650.2
651.0
475.9
651.3
1018.1
224.4
267.4
1125.2
326.8
751.0
509.0
533.4
652.4
m/z

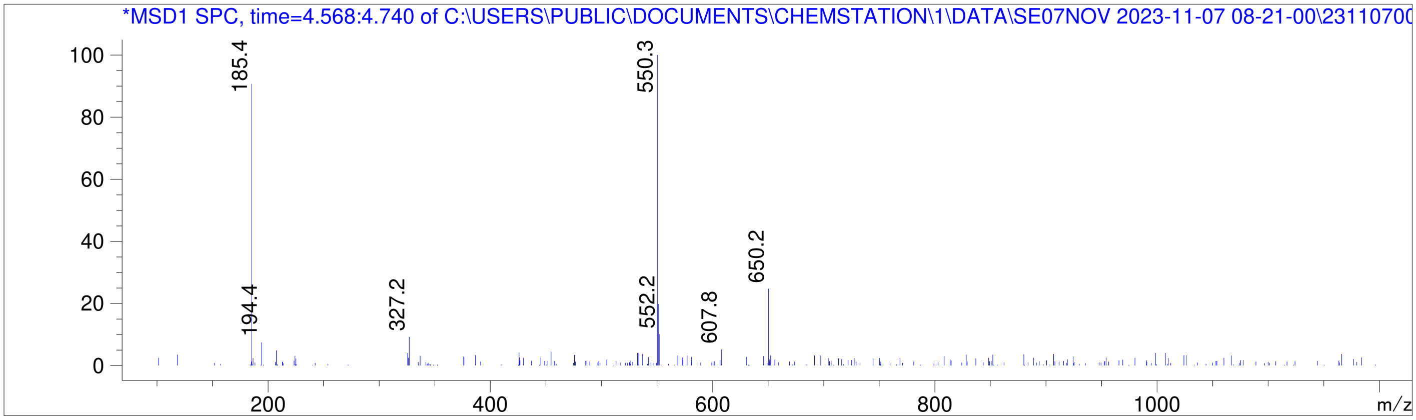
*MSD1 SPC, time=4.568:4.740 of C:\USERS\PUBLIC\DOCUMENTS\CHEMSTATION\1\DATA\SE07NOV 2023-11-07 08-21-00\2311070
185.4
550.3
650.2
327.2
552.2
194.4
607.8
m/z

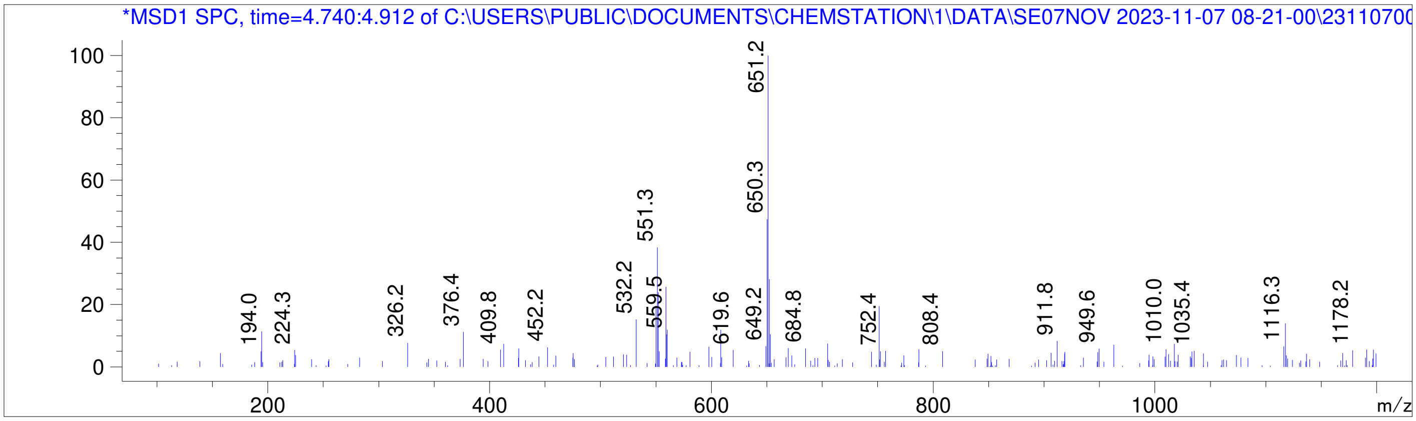
*MSD1 SPC, time=4.740:4.912 of C:\USERS\PUBLIC\DOCUMENTS\CHEMSTATION\1\DATA\SE07NOV 2023-11-07 08-21-00\2311070
651.2
650.3
551.3
532.2
559.5
194.0
224.3
326.2
376.4
409.8
452.2
619.6
649.2
684.8
752.4
808.4
911.8
949.6
1010.0
1035.4
1116.3
1178.2
m/z

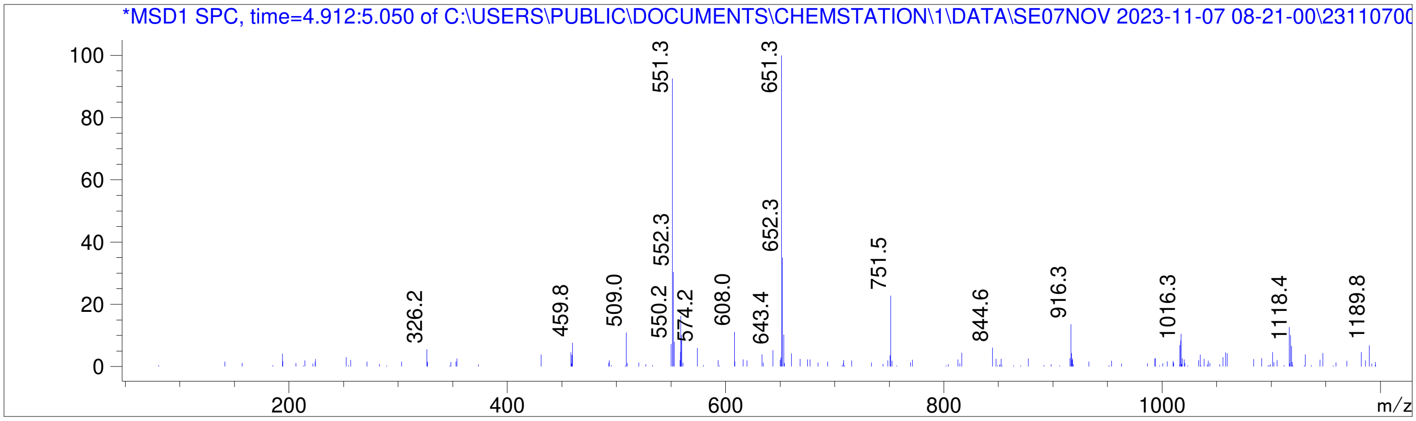
*MSD1 SPC, time=4.912:5.050 of C:\USERS\PUBLIC\DOCUMENTS\CHEMSTATION\1\DATA\SE07NOV 2023-11-07 08-21-00\2311070
551.3
651.3
552.3
652.3
751.5
326.2
459.8
509.0
550.2
574.2
608.0
643.4
844.6
916.3
1016.3
1118.4
1189.8
m/z

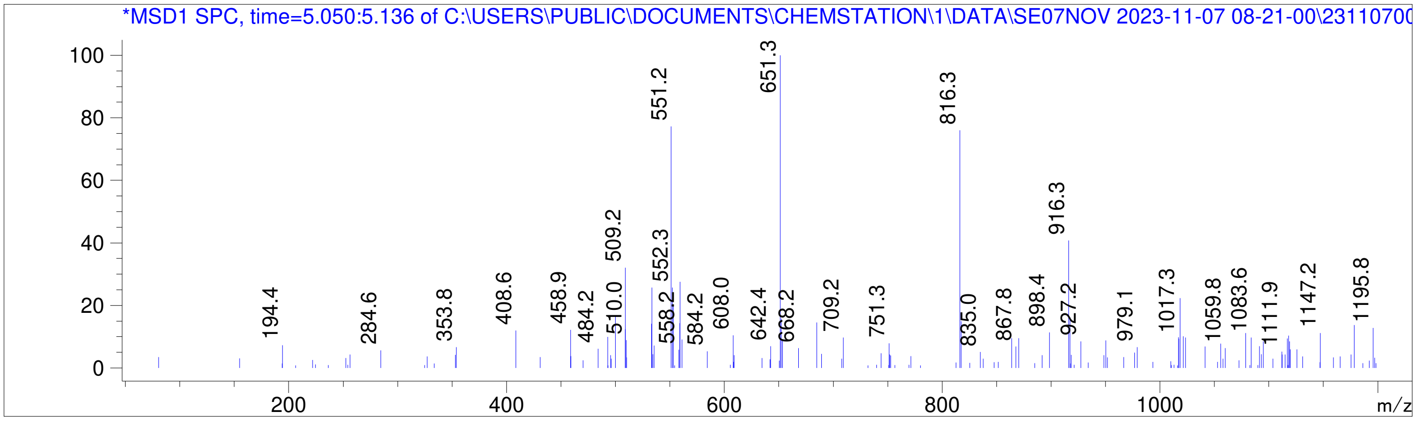
*MSD1 SPC, time=5.050:5.136 of C:\USERS\PUBLIC\DOCUMENTS\CHEMSTATION\1\DATA\SE07NOV 2023-11-07 08-21-00\2311070
651.3
551.2
816.3
916.3
509.2
552.3
194.4
284.6
353.8
408.6
458.9
484.2
510.0
558.2
584.2
608.0
642.4
668.2
709.2
751.3
835.0
867.8
898.4
927.2
979.1
1017.3
1059.8
1083.6
1111.9
1147.2
1195.8
m/z

Sample-> CPT22010446-13-D2-80deg-1h

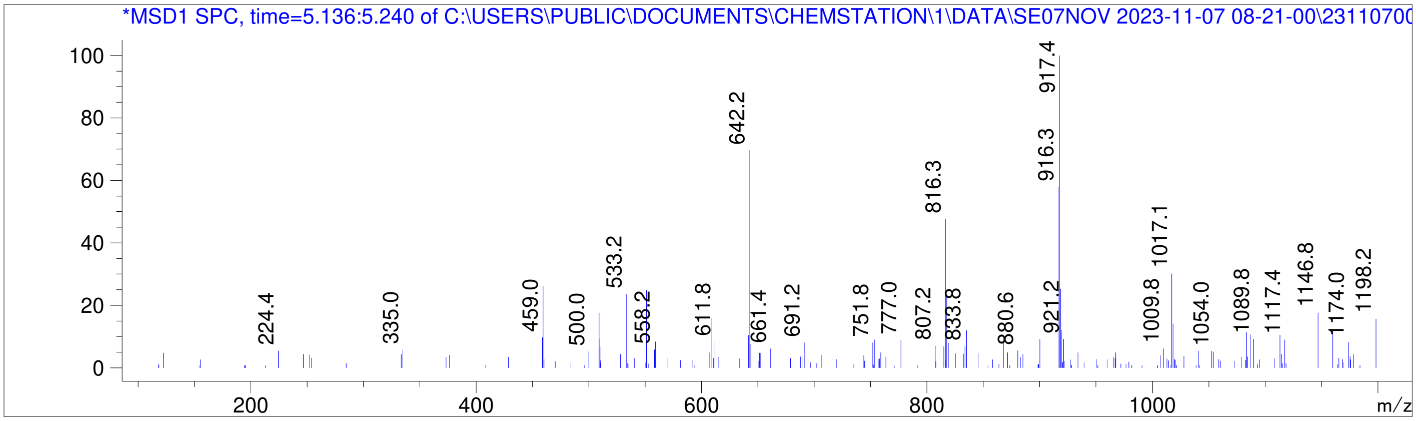

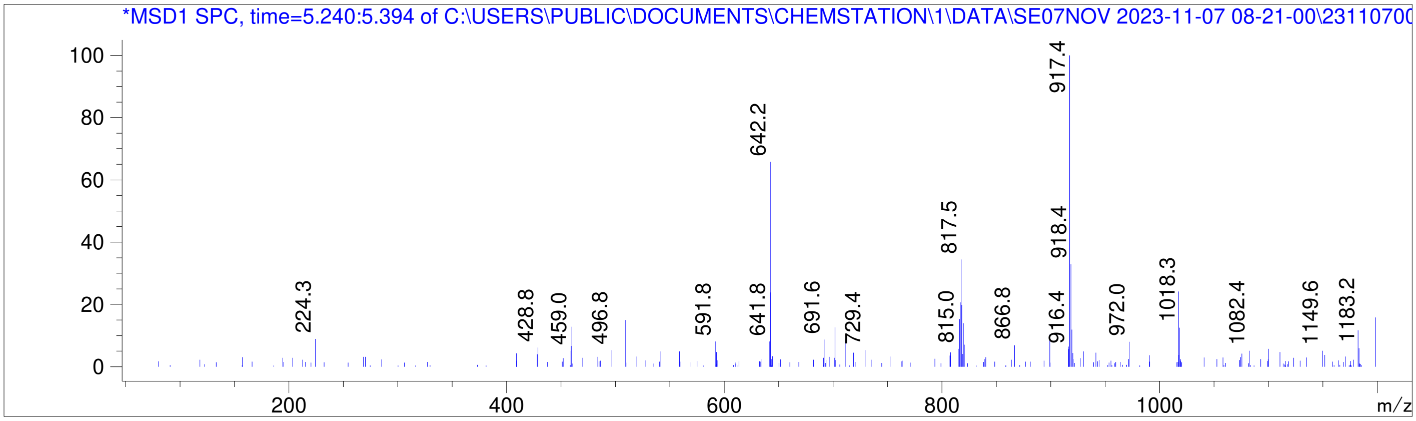

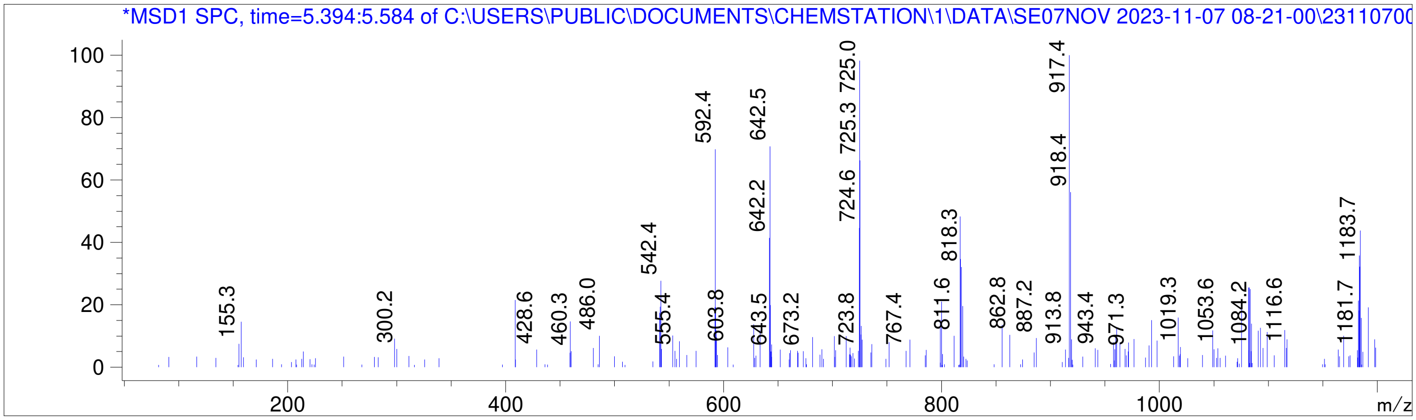

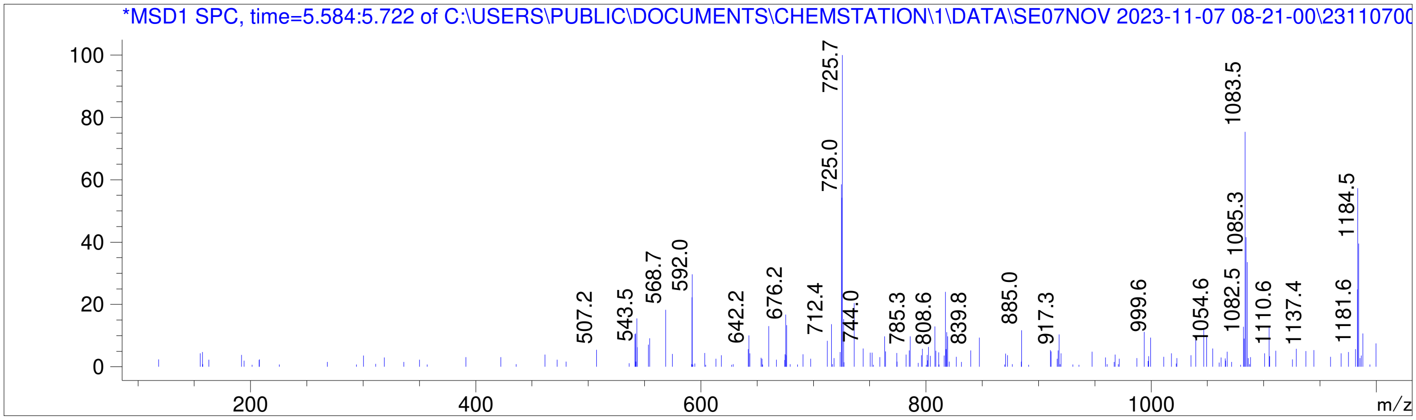

==

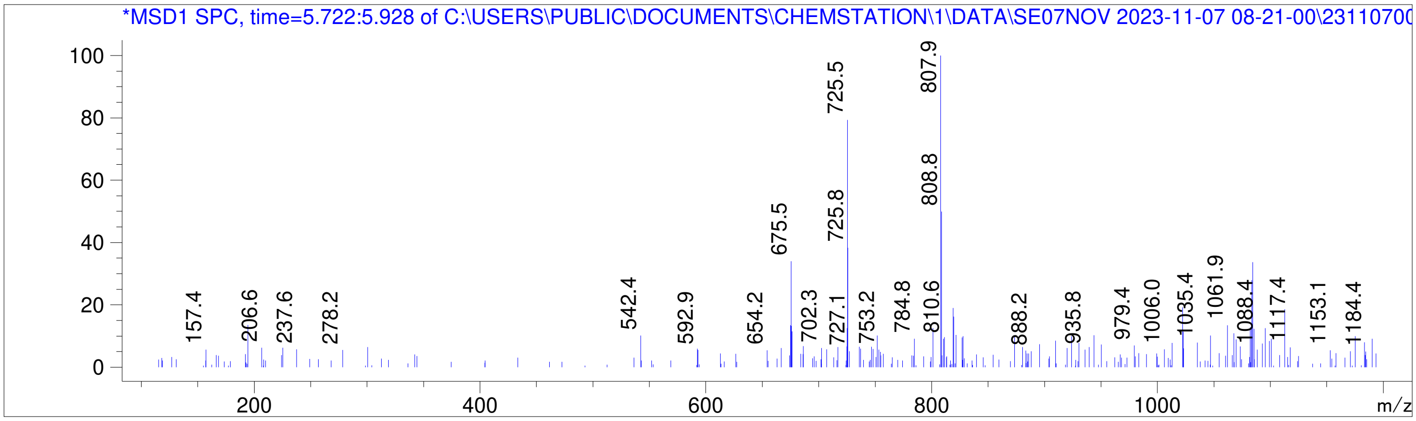
*MSD1 SPC, time=5.722:5.928 of C:\USERS\PUBLIC\DOCUMENTS\CHEMSTATION\1\DATA\SE07NOV 2023-11-07 08-21-00\2311070
100
80
60
40
20
0
157.4
206.6
237.6
278.2
542.4
592.9
654.2
675.5
702.3
727.1
725.8
725.5
753.2
784.8
810.6
808.8
807.9
888.2
935.8
979.4
1006.0
1035.4
1061.9
1088.4
1117.4
1153.1
1184.4
200
400
600
800
1000
m/z

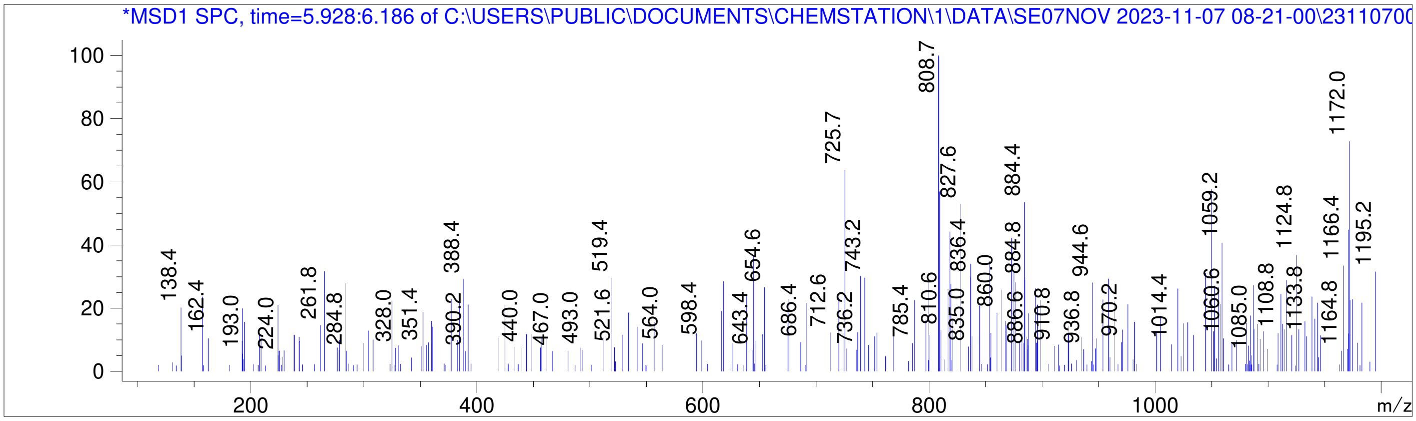
*MSD1 SPC, time=5.928:6.186 of C:\USERS\PUBLIC\DOCUMENTS\CHEMSTATION\1\DATA\SE07NOV 2023-11-07 08-21-00\2311070
100
80
60
40
20
0
138.4
162.4
193.0
224.0
261.8
284.8
328.0
351.4
388.4
390.2
440.0
467.0
493.0
519.4
521.6
564.0
598.4
643.4
654.6
686.4
712.6
725.7
736.2
743.2
785.4
810.6
808.7
827.6
835.0
836.4
860.0
886.6
884.8
884.4
910.8
936.8
944.6
970.2
1014.4
1060.6
1059.2
1085.0
1108.8
1124.8
1133.8
1164.8
1166.4
1172.0
1195.2
200
400
600
800
1000
m/z

Sample-> CPT22010446-13-D2-80deg-1h

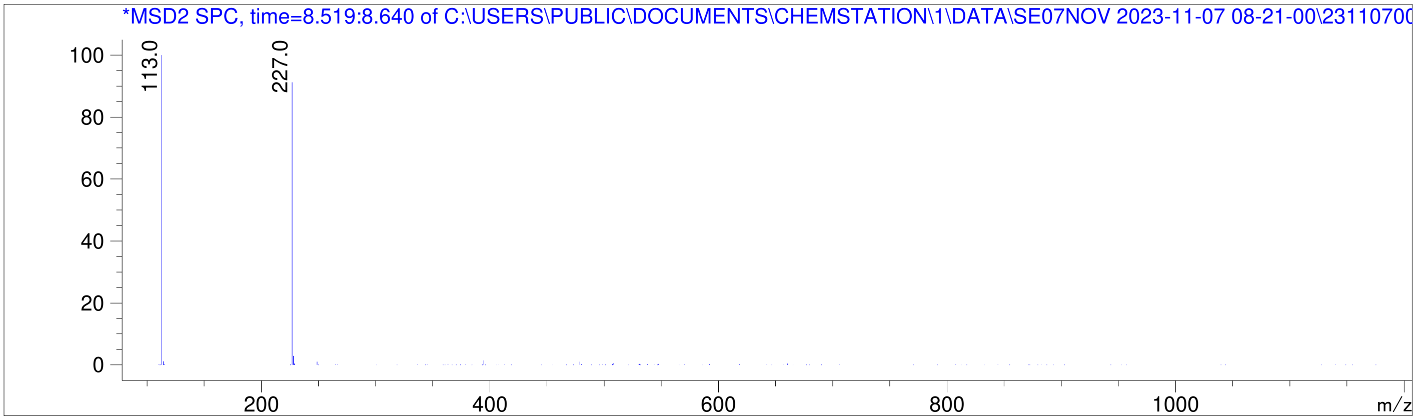

Supplement: Supplementary file 2 — Data S1 and S2 [file sciadv.adr0006_data_s1_and_s2.zip › Supplementary Dataset 1-LCMS DATA/LCMS PNA Hexamers A-T/LCMS T6 50C_80C/80C/1h/CPT22010446-13-D2-80deg-1h.pdf]
