# Supplementary material for: Astrobiological implications of the stability and reactivity of peptide nucleic acid (PNA) in concentrated sulfuric acid
Source: Sci Adv. 2025 Mar 26;11(13):eadr0006. doi: 10.1126/sciadv.adr0006 (PMC11939054; doi:10.1126/sciadv.adr0006)
Supplement: Supplementary file 2 — Data S1 and S2 [file sciadv.adr0006_data_s1_and_s2.zip › Supplementary Dataset 1-LCMS DATA/LCMS PNA Hexamers A-T/LCMS T6 50C_80C/80C/1h/CPT22010446-19-D1-80dg-1h.pdf]

===

Injection Date : Thu, 19. Oct. 2023	Seq Line	:	4
	Location	:	64
	Inj. Vol.	:	8 µl

Acq. Method : C:\Users\Public\Documents\ChemStation\1\Data\SE19OCT 2023-10-19 14-58-54\22010446C LCMS-6#.M

Analysis Method : C:\Users\Public\Documents\ChemStation\1\Data\SE19OCT 2023-10-19 14-58-54\22010446C LCMS-6#.M (Sequence Method)

Waters XBridge BEH Amide (4.6 x 150 mm, 2.5 µm); PN# 186006726

Mobile Phase A: 20mM Ammonium Acetate (aq) pH 8.2

Mobile Phase B: AcN

Mobile Phase A / Mobile Phase B: 5/95 (0 min) --> (10 min) --> 60/40 (5 min); Flow: 1.0 ml/min; MSD1 = positive; MSD2 = negative

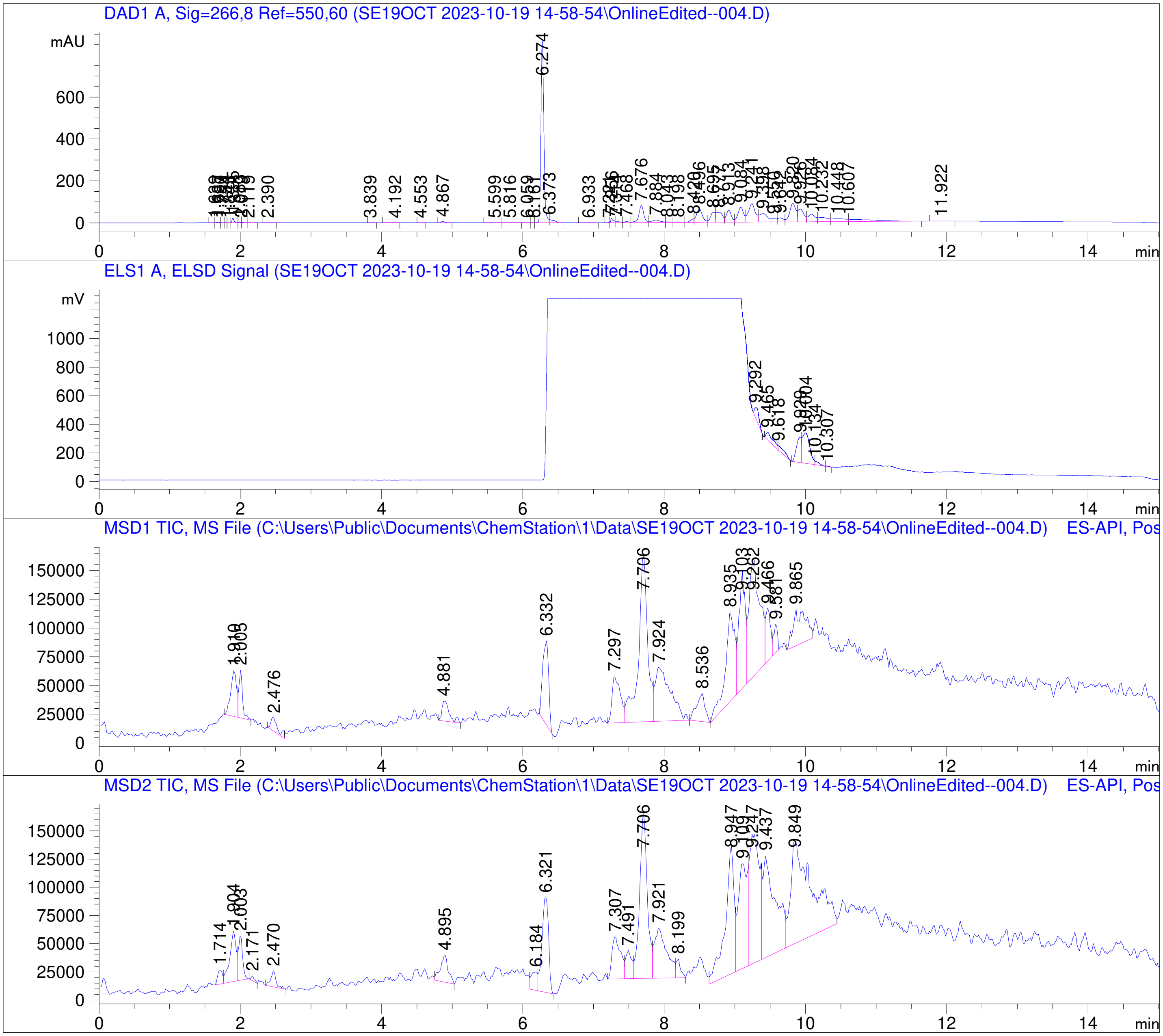

DAD1 A, Sig=266,8 Ref=550,60

Peak #	Ret. Time [min]	Area [mV *s]	Area %
1	1.639	0.758	0.010
2	1.687	7.775	0.099
3	1.720	1.462	0.019
4	1.794	0.696	0.009
5	1.843	0.901	0.011
6	1.895	42.539	0.540
7	1.983	5.826	0.074
8	2.019	4.308	0.055
9	2.119	2.158	0.027
10	2.390	2.881	0.037
11	3.839	0.162	0.002
12	4.192	0.364	0.005
13	4.553	0.442	0.006
14	4.867	21.179	0.269
15	5.599	2.927	0.037
16	5.816	3.338	0.042
17	6.059	0.821	0.010
18	6.161	1.045	0.013
19	6.274	2477.123	31.451
20	6.373	72.417	0.919
21	6.933	6.203	0.079
22	7.221	3.063	0.039
23	7.256	54.707	0.695
24	7.314	25.201	0.320
25	7.468	23.069	0.293
26	7.676	390.825	4.962
27	7.884	96.950	1.231
28	8.043	14.062	0.179
29	8.198	17.280	0.219
30	8.420	55.321	0.702
31	8.496	374.386	4.753
32	8.695	221.088	2.807
33	8.777	294.573	3.740
34	8.913	288.061	3.657
35	9.084	405.908	5.154
36	9.241	594.804	7.552
37	9.398	325.734	4.136
38	9.550	80.356	1.020
39	9.642	107.761	1.368
40	9.820	542.360	6.886
41	9.926	364.722	4.631
42	10.084	256.824	3.261
43	10.232	199.598	2.534
44	10.448	190.403	2.417
45	10.607	286.783	3.641
46	11.922	6.924	0.088

ELS1 A, ELSD Signal

Peak #	Ret. Time [min]	Area [mV *s]	Area %
1	9.292	367.157	10.528
2	9.465	397.490	11.398

Sample-> CPT22010446-19-D1-80dg-1h

Peak #	Ret. Time [min]	Area [mV *s]	Area %
3	9.618	224.510	6.438
4	9.929	834.339	23.924
5	10.004	1554.351	44.569
6	10.134	99.216	2.845
7	10.307	10.441	0.299

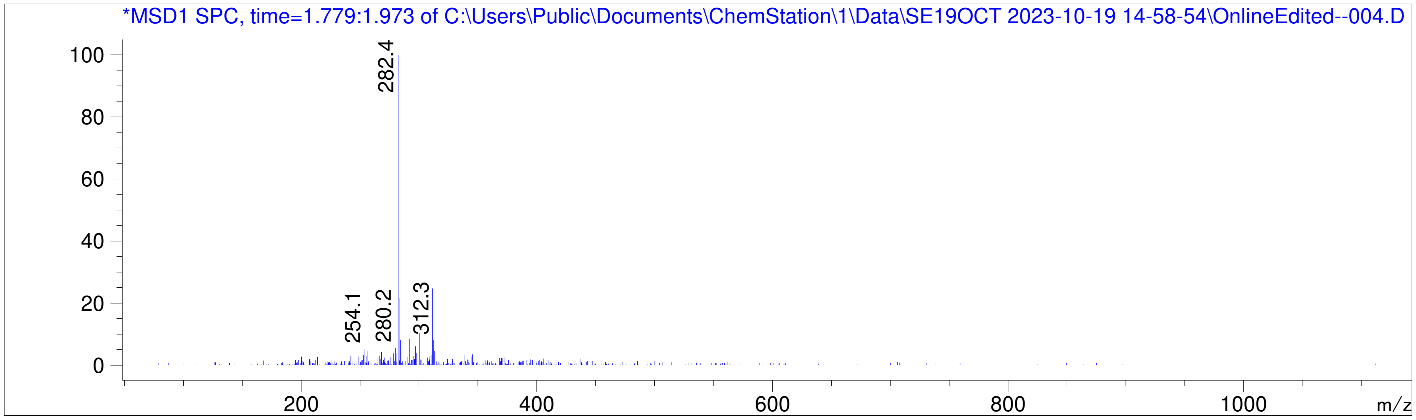
*MSD1 SPC, time=1.779:1.973 of C:\Users\Public\Documents\ChemStation\1\Data\SE19OCT 2023-10-19 14-58-54\OnlineEdited--004.D
282.4
254.1
280.2
312.3
m/z

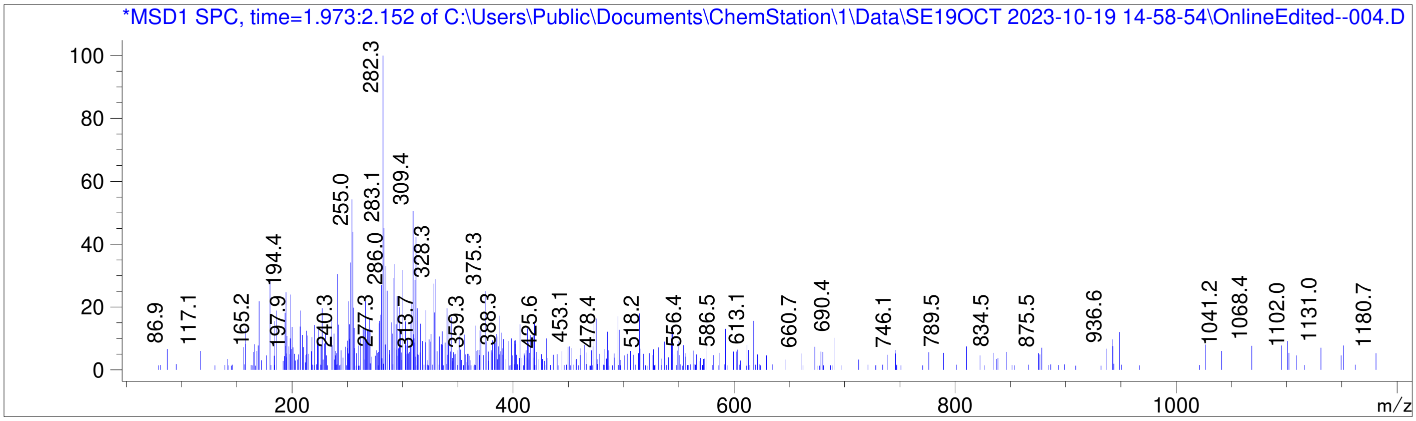
*MSD1 SPC, time=1.973:2.152 of C:\Users\Public\Documents\ChemStation\1\Data\SE19OCT 2023-10-19 14-58-54\OnlineEdited--004.D
282.3
255.0
283.1
309.4
194.4
286.0
328.3
375.3
m/z

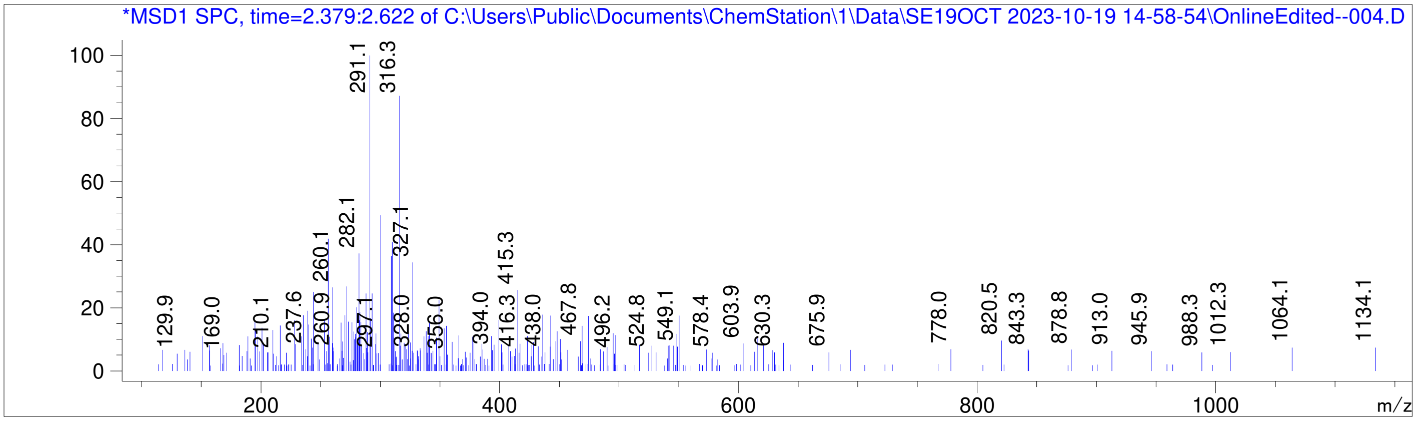
*MSD1 SPC, time=2.379:2.622 of C:\Users\Public\Documents\ChemStation\1\Data\SE19OCT 2023-10-19 14-58-54\OnlineEdited--004.D
291.1
316.3
282.1
327.1
260.1
415.3
m/z

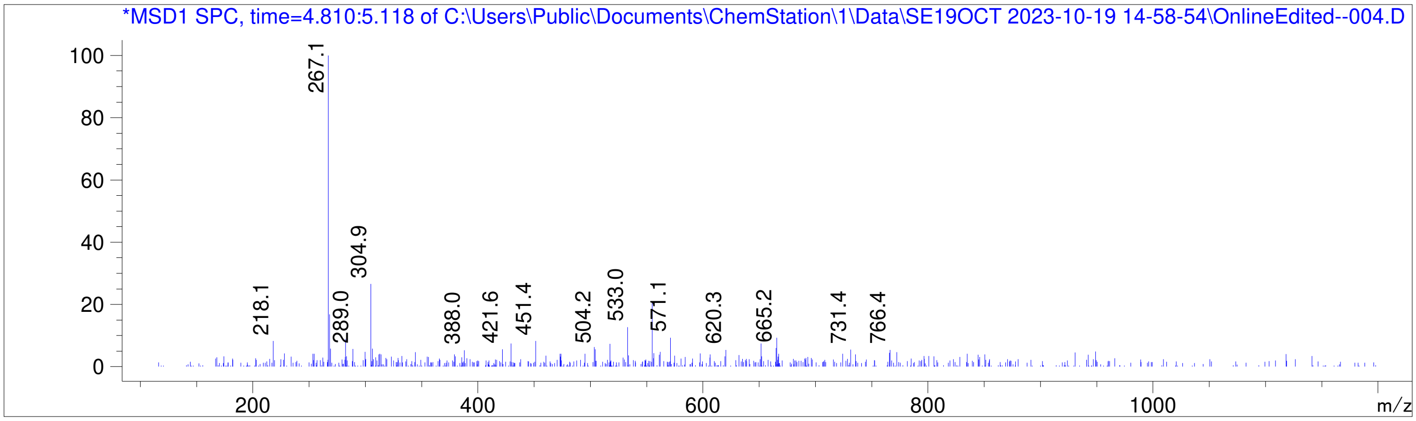
*MSD1 SPC, time=4.810:5.118 of C:\Users\Public\Documents\ChemStation\1\Data\SE19OCT 2023-10-19 14-58-54\OnlineEdited--004.D
267.1
304.9
218.1
289.0
388.0
421.6
451.4
504.2
533.0
571.1
620.3
665.2
731.4
766.4
m/z

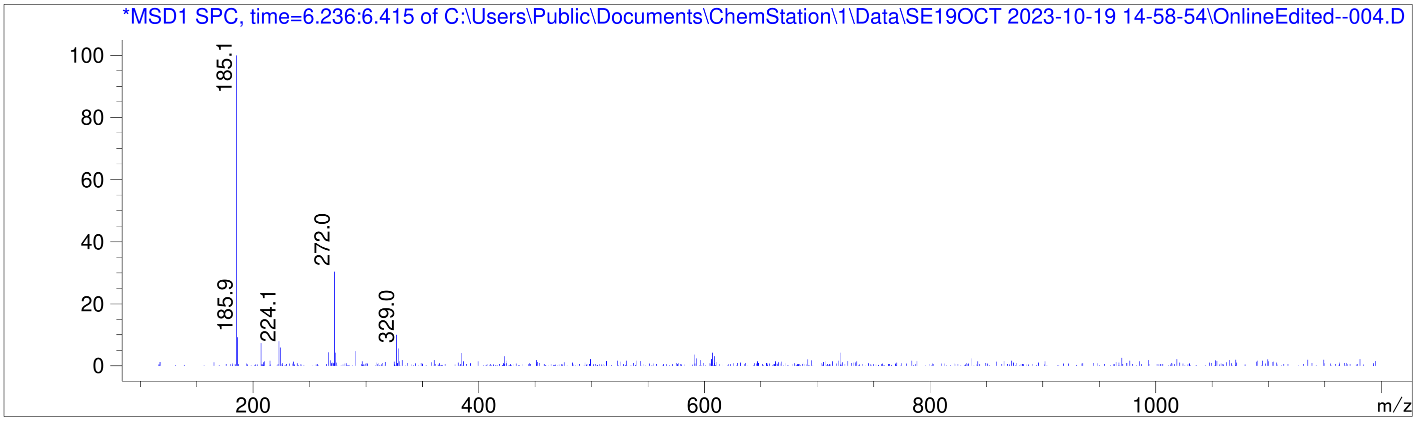
*MSD1 SPC, time=6.236:6.415 of C:\Users\Public\Documents\ChemStation\1\Data\SE19OCT 2023-10-19 14-58-54\OnlineEdited--004.D
185.1
272.0
185.9
224.1
329.0
m/z

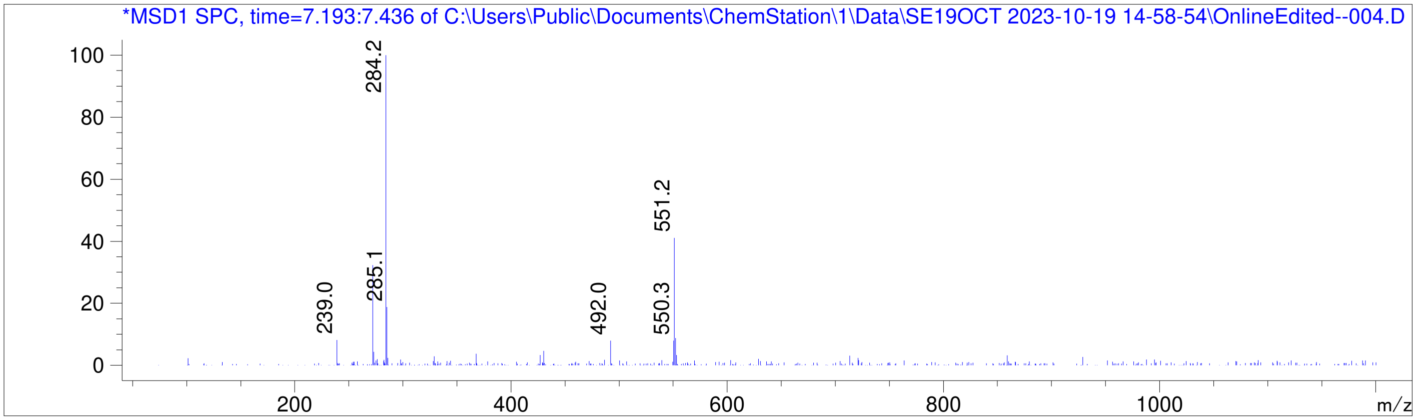
*MSD1 SPC, time=7.193:7.436 of C:\Users\Public\Documents\ChemStation\1\Data\SE19OCT 2023-10-19 14-58-54\OnlineEdited--004.D
284.2
551.2
285.1
239.0
492.0
550.3
m/z

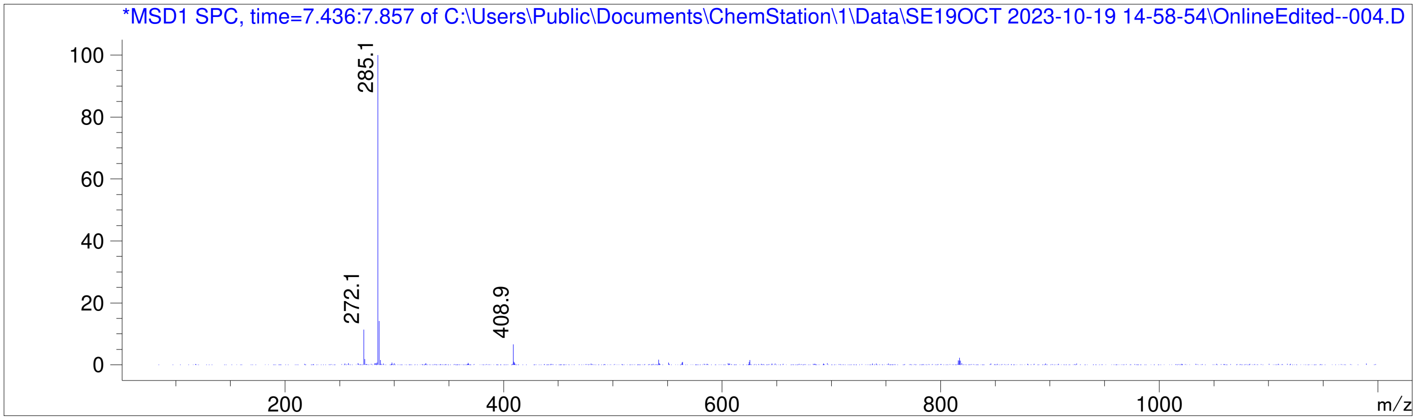
*MSD1 SPC, time=7.436:7.857 of C:\Users\Public\Documents\ChemStation\1\Data\SE19OCT 2023-10-19 14-58-54\OnlineEdited--004.D
285.1
272.1
408.9
m/z

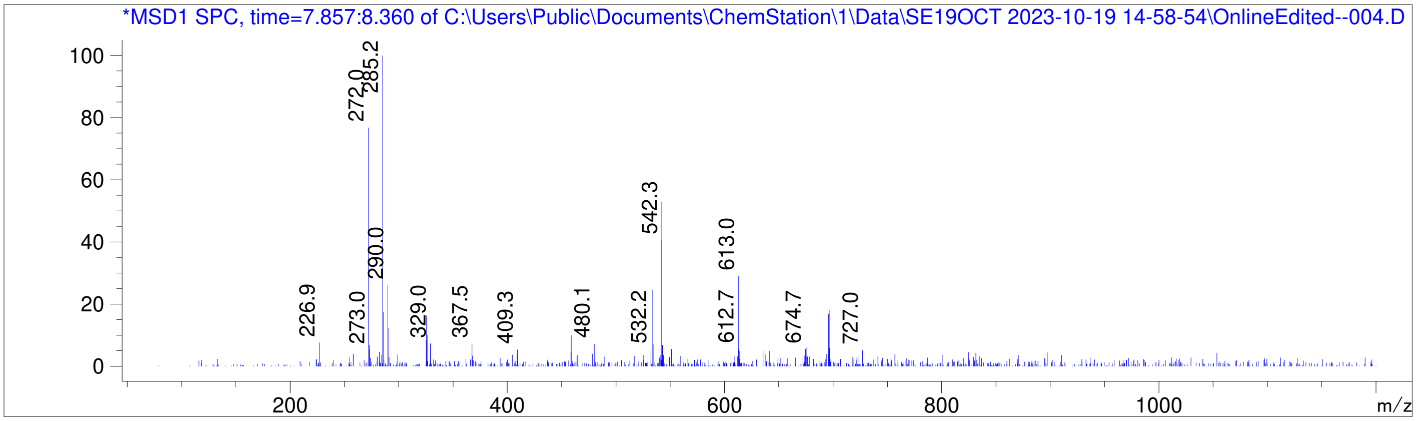
*MSD1 SPC, time=7.857:8.360 of C:\Users\Public\Documents\ChemStation\1\Data\SE19OCT 2023-10-19 14-58-54\OnlineEdited--004.D
285.2
272.0
542.3
613.0
290.0
226.9
273.0
329.0
367.5
409.3
480.1
532.2
612.7
674.7
727.0
m/z

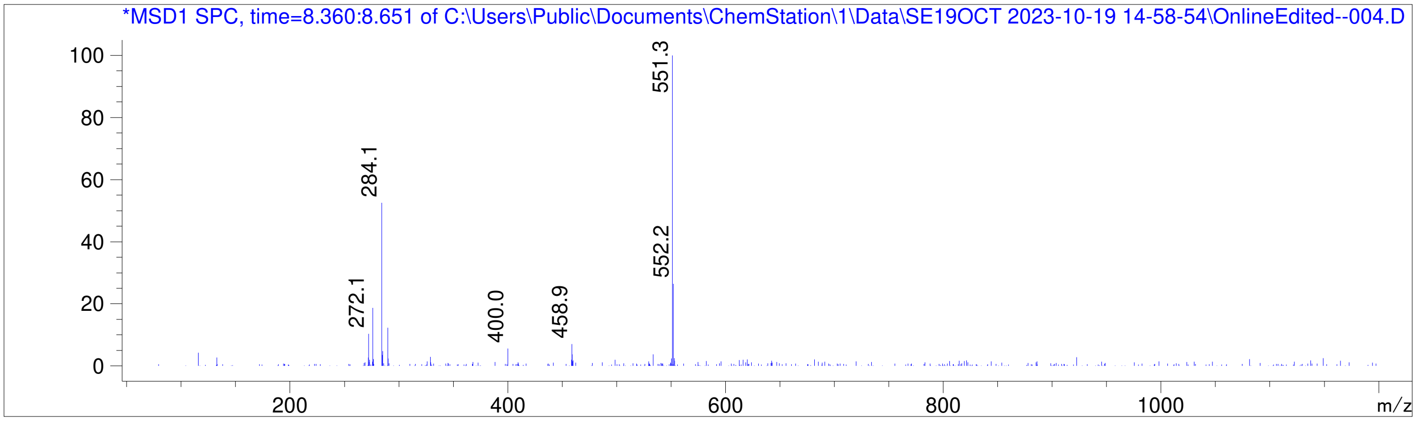
*MSD1 SPC, time=8.360:8.651 of C:\Users\Public\Documents\ChemStation\1\Data\SE19OCT 2023-10-19 14-58-54\OnlineEdited--004.D
551.3
284.1
552.2
272.1
400.0
458.9
m/z

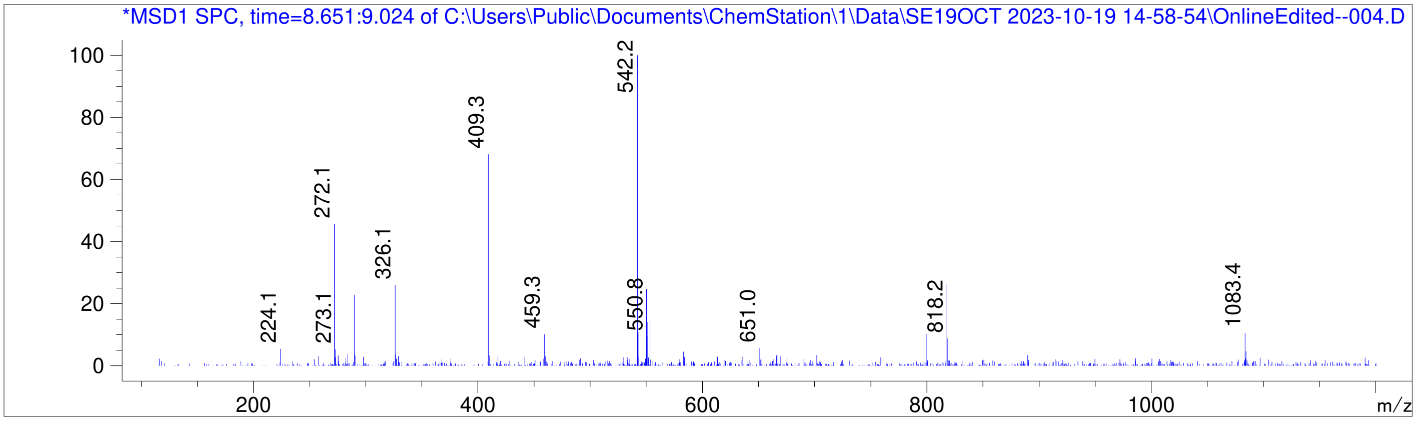
*MSD1 SPC, time=8.651:9.024 of C:\Users\Public\Documents\ChemStation\1\Data\SE19OCT 2023-10-19 14-58-54\OnlineEdited--004.D
542.2
409.3
272.1
326.1
224.1
273.1
459.3
550.8
651.0
818.2
1083.4
m/z

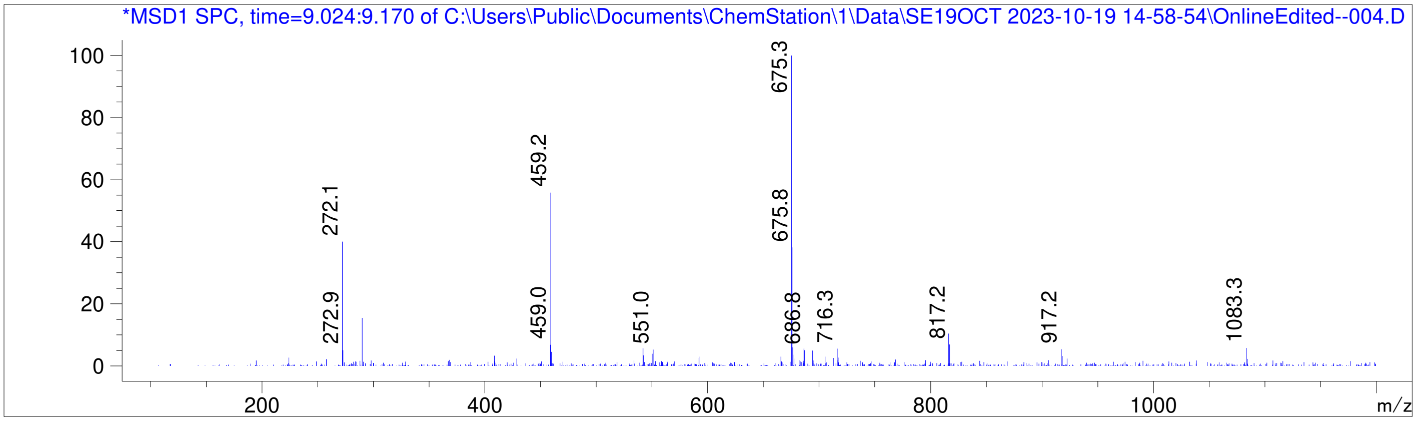
*MSD1 SPC, time=9.024:9.170 of C:\Users\Public\Documents\ChemStation\1\Data\SE19OCT 2023-10-19 14-58-54\OnlineEdited--004.D
675.3
459.2
272.1
675.8
272.9
459.0
551.0
686.8
716.3
817.2
917.2
1083.3
m/z

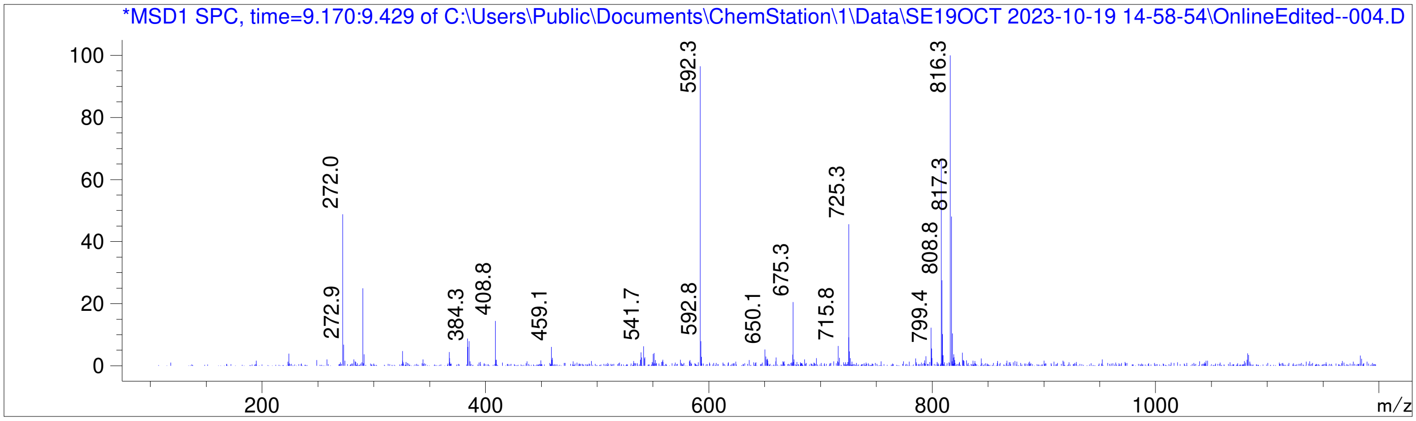
*MSD1 SPC, time=9.170:9.429 of C:\Users\Public\Documents\ChemStation\1\Data\SE19OCT 2023-10-19 14-58-54\OnlineEdited--004.D
592.3
816.3
272.0
725.3
817.3
808.8
272.9
384.3
408.8
459.1
541.7
592.8
650.1
675.3
715.8
799.4
m/z

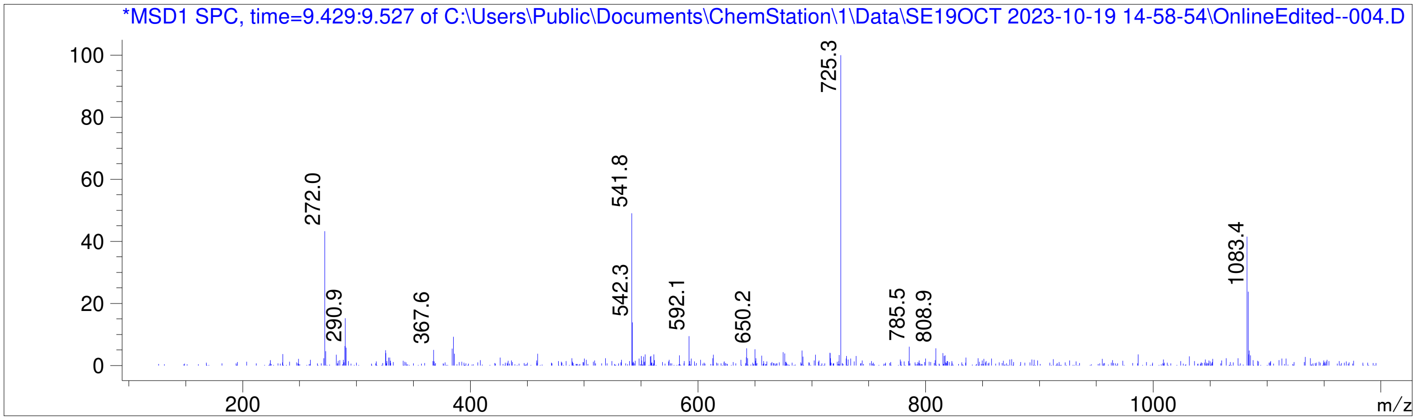
*MSD1 SPC, time=9.429:9.527 of C:\Users\Public\Documents\ChemStation\1\Data\SE19OCT 2023-10-19 14-58-54\OnlineEdited--004.D
725.3
541.8
272.0
1083.4
542.3
290.9
367.6
592.1
650.2
785.5
808.9
m/z

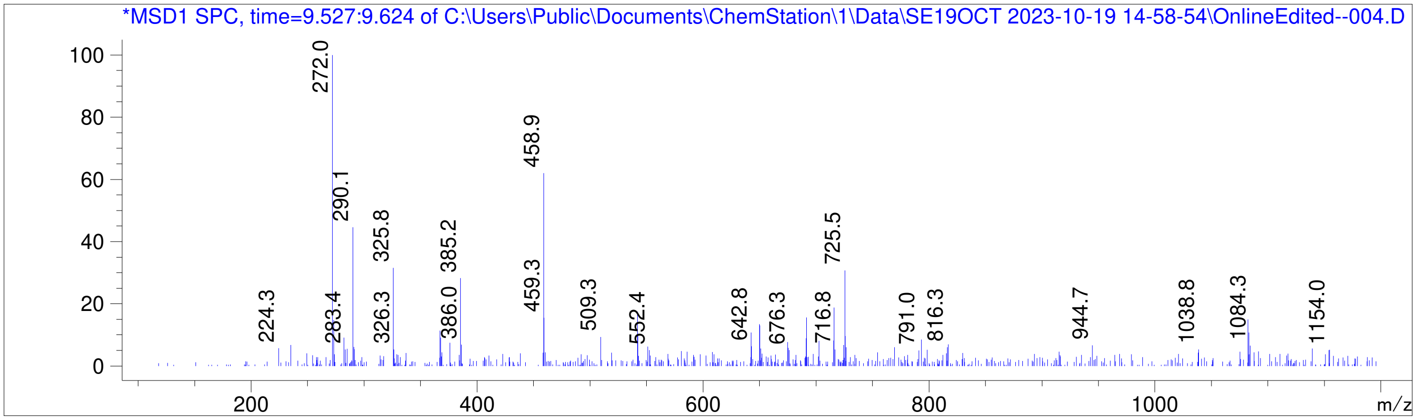
*MSD1 SPC, time=9.527:9.624 of C:\Users\Public\Documents\ChemStation\1\Data\SE19OCT 2023-10-19 14-58-54\OnlineEdited--004.D
272.0
458.9
290.1
325.8
385.2
725.5
224.3
283.4
326.3
386.0
459.3
509.3
552.4
642.8
676.3
716.8
791.0
816.3
944.7
1038.8
1084.3
1154.0
m/z

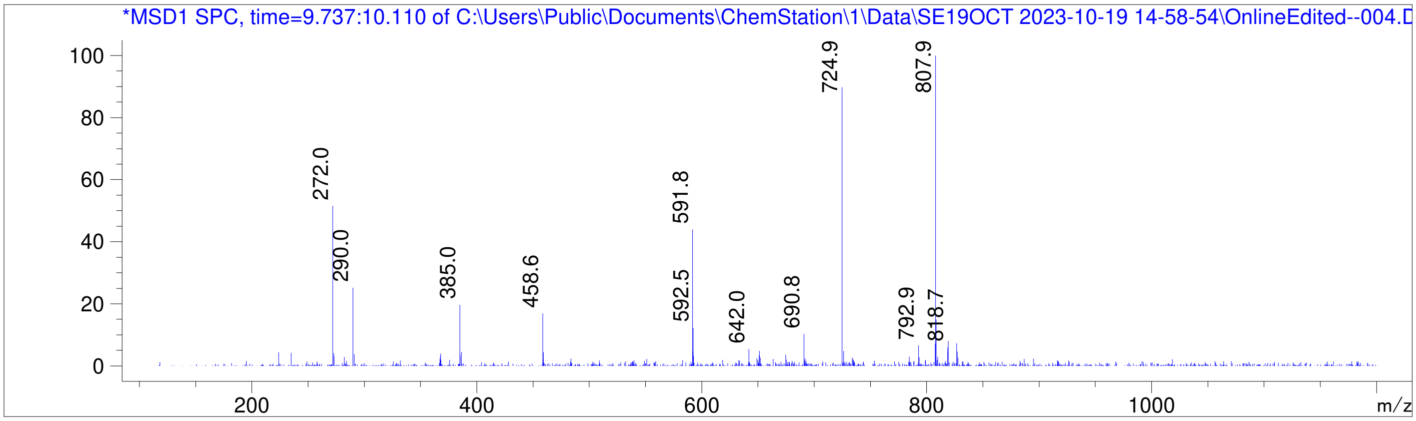
*MSD1 SPC, time=9.737:10.110 of C:\Users\Public\Documents\ChemStation\1\Data\SE19OCT 2023-10-19 14-58-54\OnlineEdited--004.D
100
80
60
40
20
0
272.0
290.0
385.0
458.6
591.8
592.5
642.0
690.8
724.9
792.9
807.9
818.7
200
400
600
800
1000
m/z

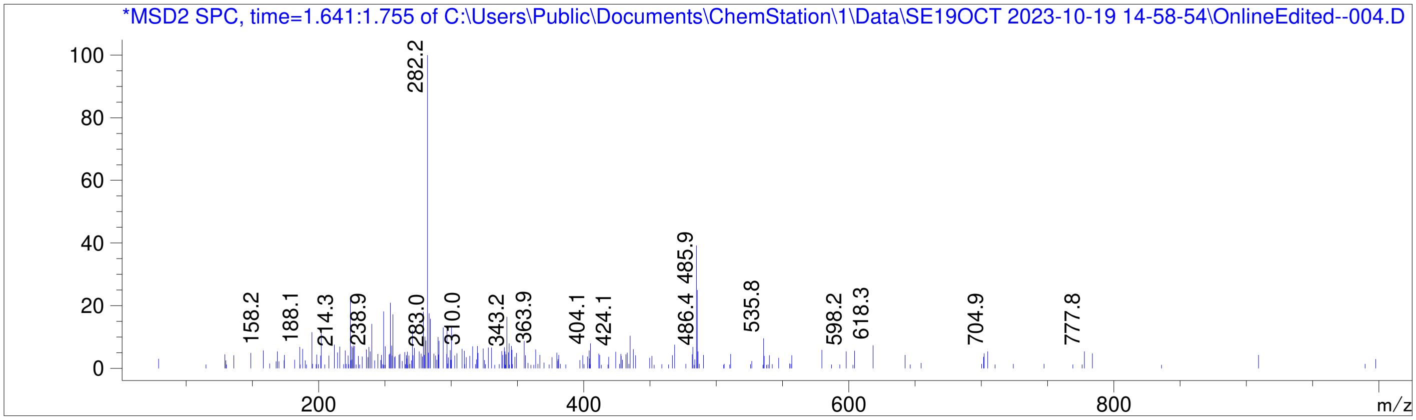
*MSD2 SPC, time=1.641:1.755 of C:\Users\Public\Documents\ChemStation\1\Data\SE19OCT 2023-10-19 14-58-54\OnlineEdited--004.D
282.2
485.9
158.2
188.1
214.3
238.9
283.0
310.0
343.2
363.9
404.1
424.1
486.4
535.8
598.2
618.3
704.9
777.8
m/z

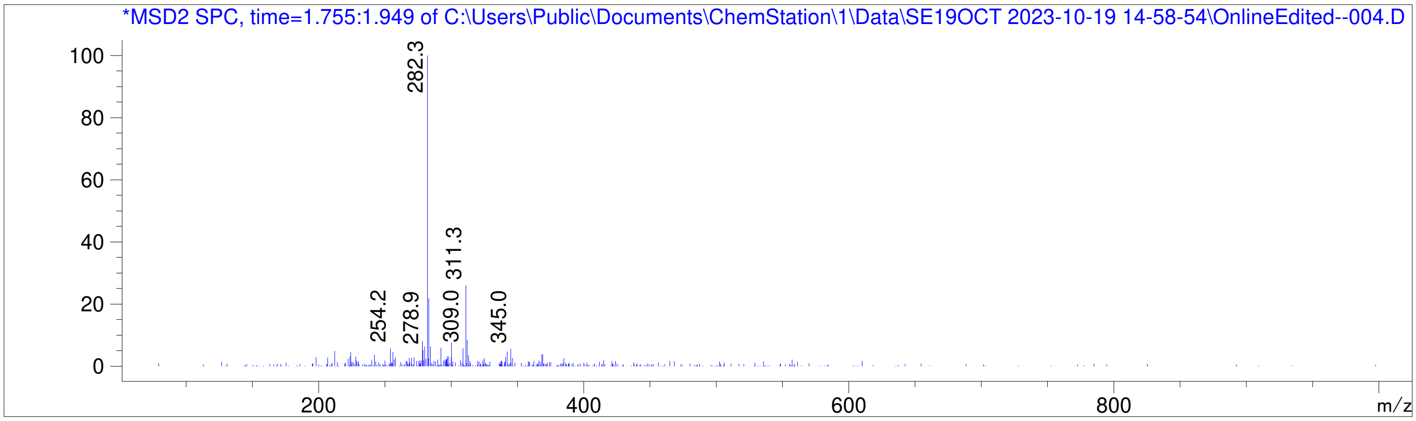
*MSD2 SPC, time=1.755:1.949 of C:\Users\Public\Documents\ChemStation\1\Data\SE19OCT 2023-10-19 14-58-54\OnlineEdited--004.D
282.3
311.3
254.2
278.9
309.0
345.0
m/z

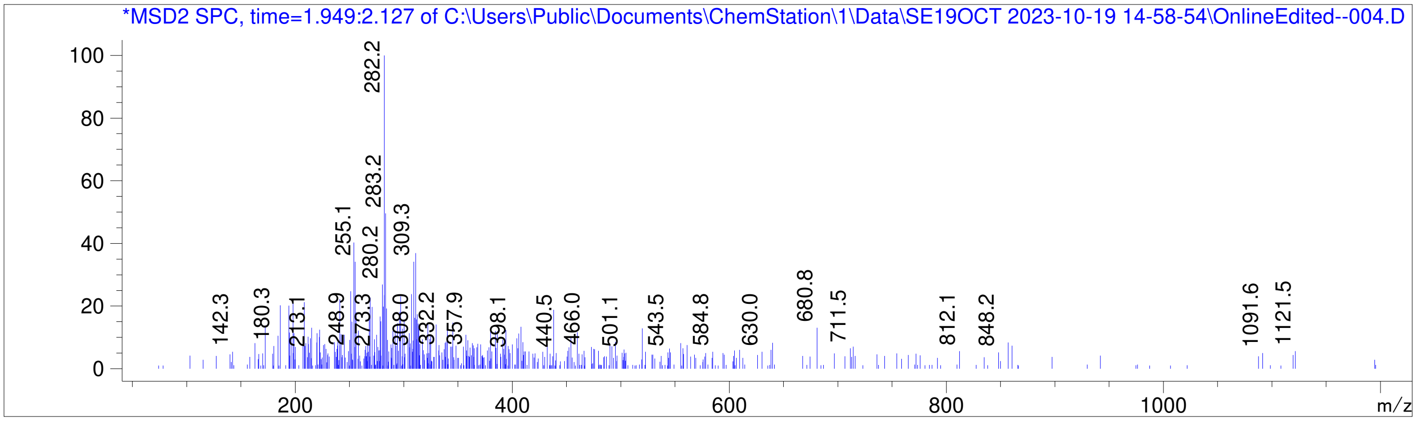
*MSD2 SPC, time=1.949:2.127 of C:\Users\Public\Documents\ChemStation\1\Data\SE19OCT 2023-10-19 14-58-54\OnlineEdited--004.D
282.2
283.2
255.1
280.2
309.3
142.3
180.3
213.1
248.9
273.3
308.0
332.2
357.9
398.1
440.5
466.0
501.1
543.5
584.8
630.0
680.8
711.5
812.1
848.2
1091.6
1121.5
m/z

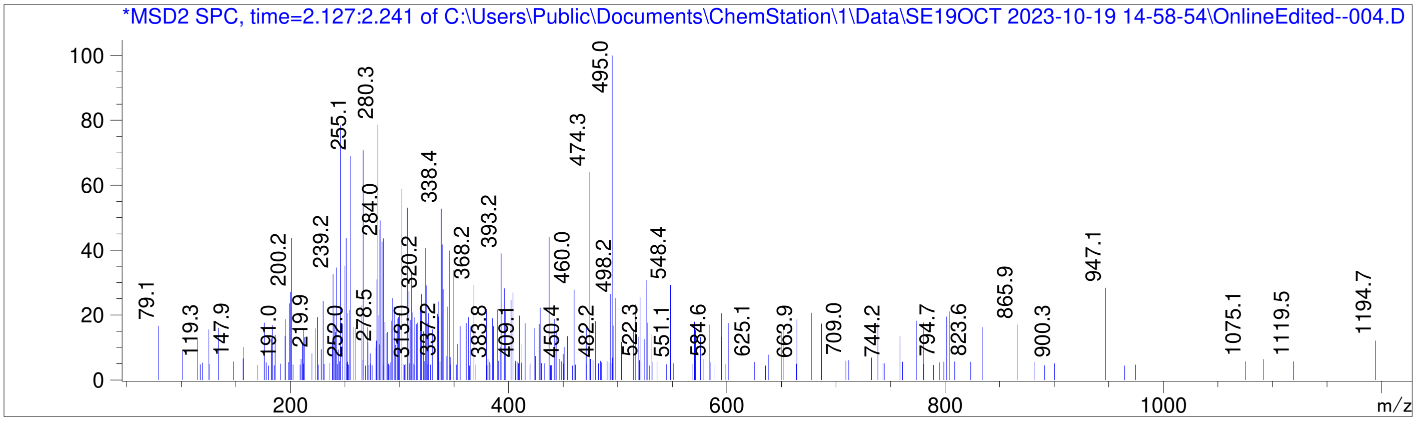
*MSD2 SPC, time=2.127:2.241 of C:\Users\Public\Documents\ChemStation\1\Data\SE19OCT 2023-10-19 14-58-54\OnlineEdited--004.D
79.1
119.3
147.9
191.0
200.2
219.9
239.2
252.0
255.1
278.5
280.3
284.0
313.0
320.2
337.2
338.4
368.2
383.8
393.2
409.1
450.4
460.0
474.3
482.2
495.0
498.2
522.3
548.4
551.1
584.6
625.1
663.9
709.0
744.2
794.7
823.6
865.9
900.3
947.1
1075.1
1119.5
1194.7
m/z

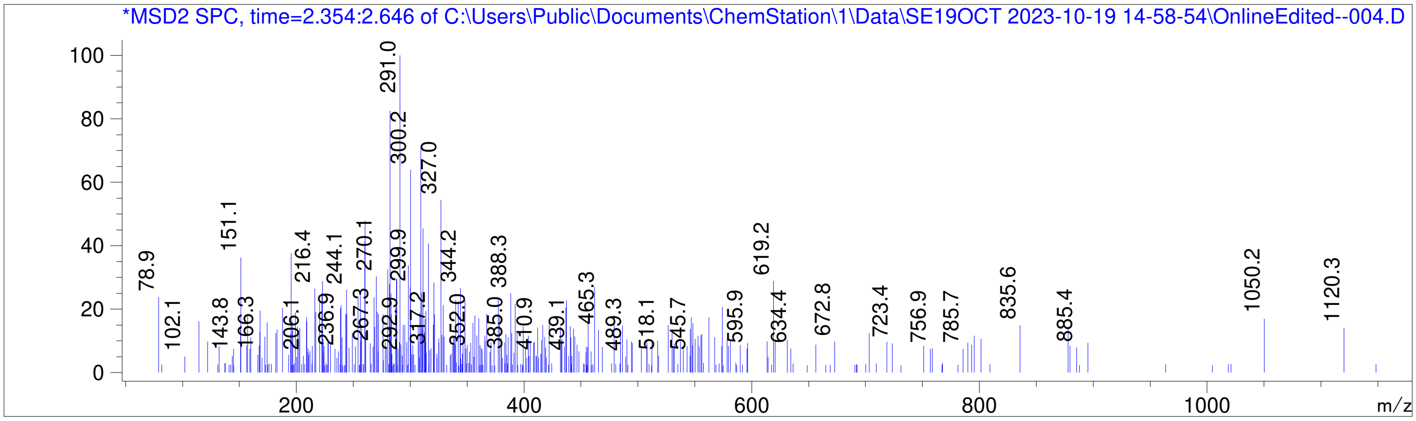
*MSD2 SPC, time=2.354:2.646 of C:\Users\Public\Documents\ChemStation\1\Data\SE19OCT 2023-10-19 14-58-54\OnlineEdited--004.D
78.9
102.1
143.8
151.1
166.3
206.1
216.4
236.9
244.1
267.3
270.1
291.0
292.9
299.9
300.2
317.2
327.0
344.2
352.0
385.0
388.3
410.9
439.1
465.3
489.3
518.1
545.7
595.9
619.2
634.4
672.8
723.4
756.9
785.7
835.6
885.4
1050.2
1120.3
m/z

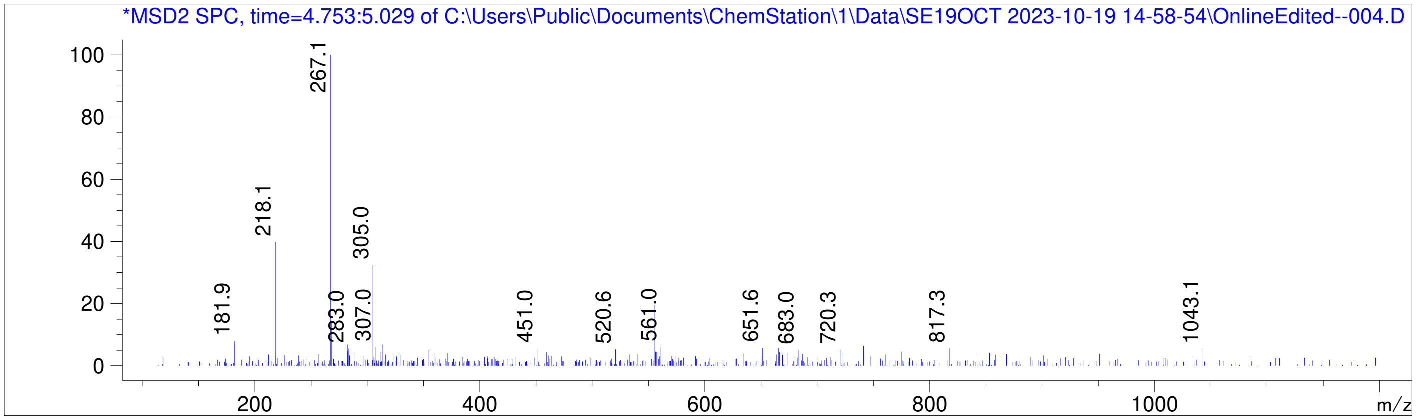
*MSD2 SPC, time=4.753:5.029 of C:\Users\Public\Documents\ChemStation\1\Data\SE19OCT 2023-10-19 14-58-54\OnlineEdited--004.D
181.9
218.1
267.1
283.0
305.0
307.0
451.0
520.6
561.0
651.6
683.0
720.3
817.3
1043.1
m/z

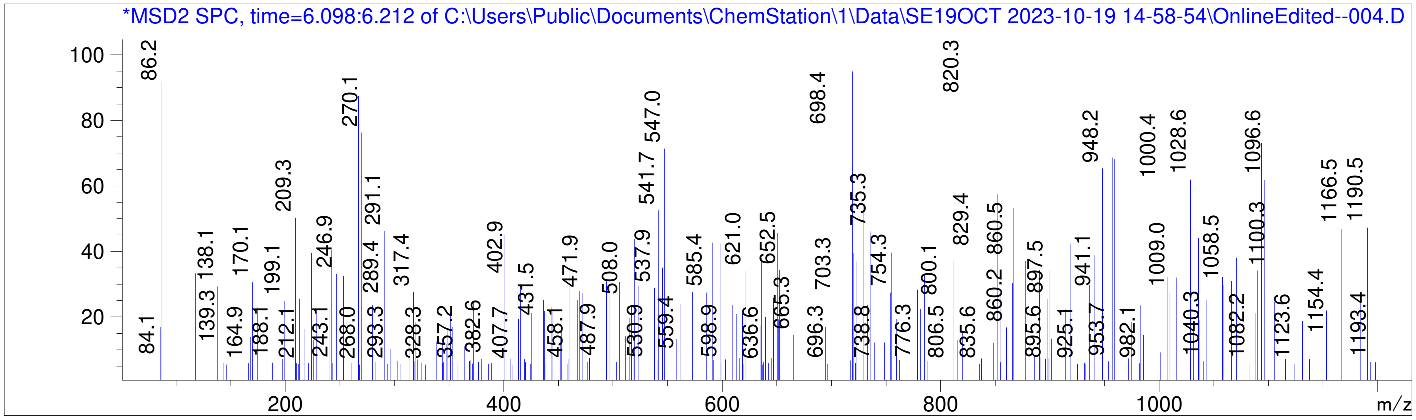
*MSD2 SPC, time=6.098:6.212 of C:\Users\Public\Documents\ChemStation\1\Data\SE19OCT 2023-10-19 14-58-54\OnlineEdited--004.D
84.1
86.2
138.1
139.3
164.9
170.1
188.1
199.1
209.3
212.1
243.1
246.9
268.0
270.1
289.4
291.1
293.3
317.4
328.3
357.2
382.6
402.9
407.7
431.5
458.1
471.9
487.9
508.0
530.9
537.9
541.7
547.0
559.4
585.4
598.9
621.0
636.6
652.5
665.3
696.3
698.4
703.3
735.3
738.8
754.3
776.3
800.1
806.5
820.3
829.4
835.6
860.2
860.5
895.6
897.5
925.1
941.1
948.2
953.7
982.1
1000.4
1009.0
1028.6
1040.3
1058.5
1082.2
1096.6
1100.3
1123.6
1154.4
1166.5
1190.5
1193.4
m/z

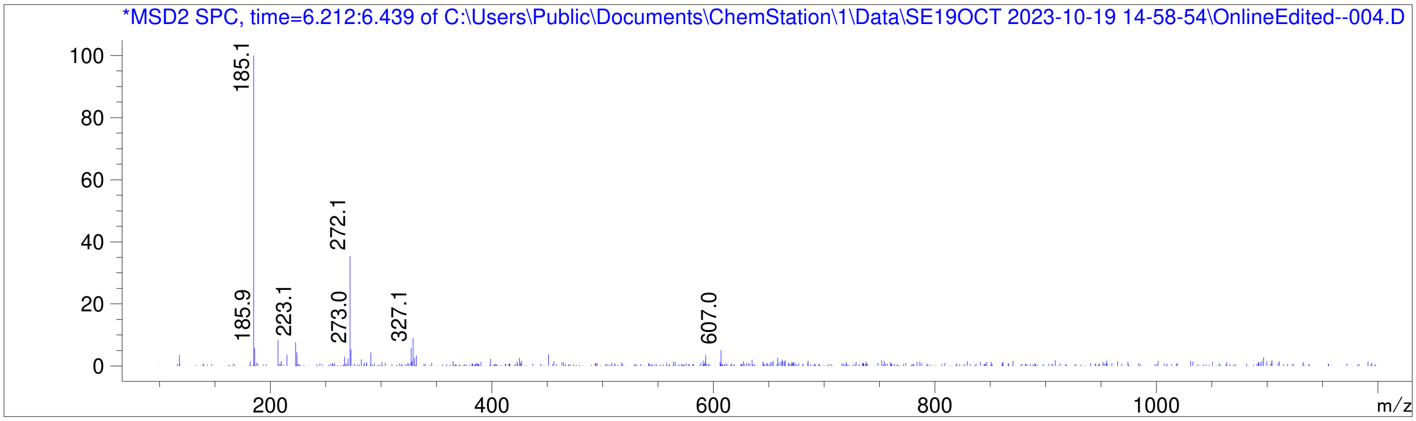
*MSD2 SPC, time=6.212:6.439 of C:\Users\Public\Documents\ChemStation\1\Data\SE19OCT 2023-10-19 14-58-54\OnlineEdited--004.D
185.1
185.9
223.1
272.1
273.0
327.1
607.0
m/z

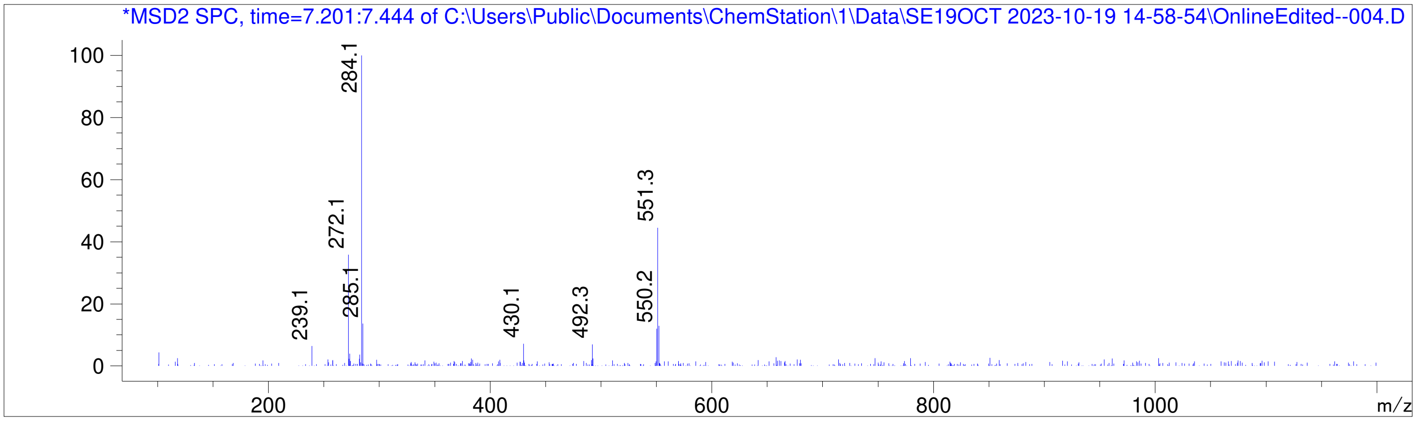
*MSD2 SPC, time=7.201:7.444 of C:\Users\Public\Documents\ChemStation\1\Data\SE19OCT 2023-10-19 14-58-54\OnlineEdited--004.D
284.1
272.1
285.1
239.1
430.1
492.3
551.3
550.2
m/z

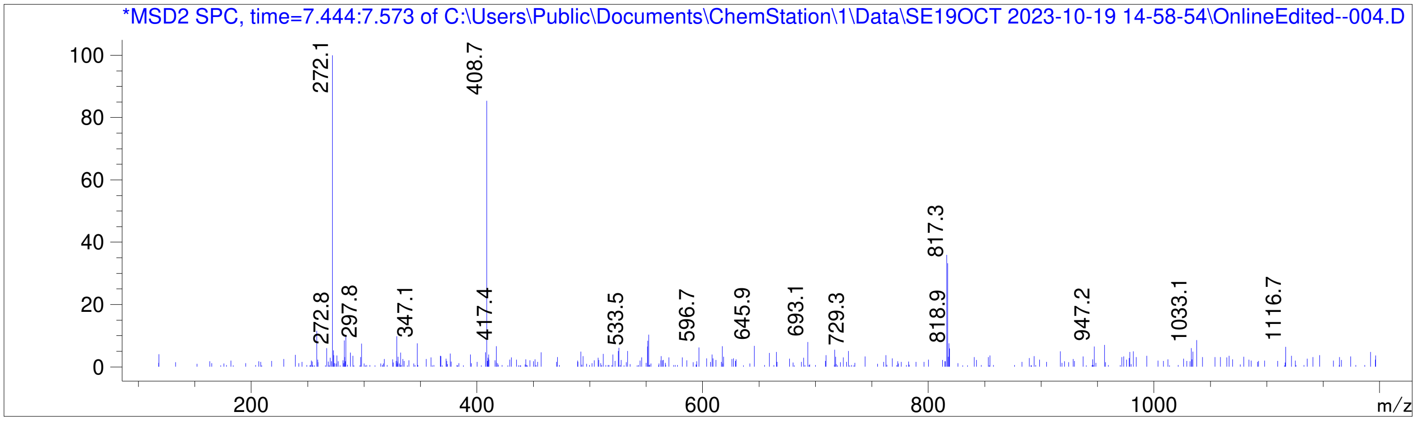
*MSD2 SPC, time=7.444:7.573 of C:\Users\Public\Documents\ChemStation\1\Data\SE19OCT 2023-10-19 14-58-54\OnlineEdited--004.D
272.1
408.7
272.8
297.8
347.1
417.4
533.5
596.7
645.9
693.1
729.3
817.3
818.9
947.2
1033.1
1116.7
m/z

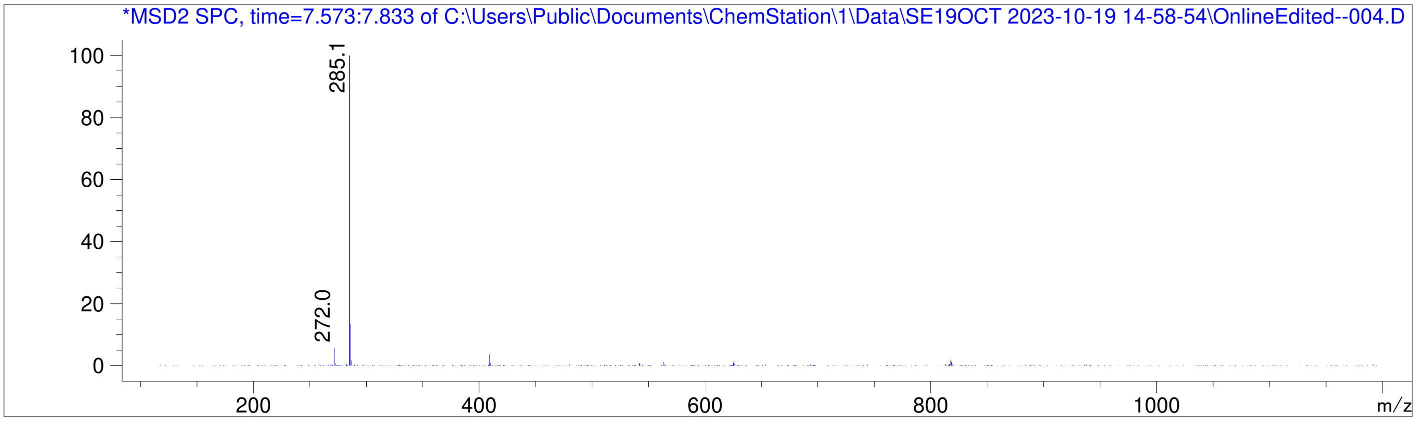
*MSD2 SPC, time=7.573:7.833 of C:\Users\Public\Documents\ChemStation\1\Data\SE19OCT 2023-10-19 14-58-54\OnlineEdited--004.D
285.1
272.0
m/z

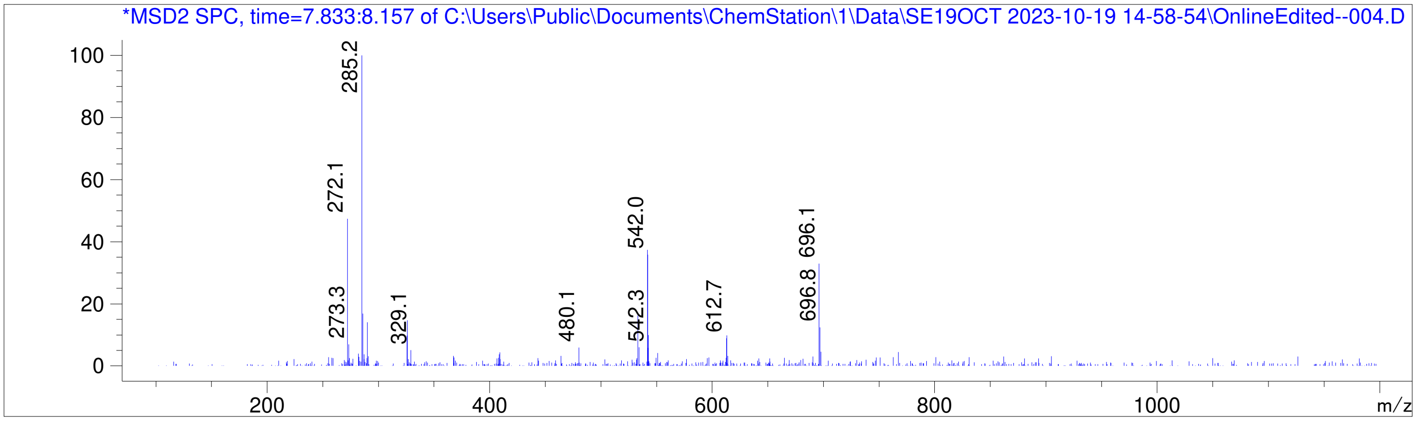
*MSD2 SPC, time=7.833:8.157 of C:\Users\Public\Documents\ChemStation\1\Data\SE19OCT 2023-10-19 14-58-54\OnlineEdited--004.D
285.2
272.1
273.3
329.1
480.1
542.0
542.3
612.7
696.1
696.8
m/z

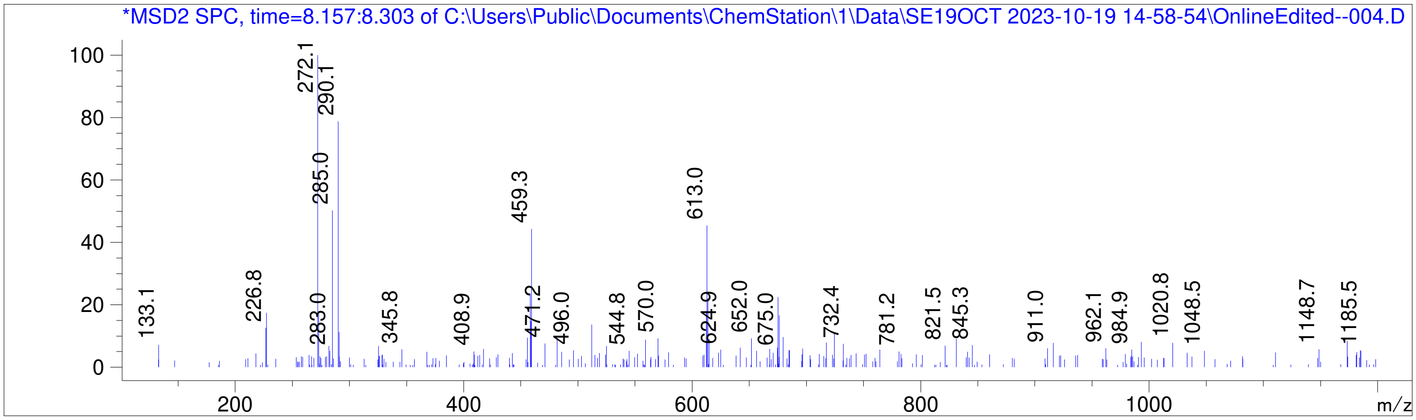
*MSD2 SPC, time=8.157:8.303 of C:\Users\Public\Documents\ChemStation\1\Data\SE19OCT 2023-10-19 14-58-54\OnlineEdited--004.D
133.1
226.8
272.1
290.1
285.0
283.0
345.8
408.9
459.3
471.2
496.0
544.8
570.0
613.0
624.9
652.0
675.0
732.4
781.2
821.5
845.3
911.0
962.1
984.9
1020.8
1048.5
1148.7
1185.5
m/z

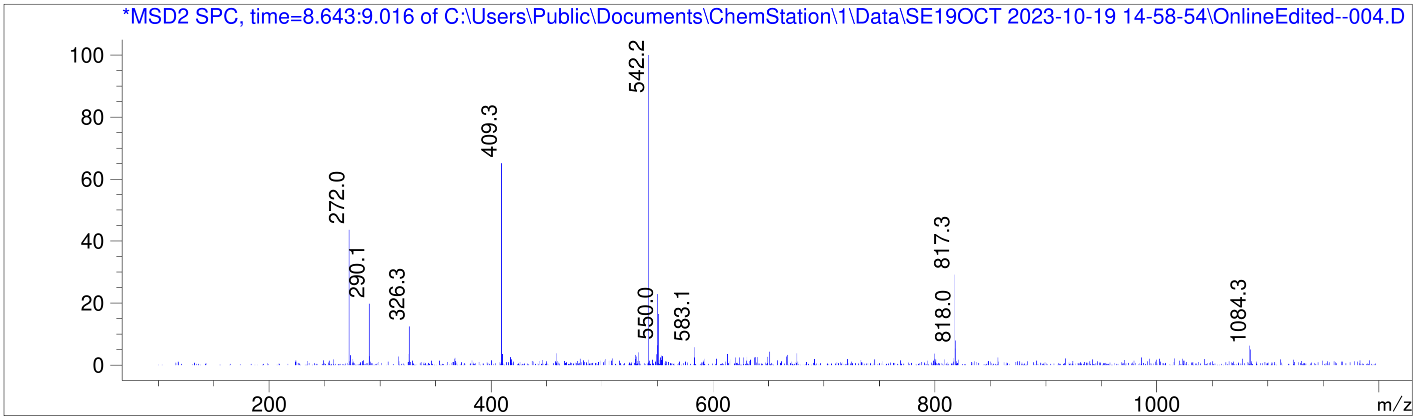
*MSD2 SPC, time=8.643:9.016 of C:\Users\Public\Documents\ChemStation\1\Data\SE19OCT 2023-10-19 14-58-54\OnlineEdited--004.D
272.0
290.1
326.3
409.3
542.2
550.0
583.1
817.3
818.0
1084.3
m/z

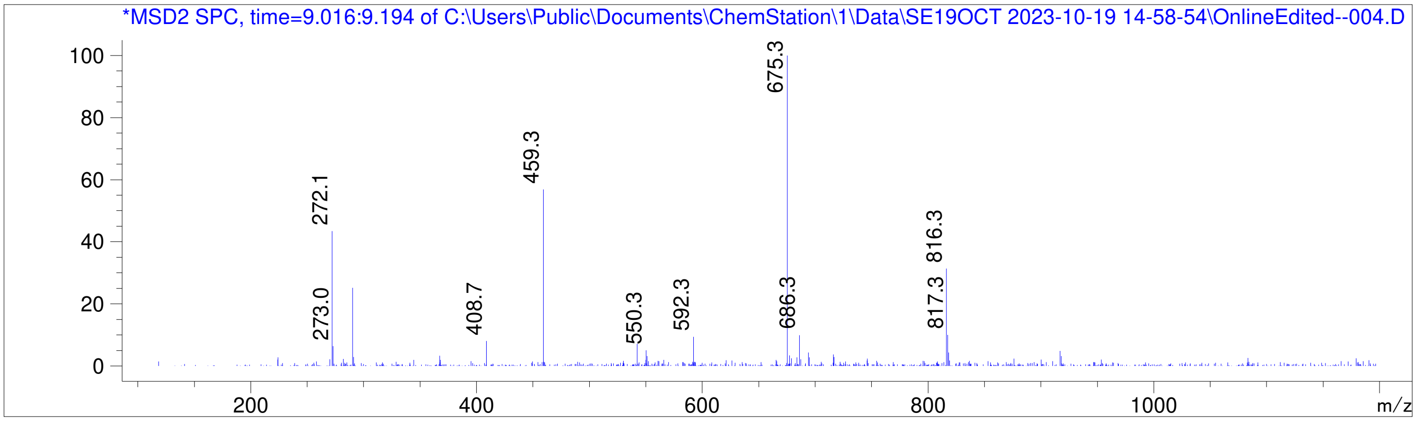
*MSD2 SPC, time=9.016:9.194 of C:\Users\Public\Documents\ChemStation\1\Data\SE19OCT 2023-10-19 14-58-54\OnlineEdited--004.D
675.3
459.3
272.1
816.3
273.0
408.7
550.3
592.3
686.3
817.3
m/z

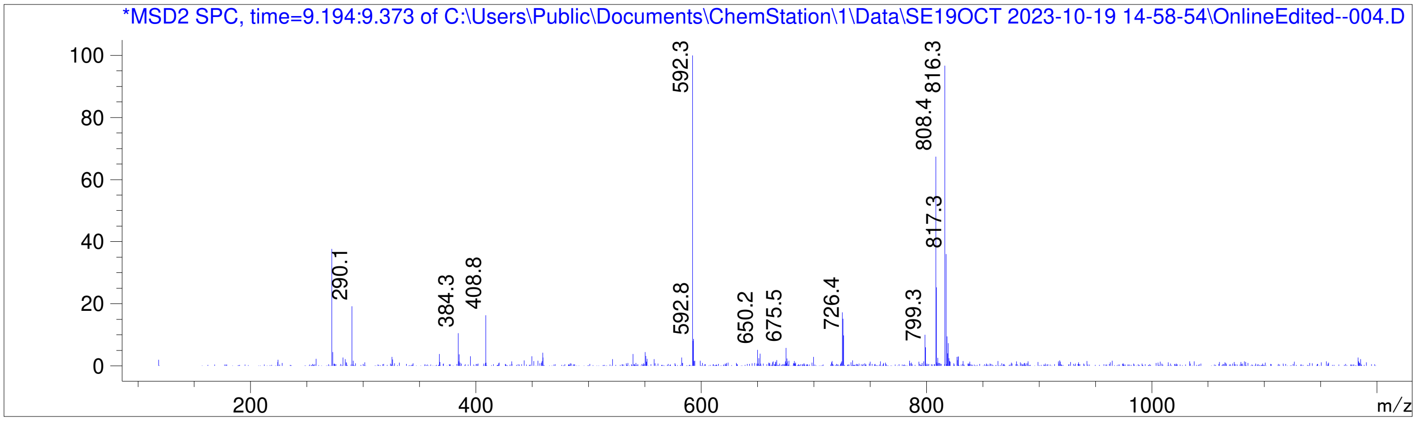
*MSD2 SPC, time=9.194:9.373 of C:\Users\Public\Documents\ChemStation\1\Data\SE19OCT 2023-10-19 14-58-54\OnlineEdited--004.D
592.3
816.3
808.4
817.3
290.1
384.3
408.8
592.8
650.2
675.5
726.4
799.3
m/z

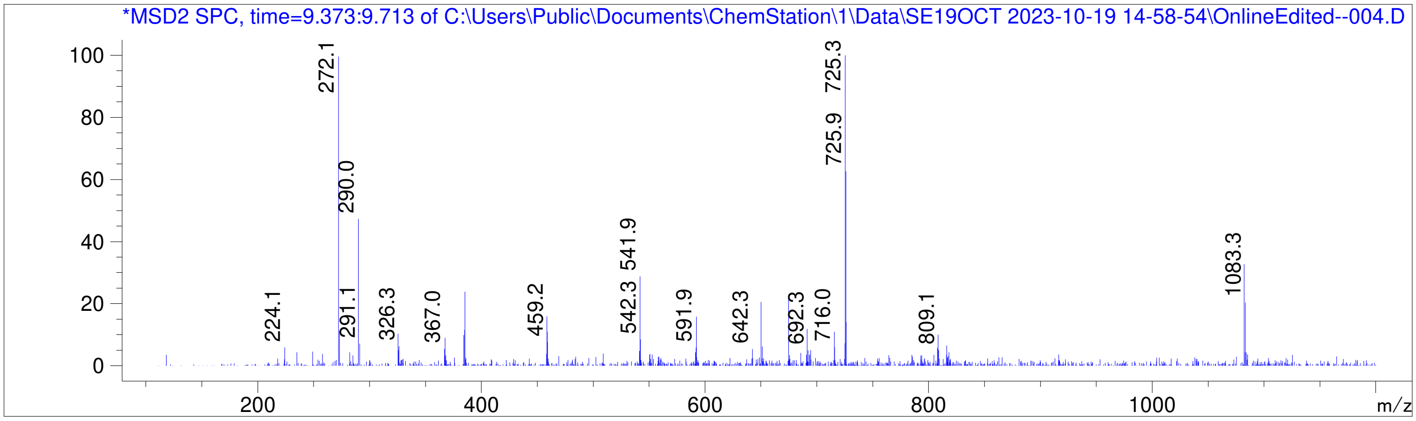
*MSD2 SPC, time=9.373:9.713 of C:\Users\Public\Documents\ChemStation\1\Data\SE19OCT 2023-10-19 14-58-54\OnlineEdited--004.D
272.1
725.3
725.9
290.0
541.9
1083.3
224.1
291.1
326.3
367.0
459.2
542.3
591.9
642.3
692.3
716.0
809.1
m/z

===

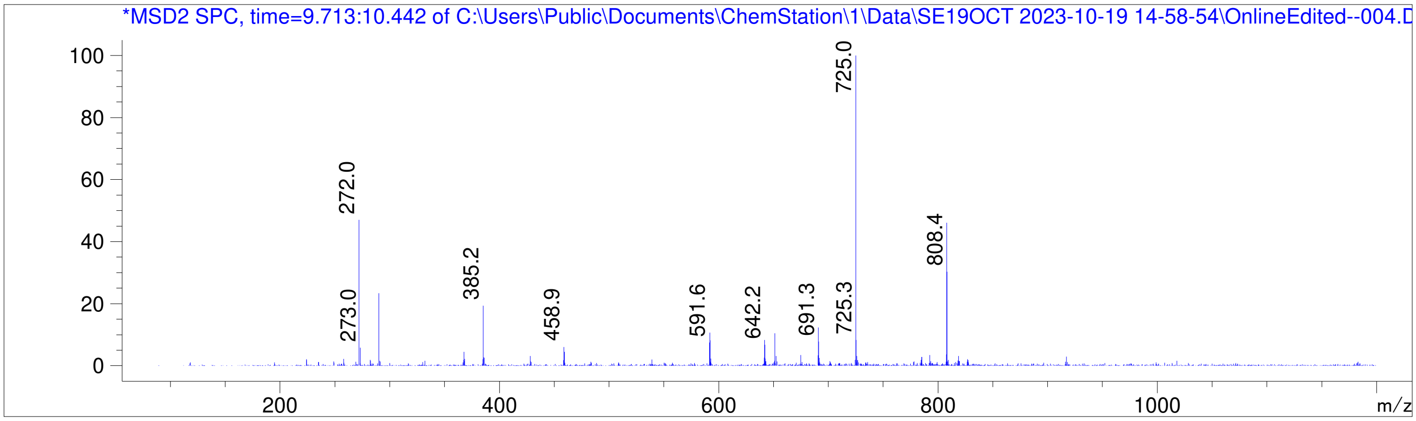
*MSD2 SPC, time=9.713:10.442 of C:\Users\Public\Documents\ChemStation\1\Data\SE19OCT 2023-10-19 14-58-54\OnlineEdited--004.D
100
80
60
40
20
0
725.0
272.0
808.4
385.2
273.0
458.9
591.6
642.2
691.3
725.3
200
400
600
800
1000
m/z
