# Supplementary material for: Astrobiological implications of the stability and reactivity of peptide nucleic acid (PNA) in concentrated sulfuric acid
Source: Sci Adv. 2025 Mar 26;11(13):eadr0006. doi: 10.1126/sciadv.adr0006 (PMC11939054; doi:10.1126/sciadv.adr0006)

=====================================================================

Injection Date : Fri, 20. Oct. 2023    Seq Line : 5
                                       Location : 27
                                       Inj. Vol. : 2 µl

Acq. Method : C:\Users\Public\Documents\ChemStation\1\Data\SE20OCT 2023-10-20 14-51-25\22010446C LCMS-6#.M

Analysis Method : C:\Users\Public\Documents\ChemStation\1\Data\SE20OCT\SE20OCT 2023-10-20 14-51-25\22010446C LCMS-6#.M (Sequence Method)

Waters XBridge BEH Amide (4.6 x 150 mm, 2.5 µm); PN# 186006726

Mobile Phase A: 20mM Ammonium Acetate (aq) pH 8.2

Mobile Phase B: AcN

Mobile Phase A / Mobile Phase B: 5/95 (0 min) --> (10 min) --> 60/40 (5 min); Flow: 1.0 ml/min; MSD1 = positive; MSD2 = negative

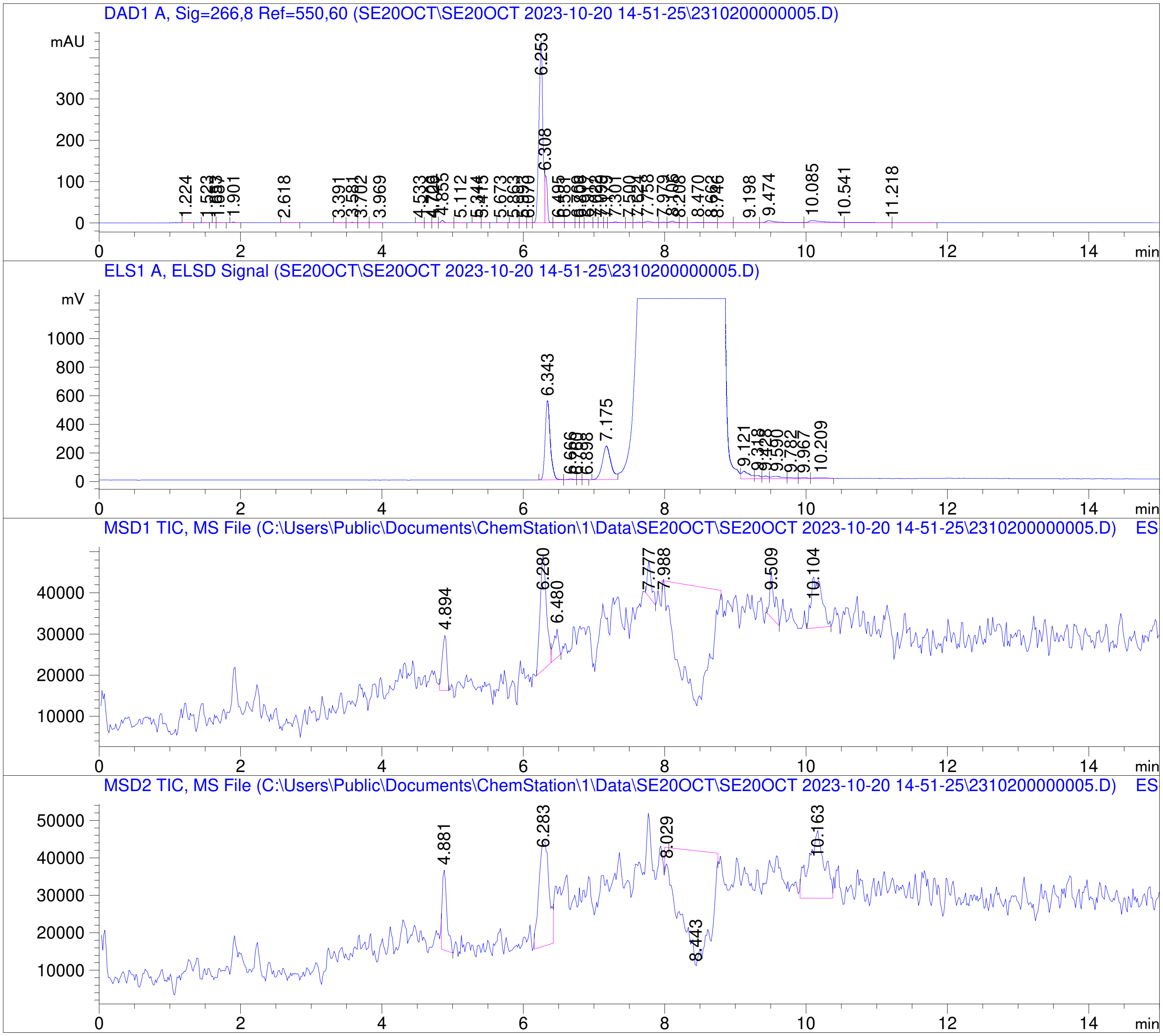

DAD1 A, Sig=266,8 Ref=550,60

| Peak # | Ret. Time [min] | Area [mV *s] | Area % |
|---|---|---|---|
| 1 | 1.224 | 0.304 | 0.013 |
| 2 | 1.523 | 0.145 | 0.006 |
| 3 | 1.653 | 0.223 | 0.010 |
| 4 | 1.687 | 1.100 | 0.049 |
| 5 | 1.901 | 3.494 | 0.154 |
| 6 | 2.618 | 0.261 | 0.011 |
| 7 | 3.391 | 0.238 | 0.011 |
| 8 | 3.581 | 3.298 | 0.145 |
| 9 | 3.702 | 0.213 | 0.009 |
| 10 | 3.969 | 0.213 | 0.009 |
| 11 | 4.533 | 0.265 | 0.012 |
| 12 | 4.706 | 0.176 | 0.008 |
| 13 | 4.729 | 0.338 | 0.015 |
| 14 | 4.855 | 15.903 | 0.701 |
| 15 | 5.112 | 0.263 | 0.012 |
| 16 | 5.344 | 0.468 | 0.021 |
| 17 | 5.415 | 0.169 | 0.007 |
| 18 | 5.673 | 0.186 | 0.008 |
| 19 | 5.863 | 0.517 | 0.023 |
| 20 | 5.993 | 0.683 | 0.030 |
| 21 | 6.070 | 0.455 | 0.020 |
| 22 | 6.253 | 1721.817 | 75.886 |
| 23 | 6.308 | 248.645 | 10.959 |
| 24 | 6.495 | 6.342 | 0.279 |
| 25 | 6.581 | 4.599 | 0.203 |
| 26 | 6.769 | 1.511 | 0.067 |
| 27 | 6.806 | 1.460 | 0.064 |
| 28 | 6.917 | 5.654 | 0.249 |
| 29 | 7.022 | 2.626 | 0.116 |
| 30 | 7.099 | 3.439 | 0.152 |
| 31 | 7.179 | 2.350 | 0.104 |
| 32 | 7.301 | 11.410 | 0.503 |
| 33 | 7.500 | 2.509 | 0.111 |
| 34 | 7.624 | 6.632 | 0.292 |
| 35 | 7.758 | 20.197 | 0.890 |
| 36 | 7.979 | 6.495 | 0.286 |
| 37 | 8.106 | 17.333 | 0.764 |
| 38 | 8.208 | 2.663 | 0.117 |
| 39 | 8.470 | 8.588 | 0.378 |
| 40 | 8.662 | 7.809 | 0.344 |
| 41 | 8.746 | 2.855 | 0.126 |
| 42 | 9.198 | 4.912 | 0.216 |
| 43 | 9.474 | 54.914 | 2.420 |
| 44 | 10.085 | 75.423 | 3.324 |
| 45 | 10.541 | 17.521 | 0.772 |
| 46 | 11.218 | 2.340 | 0.103 |

ELS1 A, ELSD Signal

| Peak # | Ret. Time [min] | Area [mV *s] | Area % |
|---|---|---|---|
| 1 | 6.343 | 2796.746 | 47.978 |
| 2 | 6.666 | 30.365 | 0.521 |

Sample-> CPT22010446-19-A

| Peak # | Ret. Time [min] | Area [mV *s] | Area % |
|---|---|---|---|
| 3 | 6.760 | 6.212 | 0.107 |
| 4 | 6.898 | 4.143 | 0.071 |
| 5 | 7.175 | 1981.436 | 33.992 |
| 6 | 9.121 | 430.164 | 7.379 |
| 7 | 9.318 | 124.883 | 2.142 |
| 8 | 9.428 | 101.241 | 1.737 |
| 9 | 9.590 | 183.383 | 3.146 |
| 10 | 9.782 | 57.358 | 0.984 |
| 11 | 9.967 | 50.664 | 0.869 |
| 12 | 10.209 | 62.595 | 1.074 |

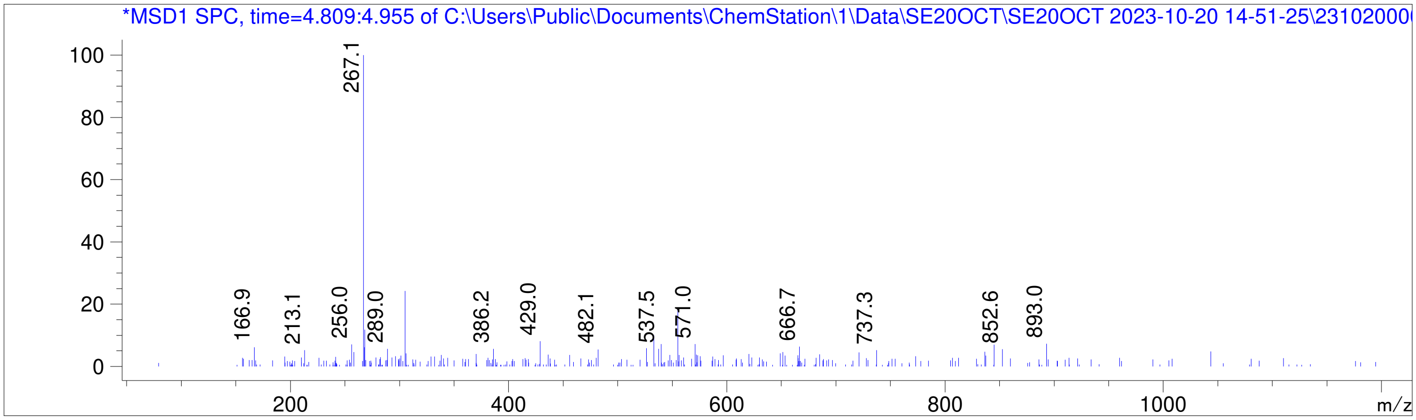
*MSD1 SPC, time=4.809:4.955 of C:\Users\Public\Documents\ChemStation\1\Data\SE20OCT\SE20OCT 2023-10-20 14-51-25\23102000
267.1
166.9
213.1
256.0
289.0
386.2
429.0
482.1
537.5
571.0
666.7
737.3
852.6
893.0
m/z

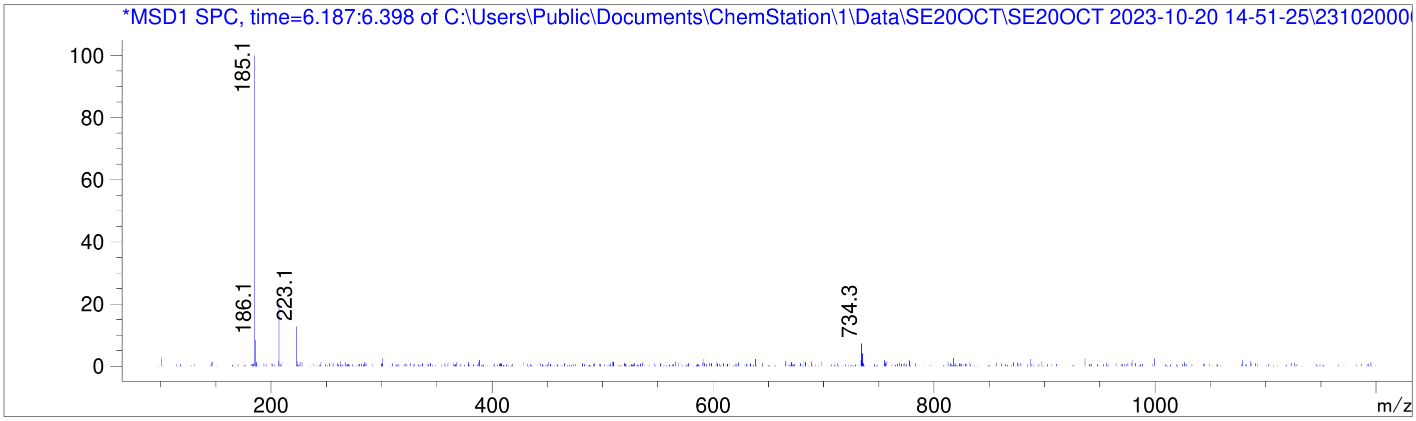
*MSD1 SPC, time=6.187:6.398 of C:\Users\Public\Documents\ChemStation\1\Data\SE20OCT\SE20OCT 2023-10-20 14-51-25\23102000
185.1
186.1
223.1
734.3
m/z

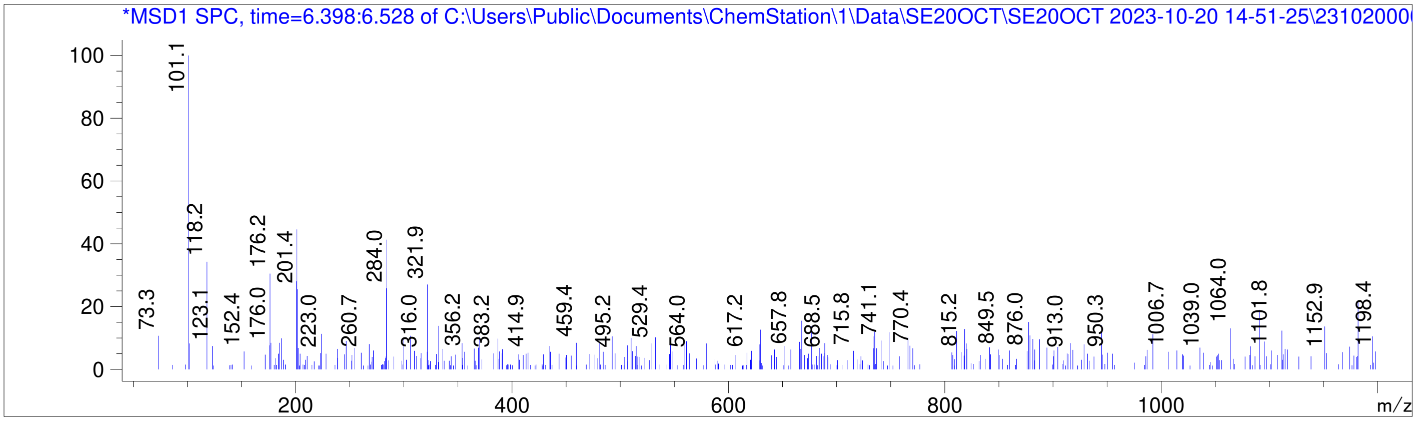
*MSD1 SPC, time=6.398:6.528 of C:\Users\Public\Documents\ChemStation\1\Data\SE20OCT\SE20OCT 2023-10-20 14-51-25\23102000
101.1
73.3
118.2
123.1
152.4
176.0
176.2
201.4
223.0
260.7
284.0
316.0
321.9
356.2
383.2
414.9
459.4
495.2
529.4
564.0
617.2
657.8
688.5
715.8
741.1
770.4
815.2
849.5
876.0
913.0
950.3
1006.7
1039.0
1064.0
1101.8
1152.9
1198.4
m/z

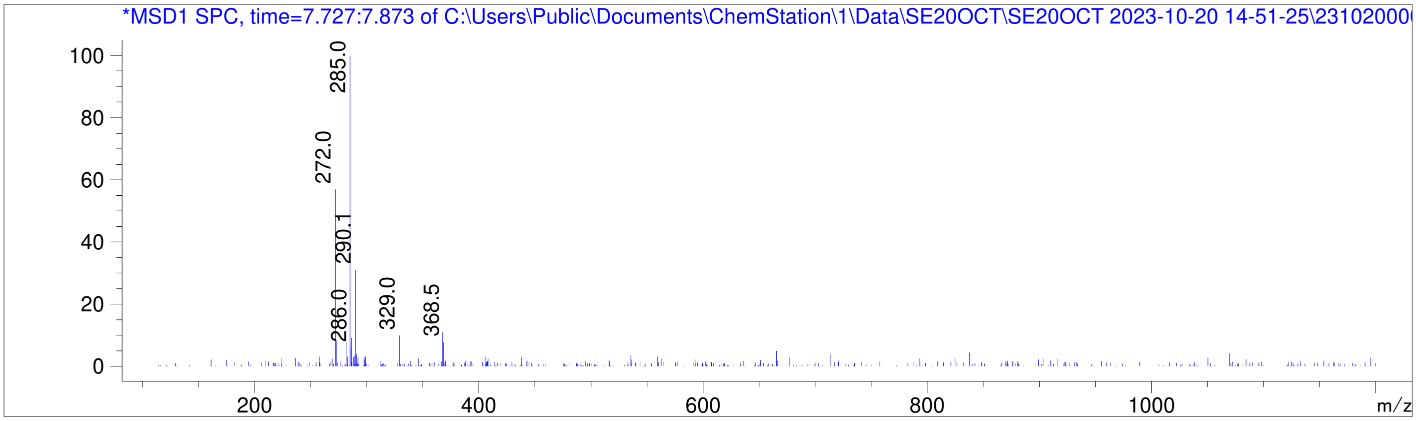
*MSD1 SPC, time=7.727:7.873 of C:\Users\Public\Documents\ChemStation\1\Data\SE20OCT\SE20OCT 2023-10-20 14-51-25\23102000
285.0
272.0
290.1
286.0
329.0
368.5
m/z

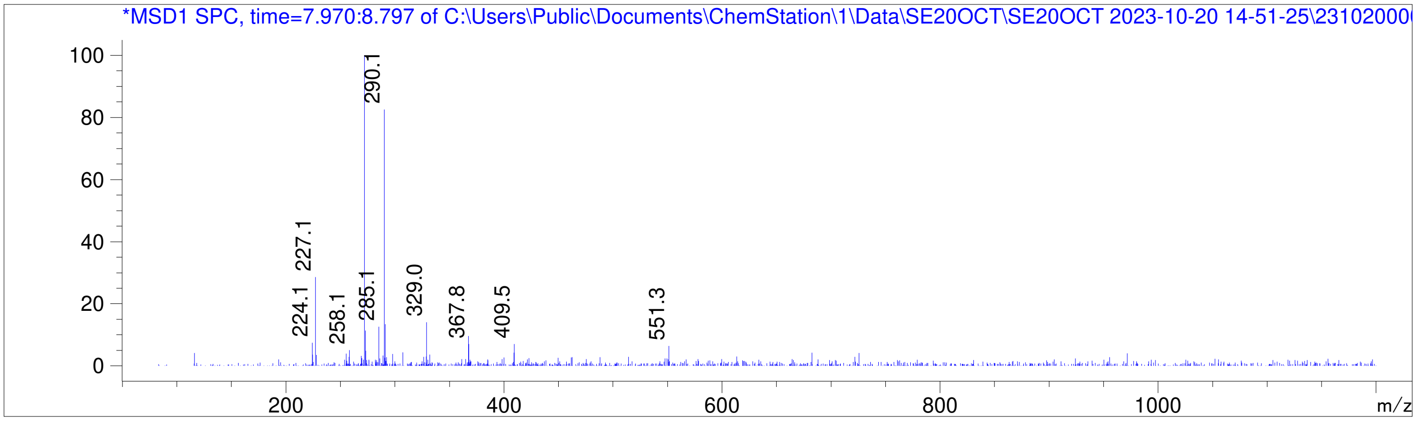
*MSD1 SPC, time=7.970:8.797 of C:\Users\Public\Documents\ChemStation\1\Data\SE20OCT\SE20OCT 2023-10-20 14-51-25\23102000
290.1
227.1
224.1
258.1
285.1
329.0
367.8
409.5
551.3
m/z

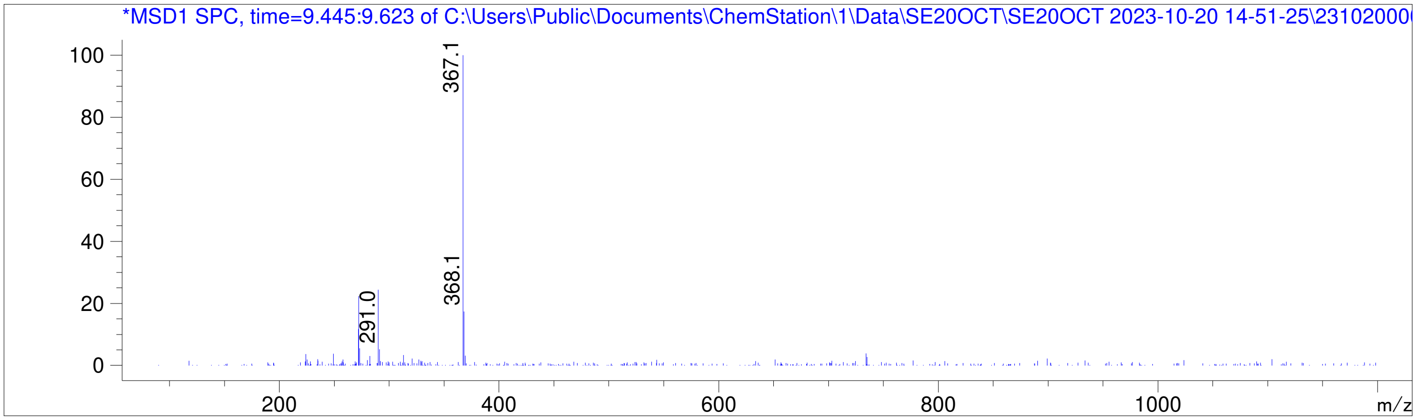
*MSD1 SPC, time=9.445:9.623 of C:\Users\Public\Documents\ChemStation\1\Data\SE20OCT\SE20OCT 2023-10-20 14-51-25\23102000
367.1
368.1
291.0
m/z

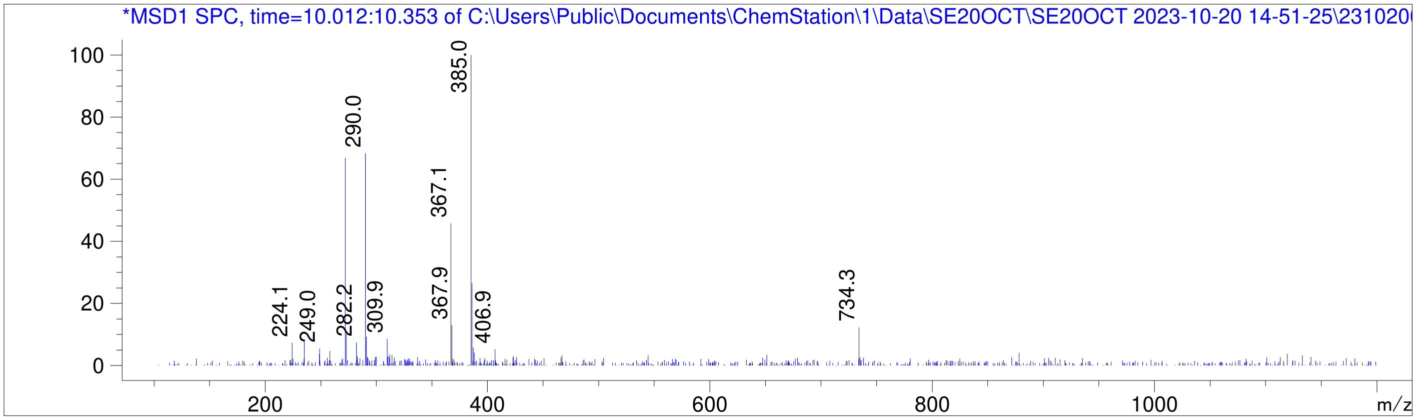
*MSD1 SPC, time=10.012:10.353 of C:\Users\Public\Documents\ChemStation\1\Data\SE20OCT\SE20OCT 2023-10-20 14-51-25\231020
385.0
290.0
367.1
224.1
249.0
282.2
309.9
367.9
406.9
734.3
m/z

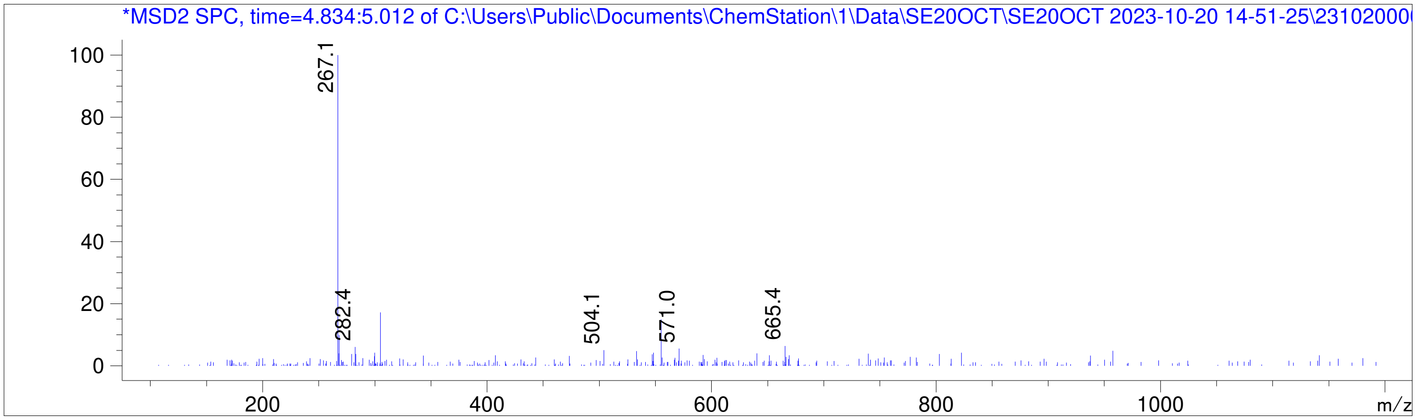
*MSD2 SPC, time=4.834:5.012 of C:\Users\Public\Documents\ChemStation\1\Data\SE20OCT\SE20OCT 2023-10-20 14-51-25\23102000
267.1
282.4
504.1
571.0
665.4
m/z

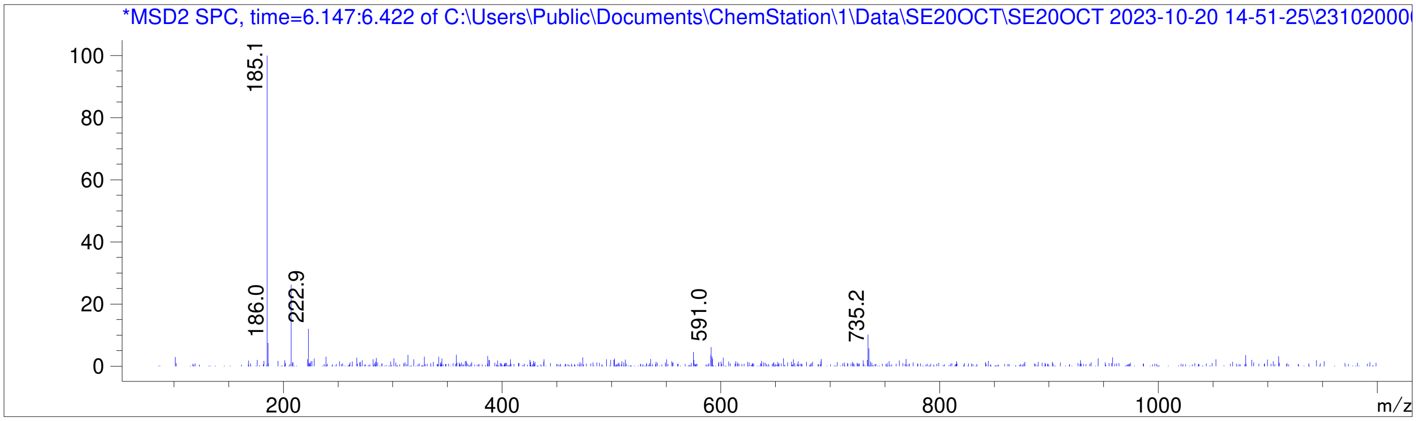
*MSD2 SPC, time=6.147:6.422 of C:\Users\Public\Documents\ChemStation\1\Data\SE20OCT\SE20OCT 2023-10-20 14-51-25\23102000
185.1
186.0
222.9
591.0
735.2
m/z

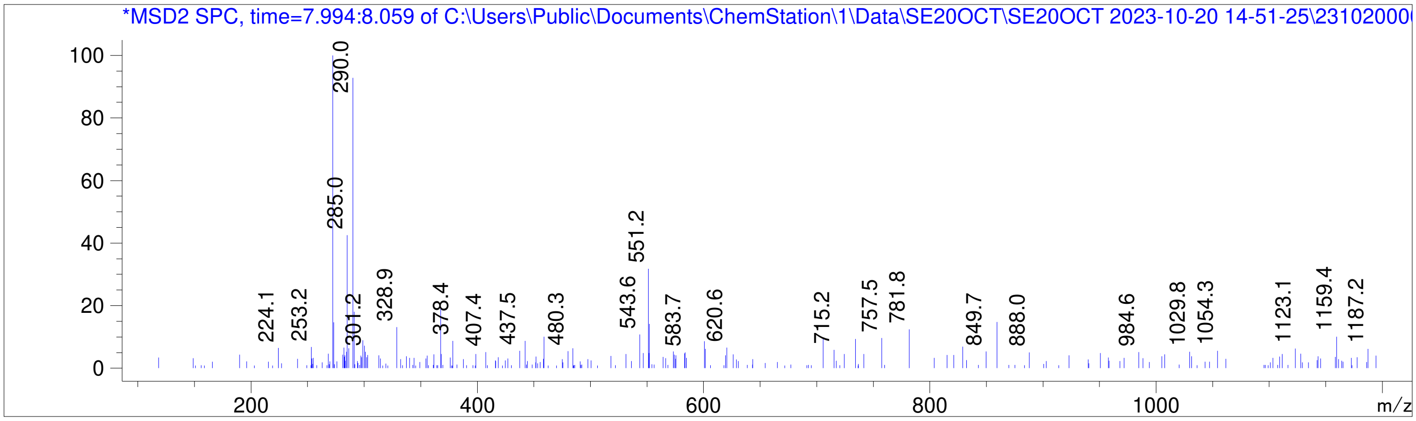
*MSD2 SPC, time=7.994:8.059 of C:\Users\Public\Documents\ChemStation\1\Data\SE20OCT\SE20OCT 2023-10-20 14-51-25\23102000
290.0
285.0
224.1
253.2
301.2
328.9
378.4
407.4
437.5
480.3
543.6
551.2
583.7
620.6
715.2
757.5
781.8
849.7
888.0
984.6
1029.8
1054.3
1123.1
1159.4
1187.2
m/z

Data  -> C:\Users\Public\Documents\ChemStation\1\Data\SE20OCT\SE20OCT 2023-10-20 14-51-->
Sample-> CPT22010446-19-A
==========================================================================

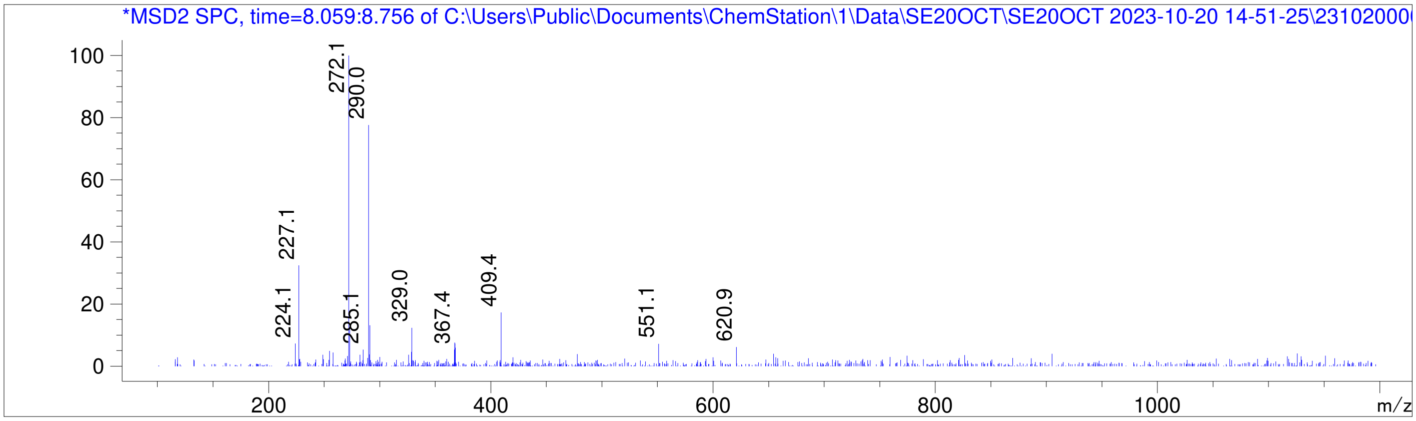


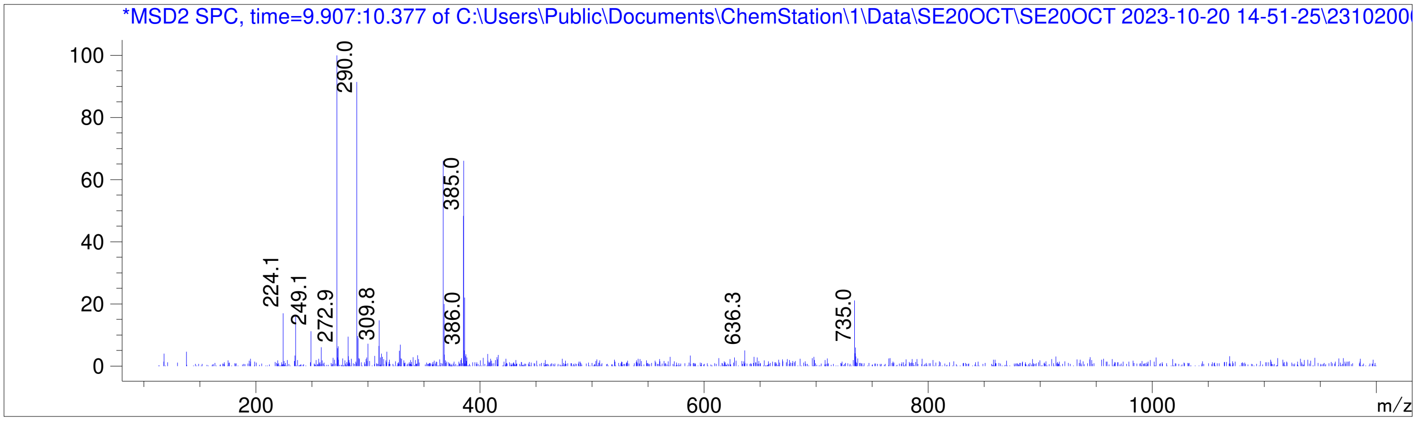

Supplement: Supplementary file 2 — Data S1 and S2 [file sciadv.adr0006_data_s1_and_s2.zip › Supplementary Dataset 1-LCMS DATA/LCMS PNA Hexamers A-T/LCMS T6 50C_80C/80C/24h/CPT22010446-19-A.pdf]
