# Supplementary material for: Astrobiological implications of the stability and reactivity of peptide nucleic acid (PNA) in concentrated sulfuric acid
Source: Sci Adv. 2025 Mar 26;11(13):eadr0006. doi: 10.1126/sciadv.adr0006 (PMC11939054; doi:10.1126/sciadv.adr0006)
Supplement: Supplementary file 2 — Data S1 and S2 [file sciadv.adr0006_data_s1_and_s2.zip › Supplementary Dataset 1-LCMS DATA/LCMS PNA Hexamers A-T/LCMS T6 50C_80C/80C/24h/CPT22010446-19-D2-80deg-24h.pdf]

===

Injection Date : Wed, 8. Nov. 2023

Seq Line : 42
Location : 79
Inj. Vol. : 2 µl

Acq. Method : C:\Users\Public\Documents\ChemStation\1\Data\SE07NOV 2023-11-07 14-56-21\22010446 LCMS-6.M
Analysis Method : C:\Users\Public\Documents\ChemStation\1\Data\SE07NOV 2023-11-07 14-56-21\22010446 LCMS-6.M (Sequence Method)
Waters XBridge Phenyl (4.6 * 150 mm; 3.5 µm); 0.05% TFA (aq) / AcN: 100/0 (0.0 min) --> (6.0 min) --> 70/30 (0.0 min) --> (2.0 min) --> 10/90 (2.0 min); Flow: 1.0 ml/min;
MSD1 = positive; MSD2 = negative

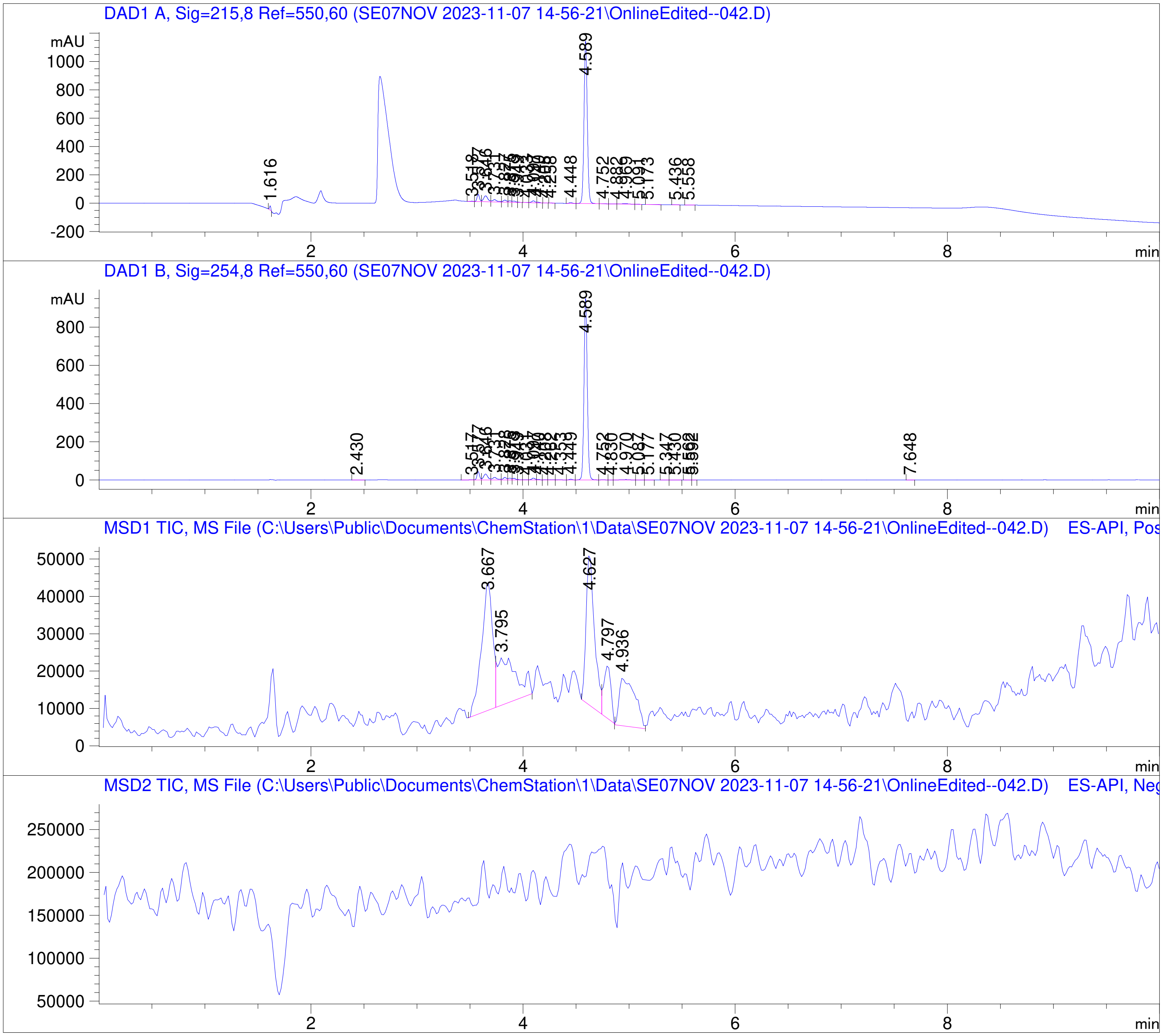

DAD1 A, Sig=215,8 Ref=550,60

Peak #	Ret. Time [min]	Area [mV *s]	Area %
1	1.616	25.090	0.854
2	3.518	3.753	0.128
3	3.577	82.427	2.806
4	3.646	115.379	3.928
5	3.731	50.967	1.735
6	3.827	34.517	1.175
7	3.875	19.706	0.671
8	3.913	20.616	0.702
9	3.949	2.888	0.098
10	4.033	4.418	0.150
11	4.097	31.123	1.060
12	4.140	7.177	0.244
13	4.205	1.424	0.048
14	4.258	1.046	0.036
15	4.448	12.997	0.443
16	4.589	2493.539	84.898
17	4.752	6.539	0.223
18	4.882	2.875	0.098
19	4.969	17.496	0.596
20	5.091	0.529	0.018
21	5.173	0.903	0.031
22	5.436	0.572	0.019
23	5.558	1.122	0.038

DAD1 B, Sig=254,8 Ref=550,60

Peak #	Ret. Time [min]	Area [mV *s]	Area %
1	2.430	0.408	0.017
2	3.517	4.885	0.202
3	3.577	74.747	3.091
4	3.646	88.960	3.679
5	3.731	44.158	1.826
6	3.828	31.673	1.310
7	3.875	19.840	0.820
8	3.913	19.610	0.811
9	3.949	4.253	0.176
10	4.031	5.119	0.212
11	4.097	24.092	0.996
12	4.140	6.611	0.273
13	4.208	2.686	0.111
14	4.262	3.921	0.162
15	4.353	4.280	0.177
16	4.449	8.943	0.370
17	4.589	2053.592	84.919
18	4.752	4.221	0.175
19	4.830	1.101	0.046
20	4.970	12.352	0.511
21	5.087	0.458	0.019
22	5.177	0.443	0.018
23	5.347	0.168	0.007
24	5.430	0.564	0.023
25	5.562	0.815	0.034

Peak #	Ret. Time [min]	Area [mV *s]	Area %
26	5.592	0.243	0.010
27	7.648	0.158	0.007

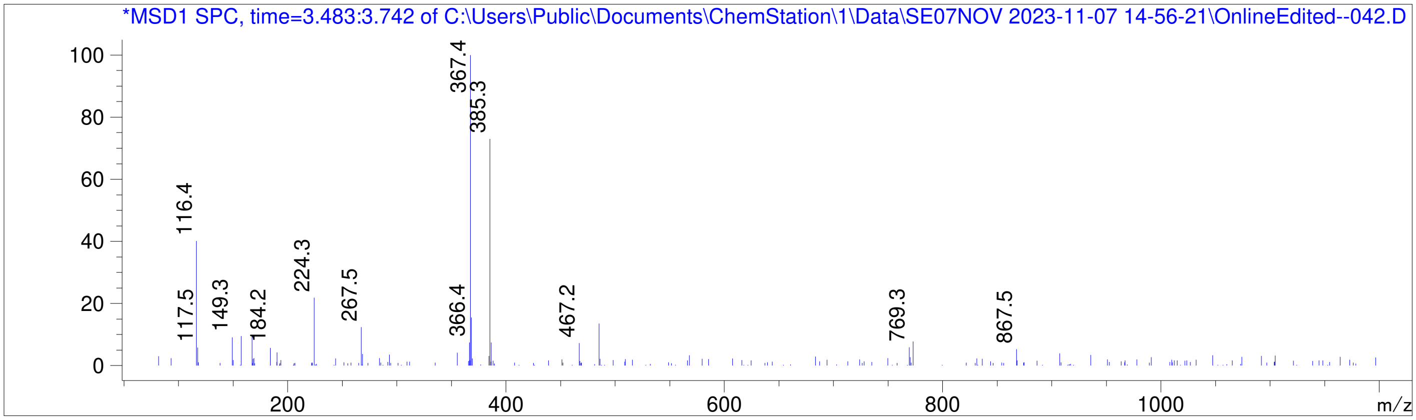
*MSD1 SPC, time=3.483:3.742 of C:\Users\Public\Documents\ChemStation\1\Data\SE07NOV 2023-11-07 14-56-21\OnlineEdited--042.D
100
80
60
40
20
0
116.4
117.5
149.3
184.2
224.3
267.5
366.4
367.4
385.3
467.2
769.3
867.5
200
400
600
800
1000
m/z

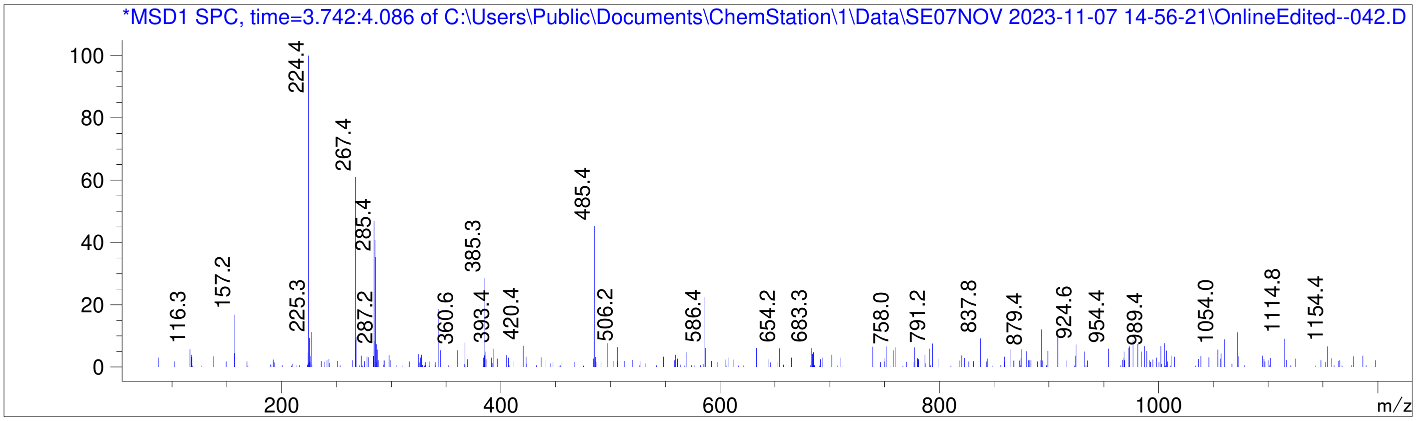
*MSD1 SPC, time=3.742:4.086 of C:\Users\Public\Documents\ChemStation\1\Data\SE07NOV 2023-11-07 14-56-21\OnlineEdited--042.D
100
80
60
40
20
0
116.3
157.2
224.4
225.3
267.4
285.4
287.2
360.6
385.3
393.4
420.4
485.4
506.2
586.4
654.2
683.3
758.0
791.2
837.8
879.4
924.6
954.4
989.4
1054.0
1114.8
1154.4
200
400
600
800
1000
m/z

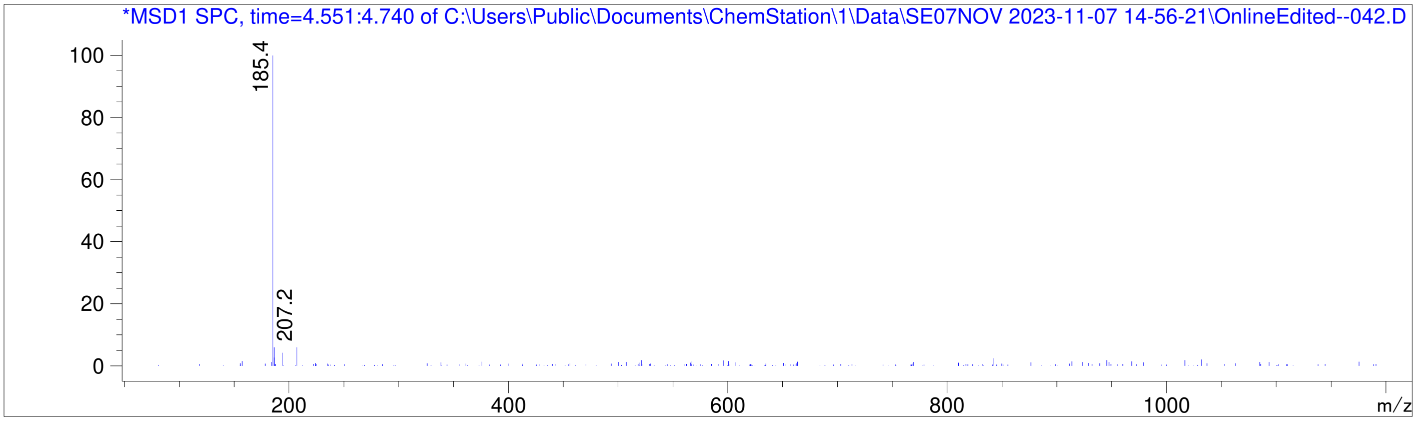
*MSD1 SPC, time=4.551:4.740 of C:\Users\Public\Documents\ChemStation\1\Data\SE07NOV 2023-11-07 14-56-21\OnlineEdited--042.D
100
80
60
40
20
0
185.4
207.2
200
400
600
800
1000
m/z

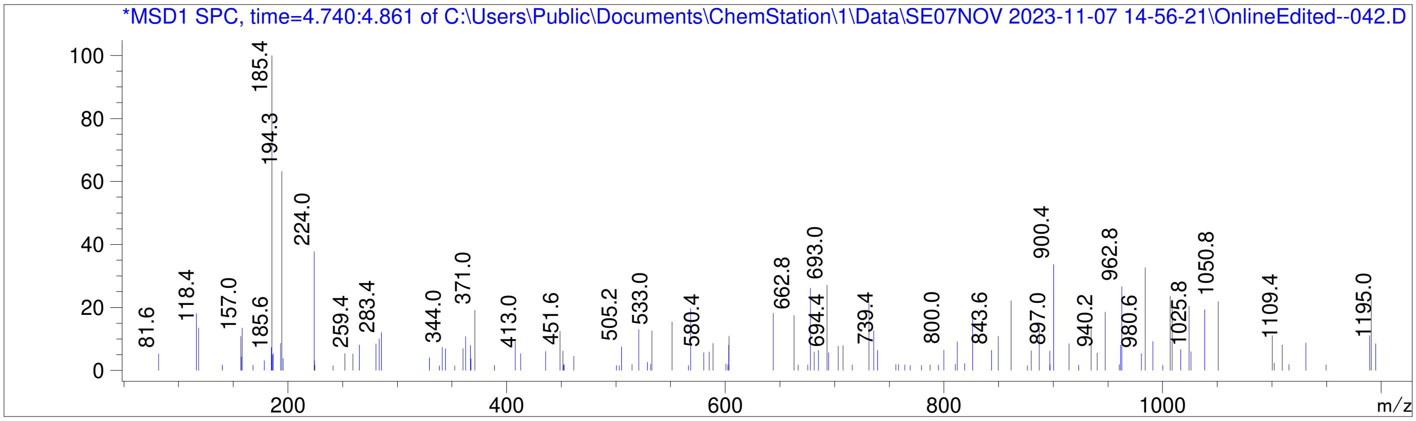
*MSD1 SPC, time=4.740:4.861 of C:\Users\Public\Documents\ChemStation\1\Data\SE07NOV 2023-11-07 14-56-21\OnlineEdited--042.D
185.4
194.3
224.0
118.4
157.0
185.6
81.6
259.4
283.4
344.0
371.0
413.0
451.6
505.2
533.0
580.4
662.8
693.0
694.4
739.4
800.0
843.6
900.4
897.0
940.2
962.8
980.6
1025.8
1050.8
1109.4
1195.0
m/z

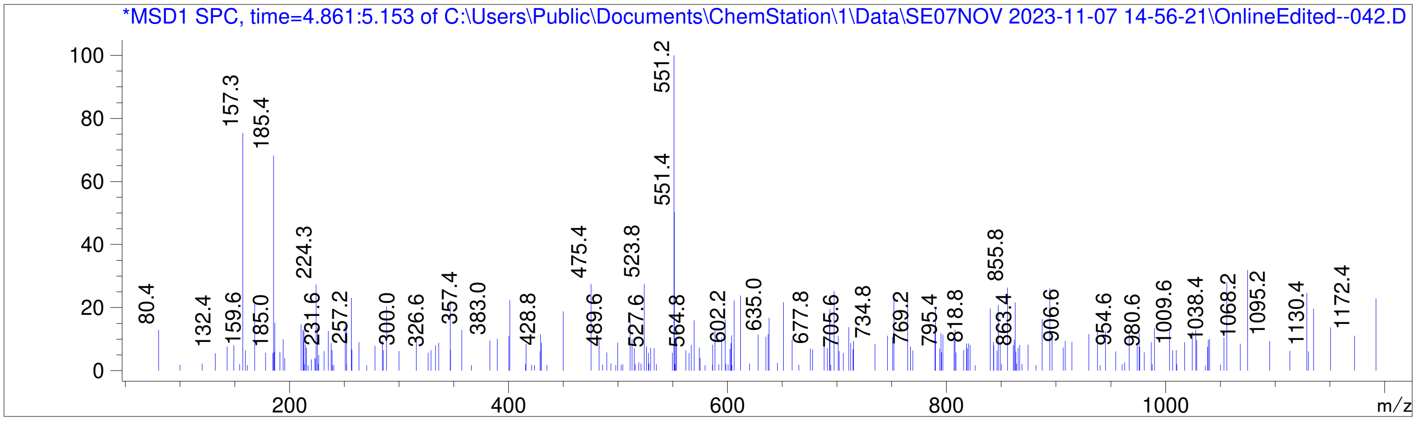
*MSD1 SPC, time=4.861:5.153 of C:\Users\Public\Documents\ChemStation\1\Data\SE07NOV 2023-11-07 14-56-21\OnlineEdited--042.D
551.2
157.3
185.4
551.4
224.3
475.4
523.8
855.8
80.4
132.4
159.6
185.0
231.6
257.2
300.0
326.6
357.4
383.0
428.8
489.6
527.6
564.8
602.2
635.0
677.8
705.6
734.8
769.2
795.4
818.8
863.4
906.6
954.6
980.6
1009.6
1038.4
1068.2
1095.2
1130.4
1172.4
m/z
