# Supplementary material for: Astrobiological implications of the stability and reactivity of peptide nucleic acid (PNA) in concentrated sulfuric acid
Source: Sci Adv. 2025 Mar 26;11(13):eadr0006. doi: 10.1126/sciadv.adr0006 (PMC11939054; doi:10.1126/sciadv.adr0006)

Data -> C:\Users\Public\Documents\ChemStation\1\Data\SE26OCT 2023-10-26 13-10-30\ ->
Sample-> CPT22010446-19-D1-14d

==

Injection Date	:	Thu, 26. Oct. 2023	Seq Line	:	3
			Location	:	12
			Inj. Vol.	:	2 µl

Acq. Method : C:\Users\Public\Documents\ChemStation\1\Data\SE26OCT 2023-10-26 13-10-30\22010446C LCMS-6#.M

Analysis Method : C:\Users\Public\Documents\ChemStation\1\Data\SE26OCT 2023-10-26 13-10-30\22010446C LCMS-6#.M (Sequence Method)

Waters XBridge BEH Amide (4.6 x 150 mm, 2.5 µm); PN# 186006726

Mobile Phase A: 20mM Ammonium Acetate (aq) pH 8.2

Mobile Phase B: AcN

Mobile Phase A / Mobile Phase B: 5/95 (0 min) --> (10 min) --> 60/40 (5 min); Flow: 1.0 ml/min; MSD1 = positive; MSD2 = negative

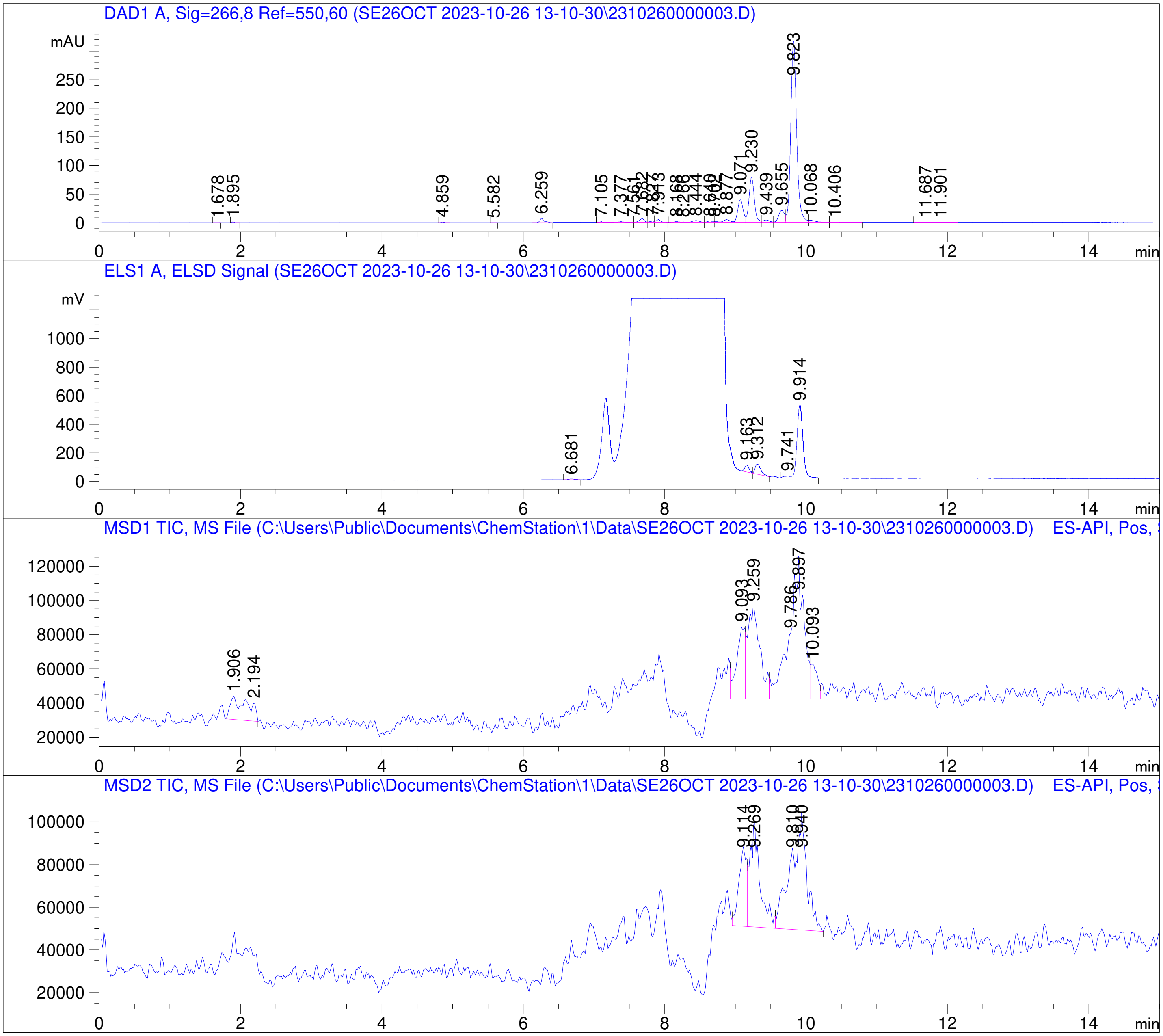

DAD1 A, Sig=266,8 Ref=550,60

Peak #	Ret. Time [min]	Area [mV *s]	Area %
1	1.678	0.468	0.017
2	1.895	2.709	0.096
3	4.859	3.331	0.118
4	5.582	0.426	0.015
5	6.259	28.473	1.009
6	7.105	3.310	0.117
7	7.377	7.511	0.266
8	7.561	0.786	0.028
9	7.682	30.630	1.086
10	7.827	8.925	0.316
11	7.913	20.532	0.728
12	8.168	6.205	0.220
13	8.266	2.602	0.092
14	8.444	21.569	0.765
15	8.640	12.563	0.445
16	8.702	5.428	0.192
17	8.877	29.301	1.039
18	9.071	208.564	7.393
19	9.230	414.372	14.688
20	9.439	24.911	0.883
21	9.655	123.362	4.373
22	9.823	1833.943	65.007
23	10.068	25.547	0.906
24	10.406	4.074	0.144
25	11.687	0.715	0.025
26	11.901	0.876	0.031

ELS1 A, ELSD Signal

Peak #	Ret. Time [min]	Area [mV *s]	Area %
1	6.681	32.808	0.896
2	9.163	201.214	5.497
3	9.312	383.318	10.471
4	9.741	83.178	2.272
5	9.914	2960.230	80.864

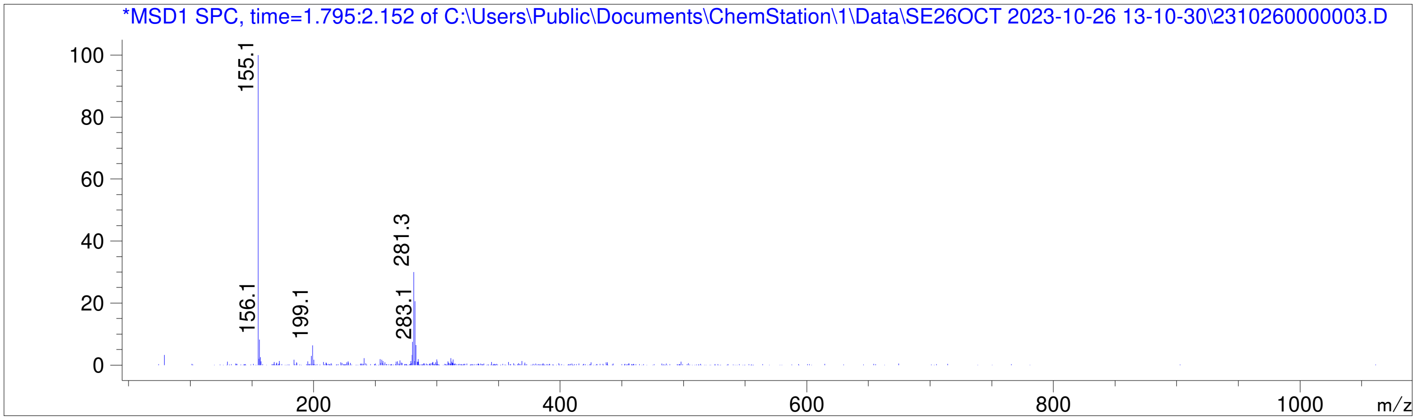
*MSD1 SPC, time=1.795:2.152 of C:\Users\Public\Documents\ChemStation\1\Data\SE26OCT 2023-10-26 13-10-30\2310260000003.D
155.1
156.1
199.1
281.3
283.1
m/z

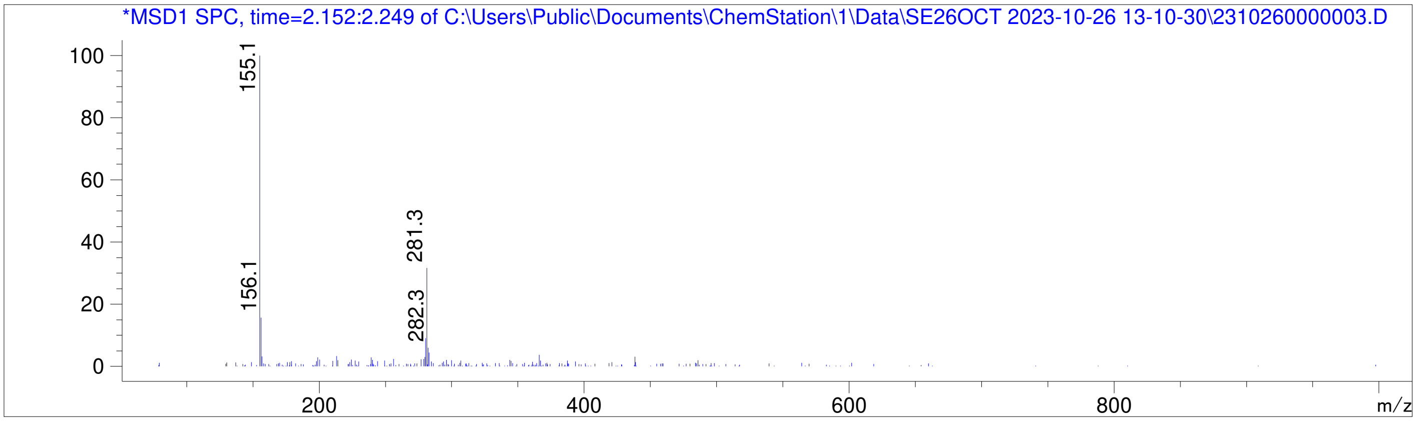
*MSD1 SPC, time=2.152:2.249 of C:\Users\Public\Documents\ChemStation\1\Data\SE26OCT 2023-10-26 13-10-30\2310260000003.D
155.1
156.1
281.3
282.3
m/z

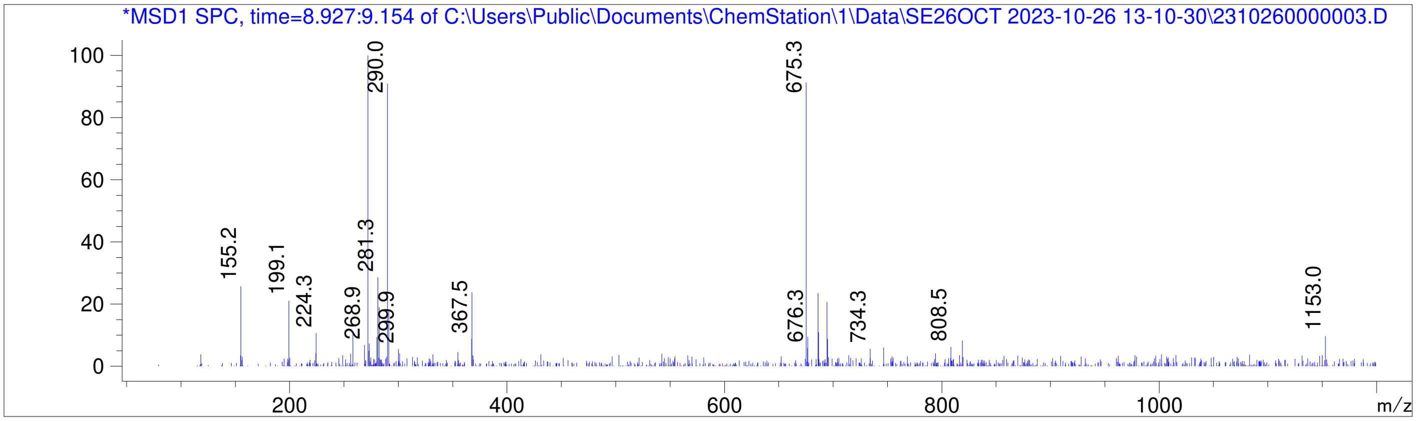
*MSD1 SPC, time=8.927:9.154 of C:\Users\Public\Documents\ChemStation\1\Data\SE26OCT 2023-10-26 13-10-30\2310260000003.D
155.2
199.1
224.3
268.9
281.3
290.0
299.9
367.5
675.3
676.3
734.3
808.5
1153.0
m/z

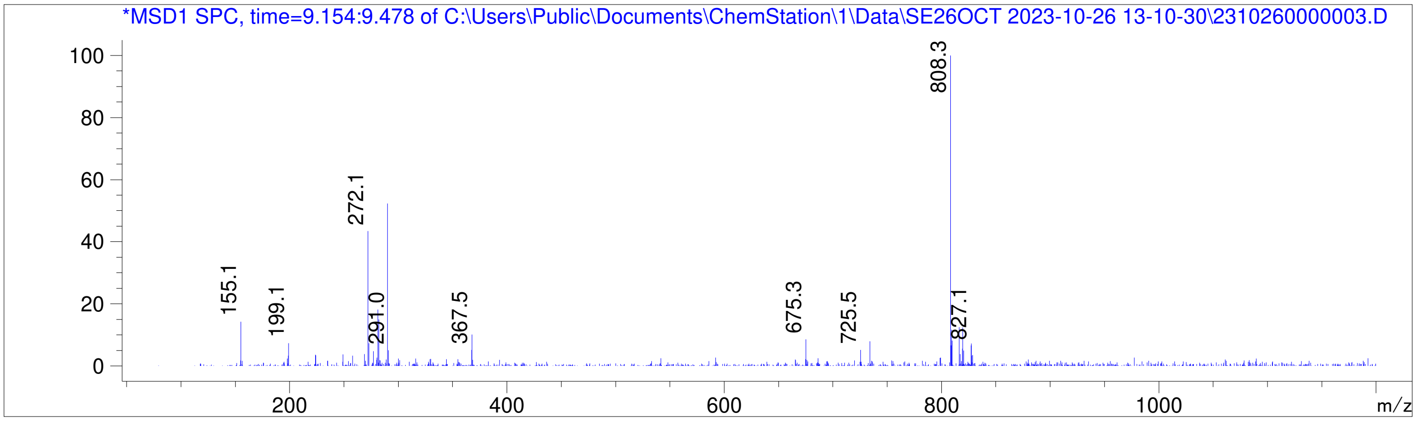
*MSD1 SPC, time=9.154:9.478 of C:\Users\Public\Documents\ChemStation\1\Data\SE26OCT 2023-10-26 13-10-30\2310260000003.D
808.3
272.1
155.1
199.1
291.0
367.5
675.3
725.5
827.1
m/z

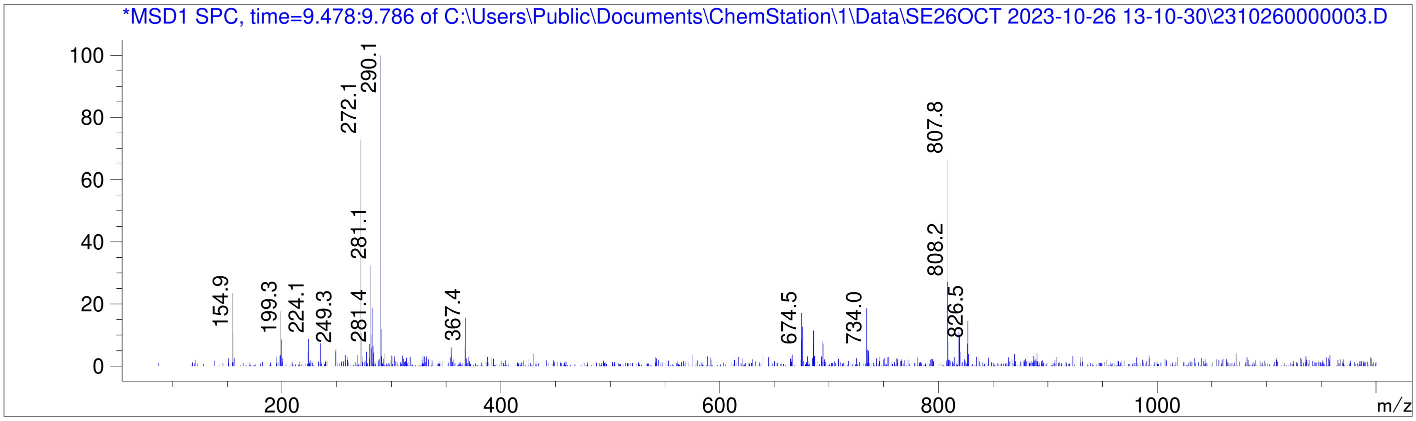
*MSD1 SPC, time=9.478:9.786 of C:\Users\Public\Documents\ChemStation\1\Data\SE26OCT 2023-10-26 13-10-30\2310260000003.D
290.1
272.1
807.8
281.1
808.2
154.9
199.3
224.1
249.3
281.4
367.4
674.5
734.0
826.5
m/z

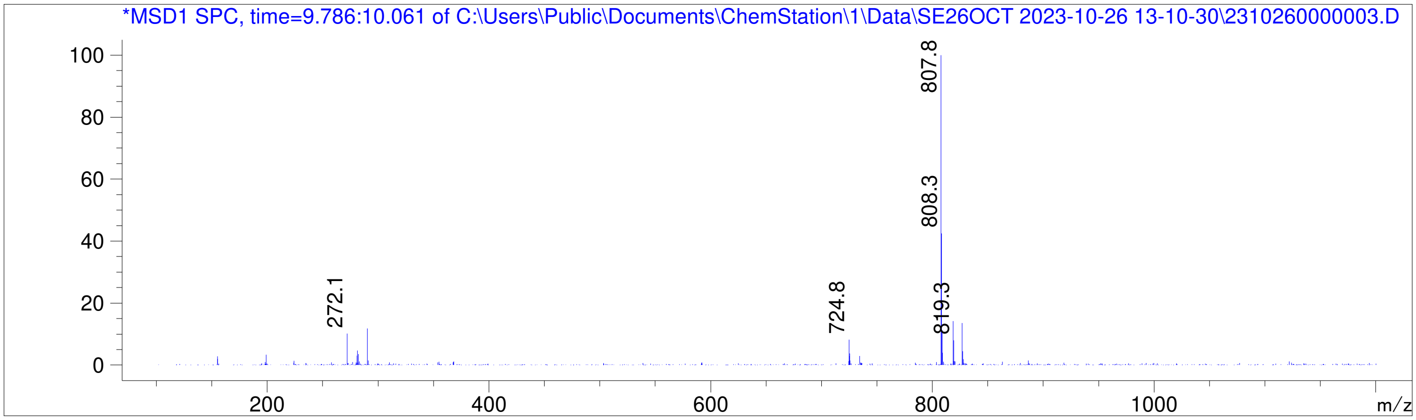
*MSD1 SPC, time=9.786:10.061 of C:\Users\Public\Documents\ChemStation\1\Data\SE26OCT 2023-10-26 13-10-30\2310260000003.D
807.8
808.3
272.1
724.8
819.3
m/z

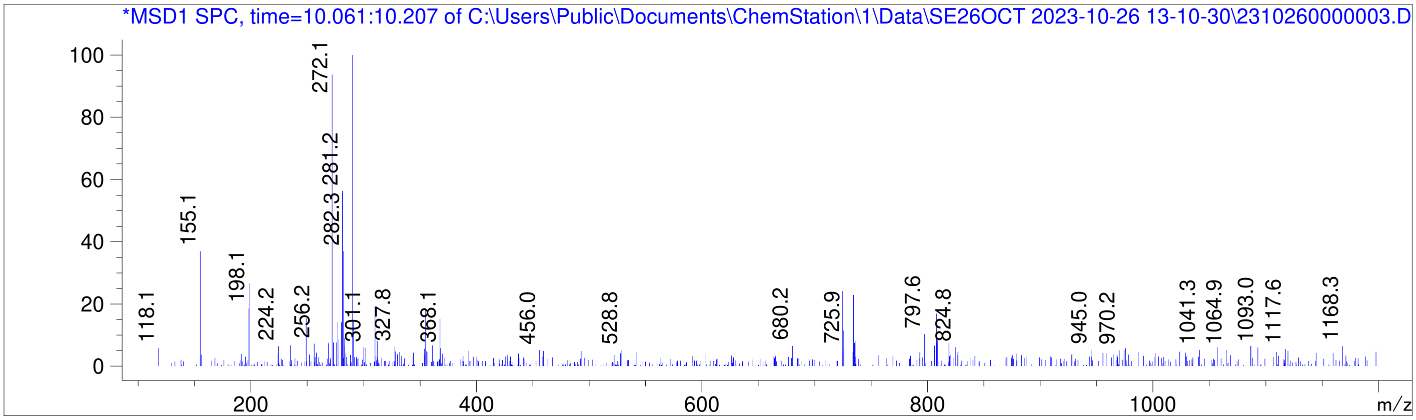
*MSD1 SPC, time=10.061:10.207 of C:\Users\Public\Documents\ChemStation\1\Data\SE26OCT 2023-10-26 13-10-30\2310260000003.D
272.1
282.3
281.2
155.1
198.1
118.1
224.2
256.2
301.1
327.8
368.1
456.0
528.8
680.2
725.9
797.6
824.8
945.0
970.2
1041.3
1064.9
1093.0
1117.6
1168.3
m/z

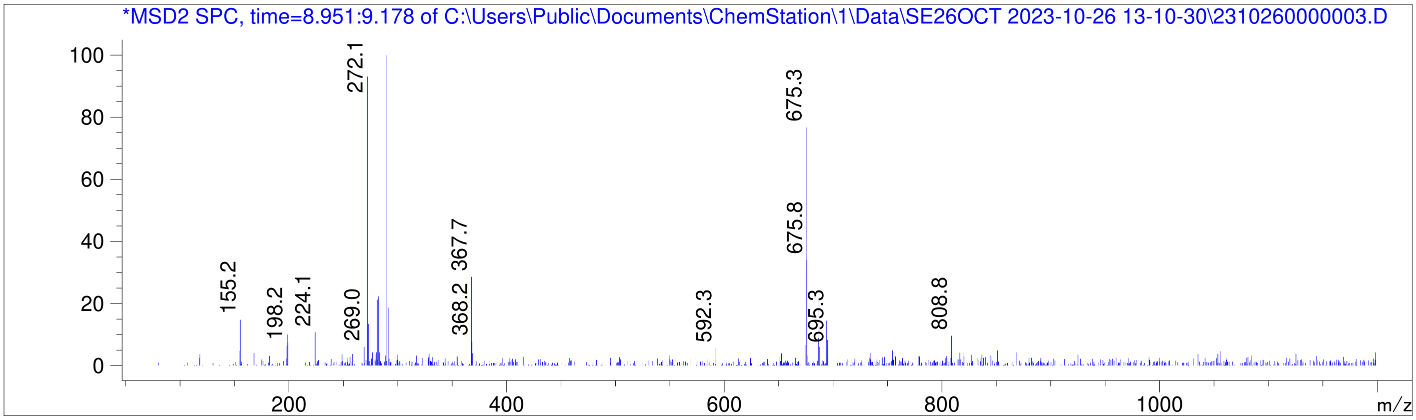
*MSD2 SPC, time=8.951:9.178 of C:\Users\Public\Documents\ChemStation\1\Data\SE26OCT 2023-10-26 13-10-30\2310260000003.D
155.2
198.2
224.1
269.0
272.1
367.7
368.2
592.3
675.3
675.8
695.3
808.8
m/z

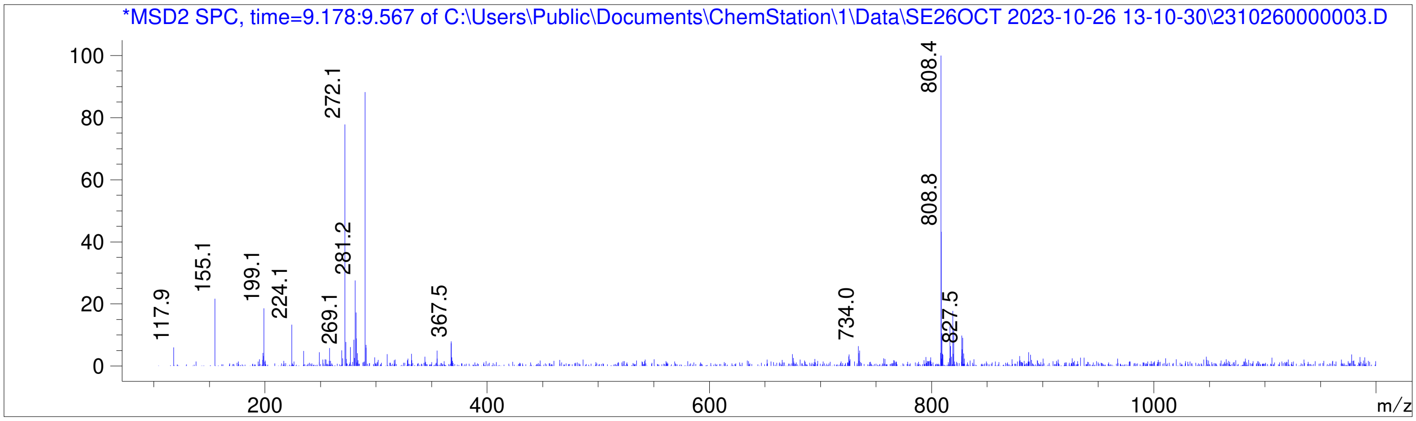
*MSD2 SPC, time=9.178:9.567 of C:\Users\Public\Documents\ChemStation\1\Data\SE26OCT 2023-10-26 13-10-30\2310260000003.D
117.9
155.1
199.1
224.1
269.1
272.1
281.2
367.5
734.0
808.4
808.8
827.5
m/z

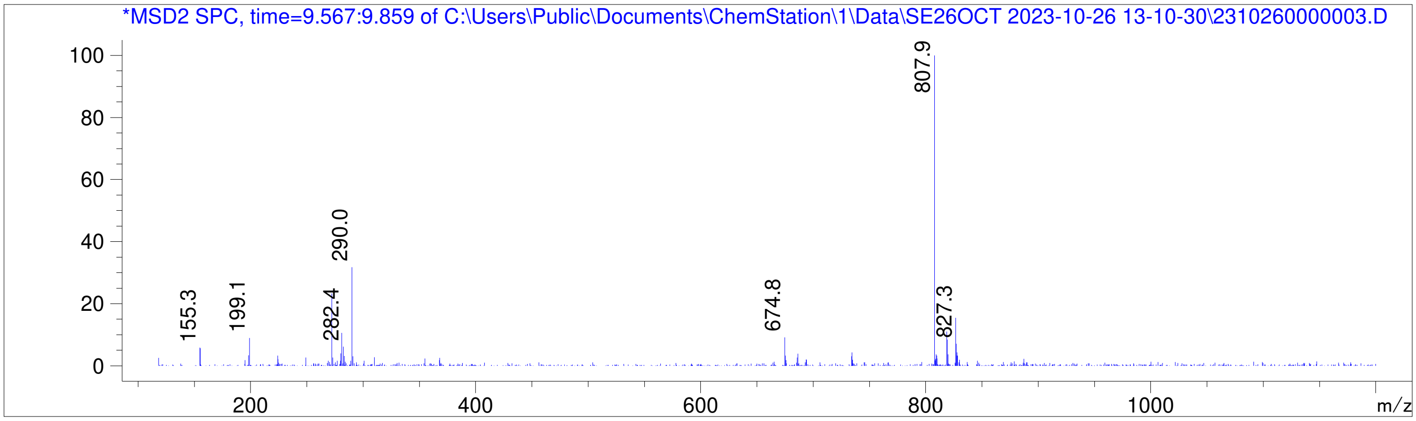
*MSD2 SPC, time=9.567:9.859 of C:\Users\Public\Documents\ChemStation\1\Data\SE26OCT 2023-10-26 13-10-30\2310260000003.D
155.3
199.1
282.4
290.0
674.8
807.9
827.3
m/z

Sample-> CPT22010446-19-D1-14d

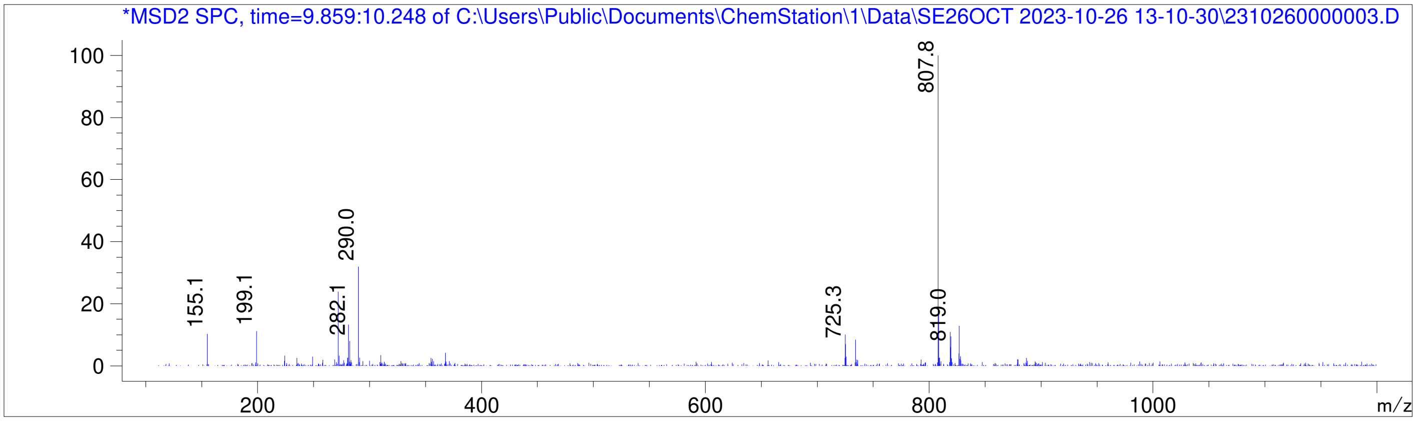

Supplement: Supplementary file 2 — Data S1 and S2 [file sciadv.adr0006_data_s1_and_s2.zip › Supplementary Dataset 1-LCMS DATA/LCMS PNA Hexamers A-T/LCMS T6 RT/14d/CPT22010446-19-D1-14d.pdf]
