# Supplementary material for: Astrobiological implications of the stability and reactivity of peptide nucleic acid (PNA) in concentrated sulfuric acid
Source: Sci Adv. 2025 Mar 26;11(13):eadr0006. doi: 10.1126/sciadv.adr0006 (PMC11939054; doi:10.1126/sciadv.adr0006)
Supplement: Supplementary file 2 — Data S1 and S2 [file sciadv.adr0006_data_s1_and_s2.zip › Supplementary Dataset 1-LCMS DATA/LCMS PNA Hexamers A-T/LCMS T6 RT/14d/CPT22010446-19-D2-14d.pdf]

Data -> C:\Users\Public\Documents\ChemStation\1\Data\SE26OCT 2023-10-26 13-10-30\ ->
Sample-> CPT22010446-19-D2-14d

==

Injection Date	:	Thu, 26. Oct. 2023	Seq Line	:	5
			Location	:	14
			Inj. Vol.	:	2 µl

Acq. Method : C:\Users\Public\Documents\ChemStation\1\Data\SE26OCT 2023-10-26 13-10-30\22010446C LCMS-6#.M

Analysis Method : C:\Users\Public\Documents\ChemStation\1\Data\SE26OCT 2023-10-26 13-10-30\22010446C LCMS-6#.M (Sequence Method)

Waters XBridge BEH Amide (4.6 x 150 mm, 2.5 µm); PN# 186006726

Mobile Phase A: 20mM Ammonium Acetate (aq) pH 8.2

Mobile Phase B: AcN

Mobile Phase A / Mobile Phase B: 5/95 (0 min) --> (10 min) --> 60/40 (5 min); Flow: 1.0 ml/min; MSD1 = positive; MSD2 = negative

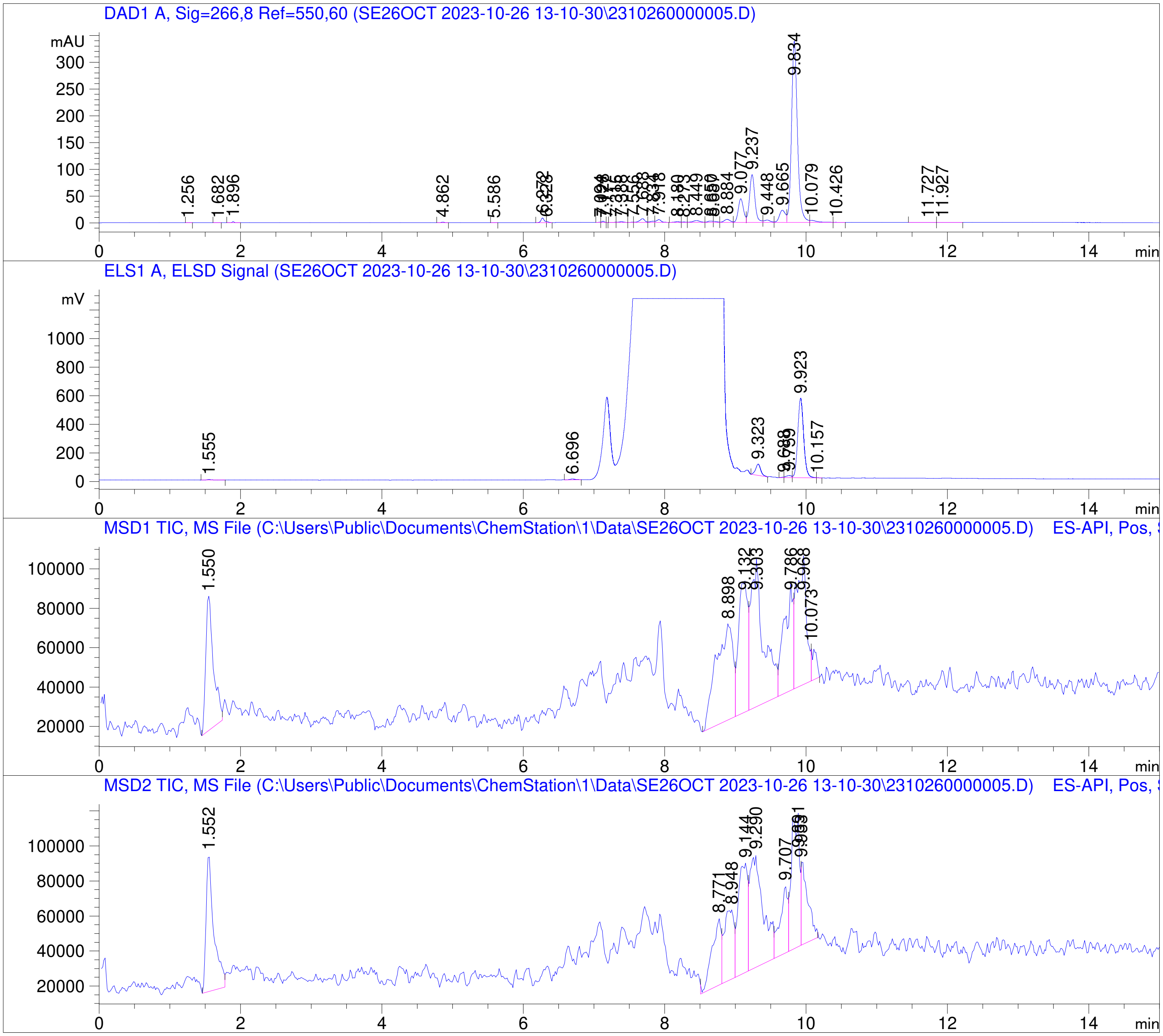

DAD1 A, Sig=266,8 Ref=550,60

Peak #	Ret. Time [min]	Area [mV *s]	Area %
1	1.256	0.219	0.007
2	1.682	0.525	0.017
3	1.896	4.269	0.140
4	4.862	3.535	0.116
5	5.586	0.434	0.014
6	6.272	27.358	0.896
7	6.328	3.409	0.112
8	7.094	0.829	0.027
9	7.128	5.402	0.177
10	7.177	0.200	0.007
11	7.315	1.245	0.041
12	7.388	6.748	0.221
13	7.556	0.768	0.025
14	7.688	33.861	1.110
15	7.834	9.220	0.302
16	7.918	23.081	0.756
17	8.180	6.311	0.207
18	8.273	2.904	0.095
19	8.449	22.510	0.738
20	8.650	11.384	0.373
21	8.687	6.705	0.220
22	8.884	31.790	1.042
23	9.077	226.524	7.423
24	9.237	467.567	15.321
25	9.448	25.220	0.826
26	9.665	132.739	4.349
27	9.834	1970.287	64.560
28	10.079	23.776	0.779
29	10.426	0.248	0.008
30	11.727	1.487	0.049
31	11.927	1.300	0.043

ELS1 A, ELSD Signal

Peak #	Ret. Time [min]	Area [mV *s]	Area %
1	1.555	26.899	0.708
2	6.696	35.056	0.922
3	9.323	394.975	10.390
4	9.688	11.231	0.295
5	9.759	82.090	2.159
6	9.923	3245.457	85.371
7	10.157	5.872	0.154

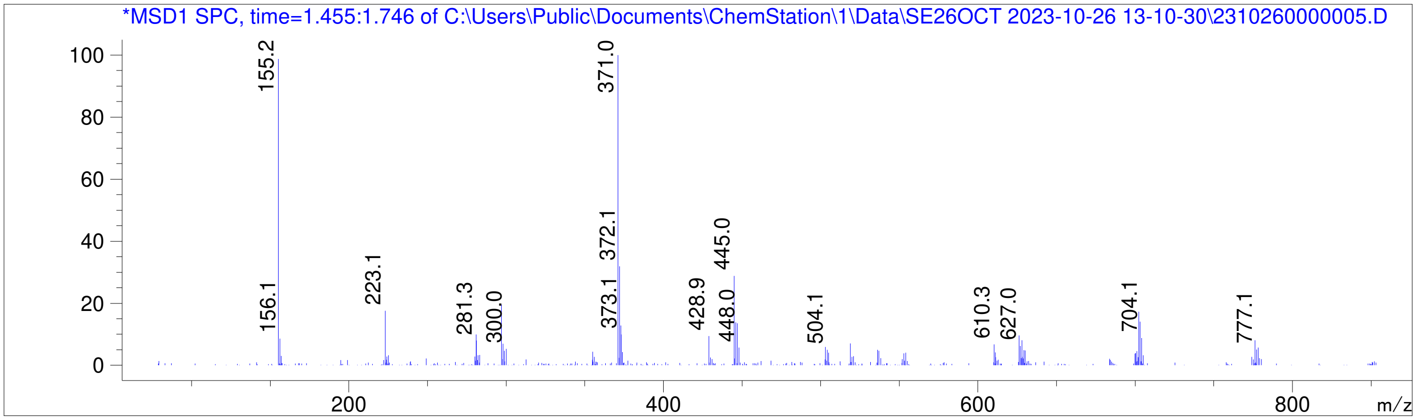
*MSD1 SPC, time=1.455:1.746 of C:\Users\Public\Documents\ChemStation\1\Data\SE26OCT 2023-10-26 13-10-30\2310260000005.D
155.2
371.0
372.1
445.0
223.1
428.9
156.1
281.3
300.0
373.1
448.0
504.1
610.3
627.0
704.1
777.1
m/z

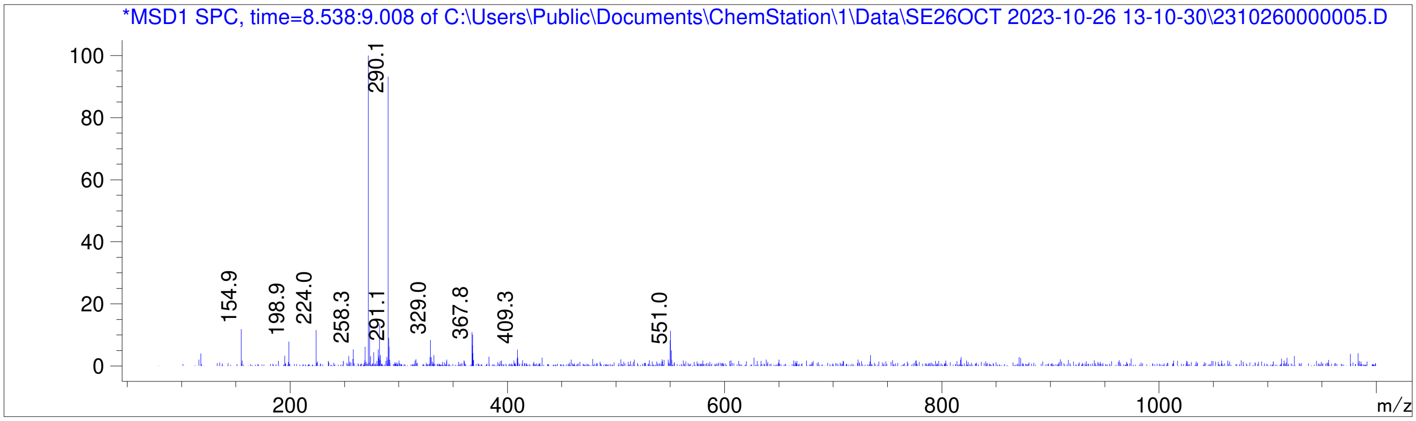
*MSD1 SPC, time=8.538:9.008 of C:\Users\Public\Documents\ChemStation\1\Data\SE26OCT 2023-10-26 13-10-30\2310260000005.D
290.1
154.9
198.9
224.0
258.3
291.1
329.0
367.8
409.3
551.0
m/z

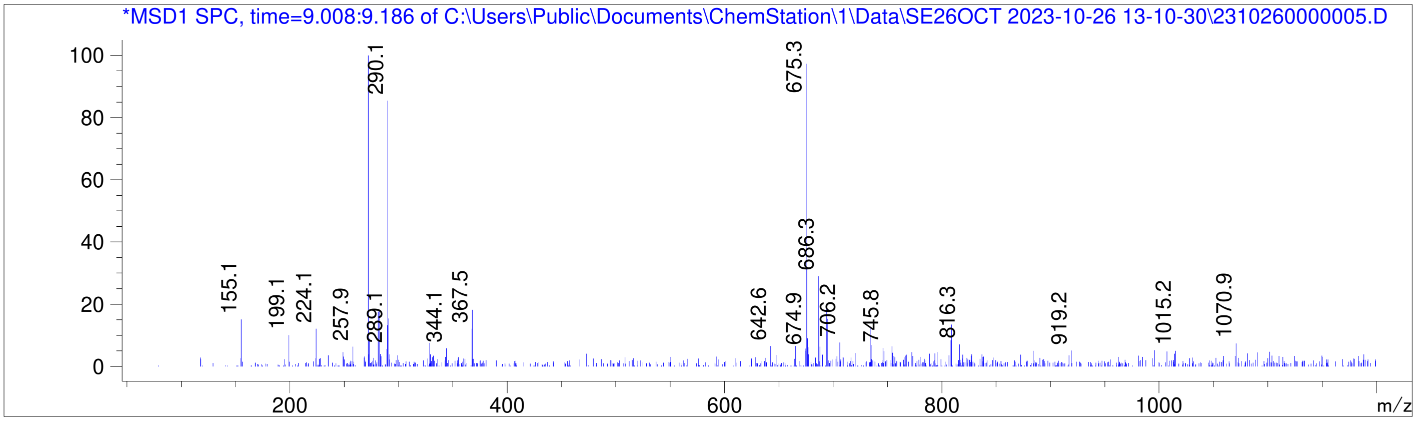
*MSD1 SPC, time=9.008:9.186 of C:\Users\Public\Documents\ChemStation\1\Data\SE26OCT 2023-10-26 13-10-30\2310260000005.D
290.1
675.3
686.3
155.1
199.1
224.1
257.9
289.1
344.1
367.5
642.6
674.9
706.2
745.8
816.3
919.2
1015.2
1070.9
m/z

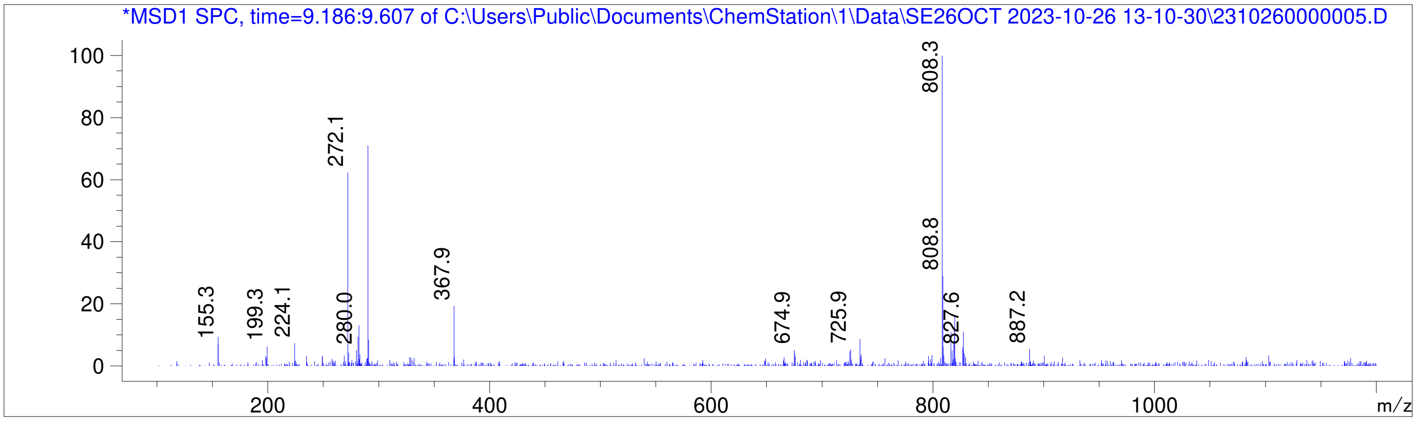
*MSD1 SPC, time=9.186:9.607 of C:\Users\Public\Documents\ChemStation\1\Data\SE26OCT 2023-10-26 13-10-30\2310260000005.D
100
80
60
40
20
0
808.3
272.1
808.8
367.9
155.3
199.3
224.1
280.0
674.9
725.9
827.6
887.2
200
400
600
800
1000
m/z

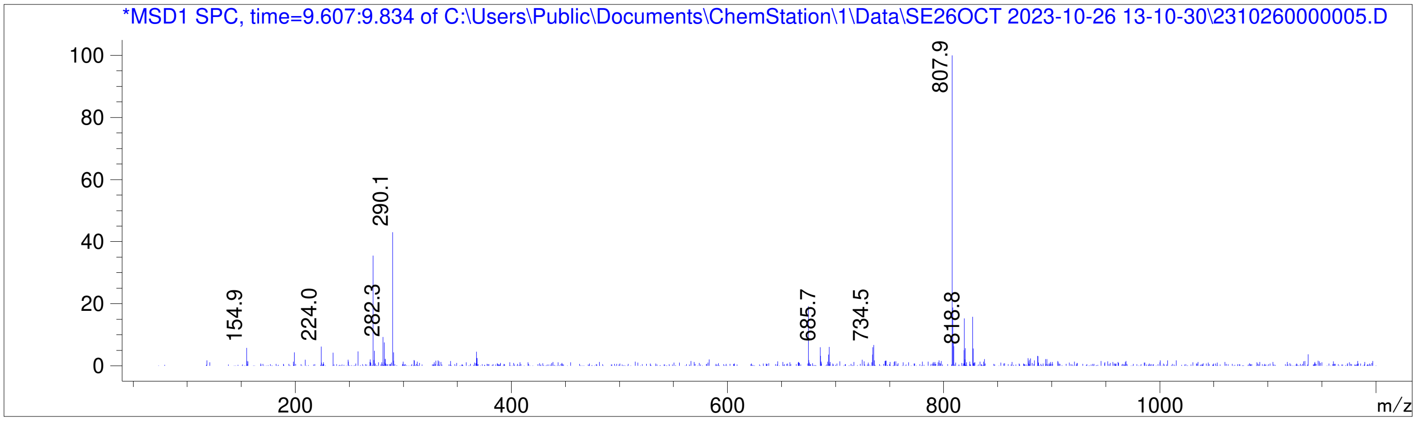
*MSD1 SPC, time=9.607:9.834 of C:\Users\Public\Documents\ChemStation\1\Data\SE26OCT 2023-10-26 13-10-30\2310260000005.D
100
80
60
40
20
0
807.9
290.1
154.9
224.0
282.3
685.7
734.5
818.8
200
400
600
800
1000
m/z

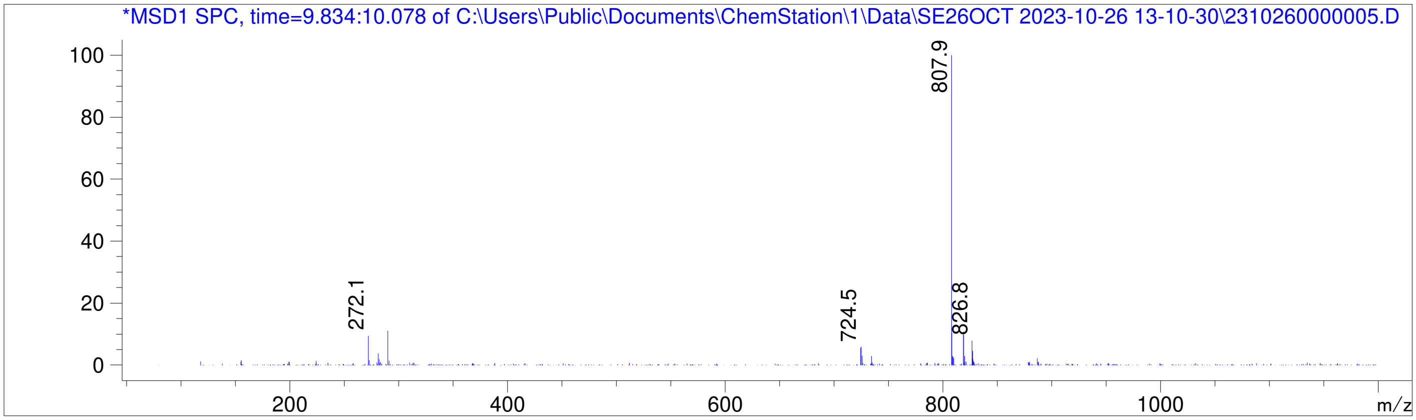
*MSD1 SPC, time=9.834:10.078 of C:\Users\Public\Documents\ChemStation\1\Data\SE26OCT 2023-10-26 13-10-30\2310260000005.D
100
80
60
40
20
0
807.9
272.1
724.5
826.8
200
400
600
800
1000
m/z

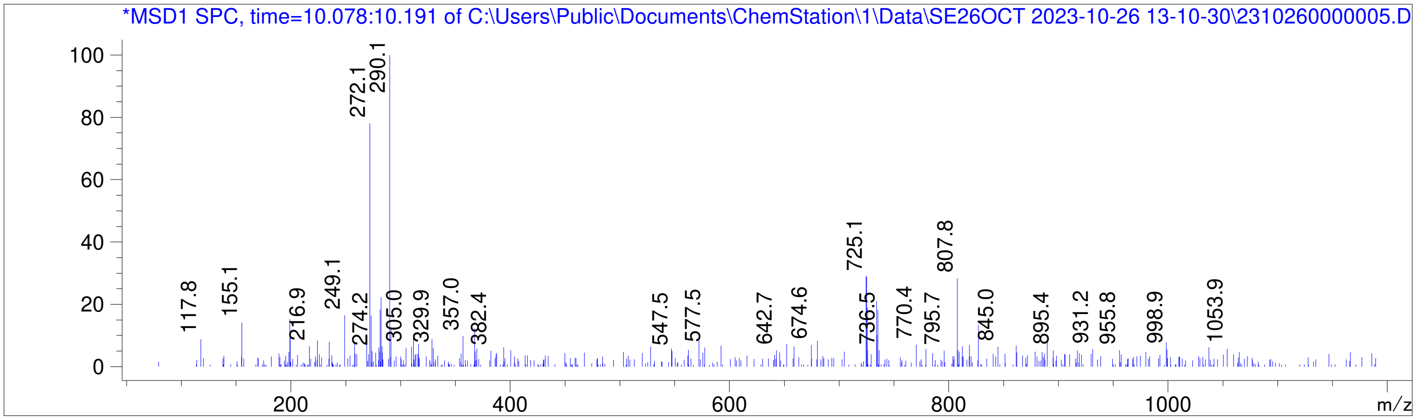
*MSD1 SPC, time=10.078:10.191 of C:\Users\Public\Documents\ChemStation\1\Data\SE26OCT 2023-10-26 13-10-30\2310260000005.D
100
80
60
40
20
0
272.1
290.1
725.1
807.8
117.8
155.1
216.9
249.1
274.2
305.0
329.9
357.0
382.4
547.5
577.5
642.7
674.6
736.5
770.4
795.7
845.0
895.4
931.2
955.8
998.9
1053.9
200
400
600
800
1000
m/z

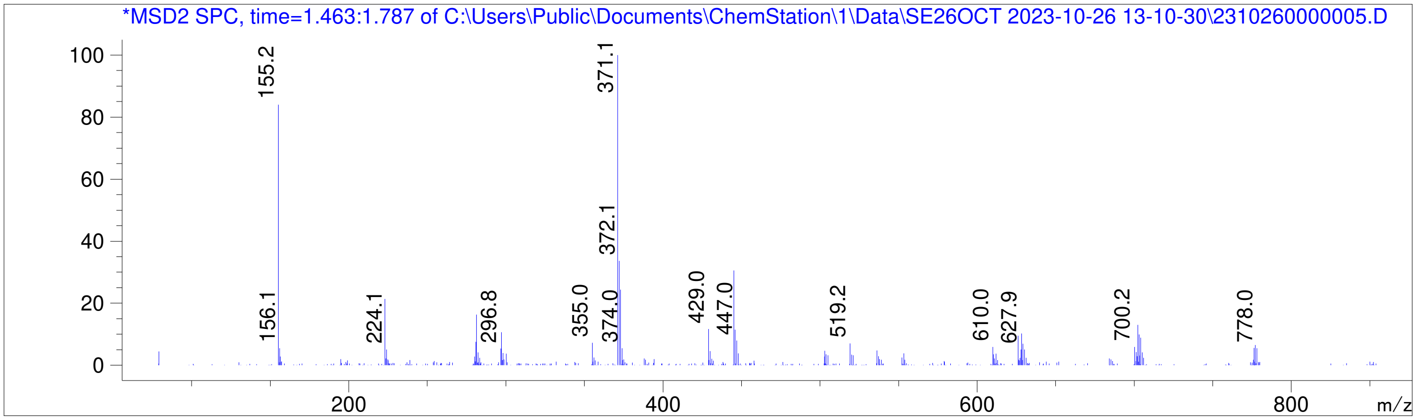
*MSD2 SPC, time=1.463:1.787 of C:\Users\Public\Documents\ChemStation\1\Data\SE26OCT 2023-10-26 13-10-30\2310260000005.D
100
80
60
40
20
0
155.2
371.1
372.1
156.1
224.1
296.8
355.0
374.0
429.0
447.0
519.2
610.0
627.9
700.2
778.0
200
400
600
800
m/z

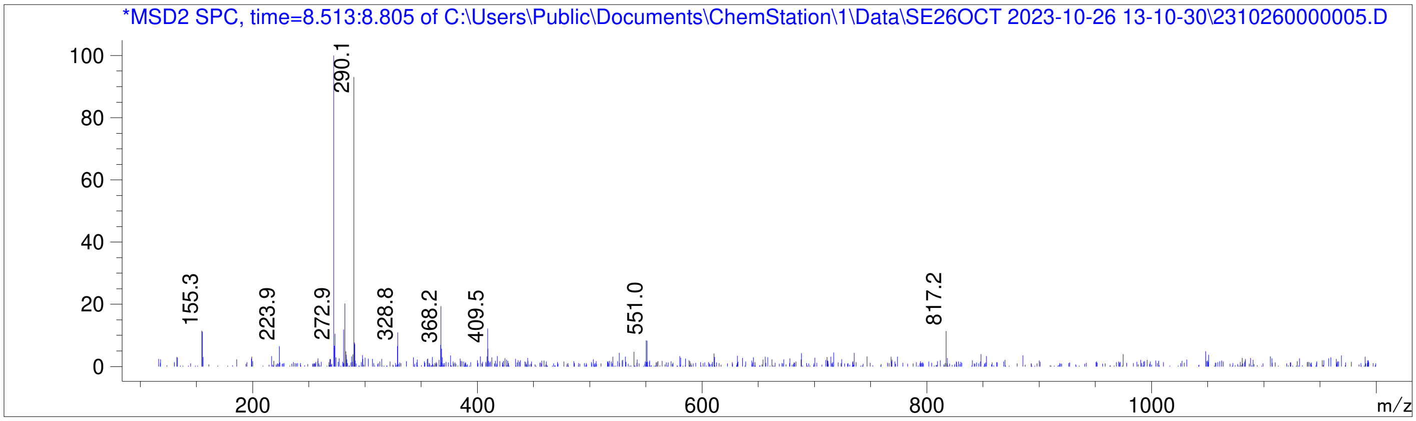
*MSD2 SPC, time=8.513:8.805 of C:\Users\Public\Documents\ChemStation\1\Data\SE26OCT 2023-10-26 13-10-30\2310260000005.D
100
80
60
40
20
0
290.1
155.3
223.9
272.9
328.8
368.2
409.5
551.0
817.2
200
400
600
800
1000
m/z

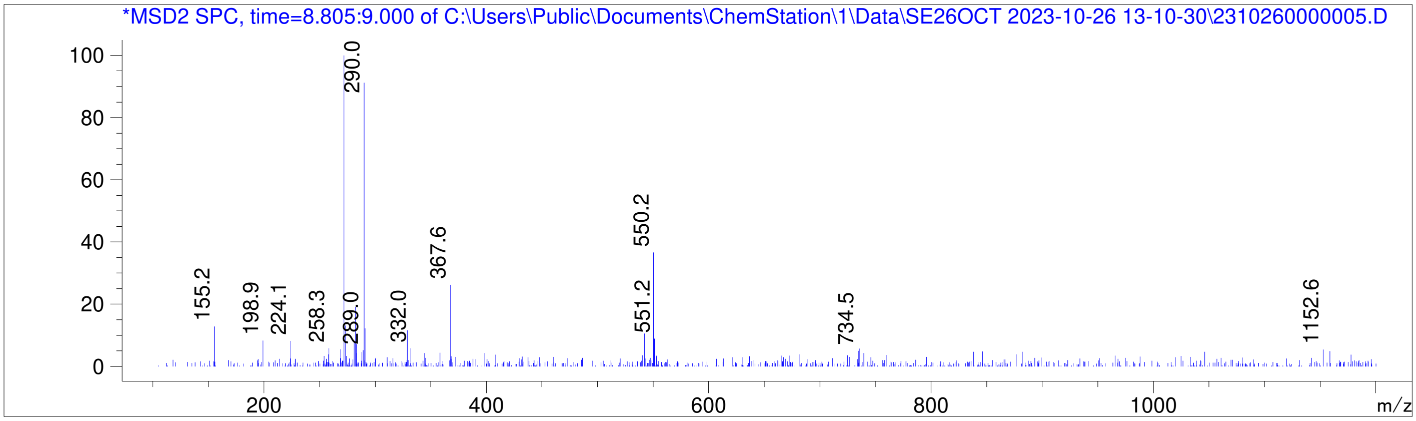
*MSD2 SPC, time=8.805:9.000 of C:\Users\Public\Documents\ChemStation\1\Data\SE26OCT 2023-10-26 13-10-30\2310260000005.D
100
80
60
40
20
0
290.0
550.2
367.6
155.2
198.9
224.1
258.3
289.0
332.0
551.2
734.5
1152.6
200
400
600
800
1000
m/z

==

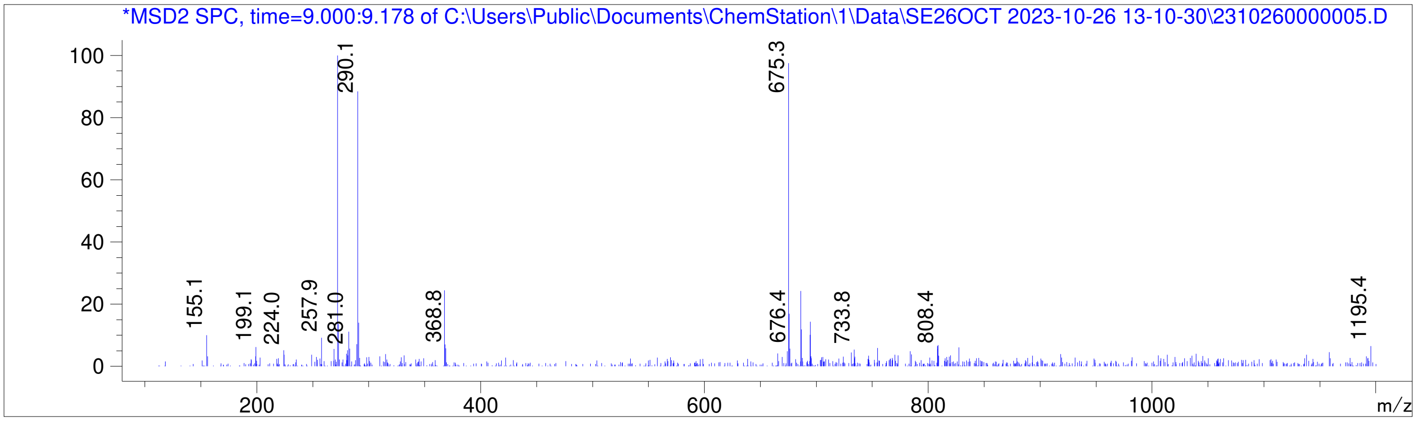
*MSD2 SPC, time=9.000:9.178 of C:\Users\Public\Documents\ChemStation\1\Data\SE26OCT 2023-10-26 13-10-30\2310260000005.D
155.1
199.1
224.0
257.9
281.0
290.1
368.8
675.3
676.4
733.8
808.4
1195.4
m/z

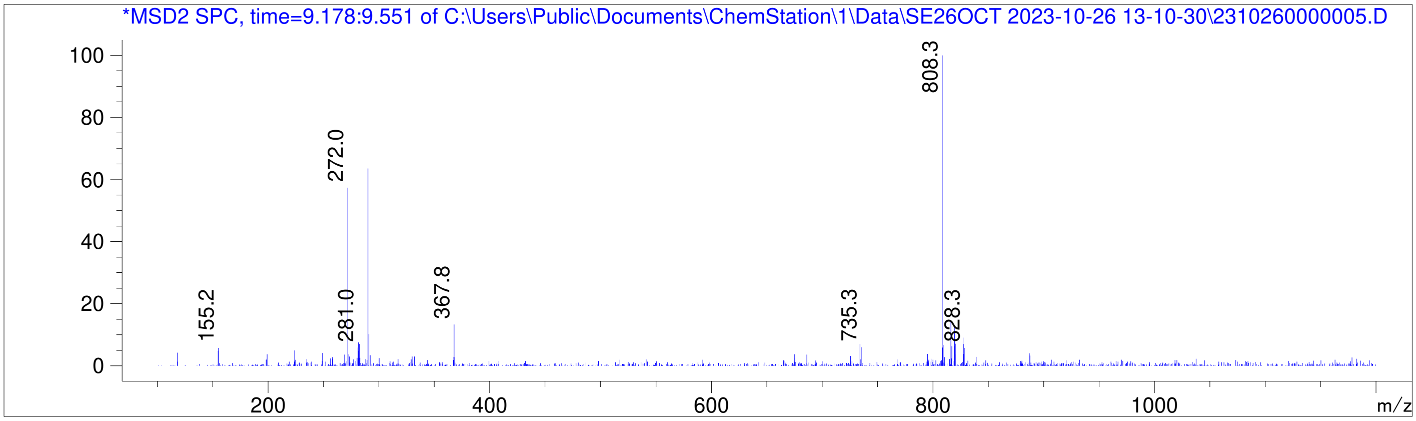
*MSD2 SPC, time=9.178:9.551 of C:\Users\Public\Documents\ChemStation\1\Data\SE26OCT 2023-10-26 13-10-30\2310260000005.D
155.2
272.0
281.0
367.8
735.3
808.3
828.3
m/z

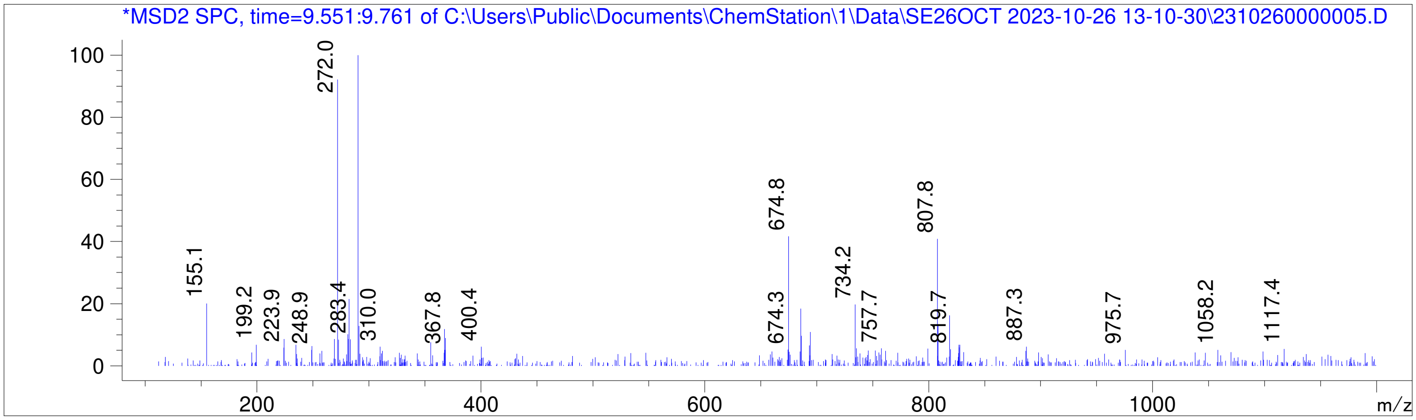
*MSD2 SPC, time=9.551:9.761 of C:\Users\Public\Documents\ChemStation\1\Data\SE26OCT 2023-10-26 13-10-30\2310260000005.D
155.1
199.2
223.9
248.9
272.0
283.4
310.0
367.8
400.4
674.3
674.8
734.2
757.7
807.8
819.7
887.3
975.7
1058.2
1117.4
m/z

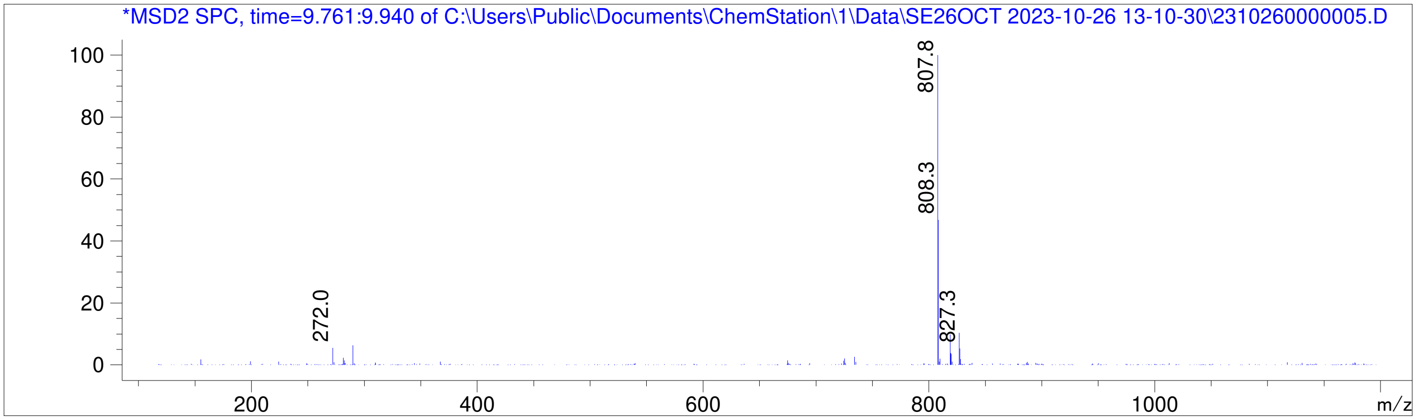
*MSD2 SPC, time=9.761:9.940 of C:\Users\Public\Documents\ChemStation\1\Data\SE26OCT 2023-10-26 13-10-30\2310260000005.D
272.0
807.8
808.3
827.3
m/z

===

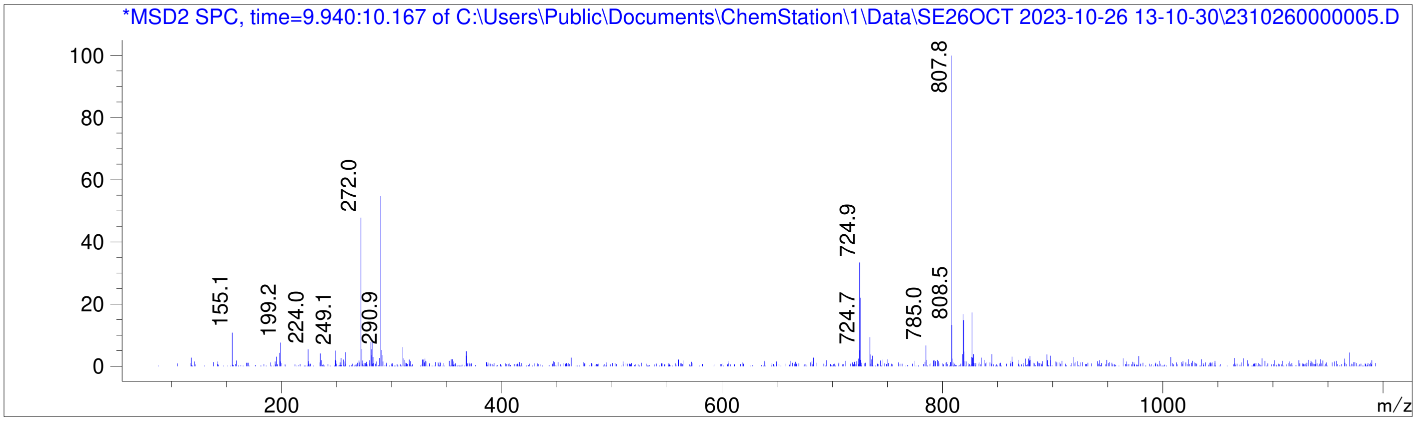
*MSD2 SPC, time=9.940:10.167 of C:\Users\Public\Documents\ChemStation\1\Data\SE26OCT 2023-10-26 13-10-30\2310260000005.D
100
80
60
40
20
0
155.1
199.2
224.0
249.1
272.0
290.9
724.7
724.9
785.0
807.8
808.5
200
400
600
800
1000
m/z
