# Supplementary material for: Astrobiological implications of the stability and reactivity of peptide nucleic acid (PNA) in concentrated sulfuric acid
Source: Sci Adv. 2025 Mar 26;11(13):eadr0006. doi: 10.1126/sciadv.adr0006 (PMC11939054; doi:10.1126/sciadv.adr0006)
Supplement: Supplementary file 2 — Data S1 and S2 [file sciadv.adr0006_data_s1_and_s2.zip › Supplementary Dataset 1-LCMS DATA/LCMS PNA Hexamers A-T/LCMS T6 RT/14d/CPT22010446-19-D3-14d.pdf]

Injection Date	:	Mon, 16. Oct. 2023	Seq Line	:	13
			Location	:	86
			Inj. Vol.	:	2 µl

Acq. Method : C:\Users\Public\Documents\ChemStation\1\Data\SE16OCT 2023-10-16 10-35-31\22010446C LCMS-6#.M
Analysis Method : C:\Users\Public\Documents\ChemStation\1\Data\SE16OCT 2023-10-16 10-35-31\22010446C LCMS-6#.M (Sequence Method)
Waters XBridge BEH Amide (4.6 x 150 mm, 2.5 µm); PN# 186006726
Mobile Phase A: 20mM Ammonium Acetate (aq) pH 8.2
Mobile Phase B: AcN
Mobile Phase A / Mobile Phase B: 5/95 (0 min) --> (10 min) --> 60/40 (5 min); Flow: 1.0 ml/min; MSD1 = positive; MSD2 = negative

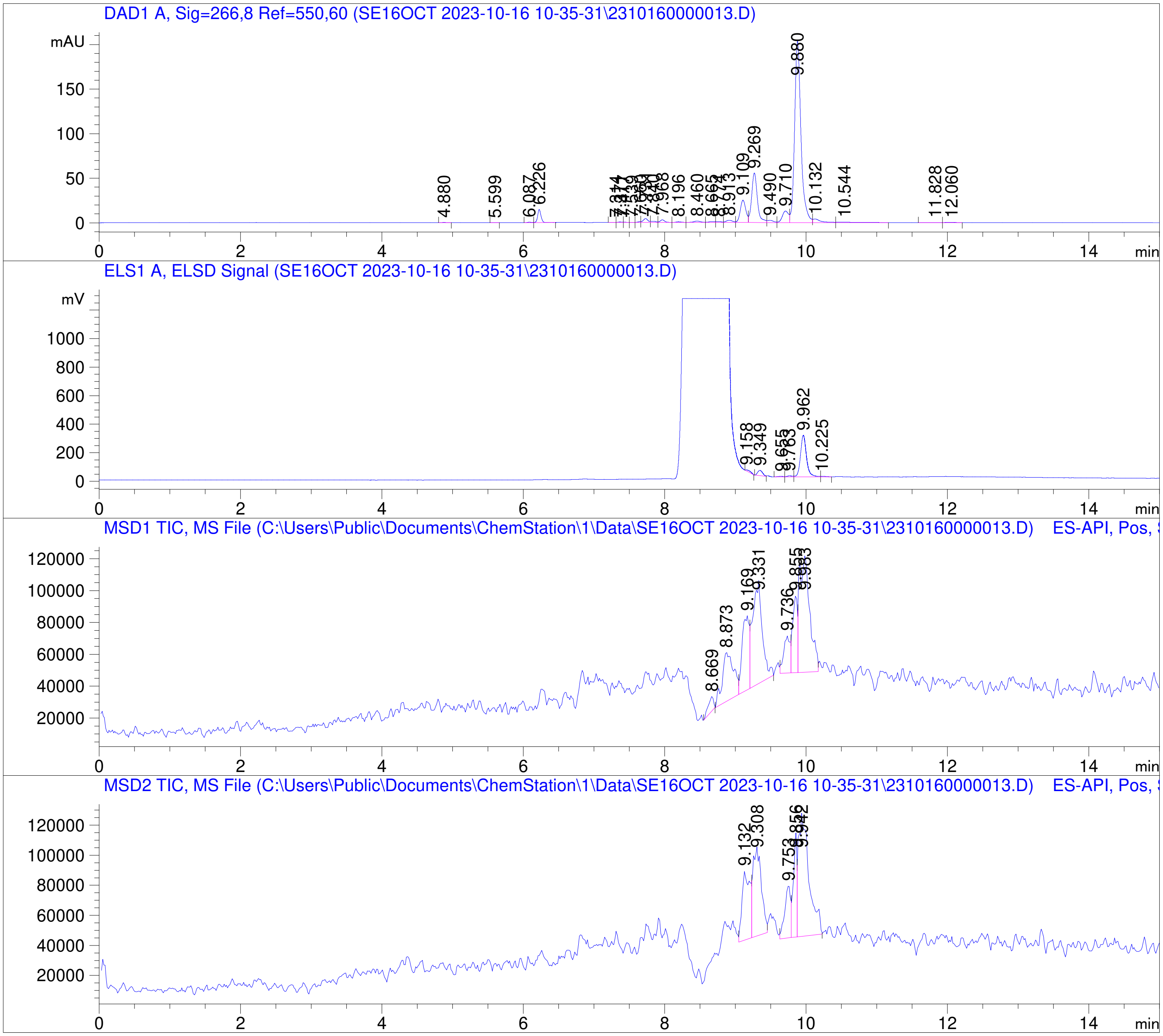

DAD1 A, Sig=266,8 Ref=550,60

Peak #	Ret. Time [min]	Area [mV *s]	Area %
1	4.880	0.695	0.035
2	5.599	0.356	0.018
3	6.087	1.768	0.088
4	6.226	48.045	2.396
5	7.314	0.448	0.022
6	7.371	2.600	0.130
7	7.417	1.222	0.061
8	7.539	0.783	0.039
9	7.660	2.786	0.139
10	7.731	20.528	1.024
11	7.840	7.387	0.368
12	7.968	14.236	0.710
13	8.196	5.373	0.268
14	8.460	12.431	0.620
15	8.665	6.816	0.340
16	8.774	5.757	0.287
17	8.913	16.821	0.839
18	9.109	137.253	6.844
19	9.269	313.635	15.638
20	9.490	14.677	0.732
21	9.710	78.142	3.896
22	9.880	1273.715	63.508
23	10.132	30.370	1.514
24	10.544	8.236	0.411
25	11.828	0.771	0.038
26	12.060	0.733	0.037

ELS1 A, ELSD Signal

Peak #	Ret. Time [min]	Area [mV *s]	Area %
1	9.158	52.179	2.539
2	9.349	180.343	8.775
3	9.655	23.452	1.141
4	9.763	44.004	2.141
5	9.962	1730.972	84.222
6	10.225	24.295	1.182

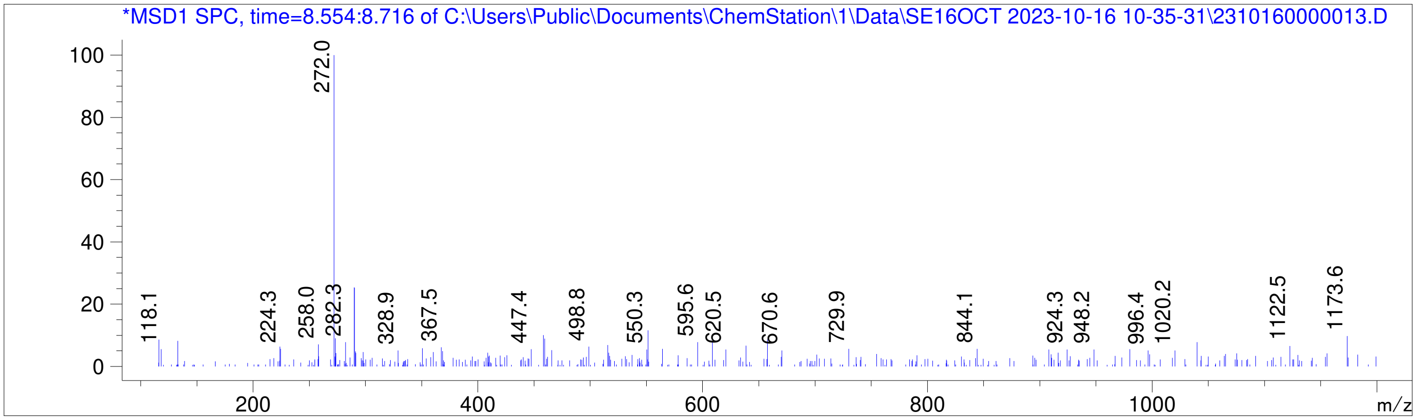
*MSD1 SPC, time=8.554:8.716 of C:\Users\Public\Documents\ChemStation\1\Data\SE16OCT 2023-10-16 10-35-31\2310160000013.D
272.0
118.1
224.3
258.0
282.3
328.9
367.5
447.4
498.8
550.3
595.6
620.5
670.6
729.9
844.1
924.3
948.2
996.4
1020.2
1122.5
1173.6
m/z

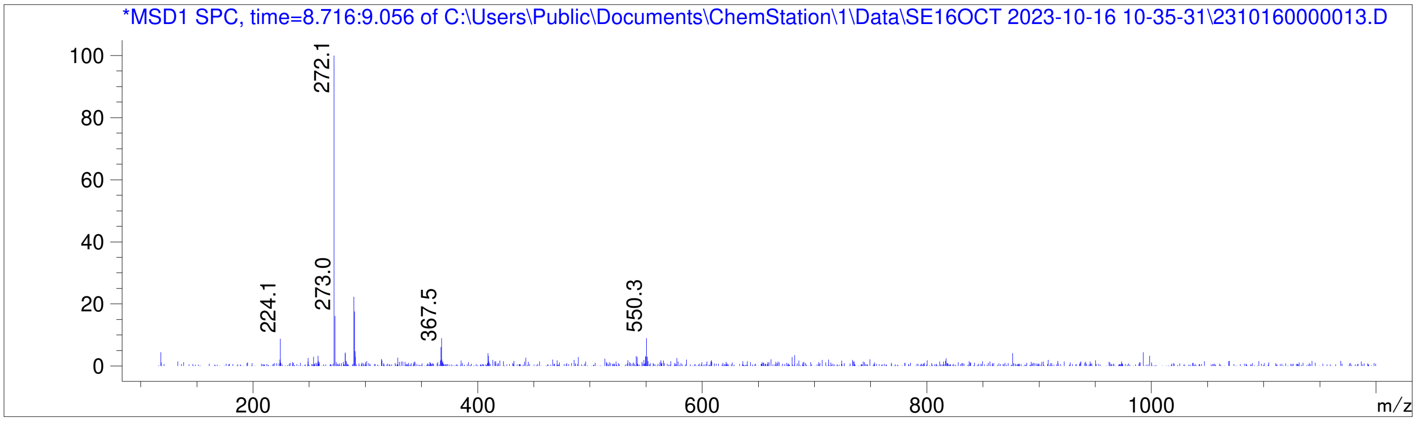
*MSD1 SPC, time=8.716:9.056 of C:\Users\Public\Documents\ChemStation\1\Data\SE16OCT 2023-10-16 10-35-31\2310160000013.D
272.1
224.1
273.0
367.5
550.3
m/z

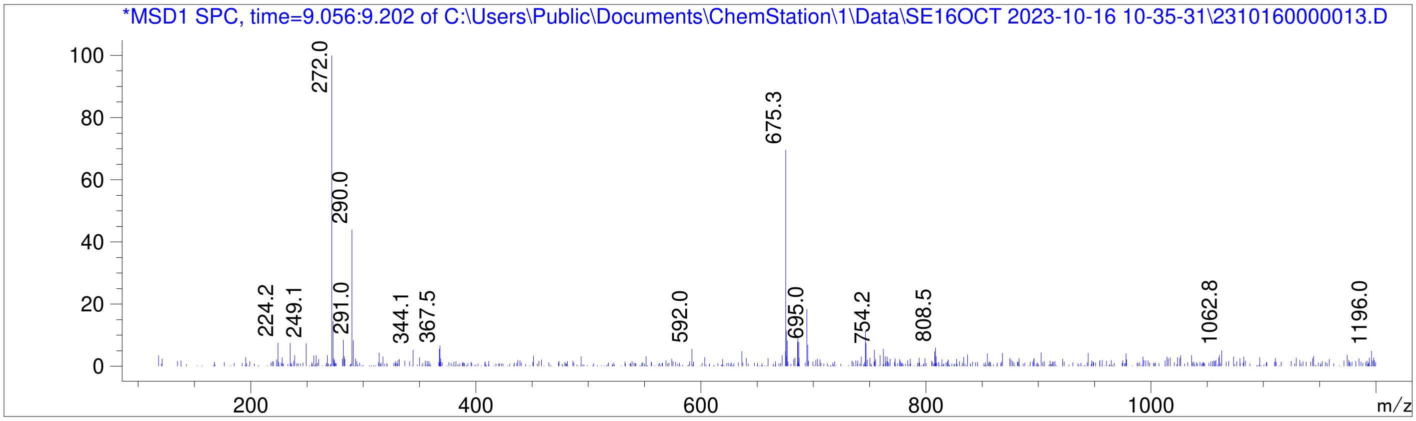
*MSD1 SPC, time=9.056:9.202 of C:\Users\Public\Documents\ChemStation\1\Data\SE16OCT 2023-10-16 10-35-31\2310160000013.D
272.0
675.3
290.0
224.2
249.1
291.0
344.1
367.5
592.0
695.0
754.2
808.5
1062.8
1196.0
m/z

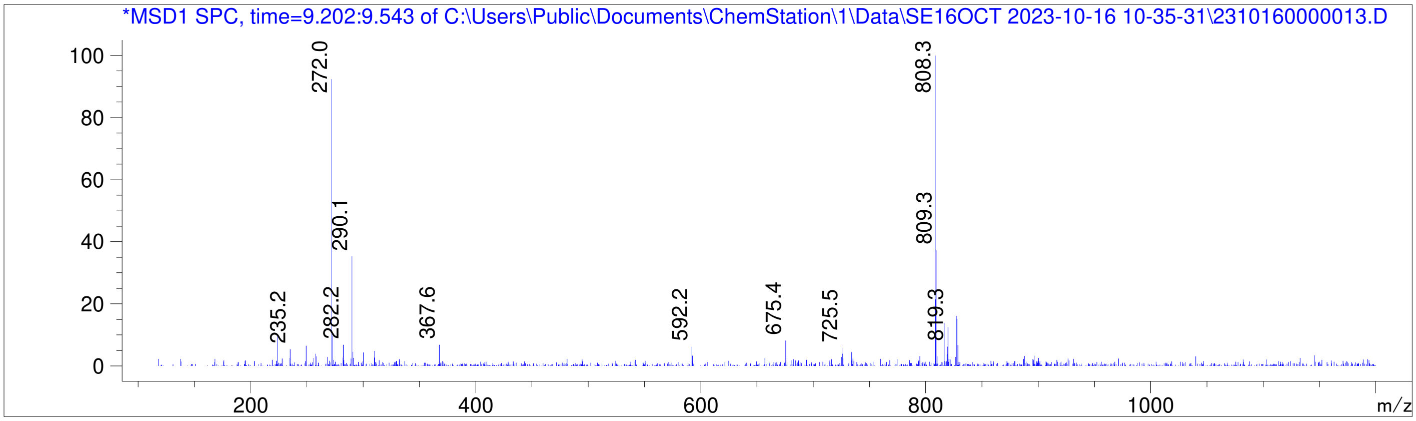
*MSD1 SPC, time=9.202:9.543 of C:\Users\Public\Documents\ChemStation\1\Data\SE16OCT 2023-10-16 10-35-31\231016000013.D
100
80
60
40
20
0
272.0
808.3
809.3
290.1
235.2
282.2
367.6
592.2
675.4
725.5
819.3
200
400
600
800
1000
m/z

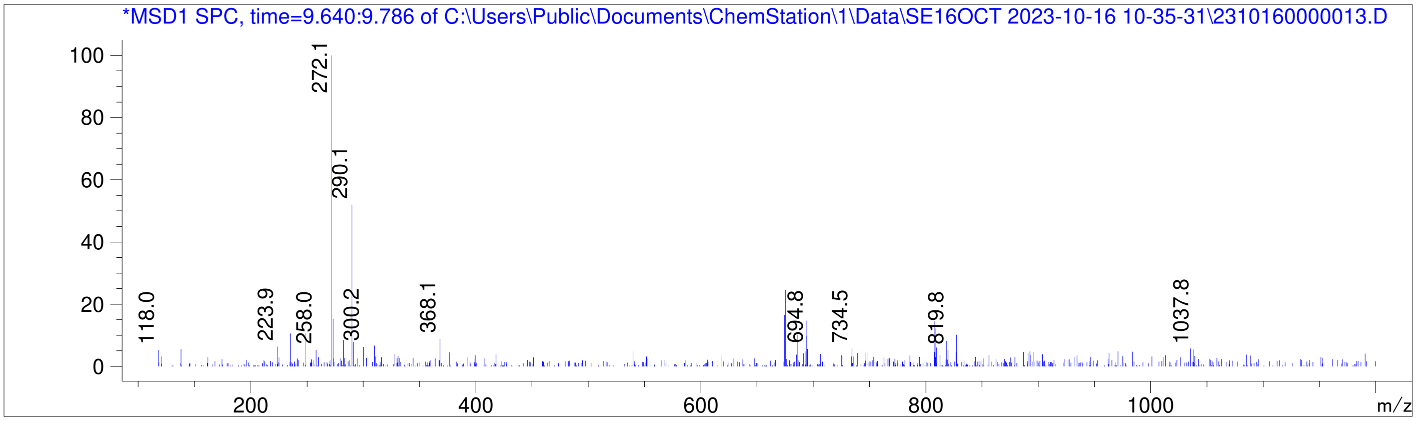
*MSD1 SPC, time=9.640:9.786 of C:\Users\Public\Documents\ChemStation\1\Data\SE16OCT 2023-10-16 10-35-31\231016000013.D
100
80
60
40
20
0
272.1
290.1
118.0
223.9
258.0
300.2
368.1
694.8
734.5
819.8
1037.8
200
400
600
800
1000
m/z

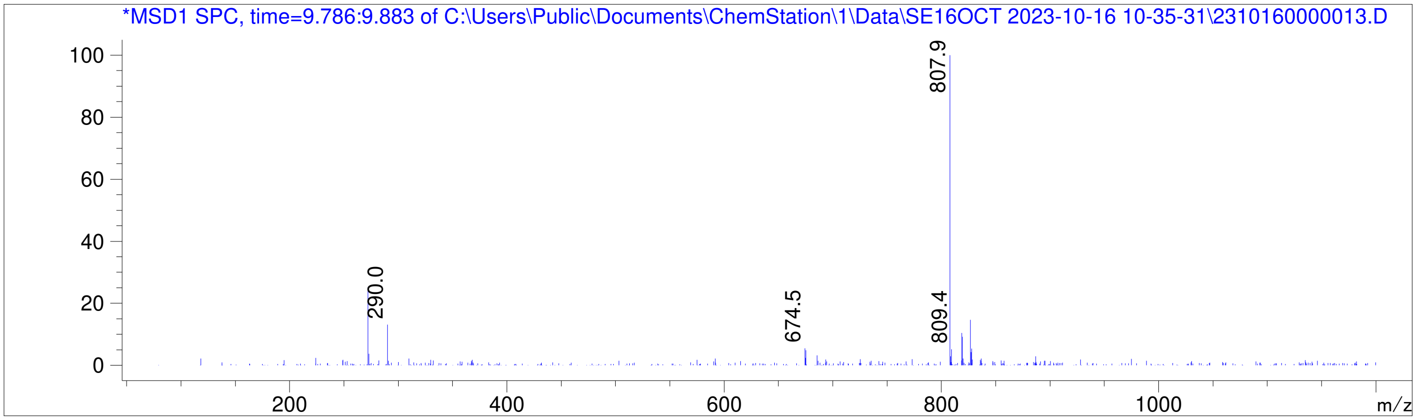
*MSD1 SPC, time=9.786:9.883 of C:\Users\Public\Documents\ChemStation\1\Data\SE16OCT 2023-10-16 10-35-31\231016000013.D
100
80
60
40
20
0
807.9
290.0
674.5
809.4
200
400
600
800
1000
m/z

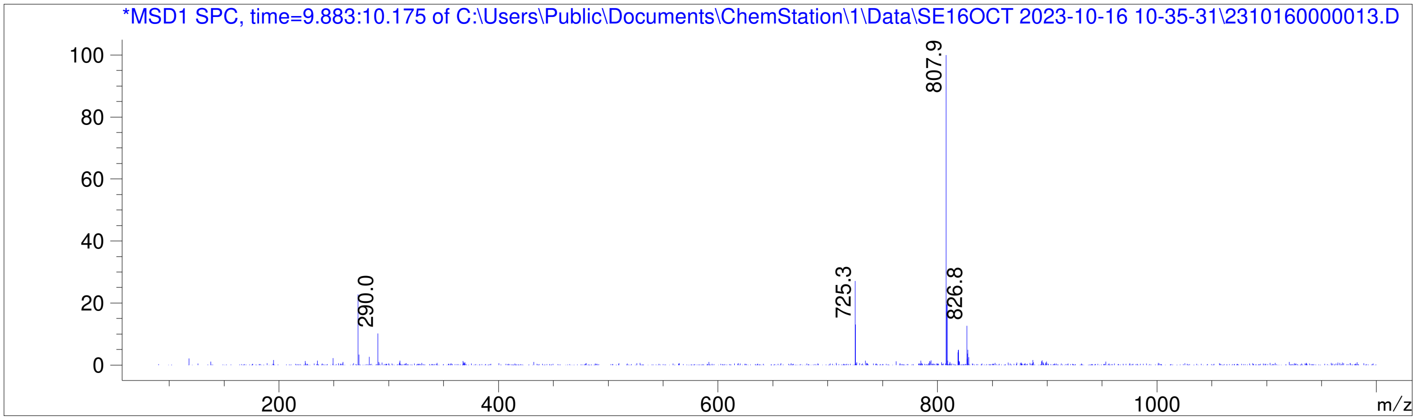
*MSD1 SPC, time=9.883:10.175 of C:\Users\Public\Documents\ChemStation\1\Data\SE16OCT 2023-10-16 10-35-31\231016000013.D
100
80
60
40
20
0
807.9
290.0
725.3
826.8
200
400
600
800
1000
m/z

==

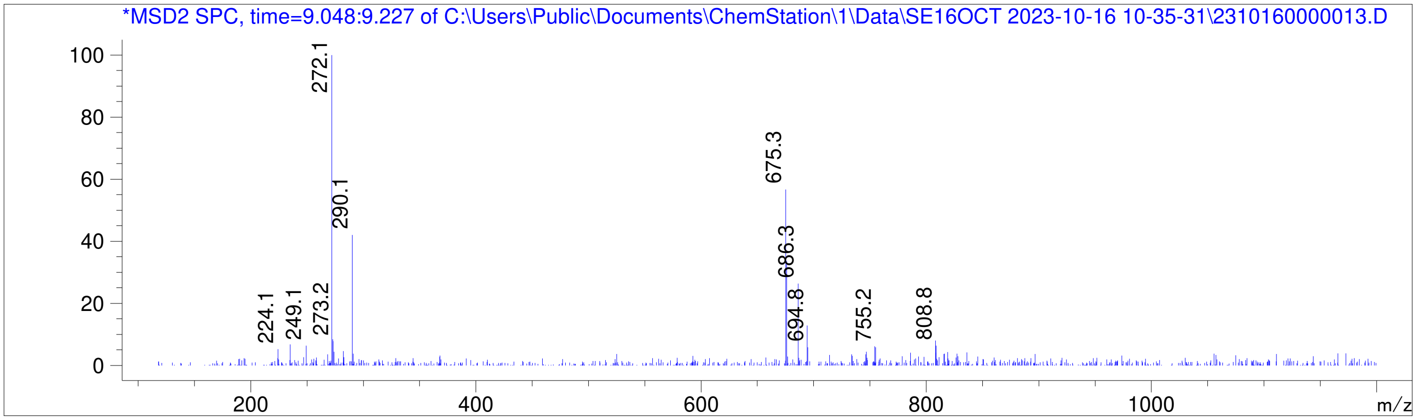
*MSD2 SPC, time=9.048:9.227 of C:\Users\Public\Documents\ChemStation\1\Data\SE16OCT 2023-10-16 10-35-31\2310160000013.D
272.1
290.1
224.1
249.1
273.2
675.3
686.3
694.8
755.2
808.8
m/z

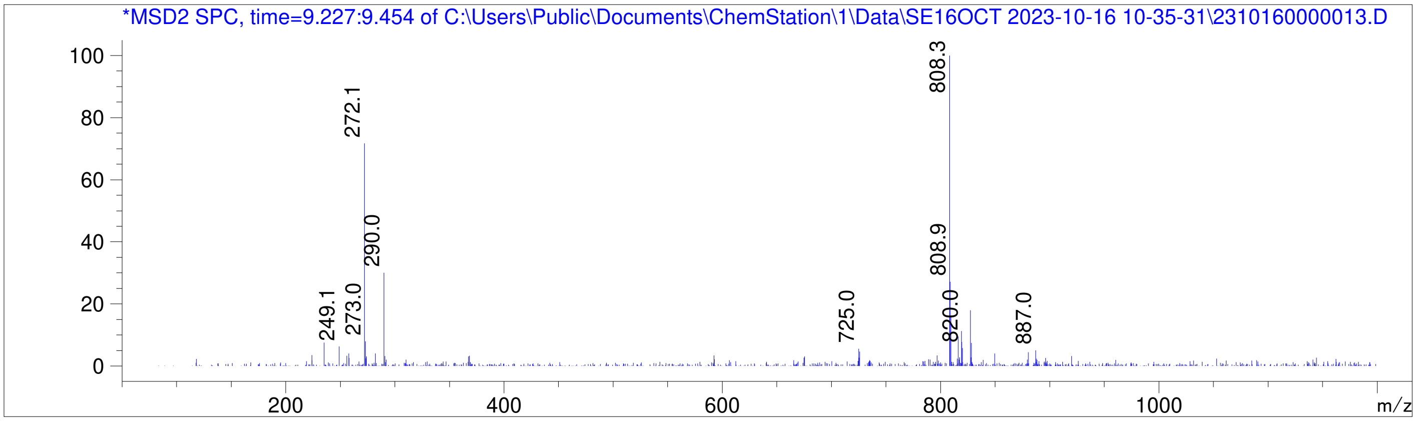
*MSD2 SPC, time=9.227:9.454 of C:\Users\Public\Documents\ChemStation\1\Data\SE16OCT 2023-10-16 10-35-31\2310160000013.D
808.3
272.1
290.0
808.9
249.1
273.0
725.0
820.0
887.0
m/z

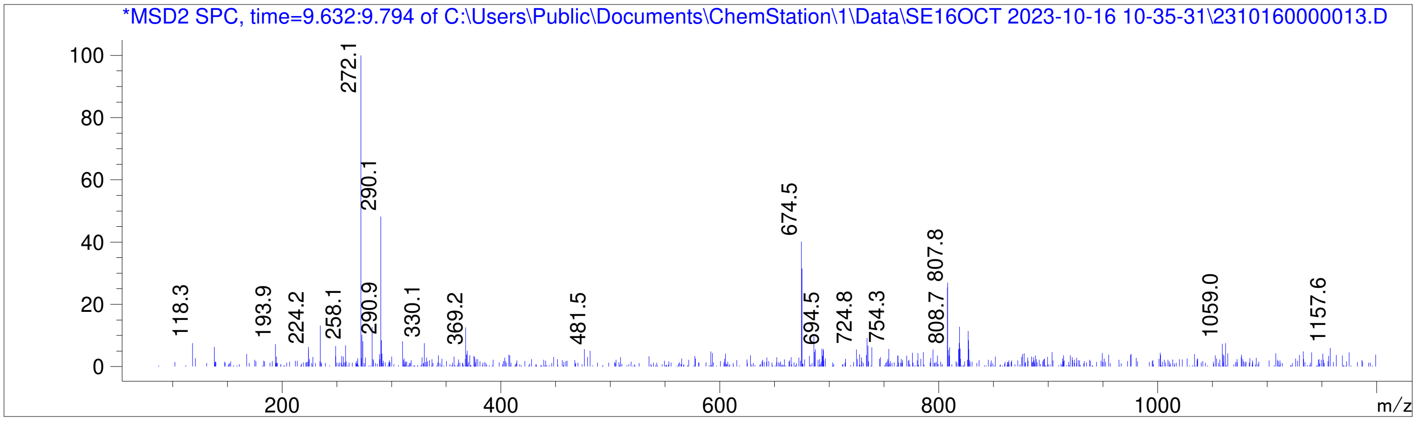
*MSD2 SPC, time=9.632:9.794 of C:\Users\Public\Documents\ChemStation\1\Data\SE16OCT 2023-10-16 10-35-31\2310160000013.D
272.1
290.1
674.5
807.8
118.3
193.9
224.2
258.1
290.9
330.1
369.2
481.5
694.5
724.8
754.3
808.7
1059.0
1157.6
m/z

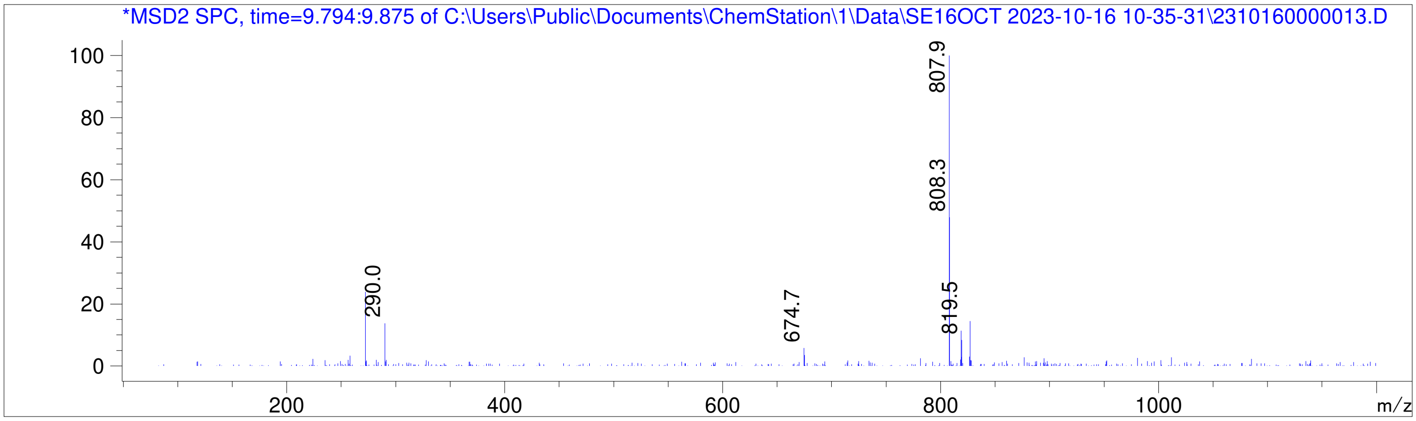
*MSD2 SPC, time=9.794:9.875 of C:\Users\Public\Documents\ChemStation\1\Data\SE16OCT 2023-10-16 10-35-31\2310160000013.D
100
80
60
40
20
0
807.9
808.3
290.0
674.7
819.5
200
400
600
800
1000
m/z

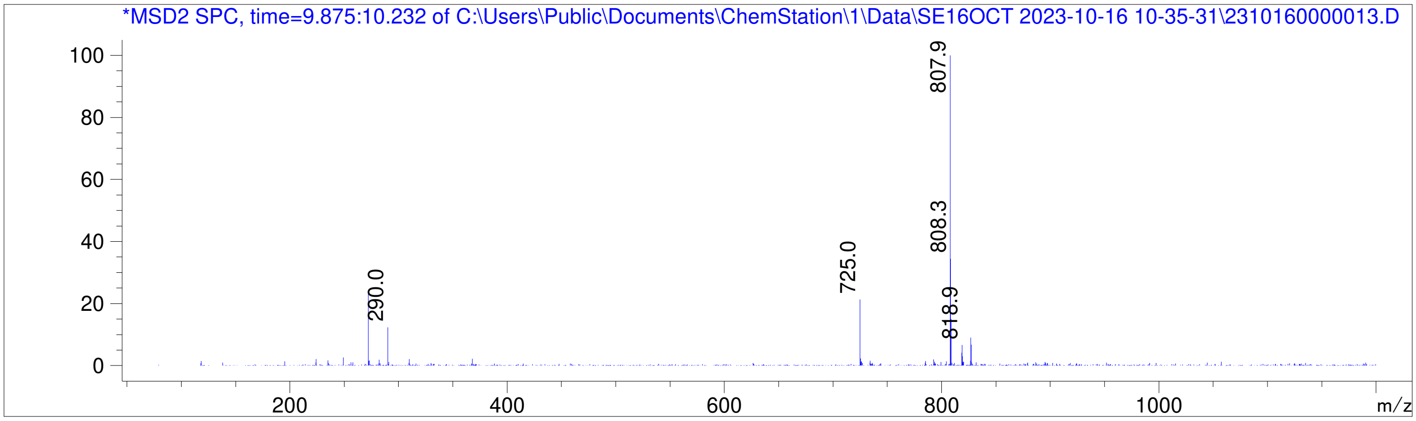
*MSD2 SPC, time=9.875:10.232 of C:\Users\Public\Documents\ChemStation\1\Data\SE16OCT 2023-10-16 10-35-31\2310160000013.D
100
80
60
40
20
0
807.9
808.3
725.0
290.0
818.9
200
400
600
800
1000
m/z
