# Supplementary material for: Astrobiological implications of the stability and reactivity of peptide nucleic acid (PNA) in concentrated sulfuric acid
Source: Sci Adv. 2025 Mar 26;11(13):eadr0006. doi: 10.1126/sciadv.adr0006 (PMC11939054; doi:10.1126/sciadv.adr0006)
Supplement: Supplementary file 2 — Data S1 and S2 [file sciadv.adr0006_data_s1_and_s2.zip › Supplementary Dataset 1-LCMS DATA/LCMS PNA Hexamers A-T/LCMS T6 RT/1h/CPT22010446-19-D1.pdf]

Data -> C:\Users\Public\Documents\ChemStation\1\Data\SE10OCT 2023-10-10 08-36-32\ ->
Sample-> CPT22010446-19-D1

===

Injection Date : Tue, 10. Oct. 2023

Seq Line : 22
Location : 15
Inj. Vol. : 2 µl

Acq. Method : C:\Users\Public\Documents\ChemStation\1\Data\SE10OCT 2023-10-10 08-36-32\22010446C LCMS-6#.M
Analysis Method : C:\Users\Public\Documents\ChemStation\1\Data\SE10OCT 2023-10-10 08-36-32\22010446C LCMS-6#.M (Sequence Method)
Waters XBridge BEH Amide (4.6 x 150 mm, 2.5 µm); PN# 186006726
Mobile Phase A: 20mM Ammonium Acetate (aq) pH 8.2
Mobile Phase B: AcN
Mobile Phase A / Mobile Phase B: 5/95 (0 min) --> (10 min) --> 60/40 (5 min); Flow: 1.0 ml/min; MSD1 = positive; MSD2 = negative

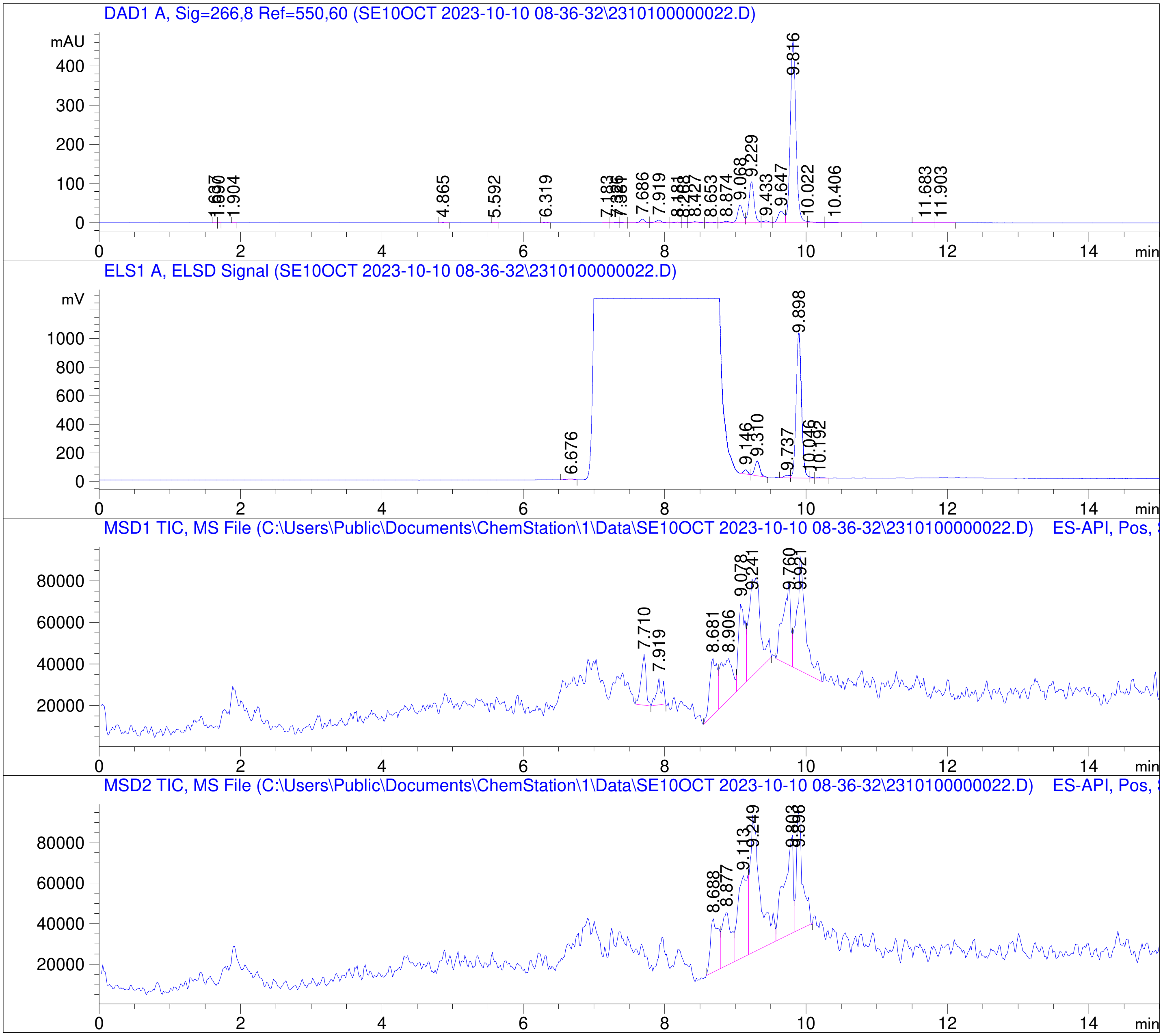

DAD1 A, Sig=266,8 Ref=550,60

Peak #	Ret. Time [min]	Area [mV *s]	Area %
1	1.637	0.281	0.008
2	1.690	0.172	0.005
3	1.904	0.230	0.006
4	4.865	2.117	0.057
5	5.592	0.572	0.015
6	6.319	3.748	0.100
7	7.183	0.314	0.008
8	7.326	3.187	0.085
9	7.381	2.920	0.078
10	7.686	40.366	1.079
11	7.919	37.157	0.993
12	8.181	10.031	0.268
13	8.268	3.818	0.102
14	8.427	18.076	0.483
15	8.653	10.420	0.278
16	8.874	20.829	0.557
17	9.068	233.981	6.252
18	9.229	529.875	14.157
19	9.433	27.967	0.747
20	9.647	179.964	4.808
21	9.816	2584.500	69.054
22	10.022	19.969	0.534
23	10.406	9.857	0.263
24	11.683	1.416	0.038
25	11.903	0.972	0.026

ELS1 A, ELSD Signal

Peak #	Ret. Time [min]	Area [mV *s]	Area %
1	6.676	42.620	0.694
2	9.146	124.730	2.030
3	9.310	504.096	8.205
4	9.737	97.254	1.583
5	9.898	5316.391	86.532
6	10.046	23.156	0.377
7	10.192	35.578	0.579

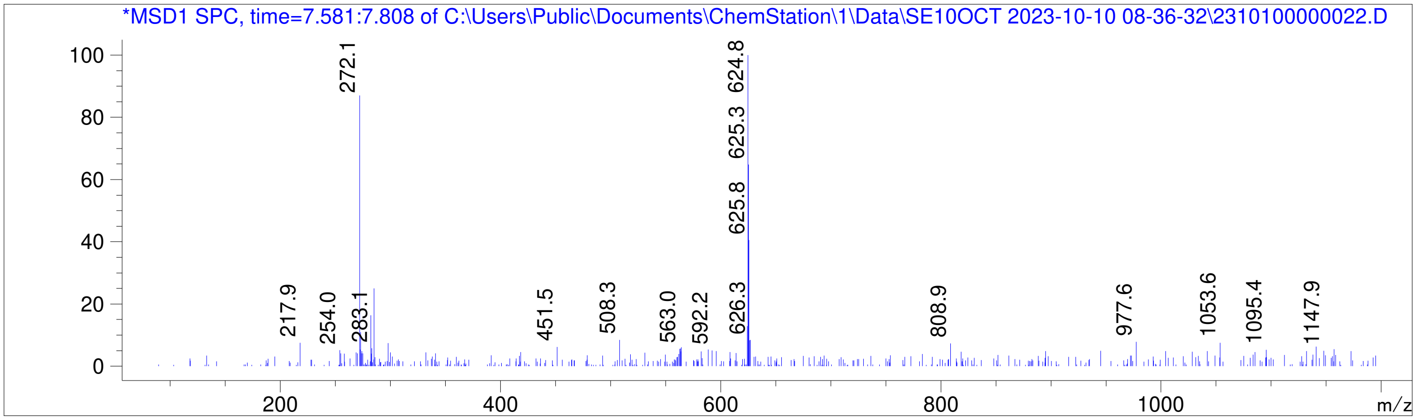
*MSD1 SPC, time=7.581:7.808 of C:\Users\Public\Documents\ChemStation\1\Data\SE10OCT 2023-10-10 08-36-32\2310100000022.D
272.1
624.8
625.3
625.8
217.9
254.0
283.1
451.5
508.3
563.0
592.2
626.3
808.9
977.6
1053.6
1095.4
1147.9
m/z

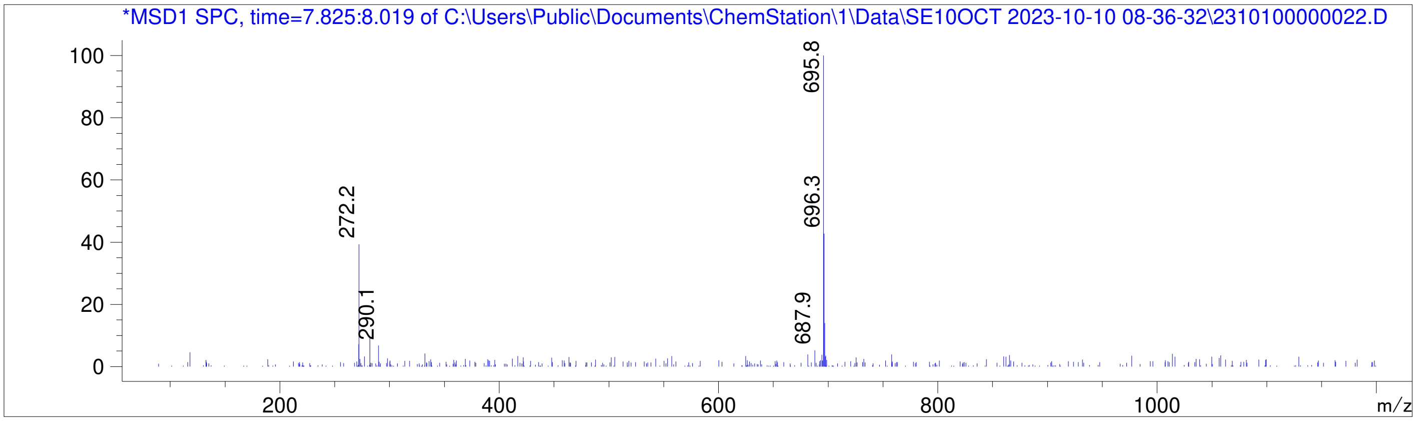
*MSD1 SPC, time=7.825:8.019 of C:\Users\Public\Documents\ChemStation\1\Data\SE10OCT 2023-10-10 08-36-32\2310100000022.D
695.8
696.3
272.2
290.1
687.9
m/z

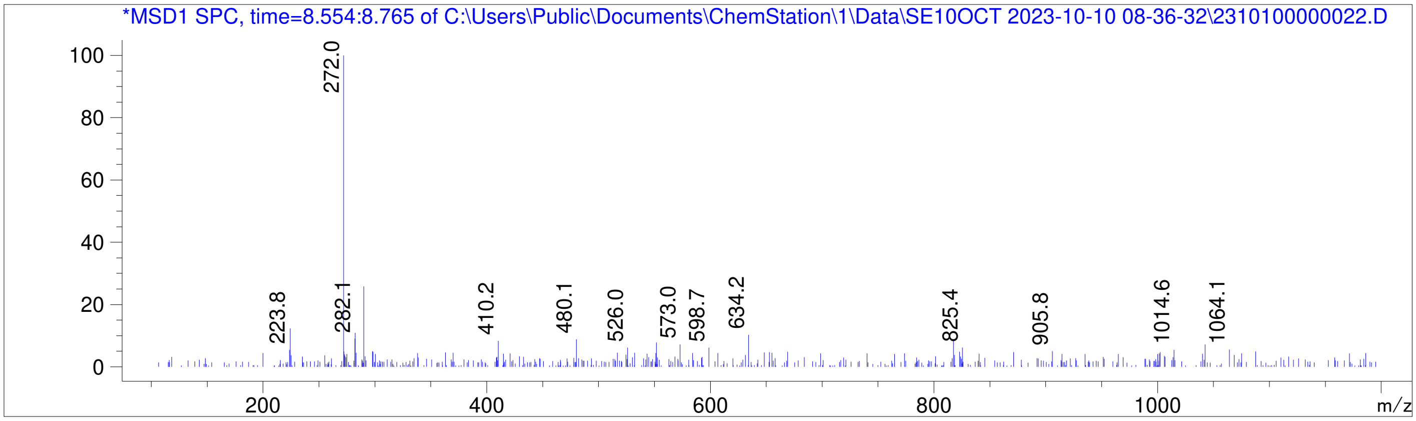
*MSD1 SPC, time=8.554:8.765 of C:\Users\Public\Documents\ChemStation\1\Data\SE10OCT 2023-10-10 08-36-32\2310100000022.D
272.0
223.8
282.1
410.2
480.1
526.0
573.0
598.7
634.2
825.4
905.8
1014.6
1064.1
m/z

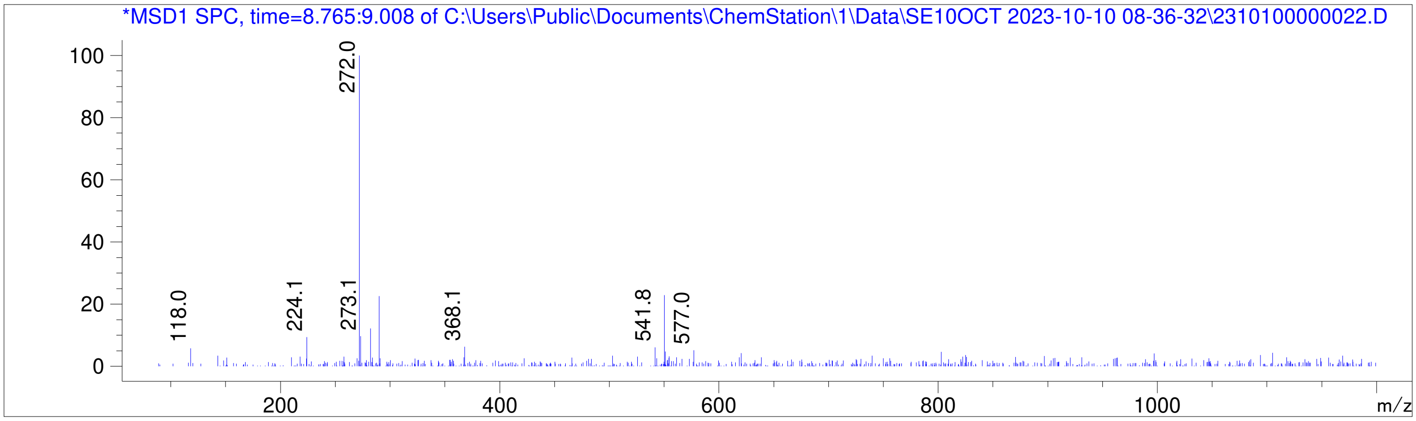
*MSD1 SPC, time=8.765:9.008 of C:\Users\Public\Documents\ChemStation\1\Data\SE10OCT 2023-10-10 08-36-32\2310100000022.D
272.0
118.0
224.1
273.1
368.1
541.8
577.0
m/z

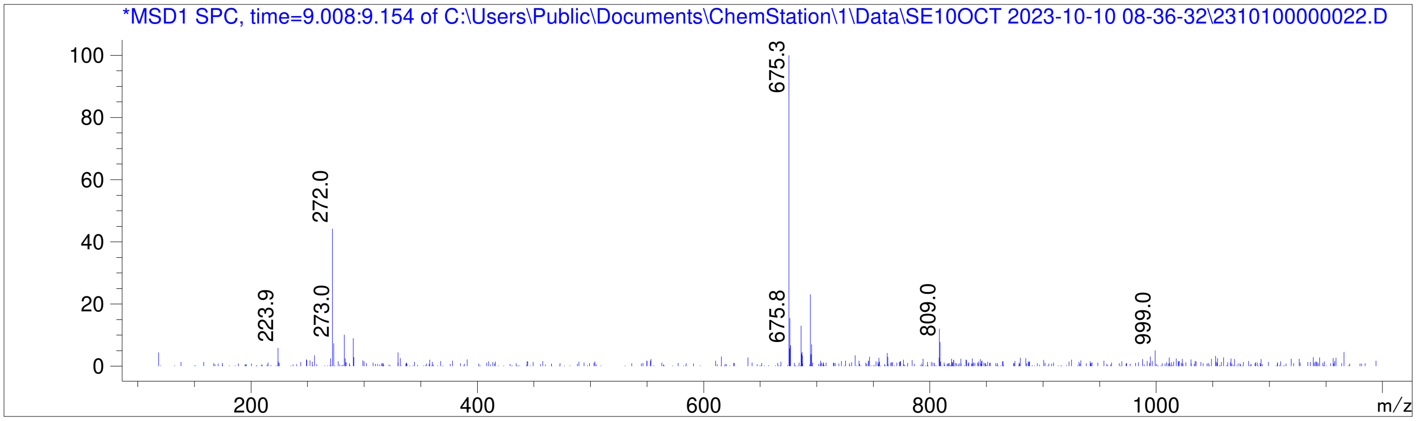
*MSD1 SPC, time=9.008:9.154 of C:\Users\Public\Documents\ChemStation\1\Data\SE10OCT 2023-10-10 08-36-32\2310100000022.D
675.3
272.0
223.9
273.0
675.8
809.0
999.0
m/z

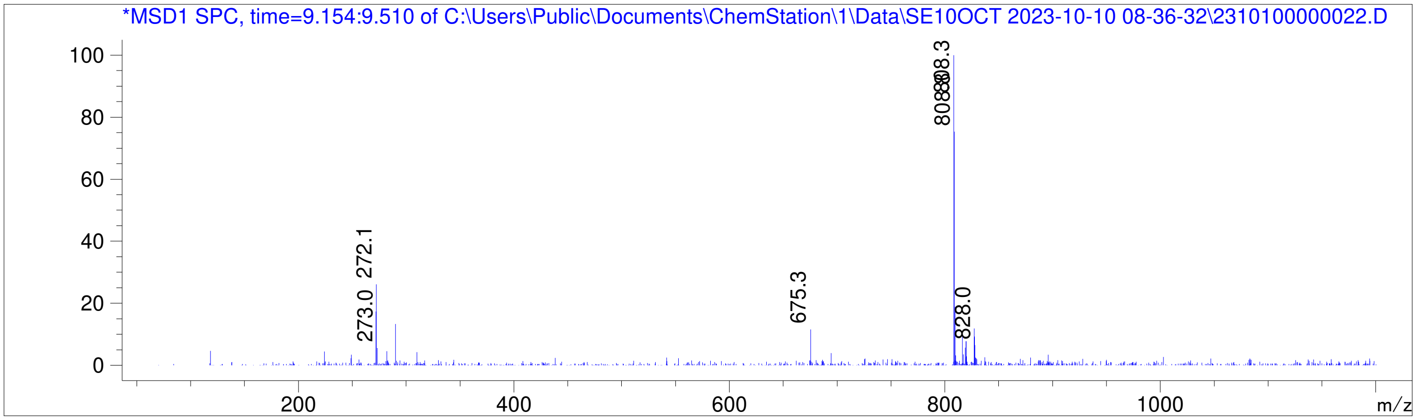
*MSD1 SPC, time=9.154:9.510 of C:\Users\Public\Documents\ChemStation\1\Data\SE10OCT 2023-10-10 08-36-32\2310100000022.D
808.3
272.1
273.0
675.3
828.0
m/z

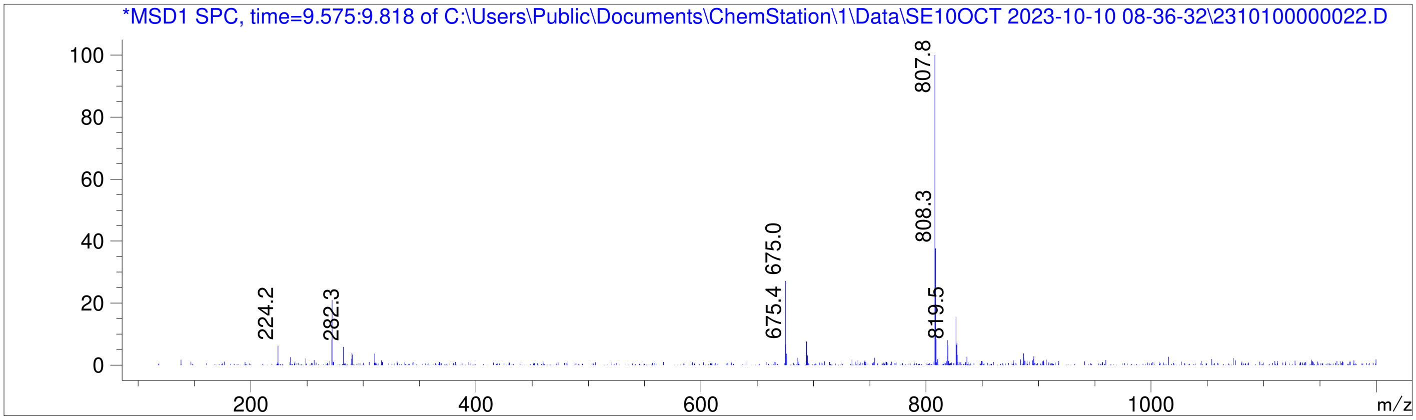
*MSD1 SPC, time=9.575:9.818 of C:\Users\Public\Documents\ChemStation\1\Data\SE10OCT 2023-10-10 08-36-32\2310100000022.D
807.8
808.3
675.0
224.2
282.3
675.4
819.5
m/z

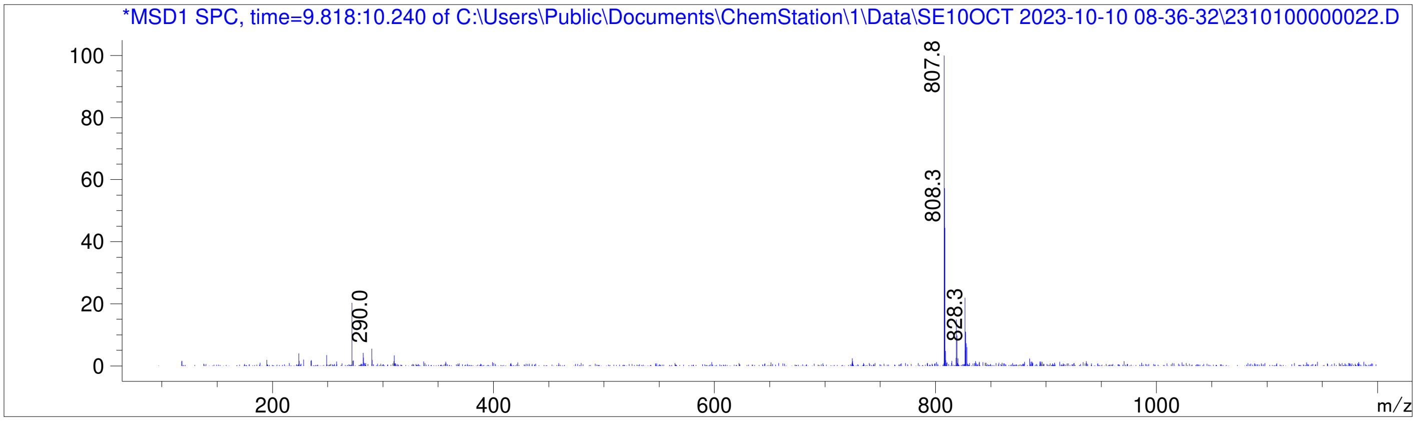
*MSD1 SPC, time=9.818:10.240 of C:\Users\Public\Documents\ChemStation\1\Data\SE10OCT 2023-10-10 08-36-32\2310100000022.D
100
80
60
40
20
0
807.8
808.3
828.3
290.0
200
400
600
800
1000
m/z

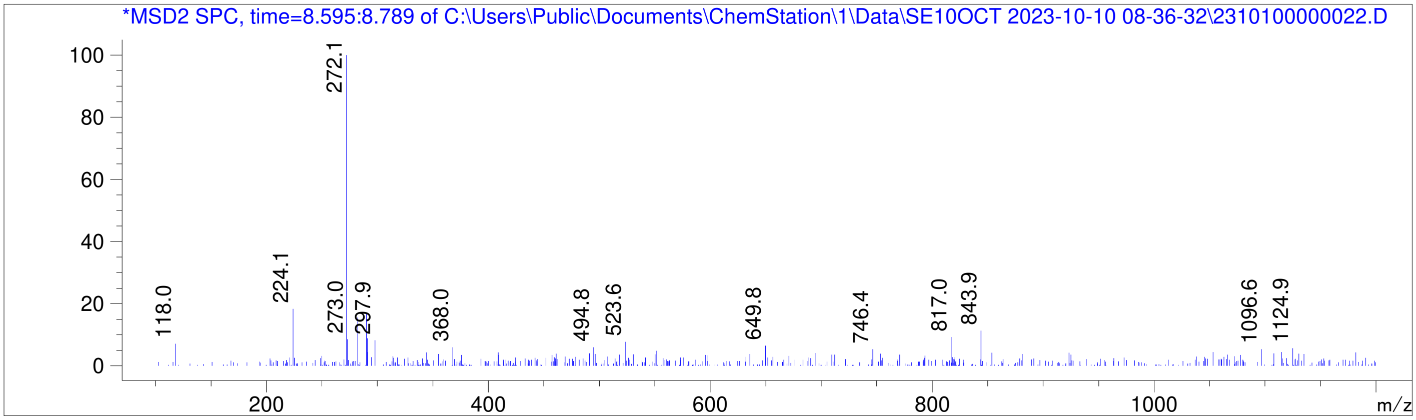
*MSD2 SPC, time=8.595:8.789 of C:\Users\Public\Documents\ChemStation\1\Data\SE10OCT 2023-10-10 08-36-32\2310100000022.D
272.1
224.1
118.0
273.0
297.9
368.0
494.8
523.6
649.8
746.4
817.0
843.9
1096.6
1124.9
m/z

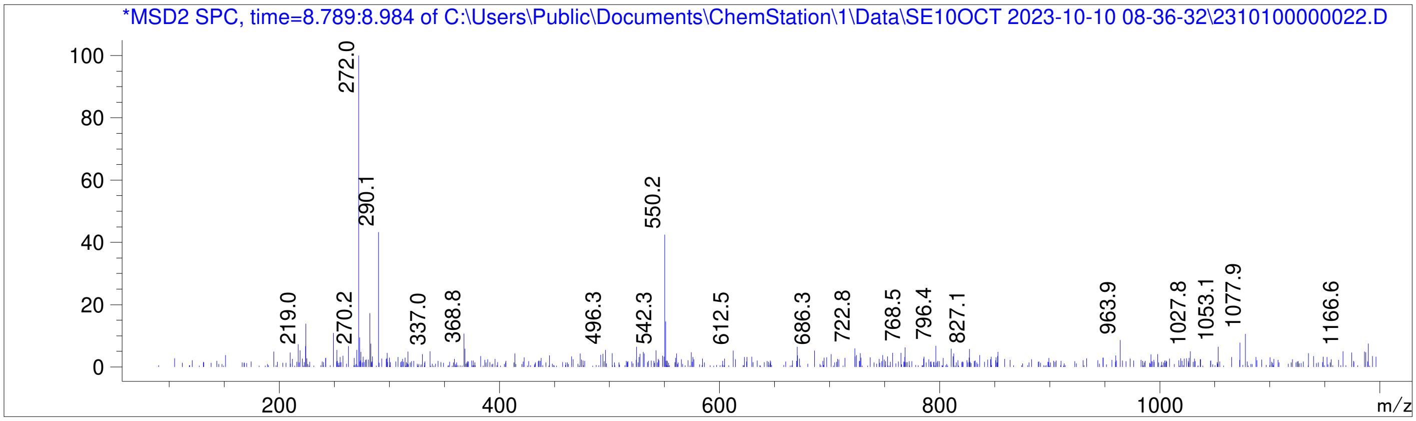
*MSD2 SPC, time=8.789:8.984 of C:\Users\Public\Documents\ChemStation\1\Data\SE10OCT 2023-10-10 08-36-32\2310100000022.D
272.0
290.1
550.2
219.0
270.2
337.0
368.8
496.3
542.3
612.5
686.3
722.8
768.5
796.4
827.1
963.9
1027.8
1053.1
1077.9
1166.6
m/z

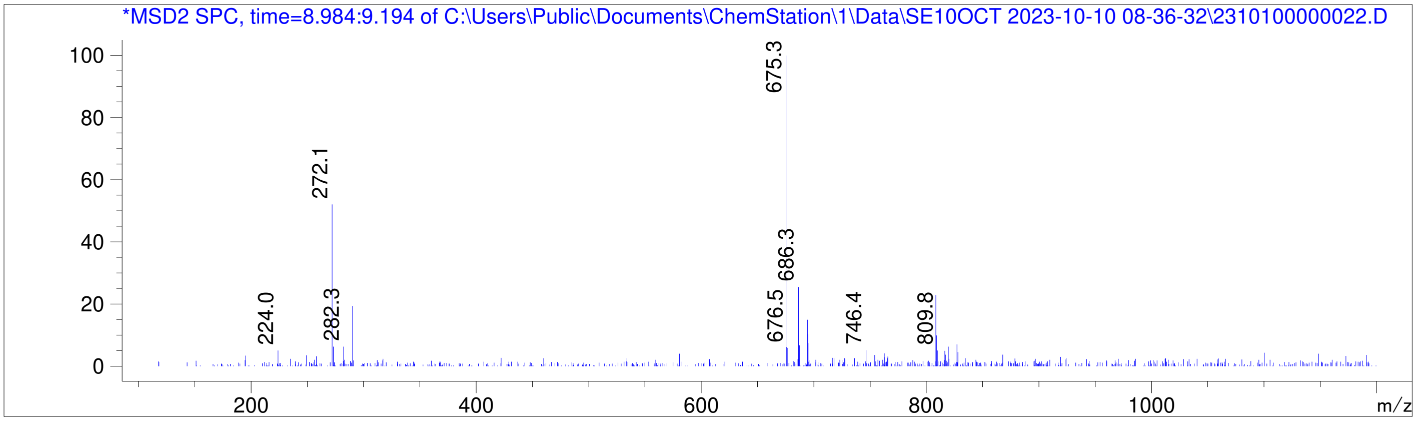
*MSD2 SPC, time=8.984:9.194 of C:\Users\Public\Documents\ChemStation\1\Data\SE10OCT 2023-10-10 08-36-32\2310100000022.D
675.3
272.1
686.3
224.0
282.3
676.5
746.4
809.8
m/z

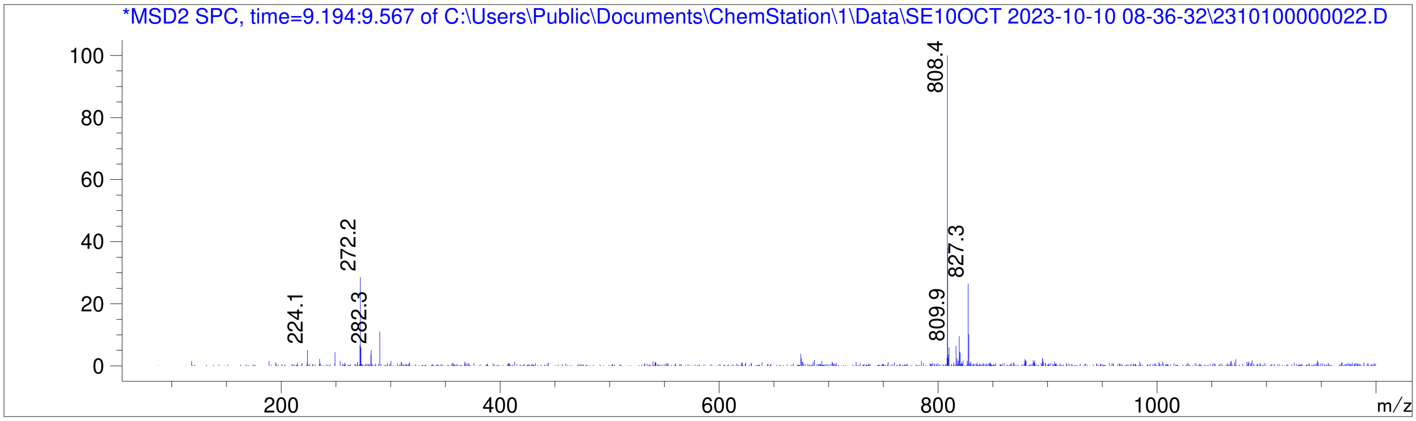
*MSD2 SPC, time=9.194:9.567 of C:\Users\Public\Documents\ChemStation\1\Data\SE10OCT 2023-10-10 08-36-32\2310100000022.D
808.4
272.2
827.3
224.1
282.3
809.9
m/z

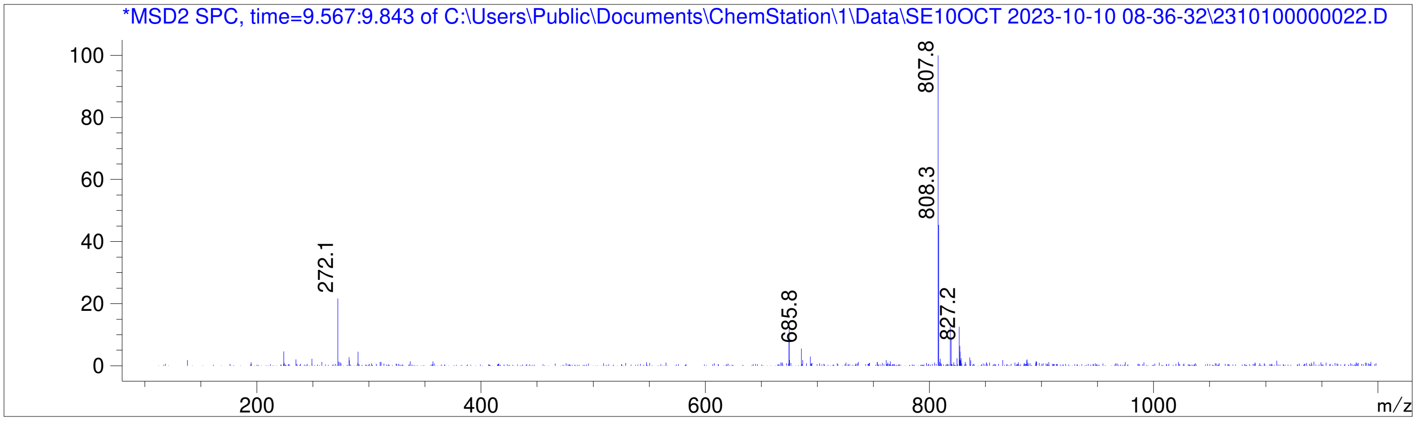
*MSD2 SPC, time=9.567:9.843 of C:\Users\Public\Documents\ChemStation\1\Data\SE10OCT 2023-10-10 08-36-32\2310100000022.D
807.8
808.3
272.1
685.8
827.2
m/z

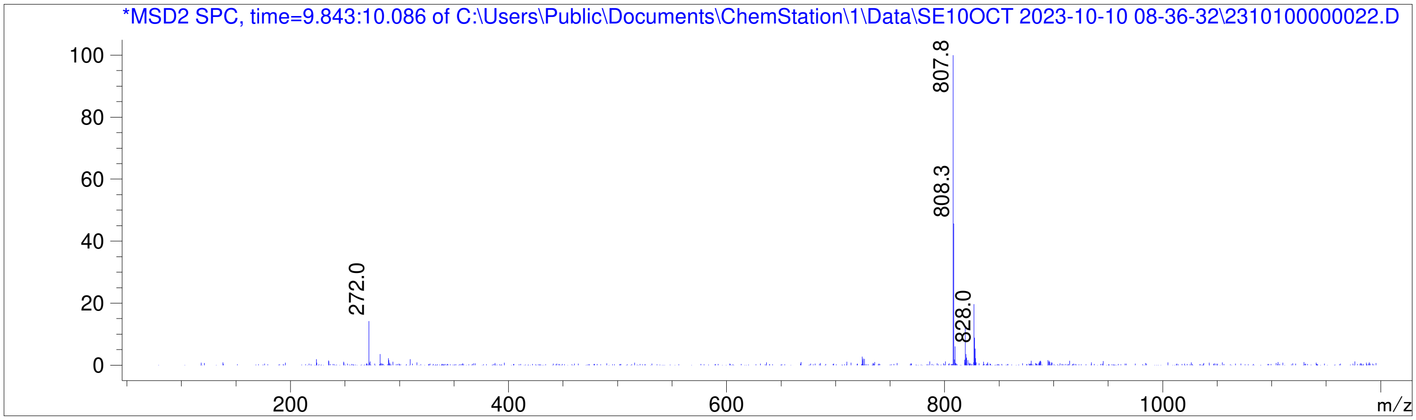
*MSD2 SPC, time=9.843:10.086 of C:\Users\Public\Documents\ChemStation\1\Data\SE10OCT 2023-10-10 08-36-32\2310100000022.D
807.8
808.3
272.0
828.0
m/z
