# Supplementary material for: Astrobiological implications of the stability and reactivity of peptide nucleic acid (PNA) in concentrated sulfuric acid
Source: Sci Adv. 2025 Mar 26;11(13):eadr0006. doi: 10.1126/sciadv.adr0006 (PMC11939054; doi:10.1126/sciadv.adr0006)
Supplement: Supplementary file 2 — Data S1 and S2 [file sciadv.adr0006_data_s1_and_s2.zip › Supplementary Dataset 1-LCMS DATA/LCMS PNA Hexamers A-T/LCMS T6 RT/1h/CPT22010446-19-D2.pdf]

Data -> C:\Users\Public\Documents\ChemStation\1\Data\SE10OCT 2023-10-10 08-36-32\ ->
Sample-> CPT22010446-19-D2

=====================================================================

Injection Date : Tue, 10. Oct. 2023

Seq Line : 24
Location : 17
Inj. Vol. : 2 µl

Acq. Method : C:\Users\Public\Documents\ChemStation\1\Data\SE10OCT 2023-10-10 08-36-32\22010446C LCMS-6#.M
Analysis Method : C:\Users\Public\Documents\ChemStation\1\Data\SE10OCT 2023-10-10 08-36-32\22010446C LCMS-6#.M (Sequence Method)
Waters XBridge BEH Amide (4.6 x 150 mm, 2.5 µm); PN# 186006726
Mobile Phase A: 20mM Ammonium Acetate (aq) pH 8.2
Mobile Phase B: AcN
Mobile Phase A / Mobile Phase B: 5/95 (0 min) --> (10 min) --> 60/40 (5 min); Flow: 1.0 ml/min; MSD1 = positive; MSD2 = negative

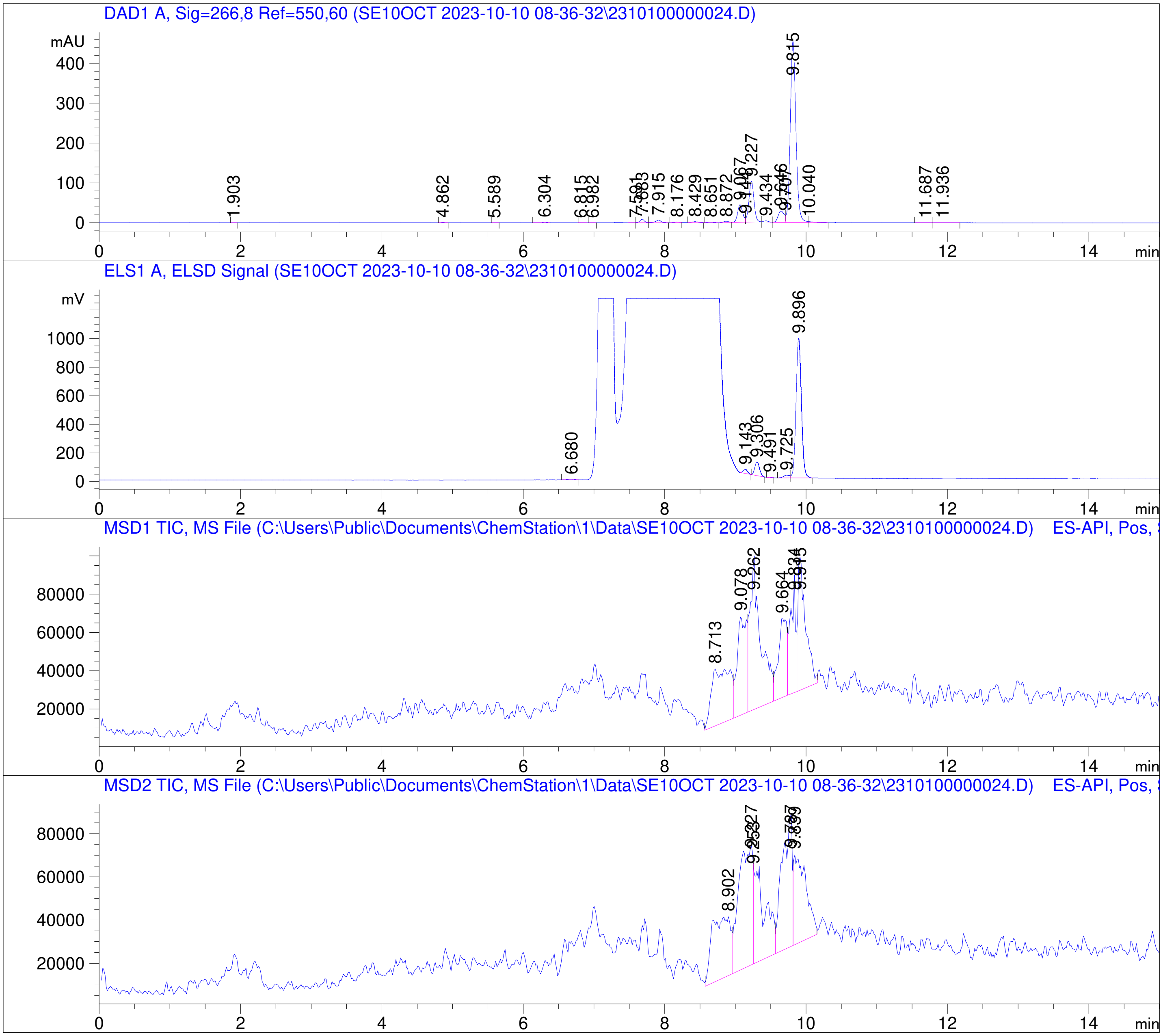

DAD1 A, Sig=266,8 Ref=550,60

| Peak # | Ret. Time [min] | Area [mV *s] | Area % |
|---|---|---|---|
| 1 | 1.903 | 0.368 | 0.010 |
| 2 | 4.862 | 2.198 | 0.062 |
| 3 | 5.589 | 0.600 | 0.017 |
| 4 | 6.304 | 5.552 | 0.156 |
| 5 | 6.815 | 0.152 | 0.004 |
| 6 | 6.982 | 0.301 | 0.008 |
| 7 | 7.591 | 0.721 | 0.020 |
| 8 | 7.683 | 36.404 | 1.023 |
| 9 | 7.915 | 33.325 | 0.936 |
| 10 | 8.176 | 4.990 | 0.140 |
| 11 | 8.429 | 11.643 | 0.327 |
| 12 | 8.651 | 6.588 | 0.185 |
| 13 | 8.872 | 13.164 | 0.370 |
| 14 | 9.067 | 215.792 | 6.064 |
| 15 | 9.144 | 1.183 | 0.033 |
| 16 | 9.227 | 501.767 | 14.101 |
| 17 | 9.434 | 11.464 | 0.322 |
| 18 | 9.646 | 164.716 | 4.629 |
| 19 | 9.707 | 1.716 | 0.048 |
| 20 | 9.815 | 2533.975 | 71.210 |
| 21 | 10.040 | 10.149 | 0.285 |
| 22 | 11.687 | 0.894 | 0.025 |
| 23 | 11.936 | 0.774 | 0.022 |

ELS1 A, ELSD Signal

| Peak # | Ret. Time [min] | Area [mV *s] | Area % |
|---|---|---|---|
| 1 | 6.680 | 33.258 | 0.564 |
| 2 | 9.143 | 124.319 | 2.110 |
| 3 | 9.306 | 448.461 | 7.611 |
| 4 | 9.491 | 6.066 | 0.103 |
| 5 | 9.725 | 109.876 | 1.865 |
| 6 | 9.896 | 5170.511 | 87.747 |

===========================================================================

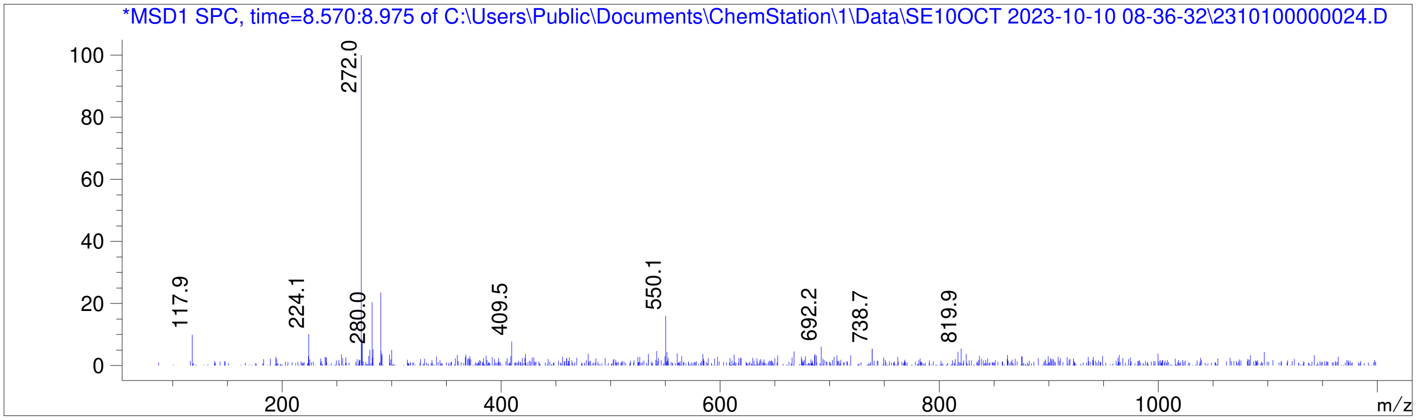
*MSD1 SPC, time=8.570:8.975 of C:\Users\Public\Documents\ChemStation\1\Data\SE10OCT 2023-10-10 08-36-32\2310100000024.D
100
80
60
40
20
0
272.0
117.9
224.1
280.0
409.5
550.1
692.2
738.7
819.9
200
400
600
800
1000
m/z

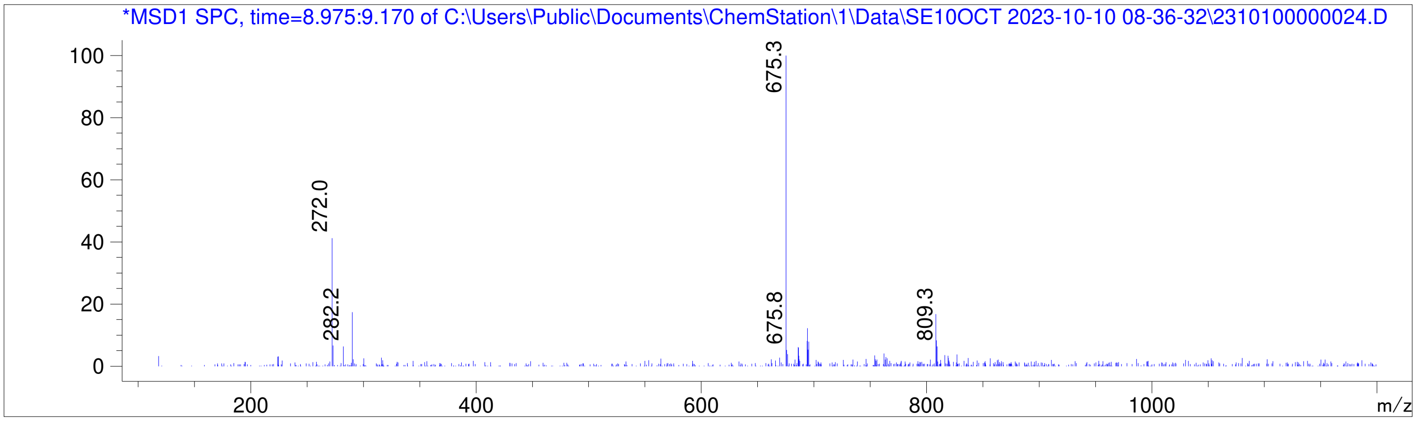
*MSD1 SPC, time=8.975:9.170 of C:\Users\Public\Documents\ChemStation\1\Data\SE10OCT 2023-10-10 08-36-32\2310100000024.D
100
80
60
40
20
0
675.3
272.0
282.2
675.8
809.3
200
400
600
800
1000
m/z

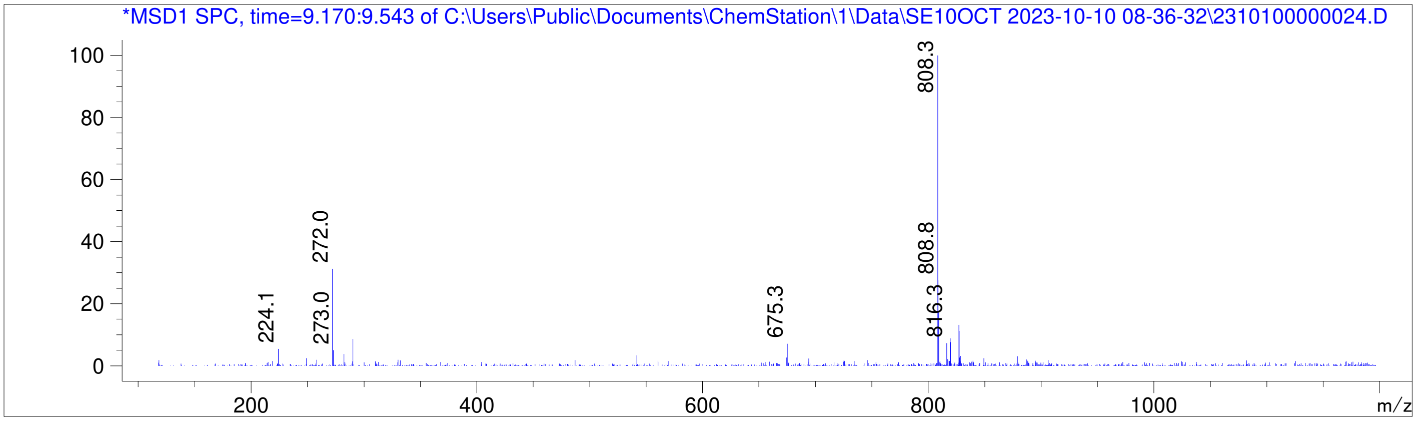
*MSD1 SPC, time=9.170:9.543 of C:\Users\Public\Documents\ChemStation\1\Data\SE10OCT 2023-10-10 08-36-32\2310100000024.D
100
80
60
40
20
0
808.3
272.0
808.8
224.1
273.0
675.3
816.3
200
400
600
800
1000
m/z

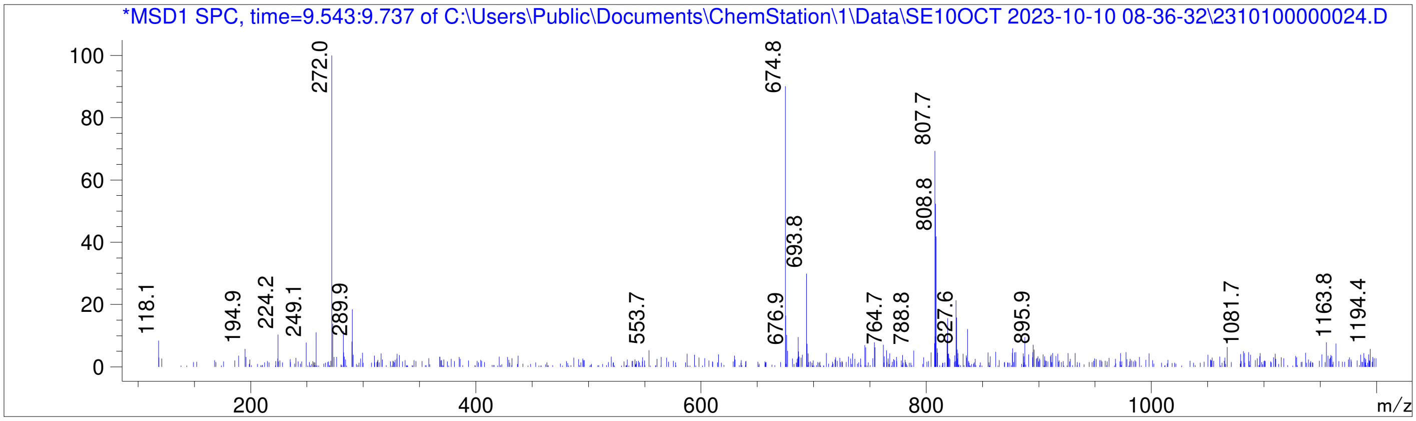
*MSD1 SPC, time=9.543:9.737 of C:\Users\Public\Documents\ChemStation\1\Data\SE10OCT 2023-10-10 08-36-32\2310100000024.D
100
80
60
40
20
0
272.0
674.8
807.7
808.8
693.8
118.1
194.9
224.2
249.1
289.9
553.7
676.9
764.7
788.8
827.6
895.9
1081.7
1163.8
1194.4
200
400
600
800
1000
m/z

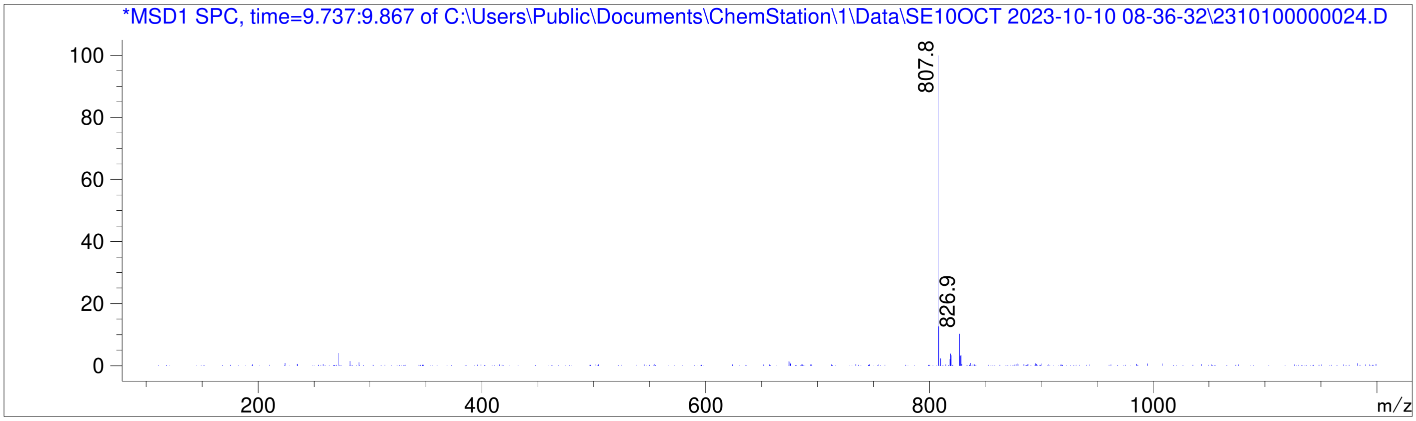
*MSD1 SPC, time=9.737:9.867 of C:\Users\Public\Documents\ChemStation\1\Data\SE10OCT 2023-10-10 08-36-32\2310100000024.D
100
80
60
40
20
0
807.8
826.9
200
400
600
800
1000
m/z

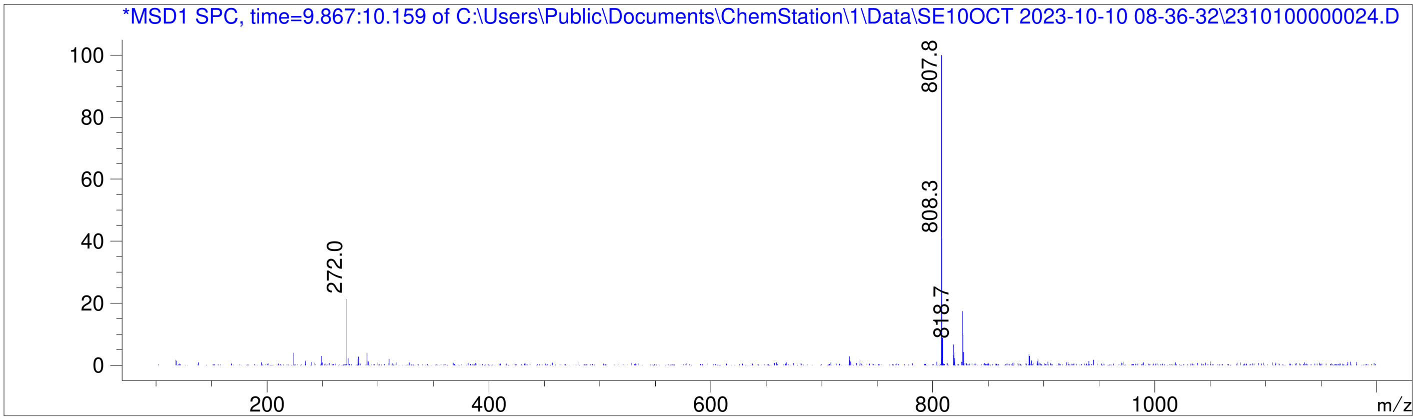
*MSD1 SPC, time=9.867:10.159 of C:\Users\Public\Documents\ChemStation\1\Data\SE10OCT 2023-10-10 08-36-32\2310100000024.D
100
80
60
40
20
0
807.8
808.3
272.0
818.7
200
400
600
800
1000
m/z

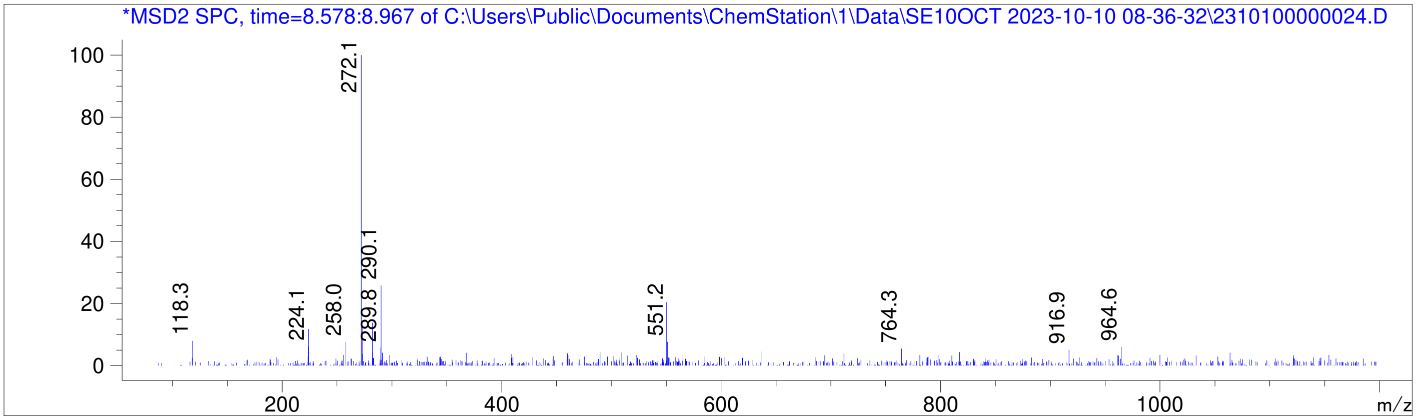
*MSD2 SPC, time=8.578:8.967 of C:\Users\Public\Documents\ChemStation\1\Data\SE10OCT 2023-10-10 08-36-32\2310100000024.D
272.1
290.1
118.3
224.1
258.0
289.8
551.2
764.3
916.9
964.6
m/z

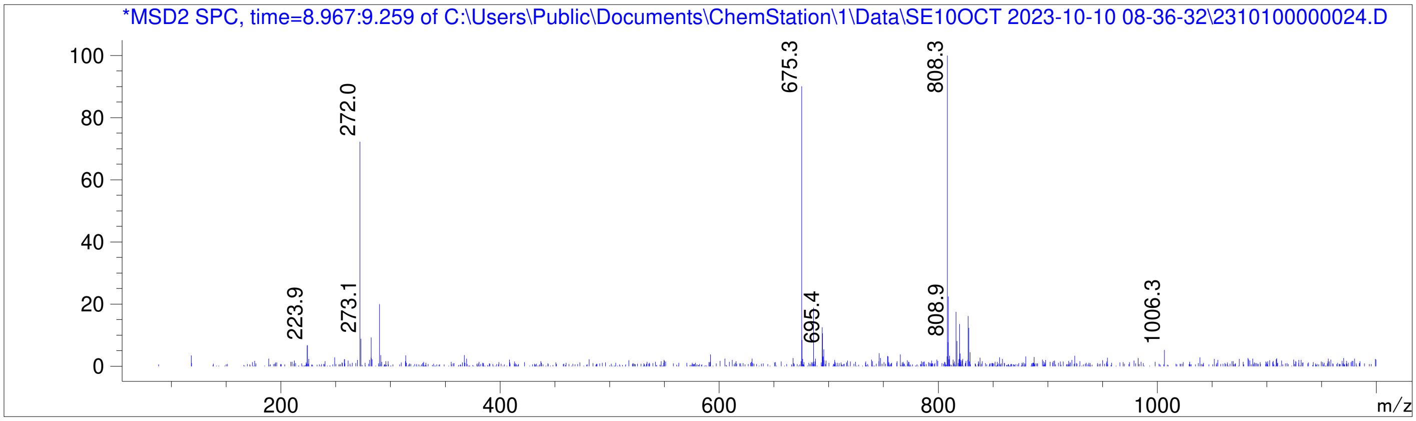
*MSD2 SPC, time=8.967:9.259 of C:\Users\Public\Documents\ChemStation\1\Data\SE10OCT 2023-10-10 08-36-32\2310100000024.D
675.3
808.3
272.0
223.9
273.1
695.4
808.9
1006.3
m/z

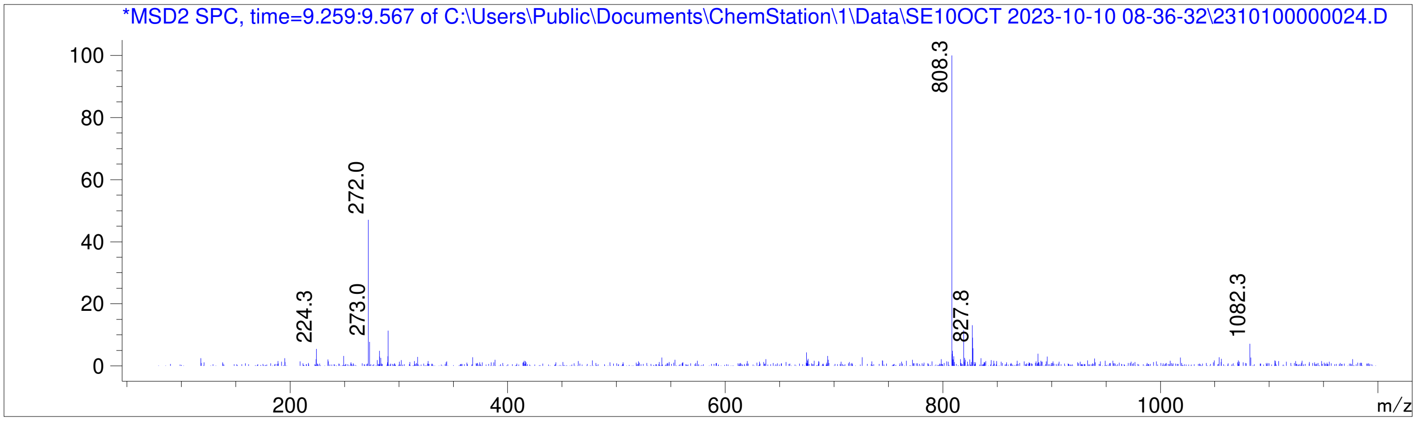
*MSD2 SPC, time=9.259:9.567 of C:\Users\Public\Documents\ChemStation\1\Data\SE10OCT 2023-10-10 08-36-32\2310100000024.D
808.3
272.0
224.3
273.0
827.8
1082.3
m/z

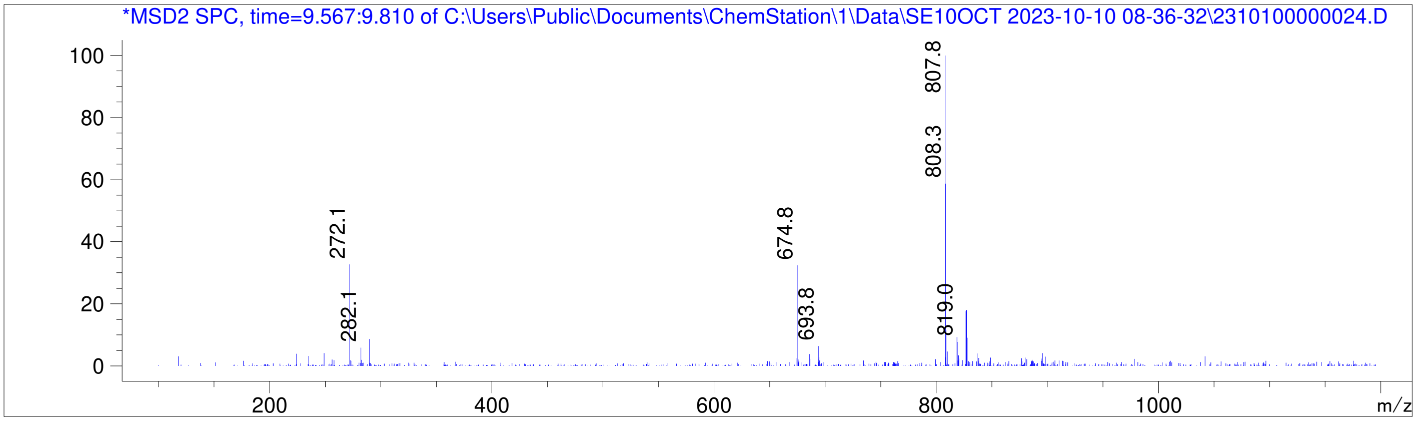
*MSD2 SPC, time=9.567:9.810 of C:\Users\Public\Documents\ChemStation\1\Data\SE10OCT 2023-10-10 08-36-32\2310100000024.D
807.8
808.3
272.1
674.8
282.1
693.8
819.0
m/z

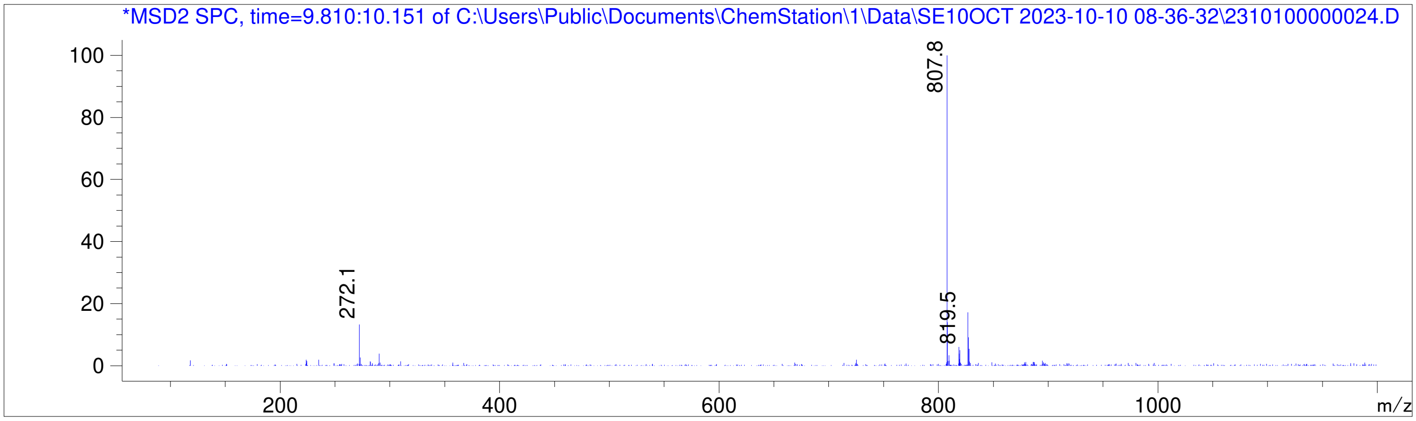
*MSD2 SPC, time=9.810:10.151 of C:\Users\Public\Documents\ChemStation\1\Data\SE10OCT 2023-10-10 08-36-32\2310100000024.D
807.8
272.1
819.5
m/z
