# Supplementary material for: Astrobiological implications of the stability and reactivity of peptide nucleic acid (PNA) in concentrated sulfuric acid
Source: Sci Adv. 2025 Mar 26;11(13):eadr0006. doi: 10.1126/sciadv.adr0006 (PMC11939054; doi:10.1126/sciadv.adr0006)
Supplement: Supplementary file 2 — Data S1 and S2 [file sciadv.adr0006_data_s1_and_s2.zip › Supplementary Dataset 1-LCMS DATA/LCMS PNA Hexamers A-T/LCMS T6 RT/1h/CPT22010446-19-D3.pdf]

===

Injection Date : Mon, 2. Oct. 2023

Seq Line : 4
Location : 43
Inj. Vol. : 2 µl

Acq. Method : C:\Users\Public\Documents\ChemStation\1\Data\SE02OCT 2023-10-02 15-52-16\22010446C LCMS-6#.M
Analysis Method : C:\Users\Public\Documents\ChemStation\1\Data\SE02OCT 2023-10-02 15-52-16\22010446C LCMS-6#.M (Sequence Method)
Waters XBridge BEH Amide (4.6 x 150 mm, 2.5 µm); PN# 186006726
Mobile Phase A: 20mM Ammonium Acetate (aq) pH 8.2
Mobile Phase B: AcN
Mobile Phase A / Mobile Phase B: 5/95 (0 min) --> (10 min) --> 60/40 (5 min); Flow: 1.0 ml/min; MSD1 = positive; MSD2 = negative

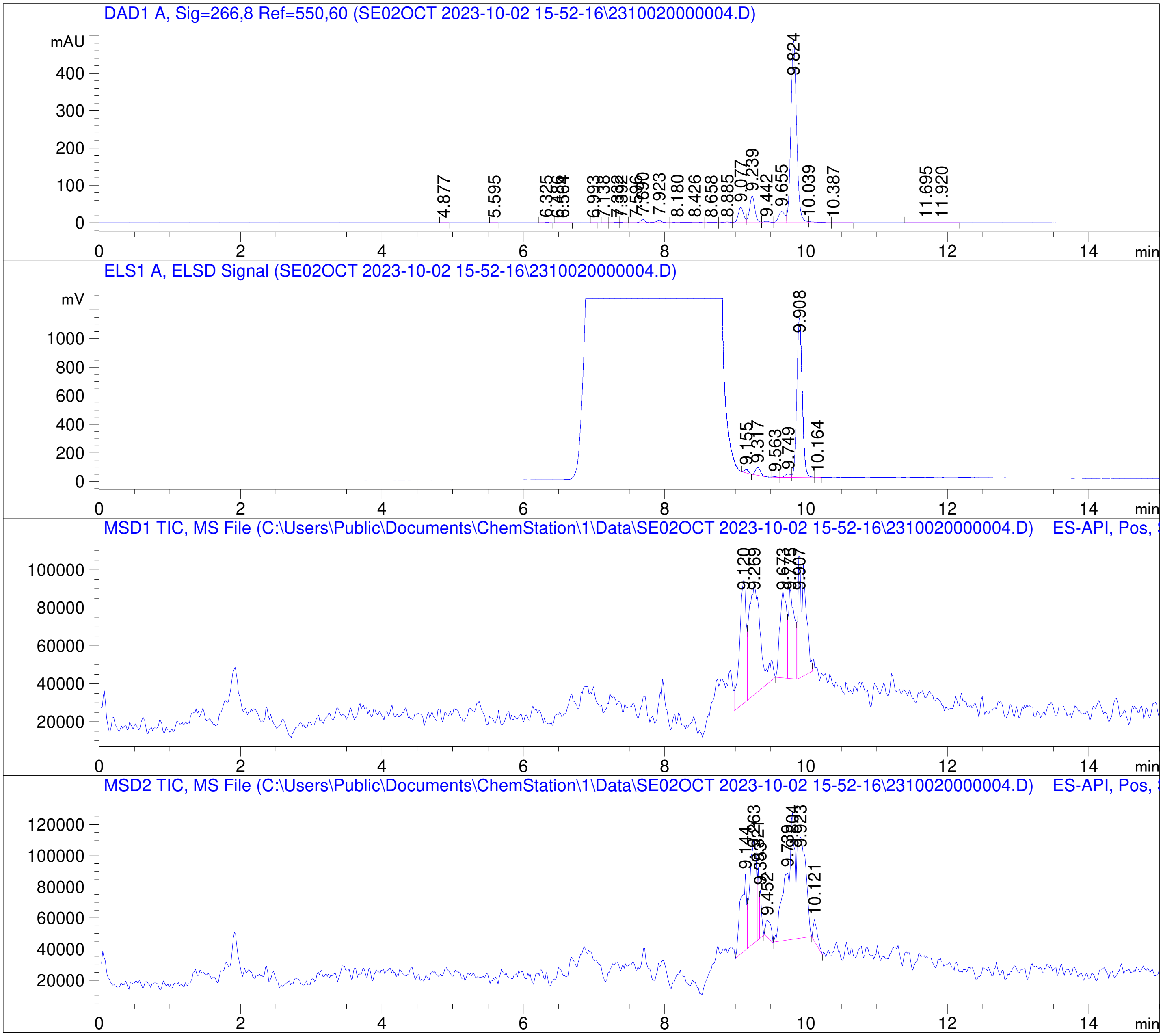

DAD1 A, Sig=266,8 Ref=550,60

Peak #	Ret. Time [min]	Area [mV *s]	Area %
1	4.877	1.165	0.031
2	5.595	0.638	0.017
3	6.325	2.154	0.058
4	6.486	0.151	0.004
5	6.564	0.573	0.015
6	6.993	0.201	0.005
7	7.138	0.307	0.008
8	7.332	3.059	0.083
9	7.392	1.883	0.051
10	7.596	0.786	0.021
11	7.690	36.462	0.984
12	7.923	36.074	0.974
13	8.180	12.044	0.325
14	8.426	13.536	0.365
15	8.658	6.228	0.168
16	8.885	12.793	0.345
17	9.077	217.665	5.876
18	9.239	369.969	9.987
19	9.442	23.184	0.626
20	9.655	181.118	4.889
21	9.824	2757.294	74.432
22	10.039	21.341	0.576
23	10.387	3.419	0.092
24	11.695	1.251	0.034
25	11.920	1.133	0.031

ELS1 A, ELSD Signal

Peak #	Ret. Time [min]	Area [mV *s]	Area %
1	9.155	94.869	1.461
2	9.317	263.256	4.053
3	9.563	19.883	0.306
4	9.749	133.990	2.063
5	9.908	5977.538	92.030
6	10.164	5.649	0.087

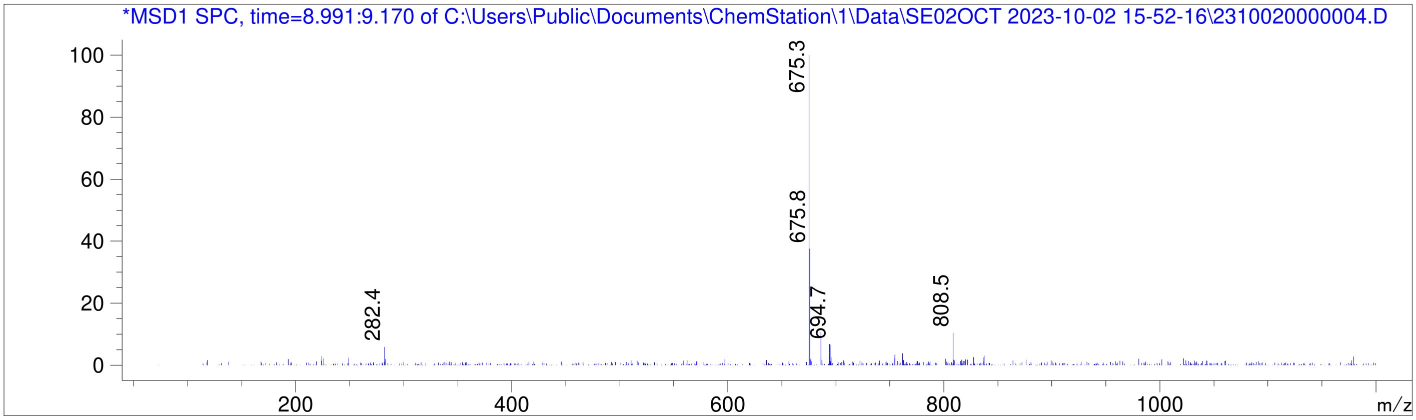
*MSD1 SPC, time=8.991:9.170 of C:\Users\Public\Documents\ChemStation\1\Data\SE02OCT 2023-10-02 15-52-16\2310020000004.D
675.3
675.8
694.7
808.5
282.4
m/z

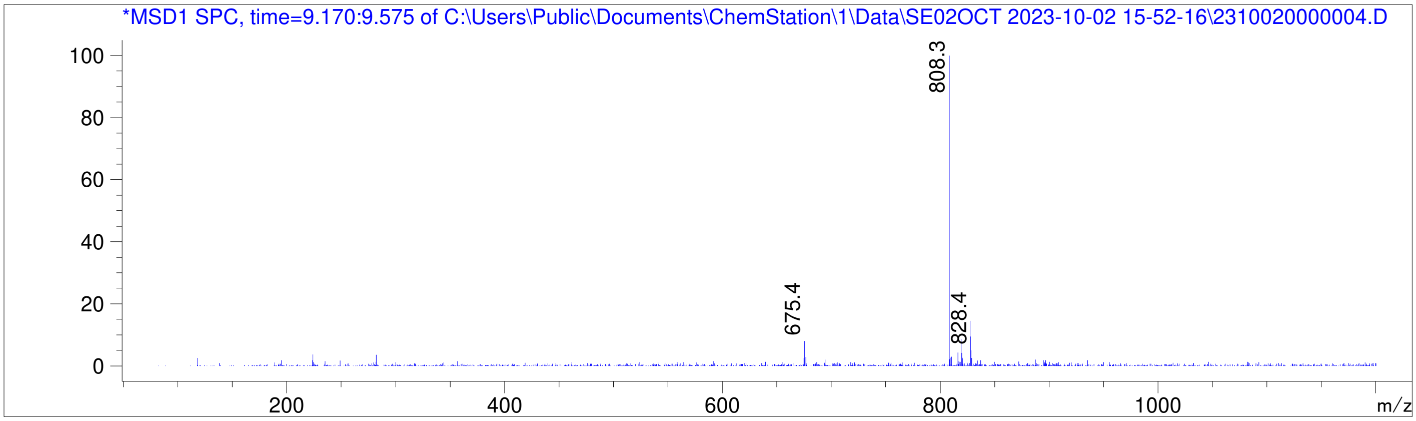
*MSD1 SPC, time=9.170:9.575 of C:\Users\Public\Documents\ChemStation\1\Data\SE02OCT 2023-10-02 15-52-16\2310020000004.D
808.3
675.4
828.4
m/z

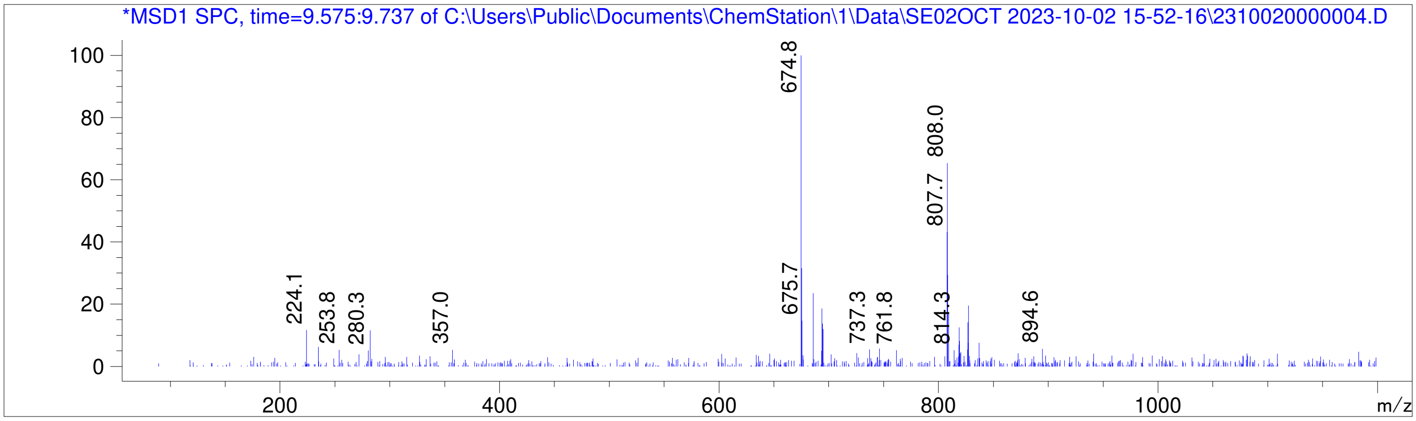
*MSD1 SPC, time=9.575:9.737 of C:\Users\Public\Documents\ChemStation\1\Data\SE02OCT 2023-10-02 15-52-16\2310020000004.D
674.8
808.0
807.7
675.7
224.1
253.8
280.3
357.0
737.3
761.8
814.3
894.6
m/z

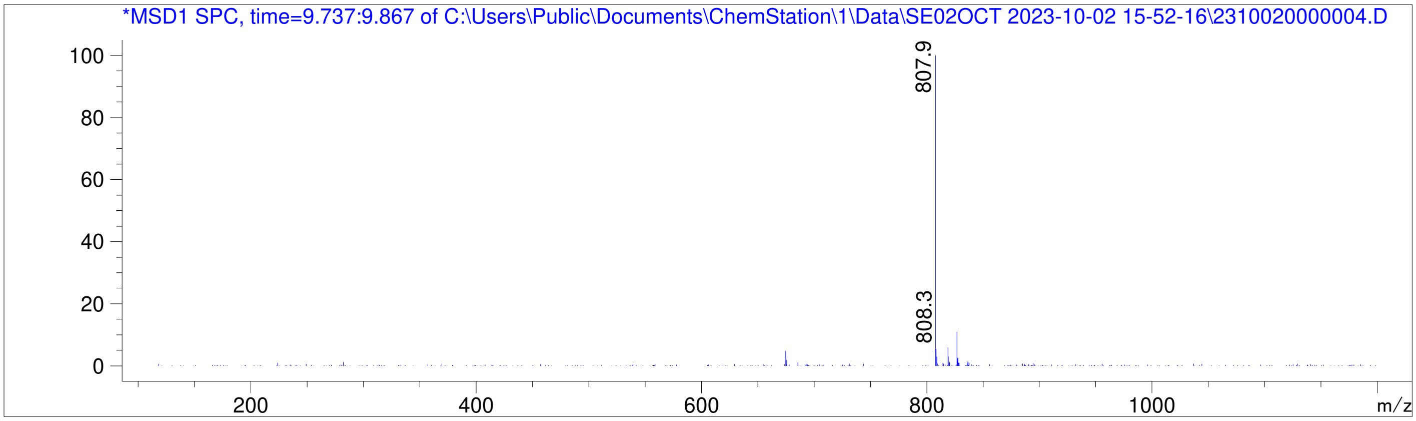
*MSD1 SPC, time=9.737:9.867 of C:\Users\Public\Documents\ChemStation\1\Data\SE02OCT 2023-10-02 15-52-16\2310020000004.D
807.9
808.3
100
80
60
40
20
0
200
400
600
800
1000
m/z

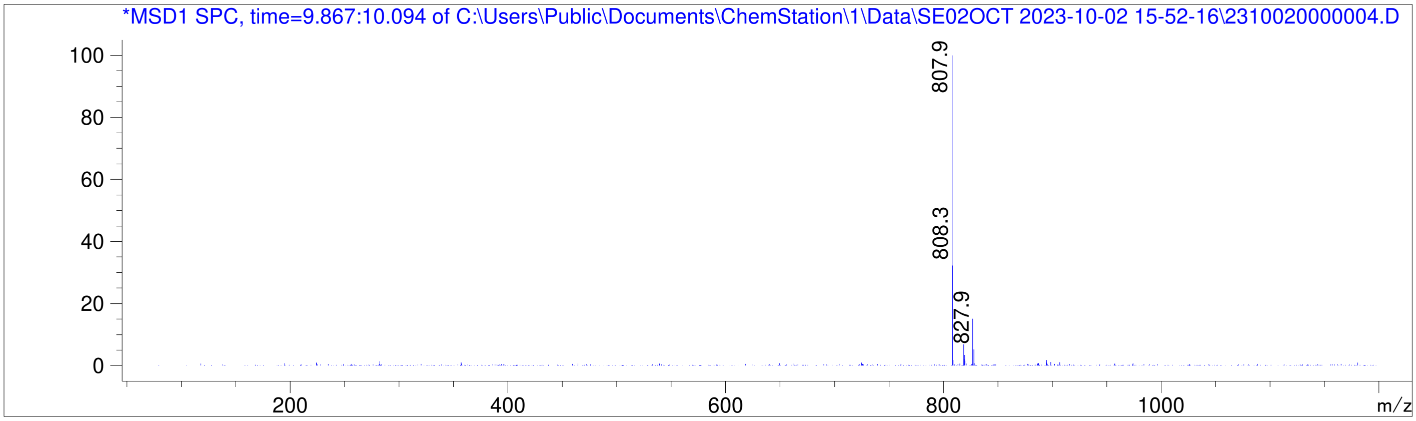
*MSD1 SPC, time=9.867:10.094 of C:\Users\Public\Documents\ChemStation\1\Data\SE02OCT 2023-10-02 15-52-16\2310020000004.D
807.9
808.3
827.9
100
80
60
40
20
0
200
400
600
800
1000
m/z

==

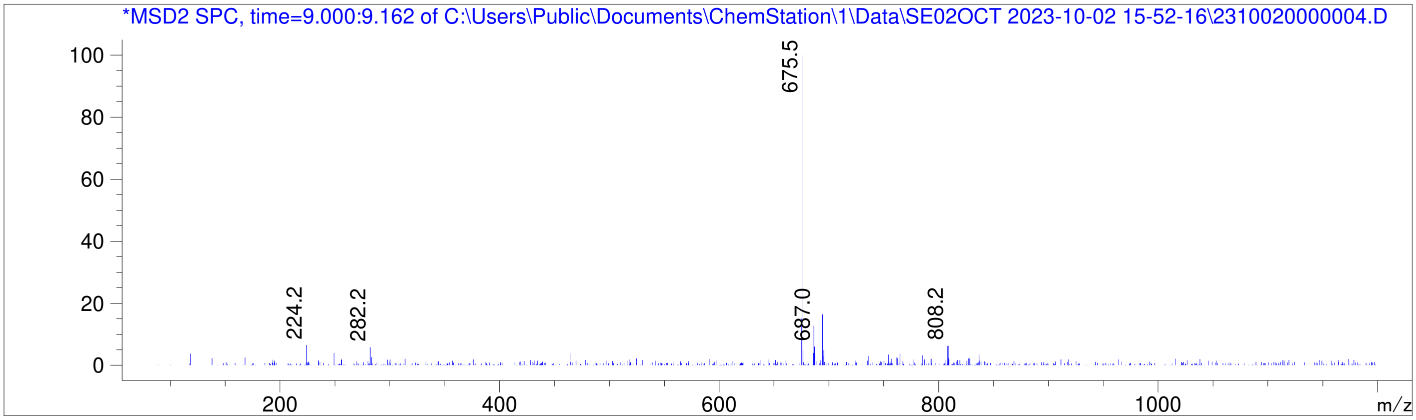
*MSD2 SPC, time=9.000:9.162 of C:\Users\Public\Documents\ChemStation\1\Data\SE02OCT 2023-10-02 15-52-16\2310020000004.D
675.5
224.2
282.2
687.0
808.2
m/z

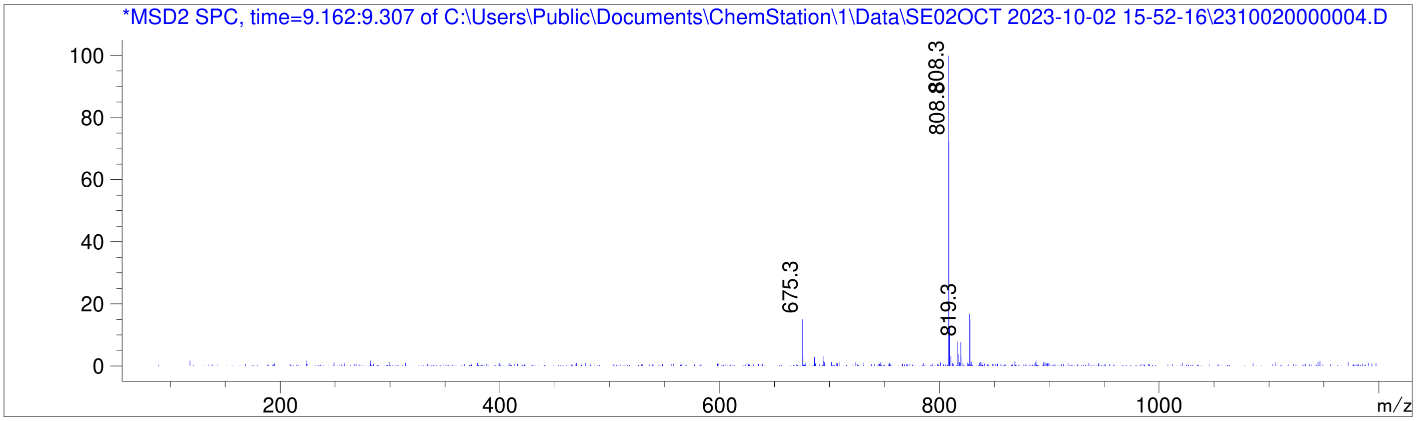
*MSD2 SPC, time=9.162:9.307 of C:\Users\Public\Documents\ChemStation\1\Data\SE02OCT 2023-10-02 15-52-16\2310020000004.D
808.3
675.3
819.3
m/z

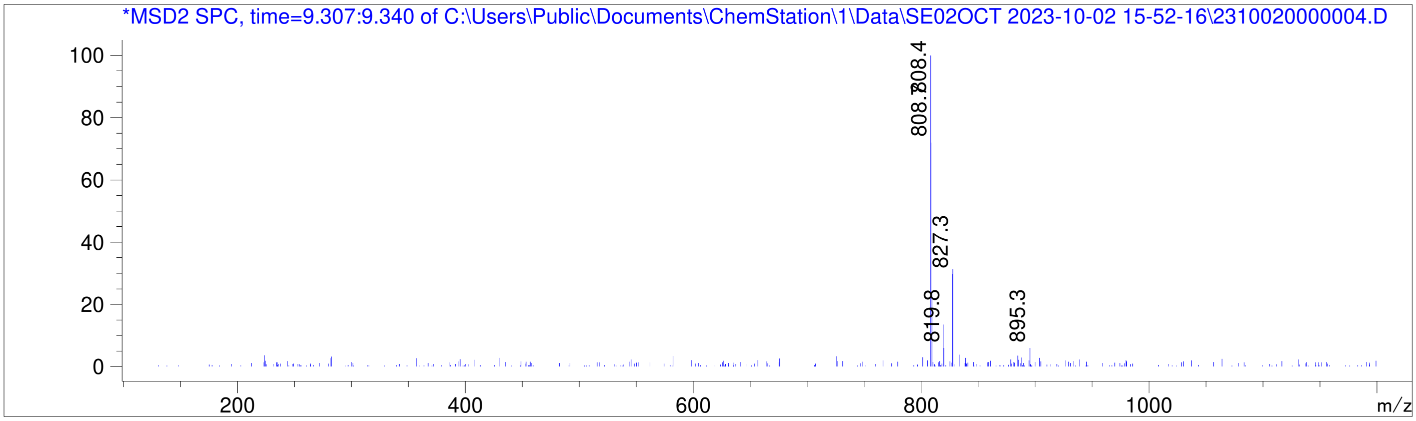
*MSD2 SPC, time=9.307:9.340 of C:\Users\Public\Documents\ChemStation\1\Data\SE02OCT 2023-10-02 15-52-16\2310020000004.D
808.4
827.3
819.8
895.3
m/z

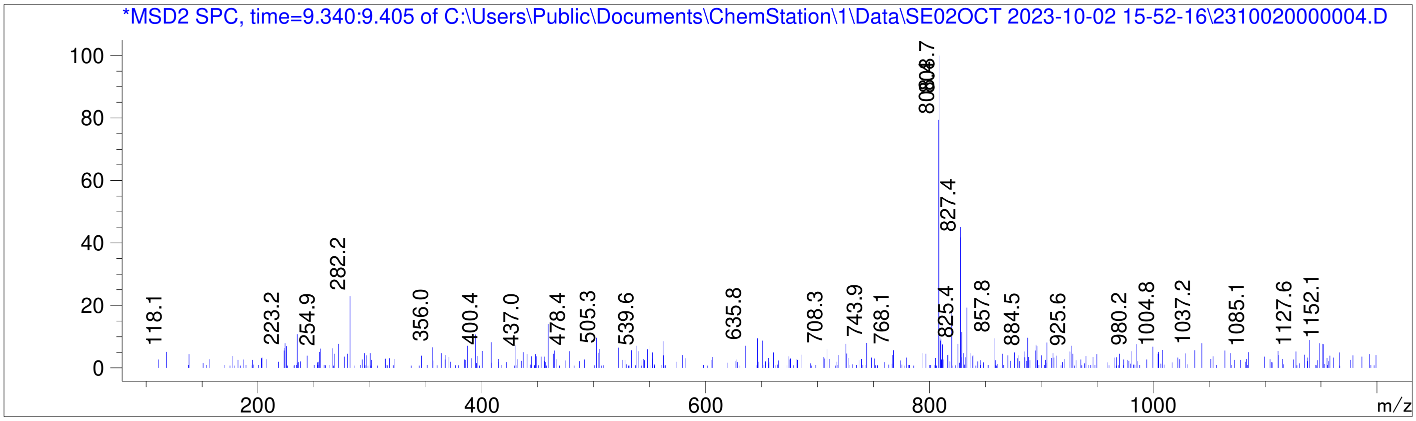
*MSD2 SPC, time=9.340:9.405 of C:\Users\Public\Documents\ChemStation\1\Data\SE02OCT 2023-10-02 15-52-16\2310020000004.D
118.1
223.2
254.9
282.2
356.0
400.4
437.0
478.4
505.3
539.6
635.8
708.3
743.9
768.1
825.4
827.4
857.8
884.5
925.6
980.2
1004.8
1037.2
1085.1
1127.6
1152.1
m/z

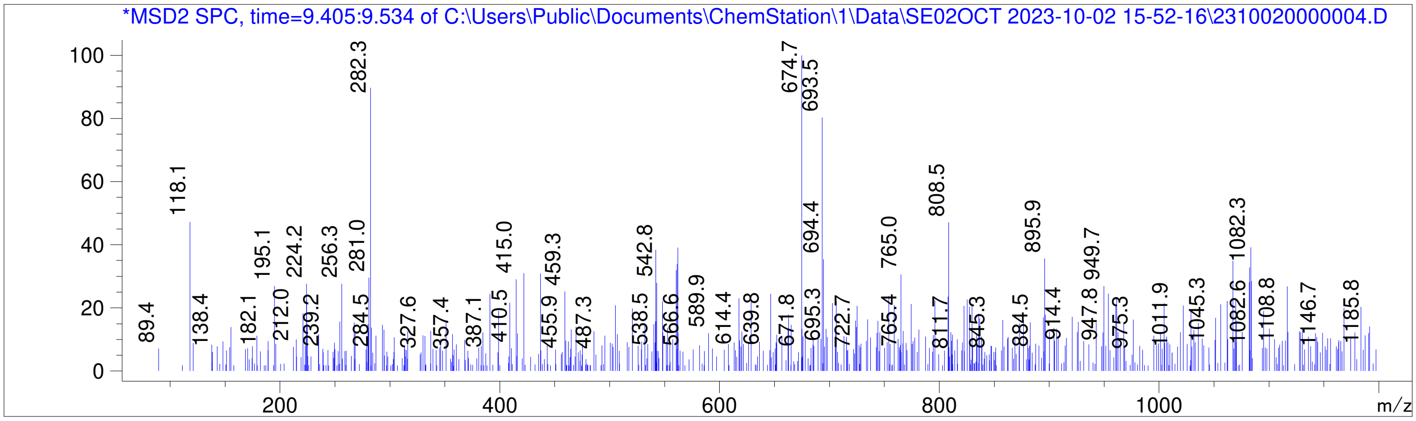
*MSD2 SPC, time=9.405:9.534 of C:\Users\Public\Documents\ChemStation\1\Data\SE02OCT 2023-10-02 15-52-16\2310020000004.D
89.4
118.1
138.4
182.1
195.1
212.0
224.2
239.2
256.3
281.0
282.3
284.5
327.6
357.4
387.1
410.5
415.0
455.9
459.3
487.3
538.5
542.8
566.6
589.9
614.4
639.8
671.8
674.7
693.5
694.4
695.3
722.7
765.0
765.4
808.5
811.7
845.3
884.5
895.9
914.4
947.8
949.7
975.3
1011.9
1045.3
1082.3
1082.6
1108.8
1146.7
1185.8
m/z

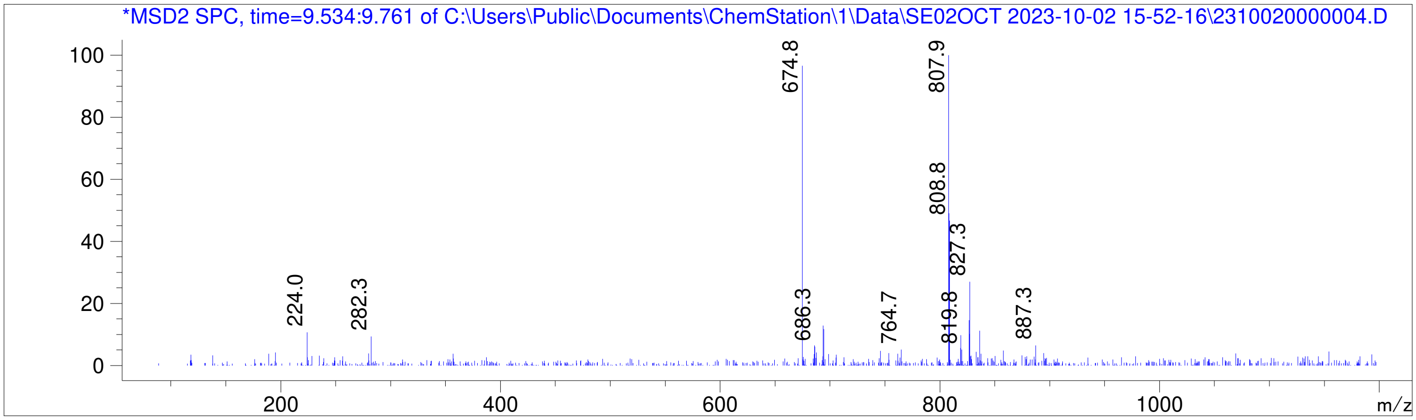
*MSD2 SPC, time=9.534:9.761 of C:\Users\Public\Documents\ChemStation\1\Data\SE02OCT 2023-10-02 15-52-16\2310020000004.D
224.0
282.3
674.8
686.3
764.7
807.9
808.8
819.8
827.3
887.3
m/z

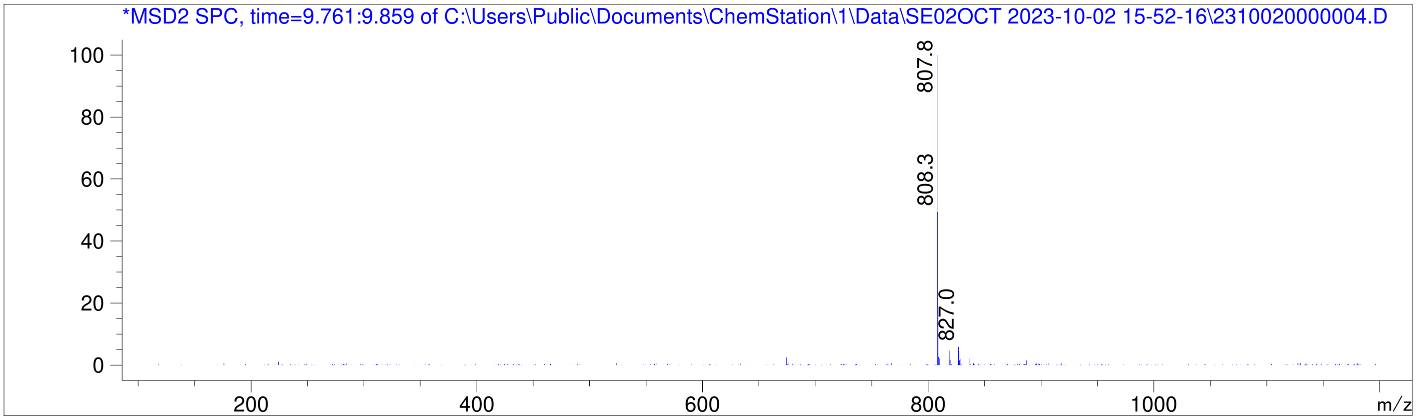
*MSD2 SPC, time=9.761:9.859 of C:\Users\Public\Documents\ChemStation\1\Data\SE02OCT 2023-10-02 15-52-16\2310020000004.D
807.8
808.3
827.0
m/z

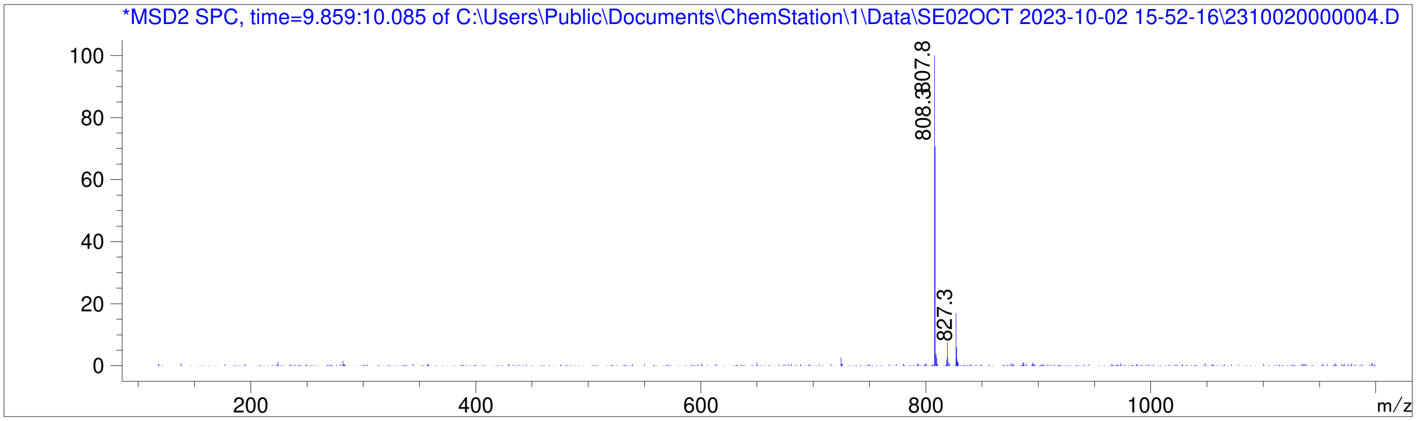
*MSD2 SPC, time=9.859:10.085 of C:\Users\Public\Documents\ChemStation\1\Data\SE02OCT 2023-10-02 15-52-16\2310020000004.D
807.8
808.3
827.3
m/z

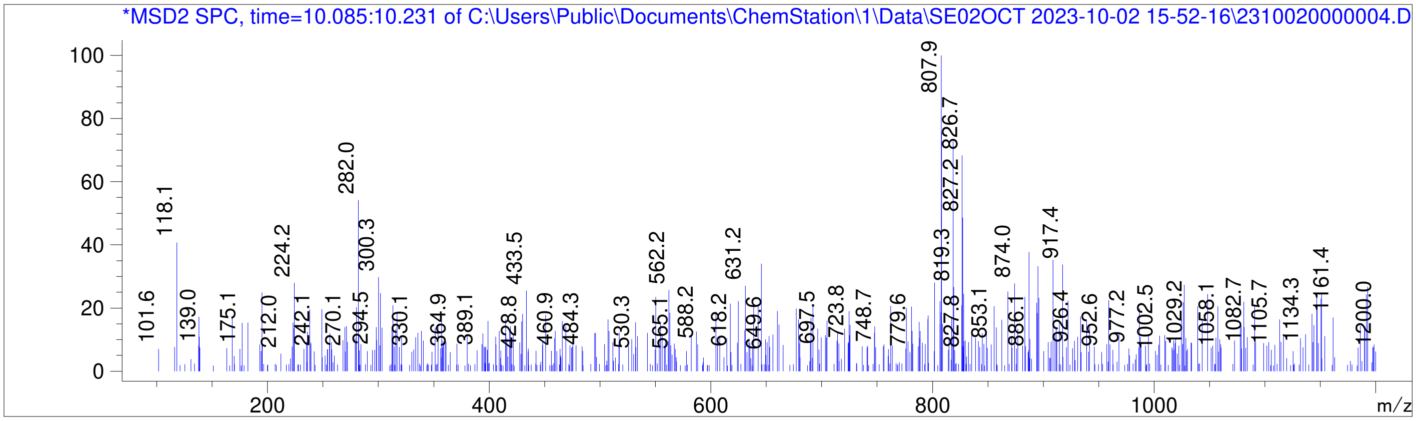
*MSD2 SPC, time=10.085:10.231 of C:\Users\Public\Documents\ChemStation\1\Data\SE02OCT 2023-10-02 15-52-16\2310020000004.D
807.9
826.7
827.2
282.0
118.1
300.3
224.2
433.5
562.2
631.2
874.0
917.4
819.3
1161.4
m/z
