# Supplementary material for: Astrobiological implications of the stability and reactivity of peptide nucleic acid (PNA) in concentrated sulfuric acid
Source: Sci Adv. 2025 Mar 26;11(13):eadr0006. doi: 10.1126/sciadv.adr0006 (PMC11939054; doi:10.1126/sciadv.adr0006)
Supplement: Supplementary file 2 — Data S1 and S2 [file sciadv.adr0006_data_s1_and_s2.zip › Supplementary Dataset 1-LCMS DATA/LCMS PNA Hexamers A-T/LCMS T6 RT/24h/CPT22010046-19-D3-24h.pdf]

Data -> C:\Users\Public\Documents\ChemStation\1\Data\SE03OCT 2023-10-03 09-25-28\ ->
Sample-> CPT22010046-19-D3-24h

===

Injection Date : Tue, 3. Oct. 2023

Seq Line : 8
Location : 58
Inj. Vol. : 2 µl

Acq. Method : C:\Users\Public\Documents\ChemStation\1\Data\SE03OCT 2023-10-03 09-25-28\22010446C LCMS-6#.M

Analysis Method : C:\Users\Public\Documents\ChemStation\1\Data\SE03OCT 2023-10-03 09-25-28\22010446C LCMS-6#.M (Sequence Method)

Waters XBridge BEH Amide (4.6 x 150 mm, 2.5 µm); PN# 186006726

Mobile Phase A: 20mM Ammonium Acetate (aq) pH 8.2

Mobile Phase B: AcN

Mobile Phase A / Mobile Phase B: 5/95 (0 min) --> (10 min) --> 60/40 (5 min); Flow: 1.0 ml/min; MSD1 = positive; MSD2 = negative

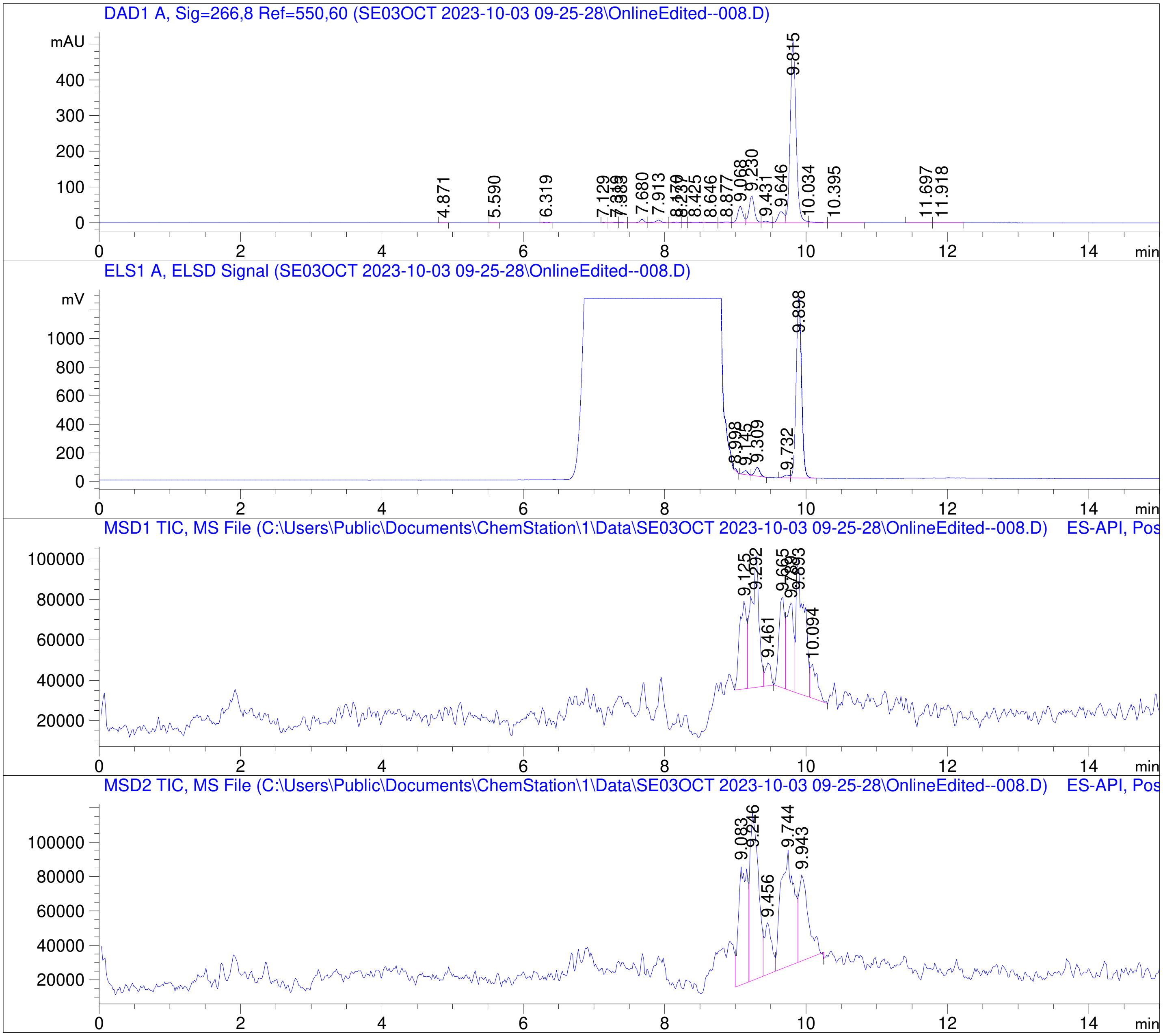

Sample-> CPT22010046-19-D3-24h

DAD1 A, Sig=266,8 Ref=550,60

Peak #	Ret. Time [min]	Area [mV *s]	Area %
1	4.871	1.057	0.027
2	5.590	0.710	0.018
3	6.319	5.592	0.143
4	7.129	1.593	0.041
5	7.319	3.433	0.088
6	7.383	3.520	0.090
7	7.680	40.917	1.044
8	7.913	40.345	1.030
9	8.170	11.218	0.286
10	8.237	3.400	0.087
11	8.425	16.495	0.421
12	8.646	9.475	0.242
13	8.877	17.012	0.434
14	9.068	235.553	6.011
15	9.230	387.654	9.892
16	9.431	25.928	0.662
17	9.646	186.195	4.751
18	9.815	2900.261	74.007
19	10.034	19.735	0.504
20	10.395	6.416	0.164
21	11.697	1.206	0.031
22	11.918	1.211	0.031

ELS1 A, ELSD Signal

Peak #	Ret. Time [min]	Area [mV *s]	Area %
1	8.998	34.149	0.467
2	9.145	133.482	1.826
3	9.309	304.879	4.170
4	9.732	111.159	1.520
5	9.898	6728.341	92.018

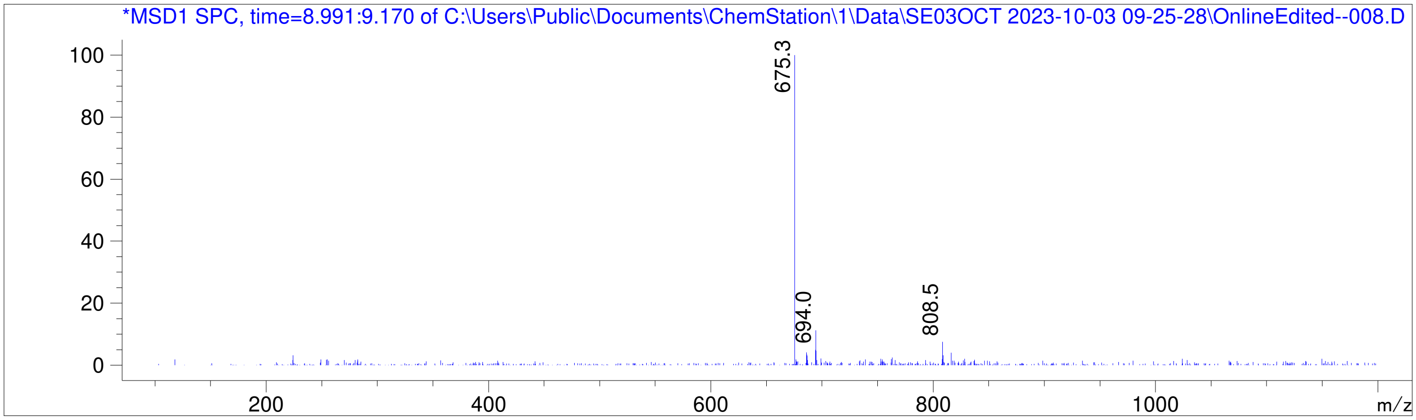
*MSD1 SPC, time=8.991:9.170 of C:\Users\Public\Documents\ChemStation\1\Data\SE03OCT 2023-10-03 09-25-28\OnlineEdited--008.D
675.3
694.0
808.5
m/z

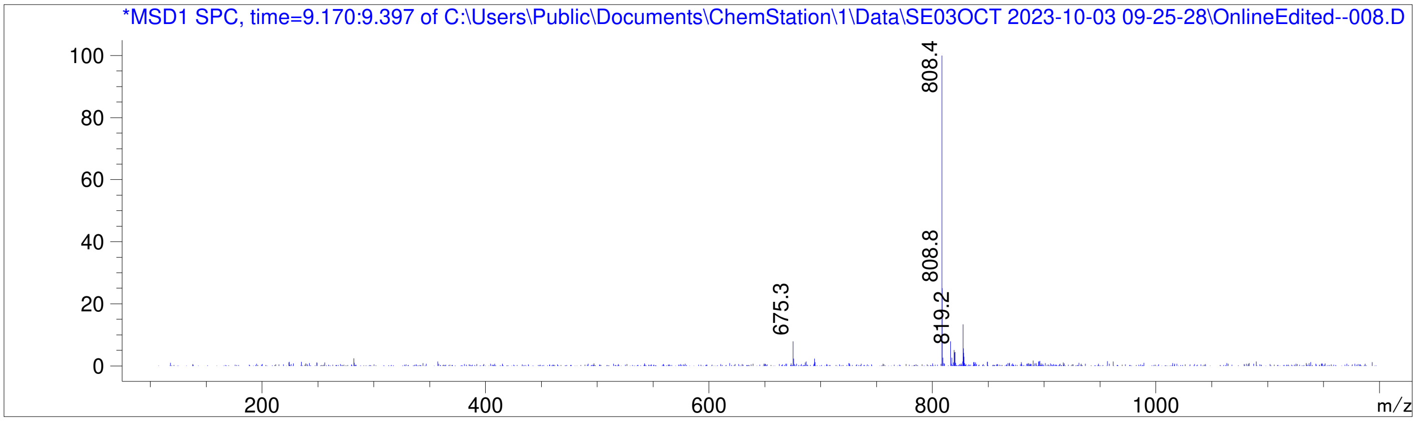
*MSD1 SPC, time=9.170:9.397 of C:\Users\Public\Documents\ChemStation\1\Data\SE03OCT 2023-10-03 09-25-28\OnlineEdited--008.D
808.4
808.8
819.2
675.3
m/z

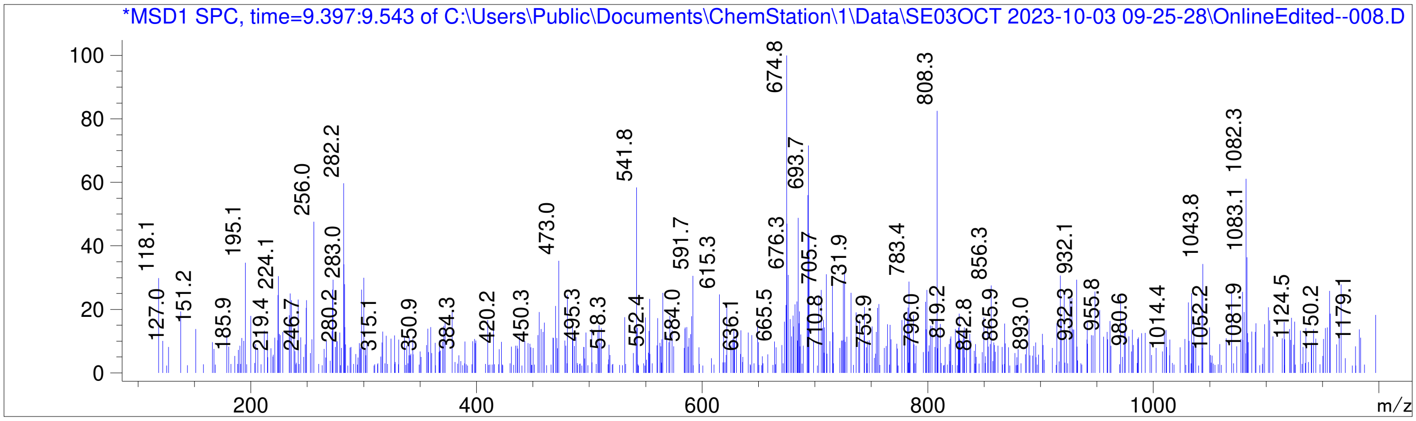
*MSD1 SPC, time=9.397:9.543 of C:\Users\Public\Documents\ChemStation\1\Data\SE03OCT 2023-10-03 09-25-28\OnlineEdited--008.D
674.8
808.3
1082.3
693.7
282.2
541.8
256.0
1083.1
1043.8
195.1
473.0
591.7
615.3
676.3
705.7
731.9
783.4
856.3
932.1
118.1
224.1
m/z

==

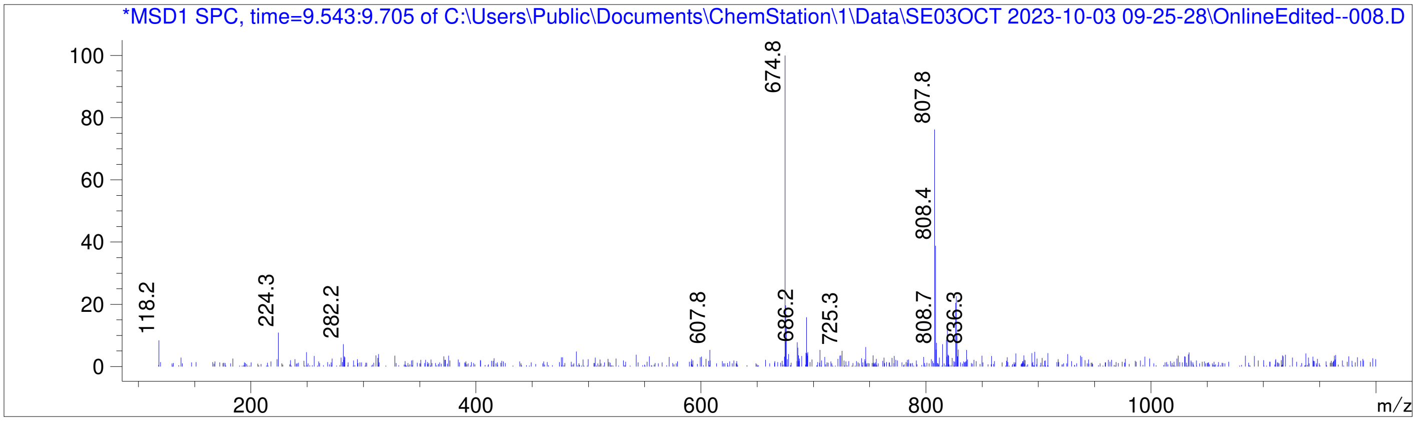
*MSD1 SPC, time=9.543:9.705 of C:\Users\Public\Documents\ChemStation\1\Data\SE03OCT 2023-10-03 09-25-28\OnlineEdited--008.D
674.8
807.8
808.4
118.2
224.3
282.2
607.8
686.2
725.3
808.7
836.3
m/z

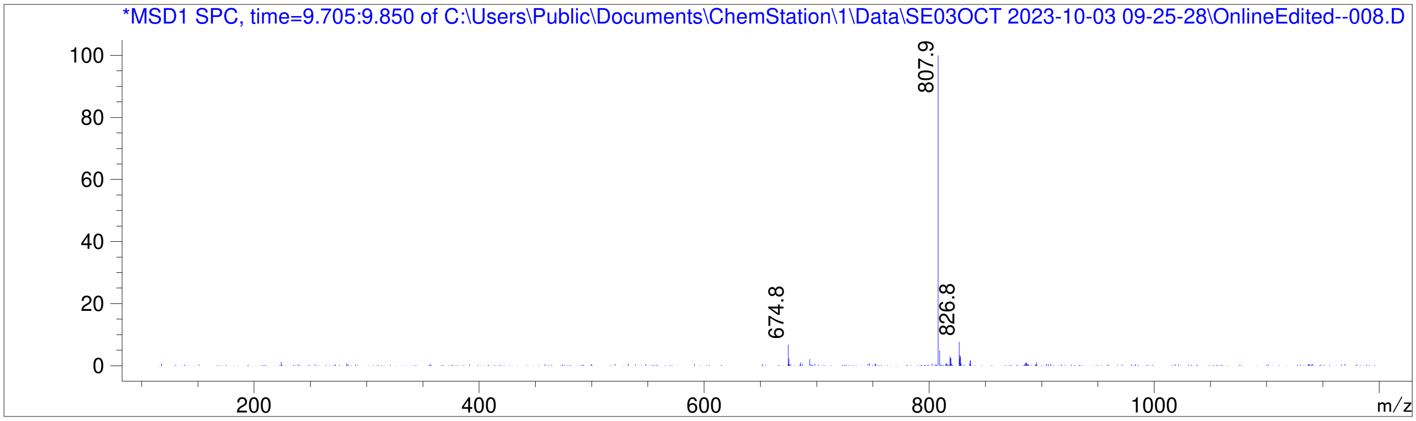
*MSD1 SPC, time=9.705:9.850 of C:\Users\Public\Documents\ChemStation\1\Data\SE03OCT 2023-10-03 09-25-28\OnlineEdited--008.D
807.9
674.8
826.8
m/z

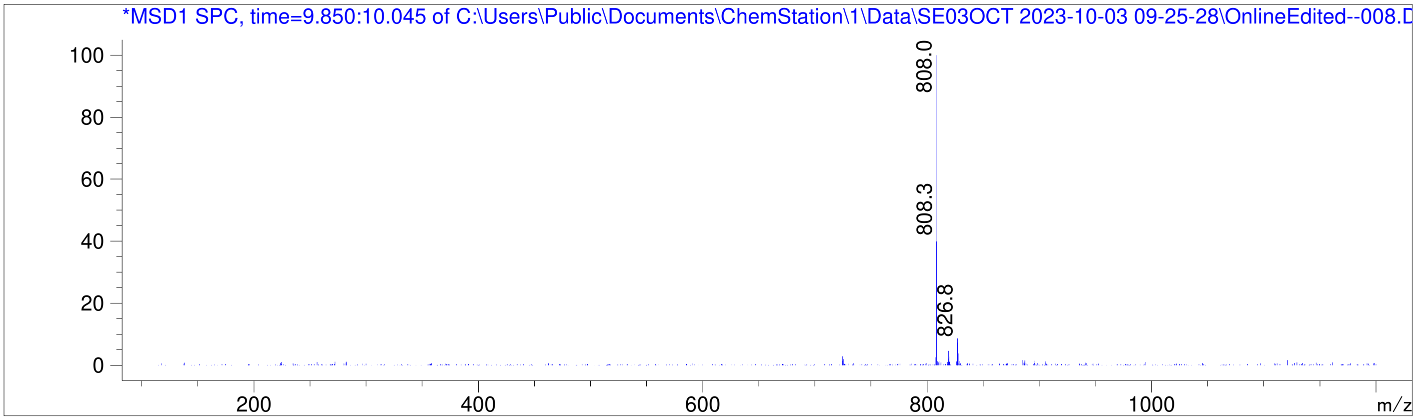
*MSD1 SPC, time=9.850:10.045 of C:\Users\Public\Documents\ChemStation\1\Data\SE03OCT 2023-10-03 09-25-28\OnlineEdited--008.D
808.0
808.3
826.8
m/z

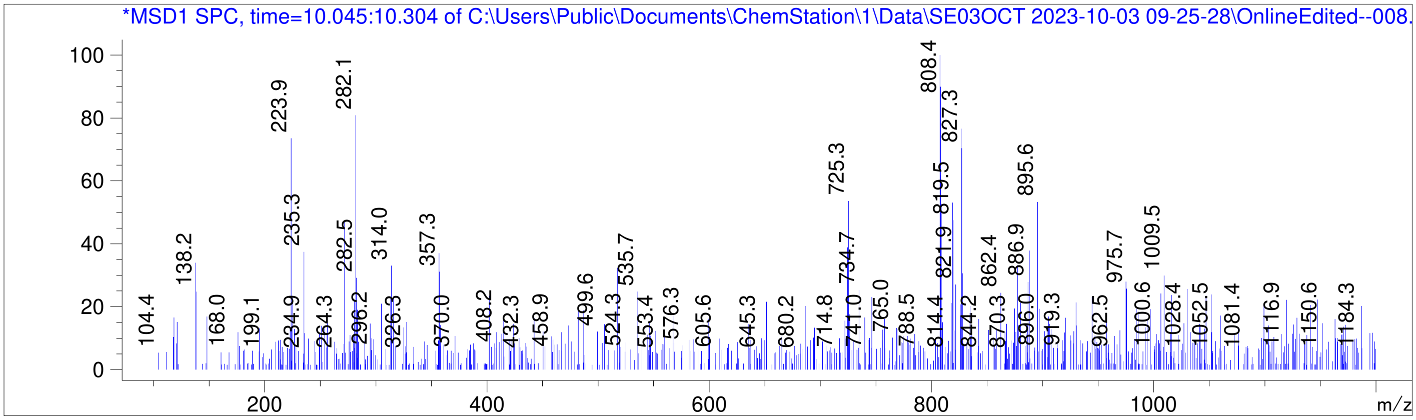
*MSD1 SPC, time=10.045:10.304 of C:\Users\Public\Documents\ChemStation\1\Data\SE03OCT 2023-10-03 09-25-28\OnlineEdited--008.
808.4
282.1
223.9
827.3
725.3
895.6
819.5
235.3
138.2
282.5
314.0
357.3
535.7
734.7
821.9
862.4
886.9
975.7
1009.5
499.6
104.4
168.0
199.1
234.9
264.3
296.2
326.3
370.0
408.2
432.3
458.9
524.3
553.4
576.3
605.6
645.3
680.2
714.8
741.0
765.0
788.5
814.4
844.2
870.3
896.0
919.3
962.5
1000.6
1028.4
1052.5
1081.4
1116.9
1150.6
1184.3
m/z

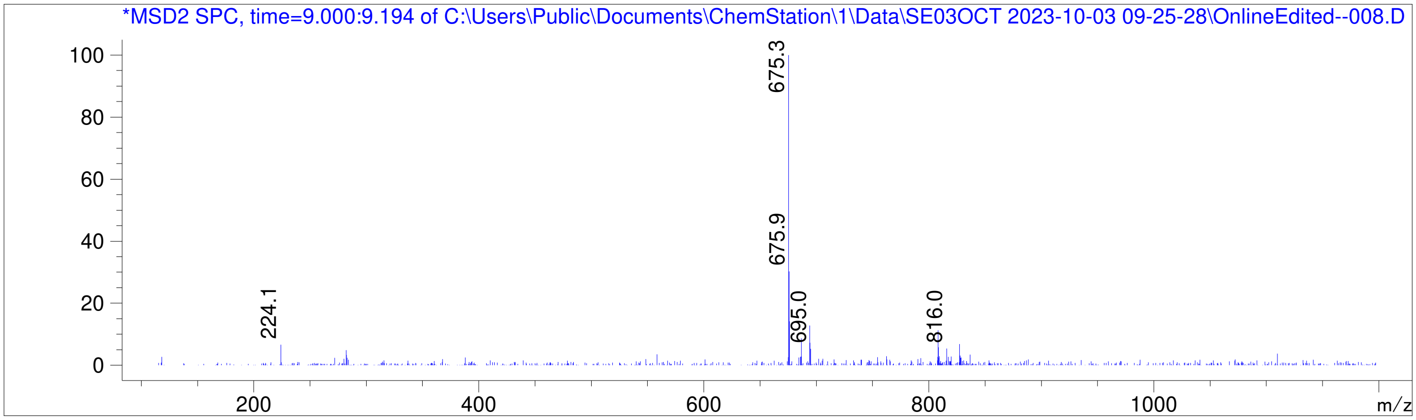
*MSD2 SPC, time=9.000:9.194 of C:\Users\Public\Documents\ChemStation\1\Data\SE03OCT 2023-10-03 09-25-28\OnlineEdited--008.D
675.3
675.9
224.1
695.0
816.0
m/z

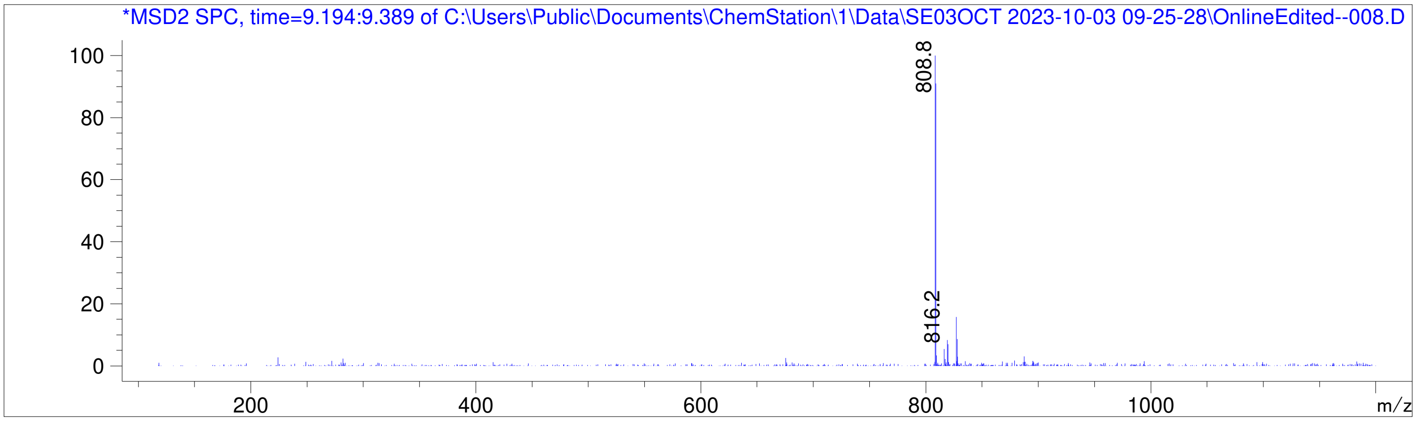
*MSD2 SPC, time=9.194:9.389 of C:\Users\Public\Documents\ChemStation\1\Data\SE03OCT 2023-10-03 09-25-28\OnlineEdited--008.D
808.8
816.2
m/z

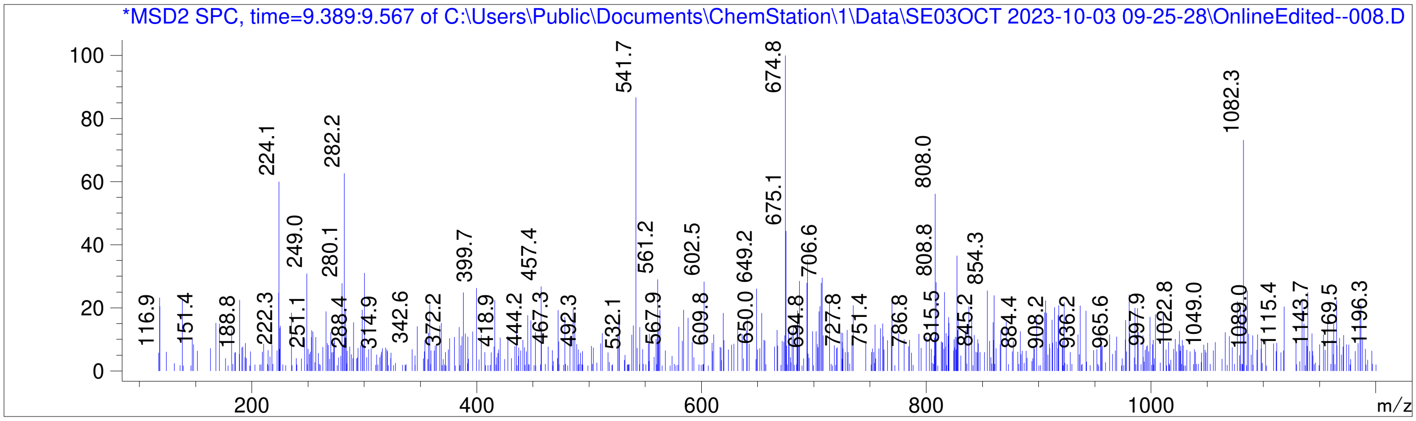
*MSD2 SPC, time=9.389:9.567 of C:\Users\Public\Documents\ChemStation\1\Data\SE03OCT 2023-10-03 09-25-28\OnlineEdited--008.D
541.7
674.8
1082.3
224.1
282.2
808.0
675.1
249.0
280.1
399.7
457.4
561.2
602.5
649.2
706.6
808.8
854.3
m/z

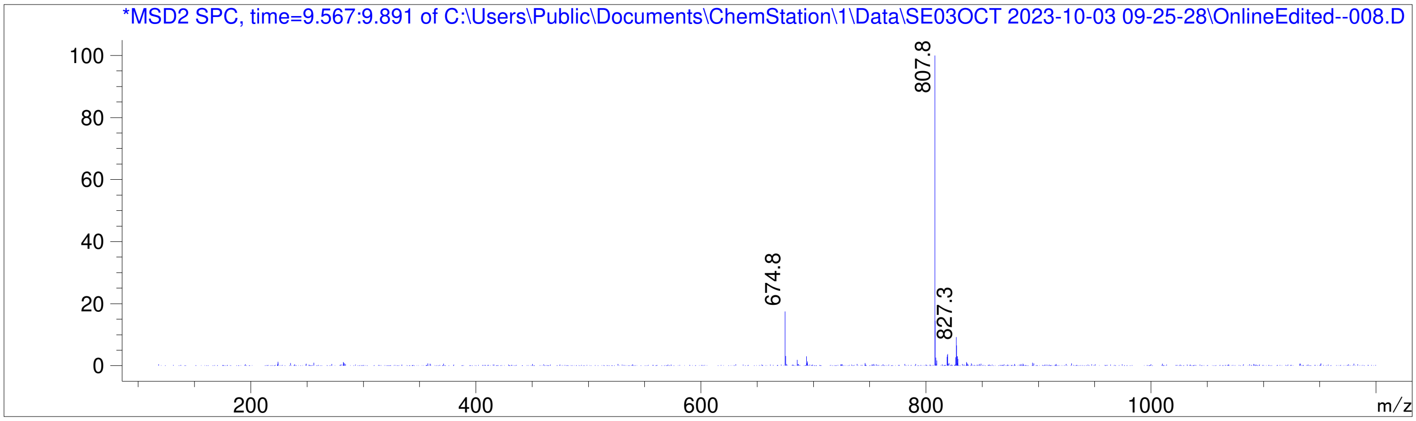
*MSD2 SPC, time=9.567:9.891 of C:\Users\Public\Documents\ChemStation\1\Data\SE03OCT 2023-10-03 09-25-28\OnlineEdited--008.D
807.8
674.8
827.3
m/z

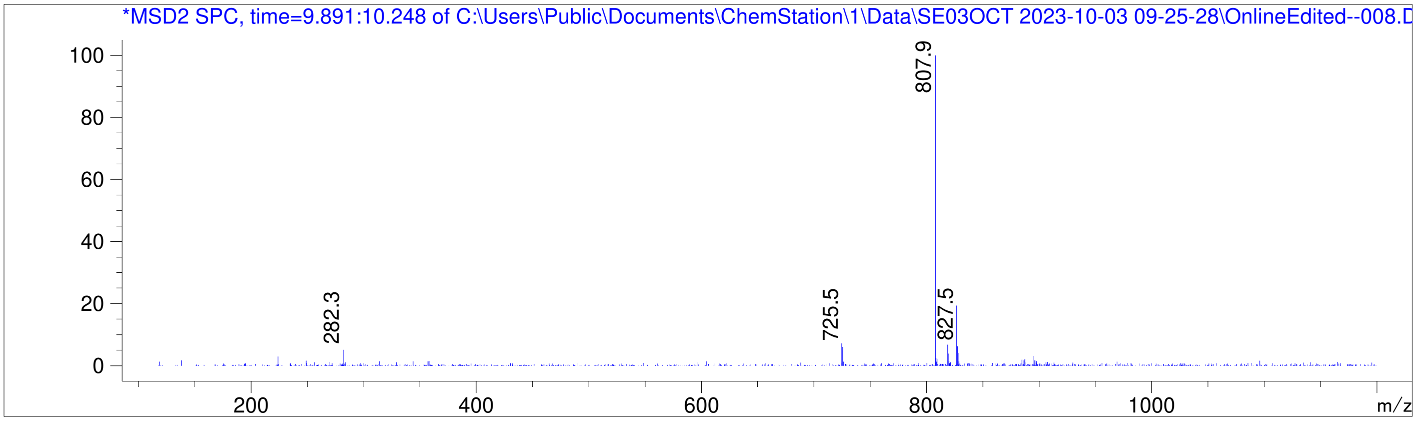
*MSD2 SPC, time=9.891:10.248 of C:\Users\Public\Documents\ChemStation\1\Data\SE03OCT 2023-10-03 09-25-28\OnlineEdited--008.D
807.9
282.3
725.5
827.5
m/z
