# Supplementary material for: Astrobiological implications of the stability and reactivity of peptide nucleic acid (PNA) in concentrated sulfuric acid
Source: Sci Adv. 2025 Mar 26;11(13):eadr0006. doi: 10.1126/sciadv.adr0006 (PMC11939054; doi:10.1126/sciadv.adr0006)
Supplement: Supplementary file 2 — Data S1 and S2 [file sciadv.adr0006_data_s1_and_s2.zip › Supplementary Dataset 1-LCMS DATA/LCMS PNA Hexamers A-T/LCMS T6 RT/24h/CPT22010446-19-D1-24h.pdf]

Data -> C:\Users\Public\Documents\ChemStation\1\Data\SE10OCT 2023-10-10 15-58-00\ ->
Sample-> CPT22010446-19-D1-24h

=====================================================================

| | | | | | |
|---|---|---|---|---|---|
| Injection Date | : | Wed, 11. Oct. 2023 | Seq Line | : | 33 |
| | | | Location | : | 32 |
| | | | Inj. Vol. | : | 2 µl |

Acq. Method : C:\Users\Public\Documents\ChemStation\1\Data\SE10OCT 2023-10-10 15-58-00\22010446C LCMS-6#.M
Analysis Method : C:\Users\Public\Documents\ChemStation\1\Data\SE10OCT 2023-10-10 15-58-00\22010446C LCMS-6#.M (Sequence Method)
Waters XBridge BEH Amide (4.6 x 150 mm, 2.5 µm); PN# 186006726
Mobile Phase A: 20mM Ammonium Acetate (aq) pH 8.2
Mobile Phase B: AcN
Mobile Phase A / Mobile Phase B: 5/95 (0 min) --> (10 min) --> 60/40 (5 min); Flow: 1.0 ml/min; MSD1 = positive; MSD2 = negative

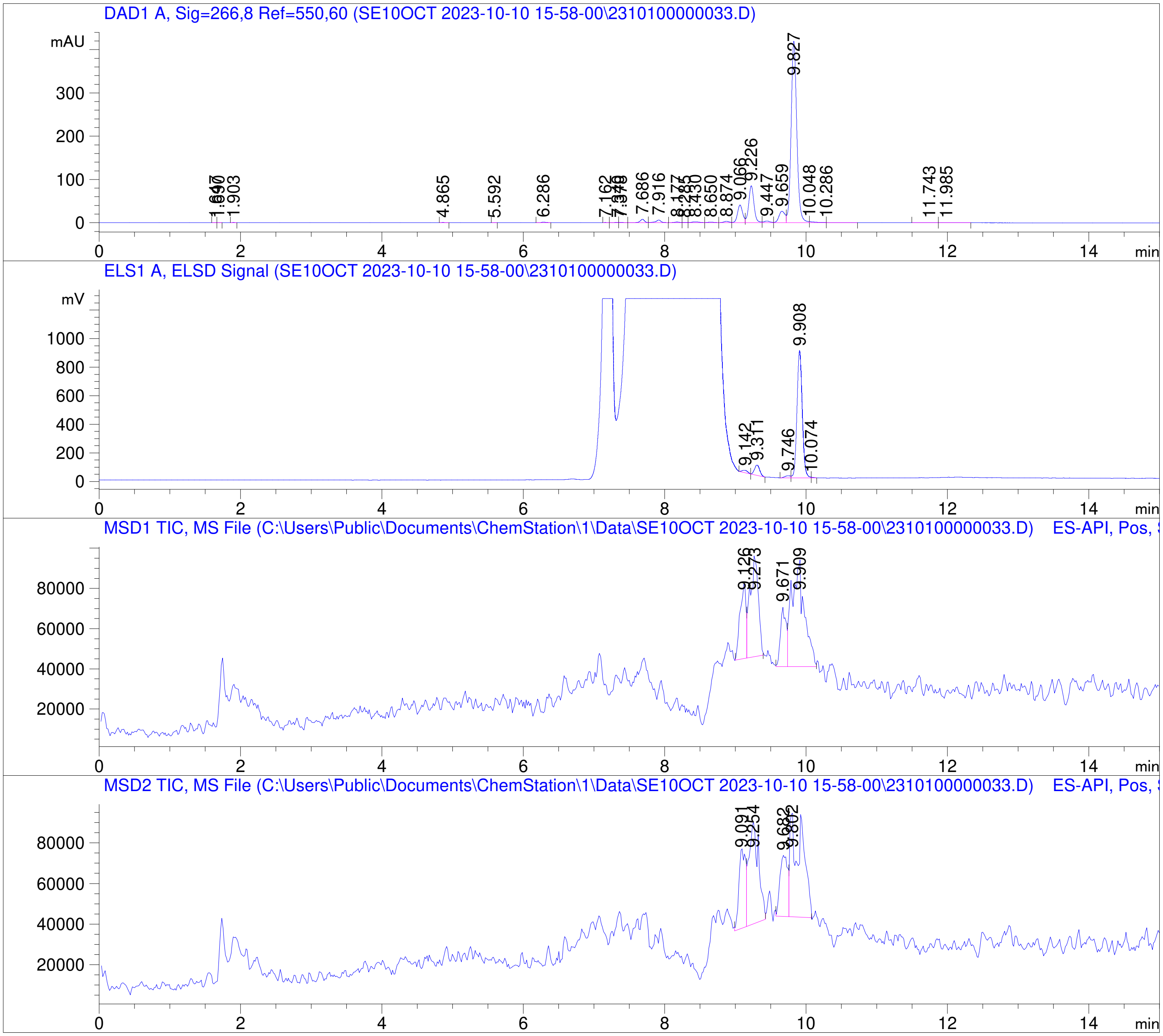

Sample-> CPT22010446-19-D1-24h

DAD1 A, Sig=266,8 Ref=550,60

| Peak # | Ret. Time [min] | Area [mV *s] | Area % |
|---|---|---|---|
| 1 | 1.647 | 0.279 | 0.008 |
| 2 | 1.690 | 0.273 | 0.008 |
| 3 | 1.903 | 1.236 | 0.037 |
| 4 | 4.865 | 1.810 | 0.055 |
| 5 | 5.592 | 0.483 | 0.015 |
| 6 | 6.286 | 5.823 | 0.176 |
| 7 | 7.162 | 1.099 | 0.033 |
| 8 | 7.340 | 2.668 | 0.080 |
| 9 | 7.378 | 2.792 | 0.084 |
| 10 | 7.686 | 33.951 | 1.024 |
| 11 | 7.916 | 30.842 | 0.930 |
| 12 | 8.177 | 7.388 | 0.223 |
| 13 | 8.285 | 2.218 | 0.067 |
| 14 | 8.430 | 13.872 | 0.418 |
| 15 | 8.650 | 7.874 | 0.238 |
| 16 | 8.874 | 16.485 | 0.497 |
| 17 | 9.066 | 206.350 | 6.225 |
| 18 | 9.226 | 432.754 | 13.055 |
| 19 | 9.447 | 22.491 | 0.678 |
| 20 | 9.659 | 159.236 | 4.804 |
| 21 | 9.827 | 2340.502 | 70.608 |
| 22 | 10.048 | 16.178 | 0.488 |
| 23 | 10.286 | 5.671 | 0.171 |
| 24 | 11.743 | 1.306 | 0.039 |
| 25 | 11.985 | 1.213 | 0.037 |

ELS1 A, ELSD Signal

| Peak # | Ret. Time [min] | Area [mV *s] | Area % |
|---|---|---|---|
| 1 | 9.142 | 86.702 | 1.677 |
| 2 | 9.311 | 364.651 | 7.053 |
| 3 | 9.746 | 82.174 | 1.589 |
| 4 | 9.908 | 4627.097 | 89.491 |
| 5 | 10.074 | 9.844 | 0.190 |

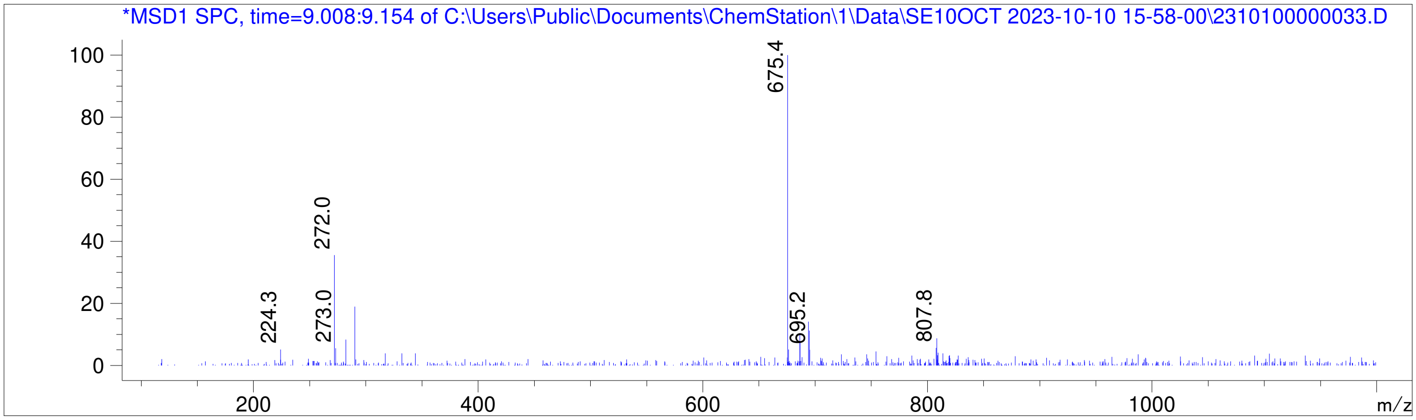
*MSD1 SPC, time=9.008:9.154 of C:\Users\Public\Documents\ChemStation\1\Data\SE10OCT 2023-10-10 15-58-00\2310100000033.D
675.4
272.0
224.3
273.0
695.2
807.8
m/z

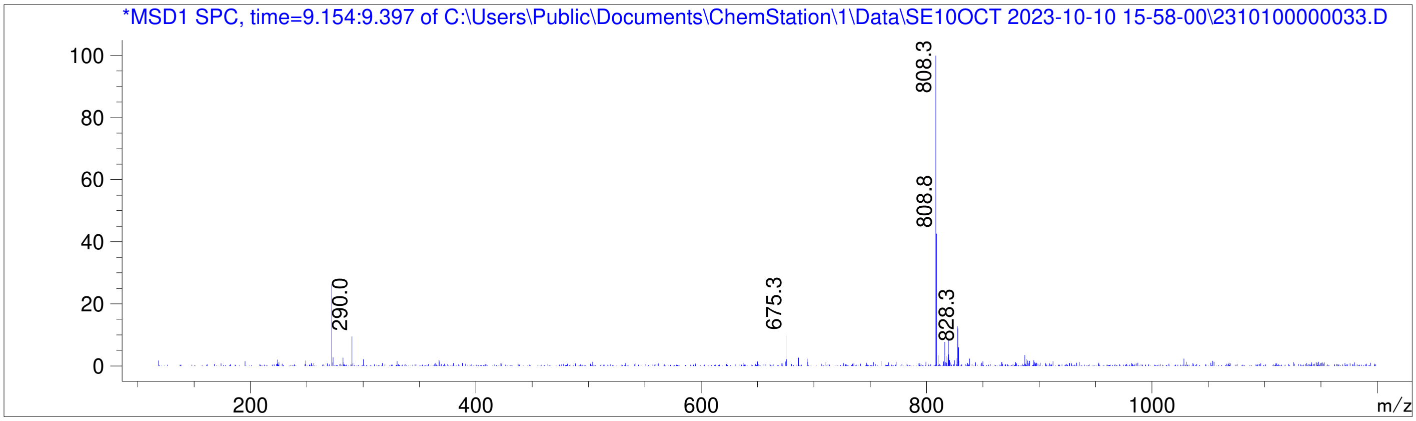
*MSD1 SPC, time=9.154:9.397 of C:\Users\Public\Documents\ChemStation\1\Data\SE10OCT 2023-10-10 15-58-00\2310100000033.D
808.3
808.8
290.0
675.3
828.3
m/z

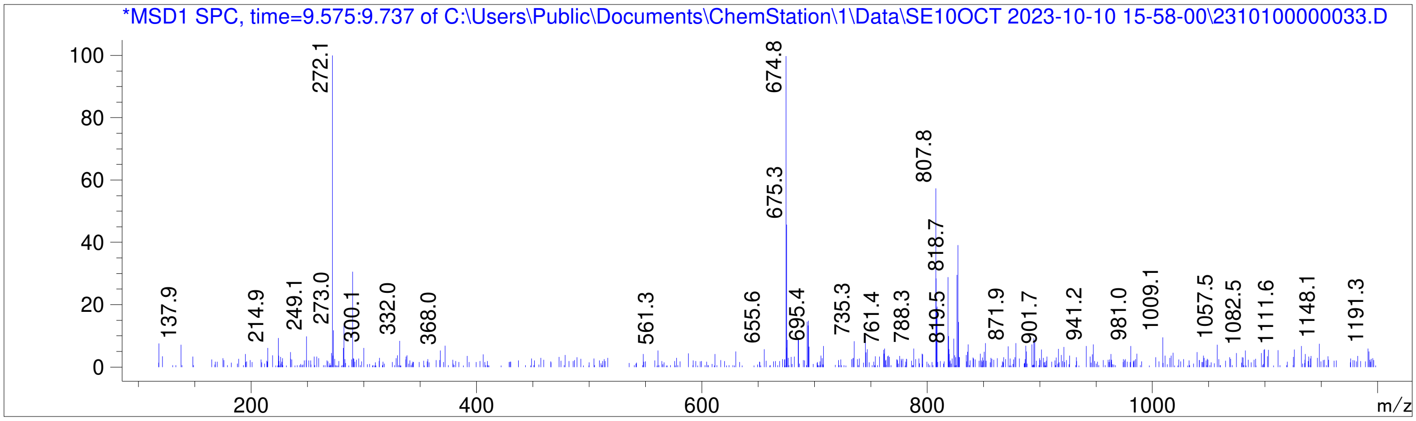
*MSD1 SPC, time=9.575:9.737 of C:\Users\Public\Documents\ChemStation\1\Data\SE10OCT 2023-10-10 15-58-00\2310100000033.D
272.1
674.8
807.8
675.3
818.7
137.9
214.9
249.1
273.0
300.1
332.0
368.0
561.3
655.6
695.4
735.3
761.4
788.3
819.5
871.9
901.7
941.2
981.0
1009.1
1057.5
1082.5
1111.6
1148.1
1191.3
m/z

==========================================================================

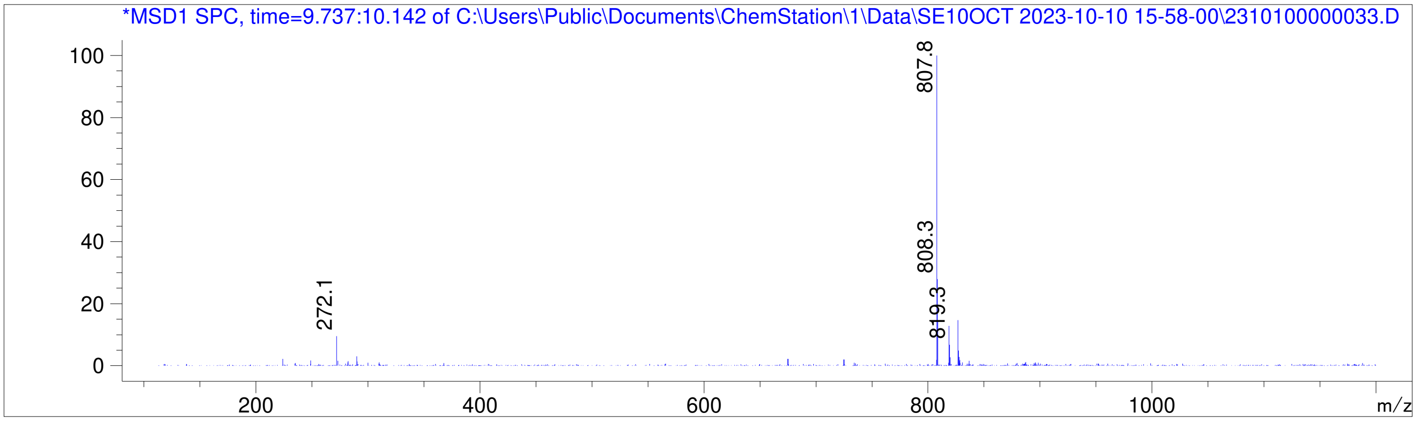
*MSD1 SPC, time=9.737:10.142 of C:\Users\Public\Documents\ChemStation\1\Data\SE10OCT 2023-10-10 15-58-00\2310100000033.D
807.8
808.3
819.3
272.1
0
20
40
60
80
100
200
400
600
800
1000
m/z

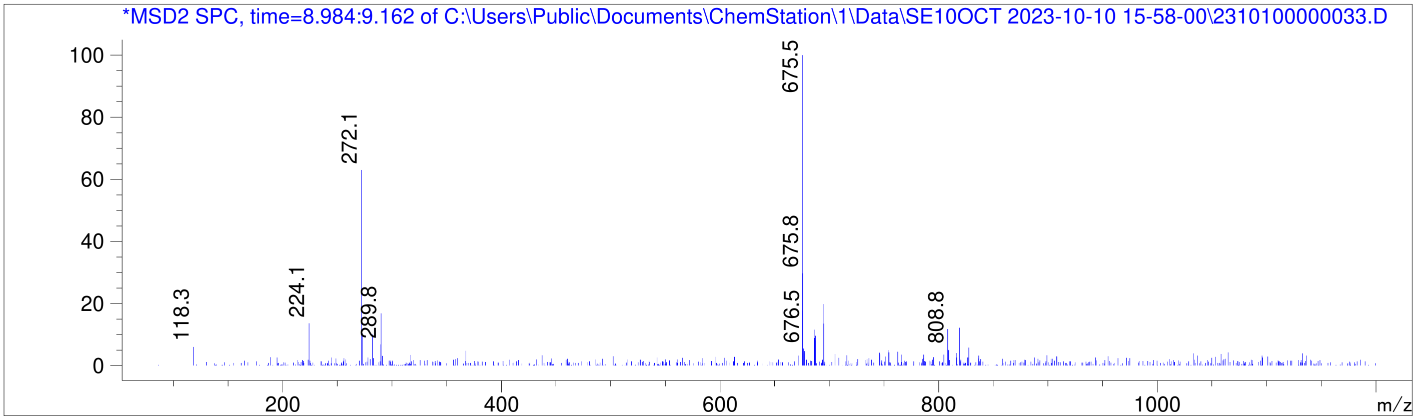
*MSD2 SPC, time=8.984:9.162 of C:\Users\Public\Documents\ChemStation\1\Data\SE10OCT 2023-10-10 15-58-00\2310100000033.D
675.5
272.1
675.8
118.3
224.1
289.8
676.5
808.8
m/z

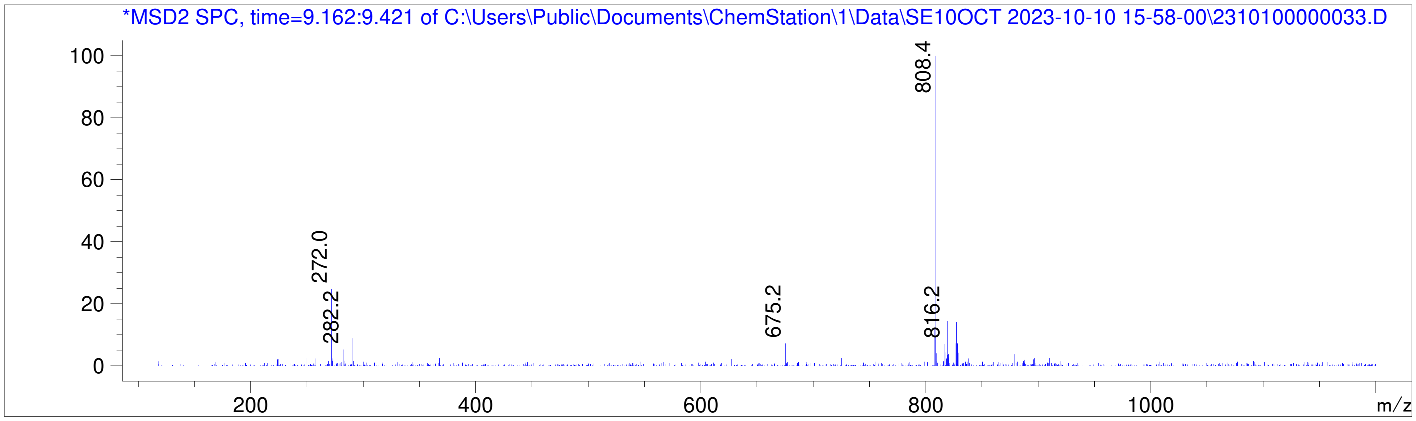
*MSD2 SPC, time=9.162:9.421 of C:\Users\Public\Documents\ChemStation\1\Data\SE10OCT 2023-10-10 15-58-00\2310100000033.D
808.4
272.0
282.2
675.2
816.2
m/z

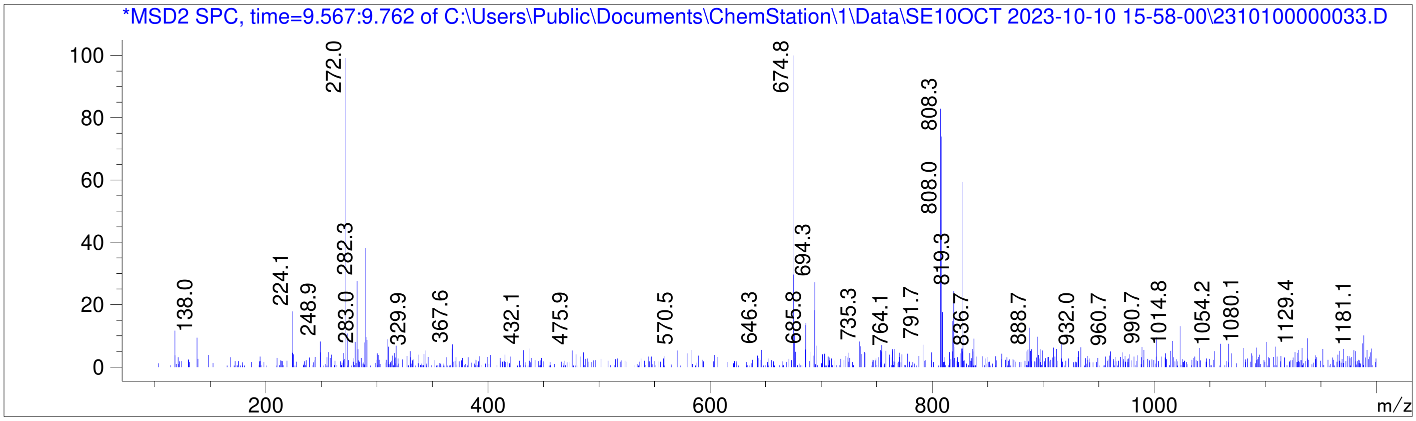
*MSD2 SPC, time=9.567:9.762 of C:\Users\Public\Documents\ChemStation\1\Data\SE10OCT 2023-10-10 15-58-00\2310100000033.D
272.0
674.8
808.3
808.0
282.3
694.3
819.3
138.0
224.1
248.9
283.0
329.9
367.6
432.1
475.9
570.5
646.3
685.8
735.3
764.1
791.7
836.7
888.7
932.0
960.7
990.7
1014.8
1054.2
1080.1
1129.4
1181.1
m/z

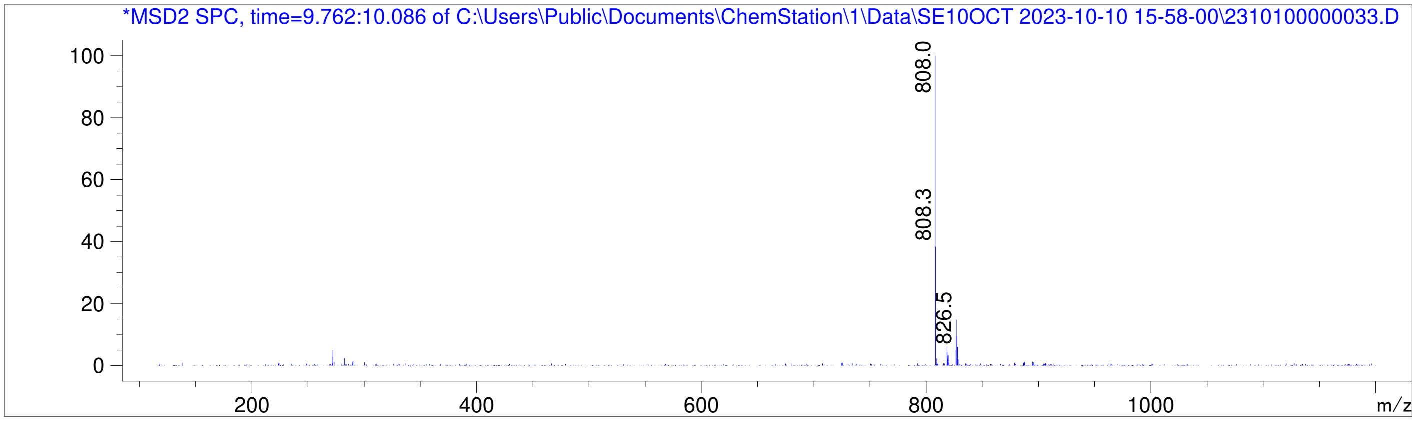
*MSD2 SPC, time=9.762:10.086 of C:\Users\Public\Documents\ChemStation\1\Data\SE10OCT 2023-10-10 15-58-00\2310100000033.D
808.0
808.3
826.5
100
80
60
40
20
0
200
400
600
800
1000
m/z
