# Supplementary material for: Astrobiological implications of the stability and reactivity of peptide nucleic acid (PNA) in concentrated sulfuric acid
Source: Sci Adv. 2025 Mar 26;11(13):eadr0006. doi: 10.1126/sciadv.adr0006 (PMC11939054; doi:10.1126/sciadv.adr0006)
Supplement: Supplementary file 2 — Data S1 and S2 [file sciadv.adr0006_data_s1_and_s2.zip › Supplementary Dataset 1-LCMS DATA/LCMS PNA Hexamers A-T/LCMS T6 RT/24h/LCMS-6_CPT22010446-19-D2-24h.pdf]

Data -> C:\Users\Public\Documents\ChemStation\1\Data\Se10Oct\SE10OCT 2023-10-10 15-58-->
Sample-> CPT22010446-19-D2-24h

===

Injection Date	: Wed, 11. Oct. 2023		Seq Line	: 35
			Location	: 34
			Inj. Vol.	: 2 µl

Acq. Method : C:\Users\Public\Documents\ChemStation\1\Data\SE10OCT 2023-10-10 15-58-00\22010446C LCMS-6#.M

Analysis Method : C:\Users\Public\Documents\ChemStation\1\Data\Se10Oct\SE10OCT 2023-10-10 15-58-00\22010446C LCMS-6#.M (Sequence Method)

Waters XBridge BEH Amide (4.6 x 150 mm, 2.5 µm); PN# 186006726

Mobile Phase A: 20mM Ammonium Acetate (aq) pH 8.2

Mobile Phase B: AcN

Mobile Phase A / Mobile Phase B: 5/95 (0 min) --> (10 min) --> 60/40 (5 min); Flow: 1.0 ml/min; MSD1 = positive; MSD2 = negative

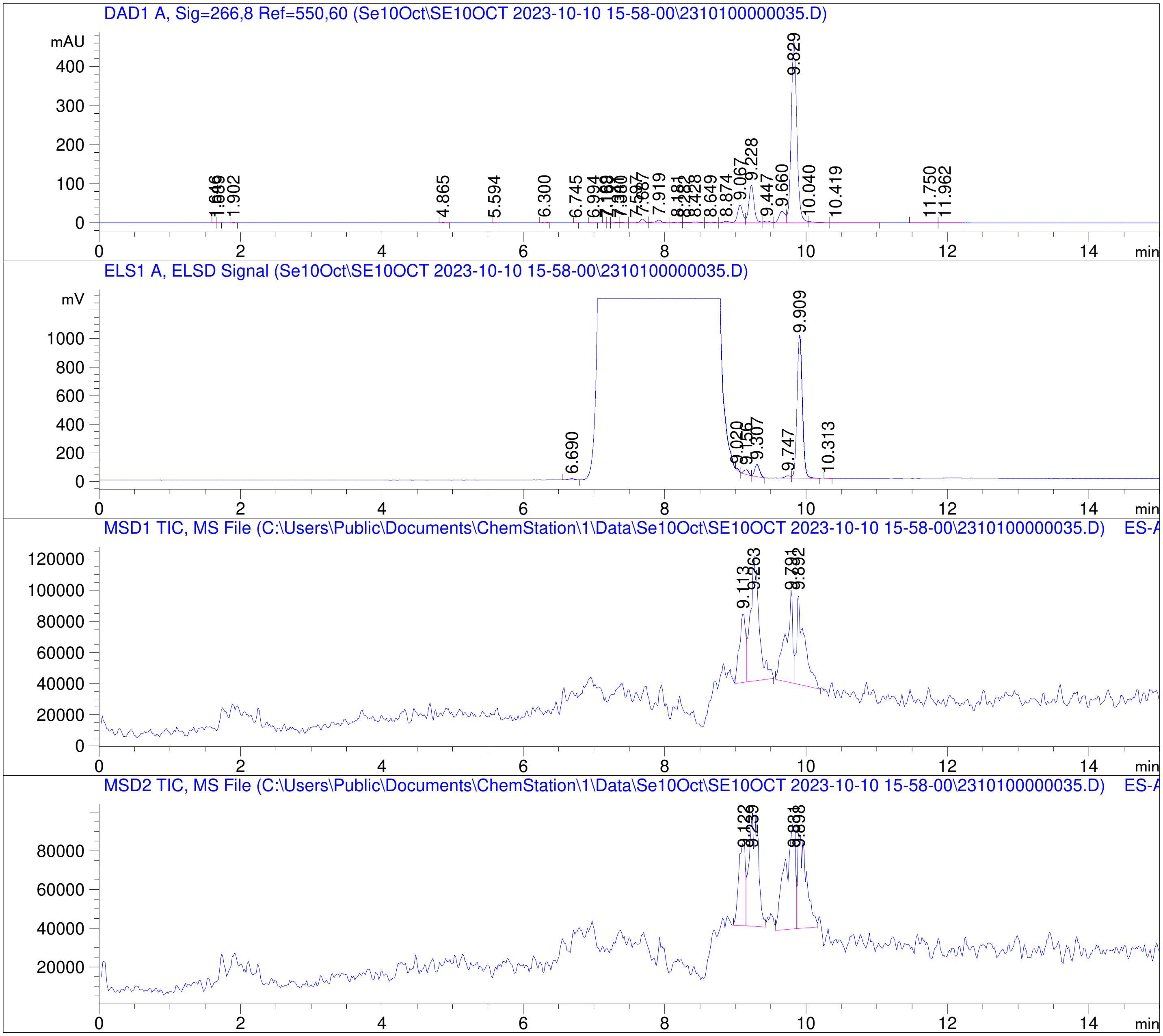

DAD1 A, Sig=266,8 Ref=550,60

Peak #	Ret. Time [min]	Area [mV *s]	Area %
1	1.646	0.244	0.007
2	1.689	0.320	0.009
3	1.902	1.621	0.043
4	4.865	2.030	0.054
5	5.594	0.538	0.014
6	6.300	5.345	0.143
7	6.745	0.111	0.003
8	6.994	0.350	0.009
9	7.169	0.523	0.014
10	7.188	0.852	0.023
11	7.341	3.477	0.093
12	7.380	3.567	0.096
13	7.597	1.935	0.052
14	7.687	38.737	1.037
15	7.919	38.481	1.031
16	8.181	11.161	0.299
17	8.282	3.905	0.105
18	8.428	18.482	0.495
19	8.649	11.333	0.304
20	8.874	20.898	0.560
21	9.067	233.111	6.243
22	9.228	493.402	13.214
23	9.447	27.666	0.741
24	9.660	178.168	4.772
25	9.829	2607.112	69.822
26	10.040	21.187	0.567
27	10.419	7.005	0.188
28	11.750	1.352	0.036
29	11.962	1.026	0.027

ELS1 A, ELSD Signal

Peak #	Ret. Time [min]	Area [mV *s]	Area %
1	6.690	44.115	0.735
2	9.020	27.563	0.459
3	9.156	155.866	2.596
4	9.307	411.123	6.848
5	9.747	100.864	1.680
6	9.909	5257.587	87.575
7	10.313	6.394	0.107

Data -> C:\Users\Public\Documents\ChemStation\1\Data\Se10Oct\SE10OCT 2023-10-10 15-58-->
Sample-> CPT22010446-19-D2-24h
==

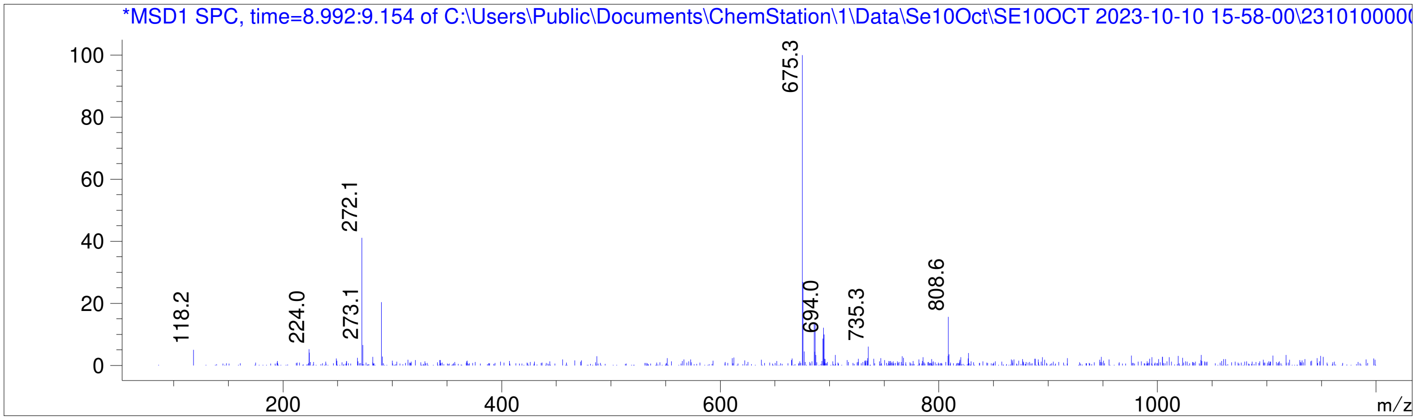

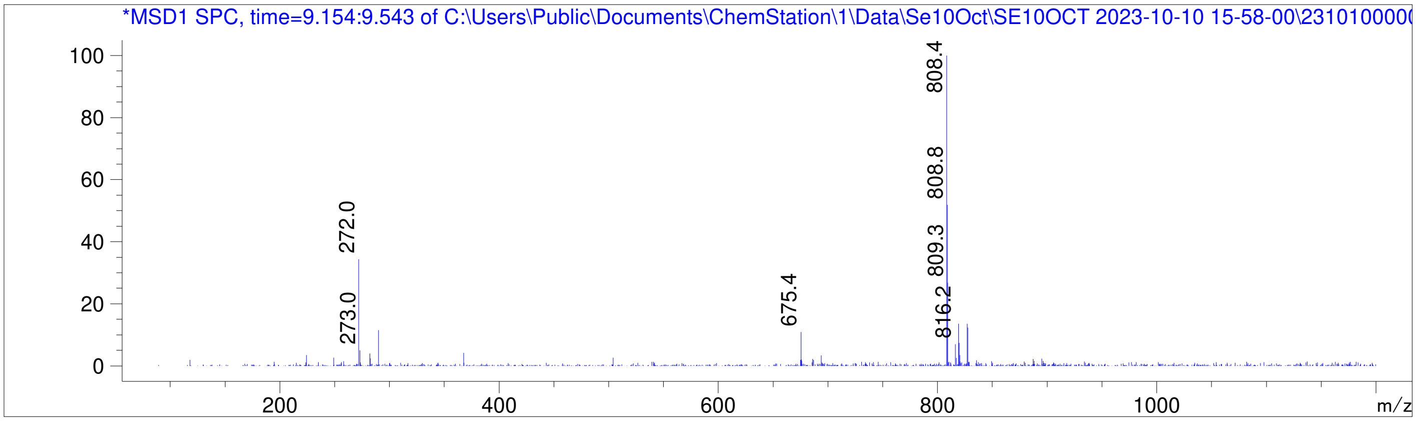

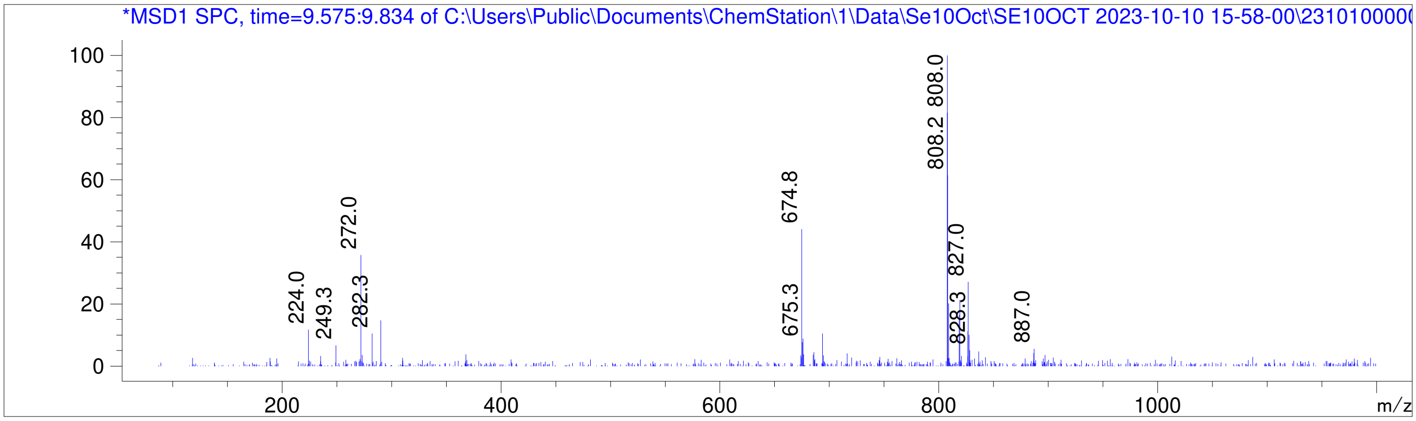

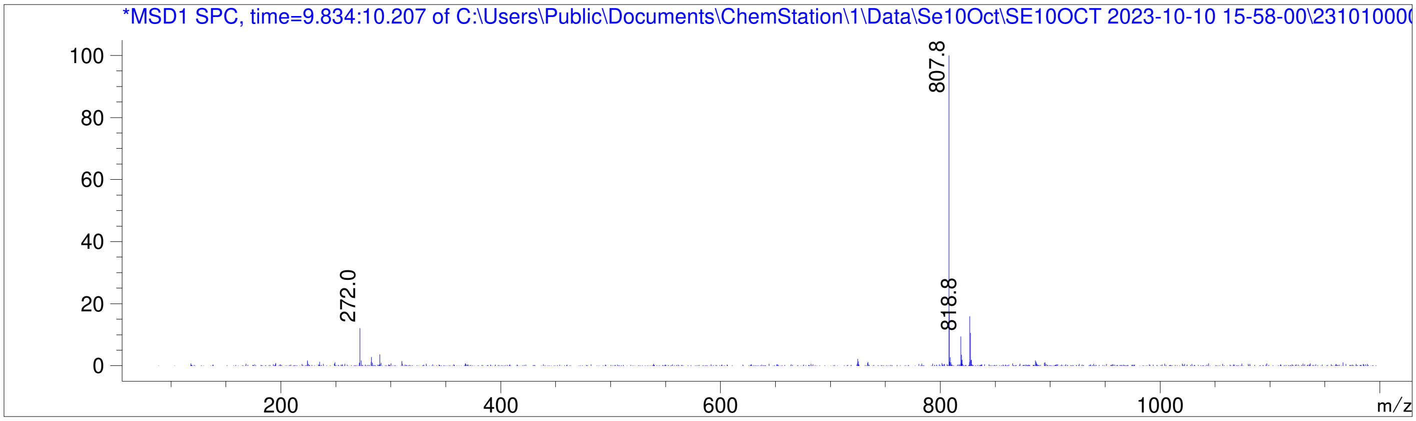
*MSD1 SPC, time=9.834:10.207 of C:\Users\Public\Documents\ChemStation\1\Data\Se10Oct\SE10OCT 2023-10-10 15-58-00\23101000(
100
80
60
40
20
0
807.8
272.0
818.8
200
400
600
800
1000
m/z

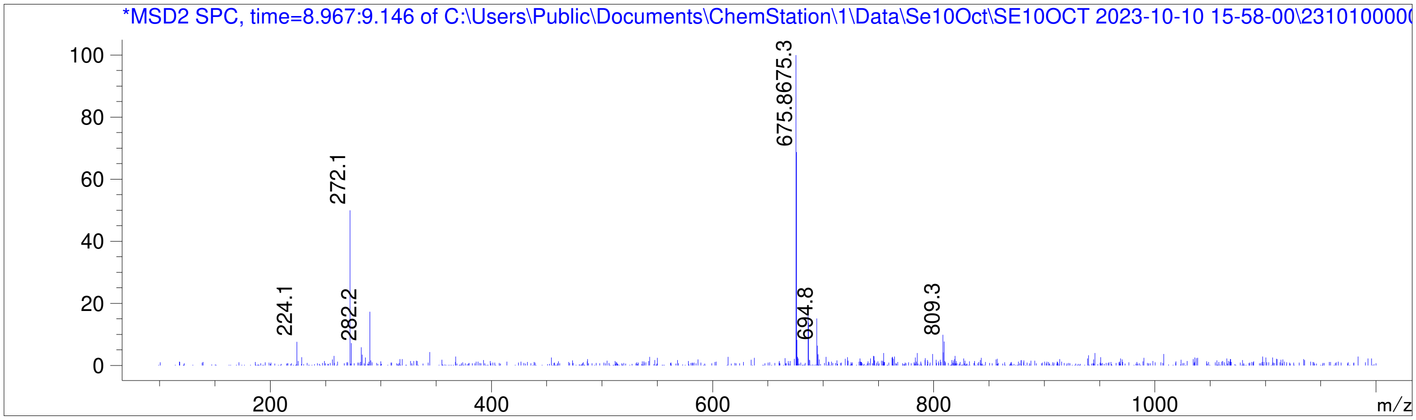
*MSD2 SPC, time=8.967:9.146 of C:\Users\Public\Documents\ChemStation\1\Data\Se10Oct\SE10OCT 2023-10-10 15-58-00\2310100000
675.8675.3
272.1
224.1
282.2
694.8
809.3
m/z

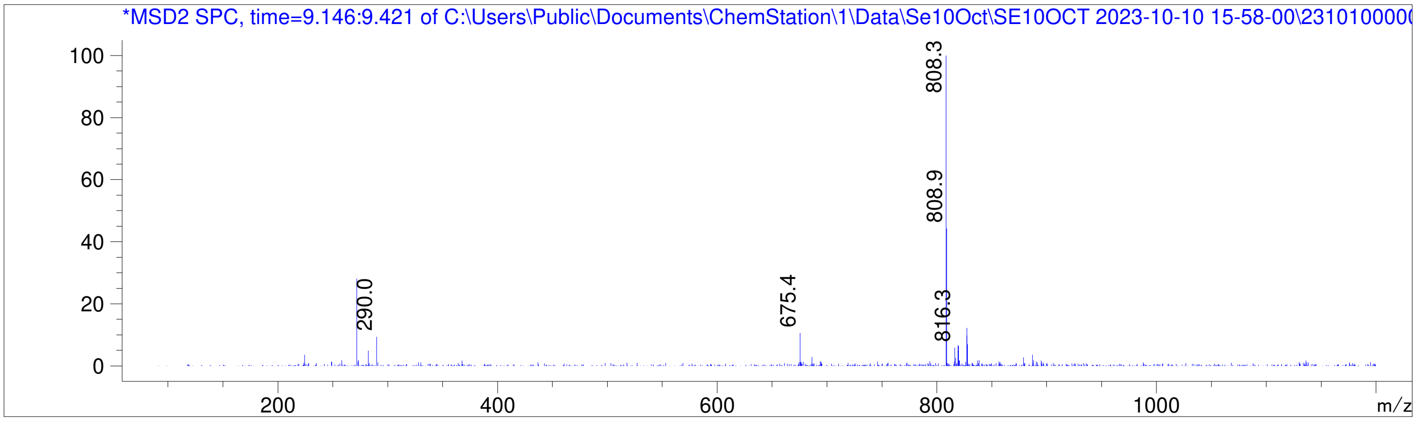
*MSD2 SPC, time=9.146:9.421 of C:\Users\Public\Documents\ChemStation\1\Data\Se10Oct\SE10OCT 2023-10-10 15-58-00\2310100000
808.3
808.9
290.0
675.4
816.3
m/z

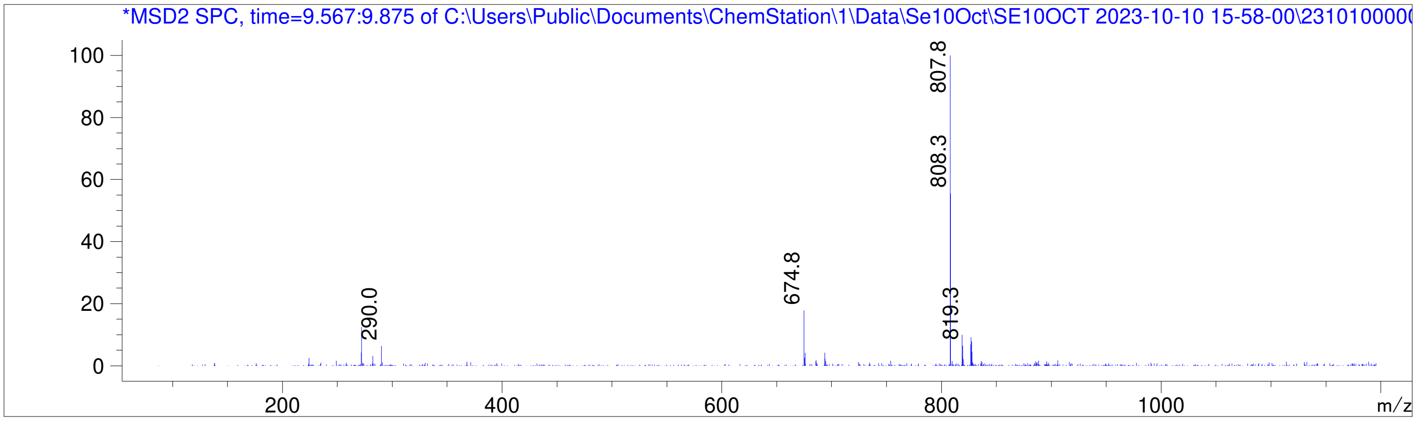
*MSD2 SPC, time=9.567:9.875 of C:\Users\Public\Documents\ChemStation\1\Data\Se10Oct\SE10OCT 2023-10-10 15-58-00\2310100000
807.8
808.3
674.8
290.0
819.3
m/z

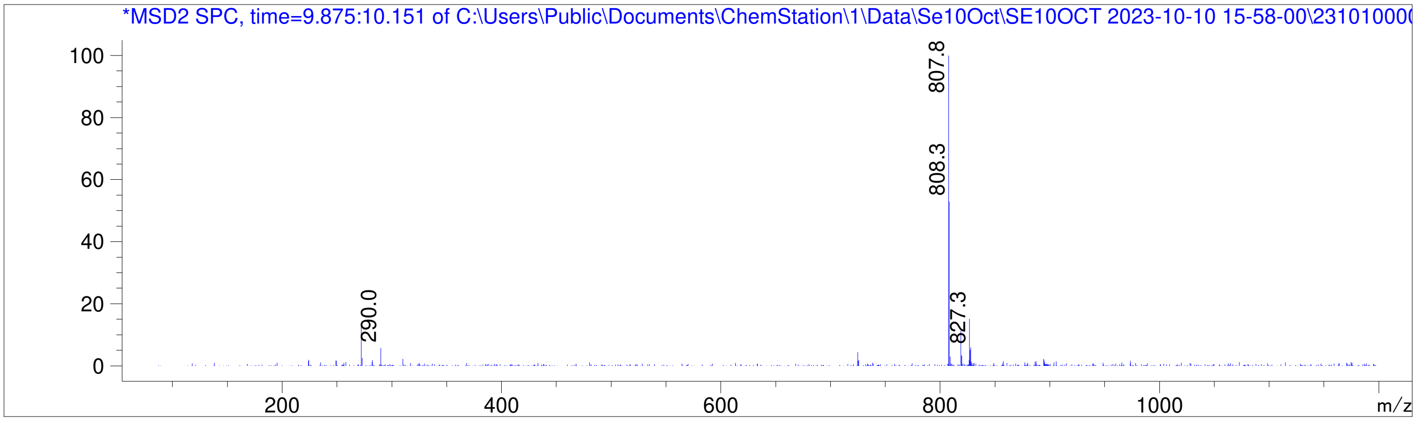
*MSD2 SPC, time=9.875:10.151 of C:\Users\Public\Documents\ChemStation\1\Data\Se10Oct\SE10OCT 2023-10-10 15-58-00\23101000(
100
80
60
40
20
0
807.8
808.3
290.0
827.3
200
400
600
800
1000
m/z
